# Aspartyl protease in the secretome of honey bee trypanosomatid parasite contributes to infection of bees

**DOI:** 10.1186/s13071-024-06126-7

**Published:** 2024-02-10

**Authors:** Xuye Yuan, Jianying Sun, Tatsuhiko Kadowaki

**Affiliations:** Department of Biological Sciences, School of Science, Xi’an Jiaotong-Liverpool University, 111 Ren’ai Road, Suzhou Dushu Lake Higher Education Town, 215123 Jiangsu China

**Keywords:** Exoproteome, Secretome, Trypanosomatid parasite, *Lotmaria passim*, Aspartyl protease, Chitinase

## Abstract

**Background:**

The exoproteome, which consists of both secreted proteins and those originating from cell surfaces and lysed cells, is a critical component of trypanosomatid parasites, facilitating interactions with host cells and gut microbiota. However, its specific roles in the insect hosts of these parasites remain poorly understood.

**Methods:**

We conducted a comprehensive characterization of the exoproteome in *Lotmaria passim*, a trypanosomatid parasite infecting honey bees, under culture conditions. We further investigated the functions of two conventionally secreted proteins, aspartyl protease (LpAsp) and chitinase (LpCht), as representative models to elucidate the role of the secretome in *L. passim* infection of honey bees.

**Results:**

Approximately 48% of *L. passim* exoproteome proteins were found to share homologs with those found in seven *Leishmania* spp., suggesting the existence of a core exoproteome with conserved functions in the Leishmaniinae lineage. Bioinformatics analyses suggested that the *L. passim* exoproteome may play a pivotal role in interactions with both the host and its microbiota. Notably, the deletion of genes encoding two secretome proteins revealed the important role of LpAsp, but not LpCht, in *L. passim* development under culture conditions and its efficiency in infecting the honey bee gut.

**Conclusions:**

Our results highlight the exoproteome as a valuable resource for unraveling the mechanisms employed by trypanosomatid parasites to infect insect hosts by interacting with the gut environment.

**Graphical Abstract:**

**Figure:**
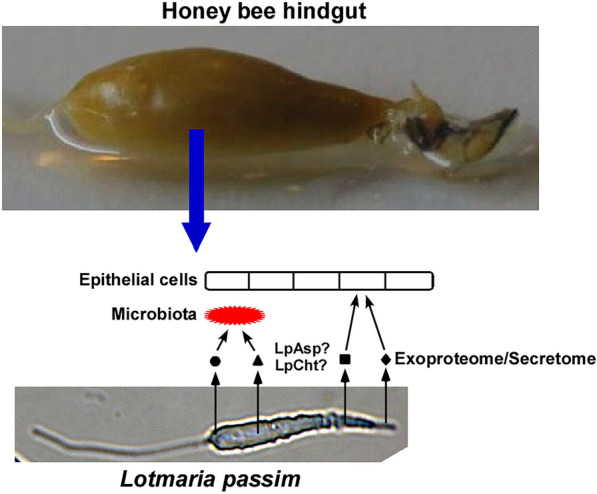

**Supplementary Information:**

The online version contains supplementary material available at 10.1186/s13071-024-06126-7.

## Background

The collection of proteins residing outside a cell, either within a conditioned medium or in the extracellular matrix, is widely recognized as the exoproteome [1]. This exoproteome constitutes both actively secreted proteins and as non-secreted proteins originating from cell surface constituents and lysed cells. A subset of the exoproteome, termed the secretome, comprises proteins conventionally and non-conventionally secreted [2]. Conventionally secreted proteins are transported through a traditional secretory pathway, such as from the endoplasmic reticulum (ER) to the Golgi apparatus and then to the plasma membrane. These proteins contain a N-terminal signal peptide (approx. 20-amino acid peptides, many of which are hydrophobic), which is essential for transport to ER lumen. The lack of an internal transmembrane domain ensures that the protein is not anchored to a membrane. Notably, non-conventionally secreted proteins, including those associated with extracellular vesicles (EVs) or exosomes, have garnered considerable attention in various organisms [3, 4]. Exosomes, which are laden with proteins, nucleic acids and lipids, play pivotal roles in intercellular communication over both short and long distances. The content of exosomes is dynamic, influenced by cell type and physiological conditions, and exosomes are associated with anti-inflammatory and anti-microbial activities [5].

Exoproteomes have also been characterized in trypanosomatid parasites, including *Leishmania* spp., which has facilitated the identification of proteins central to parasite survival, pathogenesis and other biologically significant processes. Pissarra et al. [6] conducted exoproteomic profiling across seven *Leishmania* species, revealing a shared core set of proteins alongside species-specific ones. These findings underscore the exoproteome's dual nature, with conserved functions for host adaptation and specialized roles in species-specific pathogenesis. For example, *Leishmania major* was shown to secrete exosomes under both culture conditions and within the sand fly midgut, exacerbating disease pathology by inducing excessive inflammatory cytokine production in mammalian hosts [7]. Nevertheless, the roles of exosomes in insect vectors of parasitic infections remain unexplored. Among the conventionally secreted proteins, chitinase was assessed in *Leishmania mexicana* during sand fly infection, with the authors reporting that ectopic expression enhanced survival and growth by facilitating escape from the peritrophic membrane (PM) and colonization of the stomodeal valve [8]. The PM not only protects gut parasites, such as *Leishmania* spp., from digestive enzymes in the midgut [9] but also acts as a physical barrier to further development [10].

*Lotmaria passim* is the most prevalent trypanosomatid parasite infecting honey bees across the globe [11–14]. It specifically colonizes the honey bee hindgut, affecting host physiology, and may be associated with winter colony loss [15, 16]. A related species, *Crithidia bombi* [17], infects the bumble bee gut and exerts various detrimental effects on host performance [18, 19]. Both of these species are monoxenous parasites that exclusively infect bees as hosts, yet the mechanisms governing their establishment and maintenance within hosts remain incompletely understood.

To elucidate factors within *L. passim* that interact with the honey bee hindgut environment, we first characterized the exoproteome of promastigotes under culture conditions. Subsequently, we investigated two conventionally secreted hydrolytic enzymes, aspartyl protease (LpAsp) and chitinase (LpCht), as models to determine the influence of the secretome on *L. passim* infection in honey bees. In a previous study, genes associated with proteolysis were significantly upregulated in *L. passim* during honey bee infection, and *LpAsp* mRNA levels increased notably between days 12 and 27 post-infection [16]. LpCht, on the other hand, is of particular interest due to its potential role in the release of *L. passim* from the PM and its ability to modify the hindgut cuticle lining [20]. Our hypothesis posited that these enzymes are non-essential for *L. passim* growth under culture conditions, thereby allowing viable parasites with loss-of-function mutations. However, we anticipated that such gene mutations could significantly impact infection in honey bees. The overall aim of our study was to shed light on the intricate interplay between a gut parasite and its insect host through the secretome.

## Methods

### Characterization of the *L. passim* exoproteome

*Lotmaria passim* promastigotes (ATCC PRA-403; American Type Culture Collection, Manassas, VA, USA) were cultivated in 30 ml of Insectagro® DS2 serum-free/protein-free medium (Corning Inc., Corning, NY, USA). Cultures were maintained at 28 °C in culture flasks until the early stationary phase (2 × 10^7^/ml) was reached. The viability of parasites, which exceeded 94%, was assessed by propidium iodide (Sigma-Aldrich, St. Louis, MO, USA) staining followed by flow cytometry analysis.

Conditioned culture medium was collected by centrifugation at 1000 *g* for 10 min, followed by filtration through a 0.2-μm pore-size filter (MilliporeSigma, Burlington, MA, USA). The filtered medium was subsequently concentrated using a Vivaspin 20 centrifugal device (3-kDa cut-off; Sartorius AG, Göttingen, Germany). Protein concentration was determined using a BCA protein assay kit (Beyotime, Shanghai, China) and adjusted to 1 mg/ml with a solution containing 100 mM ammonium bicarbonate and 5% acetonitrile. For protein digestion, 50-μg aliquots of proteins were first reduced with 10 mM dithiothreitol at 95 °C for 5 min, followed by alkylation with 55 mM iodoacetamide at room temperature for 20 min in the dark. Trypsin (1 μg) was added for overnight digestion at 37 °C, followed by desalting using a spin column (MonoSpin C18; GL Sciences, Shinjuku-ku, Japan) and then drying. Subsequently, 2-μg aliquots of proteins in 0.1% formic acid (at a concentration of 0.1 μg/μl) were analyzed by mass spectrometry (LTQ Orbitrap Elite mass spectrometer; Thermo Fisher Scientific, Waltham, MA, USA) coupled with an Easy-nLC 1000 liquid chromatography system (Thermo Fisher Scientific) equipped with a nanoelectrospray source and operated in data-dependent acquisition (DDA) mode at the following settings: spray voltage, 2000 V; s-lens RF level, 60%; capillary temperature, 275 °C; scans 300–2000 *m/z*. Peptides were separated using a 15-cm analytical RSLC column (Acclaim™ PepMap™ 100 C18; pore size, 2 µm; length, 150 mm; i.d., 50 µm). The mobile phase consisted of 0.1% formic acid in water (A) and of 0.1% formic acid in acetonitrile (B), and the gradient elution was 0–2 min (95% A-5% B) → 152 min (70% A-30% B) → 162 min (10% A-90% B) → 172 min (10% A-90% B) → 182 min (95% A-5% B) → 187 min (95% A-5% B), at a flow rate of 300 nl/min. The 10 most intense ions from the full scan were selected for tandem mass spectrometry. The normalized collision energy was 35 V, and the default charge state was 2 in higher-energy collisional dissociation (HCD) mode. Scans were collected in a positive polarity mode. Each sample was analyzed using the MaxQuant (version: 2.2.0.0) software package, and the parameters were: Fragment tolerance, 20 ppm (monoisotopic); Fixed modifications, +57 on C (carbamidomethyl); Variable modifications, +16 on M (oxidation) and +42 on peptide N-terminal (acetyl); Database, 9339 *L. passim* annotated proteins (Additional file 1: Dataset S1) [16]; Digestion enzyme, trypsin; and Max missed cleavages, 2.

The functional annotation of *L. passim* exoproteome proteins was conducted using eggNOG-mapper v2 [21]. Annotation databases for Gene Ontology (GO) and Kyoto Encyclopedia of Genes and Genomes (KEGG) pathways were built by mapping annotated *L. passim* proteins to the KEGG [22] Orthology database using the R package AnnotationForge tool [23]. GO and KEGG pathway enrichment analyses against all annotated *L. passim* proteins were performed using the clusterProfiler package [24] of the R software. Additionally, 94 *L. passim* exoproteome proteins were compared to 306 exoproteome proteins common among seven *Leishmania* spp. [6] using reciprocal BLASTP, revealing 67 protein pairs with e-values < 6E−03, of which 45 were considered homologs with e-values < 1E−50.

### Identification of conventionally secreted *L. passim* proteins

To identify conventionally secreted proteins, 301 proteins with N-terminal signal peptides (SPs) were initially identified in annotated *L. passim* proteins using SignalP v5.0 [25]. Subsequently, proteins with transmembrane domains were filtered out using TMHMM v2.0 [26], resulting in the identification of 157 conventionally secreted protein candidates.

### Testing the secretion of the fusion proteins LpAsp-green fluorescent protein and LpCht-green fluorescent protein

Plasmid DNA expressing the LpAsp-green fluorescent protein (GFP) or LpCht-GFP fusion proteins was constructed by amplifying LpAsp (Lp_000442900.1) and LpCht (Lp_160010600.1) DNA by PCR using KOD-FX DNA polymerase (Toyobo Co., Osaka, Japan), *L. passim* genomic DNA and the following primer pairs: LpAsp-5 and LpAsp-3, and LpCht-5 and LpCht-3. The PCR products were digested with XbaI, gel purified and then cloned in the* Xba*I site of pTrex-n-eGFP [27]. Actively growing *L. passim* (4 × 10^7^) were washed twice with 5 ml phosphate-buffered saline (PBS) each time, and then resuspended in 0.4 ml of Cytomix buffer without EDTA (20 mM KCl, 0.15 mM CaCl_2_, 10 mM K_2_HPO_4_, 25 mM HEPES and 5 mM MgCl_2_, pH 7.6) [28, 29]. The parasites were electroporated twice (with a 1-min interval between electroporations) with 10 μg of plasmid DNA expressing LpAsp-GFP or LpCht-GFP, as well as with pTrex-n-eGFP using a Gene Pulser X cell electroporator (Bio-Rad Laboratories, Hercules, CA, USA) and cuvette (2-mm gap). The voltage, capacitance and resistance was set at 1.5 kV, 25 μF and infinity, respectively. The electroporated parasites were cultured in 4 ml of modified FP-FB medium [30], followed by the addition of G418 (200 μg/ml; Sigma-Aldrich) after 24 h of culture to select the G418-resistant clones.

To visualize GFP, we first washed live *L. passim* expressing GFP, LpAsp-GFP or LpCht-GFP 3 times with PBS and then mounted the samples on poly-L-lysine-coated glass slides. We started culturing the *L. passim* for the following experiment at a concentration of 10^4^/ml in 4 ml of culture medium containing G418, and then collected the parasites as well as the conditioned medium after 4 days. The GFP and the fusion proteins in 3 ml of conditioned medium were immunoprecipitated by adding GFP-Trap Agarose (ChromoTek, Planegg, Germany) for 5 h at 4 °C, and then the beads were washed 3 times with 1 ml of washing buffer (10 mM Tris–HCl, pH7.5, 150 mM NaCl, 0.05% NP-40, 0.5 mM EDTA). As a final step, the beads and the parasites collected as described in the preceding paragraph (4 × 10^7^) were suspended in 40 and 200 μl of sodium dodecyl sulfate-polyacrylamide gel electrophoresis (SDS-PAGE) sample buffer (2% SDS, 10% glycerol, 10% β-mercaptoethanol, 0.25% bromophenol blue, 50 mM Tris–HCl, pH 6.8), respectively. After heating the samples at 95 °C for 5 min and centrifugation, 15-μl aliquots of supernatants were subjected to 12% SDS-PAGE, following which the protein products were transferred to a nitrocellulose membrane (Pall® Life Sciences, Pall Corp., Port Washington, NY, USA). The membrane was then blocked with PBST (PBS with 0.1% Tween-20) containing 5% bovine serum albumin at room temperature for 30 min, followed by incubation with 1000-fold diluted anti-GFP antibody (Proteintech, Sankt Leon-Rot, Germany) at 4 °C overnight. The membrane was washed 5 times with PBST (5 min each) and then incubated with 10,000-fold diluted IRDye® 680RD donkey anti-rabbit IgG (H + L) (LI-COR Biosciences, Lincoln, NB, USA) in PBST containing 5% skim milk at room temperature for 2 h. The membrane was then washed as stated above and visualized on the Odyssey Imaging System (LI-COR Biosciences). Proteins in the remaining 1 ml of conditioned medium above were precipitated by adding 4 ml cold (− 20 °C) acetone followed by a 1-h incubation at − 20 °C and centrifugation for 30 min at 10,000 *g*. The protein pellet was air-dried for 30 min, dissolved in 35 μl of SDS-PAGE sample buffer and then applied to a 12% SDS-PAGE gel together with 15 ul of the parasite lysates prepared as described above. The proteins were stained by InstantBlue Coomassie protein stain.

### Deletion of *LpAsp* and *LpCht* genes by CRISPR

To delete the *LpAsp* and *LpCht* genes, we first designed the guide RNA (gRNA) sequences using a custom gRNA design tool (http://grna.ctegd.uga.edu) [31]. Two complementary oligonucleotides (0.1 nmol each) corresponding to these single guide RNA (sgRNA) sequences (LpAsp_For and LpAsp_Rev, and LpCht_For and LpCht_Rev) were phosphorylated by T4 polynucleotide kinase (TAKARA, Kyoto, Japan) followed by annealing and cloning into BbsI-digested pSPneogRNAH vector [32]. We electroporated *L. passim* expressing Cas9 [33] with 10 μg of plasmid DNA constructed as described above and selected the transformants by blasticidin (50 μg/ml; Shanghai Macklin Biochemical Co., Shanghai, China) and G418 to establish the parasite expressing both Cas9 protein and *LpAsp* gRNA or *LpCht* gRNA.

We constructed the donor DNA for the *LpAsp* gene by fusion PCR of three DNA fragments: the 5’-untranslated region (5’UTR; 515 bp, LpAsp5’UTR-F and LpAsp5’UTR-R), the open reading frame (ORF) of the Hygromycin B phosphotransferase gene (*Hph*) derived from pCsV1300 [34] (1026 bp, LpAspHph-F and LpAspHph-R) and the 3’ half of the *LpAsp* ORF (460 bp, LpAsp3’ORF-F and LpAsp3’ORF-R). The donor DNA for *LpCht* was prepared similarly, with the 5’UTR (574 bp, LpCht5’UTR-F and LpCht5’UTR-R), *Hph* ORF (1026 bp, LpChtHph-F and LpChtHph-R) and the 3’UTR (589 bp, LpCht3’ORF-F and LpCht3’ORF-R). The fusion PCR products were cloned into the* Eco*RV site of pBluescript II SK(+), and the linearized plasmid DNA (10 μg) by HindIII was used for electroporation of *L. passim* expressing both Cas9 and *LpAsp* gRNA or *LpCht* gRNA, as described above.

After electroporation, *L. passim* resistant to blasticidin, G418 and hygromycin (150 μg/ml; Sigma-Aldrich) were selected, and a single parasite was cloned by serial dilutions in a 96-well plate. We initially determined the genotype of each clone through the detection of 5’ wild-type (WT) and knock-out (KO) alleles for *LpAsp* and* LpCht* by PCR. After identifying the heterozygous (+/–) and homozygous (-/-) KO clones, the 5’WT (LpAsp5’UTR-Outer-F and LpAsp-72R as well as LpCht5’UTR-Outer-F and LpCht-108R), 5’KO (LpAsp5’UTR-Outer-F and Hyg-159R as well as LpCht5’UTR-Outer-F and Hyg-159R), 3’WT (LpAsp-555F and LpAsp3’UTR-Outer-R as well as LpCht-756F and LpCht3’UTR-Outer-R) and 3’KO (Hyg-846F and LpAsp3’UTR-Outer-R as well as Hyg-846F and LpCht3’UTR-Outer-R) alleles were confirmed by PCR using the specific primer sets. We maintained *LpAsp*- and *LpCht*- deleted parasites in the culture medium with only hygromycin since the plasmid DNAs to express gRNA and Cas9 were no longer necessary [33].

### Detection of *LpAsp* and *LpCht* mRNAs by RT-PCR

Total RNA was extracted from WT, *LpAsp* and *LpCht* heterozygous and homozygous mutant parasites using TRIzol reagent (Sigma-Aldrich) and treated with 1 U of RNase-free DNase (Promega, Madison, WI, USA) at 37 °C for 30 min. Total RNA (0.2 μg) was reverse transcribed using ReverTra Ace (TOYOBO Co.) and random primer followed by PCR with KOD-FX DNA polymerase (note that in Fig. 3c, a primer corresponding to *L. passim* splice leader sequence (LpSL-F) was used as the forward primer). As the reverse primers, LpAsp-260R and LpGAPDH-R were used (note that in Fig. 3d, LpCht-756F and LpCht-1248R as well as LpGAPDH-F and LpGAPDH-R were used).

### Characterization of *LpAsp and LpCht* mutant parasites

We inoculated WT and *LpAsp*- (clones A9 and B6) and *LpCht*- (clones D11 and E6) homozygous mutant parasites into the culture medium at 10^4^/ml in 12-well plates (3 wells for each parasite) and then counted the number of parasites for 4 days using a hemocytometer after suspending them well. The images of cultured parasites were captured after 5 days (at the late stationary phase). The number of rosettes present in the images captured from three wells was counted for individual parasites. Statistical analysis on the number of rosettes was performed using the Dunnet test (one-tailed).

To infect honey bees with *L. passim*, we collected the parasites during the logarithmic growth phase (5 × 10^5^/ml) and washed them once with PBS followed by suspension in sterile 10% sucrose/PBS at a concentration of 5 × 10^4^ /μl. Newly emerged honey bee workers were collected by placing the frames with late pupae in an incubator maintained at 33 °C and then starving the honey bees for 2–3 h. Twenty individual honey bees were then fed with 2 μl of the 10% sucrose/PBS solution containing either WT, *LpAsp*- (clones A9 and B6) or *LpCht*- (clones D11 and E6) homozygous mutant parasites (10^5^ parasites in total). The infected honey bees were maintained in metal cages at 33 °C for 14 days and then frozen at − 80 °C. These experiments were repeated 3 times. We sampled eight honey bees from each of the above three experiments, and thus analyzed 24 honey bees in total that were infected with either WT, *LpAsp*- (clones A9 and B6), or *LpCht*- (clones D11 and E6) homozygous mutant parasites. Genomic DNA was extracted from the whole abdomen of individual bees using DNAzol® reagent (Thermo Fisher Scientific). We quantified *L. passim* in the infected honey bee by quantitative PCR (qPCR) using LpITS2-F and LpITS2-R primers, which correspond to the part of internal transcript spacer region 2 (ITS2) in the ribosomal RNA gene (*rRNA*). Honey bee *Apis mellifera* heat sensitive transient receptor potential channel A (*AmHsTRPA*) was used as the internal reference using AmHsTRPA-F and AmHsTRPA-R primers [16]. The relative abundances of *L. passim* in the individual honey bees (24 each infected by WT or mutant *L. passim*) were calculated by the ΔC_t_ method, with one sample infected by the WT set as 1. The Kruskal–Wallis test followed by the Steel test was conducted for the statistical analysis. All of the above primers are listed in Additional file 2: Table S1.

## Results and Discussion

### Identification and characterization of the *L. passim* exoproteome

**Fig. 1:**
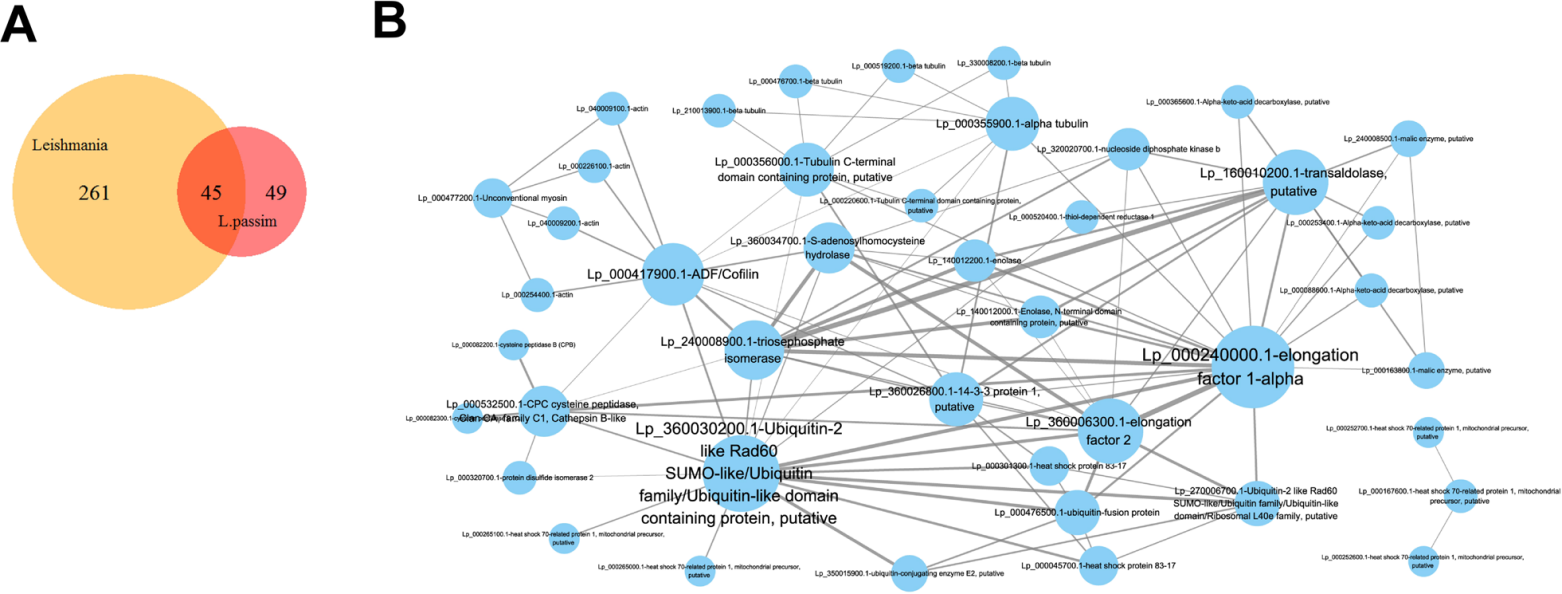
Bioinformatic characterization of the *Lotmaria passim* exoproteome. **a** Venn diagram showing that 45 proteins are shared between the *L. passim* and seven *Leishmania* spp. exoproteomes [6]. **b** Protein–protein interaction map of *L. passim* exoproteome predicted by the STRING database. The size of the circle and the thickness of line are proportional to the number and confidence of interactions, respectively

We used mass spectrometry analysis to identify the exoproteome of *L. passim* promastigotes in 30 ml of conditioned culture medium. The list of proteins identified (94 in total) can be found in Additional file 3: Dataset S2. To ensure robustness, we performed triplicate analyses, yielding 136, 142 and 223 identified proteins, respectively. Notably, 94 proteins were consistently detected in all three replicates. Our KEGG pathway enrichment analysis revealed that the “Exosome” pathway was highly enriched within the exoproteome, suggesting that we successfully recovered proteins associated with EVs (Table 1). We also observed enrichment in pathways related to bacterial infection, such as the “*Salmonella* infection,” “Pathogenic* Escherichia coli* infection,” “Legionellosis” and the “NOD-like receptor signaling pathways.” These findings suggest that *L. passim* may employ exoproteome proteins associated with these pathways to interact with the microbiota in the honey bee hindgut. Our GO term enrichment analysis results aligned with those from the KEGG pathway analysis. Notably, in the “Cellular Compartment” category, we observed enrichments in terms such as “extracellular region,” “external encapsulating structure” and “biofilm matrix,” indicating the presence of proteins in the exoproteome related to these compartments. Additionally, the enrichment of the “cell periphery” GO term suggests that a substantial portion of the *L. passim* exoproteome comprises cell surface proteins shed into the culture medium. In the “Biological Process” category, we identified enrichments in terms like “evasion of host immune response,” “response to endogenous stimulus” and "modulation by symbiont of host cellular process." These findings suggest that the exoproteome proteins may play roles in interacting with the host and adapting to the hindgut environment. However, it is important to consider that KEGG pathway and GO enrichment analyses assume that many proteins associated with the above pathways and GO terms (“Biological Process”) are intracellular; consequently, their biological functions may not be the same once they are secreted. We also compared the *L. passim* exoproteome (94 proteins) with 306 exoproteome proteins shared among seven *Leishmania* spp. [6]. Our analysis revealed 45 common proteins, indicating a core set of exoproteome proteins with conserved functions in the Leishmaniinae lineage (Fig. 1a; Additional file 4: Dataset S3). These include, for example, GP63, tryparedoxin peroxidase, β-fructofuranosidase, enolase, calpain-like cysteine peptidase Clan CA family C2, cyclophilin 4, heat shock protein 83-17, S-adenosylhomocysteine hydrolase, tubulin, ubiquitin-conjugating enzyme E2 and elongation factor 1-α (Table 2). We also found that 43 of the 94 *L. passim* exoproteome proteins have significant interactions, as shown in Fig. 1b.

**Table 1:** Representative Kyoto Encyclopedia of Genes and Genomes pathways enriched with the *Lotmaria passim* exoproteome

ID	Description	*q*-Value
ko04147	Exosome [BR:ko04147]	1.11E−11
ko04540	Gap junction	3.05E−11
ko05132	Salmonella infection	1.44E−08
ko05130	Pathogenic Escherichia coli infection	5.40E−08
ko04145	Phagosome	1.36E−07
ko04612	Antigen processing and presentation	2.81E−05
ko04812	Cytoskeleton proteins [BR:ko04812]	0.000322137
ko05417	Lipid and atherosclerosis	0.001359886
ko05134	Legionellosis	0.001488872
ko03051	Proteasome [BR:ko03051]	0.001488872
ko00500	Starch and sucrose metabolism	0.001913062
ko04621	NOD-like receptor signaling pathway	0.003757488
ko04210	Apoptosis	0.007150768
ko01009	Protein phosphatases and associated proteins [BR:ko01009]	0.007208296
ko04141	Protein processing in endoplasmic reticulum	0.007682533
ko05145	Toxoplasmosis	0.007927139
ko03110	Chaperones and folding catalysts [BR:ko03110]	0.007927139
ko03036	Chromosome and associated proteins [BR:ko03036]	0.007927139
ko00052	Galactose metabolism	0.008324948
ko03019	Messenger RNA biogenesis [BR:ko03019]	0.009719993
ko00680	Methane metabolism	0.010088314
ko04626	Plant-pathogen interaction	0.011122684
ko04217	Necroptosis	0.011850098
ko04530	Tight junction	0.015760733
ko00010	Glycolysis/Gluconeogenesis	0.015915111
ko04010	MAPK signaling pathway	0.016442493

**Table 2:** Representative Gene Ontology terms enriched with the *L. passim* exoproteome

GO categories	ID	Description	*q*-Value
BP	GO:0051017	Actin filament bundle assembly	7.37E−06
BP	GO:0006090	Pyruvate metabolic process	4.97E−05
BP	GO:0005996	Monosaccharide metabolic process	2.46E−04
BP	GO:0009132	Nucleoside diphosphate metabolic process	3.57E−04
BP	GO:0009651	Response to salt stress	3.59E−03
BP	GO:0000951	Methionine catabolic process to 3-methylthiopropanol	3.59E−03
BP	GO:0030656	Regulation of vitamin metabolic process	3.59E−03
BP	GO:0006568	Tryptophan metabolic process	5.23E−03
BP	GO:0042744	Hydrogen peroxide catabolic process	9.05E−03
BP	GO:0044273	Sulfur compound catabolic process	1.03E−02
BP	GO:0051085	Chaperone cofactor-dependent protein refolding	1.51E−02
BP	GO:0042783	Evasion of host immune response	1.51E−02
BP	GO:0006970	Response to osmotic stress	0.0207384
BP	GO:0009719	Response to endogenous stimulus	0.032515695
BP	GO:0030682	Mitigation of host defenses by symbiont	0.033452
CC	GO:0005576	Extracellular region	1.52E−08
CC	GO:0030312	External encapsulating structure	1.01E−06
CC	GO:0005618	Cell wall	4.35E−06
CC	GO:0062039	Biofilm matrix	0.000393
CC	GO:0045273	Respiratory chain complex II	0.000602
CC	GO:0071944	Cell periphery	0.003817
CC	GO:0005773	Vacuole	0.005135
MF	GO:0045290	D-arabinose 1-dehydrogenase [NAD(P)+] activity	0.000184
MF	GO:0016989	Sigma factor antagonist activity	0.00074
MF	GO:0004033	Aldo–keto reductase (NADP) activity	0.001085
MF	GO:0004737	Pyruvate decarboxylase activity	0.001832
MF	GO:0004470	Malic enzyme activity	0.004209
MF	GO:0008379	Thioredoxin peroxidase activity	0.010454
MF	GO:0044183	Protein folding chaperone	0.017866

*BP* Biological Process,* CC* Cellular Compartment,* MF* Molecular Function

### Identification of conventionally secreted proteins in *L. passim*

We conducted a bioinformatic analysis to identify 157 proteins in *L. passim* that are potentially secreted by a conventional pathway. These proteins were characterized by the presence of N-terminal SPs and the absence of transmembrane domains (Additional file 5: Dataset S4). Intriguingly, only 14 of these proteins overlapped with the *L. passim* exoproteome, suggesting that the majority of exoproteome proteins are secreted through non-canonical pathways. However, it is plausible to hypothesize that some conventionally secreted proteins were present at levels below the detection limit of our mass spectrometry analysis. KEGG pathway and GO term enrichment analyses of these 157 *L. passim* proteins revealed that they include not only secreted proteins but also ER, Golgi and lysosome luminal proteins (Tables 3, 4). These proteins are characterized by the presence of N-terminal SPs and amino acid sequences that enable them to be retained in either the ER, Golgi, or lysosome, as previously described [35]. LpCht is associated with three GO terms, namely “carbohydrate metabolic process,” “chitin binding” and “hydrolyzing* O*-glycosyl compounds.” It is also involved in the KEGG pathway, “amino sugar and nucleotide sugar metabolism”. Two GO terms, namely “proteolysis” and “aspartic-type endopeptidase activity‚ are assigned for LpAsp, but there is no specifically associated KEGG pathway. Both LpAsp and LpCht were not detected in the above *L. passim* exoproteome, likely due to the low abundance of endogenous proteins.

**Table 3:** Kyoto Encyclopedia of Genes and Genomes pathways enriched with 157 potential proteins secreted by the conventional pathway in *L. passim*

ID	Description	*q*-Value
ko03110	Chaperones and folding catalysts [BR:ko03110]	1.16E−06
ko04141	Protein processing in endoplasmic reticulum	8.40E−04
ko04091	Lectins [BR:ko04091]	3.12E−03
ko04142	Lysosome	1.73E−02
ko00785	Lipoic acid metabolism	8.99E−02
ko01003	Glycosyltransferases [BR:ko01003]	8.99E−02
ko04512	ECM-receptor interaction	8.99E−02
ko01002	Peptidases and inhibitors [BR:ko01002]	8.99E−02

**Table 4:** Representative Gene Ontology terms enriched with 157 potential proteins secreted by the conventional pathway in *L. passim*

ONTOLOGY	ID	Description	*q*-value
BP	GO:0034975	Protein folding in endoplasmic reticulum	1.57E−14
BP	GO:0006457	Protein folding	1.26E−08
BP	GO:0036500	ATF6-mediated unfolded protein response	1.5E−07
BP	GO:0061635	Regulation of protein complex stability	6.67E−07
BP	GO:0006986	Response to unfolded protein	8.52E−07
BP	GO:0031204	Post-translational protein targeting to membrane, translocation	1.11E−06
BP	GO:0071287	Cellular response to manganese ion	1.11E−06
BP	GO:0034976	Response to endoplasmic reticulum stress	1.11E−06
CC	GO:0005788	Endoplasmic reticulum lumen	7.01E−20
CC	GO:0034663	Endoplasmic reticulum chaperone complex	2.63E−11
CC	GO:0005790	Smooth endoplasmic reticulum	1.51E−08
CC	GO:0005783	Endoplasmic reticulum	2.13E−08
MF	GO:0051082	Unfolded protein binding	4.16E−07
MF	GO:0031685	Adenosine receptor binding	5.74E−07

*BP* Biological Process,* CC* Cellular Compartment,* MF* Molecular Function

### Confirmation of LpAsp and LpCht secretion

**Fig. 2:**
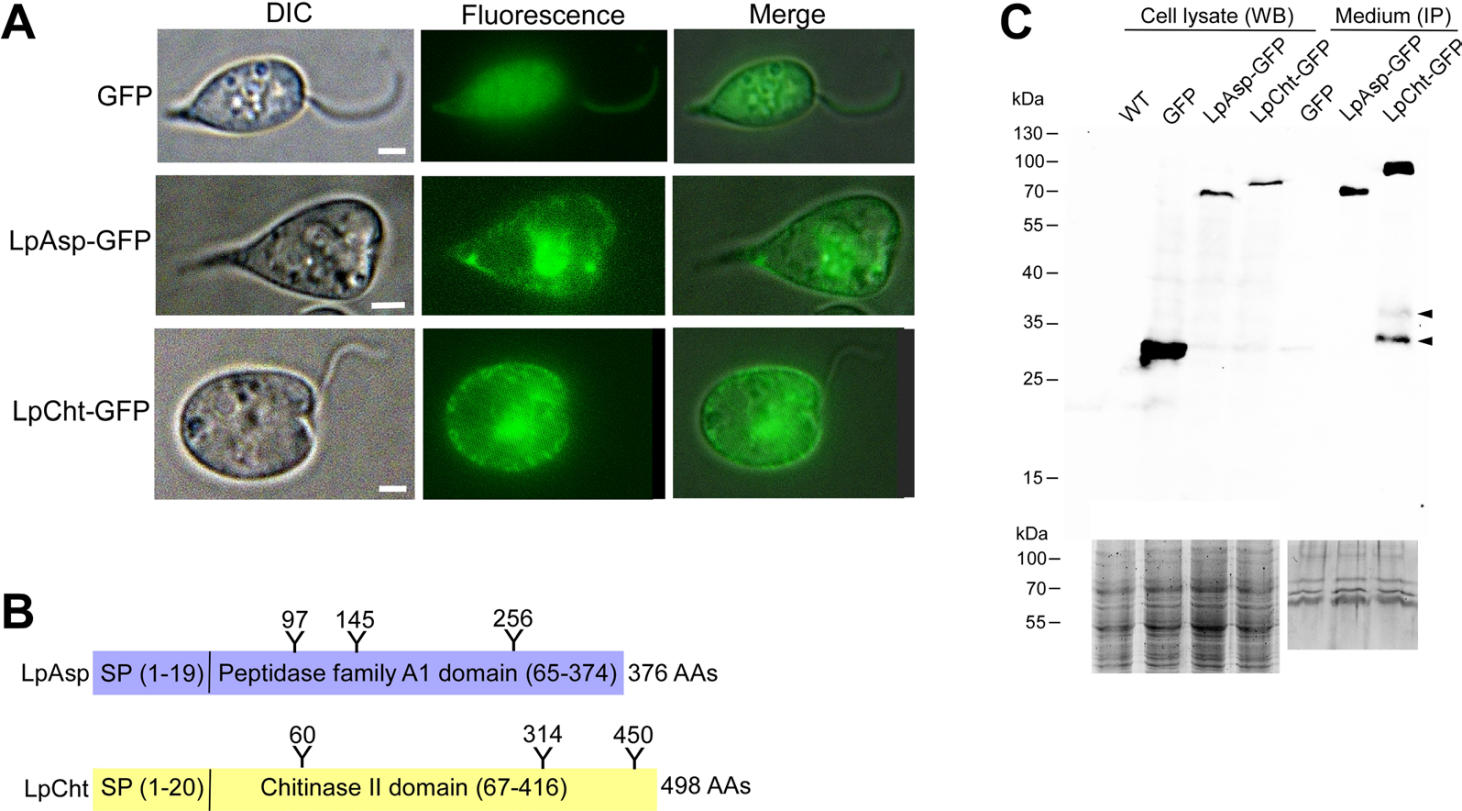
Secretion of LpAsp and LpCht proteins. **a** Visualization of *L. passim* expressing GFP, LpAsp-GFP or LpCht-GFP fusion proteins using both visible differential interference contrast (DIC) and fluorescence (Fluorescence) microscopy. Merged images are also presented (Merge). Note that fluorescence signals for LpAsp-GFP and LpCht-GFP were enhanced through longer exposure times compared to GFP alone. Scale bar: 1 μm. **b** Schematic representation of LpAsp and LpCht proteins. The positions of amino acids encoding SP and molecular functional domain are shown together with those of potential N-glycosylation sites (Y). The total number of AAs in each protein is given at the right. The size of protein is not in scale.** c** Quantification of intracellular GFP, LpAsp-GFP, and LpCht-GFP via WB analysis of cell lysates, with WT *L. passim* used as a control. Extracellular protein levels were determined through IP of equal volumes of conditioned medium, followed by WB. Arrowheads indicate two cleaved LpCht-GFP proteins. SDS-PAGE gels stained with InstantBlue displaying the cell lysates and concentrated conditioned medium are presented at the bottom. Equal protein amounts were loaded for the WB or IP analysis. Molecular weights (kDa) of the protein markers are indicated on the left. AA, Amino acid; GFP, green fluorescent protein; IP, immunoprecipitation; LpAsp-GFP, *L. passim* aspartyl protease–GFP fusion protein; *L. passim* LpCht-GFP, chitinase-GFP fusion protein; SDS-PAGE, sodium dodecyl sulfate-polyacrylamide gel electrophoresis; SP, signal peptide; WB, western blot; WT, wild type

To verify the secretion of LpAsp and LpCht, we introduced plasmid DNA into *L. passim* for the expression of GFP-tagged proteins. While GFP was uniformly distributed in the cytoplasm, the fluorescence associated with LpAsp-GFP and LpCht-GFP fusion proteins was weak and patchy (Fig. 2a). Consequently, LpAsp-GFP and LpCht-GFP were detected in lower quantities compared to GFP within the parasites. However, a higher abundance of fusion proteins was observed in the culture medium (Fig. 2c), indicating the secretion of LpAsp and LpCht. Notably, the sizes of LpAsp-GFP and LpCht-GFP in the medium appeared larger than those in the cell lysates, suggesting the presence of matured proteins with full glycosylation and other post-translational modifications (Fig. 2b). Because two small LpCht-GFP proteins were present in the culture medium (arrowheads in Fig. 2c), LpCht would appear to be cleaved at two sites in the C-terminus after secretion. However, we were unable to determine the physiological role of this cleavage.

### Deletion of *LpAsp* and *LpCht* genes by clustered regularly interspaced short palindromic repeats technology

**Fig. 3:**
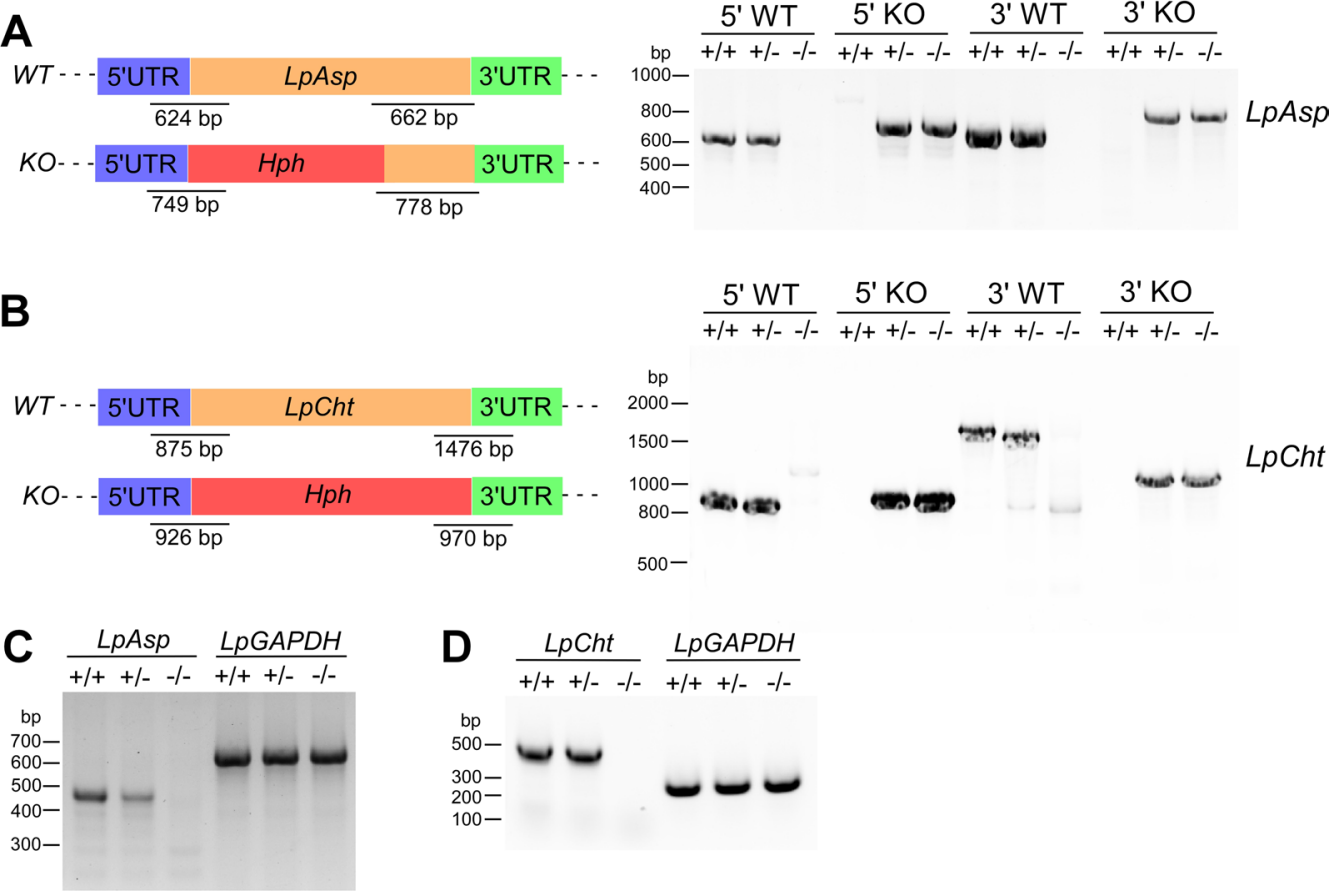
Deletion of the *LpAsp* and *LpCht* genes by CRISPR. Schematic representation of* WT* and deleted* KO* alleles of the *LpAsp* (**a**) and *LpCht* (**b**) genes generated using CRISPR/Cas9-induced homology-directed repair. 5’and 3’-UTRs, open reading frames and *Hph* gene are depicted in blue, green, beige, and red, respectively. The expected sizes of the PCR products for detecting the WT and KO alleles (not to scale) are also displayed for each gene. Genomic DNAs from WT *L. passim* (+/+), *L. passim* heterozygous (+/–) and *L. passim* homozygous (-/-) mutants of *LpAsp* and *LpCht* were analyzed by PCR to detect 5’*WT*, 5’*KO*, 3’*WT* and 3’*KO* alleles. Sizes of the DNA molecular weight markers are provided on the left.** c** Detection of *LpAsp* and *LpGAPDH* mRNAs in *LpAsp* heterozygous ( +/–) and homozygous (-/-) mutants, along with WT *L. passim* (+/+) through reverse transcription-PCR. A forward primer corresponding to the *L. passim* splice leader sequence was utilized. Sizes of DNA molecular weight markers are shown on the left.** d** Detection of *LpCht* and *LpGAPDH* mRNAs in *LpCht* heterozygous (+/–) and homozygous (-/-) mutants and in WT *L. passim* (+/+), by reverse transcription-PCR. Sizes of the DNA molecular weight markers are indicated on the left. CRISPR, Clustered regularly interspaced short palindromic repeats; *hph*, hygromycin resistance gene; KO, deleted (knockout); LpAsp, *L. passim* aspartyl protease; LpCht, *L. passim* chitinase; LpGAPDH, *L. passim* glyceraldehyde-3-phosphate dehydrogenase; mRNA, messenger RNA; WT, wild type; UTR, untranslated region

**Fig. 4:**
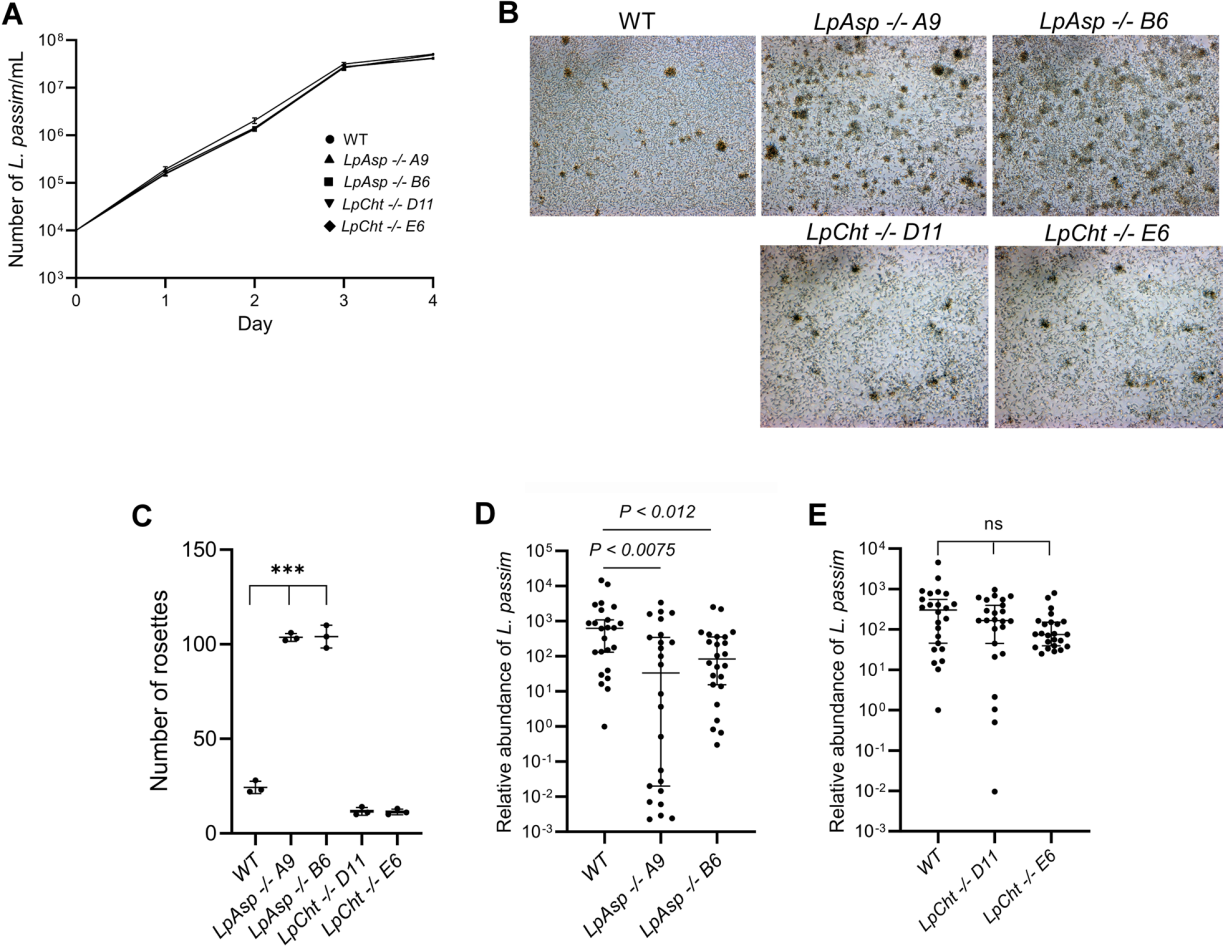
Growth and rosette formation of *LpAsp* and *LpCht* mutants, as well as their infection in honey bees. **a** Growth rates of WT (circle) and homozygous (-/-) mutant strains for *LpAsp* (triangle denotes clone A9; square denotes clone B6), and *LpCht* (reversed triangle denotes clone D11; diamond denotes clone E6) in modified FP-FB medium were monitored at 28 °C over 4 days (*n* = 3). Symbols represent mean values ± standard deviation (SD) (error bars). **b** Microscopic images of parasites in the medium captured 5 days after culture initiation, showing rosettes of various sizes. **c** Count of rosettes within three different areas with the individual parasites (*n* = 3). Symbols represent mean values ± SD (error bars); statistical analysis was performed using the Dunnet test (one-tailed). Asterisks indicate statistical significance at *** *P* < 1.9E−06.** d**,** e** The relative abundance of *L. passim* in individual honey bees (*n* = 24) at 14 days post-infection was compared between WT and homozygous mutants (-/-) of *LpAsp* (**d**; clones A9 and B6) or *LpCht* (**e**; clones D11 and E6). One sample infected by the WT parasite was set at the reference value of 1, and symbols represent the median with 95% confidence interval. Statistical analysis was performed using the Kruskal–Wallis test followed by the Steel test. LpAsp, *L. passim* aspartyl protease; LpCht, *L. passim* chitinase; ns, not significant; WT, wild type

To investigate the functions of LpCht and LpAsp, we utilized clustered regularly interspaced short palindromic repeats (CRISPR) technology to delete the ORFs of these genes, replacing them with the *Hph* gene. Our approach, previously reported, confirmed that expressing Cas9 and gRNA does not result in modifications to the target gene in *L. passim*, thereby minimizing off-target effects [33]. Genomic PCR demonstrated the successful disruption of both the *LpAsp* and *LpCht* genes, with the homozygous mutant parasites lacking the WT alleles at both the 5' and 3' ends of the gene (Fig. 3a, b). We also confirmed the absence of *LpAsp* and *LpCht* mRNAs in the homozygous mutant parasites (Fig. 3c, d). These results indicate that neither *LpAsp* nor *LpCht* are essential for *L. passim* viability under culture conditions, as expected. Growth rate comparisons revealed no significant differences between *LpAsp* or *LpCht* mutant parasites and the WT (Fig. 4a). However, we did note an increase in the formation of rosettes (clusters of cells with their flagella toward the center) with *LpAsp* mutant parasites at the late stationary phase of culture, relative to the *LpCht* mutant and WT parasites (Fig. 4b, c). The phenotypes were comparable between the two clones for each mutant parasite. Rosettes were observed in association with *Leishmania* parasites in the midguts of insect vectors, characterized by the presence of surface poly-α2,8 N-acetyl neuraminic acid (PSA) and PSA containing de-N-acetyl neuraminic acid (NeuPSA). It has been suggested that these rosettes may represent a unique stage of *Leishmania*, possibly related to the initiation of mating [36]. Thus, the loss of *LpAsp* changed the physiological/developmental state of *L. passim* under the culture conditions.

### LpAsp, but Not LpCht, contributes towards efficient infection in the honey bee gut

We conducted experiments to assess whether LpAsp and LpCht are necessary for *L. passim* infection in the honey bee hindgut. Infections were carried out using WT, *LpAsp* and *LpCht* mutants *L. passim*, and we quantified the relative number of parasites in the honey bee gut after a 14-day maintenance period in cages. Our results revealed that the infection levels of WT and *LpCht* mutant parasites were comparable, while the number of *LpAsp* mutant parasites in the honey bee gut was significantly reduced (Fig. 4d, e). There was no significant difference between two clones of each mutant parasite. This finding suggests that LpAsp—but not LpCht—contributes to the efficient infection of *L. passim*.

We also attempted to measure chitinase activity of the cell lysate as well as of the concentrated conditioned culture medium of WT *L. passim* based on the methods described in [20]. For this purpose, we used two substrates, P-nitrophenyl (PNP)-β-D-N,N’-diacetylchitobiose and PNP-N-acetyl-β-D-glucosaminide; however, digestion activity was never detected with either substrate. We speculate that the amount of endogenous chitinase would be very little in *L. passim* promastigotes or that *L. passim* chitinase does not recognize the above compounds as a substrate. *L. passim*, when ingested by honey bees, is likely surrounded by PM in the midgut. However, the release of *L. passim* from the PM using chitinase may not be essential for infection; rather, it appears that this release might be specifically required for parasites, such as *Leishmania*, to infect insect midgut. This parallels the case of the honey bee pathogen, *Paenibacillus larvae*, which causes American Foulbrood and requires chitinase for larval infection [37]. *L. passim* may migrate to the hindgut following the direction of gut flow in honey bees. The attachment of *L. passim* to the hindgut epithelial cells could depend on chitinase to disrupt the cuticle layer, although recent research indicates that the cuticle layer in *L. passim*-infected hindguts remains intact [38]. Although chitinase has been biochemically characterized in *Leishmania *[39], its loss-of-function effect on infection in the sand fly midgut has not been tested. Thus, despite the conservation of chitinase among trypanosomatid parasites, its exact role in insect hosts or vectors remains not fully understood.

The results of our study indicate that LpAsp is important for *L. passim* to efficiently infect the honey bee gut. However, we were not able to establish an enzyme assay for LpAsp since the protein substrate remains unknown. Identifying the potential substrate for digestion by LpAsp is an interesting question. Honey bee antimicrobial peptides (AMPs) could be potential substrates for LpAsp. Our previous research showed that *AMP* mRNA expression, such as the apidaecins type 14 precursor, is upregulated by *L. passim* infection [16], suggesting that the parasite may utilize LpAsp to inactivate AMPs. Additionally, LpAsp may modify target proteins on the surface of *L. passim* or the host cell for better adhesion of the parasite. Notably, secreted proteases of *P. larvae* and *Candida albicans* have also been suggested as virulence factors [40–42]. The relationship between the inefficient infection of LpAsp mutants in the honey bee hindgut and the enhanced formation of rosettes under culture conditions remains to be explored.

In the present study, we identified the exoproteome of *L. passim* promastigotes and noted that 48% of the proteins seem to represent the core exoproteome common among Leishmaniinae species, which suggests that some exoproteome proteins have conserved functions for trypanosomatid parasites to infect the gut (midgut or hindgut) of various insect species, regardless of their monoxenous or dixenous life-cycles. Other exoproteome proteins may have functions specific to infecting a particular insect and/or mammalian host species. We also demonstrated that at least two *L. passim* hydrolytic enzymes with N-terminal SPs are secreted, one of which is involved in *L. passim* development under culture conditions and contributes towards efficient infection in honey bees. Given that the composition of the exoproteome is likely to vary under different physiological conditions, characterizing the *L. passim* exoproteome in honey bee hindguts holds significant interest. Similar investigations have been conducted for other honey bee pathogens and parasites, such as *P. larvae*, with the aim to identify potential virulence factors [43, 44]. Consequently, the exoproteome represents a valuable resource for gaining a better understanding of the mechanisms through which trypanosomatid parasites and other parasites/pathogens infect insect (and mammalian) hosts and induce pathogenesis.

## Conclusions

Our study successfully identified the exoproteome of *L. passim*, a trypanosomatid parasite in honey bees. The results of our bioinformatic analysis strongly suggests that the *L. passim* exoproteome plays a crucial role in interactions with both the host and its associated microbiota. Furthermore, our functional analysis of genes encoding two secreted proteins revealed that the role of aspartyl protease in facilitating efficient infection in the honey bee gut. Overall, our results emphasize the significance of the exoproteome as a valuable resource for unraveling the mechanisms employed by trypanosomatid parasites to infect insect hosts through interactions within the gut environment.

### Supplementary Information

**Additional file 1. ** List of 9339 *L. passim* annotated proteins.

**Additional file 2. **List of primers used in this study.

**Additional file 3.** List of 94 *L. passim* exoproteome proteins.

**Additional file 4.** List of 45 *L. passim* exoproteome proteins shared with seven *Leishmania* spp.

**Additional file 5.** List of potential 157 *L. passim* proteins secreted by conventional pathway.

## Data Availability

All data are included as tables, figures and Additional files in the article. The mass spectrometry proteomics data were deposited to the ProteomeXchange Consortium via the PRIDE [45] partner repository with the dataset identifier PXD040336.
