# Supplementary material for: Aspartyl protease in the secretome of honey bee trypanosomatid parasite contributes to infection of bees
Source: Parasit Vectors. 2024 Feb 10;17:60. doi: 10.1186/s13071-024-06126-7 (PMC10859015; doi:10.1186/s13071-024-06126-7)
Supplement: Supplementary file 1 — Additional file 1. List of 9339 L. passim annotated proteins. [file 13071_2024_6126_MOESM1_ESM.docx]

>Lp_000005000.1 hypothetical protein, conserved

MPDASQLHATKLLAFPSQGTAAEFAWVSSTAVKSNGGGVGEEGIASLPAVTASAQDGVVR

LWDLQYDSASATGAVAGMATRATAATANG

>Lp_000005100.1 Cupin-like domain containing protein, putative

MSVHTPSSAAKTAASAAAPPPTLLEAFATSAAQLRHFGIPRIRIVGLSEGCTQADYEEQR

CRPGGKDMETLLQESTRRGPHGALGTASQRVQSEAEARAAEQMNEDVPIMTEQEFMHKFV

FHSRPCVILDAIASWPAMEKWRDDRYVFDLDHKLPLEPERAAHDEEEECEEESDNEDNEG

NEDAAALNKAKAADAKDSSAADAPAPVLGPKKVTVALTPNGRADAVTRVTYSTAAVPDED

AEAVYAGAEAQKYAVMLPSSDLPDDADDVQMRCEKVFMYAAEIRMTLPQLYGLLQRSPLF

PPDRAIEVDMRSYADAAQTPAIAYAQLQNNCLNTEYTHLHVDLGPNVERFGCRVFDKEAV

EAANVWFGIPASVSSMHQDWVENLYSVVRGVKEFVLLPPWEGPFIPKPEIPAAAFAIDEA

NSRIDHDDDTAKSWVLQFKQYPVKDGTTVPWMDFDITGADVEEDAEGVAHAELQRRLFEK

RCVTSALWAAPMSADDAKAAAADEAARKPLHPLVAYVHPGETLYLPAMWLHRVAQHADSI

DMRARAMHHAAAAAGDASAEPPLPLIAAVNYWYDMSFNNPSVVMLREFEKKKKKKKKKKK

KKKKKKKKKKLVTCCCCFHCSRKSALLRQSALAFFFFFVRHLGKKRK*

>Lp_000005200.1 hypothetical protein, conserved

MSDSDWEVLSTDGHAPATSPRTISATANASFDGSSSPNNNNGNDGDANESPHAYASHAID

RRTVLKPLCLTLNQTGTRLGVGHTKGYLVFRVSTSKADAPSAEVHAAATQPPFSSTETHL

RPQSQLILDAQYNVDLLTFSHVRERMQQQRLQRASQQAAAAAATTKEGVASNVLKASTGL

AHADGITSAAPHREDVHVPTRSHSDAGDERLCETPSDQKHDAESGSLGSPLLDLDNIGSF

LTAAGRAFAQIKRDDALSDVQAAPSSRREELHDHASHDKRHTVPTPTVKPQEHPEKSDEP

DIACREPETNHSAEQPTQAQLPPSPVLEWGEDEDIDVDVSDSSTHDVQLGSIGDSIFVHR

RTRCTTPAAAAADRTHPLRSADPNREEESEESEESYVGFEGGGVAVMSFLYDQTWLALVG

GGETPMGPPNQIQFVRDGELQHQLLVPHPVVRLFLDARLLFIVTTAELRVYTNPLEHNWV

CLRQRIAMSTTLAARYSAVSLAFVPSHEYTAAAESQPAPSHVQNSSLASPASVNAGALPD

DKQPSREASADVTTTAKPAPSIAATDMCVALPSMPVVVDYARSLLLLPVGDACNGFALYH

YITGPEPVERPATSDAGGSEGDVQRTHSSAPLSSTTTGRTTAYLQRIALQPEAHKNPLHS

LVLYVGWPAATTRLLATTRKDAAHAKPSNNFTTTSSASVSQTGAAAAALSAYDHSSSRLG

GGGTVTLVAASSEYATRITLWMLKHEEEDEEYNHHIQQQQQQQKGKLGTSPQDSHAHHHD

PQRSTLARGSSISAATLDVFVLLREFRVGLRPAAPKVVMAALPSVSRFVSRAQPTTATSP

PPRLPGGGSSSASNSNAASQYPSTASLVYSHQKAGESEKRRNNNSSTSSSSNAAGLYALP

HDTASTSTYRGTMAAWAKETASTVAAWATEAASTVAACTSTSAAVHHLQFIGNGAYLLCV

HGNDVISVFSTSAPESPQEAKDVTRDRAAAEQNRYSRLSIMKEYLPRALSNRLDAYTRQA

WSSCTGHLPTSDAAFVPRWLYLKRQDAVAAAAARSQSNVRCLADDTASTTTNASPESAGN

QAKAASPGSAIPHGNPSGRRFFRRLTALVGSRSQPAPLPASTPTSAAVTASTQPASSRSV

VSVQDSPRASLADLPSVTEQALTPHSSSTDFHHPSNASVCVYWGAPLSGVPQCVQIWPAS

LHSSLDGDSSGSRGGRGFFGAVRQPIVLNCATCEGAFVNVFLFEEEGEIATSSVVPYAAE

*

>Lp_000005300.1 DnaJ domain containing protein, putative

MWRTPVYRNVQKRIHQVLTRQRTAIVFSKHTAVVPYLTFQRAYASSSSNSSSAGDYYNQS

KEDEEDKVLEEEEFAALREGEDDEFMPLTHRLAHKADALDAELRRLRYTLCDQPQFLVDT

AAATFLGRPTDMKAVIDLLLAAPKSTGITDAASGAASRNPEKPKTSHAASTTCSSSSNSA

SGGRFTSVLSPKLEAALRSSPYGAWTGTSAPVARLLCQRIFWPRSKTADAAASASSHGTE

LATMDADLDYLRRARALHQLNYYSRLNLTPKRHIWLALYQILWNLLIAAQNACGCVVYGA

LRGVKERGMVAGLCTGALTGAGKGLVFLAYGWVLSPLLHLSRGLCNSLYGPVNAVTGRYM

FDAISGRWMQCTVADTVYFRHALQREKRVLRTIGRAEFRRKRMKVEHKWSARMASMGFSV

ESFAEKMKAGKAKRGGRDAGASAKAEEKLRNPYEVLQVRRTASAQEIKKQYKKLAMVFHP

DVAQSRQGGSLSAEEKAAAQHKFEEIANAYQILSNPEKRKAYDMGGAQGVHLHETKYGKF

MSRTPEEMVQSIFGGEGFRRMLVGELLRSHWALRYEAQVSVSIHELEELQCIRVRQLAME

LAAIADVHARRPTTPYYGGAFSASTAAAAGHAQSGTNAQRRNASALRGGLSSSLPDYLKS

GKPRSRAAVAGLGGRPAAAGAGAGAGKGIEKEMHDSIMEELGGGRGGASTSSAAGAGGGA

SAASSRLGGRKSADAAADLSARSPFVLHPGSNEYNCFSRDFEDRCDKFVRYLAEACFGKQ

LLYEVGEAYVVNAQRFLGIRPFYASKALVTRKIFTGMDRVYDAFKDKTKLNKQQVAHKVV

AEYFNIEYDSVVADIHAALRYALQMVLQDAVESEEVRRKRCYAVWLLGEKMMAVGERWTS

RITKDEDLNAYIQQAANSVATTSKPPAF*

>Lp_000005400.1 prefoldin-like protein

MSSITDKYTFKDDYVSPRGIPRVAFVENVAELVKSSGDSAETLLKRFSEQYSKYKLAEHR

LIRTIANLEAKIPDIKKTLQTLEYLKKSLVEEDGGKGFTTNYGITESVFCQAKVLPQKTV

HVWLGANVMVEYTFDEATELLEKNLKSATDNLATTQEDLSWLQEQQTILEVNTSRLYNYD

VVERRKKSESGDKK*

>Lp_000005500.1 WWE domain/WD domain, G-beta repeat, putative

MGAAGSSNKTPRVGGAPPRSVPAQAGASAGAKNGAASAAAAAAAAPPAPAKRGDRLRVIV

RHLRPGEALSSEVGRVTEGGGRVCDDAAAAERGSRSSGTLFGWFVEDVDNPGSWEPYPKE

SADRLEDAFLHQRDSCTVVMKKHTYSVNLNTMQQLPSAGSATATAAAATANRAREVKRVP

VVQYVDPATGCTSVKEVALYMVDPFADEEEERQEANQGIAQGAGDTMGNDTTSTAGSSSA

TGTAPANDGGAAAASSGLPHLIQTVVPHSLPIYTMETTPSLEQLCPEARAIVEPAGGALV

LSSGKDSQLLEWSLDTSRVITKYELPDIANKNSVLTANYSATGKWMIAGLDDRTARLYAI

GNQNEIHRLEGHTHKVYGAGILAGDLQAATASMDCRVKVWDVATGTCVHTITPHKSHIFV

LRPHPADPNFALTAGEDRNVCMHDFRQQNSVVGIFTGHERTIWDLDWNPVDGTFASCGMD

STMRVYDPRASTMARETLRSHTKAVHSLKYTPHGRGLLSCSKDLFVNLTDTSDWSVRWQA

KAHAVTVFRVRYYAGKEVMLTAASDSSVNVWKWTAVNQL*

>Lp_000005600.1 hypothetical protein, conserved

MAEVWRCDVCKKVKDLKGPHLNGKTSVRSDCWPCGKKRTFVKTGADSTTAAATTYTFSTA

TTAKSTVNTNALPASISVTPSTEAQAAAFQNIFAAATAAAAAAAKTSTSSAITTSPPSFP

FSSISRDGINNSSSSSSSKRTSHVNTSSSGSTRGGADGGGAATAFGTSRTSSSPFITPAV

SAASIKKFKTSYASHMTCKHEDPTPASEAGRNTSTFPPSMTSPYVMESSAGTSPPFTFRP

NANYTPTSSTVGGMWSTATLLPGSMASLGNPSAAGRTVSPFMPSAESKPSTEAADAAASA

YATLKPAVLTAAASDAPSTSDAATLVWACEHCHKVKDLKGEHLRGKTTVRSDCWPCAKKR

TFVLTTLAALAAAGRSMKLGGGSGSRSSSMAQRAAVGGDVVKSPSRAISTAAQSQGPLFT

AAVAASAAPTRFENLFTGLPRLATPPKAAGSNAEENVSPPLAENAGSQASPKPVSWVKPV

IPADANADESFFIHPEATLSSPSPSPSAQLSLTSTVPIAHEHSTTSSHGNNSAGSAAPLA

VSSSASWVAAVAASTRGTESDLATYYAAMARFDQQLLKSFRERLVGGVQLRAVWYERVLY

VIAEDAAAAAAEPDDLVGAFAELGRYAALYLAVEQKLAASRFRHVGRLFCPNEFAFAAAC

CYANNFFPSEAATTSSSSLSTTLEPTPGSAKASRFKVYGPQMDPTKSLSLWGRIVSASPA

AQLCVVSEEQRVVAAQTLYRRVSWLAAPQTMTATAIQLSVEAASAADEHGVDVVLLTGTM

KLEELPQAVPLAQLPQPYAC*

>Lp_000005700.1 hypothetical protein, conserved

MFERKAASASIGLLRTHRLHAVTARAFFSLHGSHCGEEQRGHSSTTSATIVFPVFSNSPS

SSKSEVNRKVESEEAFSAPLTSAASATSERSNACGFTGPQEDGLKDAKNSLEGADGEDNA

IEVVRQLQRLAARRPQHQSPADTRTSLAHDADAGISAVALADLMTDEASAFDDGGEHHVK

VGEGDAVSTDQQQQNAAWEAGLQLAEGVVEKNDELAEWLLFLCLRSQDGATGSPAPLEVC

EGVYQAWRQQRRKSAAASAQHQSSAQNSEALLRSPFEPKGVHHHFAVWLLREFHSTWRVV

QEACRDTTAFKATAAACVKDAGDAGFSGAPAEVSTEAEYNQLEVMHHACLLLNRTLEVLL

LDEPLDVVVDVAAACAPAPSRQPHRRHRLGCKAAQLRPSSALLVLEASRQLTQLLAQQQR

LGAPDCTAAFPFLSFLTAKSGPAKATTTPLMEVAREAFELILRSTSPPMLQSDAVVQYLG

FLISASPSSVVPAANVDHFVRCGSLTARRVSRHTNSLKAEELEQQQQHETTRAAWPLDDH

ATSNAIAAAVRTLLSAYLTDQNPAMLNRLTALLPQSTPGSLSSLFILPELEDAPLDMQQY

GNSGGSQADSHPIESTRVKKPVRPVSWCAWLLRVLLCDLEWAGRAAATTADDVLPHRSLP

PSPSGGDAPIQRKIDAAEAWQRQSKWTISITARLMMQLQQQQHFKPSSALAAAQPSHLPQ

WKKRSSVKAAQRPFQEVWRLVFLAALQGSFYHIALDQDALMAASLHRQQRSGLRGESAQA

PPILVRLCYPYYDKTLWRAPSVNLYVRLLDQWGQGEHIRQVMATVARREGDYQAEVQAAV

AAEQAEACHIKYEEESDEAHKPSSSSSSPGSPNDGAAAKPVHGVFRRYRPALSLHSCLIA

LKHCGCSPRLFAALAPGGETSSTLLSSLASDDAEVSEEGSSTAFTTAADARLAGQVLQYM

LCSLHVVERNAAQRALRGSFVDTATGNENECDAAKQEGPSPPPMTTTFTLMTEGFPLAGW

VEWVRDYAVPAVEKLYQNAGLEEEWHALGY*

>Lp_000005800.1 Peptidase C19, ubiquitin carboxyl-terminal hydrolase, putative

MTTTTSTRSAGRSGGAPVFPSPGAASTTIASSTGATPVPSVPLQAVPGSPKLNAAASPTT

QQQQQQSLATSLPRRSSAAVAVPGRGNSALNMNNNPSSAAVPLSAQTPSSASQRGFLASA

DIPISGSFSNNLDAISSSVPRYVDANSMVYTFPSFSEASFTNNGGGGMMNNNNNNNFNGL

AGTAAGQASAPVALPQAVSNGDFRLGAPSSPLSPLRRHMSAVNASSPIAVPTYNTATASA

VEELSCSSPVLRSYRQHSFSATKRDTCLARSPNSPPVTYFGSSSLNMNAPDMPTTLAASS

SMTTSGSPGAAAIHTKFLEITGHPLGLENYGNTCYCNSVIQLIYHCTPLRLRLLELHDVY

QSKKGKPGFEEDTVLYQLCNLIAIMHKSNNRSKEKYPREKIAPKDLLQCVRKKNEIFNND

MQQDAHEFTMFLINDITETEQRIMADPNNVNIFIQHEAAMRKKSASFSFWKHSKDKTISA

SSQKTSSKNDKVSVTSADNGKTKSTSQQPFSGDLTPLQVILQGQFGSLTACLECENITAR

DEVFIDLSLETAQGCSLLRCLDHFGDPEYFWGKNKLRCDECKAQVRAAKTIHVQQLPQYA

LLIHLKRFQYDVKKQNFTKKADHVALPMQMDVEEYLTDPDVIEKNLRIEQARSQKETTSS

AAGKKGDDDDGGAAGAGAGGGATNHSKDTSPDTFKPASEEVRSKLRGVARHKARFELTGF

VAHIGEGPNSGHYFTCVRY

>Lp_000005900.1 Peptidase C19, ubiquitin carboxyl-terminal hydrolase, putative

MTTTTSTRSAGRSGGAPVFPSPGAASTTIASSTGATPVPSVPLQAVPGSPKLNAAASPTT

QQQQQQSLATSLPRRSSAAVAVPGRGNSALNMNNNPSSAAVPLSAQTPSSASQRGFLASA

DIPISGSFSNNLDAISSSVPRYVDANSMVYTFPSFSEASFTNNGGGGMMNNNNNNNFNGL

AGTAAGQASAPVALPQAVSNGDFRLGAPSSPLSPLRRHMSAVNASSPIAVPTYNTATASA

VEELSCSSPVLRSYRQHSFSATKRDTCLARSPNSPPVTYFGSSSLNMNAPDMPTTLAASS

SMTTSGSPGAAAIHTKFLEITGHPLGLENYGNTCYCNSVIQLIYHCTPLRLRLLELHDVY

QSKKGKPGFEEDTVLYQLCNLIAIMHKSNNRSKEKYPREKIAPKDLLQCVRKKNEIFNND

MQQDAHEFTMFLINDITETEQRIMADPNNVNIFIQHEAAMRKKSASFSFWKHSKDKTISA

SSQKTSSKNDKVSVTSADNGKTKSTSQQPFSGDLTPLQVILQGQFGSLTACLECENITAR

DEVFIDLSLETAQGCSLLRCLDHFGDPEYFWGKNKLRCDECKAQVRAAKTIHVQQLPQYA

LLIHLKRFQYDVKKQNFTKKADHVALPMQMDVEEYLTDPDVIEKNLRIEQARSQKETTSS

AAGKKGDDDDGGAAGAGAGGGATNHSKDTSPDTFKPASEEVRSKLRGVARHKARFELTGF

VAHIGEGPNSGHYFTCVRYGPQLWRRFDDDTVSTMAERDVQQYFGVPSDATGVVTTTAYI

LLYERVA*

>Lp_000006000.1 GPI-anchor transamidase subunit 8 (GPI8), putative

MSFSFSILSPSAKLKQRHFVTVQTHTVLLLLCLLVLLAGLPSSLSLLSTVHVAAAAAASS

PSSPSRNNDNNWAVIVSSSRYLFNYRHTANALTMYHTLRQHGIDDDHILLFLSDSFACDP

RNVYPAEIFAQPPPDSTDVSGHPGSNLYGCSAQVDYSGSDVDVRRFLSVLQGRYDENTPP

TRRLLSDENSNIIIYVAGHGAKSYFKFQDVEFLSSSDIAETLTMMHQQRRYGRVVVLADT

CHAIALCEHITAPNVICLASSDADSESYSDQYEPRLGVHLNSYWMNEMFVLLNGTSCTDP

RVKRFNRESVSPLHQSWFEFNYHPKRAVISRGHADAAHRDAVNMPGAIQQWKVADFVCGT

DPVAESVEVRYDLD*

>Lp_000006100.1 ATP12 chaperone protein, putative

MRAFTSSTPCCRLLATTLATTTRAVTNTTPNKEAAAAAAAPASPSSSSATVPKVAPKKPR

RRITSKLDASGLLKNVPSEPGAHAELSTAELERKVEEMSRMNPKQLEEVIRKFDEQEDES

SRVLNEDSLYQMDVSLKPRSSGAVRVFWKDVDVVELKDRYPGWYAVTVDGRKVKAFESSQ

PLAVPSLAMACCCAQEYAEQTGYLNKLLMPMSDICSGALHIAPQMLNPRIDYLLSFYQND

NVYFRAAPIAAKQDAMIAPIVEWFERMYEMEVPRVVGIGDPHITPHATAKMRDALLAMNM

NPYQVLAMCVTAQFTSSLLLPLALFSNVVDLPTALAINRAEEHHNISEAGLVEGYHDIRE

ADVVTKICACATTWRLMEGVSLAKCLEMPRVGGAQDEAEEV*

>Lp_000006200.1 Domain of unknown function (DUF4475), putative

MPPRTSSSARPTCSAGSALPSSSATAASATSTRQRLLVSTTSRNSVNDTVRAARRPSPAK

TSTSTLVRSRKTTANTTNTDAANRPGYGELVESNQVHDDIRALGTQVQSLIARRLPRHGT

SAEQVAWGVRQFGPSRESRAVLNRENEAAKLAWETGVYCVWRCTDVPKLRAEGANNDFCC

RLGYRHVCFCGHPMAAHTVPSSCSSNSGGGANSRGATRTLSGAAPVNDRQLAQWKAPCEE

VGCGCSAFQYIPNTPLEIGEGWLTRRADWKASQWSAKCRCGHGHKAHDPSAVSRLRCRSC

GGCTGFTSAFLCVVCDLPWEAHATVWESEGTRVAAGLPVREAYAPLAGMDWDLRELVLTD

ATMDGKIEPPATYLALQARQGGVSRSPTRRRLPDPASPPKVEEMPEVPDPVLPDVEYCPA

CATIYRSAASNFCSKCGQPRPRRSSTLR*

>Lp_000006300.1 DnaJ domain containing protein, putative

MRANKLMLAGLRRLPLTSVTTGALPLAAAMALPLITHARFASSKNPYTVLGIKQGAEKAE

IKKAYRVMARKYHPDAPGGNDEKFREIQEAYEQIKSGVWIRKQQESSAGGGGGDGGGGNA

NRYSGFRYTTRTHNKSKVSYDEFYEEMHTGKVKKDPFADDEDEAEGAAAKDPRRNPFAMN

EIAFQAWLRFIIMWCLLFSSLRVVLFLIFPPKWEKPQRKPPPRELRGQRRPAPKPIHSSE

DAIVA*

>Lp_000006400.1 metallo-peptidase, Clan MG, Family M24

MPPKNASAKNNSNKHQQQQHGNGNKKGKGNRNDDAEDFDALLAAAVKATKDDAAKNHSGN

HNGKQGGGAGGHGGAPSNKSLAADEKVVPSSADHPENPYRPAVEGYPRQTWPEPTVPVAQ

QFPPGQFPAGEIMDHPGEMNNFRHSSEEKRALARANEQQVQEMREAAEVHRQVRTWAQSW

IKPGISLMLMTDRIEKKLNELIGKDGIMRGQAFPTGCSLNHVAAHYTPNTGDEKVVLSYD

DVMKVDFGTHINGRIIDTAWTVAFNPMYDPLLQAVREATNEGIKQAGIDVRLCDIGEAIE

EVMESHEVEINGKVYPVKSIRNLCGHNLAPYQIHSGKSVPIVKGGEQTKMEEGEVFAIET

FGSTGRGLVNEDLECSHYMMVPGAEMMQLRSDKAQQLLKHIHKSYNTLAFCRKWLDRDGF

DRHLMNLNQLVNEGAVNKYPPLCDVKGSYTAQFEHTIYLGPTAKEVLSRGSDY*

>Lp_000006500.1 hypothetical protein, conserved

MQRYSLRRMAAATTGGFSSAAFTGDDGGTYDKLKGEQARLRRDNQSGAADTSQNDKSTPH

RRGADDWSASGSSSRRGQGPSSSSFSTGGATDFSSATFASNDDGSSSSGGGGDATAALER

PTIDTYRAMTDEQLTDTLRLRDEQVAQLRRIYENFHYEADKHFRKMIFDYHDKTMQLSQV

HGKMQQASLQINREALARMRDEQDRMTRDKRLIFTLCTIWSLVFWIWVRRHYVQRRELEQ

EPLSAMDRAVMNPSVTGAGSYGNNFFGSSKRSGRFAETSWEREVRERREAQDARDRQLAL

LRHQEEVVKAVAARDGESEVVAASSKNTGVEQSKGRGI*

>Lp_000006600.1 hypoxanthine-guanine phosphoribosyltransferase

MSSNTAKPSSAAPTRHYPMSCQTLASQEQIWEATAKCAKKIAADYRKYNLSEQNPLYLIC

VLKGSFMFTADLARYLCDEGVPVRLEFICASSYGADIKTSGEVRLLLDVRDPVENRHLMI

VEDIVDSAITLQYLKRFLQAKKPASLKTVVLLDKPSGRRVSLEVDYPVITIPHAFVIGYG

MDFAESYRELRDVCVLKKEYYEKPASKL*

>Lp_000006700.1 60S ribosomal protein L34, putative

MSCPRVQYRRRMHYATRGNRMKMVRTPGNKLVMQKRTKRSQGVHTPWVLGHKRLGGTKAL

RHIDARLASRHEKSVSRAYGGVLSHAQVRDRVVRAFLVEEQRIVKQALKEHAKLKRSNKR

TANKKKSKRAKKEAIVKKISTKTVAKKKAPVKKAGTARAPIGSKLAKK*

>Lp_000006800.1 hypothetical protein, conserved

MFQLCFPSSPSVSTSLRSTLLTCTRRDLAVPCTSSSLHYCCCSSLCLSRRYQRSTSTRVR

SSSWEEKTQKFEERQRELQEEAARAARRRARYAATPSAAAASTAASSSSRSAMDEDTVQL

SPSSASITKPSQGFWAFLTGGSKGRRSQPMNPLMYIDRMRKKSPYDPDARAAQRLIDAVN

GLRRRNARRQDPMTVQLSEEEKQEIVNRYSETRWYGPLYRPFRHITERQIRWTLRISHVG

LLVLLVGYLAMVLVVYTKEMDTVARLSPEDQQDYAYMVQGMRYSDIFNTGKVVLDRDDPL

EALPAEVRLHMVIDACREKNWHRVDWDVELRKMHPNSAFEDRDYLHIFYWMIMDVGRAIA

GGGGLFNDRVLDVQEVRHSGEESPQERNRFVEMEPTELPTKTKRGFFS*

>Lp_000006900.1 citrate synthase, putative

MRAARCSMIAGVAGLRMASSVVDEMKDQMLKRNKVDAQKISDLKKKHGHEKLSDATVEAA

YGGMRGITGLVYEPSLLDAMEGIRFRGRTIPECQKVLPKAPGGNEPLPEGMFWLLMTGEV

PTAEQVKALNTELHHRADPEAIAAAQKVIAALPSNAHPMTAFSAGVLALQTHSKFAKAYA

TGKSNKKTYWEYAFEDALDMMARTPAVAAMIYNRMTKGQTELAAASNSDLDWAANFTNML

GFKDQEFWECMRLYLSIHVDHEGGNVSAHTTTLVASALSDPYLAFSAGLNGLAGPLHGLA

NQEVLKYLFSMQDRVKADGVNVNDEAALEKALTKYTWELLKAGHVVPGYGHAVLRKTDPR

YTCQRDFCLRHHFEDDLFKLVSTIYKIMPGILTEHGKTKNPFPNVDAHSGVLLQHYGLKE

QDYYTVLFGLSRQLGVMSGAVWDRLQGRPLERPKSTTTDALAKKFLSTSL*

>Lp_000007000.1 Nucleotidyltransferase domain/Cid1 family poly A polymerase, putative

MSQQQPHQHYHNYYMYSCTSPQQQMNYYSAMRNSQASPGQPQLTSTTAEAISGISSGAAE

TAFDPAALAAAATTAVASTTGTQGSVAPSSFSASTAPITGATTATASPTRNVMLHRSRGA

ATHGPISLGPGNESVLHPPRLPASPGALFLQGGHLPTIRVGTAGTLVNAPMPGRNSLNWN

NNANSNAALSQQESGANNGNSDRGAHPNGNTATSVLSGGPMATAGAAGRCGSPVNGGGAP

RHHHHHHAPFAFSTAASSAPAAPAVNPRHLRTSSSYSSGGGGGSPVMGSLRSRADLTTVE

DTCNSHSNVQDNTSEHGGHTSQQQQDVQQQQQQQQQEWSASPLTPSMLQSANNGWGSSPN

TNNKTSTSSSAGGLKGKGGTGGSGTNASRTNSGGRNSNNSNNATQVYSPNSNPVTVGGSS

GGGGSGSGGGGGGMSAYNNFRNAGGGGNGMAMQGGRYGGLSNPAQQSQNSYMNQVLYADQ

SDGGMMGLNTNNNDGNNNNSDPKMMSHATGNRRVAGSNNGMTSGHTANKDNMLAPMPYYD

FVTVLPTFVSTCLTMQPEDEAIREQLCWTIEAVLRRHLTKNAELRVHGSITTGLALPSSD

VDLLVVGYQPIAPLEALQTLSKALMDLDEASLKDALEFQESVEREKQQQLLREHTRDAAA

TTAPLSAAVTQTTTTTSATTTSDTSLQEAHASASHADHDVEQQDSGQNACSRSSAAPVAQ

RIDGAAAKTDTGEVSSGAVVSVVAVPSTATAAGATADVAETASQRPAGITLDGRKAFLTA

ITTAAPFFDVDDATLDYGSTRHDRAARGGGDDNDGDDGGAVSSFSAMAGSARMGRSYADA

DAIAAAEETEVYDTVDDVEQAEEVFRDQIEKDYNFSTTLDLSSLFAPVLSSGSGGGGVTP

KMPSSPSPLNGPVSLPPPPPPSLPSTHASAGRETAAAAFTTTAKSTGDAHASLSATPTST

STSSSPVSSSVSVRSFTPATDVNASAVSDAENTSRTRAQKDDAVERPRCQRSQDRDVSTT

DRHNRSSSSNHTHVEKSRDTDDKVHDAKTETDAEVQQSPPGTTTVVNAAHGAAMATTAAA

AAPPLQPPAPSTTTTADVSAKGGQQQQQQLAAAAEQDGLANVLVGPAAGRPGGHLTYVPV

HDGHFFYVQAITATRVPVIKLTDKATGTKIDITFAGGEHWRSMQLTRSLLDVFPQARPLI

LFLKFCVRSLGIGESEPGGVTSFTIYLMVMHFYNECRKRVISILQEREAAAAAATAFNEG

HNVDSSMTGGGAGGSRTIPPNMSNNNNTSNRTDAHADDTTERGSTPLPRPSAEEEQQLRS

TRGSVPLPNTTASASSTSAISPILAASPTVSAPASAAASATAPLDLGLLEHFLGEYLARV

EQRHAQLLKEKAAAAAAAAASSAEARTDDKAAGEAVAAGSTDEACILSERHQETVAASSP

AKESTHEDQSQGCCRRNSSSNNVGACATIRDAYNSSDKAHLRTIREVILGFVGKPSAEMT

GVVTNSAETSEGAEPQQQQQQTSSPLLLLRRASTVFSGNSSPQPQSAASGVNEGGEGVMT

TADTSAGVAEAARVRHAPPLHHYLLKTVEPQPHERNPAAGGGGVAPKIDEDGGLAEETSA

MAGEQAANSPREKAVNVSAEGVEGHKVKEEPEAGQCEPTATTAAEKNAVVHAMEGEVASV

LNKVTTAAEQKECGHDDDDNNSEEKASKPAASSRTADGYAITSSATAAAAPSQETTSSSA

HPHNSDEQTTTTTQQQQQCNESDNNMGVLCASGNSTKSTSWTAYDRFAEDFLQRQVNVSD

LFLDFCHYYGCAFDYETCGIRFSPDGRSEVVDKPYLCSRRGQHFHLTSPFDPEYDLTARM

THMRGFQWLCWWFTEWGAARQAPMFYGDCSLQYVLQCLSPQSADADCAAVHQALMAQSAA

RRSRESVGNMQSYPRTMNYYGMQQQQQRQPYSPRQPAMDVNRGMTPNMAYQSPQQGFYGP

QQQQQQQQIMMTRSGAVDDGYMSNGHGQDLNAEAFYIQQQQQHHQQQQHPSMSSYGQPTM

TDPQFQQQQQQQQQQSSYVLSPTDMDMPSSEVRSDGAGGHAGIFVDRSEIARPTSALLRT

DDVNAAVSQHRWPPLSNAQLPFVDSHEALEGLMVCGTSYATSATSRTTNGPVAAVGDSGD

GVGGEEEENDGPAYSTANDTTTITTVTAADSVRDDEHNGNSPLERSNEDVAVADVTAVQY

QQLQPAEEEEDDDDELRDEVGFDEMMGSEAAVMEAMMAMPFTEDNTQRNVSAQQQQQQQQ

MYGGGYAVQSTQQQQQQRFYRRPYSHGTAYYDMPQQSQQAADTASFYYHAYTNMHNAYAP

YPLPLQQPQQQQQQQYGMSTPQNFYAASPAMVYPPQHLMFQQQQHAAAAASNASLYPFPF

LHYDVQQYLWMQEQQQQQQQQQQQQHPNGSTMRRTLSREQEGEISSTSDATATSPGSPVV

MPPSAVNTIQQQQQQPYSSGYTLYPKMYNAMWNSASAGAAAGGGGQPNMQMTRPYARSGS

ASMQHLERAGGTIEGVPQQQYHGARGGHARSAPRHHVSVHETRGPTTSPSQPQQPQPHAH

PSRPSAKPHHSSSSPSHADPASATTAATASAGKMGSKGPNCNDEDVDGDSSANRQHVQSL

QLSKLADGADQEQRSQQGTPRVLSPTAVPQPRAQSLRPQ*

>Lp_000007100.1 hypothetical protein, conserved

MAKSSRSKWKKQHRRQKAKEEAVNTVKRIKQLHRKLELTIKGGISAVPPQEPEKRFHFLN

PELDPRVPNTRKDLNNNYRAELNHVHYDYAKPLKLAPPKTNVYGKSDTTAPHPMTLNYET

LDADAPLGGHAQTKVDVERMARRQLEEAAAAAAAEANANEEEEAVDTSAVEAEAEDSDGP

EEFVFGMDDAAPRRASKAATKAKKSPTTTLSKKKKSGAAAGSSSSAVTAAAAAAMAAVAE

EDAVEAQNHGSKIASMQLSHKKNGVTVSAGGASKMKKVGSRDGASGTRVVSSGGKAAQKK

TGKRKIASK*

>Lp_000007300.1 hypothetical protein

MRNNDVFSSFFASSLANLKAQSPKEKSTVTDPTAEEQLLLRGTSFGTFSIPFDTFRRANG

NATSLYRHSSLTQMGGERDPTTATAVSTTATTTTVATPYSLASAPVFVAEDGVSTMFSSP

SSSSFERGQKICREPYANCKGVSVLGERWGSGAYDYENSGRALQTTPTKTATKAPAITAR

ADVALPAIVTAPKCPPRRTVVAPIRTVQAPTPSAIEVDFSCDDDDQPLPDENKTQQFFRL

AQQHRDQRMRLIKKHKREQRRRNRAKTQAPAANKTGDSAAAASLPLPPSSPQQPSKAHDD

SIPAAKDSVQPPNYRVPHPPFFAAPAAHDARHSFPSSDGGYILDNDGNDYLWLIDDNFQA

AESVVKKEDEAEAEADDSTSSSSSTSASSDSRASSSAESILLEERELWGVCPRRDFPMVK

SPVWAMLDGPQHHGGESTSCDDDASLSGDASALLSGRADAVQDHPEDFGEVMELVVTTTL

PPVESTTDSSQHLNESTTPAGTTTAPALRSEVSMHALKIRHNPDTRLTDNSSHVTMNSTA

NLSDSPEAPSNVSSRSSSASLTPPPLLGSCEATTKVAVQHRPLPPRTSPGSRGHEPSQLQ

QSEEVRRCRQEVSIAKVENHPRIPPRMDVGPAEPRRYRRANRFRRIFKGLL*

>Lp_000007400.1 hypothetical protein, conserved

MSRSQLTIDLEDFLGHPDPNPPQTVLTGITPIAPFRGSAAATQETATTALHHQQYSSDSQ

NTLSVEDTEPVPIGVRHRVRPRDGADDSRSRESQRRAVLERHLALHHHTQRPQDGLASGD

GDLSVQGGDLSLHGPRRRLETDRHSVSRSFTDAQEVERHQQQLIKIFTERLGYIESRFEA

RIEELTRKLDDVNTQYSAKFDSTVEKMHEALTQKTAEVSRLETELKVLNENVKNDKDDVK

ACRDSIEACRRDVASLNGRIAELCSQQRTPTAAGLAAQTSAPPAPFRPSNNGDGTTTTTT

NSSNNNGGGVFVNLGAGANNNGTTSNVNNASGAFNPFGAKMADAKAPSSANGTPQPPADT

TTKTPAFGANPLAAAAPVASATEGGDNGNTSNGVNPFTRGGDSNSSKNSNSNDGTNGTAA

PKATSGNIAFGGFPSAVITGTSDATSKASPFGAAPAKAPTTTAFTAGNEASTDATASAAA

PASSSAPSFSFAGSAAAAPATASATTTAAAPPPPPPPPASLGRPTFSFGGPVTNAGTTAA

AGSTAAPAATPFGAAISSPFGGVPSSGGTAPAGSNLFASANANNSSSGTTAGVNGSPSPA

FSTVTGAVTTSGNTSNNTNVGAPASSGFSFSSAANANNAAGAKTSASPFGGGSSTAFGGA

STPSVPAAATGGAPASSNTFSFGASQNTSDATSNSNSRVDAASAAPFGSGTSTAFGSSNG

TITPSPFGGTRAPATAPSTAFGGQFNSTVGGGGGFPASTNAAAPATSATAASPFAASANG

GMGGPAFGAGVNASNNSNSGATGGSNAFGAVNTNGFGTAATGGGNAGIPAFGATPMNGPA

PMFGAAAKNNSNVGTNGGADGFGGAATMNSTGGGFGAAPPAFAQPLGSALGFGGAAGGMG

GNNMMNGGNAPPKTSLLGSTRQRQKRRY*

>Lp_000007500.1 biotin/lipoate protein ligase-like protein

MPSPVPVNIHFLEEVGSTMEVGREMIASAAGKPFGIVAAVQTAGRGTGGRTWTSPKGNMY

FTLCIPQKNNAAYFKEELVPVLSLVCGLACRRAILEVLHLDASSAKAADAAKALTTKWPN

DVIFRHKKIGGTLIENDHDYFLIGMGMNIAVGPKVTDAGREATTINAVADEFGVKHTDPK

ELASAIWEQFFDICAAPGTTRASVVADFDAVMDKTLKLHKRLPDGRDPEELTAVSLNSWG

HLKVRHTDGSTEVLSAEYLF*

>Lp_000007600.1 hypothetical protein, conserved

MRKLPAVVWPIYGAAGQRHCSTNGSSDHNTSVAFSSASSTSTTTTTGTTNPPQPRPSSRT

TVADQPLHNAADESADLAERVTSGRSPHLNAAYQAKWQRTSAKPSSSPTHDGPPQQRRQR

RRTTTTTATAFAEDSQTSNADLPMVHPGAELPPLPQRKLTEKRVGKFSFVSDPSERASRD

FYGHEVPNNTPKPKLPMWASSLTSPALGKVRLVEDTWISQDARGTIDEDTTNAFVKWLTD

TVLPADAVLRKQLKSNVVLDLRKATARGVYAKRAFKKGEVVLTIPLSQATSSSTSATDTA

AAVAAAGQETAKTHRLTLNSEVLAAYSASAHKRPGLPDYAEIKKVLSARRSSFDPIPHPL

FIDQVHAALLLACEKAEGTSSPLYPYLQLLQRDELFNDDRIKELHLGVLDPPSHMEYTEH

CNRFQHYLRELHKTWWAAYENAVNVHPQESRVAEEEEAKCTLKAVTRPTRQVLTPSSCLA

DALFVNDASNNDNKTQVNSPAEQRTISNAVTDMVEQPSDTSRSISGVAETVSPSATSAQL

PPPSLADLEWALRVVLSRQKVLPHLRLRQDAFAHISSENVEGEELNRFERAVMKGKYAFY

QHVLRAIDEDRLHVNEVDPSSIATVVPLLDMLNHPPGGVGNVTYSVEKQETVAKQEKKAV

GEVGLSSASSADANTPLSSSAVLPDNPPVSYQVVVRAAEDVEADEELTVAYTKCYSVAYT

LYRYGFLALGRREDDTAALLAANGMKQGGAQPVVGASEAGEATPAKGWWASLFAE*

>Lp_000007700.1 hypothetical protein

MSTVETTHDPHAAAAAEEERRAVPPAYENGVDAAHAETPAAAQYDSEPPAADGGIDVPRE

VAAAAAEKPAKPQVVLRPPVVARHWHVSSRPAVQATTSTTSPNGSYHCSNRAPESKSHAV

PYEGNDIVHTKRSKKTKKHSSAANAAVAAGVEMTKASQPADEAEETAAAPKKNPKKAAAK

KSEQADLEQAAAKGENKAEAAPHKAKKVKKVSKKKAAAASAEEGEAKAKATPKKKRSVTK

PKKADMAAVGEAEEPAVAEAPKKVKKALKQASEPEAEAVTAPEPKEQQTAVVVAQKTTTT

TTTVVVAKVPADEAGAAAVAAVAIAEAAPQEAEAEEAALKKDERLAGTDLPPEPSSMLEE

DVAVDAAADDRDEL*

>Lp_000007800.1 RNA recognition motif. (a.k.a. RRM, RBD, or RNP domain), putative

MTSLDKAVRPRSPPGSAAKSATEDTASTNTSSGPLFPSCCFSAPLVASPFTTSYSPFQTV

STVSGASSHVSVKQQSRAEVVARTVHLRCLPPFLKQKELADVFDACGEYLRVRICGNAAT

HQKWIYGFVEFATPEAAAAMLTHSGMELSNGPDKPPLRLKCSPSKQPILDCMAYDADVIG

GTPCGFGRGYLADCTLNDALLVVGNGSSGGGGTTAADGKGGGGRGNRTSAASRALTALTV

SSIRKVAATGCGCGCAGVNCCGCCRCWKGAKAQENGAAEATGDACVKEGESVGTTGGAVD

GGGGGGCGGCCGGGSSAEVTAATEPAAATSKPEAAGLTIAATHAPGEQCEPTKGAVNTPV

ATSATFADLSQLPATMNATELDVLQQLAAAFDGLRTTAAAEDGQLDGPAIVRRAETMALD

ALARASHLSSDTRLQDILCDLTELLSFLDASAAVTGGAAQTASGDCTATLPQRVTQLRML

TNLVGALLCLLRRSVADAVPYVEALLVTFAQIPPSSLLLQAREHEKRGGADVKSAVDHPK

EREEEGIAVFTVPGQGGGYRDTVDASQPKRVSSFPLGAFVDEDVAECLLDLVDGDDDEDD

HRHKNNSGNKKKNNSRGTVRVHTGEESTLNGDVDDGLASCEKDNNNKAQSNTGSSEVAEQ

QQLLLLSDDRSPSSCSDESTAYLNSADMADLCRRDDAFTRYVLNAVVSVGIAMERVQPVI

ARSAYTLANARAAEVLGEASSMVAASLAASPTVPRLCELLFKDTGTSGQTRDITFFPRRF

FESVNAVRQRVHDTRGTECFWRQLPPNHVVPLFKF*

>Lp_000008000.1 60S ribosomal protein L35, putative

MSHHIKIKDLREKSKDDLLKTLTEYKKELSQLRVVQQTGGAETRLGRIRPIRKSIARILT

VLNQNERSNLKKFYADRKMRGKTPKVLRTKLTHRRRLALKDNEKNRKTRHQLRMAHKFPK

RVYAVKI*

>Lp_000008100.1 WD domain, G-beta repeat, putative

MPSEVAIRCMTAYTEEAEQGDGGGLVPLFVGRDDGTVERYDAMQDAELGIPSAVFYAHRR

PVTGIVATSAAELRTCSLDGTMKQWTLGEAPPESVNGGGARVRASLVKTTTFPFGVSVMV

QESGNRLLLGGDDGSLTLMEGERRSTWRAHDGGAVTAIATDAGCSSAVVTGGADGVAYIW

DMEFGRPVCELRGHTGPIRALSFVSVPAAVPVVRQKRATAEAAAREALVYGGAEEGSSAT

CIVSCAADSTVKVWLLPDLHEANAEAELLERQQQQQQQQLQHGVGGQRISFGISFQEPNA

STTEAAADGAEDRHESNGPANPTADEAGGSETTATAAEASQHAEKQESAEATTTNGSSTL

ELDAKEQFLRQQVNASACKSAIKAPERRVVPFQAALGTVELQQVPFSCATPSSSPSAGAG

NGGGDGDGPAGPSLLFIGAAHGDVYGLRTRRLVKEVCLHTAQNFQKVQQEVSQVRKTLRD

AIRVYNGAAAAKIKAEETTQLTIAAEARRGEAAEERAARKREADERRAARRRQAAEEADD

EEEEEAESPFDQDDDDEGDVDQDEENGPDEEEEAEAEAEANADSLNTLKPPRPASWKKLT

LEQRAALEEFCTAQETERDRRIAALRTSVEAHIAQVQPLAKTVYHRSRAQFTNLSYTTVG

HLHSDTAVTAMAAASLVPGYTDKVYAAQVNVIVPVTVAIGLEKL*

>Lp_000008400.1 ubiquitin-protein ligase, putative

MSQFIFNGSSRMRNVTFARDHYKTRTSIIEDAQRQRLRREEEVRRTRAAQRLQRVIRQWL

ATLKVMRLALKNVHRLPSDVSQLLSLASPQGTTESAVTNGVETKLTARLHSACWSLSYAV

TRPLRIPIHGVATPQGFLEGQDSKEEDEETAKKKASSTVSQTFKSVAELQAYQQRVLQHY

GSLVYTALCSCARWTPLAQEGVAASPRWRHLLLSLSPKDVSLLLFVRLQQLPHVLADDKG

TAESSAMADATRGRSTAALFSELTSSLVSAVTGHNAAFCRSLNEEVEVRAKGEAGAGAAC

YSLLSSSPAYVDRWPAVQALTMVLELAVQEPARSQAWIRREYAALLRPVLSAFPAAPAAS

LSSRTVATTAAAMPYSTVDASSPSPSSSAFARACWQCLCATFSDDEYLLLTADPLVMLLS

SEEAAPLDAGVAASATVVGSDACCAVLIEECFARTALDTRLTLGDEVNGIKGGPLRTRVL

GRLVRLLPHVQKLVHSTAAYAADVVKSYLLSLSCLSERLCREPVFIESILADHYAYNTGQ

HTALTDAAAHPATTAATPSVRRSQYRLLPSYLFSSEGGLRLVQLLTAADEEREQLRLQAL

PRPTVETPSDASAPLADAAPANDSEANTAGSPAPSTAVAAAALSSSPPPSPYTTPSASSA

AAGTASTGSAAVWPTSATMEQSPLEILCNVFAWPLFTFSKPDYSRYQQETLALCAKLVRT

PQLLRRLWSLYWQSCVGLRAVLPPGPVLQKLCARPVCAEGNNGEEEKRRAATAPVAPQPL

LRGRRVPVAAAAAPLLATPLPRLPSWETHPRYPMSFYDPHPSLSIFFFTLLAHYVNVRDF

ADELRHGDTAIFTVEEACALVLALKEIVHRAHLYGVVPDSNGEAVAHAACLLLSRLHIVN

EADPFMPACAEGLWMSVGMVAAEAAVTSIVSRWDEASAAVVVEDEQAAVEEDAAAGVGGS

AAPAVTGSTSGGAGAGGGGLSVWGGAHNTSAATDAAVLSRLPGDDLMFHGSRGWDTKQRY

IRLLVHTPFLLPFPARALLLSALLASQEERWTPPSDRPAVVHRGRVFVDAYDLFHDNPMS

SNVYNIRFVSEDGTMEAGYGRGVYRECIVSLCREGFAAEYGLFRQSADGYVFPNSFSAIA

TGDPQHLQKIRFLGAMVGRALRDGVLQDVPFAQHFRNAILGRRNTLSNLKGFDAELYHQL

MSLTQLDEEALQAVGLTFVYTVNSMGVTKEVELVRGGAQVEVTPRNCLYYVHLVADFKLN

RETAEQTKAFCAGLHTVLDSNRLQLFDSNEVGKLFGGDESGEIDLQDWKEHTVYDNPDDV

NKPQVHLFWDVVESLTRKQQSQLLKFATSMTRPPLLGFSFLSPPFKLQLLSTNVSGDDHL

PSAATCFSTLKLPPYHDYATARAKIIAAIEETGTFEFS*

>Lp_000008500.1 hypothetical protein, conserved

MDEDNQYIDLPGVRETSSSPDYASYRDDIPSSVQQPPRRSTTDSPRHSNSNGHSGESSVP

PSHVKARRTSAGAPGAVVAVPGGVPLRRRDSVKHPYSLNPTQPTDKALGESSDSAAAPAQ

EPPYREGNLFRASAHSRRHQASVAVLNRVAAESAGDPVNTTVSASPDRPDVAVPVATTQE

DVKRYVRTVMDEHAATLGTWEQRVTVAEVYCDKPLRTGGRPAALRAQDRGFLERVLSSLQ

AHLEQLEDARSLLVQTRAEVPRTSAGGCGDLDAWDEYCSCYEQTLVDLTTEAKKMRKRVV

RLLNGEQQDGVRTHSTRVGSVPQSGNGVSRRSSSHGTRRGRQLAPSEGCDVQDVSAHANQ

HTSVVDASPNAMTNNNPNGSAAAAANGRSPHVPLLGGGAVQRGSHAIRPGHNGDSAARAS

PVPESQRSWTRGSSMQNPLRCSVGSAQSLTSSRTQQQTRALHPDTVVRLDVPTTSPNDQQ

SPYGNLMPPKRASSPLRDPSHAVLVVPPPADVVAERNEEAEAEAMAMSATARSSHASHVS

HASRASRASRASRASQRSHAASTANLASEMPPQLRTYSSPRGSARGAAPTSATPSVHPLR

RRQLLEVIEHYEKHSATLQRGDLHRARKCFYELYGEQQGLHQYCLWIDDIALNALRMP*

>Lp_000008600.1 adenosine kinase-like protein

MSGDEAVPLYVQCNPLLDVVAEVDDDFLKEYELEKDCAYVYNPHYRKLFETILNHQAVYA

EPGGAGLNTARVAQWMWHNVLEKTNGHVMYVGCVGKDKYGDQIRSAAVKDGVTMELEVNE

TQPSGLCAVCKVGDARTLVANVSSASALSDDFLASSAVQQGQRSARVIYTTAYANVCRVQ

QTLRTMASTRTRTLPGGHKQLTAMGLSNKNVLEEFGEDLVDVLEKLDIIIGNRDEMADLA

MMLQWVPTDMTDLELAEKIATEMMYDQHTVRRVIMTRGLQPIVYATSEKVVGEVAIVPKV

PGKDKLSKTGAGDAFCGGFLAAFVTNPNHLEYCCRMGARAATYVINHDIGTLATDEEAIK

EVRASI*

>Lp_000008700.1 adenosine kinase, putative

MTALPQLYVQCNPLLDVSAPVNDDFLVKFHVQKSSASLLSEHQKNIFEELEKLPNVAHVP

GGSGLNTARVAQWIAQKPHGEFTNYVGCIADDHYGQILKSAAEKDGVNMHLEYTTKAPTG

SCAVCISGKERSLVANLSAANLLSSEHMHSPDVLATLQRCKLFYLTGFTLTIDVNYVMQV

AEAARTANGQFMMNLSAPFLLQFFSENFLKVAPYVDVFFGNDDEAKALAQLMKWDHQDTT

DIARLAATKLPYNGTHDRLVVVTQGSAPTIYATRSGKQGQTAVPPIAQDAIVDLNGAGDA

FVGGFLAAYASGCPIERCCDVGNYAAGVIIQHDGCTYPEKPEVSP*

>Lp_000008800.1 amastin-like surface protein-like protein

MAKKKSFYQQDYASHIGACIMCVASFLSVTFLSCGAPLGMLMLRSWSTSDPDHPGVYYDR

PCYTLWGMRNNCWNANYTVRIDDPVIKSCPAMRRRFEAAEAFIVVALFLLLFVFGASWLK

ICGSNIKTIVTLLAVFTLGCTIVPFAVVTSFYYTSYCNKSFLTHRNTRYGAGYALTVTSF

CIQCVGLILFLALEPEVVEQKRASASKEKEGSDAASSHESSH*

>Lp_000008900.1 surface protein amastin, putative

MECKKAADLFKVAEAFYIIAAVSTCVATILGGVFFIGIKTKIALYVLTALNIVFTLIPWA

VMTAVWYQDYCDGSKVVINTENGHTAGIPYGKQLRDNYKTSAAYGLTIAAFCIQIIGLVL

LCVM*

>Lp_000009100.1 Protein Associated with Differentiation, putative

METHEKVEIVKLATGPQKPVNEYKRFAILVLGSFGCIVCSFSYAWNLVASSMQNRYKFSQ

RDMSTIVTVGLVLQYCVLPYAFLFDWLGPIPITVLATIYFPLGTLLMALCFMDKIKGGVA

VLSVFNALMSCGCCLFDLSCCVTVLSYFPTNRGPVTALLKTFTGLGSALVACLYAGYFNS

DPKKHFFFLFALSIVVGGFCIAFLRLPSYHLTQYEQKHLPIEERQRRENTKAQYLRQKVP

LWRFVFGFVILIILIIFLPTQSALTSYKHLGKKPKLAFAIVTTILTLLYLLVAAPVPFWD

PVPQCSSEDSTEDVIDEANLSAALETGSPNAEKDDFNEPYCDSDDAVGNDGLSKGYNDAV

VVEAFKEKEEKEEKEKAVETEIDYIAPAYQGSFLHNLLTLELWALWWTMFTVVGTEFVII

YNATNVLAALDGKDPSSSLSTLLTVLNGVGSAVGRLLMSVFEVWTQKRKAEDRIPITVSL

FFPTGSIILSVILFLVLPGAALPLPYVVAALGNGFLAATIVLVSRTIFAKDPAKHYNFCF

TATMLASLVFNRFLFGEWYTVQAEKNPEQNAKNHRCYGKHCVLMPLVVLMSLAATAFVTD

FILHVRYRSFCQRALAERARLRELEKGDGGLSTVDDGLQLENIPSRAEADVGAEVKANTH

SDEEANCGL*

>Lp_000009200.1 hypothetical protein, conserved

MTATTTPNSAQRSTLSPAHGTGKTPTPKPLVISVPPTPPLTAEEEGSSDAQAAARPPPPP

PPALSLATPPSLCPQAVAGPADVSLSLGNAQAASVPLPPTCFDASSLRLKLSYDIVLMLH

ADVEQEGREAILRLFKSQREATEASWPQQPFPRPSPTQTRKDEKEEDCLYDTGAVTEPPE

ERPEEEEEDPRSRGAAQSTQDESVAADNVLSKNTSPAGRGAAAGSLLSTSFSRTSSNKTS

GASSCLAGKHGSTSASATSTPAVADAGGSAPHTTQSAYVLVARLVAAATDEVNLLGVDDV

TPHPWRRWAPHW*

>Lp_000009300.1 WD domain, G-beta repeat, putative

MALSPIAYISPGCTAQCRQCVSAKPDSFAFASVQSISLYRVSTTNVAIPVTTSSDAAQQQ

PSTLETVSIADYPLTNLFGHGANASIGAFAYNDEYMACLTPQNKQVLMWRLKDAESLTAK

KIGATSLATVFKRDGNPSTMCLAGKHHILCGTNTGRLISLNTSVENAEPRSTVIPPSQQQ

RPNPQLPLSPLSPSNATGANANAVAVESVECVTAAAARPDTVACGTSDGTLCLFNLNAST

GLRVTASLCPFPPKEKEKEKGGAALDITALPVTTVAFDPTSAQYLAVGSQDGALALCDVN

AQNMVQTFDFAKLPEKHVSSIAWIPNEAGAFYTASTDSTVLRKWSVTSKSSVGAVSVMVS

TPAPQQDTQDAESSSETGTQVIGIRSVACIDQHRVVVGLTDGAVKVYDVAQQRLDCDVVT

GHTDAMLSCRLSKHDCDQAATSGVDGTIRVWNLRTLSMQYSIRAGPVMVHSVDWSPNGKH

IAASLGSGEVVMYSTSTYRESWRTPIFSDLVYRVCWASGDSSLIAATSRTGLAMLSSKDG

KVVRRYAAPHGALYGVDIEPTKSKAVAVACHDKRIYIYNLSSSSERPALVLAGHADAVCD

VAYNPTAPNYLLSGSYDGTLRVWDLSSNDAHTISVSSRALKGHSDRVRSVAWCSLAPYLA

LSGAADASIRLWDIRNGVPITTVRGHNSDVVALTSHVDRPLVFLSAARDSTLVAWNVALL

RQVYLDAALGTLEGCIVADPSSLMGVSTANVTVSQVAGPAVQRLVKELSECASKPAERLR

TLASFFEFPNGAAEVAQMALYTVDPAQYTSLMNEGKPTNAGLVVPAAALADVARARATYT

NDRAHGKTVNAAGAAYKKQRLLEAAEELLRTGQLEAYCNTLMEAEEWDRAIAVGPAVSRA

FWRGVCQKAAEAMEAAGDARAVTYYIVGEQSYKAAQLLTRLSQRNYDAATVVCQTCPQVS

EDASAQQQASEPPHHTTIDQRGVEAMTQQLQRQRSAVLRRYTNPQLFAAVLLAYGHNDEA

VRTLQYCGDVVLSHLLVHTVPLREQGSIDTAFRLSMLQSIRQHKWDTALLCATRQSNPYD

SLATVLALFQTAQGKQLAGKMPAQSLTSANLSNFHGVSERLKSFYDQVRGECAKLQLPLD

AAAIQQRHANDGLASQNQLAGMVLAADPSSGPVTDSTILQSLTGFMESLLNVALQDADGA

NAPFYLRQAYNVSAYVSVPLETPTKPSSSSPSTSAVATAMSPEHKHFLALTFLVATLMSV

KVYRFPKFLNYAFGKARDLASGANNASLNTLLSTAQGALGTYSPQSKEVDCSSVGSTLPA

LTSEGRQIISALTGDLVCGAVHVLEDGSSFISKSEALAWTLCSHFSPLATGARLTAL*

>Lp_000009500.1 hypothetical protein, conserved

MADTSKLRWALYENVYQPALVFPPEELGDADLPAGHSVVYLLGLDSNVVVPESDVSLYDP

RDAEKMSNPAAALGVALAQQILEGFPGGVGGDARVDLEAQESHHTNNSNNNNSSKSHHHH

HHHDDDDEEGEADASQLRLLDDDEHDNSDDGRNSGDDGGDDEADARRRRHEEKKLRREER

RKAKEAKKAARATVKREREELVSAPAIRHRDTSDSDYEDDDAGLDHNNSEDDDDALLAAK

AIEKKYSRETNKQKAKTRSSSGKVKLEKLDEEEEDAGSGDPSHARSGKKKSATAPVEGSW

AALEEDLFSDEESEDNGEEVGPDERPHASSRAHHGLRSGNRMGQALAALNATNASHSIPG

LFGACPYAQPYLMEIHYEYEQLAQEAANEGLLLTLQEGEVVRAIDEDLRQKAAQKVYLEE

ALSARQHRQDAAAASQATNNEMDALYDAIAKLEAPLRVTETVRRLVRKQMPASAAAFDRT

AAVRARRAVQRRTRTFQDTTVVALVDHLRSVDAAPREDGVEVMRRYIQQRRQHEELKSNF

RGLSKTGFYAVPKPLEKWRRARDMMMLANVGVEAQKVLPTSYISQAHSTMVRRMKEHAEK

RYDRMPVTARDGASFLDSTSFTNNAMPSPSALSAGLTQQQQRLAASSASVQRREAFFQFH

ALRCDTTAPVTVETVALNADFAGTGGGLSQSVLASVGDFSASMAPDAATVDRVGMNDTVN

GGHVDDNSVQTDEGLSQTSYSVHSAAPSSYPYLFDSEPQPRRSGHHHQHHHRQRSSNASG

LSSLSEGASSAVSSRAASVGSAVSGHPNDEENGEANEGQQAGGVPRRPHRRGMTSKSAAS

APAAAAAAATATSTTTPGASDWRANAKRSIMEQLTLYCRGRGGKPAILSTDQCREIGRTL

LDRAMRAEAERQGVSLAVQSNNLAAPFTKTTEQRLKKSVDHYLERRIAQRTLPSVGGEVV

AGMAEYNNNNNNGGVNAVLPMPGAATYDAAAAVARQRAVADTPVYEN*

>Lp_000009600.1 hypothetical protein

MSAYQKNNSVEVRRAECARLQAKYPAHVAMVVEAATSSKAHFLALPRDATVAELEAAVRA

EKAMGGVGGPCFASD*

>Lp_000009700.1 hypothetical protein, conserved

LCVRRYLVHTSEGNNPRQVPLYAFVTVRCACPSVDRELVSRVAREELPGLMKADKSIEKE

KQKQLQEEQQEAERAAAHESETASLMFGRTGVLCRTRVPHKSLIAGVVLAALTLIAEVYL

CVSLSALTDNLIISFLMEGLWFVVAAMVVWVAASVTTIFHAFFMRVPLRDNLTLLMLRAF

FCAGGVGGSLMSLVVLVTRMSSVEIHEFMTKQSKDDLCAFYQAHQCTGFYSSCAHRSAAT

TGDALCRPSCLVHVAYETTCHSAMTSPVQVAFLPMMVFSVLSLFACLYSLFLLIRLLPFA

HSAARRRTS*

>Lp_000009900.1 hypothetical protein, conserved

MPSRAAYTAVVTSLLNHVRAQENAVQAEGEAVQPDARQREREALVTEVTERLQQNLSSYA

VSHLVVPVDKPIEDLPSIRAPELQLIGRCVSFGVKQTTSGGSAYYHYTGLVSMVTASAVT

LMHVNRYTETDFKAYRNRERKATHEPCPHDFSESDGAAQTLILAVNDQVSATRESSQREA

DVSHFPCRFSEVFGSAFGGSTRAPADLRGSLAPAAEMVTGTVLDNLAAHHRHRESTAART

QEPPVAAAMASDKQRRLTSLRNFAGSVGPLPYVSFLRKNIHDVAFGRSPDSSFFSLFQNP

SKHTTDMQYLRMFVRRYLVHTSEGNNPDHVPLYAYLTEKGACSDLLNHDHITQLVREEVQ

ELTKSDHVIAKEKKRMRNREARREATLRDYRAPSGIFADTGFLYLTGLPQGTLLTAAAML

FFACAFAGNYLLPVVITGDGLINSFFDDMNDTFMAAILTWVVASMLAFFHAVVMRVPSIA

NSPLLLLRVLSSAGTVVCAVLCLMLITRCTTNEYLQQQMRVSDKGELCAFYEQNKCAGFQ

VGCSVYGRNDPLCVPCVPVSYPDSPCYSVIMDKLQKVVVPLFLFSIVIFLSAVHSLFLIF

KLFLVRRSVFITS*

>Lp_000010000.1 hypothetical protein, conserved

MPNREETEYSQRGTGRESDERDASLREPVEQPAMFEGVPVTQDGNEEPKQSIDDIVANLI

IHEHKPIENLPTMSAPELKLIGRCVSLSIKQTVNGVTAPYYYTGLVSAVTAAAVTLMYVN

RYTEADFQVYKEREKVAVRAGTASAQEMTQRNASHTDHHHSTHGRSPSSEIDQDQQHVGV

PAHGCSVKGEASLDHNGELTGQRDAAAALPLHVDAAGSLAPAVVMNFDYATEGDKQAFNV

AQDGEVEESSNDPVSGAAGPPLVLRPRHFRNFAGSVGPLPYVSFLRKNIHDVAFGRDPGS

SFYSLFQDPSKQLLDMQYLRMFVRRYLVHTSEGNNPRQVPMYAFLTVRCACPGLDHELAS

RLAREELPGLMKADKSIEKEKKRNRNREVRRDLAIQEYHAPPGPFSRTGILYLTHTPQST

FLSGVVIVLFAVAFVIFLAVTLAVVADALMMLYLTQIMSYFIASLVIWLMTGVSIVLHGV

IMHIPLRDDLQLMVARALLTLGAVACSIMTLVMLLGRLSNRRLYHFMELQGTDLLCNYYN

RHQCSGLTEPCTSPLASTDALCTGCPATAHYPSVCYHSLWSQLQILVIPLLVFCVFNLLA

EIHSISQFVKLWLMARALNDRVRS*

>Lp_000010100.1 c2 domain protein, putative

MGRLEIRVCGARNIANLQKVGKPDPYVKVKMGDRKKTKIRYKTRVIENNLNPVWNELFKF

QVADYDSTQVVFEVWNDNIMVDDLLGSYSLSIDGLTRGVVRDMWAILAGTKGSSSELHLR

ILAVDFGRDPSPTDVIISSLEQDNLAPPTNQTYRPPKNYTPAPQVIAQQAYPAAPQGPPP

PVVYGQQQYGAAMPAYSAVPTPMQQQPQRPYGYGAPPPSQQPYGYGAPPPPQPMFAAPPP

PQRPPYMGGGPPPQSYGPPPQQPGVQMAYGVPPDM*

>Lp_000010200.1 c2 domain protein, putative

MGRLEVRVCGARNIGDTQKVGIPDPYVKIIMGDRKKTQIKYKTKVVNNNLNPTWNETAKF

QIADYDSAQVVFEVWNDNIMVDDLLGSYALSVNGLQRGVVHDMWAILTGAKLSSAEIHLQ

VLAVDFGVEPQPGSRQFSSIEEYISNTAAKPVMSATETSGAPFTTSNDFDPVKKPSPVMG

IPLQPTQTAAPPSYAQQQVQYVQHPPPPQQQQQFYPPQPQVVYVQQAPPPPPQPQPQPQV

VYVQQPPQQAAYYQQGPPPPPQPQQQYGYYGPSAPQQQFYYRPM*

>Lp_000010300.1 hypothetical protein, conserved

MASMQHDFIHPRQLDDVKAIRAAIKSFTANMKSMMLLMKDLGTTLEQVSHSFDALTSLSF

SDDGVKQYVHHFSEEVVHMMEGTAFQNYNKLVHEEVLAPVEQLRASLKEAEKAAKTEKSD

FDRYKKAKQKVDSQEKSYATKSKPLDTSKSYPRNVQSRDDSLLRLQKSKDDFENKFVGLV

NEVEKVTATTLKRYLDLNAGYMTSVVDALTKTDPTVEEAVALYREEQRQQRQSAIQQRCA

EVDTQFNESYASQGYRLGPTNTNRTTTMDNTSNGKPTLSSSTRQQAQQVNAPNTVVAAAS

SPVKETQPSSITPVQQGPPAAVAVASGSPTGQKPNVVQRGAFTVTTTGANASPLTPPAES

AEAGRTAGYVDEVEEEDFGSVSQVGPAGRVAPPLYNFSRGPLSQVSSVRPQTGYVAAENA

NLTSEFLRKMESKSLTASEVCATRSPQPWSN*

>Lp_000010400.1 hypothetical protein

MDFTNDPFVFHAILMILTPVTLLVFVLMWKNAREVQRLKANAVLQPPCLTSSSQPPCGQP

TYIDRFTSNSPYSSADATTGKSLLLTADDNSTYSYFGDFLVPRMWLLTPEDSAAVDSALR

CG*

>Lp_000010500.1 hypothetical protein

MTSAADEIRSRQAKRLLELRPIVDELEQASRDALKAWKFFTISLETIAHLNDKLASAFPT

AEDAKAVGLDASIVASADTVKLCKTFRKTVNLWSLSDEVVDMRAQLYEEWAVWRRTQRRG

GNVQALALERRQEMEKFAELQKSATALRHRSNSISNAASLKKTLERKMKSTEESMKKKDD

EISAEFKNLLKKSVLREHNGSLKTGYALRNAGRGMVKAFDGFSNCGELASAVVPEITRTP

TIGVPLEINMPTALRTQPATPTAVEAVYGLGFPYNSQASILNDDVTDGEENQPISLTD*

>Lp_000010600.1 hypothetical protein

MTLSPEAVWLQDEWSAKLERTTHELAKAANEAAKKYEAYLTAMTWVGQLYAACGELMVSA

NTPPLALAEPIAEAADARVVTGRLMSAVEHWRRSADEQALVAFLASQHDVWRRWREEATR

THQHNNTRRDVMQLVDETRSRLAAAQVGDASRTATKKLETQMRDLDEELAKLHNRVQRDM

TNNVKLCSRDLVKYGGRLVDVLSASGYNATCCFQAATAPQASSRAAAPATVAAVSLERPT

SSDFTNTPSDFST*

>Lp_000010700.1 hypothetical protein, conserved

MASEDYVMTDGDCRAQKEWVFKIKSVSTSLARSAQQAQRNFDQYLTAMELVSALYKDYAA

LLSTANEPFLMIQADIPDLAELKASTARMHSAVHKWEQSENLYALRNHLHMQTDVLKSRS

LLAAKVLDECTEREELYSRYTTKSAKLARKLRRQSPEAEALQKEVDKLEQRIARVKEAVQ

RDVKAVAVKSSASLRDLTMQFFKAVFKNGAYLNKHFHPERLGVAEESPVSVADENMDDVE

TGVWGLPRSPRSENSSIAPSGIYGVNHNRYHPQEAQPYAAASVNASQNPGIPLRYDAVTA

VAAATNETERSG*

>Lp_000010800.1 hypothetical protein, conserved

MDLVLDTAAAVERHRQSCWLHELHVEAKRLQKQAQEAQRHYTATLSTLKSLAHTYENINC

LLVSVSRYFSEVSIGSRIGVGTNVEKFKNLVTTMEESPQCKKLQVLLKKQIDDADELKAL

VKRSFGALKTRDRHHRAFASAQPSATQRSRQLEAEVELGKEVRYIYAAASWVLDKECIGF

VRAFTGGASAVIQLVQDRYTRSLLAATDAVGASHGYSATGVVQNPTVSQETRPTTTTATA

VAANTQSTEGNTQAGRYGLNDSDYVSQPGSDVPVAMQPPSPLLPPSRQQTDAGVVLRFVK

Q*

>Lp_000010900.1 hypothetical protein, conserved

MPTSNPQRNKAPASPAAEEAKKAAAKRFRLEYRRPWLIFYPTAASFILLNYLAFCTTTDE

AGTTRLLPGYIASYAETRGTTSFESVMYNVLLFFEEKVMGSLYQLGVLLFRSLFGIQMVC

VGAWLIHFFEIGMCARICFSCNATAGTTALSLLCTTLAGFAQLVPLTSSRDAWVAKVKKV

ASEEASDAAAQKSKKAK*

>Lp_000011000.1 hypothetical protein, conserved

MDSTAQWCTSTAAGVWDKTRSTVQLVYHVGDQLRIVYNTANSREERQHDFNATMLRAGGF

LGAAVLLIIVDAHGGVPNTWRWLRAMLAAEDPLSGPVASAEAS*

>Lp_000011100.1 ABC transporter/ABC-2 type transporter, putative

MSAGKVESPPPLPPSSPSLLPSAATPRQHRHRRHRHHLPTSPGTNNNNSDAVPSNSEDPH

PLVADASHSSVASSRSRNRRSRRANAADRAGAAFDLLGDGARNGLTLCCDGVSVQRRHRD

VIDDVSCLFHGGRVTAVVNCCGTHSSLALLAAVAGRIDCVAGNIVMNGVPVSASTYQAQM

SFLEEKDALTDDGGATSDLLTELTVRENLEYASALRVANSAQAYSVEEVLQQLLLEPHQH

ARIRDCSLYVRRRVALGKELLLNPSVLLLDEPMGGLATHEAQQFLTILSKMAAPSAADAE

ARHIMREAMAASAGAEPPSSPPRVSIYTPRHSRMSAQASHGSGSGLSPWPPSASAPSSAS

AAAGHDSYDTQGGLNTGNSDGVAAPVFGAMLSGETQRVVILSMVQPRWALLQYVHDVVLL

ERSRCVFAGSVQEMLSVKLPKAATELRAAEVIGGTSSAAVGAAAASGVREGDAGGNYPYH

DDDNDGMDPHMRSFTQVAMSSFAPVEDAPAGSYDAVAASIHRTRLNEEFVHGLYRLATTA

AAAAAVAAAGGGMTLGNDFPEYGSFSDNNNNTNMDTSGTGARSPASVEQQSRSSLPVPVR

ADNNYATPISQLYVVQLGHTKAQVTAYMEVCAAGLLALPEASHQAPSGFVQLLHLFRFGF

VELRHKWLGDLIALVFMLAIAAALAAVYGVQAGNSGMQNCAGILFFIVSLVVLQAVLSLD

AQRREYAAYYRYSRSGYYSAWTYVLFRAVTALLWRFGLTSFIALVVFVLSNFGEPWREYR

GVFEFGVIMAVLSFCCYFLVWFLCAWWPSDRVSRFLVFTFYTFNIVLAGLVLNLTTLPAV

VQGVSFLSVMRLAYESSILTHFAAKSFGCDHGNNGTSSSNSSDDPSTGTLSSMLPCYTGA

EYAAYMGFKAARRWPNVGILAELSAVLLLASWLLMALYHPRRRLKAIV*

>Lp_000011200.1 HEAT-like repeat, putative

MSSPNTSPQHRSNTDTSKKCSRGLATPSSSTPAPSMNATRQTGGHHTRTARDVLLLIHHP

VHTLSFLQELLRPTAASCVAAAAEMLHVEEDAAAATVSAGRSASTSATAAWSKTDSGSAS

AAPLPSLPPPPPSYAAAAAANATPSSDKAMREAESGSSVGPQDSLSASAAAHQPAVPSLT

LLLECTTQLSVLLAQWRACNAALRREAWPRPPAAPSPPAAAAASGNRKSAGVAPSSPPPP

QQPPTRPSAARLLQLMNVILWYCLATADDVRLAYGGRSHGTSGRATPALPGMAAIVAAAG

GSQPAAATTSAAVGGTNCNTPSGSRGLRASRSGSSNSSADPMPASDWLGSRMSTTASSAA

GVPTVTMSDDQLAEHLFRPVVDAVVLLQDQPSLTAAATAGSGNTNSGGSSKHEEKGGKTH

KTPATASAAVAQASEEEEQEDVREELQTVALWMLAAVLVRFADAPFNATVFLPVLFPQVD

IRRGSGAAARHPLLRPLLRGDTASTTTTNTNATQTGGRHSGGGIGGHTSLRYGAAAALAA

LLRKLQPTLQYAEEPHPNRQAFVSLAAQCGTLLLSLHESLRWGFENEVTVATRAARHAAT

ANNTNTSNDGMTATLLLSTYAVVVGVTPYNRCPRSRAAVLQTLQLPIIRTYLADETCAEY

VPTTVLVSSVFKNESMCGAAAQLLSGSDAKSSSSSFTVTAGHGDYVDSGDSGKIPAAAAE

AAAAAKASSAFLAALLQHADTRVEVWRCMVPLSRLYPRIVHNEFDLLMAASVRVVATLAK

WEASEQAEEKAALTAATAKEVAEGEEEEMAAATTQGATTPRGRQQQLSSPPPQQQQQRPA

DPSFTSTPVRANAIRSIHRDPNNDSRQNEGNGASSPCSLQPRMRPPPSTPPEALAECLRT

WLHFMGYVWKSFDDNACDPALRPEGQLDRATLAQKQRIHEELLRPAMRLGRCGEEVRTMT

LRCIAQIGNDYLSTITDRSLGEELVQYVQVCLADLQPRVRGEALTTLGMWLWQYPSMDGF

ACVAIDNAVHSLTVDPNALVRTKAAFALSNITGRLPEGTCPTVRDAPDYISTLCATAMHA

AVIDSESGVQGHGIRMMNHLLQVLDFEELISEMAGFDEGVAEGFLRVLLECLRARTRSNN

HRGDGGANADGASRYAAPREAKHRWNAAHALGMGLARDVVFEAEPKYALEAVDALCTAVV

RDHIFKVRTQAAGALGRIPGHCLCALYSAEDLTPRVVRSLCEALETATSTENFRQYKEQG

SLHDALRATLAVMITTARPSIELDKVFASYQRLLHKEQLL*

>Lp_000011300.1 hypothetical protein, conserved

MAFFLKAIGLSDDIPGFPFTPAADDPGHVVYTSPLMCWVVRAGTPHDDAHKKVSIFTCTV

PSSSSSSSHNNELVKQMCRNALRRAKSVMIPGFLKCFGAAEYRDTIYIATEACVSLKEVL

ESRELRTRLYGTAPAEYDACVAFGLNTIGEALNSLHQNRLVHGNVNCQSVFVLPSSGVWR

LFGLELVSSSEEVVNGNSSVSVFDNARRAGVLEGYRCPPELSSAASSSASSNNNNNNHGS

GGSDGGANADVFAIDSWGMAALLYESVGVTAEEAFDGKLSSIMHTLSSAELRNACRQRLP

KSLHSGCAGITAANPRLRKSVQTFLEHCEFVKDCAFVQYMKRLSEALLLDVAQQVRLVES

LNEVVDTFPLRPCLCCVLPQLSELIRAAMKLHNPNGAAGVSIGPVVSPVLKIATRTNAGD

DFDAYVTPVLVQMFQCSDVLMRYNLLLGAEVYGGKVSPATLNNTIWPLYAKGFQYAMPNV

REYSARGLVHLAPHLSEAVLGDQVPRALGLLQRDQNNALRANATIALYLISGYITPPSQR

ALVLLNFCRPMLRDAFEPSRVAALRSLYGVVDCLSAKQLAEGVLPAVASLTVDPSSEESR

SAALALIRVAVSRLEENHKQLLEAQQPAAPADAAGPKLGSTPLATSVATGDGESTSSSWG

WGFFKWLPPATTETSTAPSAATSVVGAASAANGAGGWKASPLPSTASTSPTNAVTVQPAP

VVLTAMAAAAATAGGGSGWSDDDDNGGVLNSAGNGGKAKVANDDDWDDEDDGELVKPAAS

TATPAMKLHPLRQTSGMGVAPPARVPTAPLSASLRPSTLSAGAVPAGTASAFTTSLTPAT

SNASGMSTPAGSSGSPTCVMPSGAMKLRKKGGLGAARLD*

>Lp_000011500.1 ATG8/AUT7/APG8/PAZ2, putative

MSAYAASTPVEERVAKCASLKADSCSVPVVLEGAPGGKVHFSVLPRDTTVAQLMSVVRRF

DDAGAKKAVSLLVADCAVAPTTTLGELHDACKREDDGMLYVTYTSEAAMGGALDFCCLHV

D*

>Lp_000011600.1 hypothetical protein

MQRLANLLRVRGANLALRRAVLAVPAPLSTLQRRWVSSAPPSDAELPLTLAGNAQHEHES

HHHHLPSTDVRAGDDADFDEDFDELEAASQELHDLCYAMIAHLRFADPQADKSLFERRVK

RSFREPKPERTRRLIH

>Lp_000011700.1 hypothetical protein

MLFFLLLWSIGLSRTASCAARTLDRIRPQRQLLLLLGLVVQRCVGLVFHCFTHITAVGSS

GCARRTEDRLRPERVVVFVLFGEIVSVLRHHGERGGRGRRCNALSGHAVIRTGGIFLRRI

FAFLSSLLCLGSTGLTGTTDRALRTDGGTRLQCGVLVRGGVLRVLRLAGSRRVFRAGATG

RAARALDGAGPQGLLFLLRLVVGCAAFTRSWRAVIAGTARCTSRALLREGP*

>Lp_000011800.1 hypothetical protein

MGGGVFRASCGARRTKDGYGLQGLLRLLGVLIISLFLVWLRNQLWRCRGVASPGLAMQLR

CTCRLGRLRRCDGRVVHVHGVIVVDFVVVLRITSRPPRSLRRGRLQRRLRAFVVGSGGVP

GGCGVVNRCLRAVIYLVAVGAVAHLRTRRHLFLVLVSGVRVSRRLLRRGCAGEVGVVQRA

GKGRNGAAAGQPAWLSQSLDPRRLLRGRCFVEAVKLHFRLCRYGYTLGLVLTPVVPRVGV

FLVFVERVRLRIFVAVVIGREAALRRLAGPLGVVVLPLLRLLLGVDAAKFVLQQLVLLRN

HYITYIFHLVLGTALELEAVAVLGSQQVLQELLEGLVLARRVARLRLSRRGFLRRQARAG

LGAGVLGGRRRVGDGVLRVGRLRCATRNGRRVGRRVSAARCGPRAGAVGGAARCVLRCFR

CRHMVRVHR*

>Lp_000012000.1 JAB1/Mov34/MPN/PAD-1 ubiquitin protease, putative

MASEVVKLTPYPASFIIEHAKRRGFAAGYVIGTRTTEEIVVTDFIPLTHKDTEVPSTKSY

REELLRRRAAKRRFTKHEVIGWYSAGRVDDQPTAEDYQRCCNAPSVIFQNRSCLHLHCEM

PCDNAASPNITWTATTISENPEDHTFKCVDHKVVLAPLNSLASDVTINHIVSLALYNGGH

AYPRSKLLNVDEVAYVASLDNKTSTDAVNEEQKKLQQAVSTAEQIVTGGSGGSREAHEMM

TAVENFRAIREEALQRQRDQTGRVDFNSQQFKDALMIKCAATVLRKELITIEQLSTVYGE

DKERRANTEGGEDGVAKEKDQPSQQAAAKQKQ*

>Lp_000012100.1 WD domain, G-beta repeat, putative

MATPYIRCPTPALAEEALKLEWAFGLNNDFKGGVHNLSTGVEQRSVFYALAHTGVLYDAI

HNTQRHLLGHRYSIISSSCCAHNHRFIVTADEGDAEVRGRIQRRNVLTQTAKSHGDADDV

DAAEAAEEAALHVDPAVARRDDEEDGSTMIVWDATTALPLKVIATGKYGGVLSVAMSPDG

QYLATLNRRDPQEIMIWSWTQQQPHAVDAFASVDVAPVFVRRIAARDEQTSVAFAEDHPE

LLCTNGLRRVTFWSWREGILKYYSPPILQRRLKASVGNFTHTVFVPGTTMACSGTVDGDV

VLWSLQPQDRVMKEQDKTLFKMVRVHAGGVTFLTTVHGYLVTGGMDGTVKFLDTKLRLVA

WFEDLNGGPIVSISFDRAPSGTSYEDARAAAPDEVESHLLVDGTTAAAMFTAPDFMVSTA

HSMIIDVPAKSFHTTGGQTHSGRCGAAEVARGRLIVQGQDRAVQCLSAHPRLPRLAIGGY

SGNLHLWDYAQKCVVLLSLFRNLLIRSMAFDPAGKWLVVGFTNGVIKVLDAETLEEVQTL

RPTSSSSTSTSSSSGATATTTDVGANSNTAGEDGAVASNAAPTNVKEAAPSDSKSKAGST

NSIGGKAATTTTTTAAVNNAAPPPTKTAASVEEICFSADSRFMATATADGHVGLYEYIVT

SSTNAPVTSAGGAAGLPKKQKTEWQLVGHHKTHKAAICGLHFSADTGIGEGADVRLLSVG

ADKRLIEYDLENSTPEAGLVLQAAHKISQESVPTGFVWLPDGAALLDAASQPRDFYESME

NSQVEEREQQRGSSTALRGLDQRPSSAASPTAQTAHENNGNIHDALLIANNEYKFQVYLS

NWSRQCVKTVLAPTFGGPVARMAVVPVQPPETQAGTTSEKPASTSRASGAASHCLVYATS

EKVIGLVQLPLRGDPLCSIGLLAHPGSITSMVASHDGAYVFTAGGPDQSVMQWRVRGEAI

LPPAEAAAVRQCTEKGGVPLRHLVSAVEGGAEGELMKEVVDYFYYAQIRAQGE

>Lp_000012200.1 acyltransferase-like protein, copy 2

MNATATRVSSQVAAQFSHLSRSQVRGSILALALGVGYTFLRFRVLPVWVMRKWFLFSATV

LIIPSSALLYLIDPLRYLGVPRRVVQLICLYIMAYAFKAVWWVNPQIRMSVKFDANVDGK

PTCWADVPNTRMALTMNHTSFWDVFEMTGLAPMLHMRNMRTMMKATLRDIPIFGGIFDRV

GHFPVYFKSDEDGNFHVDKEKQAAVAQLVDAHVTNGGNLVVFPEGAVNKHPDTLLTFRYG

TCSTIVEHRMKVYYMVSIGNEKTWPAQLSYGGFPADIRVRIGAFPIDYDKEDSKSVAQRL

QQRMQQVRDELVAEVTAEAAAAAEPSKKKAVTVDPAPVLERHAKKPAAA*

>Lp_000012300.1 acyltransferase-like protein, copy 2

MTCLKAVAAISFAVWYVLLRQRWVPVPLMRVWFVFVVLWIIFPISAMTKVVVQLRRLGVP

TYYTQRLCIVPLVYAFRAVWWVNPQIRMSVKFDANVDGKPRGWHDIGLHNQAFIGNHTSF

WDVFAFLCLAPMDHLIHTRTMMKASLRDIPIFGGIFDRVGNFPVYFKSDEEGHFEVDKEK

QAAVQVDVDAHIRSGGNLAIFPEGAINKNPRVLQTFRYGTFATIFEHRMTVYYLVHLGAE

KAWPRWTMMGGFPADMHIRGGRFPIDFDKEDSKQVAHRMQQRMQQVYNELLTEWVGPEAV

EAVVDAESVDKKKN*

>Lp_000012400.1 COPI associated protein, putative

MQAVRSAASHVPGSRNCWQRNWPRVFLPFSLATTVLCFVAIIMGFIKAILKPHEYILDIY

CLIFALLGLSAEMRQFAWARRIVYFWMRYFYFLTFYKARAAFYIMFGCLLLNNSVLDIIA

AVVTLVLGIMMLLVSLIVNLPVYEDPREQQEREEEYRSYYSGPTANHVGATAINVAATTG

NANTSKAAQNVEMAGFGPNNNNNVNDAQEDTPA

>Lp_000012500.1 BT1 family, putative

MAGFSNLGQTTAASLGAIIMEYGWPVFSRKNRCDYQNLPWLLFVCNIMTPLLVIPLTFML

IPRAKISDNIDIDGNVVRQKVDEAMKKAEADSQEEQRELYSNRNASPVKK*

>Lp_000012600.1 Adenylate and Guanylate cyclase catalytic domain containing protein, putative

MKFEEPFLEACESFLAPVGAWNIQVRSILQELWSTYVTGYLTARPPEEKAKGKGATAPDG

KDPFCLMFTDIEASTRLWAADAQSMSTAVTRHHKLIRGLIEDFEGYEVKTVGDSFLIATR

TVTQALLIAFGIQLGLMMEASGPNFHMVENPQGTGDASCWRDDALRVRVGIHYCTDASAV

YDSVHSRFDYYGPSVNCAARVEAAACGGQTLLSSDAYKRLQAEEAFSKLPVSSLLVEELG

KLVQAQEARGIDAAEAAEVVAKLENEQHQKAEEASMHDTSPAAQVSGRHSSAGGVATTLM

SLTTVKDEGLHPLKGIAAPVQLYSLLPPGLTGRDFANLRIYASSELTELGTMQSSTTSAG

LPTFSTTESVH*

>Lp_000012700.1 Globin/Adenylate and Guanylate cyclase catalytic domain containing protein, putative

MSTAGNASSTHSGAHRLPPITTTTRLDNLVGANAASTRQPAAPTSLRQPPLNTSGPTSSH

NSSSSGSFGAPIRRSERQPAAPHPPPTQTSEGRPKPKQSAKVNSSRRQNPLNAPTTYYYS

LQYAIPKGQQQREQQRCTAKQPVQDSVLSLSSNGTSNHSSLLQPGARDSQADALVGAAAV

PSPTNIEVHSGESDASVEEQRHAFLAERQGSTSNHVSPLTPASAVTANASTDPCTAQMAE

EMHELLHLTRDMHSFFLSSQQQQQQQQAVGMVNAPLRPAAPAVARHGEVKLRCGAARLNG

AAPTAQKEGGESGSAKDATAVVPAERRKSHLHLEEEGAEKRDDNSSRKQSADSRYKEALR

SADAAINDSHVALQRVEDYSASLYTLVETWMQLYENRFDKFARTVVQRMLDKNPSYRVLF

FDMDVPAQSLVIMNMVGRAIISFAPPMDLMEIMNEIGARHNLYGVHMSHFESMRDAFLEV

YEEFVEPEVFSATIYVWRTFWDAMIRLAVSGSNSERGKIYTQRRNVVWAERLKKIFNRLV

PLQRKGGFHQLMQAMYPKALARFPQAKNFVRMTEKRTAHRMMEVIIRIVKSIVENGVPDK

IEYNEQLSVDNLYTDKTQQYDERIIMKFEEPFLEACESFLAPVGAWNIQVRSILQELWST

YVTGYLTARPPEEKAKGKGATAPDGKDPFCLMFTDIEASTRLWAADAQSMSTAVTRHHKL

IRGLIEDFEGYEVKTVGDSFLIATRTVTQALLIAFGIQLGLMMEASGPNFHMVENPQGTG

DASCWRDDALRVRVGIHYCTDATAVYDSVHSRFDYYGPSVNCAARVEAAACGGQTLLSSD

AYKRLQAEEAFSKLPVSSLLVEELGKLVQAQEARGVSITDPQLAALSVTVQQAELGMEST

QSNARRSVGALTTMAALTHVED

>Lp_000012800.1 Nup53/35/40-type RNA recognition motif containing protein, putative

MWKQTVGDWQERRHRRRAPAPATPQANLPESVASTEHSARGFLPRGFHDRAPPSTYPASD

GAQAHPSAQPQPHQFIARALTDTWVTVTGASSDDVLDVRDFLDTNVGPTVSHYLPPLAHV

RSDAVYIQFASPLQAAQAVRTATYTTQLESGVAAVGVKTTIDAQAAPARSPCYYLEIVVS

WCTDQIFLAEREKRRRQLLESAAPSPQRLTSVHMSEARGSADRESSLSLESSARGSELLA

SAAGTVAAAPGSGSGNGDGGDTAAHPLLPRRRAGQAGEARLSPSPWPSSPSPAGSSASPA

SSTPTPTPTPVSANATAASYPPVRDPAAYYRAGGAYRYEDSFFSDGLHSISRDNRQRSTV

LGLFFHPCSSSFGILFSLCLYNPLRVVLLVLWTLSNAVVQLLSASSASAKRAPLSRWKQR

RTLRASGVVPTSASPAEYVAFFFYKYVPFTPDPEEVDVVLWSWLALRSPYPQRSLHLLKQ

SLLVRQRNAGVAAGQLRSGRDADGIGNTDVHYYAQFGSVDVLGGEPYNSWERQQRVERRC

ELPILLQWRPAWGFLRYSSISLLVLTVLLGCNVWNLWL*

>Lp_000012900.1 hypothetical protein, conserved

MLPYGTLWKLDQMLHPVGPPQRRTVAMRRRQALFFFVWGVLLVTVFLLPYHCSSLRRLRL

EGARRVRGDSAPEMDVAQLKQRQKSALVRLTKQLTARVNNNVRTAADHDHDVGNAAARTA

ADFATVFYYHPSTEVRLSGMFDHPELRKLAGAGGGRLWRRLGGGTPSTAQRWTVAEATSA

GNGTGSRICVVALHRHYLAARLSNAVHGDGDAELIDTLRVAVHSVNRLSPVTVSTDNAAV

AGANVGVPSMESTLARFFLWLAGDAGLGDEAVSRAVAGCAAPLFRSLRASFDWRASPAGK

PARTELEANLLEVEERQPQYVCLCSGRKPASAVALRDTDGSSASPLFLLHPQQFTGEKPS

NCAEACVA*

>Lp_000013000.1 hypothetical protein, conserved

MVDSIVAGSFSPTARVTTLNMTTPSTDLLLRQQQQQQRRRLRGQGSGGVVPALRPYVHGV

RLVKEVSKAHLGRRPNAEAAVLLQEAVESTLATPPSWSSASSFCHPTPRVNDAQETAEKA

YVAKVNAFDLLQRYLTACHDTPSLHTQLAVTKAVAKRTDSALSAANAASAAAQALTARVY

EHCLQQVMLHQLLREAGLEDVSEDWSVTEQVPPETIVPALVDFRERFGAHNVAVTASALD

YCTHSTTPQPAAAPATVEKLFSDKLATIPTPGATVTRDAAARKVDEVHQTSAYAHNALNV

HPTRRVTQLWMVGVAAPPQTSKQRDDAAAGQVDSWEKKREGERQRQRQPTSMPQSCRARK

WVDMVPRAGSTTSMEVYAVWWSLLQAELKKKASSATLGRSLSSAADEGKCAPALTTRLVY

QLVKQYVQRHAEASTALLRTAENIPSAPSEAATVSTAPMRFTTHETLFPCGDAMLEVWSS

EAQRTCRYIKTNEVTAALHAGARPPDALSSPLRLAVRWDDGLLFSCSTTGAQQSSFVPLM

DVQVTTSNFVLQRGADDAHLLLRRFPTTSSCAAAAGIAAAREGSGLLAAGFPQGLLSDAC

ALLFEKRTTVYGVRDWPLLEDASATVAAAVLRAEVVVESSLEEAEVSCEVDERTGVVRRT

YPSGTQLLLLPSGTMLTRRPITQEDAGASAAKQQSSFFSSSLSSDQPATQQWCETLITCD

GRCFVRRVSTPMSGAAPSAAADMKAEPASPLSATTTTAAATGGVFRRVTPRAASGRPAPP

FTLAESTNDAVHHCRVWSRQDGLAVVEYTALADVPAGVAGSAAATPSTMARVVVFPDGTS

ITTLAHRNVRQRFLMSTARDAGDGGLAAAIALSSTSLSAMLEEVEAVERGLFSAGGAAEA

AASVRWLVEAPTLPRIYLAAANDGEASKAAFAAVFGDGTVLQRHWVPAATPLPQDTSQRP

PSSSSSVTSDAAAVEDDEHFHHSGGGGGGDRGSSAAALYSGGGVATVLIRPSTSAVRVLH

QHAMATLEPADVLAAVTRTSPAAYAVGLGLPFFDFAYGGGMRVVDAARCVWEVRGLAASA

GPKVVYPDHPRTYKELLRELVSAHYSPHRLPRAAEMTYASEQRRECVLYAEQRSTGWCPP

LVRRLRELSEPFIRAAKLLRTDVLAPLEEAAEATRVSSIRPVCFGQLANGESIRYWCAKD

VVPASSSASEVAATVSAVANEPHVLQHVVPSDAAGGVLRYPCNGPSHASAAEVMAVLAAD

TATAAGSDSSALGQRLAEAQRLSLLCVGGLAPAFPSAYPAASSQSLAMTYASTLPPLVML

VSHGCRWLPPALQPPQQFSSAYDKSVVHAVAAQNDVAWAHNATASSAAAATTAAAYAHYV

TERVDANAPPEALQAAVARRELRRCEELAQLSHYHRVLTAQWPMEQPDTAREEQVRLETR

YDELQRTRRPPPSEEKPSFLPSVRKDANAQGFKLLGSRLMS*

>Lp_000013100.1 hypothetical protein, conserved

MSARAGNSIRSSRSPAVNAARRDTIKASSSPSHAPANGSTLPASADPSSSNSITPRSQNG

SCVQRNEPLPAPSTLLRRPVSHNRNIPFHTGSIRTAPPPAVLPRPQREASQPPPPQQQQQ

PGGARPRASSYDVTQYRQPVQQQSHQHPSPRQTSAQTTANSSSQTLIIHNGGSVPSSAPP

SAPAAHPLPRERGVYPAPHGAARPSPIPHHVSRNDSDGYPTYSTNSSAVRDSKRQQEISR

GSPHQRPPPPPLSTAVHNQAMDIAPYTQELQQEQPHYSQPSQPRVQQRVGGVYLESMDTI

PKSTTRTPLPQQQQQPRQLGYVATDAPSTALTLRPSPPVAGQPRRRSKEDSCDCGTGTED

LEAYVHAVPPRRIQPPPQSPRQQQLEAPRPADYYSLAPSLRLGQHRHVRRIVAGEISREP

PQPFYDPLQQQQQQEWWEDEREAAPREDWTHAERVAPPPKRALPPPPPSEQQQQQRQASQ

QYSQQYPAQQQWVPPRQPSHTVQRPDLESSNSLISEVSSTHSSRTRQVFRRQQPQQRPFM

TGALPAGSPYGAKPTYAVVIPRTHRVPAGLYAPPPQSLTVQPPPQPSWEAYEAPVADTQP

TVEEPYYVLDEDNGRVVEYEPDAPPPQQRQLPLPAPSARARTARPKEGVPPPPQPQEYAE

RPVPLMRQIIHFYPSPQQQPQPQPQRQQQPPLSDAYNELTPSERTYNENSYSYTDDEAAA

QPPQPPPRRRPPPPPQPSSRKPKPKRRRNQPSALPPSPSAPSQQKHHQEHESGHHHRLVH

TLSGGSQQAPPPTLPPPRPLCSNDDFRPSQLDYHRRQNQHHRDRNRDDYDGREVSDRRYA

NIHPTRSDDHAAAAGRRPPAQEPSEQPSSATLAAPLQPGYRLDEHDLRLDYNLLSEQLTA

AREGEDNEDKQMQQRQHQQQSAHPPKRPQRRNPSPMISRERQLQEIKPRPRPKLSATVAG

TSDDAYTQAAAAHHTQPPQPAEQQKLEEGVGKSRDEVHEEEKVEQAKHQDATAVQQQHLH

STVGDLNGSESLDLDSSTIDPDDLLAQQEAKKLAAELVRRSLLPPTPCATSQLTKEQALY

NDDSEEEEEEEETAAVAKPMVGATEVVAPQPTQDGTAHTAEQQQQQQATEVAAEEESTVD

IAVSSSTTSSGKETQNGVATTHASPTSANSEVPHSIHAMQGPESMTVEGPLVRNPTLEGL

DLSAPERLTVEEEEEKKEHDVPVEQQQVEKLEAAEEEAAAFVSRQGQQQQSKQLEHPLER

EATTAQDDDDNHDNHDDSVRELRRASRDDATKQSSGVNRSPDHHPDHHEEQSPEANVKDA

ELDLPEAEARQKVKEEEKEAENETEDGAAAAAAAFPAQLATPMPRPLSDALEVAKKHPQQ

QQEQQPAQQPQPPEKTAVHQLVKREDELAEDENASRVVEQEDDVGASPLEIGERYGYVDP

LDEAAMDAAYMLDTLEDQYVRVNEETGEEMEDLVHVVELNRYDRAAEPRYFVPKGQYSDV

LPLLRYPVSVTNSGADGSTTATPSAADALTYVPLTETAYLNLCAVIWLSRFHSEDAARLL

SLAQGLSPAAPSQQVSTSEKDASLPSQVYYFDHPSAQLLEVLDHTIAGHVQSVVDEACAQ

SGESVATTCVRLADRNNLSYLPPAVWQQTTTGSQLSVGSIADHVTEVLLTATVPREAARR

QAARRRTTNEAERQAQKTWPLFLGALPWDYEEAVETGVFAAMPTAAARLSYAFMFKARMH

NLLGGLGALTFSAITAQVPDTAVMDSLLMQEKLRLALLHSHKNVAACCALRRSAMKSRNV

SSSPANSHVAADHDDKDSLLRDDVNNANNNNANAKWSSPVFSAYLQLWEKEKKVVATPTD

SLPRDQHSKAGTATSSPNAIGPGDGAEGNDDEMIDLYSVAVQLNCFFPTKTERGMHQLCA

ALIEDLISMGALVVPESVCTPEGAVLTDSTAIVDALVVEGAQAMRHIIMTLSELGYTRTI

STDDYVDAGGHVDAAGVREAMEGYLVLFDPGVMYFLSTPVPVNDLFQYIRSDGPPTMQPH

SLGRKSMFVEVLRLQHLAETIQYAMEMQDAILVASQYNHLVAQHYKRMKQQRQQRQTAQP

TPRTVAEELRMDAVVVGQSMDLLLNAAPLLSREAETNASSLAELSGANGGDSADGAAENK

NGGDARKRNGGPLHGDDDNNDDEAEDVILGQDGVEDANAEVVLLRAVLIALCCADPTKPL

QEVQEYLRHLLALYAEVRDEHNNPIDSSDPLQVTHARATAAEWATVDWFGQTAEGGTLQP

GAAPARCDEMRVNSVELAALCPRVLGRRVGLASTAVADAAAVISASAQALAVRDGARATD

GSNNEDSVLTADASSRPPLAPLTSPHATAVHSHVLPP*

>Lp_000013200.1 IQ calmodulin-binding motif containing protein, putative

MGNGASSDGRAESQRPISRPHSAAAGGGAPPKRQVLANQSRRRSANSFRSPAATQEAAAS

RQNAARSAEAKSAALQRVAARRCAAANPTEENAIRAFTRNADPTSLRVLQLIQQADNVPS

SNPAEKLRLLRQCYPLLEEVPKERFDQLAVTVYQQEGDIYYQQMDIQNAKDTFSRAITLA

EKRVARQDTDMYMVLKRYVLAMIGMARIWYDHERDHQGFMFVDHKHPKVPAVDGTSTDDG

SSSLSSLESSLSSDAGSVFSLNEAILRSMAPRPPRRQTGPLFKRLSVKAPHTLKNAQYNR

EDEFVLHSRMTRELVASPCELLLLRCCEVVQIGHNAQSELLIPPQIELAQIYEDLELYSR

ALLLVRRCLGILCSVYDYDHPWVIQLMQRSDRLKKLQDAQLRNAAATKIQATWRMHKAMQ

ALADALGHPVKRHQWIPRKYRTTPDLDYLGDVPEGQPLGEDSAAPTEEAAADPRTRAVVP

QTQHMPEVSLGNDDLRPDSSYEDAEQQPPEALVRYNPTEHREGGDTFTAMVPNANVIGTT

QDTQTDTDVQHTEFGDVLTVRTTTVTKTITEDLAPSDDEEEEEEEPGEDNGALVAESPHK

ATPPQQQQQQRQQPQRCPPPPHNSSLYSDEEEDYDDYEDEEQPASSPFDTSTEAQQRRPS

RRPM

>Lp_000013300.1 sulfate transporter-like protein

MRLAFQGFDVRRTRRSVEDLSEFFALVEKMLRESATVSSPTAGSMSASSSSPDDASLDTS

VNLSRLHFSSPVAENWARVLAIVRSRPEGLKVQIKARFSSGSGAGSFVGSGTSENNGVPV

HPMQRPSGASGGTGGQWRRLSLGTRSPMGYSSASNVDDDDALEPVAGLLVVKWGGVLTRS

GISQAHVMGERLFARFYAAGDLPLRRLVQFTRHPLVTASDETRVVNTALVVANALTQSSG

IAVHRTDVMLNDKLTKTLGPIAKRLLREEYKYFESLLHIESAAAARHFFHIPGFRQLLWI

PPLDRPLAVASSSDDDGVFDNGANGGKDDVHSESSSNNSSADHTRINNGGFAGDGGGAQR

SRSAANPRSFGFHGPQDISFTHSSRTFGMTADQQQHPNYNVLRQDEFYTPYQVLQELEEL

MTTMSTIKFPSALAKIPLSNHETMHEVQLRYDAMLTNYLGAKRGILNGTSSSGPRGRSER

NADARQRDRNLNTKTPISPMCNLSDIGPDETKENQMMLVEIDESAGSSDDEGVDNQILED

AALSREPSEQASPNSATRTSYLIAPDSAASGGNGADRAARGTHAPPTRRARSTGADGSAA

SATGAAARSSYSMTETATGPLWAPPGFNASRGNNAAAGSSGGHSNSGASSSKRYDVSDVS

KLRDYGSYDIAYNMPLLLQLHNASQDGSTAFLIRKFARALQRFADITEYMSRIGENVLEG

VNSSKRFIIGSLVSEGLLKRLHDDFRELATTDEERGVQELLMKAQAQYMTEKGGVEQDVR

HMIRLFSSLPCRPKRSDDLVGTLPADVWGSETPQEREFRLRFPFISIPKAEPRTQMAATD

FNGCTRLYFTSYLHVEGVLQCLFESATVEGFSRPSKEEMEATHQLYMRHLIFKIFRRRNV

SFTESEVTLAFRNLNLLFRSDVEYDKQQRDLRRGLAAIYHSMYYVSMEVYLSLGDADEGQ

PGETDEFPLFTLNPDNDSGELWVRTDRNGSGVASDDGSAAPSATGTAEGREEQEMTAVPP

SLGAAAVDAAEQTSNNFQLPPSTAPVTTAATDTAGADSDANNESYYMLARSPLYVPRPPT

STVEARAAATASPPFSPAVAPNASLVPLLSPAAMATTTNTATGAAADDNDESDGDSDVAA

LNRTLRRAANLSVSTTGPPAATKAKKRSRTPAPVTNVKEPVKEATVLVTPEAGRRTAVVQ

EMRHRVLNVTPMRRVHEGLSLNDFVLLMKEVDSAVRSQTATPSH*

>Lp_000013400.1 differentiation inhibitory kinase, putative

MFYRFFGKSHNSKASFDANSSDAAPSTAAATAAAASGSATPATGGGNSAPAAEAHGASHN

GDGGSAVSGGARGFDRRAFQGEERLLEYPRGPRRYPLDPDDDAELLVLLAQQYSITSVVI

THYRVKENYVEYVIECVRGYDAWRVYRRYQQFKTLDHDLRQLCVGRHGSNHGAYGVVPVL

PGSHWMDVTNQSPELVEQRRRYLEIYLQQLLVPKNLFYVARTALFDFLHDGEVPTNLKST

GIQPLLGLISTRTNQIDCDNSDEAIQREQQQHESQRRPAQQSSDANSHNHNRNGNNGNRE

GDSAAQSSGVFPGTPAAVEADASYKAGTPTCTTHAGLGRSPSPNEAAPLPQKTATSPSYT

AVGSHESPSSQGAVTFSAASSTHHASGEQASRDNHAGESHAARSENYEGKRRPQKSNART

LAEEAALAATASLQMSFTDERLPPASANCAQCNAEFTSFLYPHRCFFCRVQFCTTCLQKV

ALLESGSAPASAASAAPEALALRELRRSPLQIANASREAGSVVARSVLACRQCAENYSRR

LDRCIAAPGGGAYRGMQSTLAPPTPGTTSNSGSGGSGLSTLGGGGGGACGGNGSLQASPG

ASSSPSLSPAACVGGERSSMMLQKRNTGPGSPQLRGSPSGSFAGRSGGFSAVGFHDFQLL

TVIGRGTFGKVLKVQHRSSGNVYAMKIMNKATVYKRCMTSYMKEEKSILTSMKPHPYIVR

CYFAFQTDYYLVFVMDYLPGGELYDYLYPKLRLSPEATCTYAAELVLALEHLHRQDVVHR

DLKPENVVLTADGHICLTDFGLARRAFSRSRRRSFVGSPEYVAPETIQGQVQTAAVDWWS

FGIMLYEMLAGRTPFHARNNNTVYDNVLHKELKLPLLKTGGDVMSPLSPASPKSASVADD

ASGFKGFTSEAASLLSGLLARDASMRLQDATVIKKHPYFRDIDWEALRRKNVQAPCIPGD

LRDNDVRHFKREFVSEWASVPPLTSMTRASIEALTRCFENFPVSRGSDSASSSSSASSAL

SPTTGSPQTLKSGASGSHPEMRALSPFLTLLPPSVLSTDGRTLREPVHFAQSLEAAQGCF

HGVWRVVSVEVHALEDSRIIFPWGGDVAGVLIYSPGAHFSLQLSPSARRPMGPVQRVGQL

SKEDLCDAYCSYIASFGRFQVFPSSIDDGCGVVRHYAEGNLCPNLMLTNTVFQYRMEAEP

VSKSEERQRRKETQPDPNSTVDSLKGRSGVAESLPKPLLGTSTCDSESSVDGRASNSVAN

TTTTFVEAVEGCGTTTSPPPATPPKRRFVLRLSTRPQRAFEEDFMAFTSLVFEKME*

>Lp_000013600.1 hypothetical protein, conserved

MIRKKVLSIGNNSATLLVQDTEANGALRVLRRVSVAGWSNEDVKMVEESYEMLRKARLRG

FVPIHTILVQNSFLSVVSSYAPEGDVSTFLEEEVDSPLDEQMVLRWLCSCALSIREMQTH

KQFFPGLTTDRLFMDHQTGGTAHILLGVPLPLPVYIAQMQERRRNGVKVSLDYPPEVLAE

PNWTFHETATDVWCLGRLGELLLTAKGTGLARRSGSTRQLLSRMMTPEPSKRPSMESVAQ

SLIALAGNVKLGQPVWPAETSNTTFSAILNSSPPPSHPPLSAQASHHHSHLSASNNSLTS

HPTSPATTSSPPEVPATRLHESRSKTETAGPVAVLASQSPAVPQHREPVSRPAYAPDDSW

HRRAQHQFEQLQRLNASPPKLRGAGDGVRSPRGANVERRRSSAEAKSPSPRRGGRSGGVV

DHNTRMLNEMFAEQEGLLRDTNSDRPPQRRTSQHAEDLQLLQAEAARQLRLETAQRQQEM

RKHFTEWQRQNNERYTGADNALVMEQDGVVIVAPRPGPLPGQPPSHATATTTTTPASSAV

ASAQPSEKAGDGRSTPLTTSDDGPNEAGLAPSPTALTTVSRNAATRGSKPRPKTPQAASP

VRRITSRPAVPSSSSRAGAPAAANAAAPGGLSAAAVPRHSNGSPRSPHGLQLSSVPPHVR

RSNESNTRPTSSPTEDVSSGPTRRSSADPATLSAVEWSVDGIRSALRTLLRNRDLYGDVM

QEVAVFVSQQEEARLSARANEIFMQRLRKLLPEDRLFYGAVPLCAQLVALEGPEQISGKW

GRAHPSR*

>Lp_000013700.1 hypothetical protein, conserved

MGGAPSRETLVRHIVRDPRLERLTGGGASSAVGDVSVSQQPEIILIPLIPDYFPSSVSPL

FQQLMQVRREATVSYYYRDARSTVAEHTVVRDHAGSDKELYNWYMSLSEQRLMETDNELI

ALSRAGRVVSPVPCAVAFVYNANAAPATSSSSSAVVVATAAGPDTSAAATTATASDGAAS

LTNSGSNTKRKDTSRNKQHRRGKASTGGFNNGYMTIENTVRVPHHNMRVRVFGFVGDTSF

CPPAMLPSQAAKSPMCLTIFLSKSAGGNHRVQVTLLAAHTYMQQLQEIGILRYVDRGVGG

PSAADTVAAAAANGAGCSKASSPLPGSLAAESTDDMDPLAGLRCAGVGSRAHFPNSDPYR

DPYADPYSVDENADPYAVAGESEHEPQLQQPPAACHVPRGGSNTLLDGLGGALLSPAMEV

AKETILRRPLEMVMHRADVPALCYVRELRARMDEDTAAATITAAASSTTRSADHLHDDEG

EEGATNVDSSSGGRGGKNNGSQKRSRMQHNSSQAEARSGVVSHNLYEKEDDDDSEQHSLK

RLIHTVIREEELHGLIDAESMGRVLLWASCFYVTHFEQLVGGEAARLRQELAQRNLSENP

LDGTLSLTEDEPRPPKTLPATGDVWIPPHDTYVEVRETLSVPGGASNENSANHLLHHEME

NWKTVVAGTVLRYYAAQIDTASTATASGGACRGAWRVSKREFAEDILLSLCRNACHASRG

APPTDEHWLLNRETGLPEGLMAGLLIWRELAAGQVVYYGTTPDFLKQWLQSGAEVEAAYR

HQAKKAHVVGAGAADATKDGDENSKSVTSSLGAAAGENGAAPAATCHSPSITVATTSAAA

ATTAASKGLRSSYDANLLANLSGVSSGSRGSSIKEQDKGSGSSSMALYSAHTGFVCAPEQ

NAKTPLGPKCDASSPTSNGPASTAERSSNGSGLSAEARKTKARRVLTETSTPAAAVSGGA

CDVAKVSTPVAHSRAVLGSSAGSSVDGRLTHSLSAKQHSPFAAPVASSWSVVHTPSSAAS

SASRSSHMYSNLGYLDQLGSLSGGHANARAPHVSNPFAYPTSSSASNHQQQLSHHDHGMP

VNLANACWIRVPDPTSRDGANTPTVNVVVKQRRSVRLTTGISLLDSPSGNYARALQEQQQ

QPWGDIATTSATQPISVGTAAGGSTAVMARGSLPIHILGSRGSSSEDSSFRHGCHSPTLQ

EQSRSLLHGDDALISHAAATRAEPISRTTPQHSAAMDVRPNYVGTPVESSTSLKWTTSNA

AQPSSMRGASVSFASGSARHGSLLQGLRYQELCQPVLSRPSSSSRSELLASSASSSVGSF

QRKEAMRPPPLFADGNVESRPADGKSVGASVPSQNTTDGTSLARTSAAGGQTKMPLISAV

PMAVAGGDAGAGVHSRNNSAAHTPRQENMSDSAKSSVGSGKYRWEWRGVSLSS*

>Lp_000013800.1 hypothetical protein

MATKLQASLMTSSTASVADEAAAITAGYAQGRTTVYYTDCKSLPSVHSSLAATSAVDRAS

SPLQEGCSQQLTQLGSFSFLPHPEYAGRSGKTVNKLLFLHHLIS*

>Lp_000013900.1 Domain of unknown function (DUF3342), putative

MSSSSTTAQSSSHGMVVEIAVQDPETTQERVFTCSLAPLRSHMRYFEPIIAKQMNEVKVA

AVASPSGTTPPFTLRAKCDHHTFQWLLDWMNGNAPAMSLHNVVSITLSSSFLQMRALAEE

SLLYLRTHLPEIVSSGIDLRALPVELVLRLSRIVRDSDLAAVLQHLYDRKNTTHPNRIFV

ASLLQHYVCYRLGVEGEDEGSDDDSSPSAGKKNAAGRRSEVGAAHSSNNSGNDKRRSAVG

SGSGVGSLTLKSSSGLRWCRLCACLFDQAEVQRLIRAFQLSSPECPAQAAMAGESSTAAA

PDASPALSSSAGATAMANRSRSGSAPNTLSGPATVRYVGPRGEVFTTHAASRRALPVVLE

APPLLKYDSAATAASITASAMRLERWAWRIIGATRYVSCRRCFHLVPLVEVPSHHCSSLP

PKFASPDNVAEDVNHLVRWFIYCTERRVYADEGGLTHVHFSGPRHVLAEEVVEVHVAPKS

EPAMPTPQAAQGIASNDAAAEKDKKGASTPSLSLWAAKPFYVAEVMDKGIVDIDIQNYVE

RQHRFEVEAQQRRASVLFNPPHMRLASSPAVGQASFSSSPSPRFVSSVSGTSNAGSLNRG

KPGVKPMRVRASTRSPYGF*

>Lp_000014000.1 tRNA pseudouridine synthase D (TruD), putative

MQLQRIDTLTYLLGRYPLSDAAAAPTTHIAGLHTQQNLRDAKPAPGPSLASDATATSAMG

KETASAAATPVAVATESEAPRSTCARARKTTLRAKKNRLAALYAKALPAAHKTGAAPAAN

DFSSTLSAATAGTAPCSAPSVSPPSPALHKPRKPPSRLWAQKKKVDSDFSATPAVEHGPF

TPLEVVEQLALQWWRKDRRVQAEKTKRDAAEEAAWQALLRHPSHLFADDDAVYARFGMGA

YVSPNLPGFTGLFRQQWQDFHVTEMVVTSQDSAVSLRSTSPVSAERSLFCDATPVSRDFD

FSIPPLPSELLAGADEASAEGAVAVDRDGDDGGSGGPDPSFFAVDVKQRIQQLRTGDVHE

RDFAAVSEALSKEKRRLAPSDEGFASEPGTTTHEAKKDSSSSCEASVDSINSSKNGTSGE

ASHFDEAAFHKGSHFLQCTLHKQHVSHGNALAAIAQTLRIHPRSISVAGIKDYIGDTVQR

VRLENVSPASALAANRRFRHKRLRITLSDFSYESAPLSPGDLFGNHFRVVLRDVDVPKSA

LADAVAEFVKKGFPNYYGCQRFSWFAGRNDAAFALLRHNWLAFAFLFLNFTSKDCSLREL

LQRPKKYPHPTQDEYRRGVVRRLRQIAISPADLDVAPFLSCPRLNAPLTHADGRPFNEVE

ELICAQLREAYFDLNVQSRRLTAQRLSSYLWNQVLTLRLHHLGGEAVLDGDLVAPASIRQ

LSMDVDDRQDWYHTFGDRVTAENRHQYTIEDVVHPGFSFDAIALPDNVVGAYYEQVCGKY

ALDWTAQHSRTGLRDFREPPRPIIRKPLNLDYEYNAEARVLTLRFALERGCYANVALSEL

MKSVRCLGTEEVTVLPLPEALWDTLGDADPGYVTTLQDIYAGYEDGVGFMSDEAPVESAA

VSETKPWDFDGPLFLPAAEDPFRKAHRWGSQHLLRNSERREKEAEDMKRLLFDRQLAKQL

KEGEVDTYAGHIVPLPPNASAKQVYAKVMRRKRRYAGAPRMVTRMQRSTAPASSYRRHHG

AKKLPSFQSLNKNSWNFTW*

>Lp_000014100.1 hypothetical protein, conserved

MYRPSFSEEAAPTWGAASSPSPAAAVGRHRTPCDDAPAYDPLLWKDTDNSYISTLATHVM

THQPPTTPGFGPVSGGKPAKPPLQAFLSSSWAQSPEEEAAARLSKFHAVYDRKPTTWLGR

VLQFVSAKLFPRWHEQLRPADVEALPTRDLIHLMQLALAAGEIDRSAMLARELSRRKLAL

HMQAFPARDPNIGSGNGGGGGRETGVRVERAAAPPSPASPLPQPASAADYDGFLPRRESP

QMQPQRPVRSWADSLGVPSGRAPSWSPPRRELSAHSTRDYSAAGMPISVERTAPWGGEAV

HQSSQPSAMAPEASAATSSYTTDVGRRSLRGSEKSSRWPSGARCCGQWPLSPL*

>Lp_000014200.1 helicase-like protein

MASLLEDILLRHQQWEDEVEGKRSALQEPNSANVPIQGGTTLCQTRTVAAASASVAAKDT

SASHGTELAVKETCYRSRPRGLLSSTVNVNLRHDNIPVTSCSSSTTFTSSAQNTVPKASS

VNSSASATAAAATLALPFDPYPIQQEMCDTIQSVLTSRKPHVVPVAVAEVPTGCGKTMAL

LSSVLRYQQELKRMSAKEVDAYLDVRRPPWQRTPAAKSKHKAGNNKEPTKSSSDEDNNGD

NDFESSVDARWTVPRSFFKHFRVDSKRKIRAELDVTGSQELRRRFLPPPCTIYYVTRTHA

QLRQAVRELRRLHGAASAIRMNILGSRERYCIHPKVVQAKANRTLPVEGNNLGEVCDKLV

SVGLCEMVDKYDELSCSAIGGAIGHQRGLIWDMEDLVVEGSARNMCPYYAARDLVSFADI

NFCTYPYLLDPLIRHETKMEAALKNSAVVVFDEAHNVSAVCQDALSLECPRSVLDLIVSE

LEPLVANRHTLARSAAATPATTTVATTTTAAGTTGADSSNGFATMQYPRELHLGPFTLVE

IFSFLLTIFRSLGSFFEQAAVPSTAATTATSQHRRFSEKRPRDAGSDGGDNDETYTQGVE

LEHHLRCTMEAFTRTLHQNTGGGRAGARGETAALASLQLFRRAYGVIMSLGVTFNPFLFT

VFGLSMLKRWLLLLRFLLQRPNSFVLTARDASSADDGGGEELHASVDGDATAATRGGKAN

RGVVEIRCLDGSLAFNHLLRSVHRVVLASGTLAPFTQLAQDLGLTPSQWRTVEGLHVVPV

TQYRLTALTTLPSFTSSSSSSAPNMPLRCTYASLSSPSFLKAVARCVVHLAQTVQEQRGG

GRGGGVLLFVPNYAVLHRLAKNTRELLLRLPGAQRAGSASDASSIPLYLEPPKAEALTQV

LRSFQCHTQSPRGGTALFFAVYRGKVSEGVDFTDDMARLVMCLGVPMQPLKSWKVVAQRA

YSGPEWYTTDAVRAVNQALGRCLRHVRDYGAVVLLDERYAQADYQQRLSRWCRAALQTDA

SLSHLCEALQASFAQWRRELAPPAASSILRAAAVEDDGGAAGASVNGACFAALKRGFEVD

SQAQESDSARGRLGTDQLPFRFVRRVPARHRHTSAEELASPVLGDVQPGGKVSRCGAPER

AAEGEGTVAASARESLLQSPFACTAVKLLYETAHSTRDVSRDDLHAAIALLTKTFKEEED

DDDEASNQDWH*

>Lp_000014400.1 phosphonopyruvate decarboxylase-like protein

MLRSSCVLLGPNKKLSPSFVVDSLRKRGTIAFFGCPDYHLRHLTSYLADHSNAGDYVTAT

SSGNAMAMAAGHYLSSLRTPCVFFPNSGLGDTMNPVLTLFSQDAYRLPCLMLVSWRGKPE

TKDEEAMPGLAAQGRMTEHCLTAVDIPYSIIGDSLDAEMNWDVIVDKAYFHMGTEKTPFA

ILVEPDALEAYAQHRPDADVLPPAPLDMATTIDQVCRQFNATDAFVCSCGSVQTALRRAR

GHAAGGPSAAATQDFLLADSVGHACGVATGIAVSRPGQQVVCLEGDGAALMRLSGMATVG

GLSALKDVKSGTGLLRNFKHIVLNDGVYSSEGGQSTAAFDISLTGVAKACGYFTMRDEPV

VELGDLVAALAELRQCDGPAFLEVVVSKTPTAAPSATEMSRDLQLEKQSFADFMTRTKPQ

SGKK*

>Lp_000014600.1 hypothetical protein

MQVENSRGYWQRVKADNNPSTSGSFYRSFLSVRTDNAVLDAFVAAANSGDVSGVVLVDDW

ASKNTKAGKAWIAGLVIGVVASVVAEVLVVLQIVFKQHSRHRGSVYSNDGLYADLEMSQR

HIDATAPTPPLQMRERHTR*

>Lp_000014700.1 hypothetical protein

MHSTSQSISNTTLRTRVSRLRMLLVVVYCLALLCGNSVAVARTLAAPKLTLYHLETRQVP

GADAVIRYVQHDVSVQLLADSDASQAASQAESQGGSQTASNPAPAAASSSASKVSSAAPA

GPAASSSSNVHSGEASRASSIAAAASSADHLSSVASSAETTAKPTSSADVSSSSSAESAE

AAHSL*

>Lp_000014800.1 hypothetical protein, conserved

MDLPLHLRLAYAACFSDPYADARCLHGASAALHESASLAAPKESSWFSDLVQLIEQNRQQ

TLAPHAPENHDVSHVELSHRSVPNLPVSEEEVKVEVEGTLDIPRRLTSIEKSPSPLRNSS

STQSLELPVITAQEIASPHPNVSAAMSMPQPSGATSGVGSQASAPDQKVNVVTQTDVAAD

TAPHAAQQQQKASCLASDPVANTGEAILADLTTSLERRLQDCVLKTLALDDANQQLICAF

QLPPKNTATSSDQGGLPLGTKTAAAVQASQQTLINSGALSEAPPSPSAAAAAPLSIAAVA

ANRSDASLSEEARKELAMHLYALRHQVVAMRQQLDTHEAAHARELNRFKSSSSPSSGQRP

PIRVEVVRAYEEEPTPYRKGPWRQADFAAAGATRRSSDNRKDPGKAEGGEDDYSSNDFTD

DTSEISTEERSSDESNMQSAPKEQQRQLISAPRKDHATVSSVDSDSSVSSTEELFQRRQA

AALQARKQRTAAAAARQQKRSSSSSSSSPSSSPSSSSDRSTTSSLTDTSTISTSDTHTS*

>Lp_000014900.1 hypothetical protein, conserved

MSLSALRAPRRTFFDSVFGRRAVKVKLDDVAYDKTTTRYSGTIFGLSADNVIFYAKVAAG

ALATLVTVYIFFKGYILLSRFSLQTVARLGFMGGFTTCLICYTTALALIRRYRINANTVY

NQSIALVMHNEKVVQHLGSHPRTGEFRAYNAAGGFKLPLLRRIRSGSYELADLLGLKQRQ

LQMLFTLQNPSSGNEGLVSCDVRKESTGFMSSTNVYKSLSITLYSGNKKTEPETIILIGK

PEDVVYRSLLLR*

>Lp_000015000.1 hypothetical protein, conserved

MLLAPSKLSSSPLLQDVPRTDKVVMASPLSGSMAGAGTPAVAERAGRRFSSGGAFDSCRN

SDCSLDDDNVELDDLTSVFLHGDSECNGVSLSGSTTVLGSNHYDSGGRATSYNTSSDGAQ

SPTPKRKRPHFFKQLANEFRWMHNNFKHGYQSGTTPKHQQQDSFGFLPTPQTTRENFDDD

EELLTMGVCSSLRRSSCSPNAPTSASETGKNVHGGSQKKKKMMRWRTMENGEVEAVPEPP

MPQTKPDPYAPPECRYVVRHPMASSANGCYPAMESEKEEVVLPDACLTESLTSYSDTDDN

DHYRGDTSLPERAGGVNGESSGHYSSRASSDVGAVATRGTCRSDHRLAKVRRASDGILED

MDEDVAHHTRLFATCDDNYDAFLRDVAQLQWQVHAMEYNISELRKLYSREQSAQLHSKGK

GQHLSWTRTVQAANTIHFWRMKVNLLRSFESQYVSTVQHLQQNLHLVDPEKESNAAVTKS

GKTDGAPSPVTMQSRYGFLRSSRVITAQQQHLRNLGEQLKSCWQASANLREEIQRFELRE

ARSPYDTFAVAGMDAEHFIASLPPRISAYGPATTSARSSVLSLDSMDDAQAPLLLLTAQN

LSPTSEDDDNPFFPASPTPTHDEAGGGKRRGSSNLARATGDAAPLASCMKNTSSSTIGAH

STPSPSPPSETALTPPTSSPSSPKRLKYVIQSAAFDAQDGAATAADAFNNNSVNGRQSNR

GGFTSRGMTPLTGALAASAEVQQQLQRRKRERHITFALEPEVLDTRPRFSTHSRTIQLLE

QICMDKQAPWNGLTLLQAALEEREGELAAMLDRVEDEQREVLARAVTMTPTCASAPVLRY

ESERREPHSRPETAPSPTLKPVPASLPFEKTQNRRKPRKVKPKDCAQCRVM*

>Lp_000015100.1 ATP-dependent RNA helicase FAL1, putative

MATEQVDDIQANVLAIPTFEAMGLKEDLLKGMYSFGYKQPTAIQKRFIMPFLNGRDVIAQ

ASSGTGKTSAFCVCLLQACNPQIRETQALILSPTRELAVQTQDLCNNIGHNMGLTAYACI

GGKSTEEDIRRLESGVHIVSGTPGRVFDMIRRKSLRVNGLKTLVLDEADEMLGKGFKAQI

HDIYRMVPPLQVILVSATLPADVLEMTEKFMTEPASILVKRDEITVDGIKQYFVSVDEEK

NKFDVLLELYDSLTIAHAVVFCNTRKKVEQLAKKMTREKFTVSAMHGDMPQAERDEIMRQ

FRDGHSRVLITTDLWARGIDVEQVSLVLNYDLPLSREQYIHRIGRTGRMGRTGLAITFVR

RDELRLLRDIEQFYATQIEELPANIGEQM*

>Lp_000015200.1 hypothetical protein, conserved

MTDNDVDDILQKFFVTIAEKDSDAFKLYEDNKRMRNLVEQTLHAREELESRLRSMSVLVN

SLQTALREKERLIEAQNGEAAAWREHIAELRTLLDRPSELDHGLEDVVKDVRDMSAELRS

LRQGFKYARWCCDEWEEYATEAAACAEARERAVLQEAAFAVLETLCSRASEVQDVRRDAA

ARLNSAAASVKQWETWYAEEEVKSQQQAETHERAMQRATLETQEQQTRGDALLRELEVVN

ASLGTQTRCHQQQQQRQWVEAESFSQRTRILEERCRCAEANWASTVTELRAVVQLLAEAQ

KARDDVTAKHELVLLTHDHERQRLRKLSKQVEQLKQQQSELEEVQQRCSAQQAELHTLRE

KYRTTADKERSLRQQLHSAAEAAAAKLSAAEDATAAHQRHRYVLEERLRTTQEELKALQR

ETVALRQQCEEHRATQAVLQATEAQLRETERRSSNFQDALERFTAEQQAQLQAAEARHTE

ELAALRCRHHEERDDAAARARAAVAEAETMLQHTREDSAKEKAQLQQELREWIQMVESLK

KEQTALRDTLEAERAARQLLEQQYQQETTVVRSMMLEQNREEAQRGPQLAGMQSQVSELT

QHNELLEEACRRSAGVIAQLREALHREQMTSRSLRHRDASFSS*

>Lp_000015300.1 hypothetical protein, conserved

MPLKANSLPTKDSADPMLQGTYVQDDAHDFEPSTDEIAAFEERLNHHGSGNSGVNTTAHA

ANPRRHSARVPEPARPANHVVPSVGHHPTSIATTSSAPSAAQRGLGRRRLSEAEQEEEIQ

HKEDVDDVRQLNARFGDADNDSD*

>Lp_000015400.1 Protein of unknown function (DUF423), putative

MVYAPLLFYAGFLGFTSLIAGSVAAHGLNDYTSKQRETFKLAAHYQMMHGMVTVAAVALS

EALRPTNVKAAKWIDIAAWLLAAGTSFFSFTIYATTLGAPSVLGPLTPFGGLLMMAGWCT

AMLSAFVL*

>Lp_000015500.1 Protein of unknown function (DUF423), putative

MVNGPLIYSGILGLTGIFTGAVGAHGLSSKTNEERDAFRTGSHYQLMHSMAALGALAFSQ

AVKASQPVAAKRLSIGACMFLVGTTLFSGTIYARTFGAPKAFAKLAPLGGYIMMGGWACV

VAAAFAL*

>Lp_000015600.1 Protein of unknown function (DUF423), putative

MMSLKSAHVPMILVGAIGTSSLVMDFAGSRLKSKLNMRQRRSWLTANQYHMSHTVALAAI

AWLMMLAKESPEASSRLQKGFYLVTAGALGFAGSIYSLCLRWCPKFMGPLTPTSGLVLVL

GWINVGLAGLYW*

>Lp_000015700.1 Protein of unknown function (DUF423), putative

MSANIRTALSGTNVPMLLAGLSGGLSVVLGALGSHAFLEMMDTNQFKAYSIANQYHIVHS

VAMLVVAAVAPRVTEPAATHFRRAYALFAGGTFILAGSRYVYSTLHKPAFLGQTPILGGA

LLAAGWVCVALGGSTMSD*

>Lp_000016000.1 hypothetical protein, conserved

MPIAPNFSLRSHHPRGLQSGTEDAFPPDHARGSPQPPSTRPGSNPSPRLAGSATRDGAAA

LRAPSSEALVVDRSDGRATSHSQTGAVPLFTAEEVEAASPGRRFMNIHRRARRERARSGT

VLGPSNATESRPYANNAESIEDYSIPLPVDTEASSATSPALTGASLRRGQYAAPGLINSA

DSGQHADVADTRVLLGSREVDQNPASAQLGASSSAIDSVGPLSGRGSGQGGSTGYSIPIN

VVPRADITILVAPPPVHLTTSHDALQRLTQGSVLAARSVPTLPRSAATSAQTTSSSAAGT

TSSSCRTPAAPVGSHSDGEVVGSHAAASAAVTCARAFSDDDSNALPTFPACEARRAVVWQ

LPILTITSRGHVARPSQHSSSAASLRSVSSPSRSAERSTSRVGRTESVDSQRRSVSQSVD

SRACLPRAASSRSSREVSWVQHITAAEDVVGRDAAGQEGHVGAALSNTSASTSTLATSAS

DTRAALGASQSPSACEVSAAHLHLQHRLTRDDGDSSHGCRGGKAPIAPQDSRSCGRRSSG

GGVSPHRSRPASRPVSLTQAPPALVPASAAANAPATPRTCARRPYAVASDTSSSAASPTA

VHLPTAKNGVVLLSHVRDGPAQGELYRLLRVFYREPLQQFPQALPGPPLAVRLPSDYDDD

DDDHDDRENGGNGDNPSSVSSHEHAEEPRRRSRSPYAMRHGSFSRRRSRSSSVSTRSSAS

QMMVEVNASNPRELFNRLLLDPADCAVEVRGLPSRPTSTLPTSSPRRSRRSRTSRSTHHG

SQPSTPVSYADRSFDRAALERQGTERHACSAFTTPARNQRPMMSLSGRSINAAGASACQA

EVVANGFFNAELRQEYAESYEAAAGVAEAEYGEGCYYTLPLIPLSSSPLHAAKKPRNTCE

AKSAGSVSKEATPGTGQYRPSGASGEMELLPCLSGARRPSSSVQRGCLASSASSGSGDVV

AWPTARSMHGADWSNSLASRENMAPDQLTPEPPKSSGVRSDMLNPRSTSLRSRESLSAAH

SGHSLAQASMDTPRQISCIAPDDLQVSQLNGNCAEAAVTVGAYQAMARVDLSACAPVTRS

DGRKAVDGG*

>Lp_000016200.1 hypothetical protein, conserved

MDKATFHRLWTALPFELVDKSFWVALEAFCDQFFDPHSPFNSQTFFSVQHTGRILQEELT

LVSPKTVEACNRLRPLYDTFTLWNPLALAALLKAPIEVVTAQLLLAVGRLWMTIHFVVNC

RSCGCDIAHFQSVNDISFVGTFHSTNAFRCPMCTDRTEVNELQDVAVYFQLQHLPTIFAR

RHHRLYFSEEADRRRLESFFCPAQAGFAFTVHLPEGRYLLSAPFCGAFVEFNVELPAEVM

GEKDPYLSRIVDLKTYIRSQNSSAEPFGQPIKRRS

>Lp_000016300.1 serine/threonine protein kinase, putative

MSATSDALIKKVCSAFPATFGVDTEDEKTVSDGRRSRPFRYYLDTVLGSGTSGTVLYARR

VRDDQPFAIKVMDVEGYTPQEIMRASGEVCCLLSCDYFSILKCHEDLAKRDPNNPENVHM

IALVLDYANAGDLRQEIRNRSKTNRPFAEHEAGLLFIQVLLAVHHVHSKRMIHRDIKSAN

ILLCSNGLVKLGDFGFSKMYANTVSEDVGHTFCGTPYYVAPEIWRRRPYSKKADMFSLGV

LLYELLTLKRPFDGANMHEVMHKTLAGHFEPLPNSVSKDMQTIVAALLQSEPRKRPSSKT

LLNTPTCKLYISVVQEIVQSGEAGGFSPDQVAIITRQLKQTKEELQVDRRRPQLSAEEVL

RSAVRVSLSDVPGRTGFILYGGKLSKQSSDLTWKRRYVCIYGEVEEDRPLTGDFASCGAS

LELVQAVSRDTLEQQCISTPFAELEDVFPVISKYTGSDAAHAFAVAFKNGRRILFDAKDD

NDRDEWMRAIQNFLGIGDEDD*

>Lp_000016400.1 Protein kinase domain/Protein tyrosine kinase, putative

MTAAEQSAALQRLCTSFPDVFCTDTTAERRRNVKYCVEKVLRFVDTTVWFRAYRLSDKEP

RIVKYINLTNEHPAIRAQVQSEVRCIVSCDSVFIAKCRESLVELDDSGREEIKTMALVLD

YCAELPLAYVLPAPVEGRHSKESSIIAVFLCALMATYHIHSKSIVHCGLSSHSFFFNPQK

DDFLQLGDLSSCQVATNPASNEFGSLFKSKPPYVAPEIWRFSPYTEKSDMYSLGVLLYEL

LTFKRPFSGDSMRDLVTQILAGTYEPLPPEISPEMASLVGSLLHRNPAMRPTAAAVLRNP

LFIFQLATRVSASALIKSSAPEAVLRRKAEEQYVYVSLINETIPRAPAGRVVSNAFRVGS

FAYNDDDSALPSVVATRPRPERVSCNSRLHGLVFMRVRQGGWEPFTLHLDRIQGDPTATA

ASSQLPPIDSANRRASASASSKQSFFLRFYPRLESSLAVERRIALTEVASVFPLPSLYSR

ERPGLVFAVMLRSAEALEFKVKKVNTCDKWLDILFKYIPLSRAES*

>Lp_000016500.1 serine/threonine protein kinase, putative

MSSGSGDAMISRVCRNFPDTFAKDEATAREGGKKYWISRVLGSGATGTVLCAKRVSDGEG

FAVKCVDMEGMSEADKNRAQAEVDCLLNCDYFSILKCHEDLAKRDPNNPENVHMIALVLD

YANAGDLRQEIKSRARSGRTFREHEAGLLFLQVLLAVHHVHSKRMIHRDIKSANILLCSN

GLAKLGDFGFSKHYAATVSEDVGHTFCGTPYYVAPEIWRRRPYSKKADMFSLGVLLYELL

TLKRPFDGANMHEVMHKTLAGRFDPLPSNISPEMRTIVAALLQCEPRKRPSSKTLLNMPI

CKLYLSVVQEIVQSSRPSRVACAIRSRGKSRRRSGCW*

>Lp_000016600.1 hypothetical protein

MTQAGQLLQIAELRNVGNVAPAEVHVVQVRHVHREGAVENADGVLCGVEGTQFAQRSEVR

ESAQLIAGDPQLTQVTQGGQTLKGSDAVALQVQPPQLRHVV*

>Lp_000016700.1 hypothetical protein

MVGHGGVGATGRHKSESEYLSDLLHFHANFFPPPPPPRVPKEERRNRAELWYHYASKWDI

THRLPPPPRTPLPEMELEYRMRLQRMTRVPYHGDGWLQMGERWFDVTQMLFDFPDENVGE

EVDFNVVLQQAVLA*

>Lp_000016800.1 Symplekin tight junction protein C terminal, putative

METLLTQIEEAVSVEALDLATLSDLRSMEEVRQTIELLLDAYNSHSGTVREVVQKQCVLR

SLAELLERVQALHEKLSSSSSSTAADSSAPYAAFWQIHLLHPHRHQHKQVGQTSSEQSNT

EWTLTADDHLLLSMFLNETATTTDASTFAYWSRAAVVLLPLATAIAYPVMPHAHDSAAVA

DAAVSRAEGEEMETTRASLESELHSLREIIAKRFTASVDSVRRVTCYEDARRCQQKGVWA

LIGCLGHPTAGARVTDELEAAMDGMLQPLHRALQEQLNAFAGRAEGGVGGGGGGSKATSP

LLLTSLLQDVEDVLWAIPHVLETGPSPATTVAAPSATPLEQLCARVASFTIDALLKFAQQ

YITHRQQRQMESMEEEGADHPGGTGSGGGVNAYSAAGSKSKDIFRVYHVQRSVQRVLSVA

LQLRQTPTIAALKLKAGAGESLLVSDAAYNGLQAAVSAFGGVVHVVDPFLLAAPANVVDG

DDVHVGVDATDASSHEPTMSSGSKTELWDGTAEDGGCLRECRSVLAPPHHLTSGHAGGEG

EGGAAAEGEFDFFNEEAATLLGRREAYLKTSLGPVTASALVDMVMLTVSQMDFLSEDAIQ

ELHRRGLEEMQLVAAYQAQREELEKLRQMERQGVEAIAPGKLIEHVKDRVALQARGMQIL

RRVSARARLERAAFSSVLTSYSHVRGEAEARVRQTQALIARCLVQLPPSLADSAMDELFL

SLRKELGKARAQKELAEAAAANGKDSGGGGNAGLAILFDDHSYYQLTLQVLFMYYATQAP

IEDRSGMLNSALMLGGAAIEEDNSNNAHNNDEGEVLRTEAGLSVEVDGPIAFLYDDDVHR

ASQDVGQKRQRDDDRENAGADEDGGAAATGVGGGGGGDGVLYTPERAFGFLNDTVAAPST

YSHVLCRVLELILSARLYSILIDVLLQAPVLTRYVWHHLYKDFCLSADQTRCVIGMGLVR

ALAVRRPVYRTCAVNILLQLSLSTHAYARRLAITAIDALLSATNPQGAPLLDQAAEAQIV

RYAKKQLFAIPAYQRGGAASKLKRAREEGEDGEANTNAASAAGGEAGAGASTSDAATAKD

RARMSTVLERHLGLFLMLCARQPRHLFPALLEVFQECVDRENMMMMQLLPDNVDVRRMTQ

DLFKADAATFVATVLPLLRKRCKEARPLVQAMLWAVRDQLGVMAREATAAAAAIATSADG

IDPAKQAAAADLLEELRSVSTAVVGHAKAMYELSGIPLGDSQTSHSLPDIRYVAPFLGFL

SAKELKQTYLKNFLLFVQVQLQFQRRYHGAFHRLPARERTYVLEATELRTFQVQVLREVF

VKCPVPFQDGVARGLTLVNFFVFLHRAPQESQARVATMLPDALHGRSLGAAGGADGLAGG

GGVLDGLHEGTARHHAPAPKSKTDEFPPISVGTTKEVVSLCMELTRSFDNTTTEKLYGPA

EVQKALQQLMHPPPVPSQLMATVLEAAELFMRSRNTEFLKFVVQTVLTPLERASVWDTDP

KLWKGVILFAECYYRECSNFLVNLPDQVLAQALREHPTLCELFKKEHGNNASFGHILGNL

*

>Lp_000016900.1 hypothetical protein

MANDVTIGDGVLEPPSTTVIVVLVFVASFAMVLFCSSALLCLHRLRSTRRLVELLTSRLN

LNTDSTTSDASRYPVARPSKYMKRFAGVSVVDANAIVDEGRYERLREFPEALVEVSEVGT

GVDSADFSTSEVATTTASVYSRSGRRFTHPRSGGLPDGEGSCASDASRFGEHSCPGFPSL

TMEPERGAGQGGGGTIHQVPPSANVKGNSGCYDRCHNMSRKQKGEALESQPMSRLRTAAP

HSRSGQGAEEGVMTNAQQTFQPSHVQDASPGIFGRRVITNISVEDLAAHCAALRESSEEE

NERNSADKGLRENGTLVDNLMDAADSSRIAAVASSFGNGAAAPQCRRHHRHTREEPTTPL

TPYEYIMRKKEKRAQRTARSVVVVKPASSSEASMSKNAGFTLAKSSEDLSSPASSQRSNE

SREFIAVERAGEGDEAVPTHRPKRTVRREHHRHHTNKGGFIVCQDFVPDLASRRSRRRQA

GSKEQKFVFQLRDEEALYGRSQYLSHQSDLDGKEE*

>Lp_000017000.1 nucleoside transporter 1, putative

MSSQVATPNYSAAPVRQWYQMTAAEFYVYVVAFLCGVSMLMPVNAVFSAPSYMVEYYLYA

TQNPYLVPKMTNFWSNVMTYYNLIGLVTGLVMEPLTLLKSFRRIPMLVRLLGGLIILIVE

IIVLMAVPARGTDEGGAVATICMAGFISGLGKSIFESTVYGMFGAFPPSFTSTMMGGVGV

SGVITSLIQIIVKAALPNTYEGVKRQSYIYYSLDVGIQGTTFFALIMLRFNSFAREHFGD

LGGVKSKVDAGSFAWDVPHGSGMEAVEEMKHDAEPVAGEVQTQVAKMKKDDPLLEREHSD

KNEDTVEALPEVIAPTSNEILRATAVFTVFRDVKWMFVSCGFTFLITLFLFPSIATGMFP

RSKWFATVSVFIFNCCDVLGRFSPSLKFMWPRSYEKRWIIVAASFARVIFVPLLLLYSYH

YIPSEAYGYVMEVIFGFSNGYVGSMSLVLGPQSRGIDNDGKRFVAGTLMGISILVGGTIG

TVLSIMTQTVREKYG*

>Lp_000017100.1 DNA Polymerase alpha zinc finger containing protein, putative

MRLCGISPFSPQAQLVPDPFLNYRLLFLTCSFCNSRISIDLLAKRKTATAPFTCSACEAP

ISASAVEGSLVRHVSQLLRAYNQQDFVCSKCREMATTFVAESCCGPLVGEAKPIDSELRA

LQCLGKIQGFTWLVESVEAALLCS*

>Lp_000017200.1 kinesin, putative

LKEAGGSNYTNEYVRGLEKKVKELEWKCADQERTISQLRAELESAGIADPTLVGTDKRGN

RAADGAENGSQRSGVVDAATYRRNNEQLQSELQSANKEIARLQTELLESEKKVPAGQPAD

ADAKIAQLQKAVDMWRSTMRDYEAHLSNYSTFQWRWTTDIVFTTFEDKMNVLMRQCQEVM

MNKDAWVMDALNRGESEQLRAAAKIQKQHNDEVSALTAQYRDSLDKIRKEYAAKEEERVK

RQREVSSTSELRNQAARETYEMEKRRWSQESENMKLNYEERIANMRAEYQEDLKRLRESL

TANSTDRQRQTTSLQELQESHEAYVKSITDDMERERKRRDNELANVKKQLTNELARKEEQ

LNSRDAAARRSEEEAAKLRRDNLKLQTDLRAKEKQLNAEIKDLLVKQENMIALSDTIVSN

YEKSPENVGRDIELLRAFISDQDYAAFRAKAKELAYRDPNLRRLSAINNSSSAAKPVATT

SDDEKKSRDVEEIRNAIENMRKTRDAHQANMQRVKQDVYEQLSRSRGFQTEGEGGKDGE*

>Lp_000017300.1 Uncharacterized conserved protein (DUF2036), putative

MNRIFVRSDFPEQHAFRLVSMDDAWVKRHCEHSHATPPETQLSTRKRKRDEEAHADTSIV

FVMKGDDQLCLHDQTSTRSVRRVEYSNVLMLAERRLNATGKAVLNDVKSTMQKAAPLPPT

RLRECHDDVVVASLTRMFDSHAANPQLNVLTVLAGSYLSIEELEAEAEERDGGEESLDDG

VAPHSRHRATSRCFTFAELARQLRSSPAELADVLQRIGAVVHRGHVRLLQPSLVYEALGA

VLTYFDAADAAAMSWAAVRTHLCPAIYPAVVLHSLEAVYGAPSP

>Lp_000017500.1 hypothetical protein

AAAAGAGDASAAAGGPASAAMTPVTAVSSSLLGESKTGGMSEHAVSTLVRTQAQKFCEQV

KVIDMDLVGMQKMVSDTMRLLQDSRYRTEAQNSGRTFAIRNKARLEKSGATVASPPGQTA

ATSAANNNSGSTTTTATAGNNDAEYGVMTEGEHELVLSIRNFVQEVQGGSAAAASAALKA

LLAKQLHISVERAANEAGKLDVQSPILTNINNTESMVGFRRTVDESLKRISAVTKRYQEL

NRTTIDASAAETERRLAEAEAFRAKLSTSLRSLKEEVNEVKETVSRYQLSQMNGAGGGAA

APMDPEAIVAGAVEKAKAEEWKEAVTAILAADDISVLLAFLENPVCKEHMAVLTSPKTLT

LPLFLSLCLQLTFEMAAQSGSVPLRLYLLSDFYVEWDDYLHSIRNQASKDAQQNAIYEIV

KRELSTVQEALEAVPHELLDRKSRNKERLVRKLIAQLLAGE*

>Lp_000017600.1 AAA domain/Dpy-30 motif containing protein, putative

MEGPINVYISYVDDFVGQHVLHQFQQFPSTYRVYGCTWDSAAAEKLNAIAAVTAATGGGA

HVAADSGLCHAADNGADAVAAGAAGVLSSGAAASTDDAAHSPLLDDSRGTLTPRETPHVD

TASLPDDCVHDSSLNRPSSSFSAGTGEAGAAKATTSAIAPAASTTAETSALLSASTFFSR

HDVAAARAALLACDWIIVELRQAQTVLEMVHFLQLQTSFVRPKRLVLLSSLMTWYATPAL

TADGGRNGEGTMDDRDGQGDAEDEEGSADDAGEAPYPPPPPTSLQTILRQAQEVARRAVV

DAMRGDGKDEVDDGETDPSMRSREEGEGEGEGDDAASGGPAEILTEDQYNRRVPHLQYMS

WRDAERAVATAHHAKGLPLSTFVICSGLPYGGEEGVLEPLFRLAWSAQPEKEDSASLDAV

AGATAAAAIPAATAGLPLFGDGKQRVPLVHVQDLACFVRKLLRCPSASLPFPERRYLFAT

DGANDNSWASIVGGVNRMFGGRCAVQVVPPSSYPLYRDVDKFTLNLRVEAESMKALMERQ

DDAETEKEEEGEKTSAGPAAVTRLSNTWVAKGGLHRHLRAVAHEFAVARGVSPLRIALVG

PPLVGKTYLATRLARHYRLPCVSLDSVVAEYKAALQALRTRIEATKTAVVTAERTRRLDI

KRRRVLVRQQREWDAAVETSEASAAGDGVAAAAAISEAAEEEEADDTGSNTASNAVAGGG

QSPPSLQREVAATAFDLTSAEEEELMRFVQDWWAEQPQVQAWMAKAAEMERVLLLRLHPR

PPTPDANPKTRAAGGGGGGNAGGGGGKKKANAGKDAQKSKEDEEELQHLKASLQNAPFQS

RALALMLRWRLAQPDCGVQGFVLDGVPATLELARMVFAEDGGDAVQPPATEEEALRPRYA

GEGAATAAGGAADKDDEEGGGSGGGGGAAGPAAAKELANDARLPDHVVVLQASDGFLLSR

MRAMGVAAEQQQQQQQQQQSGADVSSATAVDVETFQQDLQTFKREYEEATYSLLSYWECA

VATSAVDGAPKSERRTEVHLVTVEGHEPLVPPPPPASAHAEAPMGDTEKLLQNVIIGTPH

NFGQSLEEAQRDAVRQRCLQEEEESAHAALVAAQAAAESKECAAEQKARAAEDDALAELK

KADLAELEARKRPMTRYLQKRVLPLLHKGLLEVCAKRPEDPVDFLAEWLIRHNPNDDTIC

DL*

>Lp_000017700.1 hypothetical protein, conserved

MASQAPLSSRPANTAEDTLQQSTKMTATRRNLSQLFDGGASSLARPTASDTTGAPRVSHD

AVIQSTLNPSLLSSSASMATAATGLLAAPRFTYEGRSSTSTSMHGGSNRAPGGAGSHTAI

ESDSPAASAAAGLTTALHAVVQAYRDTLPCGTCVLALYVPPKVSNTAVAAAASPLQNNAN

TLPSSSASSLSSLLQQQLQLPPSAAARERLQRDGSHAVQQQLTASSSSSPLLLLMNVNRQ

VLCSFPLDRPDVVFDNGGLQLQQDRQQPSFLAFFGASTPLTPEATSSACCRWTCMFQDRE

KASEFLVATYTVAQHAATLAKKAGTLSGVVPTVRVMPHSSSSSPVTTNVNAAHDGVDGGD

DAVESGLDDVEDSDMEGPGGHDSSRVRLGVPTKIYWTTWMLRRVSRATPYCLPTDVVDGT

SPLLPCVVAPGAGVLRDGLEAALVGMRSGESRLVFMSAEETQVRHPEQTSSLTPLSMSKS

PSARGSRRRRHHDDVQDARAELRTLDKPAVAYVTCVEPVVSTPTTDAPPAQPAPSQQMAS

ADLANSAASVDTGVLLQQLLVNPLKQQQQQQQQQQQQPQGSASAFGALERSLDRVMLQLG

SLYEKVDRLDIEGKLARNNAALEQAMKRVVGLAPQDDVAVEDTLKDRDALLASIERYRHQ

YEEANANYQQALEAVGRATERAHALEKDLQVQHDLWTRQRNDAAEQTRLKLLEKDVRHRE

ELERVAETRYAAGKSDGHAAGYREGRQAALTAIDGEGGVNAVTAEWRAKLTARDQQIVVL

QTSLQDAKFHHERDRRQLMAEIDVLTTLNEKLQHLQANADVRMPEETTQQQCKRVKRTLN

SVYAQLEAQLLTLPLHRLANGDHINAAGENESIRVVSVDDALAMVMTVIRAEAQSAVAQI

KADGERRAKANADVRALTLARQHTQRPAVAYGGEEAAAVGDNYKKLRDDADSNGVRGRDA

AGSASVEAAARDPTARSSENTSVMSPEAGGERGTDEVDRSPSAFSSSSSPFSAASATPSR

QATRLSDPPPPPSMRAETTHDIHSDMSPPELTGRGGAEYPTDVAHASSSQDTTDDLPVGE

PAVSAA*

>Lp_000017800.1 hypothetical protein, conserved

MVDLDSVVGKVLVGATSSTTAPATDGAREAARLFLRDVTHTLRCHAVVAGVALAFTPVVY

QLTRFLAQHCRAHLVSMAKSVEQRSELFDGSEADGVIIDPADEGGVTVSYNTESLAQHRR

SSGKKQGDGDSTASPIESLYIRSIRSNVRSSWVLSSFAHRVLAAQRAPNVQFVRVCSRAA

VLQYGVAVMQLCTTSCLFSVAALSAAHVCCTVLDKAVPLVDASSNGSSVDGTSSEGGVAS

FVAALRHFVVGPLFSTAAPPSPSVGCSYGLYNFGVSAASAAGRLVPLLGTLQTQLDRFFN

WRQPLYREWRGDVGSFGVISPADPFMSSPSTSATTTTTTTVAAANARADAVWAALTPRGF

FIVTLLRQLPLRVLGGVKELGWSVVGAVRQWQHHTTSSAADSSSSLPTATPPSASSQQQQ

QQQRVPTADAASVKKAKLHTRLICIPIVRVATALAGDVAFAGVVFAATLLASSQGSNGGL

PVLVHNVAGARYNAYCWVMLVSTILNLAVL*

>Lp_000018100.1 Calcineurin-like phosphoesterase, putative

MNALTQTEQAEEYIDRFALRELVEEWLSRVSVECPEDPYQYLIDEAVDQRRSGGGIVTCP

NSWCNMTMPASRFPAHQQMCNNAANWVRCVRCNLRVEAAKLSQHRLYCKLERCVLCGEMV

LPRMLPMCPYRKVAKAERDRQAAMHRKERRLEKEAISAVPLITREAANPPQSRQSDVSLS

KVTSLPASPLPPPASSPSSCAGKLVVTVRSQTPVASLETADPNVAADSGSLNTCALGATV

KPIMKASSRASVTQRHKSQPGCGTASPCTKQEGKSTGDAASEVARGTTSGVPSEGGESSS

SSSVPRPSFPALLAADMALREKLENYPAALVPVLSTIQAFWRRNMTLHLFREQVFSAVWR

HMDSAQEGAAGQSKEGAAIRLVERNLRSERRLSSIDNDLGGNAGSRRRSSTRVPAAASAA

AAAAAAAAAAVDGSKELSSSSTSDVLKDNDVCADEDPIASFQSDPVYVADNALGNGGFMR

VRDLEALTRHCQAREVLQFSVVLKVVRAATKVLRQRPLVQRITIPANSSLVVVGDLHGQM

KDLEYIINYMGLPSAERYYLFNGDFIDRGPYGCEVLMFIYGLLCTYPEHVFLNRGNHENY

STNTEYGFMAELYAKYAGRASYLLDAMTDSYEVMPLMSVIDNRVAVMHGGAPRLLCKLDE

IEAIGHVRDIPVEQQSTRAEQLLAELLWNDPVEKFRSRQLGMNHQGEGWRTSSRGCGVEY

LSNITEQFLKNNDLKLLIRSHDVKSAGFELIHKNKSITVFSASNYGGVSGNRGAVAVLTR

EAEQPVFHTWFLKEDHREYQQEGLLDGGIDAFLQDGRSRRHGVATSSSSSPKKLGGVPVT

GSNATGAETVLAPGMMAKGSVSSATRLGDTSGALVVVGGGGASPPNLQNSASFGASVQPQ

PPKRLNAAMLAEASLMDGDEYICAFYLTSGAVLDDEDLMSDTSSRSVAVSDSEDRISTDH

AGGANSAVAVAATSASASATALAPASVAAGDANCASLARSSTSLSTKANSTSRASSLTQR

WPTGSAVVAGAGGGSVNNARLAATGSRGTQASLASHLSLSSQGSLRVGCSLAQDMSILVQ

LQTLQQIRELIYFQRYALLVAFNQVDEMHTGTVYKAEWCVVMRDVLKLDIPWYYLCQFLA

PRLVVDGVPSVEYMRFLRHFDVSFAIDFRLSWQTSTVQRISSGLDLPDDIINAFCERPTV

NASNTSNRNAATASLCGDADSSSPSLHGKTTPELLLTSPPTMTPMELPTAMNEARLATMD

SAPSVLANPAKPLNLAASKGQGDGEMSATVVSATDLTTSEKLLTEDEDWWHDVQLDFKTF

AMKVRVLSPAAAAMEDNEIFALFCFFDVSMQGHVYVGDMVDSITAVVDEEGSVNEAELLV

SGEIDFSTVTGRGPPDGRDDTCQPCSPSCSISISSSSSKTGETPCGVSNLANAKNDAASP

SGRNLLASKLKMAKGKSRLMRPPSSPSSSCSSSDEERHEAPHSVSHTAPSSLLGGGRSVL

HRSSSSSSKETDASSSVTSTRPTASGRNSDALEAPLPLLQKNRDGSGARTPTKQGNSFDS

GLAPPILASTGRLENFKLVTPPSQVLALPLEYDDDETPPNDIEATTACDAEALSASKDMK

RTENTAAATAAAPAAFAHGEKGRASRSLACNVLARKDSAVADIGGKNSGAAAPTSLVRVH

SFNEYAVSMASACSVSTPAIGASKPFDDFTSGIFQFKLDNESSARGTGSGSSGIGTGPTP

LLSVEQRVNKRLAMPPWIYPTLLRVQEQLLGGYVRLRFLFQALNRSRTGHLTEAEFLPLM

EFMNCVFEHSLSAEQAHMLFVYVHDSAINYMQSMRNRRRQRSSSLYSEGFDAAAMRRSPV

GTVAERVRSTGEADAASAAPVDNCTYTRHRMQEEQARGERYILLIEFLAFFGVKPVQYED

DLEALEELLYEVTGTSVAAATTTVSTFPDVARESSADGVEGGRPFASTGMELDDKAGSLS

IVSNTVSAAGGAVTCVDSAERHTPAQMRVNGPLIETPTSFRMTDMADIAARASTAAPLAL

SRAELDPSASQPHQPGRLSGDAATTSSNALPNAAGHPVKNTSFCGPRPRLSSSSAIPADE

R

>Lp_000018200.1 hypothetical protein, conserved

MFVRIRGKGACPENSQAAVMHIRGFRHTHSAAPPDCFYPLLTAVAAASATHPPPTTTTTS

SSSSSPSPPLAADAAIHLSVQMRPFRFALFPVGIHAATLTEAQARWITPTATTTTTTTTA

TADAPASPLSILDSEVARMVWKETWNLYDILYDEVPLLPWTEDASAAAAVAATVEEELQK

SVGLATAATVLQPLDALAAETDGFVLASHRSGARQVVVCPHYYAAAALPESTFTKIQQSA

PAVSAVPSHGPVVVLDLHELPVALAGGKAMANVAWYTAFAAMQPDSALLRARAVVVRLPY

AAVPNYADALATTSAATAPTMAAAFVELVDFIGEHITTVRHAMLRELGSKTAELVQAYGT

APALLVVGRAPEAMSSCLHALLDVSAADTASSGVVVFHGDHLRCAAGGAAETSPAHEFGA

RMAAEYRAIGEACGCAMAVETVLVPLKALLDGEEEPWDAYTLRERTRLNFCPCCGDCGHD

GESDGADHGHSHGGHDHRP*

>Lp_000018300.1 vacuolar-type Ca2+-ATPase, putative

MGATDIVVSSEEAAAHHPDDHRPATAGTAAVPPHTVPLSAAEKAIGGQLQVPLENIFGRA

SEGMPMYKKLGSVAGIAKSLNTSLKDGLDHQSIEARRHFFGKNALPEDPPLTFWQIYKGA

WEDSMIRLLAAAAIVSLILGLTVPEPGETQVDYRTGWIDGFAILCSVMIVTTVSSVNDYR

KEQKFHKLTEENSAQPIRVRRDGRDVTVDVTEIVVGDLVNLSPGLVVPVDGFYVTGMSVV

IDESSVTGENDPKKKNAEAPIILTGTVVNTAEDAFMLACAVGERSFGGKLLMESRGAGAP

RPTPLQERLDDLAEKIGRIGISAAVSLFVLLAVMETFRMLRNKPNTSYRHFLDYFLLCVA

IVVVAVPEGLPLAVTIALAYSQNKMHDDNNQVRRLRACETMGNATQICSDKTGTLTQNLM

SVVQGYVGMQHFFIKRPGDLPESIKMPDIKPSSLEKLIEGLAINSSSEKVVSTTDKEGHT

VAPYWQWVTDKGNKTDNALLDFVDRVMMTEADAKNMKERPHQRMREACRKRGFTVFPFTS

DRKRMSAVVRQEDGTLLHHVKGGSDRILPLCDRYVNEAGDEAPITDEVRERIARQVKKLA

DMANRTIGVAYTILNEKDLPENEPTEKLVWLSLIGIQDPLRPEVAEAVQKCQMAGVMVRM

CTGDNLDTAVAISRQCGIFNPYYGDVAMTGQDFRNLVYDAYGDEERMEKFWPVLDHMTVM

ARSQPLDKQLLVLMLMTRGEVVAVTGDGTNDAPALRLANVGFVMRSGTDIAVKSADIVLL

DDNFRSVQRAVVWGRCVNDNIRKFLQLQLTVNYVSVALTFIGCLMAGGASSPLTTVQLLW

VNLIMDTLAALALATEEPNEDCLRRGPVSRKAPLISNRMNLTIFTAAAYMLLLTLAVEGY

GNVWFRAGLKEGVEHQTIVFNVFVLCTLFHMLNCRKLYDELNMFEGIWTRSRPFIFVITF

CFGFQILAVETFGEFMNVTSLRKDEWFACAMISVIVLIIGFISRLVPVREPVFDKTFDAE

VLDDDAKAMLTKLDSTVATAQEEDAVYGTGEYLDRARRLRARALWREARHHHVNAGRVLK

AFRRARAEKLEETTSFAAFV*

>Lp_000018400.1 hypothetical protein, conserved

MATTTSTMKGQGAAARTGLAATTGTASVNALDDPNLLSTLVKSAKSAHSTLGRALDTTLS

HQRFGGAEMEPSALTMNRLERERQLRLQQDLQRACGDTSLATRGSRTT

>Lp_000018500.1 kynureninase, putative

MRNAATEALLSTVSTTGMALTEDAFADHMDSADPLREHRSAYHIPSMRDGTPFSYLVGNS

LGPQHVGVEAAVAGFLKKWRDQGCEGNTMQPNPWFEADQACIKDMASLVGAKDAEVCIMN

SLTVNLHLLLTAFYRPQVAKKKIMIEHKSFPSDTYALVSQLEMRGMNPAEDLITVTAPGT

KSYDDPVSVIPTEAFLTAIDKHGDDTAVLVISAVHYLTGQWFDIPAIVKAAHAKNILVGV

DCAHAVGNVPLQLHEWEVDFACWCTYKYLNSGPGNIGAIFVHNKHTSTSTPLKYLKGWWG

NDIKTRFTPHRAFEPAPGASAFQLSTAPAASCVMLGPSLKLMATVGLQAIRQKSLLLTAY

MELLFTELVPPGCVEIITPADPNQRGAQLSVRLLPNKLKSSELDSAAYEVGAGAEGTDDA

SILQRQLLDEGIMVDKRPPDMIRLAAAPMYNSFSDVLRAVRTIAELF*

>Lp_000018600.1 GDP-mannose 4,6 dehydratase, putative

MSASNGNPPMQHGDASAVFSSDMSTSSLRLVTSPAEGNESVRELLQHLPGRRVLVTGGCG

FIGSCFIHCLLRWGPSDVQVCNLDSMEYCAGAQVPLMRFADSAVDRSGTADEKSLSGSAA

SRYHFIKGSILDAELVLHVLRTYQIDIVAHMAAQTHVDNSFLDSLRFTQANVVGTHTLLE

CARIYGQLTRFLHVSTDEVYGETVEGSEAATETATVLRPTNPYAATKAAAEHLAFAYFHS

FRVPVLVSRGNNVYGSGQYPEKVIPCFITKCLRGERLPIQGDGHYKRSFMYVEDVARGLL

TILVRGVLGEAYNVASTEEWSVCEVARRVVACVTGSDAEAARADFDDVYVRYVEDRVYND

ARYFISNDRLTELGWVQRVPFEEGLRRTVAWYKSHPLDGGYWEENPS*

>Lp_000018700.1 ribosomal protein L38, putative

MPREIKTLKEFLAICSRKDARCVKVKKNPNATKFKVRCSRYLYTLVVNDKKKAEKIERSI

HPSVKKIAVTARSHAKTNAGAH*

>Lp_000018800.1 hypothetical protein, conserved

MLYDDFHKPFAVGVGLIALFYGYNHLLVYLSREEEDEGRRLDAQSEEMARQTGKLKADRF

LVKPSRQIDDPDFLDIPAFGGKGVNKSRLMSDDSVSSGPLHSERNRS*

>Lp_000018900.1 long-chain-fatty-acid-CoA ligase, putative

MGQAVSWYFDGKNSRSEVDFPPYLDYMSYGRQSLLVKGTEKDDRSPIYRMSKTSDEDFKR

LRKTWYEDGSCLAVVEDYCKTRGKMTALAYRKLSHIEKHEETTEDGRKKVFETYVFHAKH

QTISYEQLWQNVLNFGKGLAEIGLKKGDTLSLYEETRWEWLNSLYSSWTQGLVVSTVYAN

LGTDALQYALNETQCNAIICNGSKVASVLAMFKSIGAKSHTKIIYLDKLPSGVDTGEFEL

YAWSDVMLKGQSSKAHYHVPTAEDREELALIMYTSGTTGNPKGVMHTHGSLYSGVMTLAE

RVQDIIGDMKEQEWYCSYLPLAHIMELAVTSVLMRRGVIIGYGSARTLMDHYAKPHGDLT

EYKPLIFVAVPRVFDTIKKAVEEKLPKPGTIKRRIFDQAYAARLKALKEGRDTPFYNKEV

FSVPRSALGGRVYAMLSGGGPLSAATQEFINVVFGMVIQGWGMTETVCVGGIQRTGNLDY

DSVGQILKTEEVQLLDTDAYKHTDQPEPRGEVLVRGPFIFKGYYKQPELTKEAIDDEGWF

HTGDVAAVEACGALRIVGRVKALAKNAVGEYIALETLESIYGTNDVCLPNCVCVLVNPHR

AYIALLALTNKKHVEAFLEKNKLSGSFPDVLQDKAFQKAVLHSFQSTARAANRASFEVVQ

SVKLLTDEWTPENGVLTAAMKLKRSTVDEKYKKEIEELFTKE*

>Lp_000019000.1 hypothetical protein, conserved

MLRKSGVLLDKSMFAAKRRVIVPIHPTPNFPAHFIKAAFTTDPLKEKQKARFSSGGEAMR

EVQDIPKRLEGQRSRAELAARGDEEFAALIEFIQGASYDQLISGRRFKKIYDKLSENDDM

FVWLCHTAMAVLNPGDVRSRLIYNHLKALAEAVANGEMTQRTAFRFYESAVRSPAYREIA

ARQLETGAATRLAGIAAAADVMRDMGLTRRPMSSYFELYQRIVERSEAMTPWGFPPLFQF

EERLALEPRLKFFSRAGQQQLERRRRGSIFSPHTILQGRRIFWIPPTWNRAGRFIGPHIN

LYPGMTPD*

>Lp_000019400.1 hypothetical protein, conserved

MSNLFQRIFVQVLTKLGSESTVIQRAAQKAARMQYDFTHNTLTPASEKAGVWAGAAAREV

YLDIKAATAYLKNASPPPEPGKQEAPHKNESTSAGFAASKGQKSDQQ*

>Lp_000019500.1 hypothetical protein, conserved

MGSLAGESFVEEVCSASTGAEAVAVVQRAVEAPTVFFYGALLDAVRACAQKQSSPVQDDS

LRDWIEVVELLSFKTVADVDACAAGIKDIVSKHPVIVEKLRILSLLTLCSQHNMTVNGID

LPYEAVAGAVGVKGSIEVQKVVLAAVQHKLCVARLNEKTATLRVCTYESRNVEADEVAML

KERIDAWISYTENQLRDIQ*

>Lp_000019600.1 hypothetical protein, conserved

MQNPATPSKPLLVPTPTSRAAPPFIMVSEEELAPYSSTIVSLPPTIPELIQCWEQEHERC

AQMTRFVERVRMEATKMAAAAPLITIATEALAASPKAASLSTATSTPPPPSNTAAAAVTS

ANASVNASRLTGSFVPRDVATDGSAATTTAATGAATATATAAVVSSGSVVRGTVLTGKVV

SADRSPTNPLRAVVASVSDTSTPVKPPSSSSPTATTPVAPLVSPDELQVLRAKALAAEAA

EQLLASTQEELKAAERHLVCEKEKLAKAEDALAQLRAEVTATEAGASTASQHDTPTPSAL

FAQLQVSYELSETVLNDTKLKYQSVLYALAQLNEKMATNTREKAFLEEKNTHLEGQLQRL

LMSSVVTGSLTAPAVPAQRASNRNTVGESSTDGAKPSASTATASVPVTAGASRLSASESY

QKSALEKQIGELQSVTDKLTRDLHKQTARRAKAEETVRTLQKEVMDLKASALQCRRQINE

SELELERAVSRKEDDERLRQLERDGNRLRTALRERTEHFLHEKTEWERQKDSLNSRARVQ

EVATRQLLRRLFACQVREVVLRQCDYAASTRRNEAQSSLHASRMTTPAKKAAPPSTAATG

PRAHAPPQRPGESPTQTSATTNACPSSAAEVPTAADMTTLVNRERQLEELKHTFERRVAL

VDAQRKAELQQLHALNKELRAALTASQEELNQKARLLNSLQQQQQPVAFSKFSNSSANLT

PMAPGRSSSSILDRRDSAVAWVCRTDGDSGPGASMDELSDGIGGVYSSVHTPVVALPRQV

TEIERRYDPEHMSTWEAVQVENEALLDRLTTMQEEKWKLTSYAEDLQRQINVLREELRRN

ASTLNQLLAAGVLTPAAVSRGSAEGSLRALQCLLQETLEEKFALEEQLRSLNAR*

>Lp_000019700.1 Fcf2 pre-rRNA processing, putative

MSNQSVEDMMAKAVTNTGDFYGGRNGVQGIQDRKDEIRAKRKEQKNTLSQWYGMKKRPLS

ADEKQEVELLKYRNFIDPEHQHQAPKKTGDIGDNEFLEFGYFADIGRNKRRRYKSFADEW

IEENPQFADVVNTRIKRNAKANQKAKATAAKKAAAAAAKAKEKRTSKRKSKHDLL*

>Lp_000019800.1 hypothetical protein, conserved

MSRANVFGPNSLYSFTKFGALNRSNGVVLNKRMKDVFRLENQKHMRNDFDRERRYRFCTR

CGITSVTVNFDRVPSARVGLWGRCVNDQDYTHHRFTELSQREYEQVRDWPVEKRLNWWRF

ENDS*

>Lp_000020000.1 RING-H2 zinc finger/Ring finger domain containing protein, putative

MTSTHQGIDFIIRRITAEQAVEMTPQLWAQLKSLPPEDMLTQSKYIDLLCYTARCILAKI

EKAGFNVETWKSYTDFFTQAIMAYGTADMPSYLRIAERLLSAVLGFPSGPQPLLKEYLNC

AVAQNTQRSAELAAHLATQPSGALPQWERQPDLEYDDNFVAVIKAKPVSEAMKILEKLPS

QLTFAHRRAIMHLTLCNPLPIPGCAYVSTGYCCDTCHLRGIRVGFQAMLYDAEEGSSSSS

TGFTSANVRSDAQISKKKTYGFDICIACAVTFYNQQRKSLLALLRFPHESYSFGHASGVR

ISQVRYHSRRRAVSPATSSSAFPLSREHSGTEAHLRSPQLSGSLPSQERRLSGQSAQSGS

PCTSLSKLPAGDFGIHVGMSNSSSCGSSASSADGTAERRATAKVSNQPPPVRPPTRTKNS

GLHAPPPPPATRCATEDVMCITLNVTIAPYGARPVAWVLSHTEKLVELDTMEEVLRKDIG

PSSQWRARVKAEAASAIRRRRVSAASSGSFAAQGSAPAIPRTVAAASHSLDAAPSMPTAT

VAGDSPRELLGTMPPSQTKVASQHAVLPRVSSSLASKEEEEMCAICLCPFESEEPVIETR

CHHWFHVACIEEYARIAEDVCPLCRAECALPDMSLATTLKNNAYKIVVELTEEQRQLPYV

DVCVGSVVTRDGNYHNATSIAAAQCVRVRPSQLRGFQVKSPVKALASLPLTTA*

>Lp_000020100.1 hypothetical protein, conserved

MRADDEFLSAFSFADEASSLRLSADRRRPASPSPVRGSSASSPTLCSLVSDAPLYSSVYP

FDFYGVGSTAAGSSSAPPALAAAAAATAGTAYSYGAWNAWKDRGAESCAAAEAAVYTAEK

QRRVLKMEMDLAAEQRGRLEARMALRQQHSASRRSPDAVDGAAASVSRGSSAQGGTSSHV

LCDEAERRMCELVSSREASAEVLAPPPCSPSPPSAHVQAAAAAATGPLFADDPAVAAAAE

ASETTAPPSPLKAKPAAFARSTEVKSYTAVSPAEAVDCFARSSTASPSPSRAGPETEWSE

SPSASRAASERRSPRTAAAADATTTTAAAPASSSFAPKASTRVSAERPPSQPSPPATQSS

PPQSSRARKSPSRSRAAASVEELQTRREPQKSLNAILEEEEEEEQRMYAAYQRKLARIQQ

ALRTSHLRPSAAEGSASIEARLAYQQRRHIGTPSAYHPDDAIGGKGSAWPLQRPAEPHAV

AATAASCSTDRRTLYATAGPLLRRAPSLESAAVVVTQVGGPACQLRWDFVHSGNVVVSPD

GLVCRADAADAIALIEAEYADRLHDVLPRLIIPFYAIGSLGATRDTLIFAFRWISSSSFK

SGGARPPALAFGFATRGFTGYGTETPAFLYLSSGTIAQGLPALDNLDVEHRYGPPYRPGL

ELAARLDLRRGELEFFVESVSMGVAFRFCPALHPAPLYPVVVFSMEGDTAELLYSA*

>Lp_000020200.1 B9 domain-containing protein 2

MPELHIIGEIQTGYNFGGSSYFCTYEIITGTQWTLIEGRTSGSTHVMRDSGDGIAWNFPI

DVHYSFNSVQGWPKIAIQVWQLDDYGCKDIGGYGTAYLPMPSRGPQDMQLSTWRPNLWSP

SALVRLWQTMRLFIMGGYPVLRDNSLIADNEQRFKLHTVTSGTVKMLFTVFGRGMRQAGL

IYA*

>Lp_000020400.1 amino acid permease

MAHNYSSSPPYTPQREEYSDRASGSMPPDNDNSNTCTTTNTTRPTTTTTATTRAKREKRR

SRKHAQPQHTAGCGGVLNRIGNGVIGVVHKIVPPGGIISGAFNMASASIGAGILGLPAAS

DSAGLVLAMIYLVIITYFSVFSMYILALAAQSTRIKSFEGMARWLFPSGKYAFSYWAAFI

RWFHGFAGCVAYIISLSNCFSAIFGDAMKRHPDNSAIRYLSKTSGNRLLTSMVWLCVMVP

MVVPKHIDSLRYASAVAVTFMVYFVIVVVVHSCRHGLPENRHHVKVVGDQDNDDKLEFNT

VFLFRSGNSVIHSVGVFMFAYVCQINAHEIYWDFRPEIRTTKNYTWAAFIGMLLCGTLYV

MVCVFGYFDFGSRNLLGKSLLLMFHPLEEADVMIAYVGVLIKLCVAYALLTIAARNSMYY

LIGFQHRYRNRPDAAAAAAITPQHPTEELARRAAAAGPTNGDAGSNDDVVKPTASGNSTA

GQAVAEQDLVDGLDEDGVSCRVPNGKQAAVPQDVAEDVAADGVDKDFEDNTAEDTTYVDN

IPFWQHLLVVLLLAVASLLCGLFIPNINTVFGFAGSISGGFLAFVFPALFFMYSGNFSLA

QVGWFTYLNTYVLLICGVVGIVFGTGGTIYETI*

>Lp_000020800.1 PUB domain containing protein, putative

MASVSIPKQWEELCVSTDPAAFHECVDGIFRRLLANLLLDFQNPKYRTVKKGNKTLHRLA

SPFPDEFIRFLFSCLGFVEHEQTFHFEGSEETLKHADQVYSRLESLVDKVRLDAAERELN

QLKTLTGKKAPHPPGISAATSTQTVSKLNMAAAKDLEKQERLHSQQENTQKDMAEGRLVE

EAVRKTLLNTGRIRNSFFEAKDLTVRTMRHGRVYACTEKCGNECLEAHWHLLTGKNILYS

YLAHLNADGTRLLHLGVEHGYQYNSLPGSPHFGKMVHFSEKLTDENGKLIRLQHDDRPAA

SCVYCGRLFSELLL*

>Lp_000021000.1 hypothetical protein, conserved

MPTETPKAKAAPPPTAAEQATTTKEEAAVTGAGHKDSSNDNNRNSGGGSGGSREWRSRLM

NWRTAGGDSSSSTAEDVKGGDTTTAPAAPAAAAAQGGKAETAQEKEGGTLEKGADKAASA

AAPPRPMNIWLRRAIERQAESAIAEAIRDGKPIPAAAVAAVAAAEAAAAAAKSAKNSAEA

ATASTTTTDADATAAAVAPKVVTFADLMRQHREGTLAPVAQPPVKTTTVVKKPKTTKVAA

EHQSETSAAAAAAAAEAPATTPTAKKRKTKTTSESAEVKENPTTATAATTATTGAVHQGV

VRKRLKASSSSSTALNSASVKPIQESNAAATPTRTITVKGKVSLEELRARRNRTAGGSAH

STLSTPSGTGDAAVAPKSIAGKDGYNERALDAISQLLLRYCLANLTPSPVAKTTAESAAP

AKKAGNQEEALATTGAATTENGAALTTATTSELAFLDQLMEQFETHSLTPASLELLRELL

QHVRVLPRPATTAAANSSGTVEKEGEPNTAATSALAATAAETAAVDTEAYREALRRREYV

QQVIMVLINQLRQLKREQQQRQDYETAAAATTAAVVSTESNGNDATGTAAVASAVAPQPV

IGHAMPSTIKVGGRPLNPLPIGASAPRGNAQQQQQQQPCTLAYDEQTRRVAEIAAGSAAE

DETMAAAAFASMMAAYGHMNPYAHLTVFSQHQPVVPPVGWGSFTTSRSSSGSMGARCGCD

EPPASTHSSSSQHTHLNNNVAARLMPSPFPGAFPIIPPPPPSFASHGGDDGVTTTTTAKE

DREMQELFRVIRRQLTQPVHHGSQGTPVSVAATAYPSAVDVSQAERAILRHVQEDFKKQQ

QQQQQVSIGSSPAPQAPPSASTPLAASLDASANQAAATSHRSLFTPQGKQQSPRKEVEAE

AEQGASPPARSTTVTAAEAAAAAAKDDEKPSVTSASASRTLNFEAKPFVPAQAIATVSAA

ESTPRKTTFNYMAAPFIPGNKAASSSESNCGAATSAAPATRKSIRFEAMPFVPHSGAAAT

TDNSCTNGISNSNNRKSSTGAHVANTSSQMNAHAQPFVPASAPQRAVASISTPTQGSRSH

HPAGGAQAIAFEDASELVNTATDPWASYEMFARWRDIMETLWSRITKVSSPHSTSQVSPR

HSQDR*

>Lp_000021100.1 elongation factor-1 gamma

MPLTLFSGTHRENARTQKICVAAALAGVGVEPHFRTHGVENETPDLARTLPPCTRLPALQ

TDEGAICESHSIARYLARNEKRGAKLYGASPCESSQVDMWLDFAATEIDAHTLPFLAAFF

MGPPVAADALAPREESLAGLELWRETRTFLVGERMTIADLAIALALQWTYRASTEQGGAL

TKKFRNVSRLYHTVMQQPKTVEVLKEGGATFAPANPPKKAEEKKPKAEKRAKAGEGGGGG

GGEERGGEKKKPNPLDALPPSSFVLHAFTREYSHTHTRTVPAPYFFAIYHPEGHTCFWCR

YKYNEENKMQFKTANLVRGWFQRMEHVRKHAFGRALIIGEDKKHDLVGMCVFRGEGMREI

VSEGVHRDLFDWEEIKGVQGEKDKITDYLCWGGPTIPLPVLEGRCFK*

>Lp_000021300.1 SLA/LP autoantigen-like protein

MDDRCLKLAEELVSSRYIEAGRESLHATARTLRSILAQRRCPDKGLSDAEIQLFLRQLAL

MDTNNFASHIGGGEREGRVASALVRARHYNFTHGIGRSGDLVAEQPKAAGSSLLYQITNV

LLLDLIRLSGAPSTEAAVVVPMATGMTLALVLRCVAKARVKELQQQHQHEKEKEKSNDDK

QESKAASSSLPSTVTPQYVIWPRIDQKTALKCIDAAGLTAVPVQLRPTAPLSQHAPKADQ

AVTSASASAVAVEASCTASVSPTPFFLQCDVNDIATAVDAVGGPAHVVCVLSTTSCFAPR

VPDNTVAIAQYCMAQGIPYVVNNAYGVQSRRIMTRLDAAQRLGRVDFFVQSGDKNFLVPV

GGSVVCSSSAEKCHIAAAMYAGRASASPIIDLFITALSLGRQGMQALWADRYQCRRRLVA

QLRTFAQERNECVLADDDDDEDDVDSSKGEGNGAEAHIVNERKTAAAAARHELKDKKRAA

PSPRNDISIAVTMRSFRQAAAVEAGTEKDKTPPSSAWAAARALGAQLFRSSITGPRVILP

APTTVTAIAGCTFHNYGMHQEGEPACPMLVIACGIGMTGYEVDALMHRLRELWPVVRRQ*

>Lp_000021400.1 translation-associated element 2, putative

MVKQRMTALDVRATVEEMRAQLIGLRLLNIYNITSKMFLLKFGQGENKRNVLLENGIRLH

LTELAREKPKVPSQFTLKLRKHIRAWRLDSITQLQHDRTVDLCFGVAGTEGCFHIIVELF

SKGNVILTDHAYKMMMLLRTHRDEEGLNLVVHETYPVTAPFTVGVDGSGGLARGDTPAAS

SVSAAAAAVGTTEEPHVMLYPPHIDAAGRLHVHRATDADLTPAQQQLKAERTRTLKAEWD

LGLSRSNDKTFLQSLVAGIEHFGPDLAQHVLTVTGVPNAAQKNWKESPDAVFDKVLPGLL

EAHDLAKVDLTSAGGYLIKKTAGKGKAKHDAAVPPAASVGPAATALAPAVAPSAAVTVAV

AEEYESFTPILLAQYTQDGVEALYHASFGRVCDDFFLITETERIDASNEKRKSVAKSKEA

KFAADHARRIHGLEADIAMMQRKGAQLILNAEKVDEAIQLINGALATGIAWDALRSLLKR

RNAEGHPVAYMIHELFLERNAISVLLESTLEDEEEDCEVPPLVVEVNLSKTAQANAADYF

AKQKQSKSKLERTVASTDKAAAGAARKGARQAAEQKERKVIVKERQRNWWEKFFWFRTSA

GDLVLRGKDVQSTELLLRRVKALGDFFVQCEVDGSLPCLLRPLSQLWWQDSHRSSSGNDG

GESSSGEQAGNASSSFSSPAQPIAAASLCEAGAWCVAFSGAWESKQTTGAWWIYASQVTG

GSAAGMYIFSGDRHYLPPQPMSLGCALLFYVTRTVDQPAVIAPADSEAAKDEKAEEETEA

GEQPAPEPSREEVLLQELPRLLSKAQLKAAATDVTDLDTSAGDVVPADSATALADMPTIE

ALRADQRKQQQAYRGHGHHHHTSGNNSNTKIGGGGNSIPKGKKGGNYARNSRVDRDALQG

GGSASGNVGVSVVAEAEAVADTRTAANTNTQQQQQQQGKSLSKHQKKKLKKIQDKYGDQD

EEDRVLGAKVNGNQLSRVQLLELERLAAARQQEEAERRRHEKTSCKAARDAGSLRCRNVE

DAAVKVGTPIQEEEEEEENEVEEVSDFTDNDNVDDVDNGLAGDSEKEEPATAVASPTDPM

PGEGHANYKDGDEEDDDGGSGSQVRQASPSATTTATTTVKNDDHNAMVVQQTAELHRVFP

HYTMRPSPSDVVQHVVAVCAPMMVVNDYAYHTALAMGNAKKGALAGKLLHVFTERAERGG

AAAAAGAGAGAGVRRKGQKKTTKAHADPSKLAAEPDDGTAAAVNPSVVKALQMMSSNDIV

EQLRANVKPVDFKI*

>Lp_000021500.1 calmodulin, putative

MINEVDQDGSGTIDFPEFLTLMARKMQDSDSEEEIKEAFRVFDKDGNGFISAAELRHVMT

NLGEKLTDEEGGGRDGPPGRGGGCRSNQLGGVREDDSEQVEPKSKSRKAARQ*

>Lp_000021700.1 Leucine rich repeat N-terminal domain/Leucine Rich Repeat, putative

MTRLFLSRIAASFPSQLSGWTGTDYCSWSGVSCAANGEVSVDLSGRGLVGKMPEVDDDEG

RLSLVVSIDLSNNPGIRDDFESDWKHLANLRVMNLSYTSLRGDIPNDWRRMSSLEEVYVH

HTAACKSLPEWRGMANLRVVDLSFNNFQGSLRQEWSQLPALQSVNLAGNAFCGCVPQSWN

GNAVLTNAAAGLAASDASTCHANRCTRAKTCPYEPNSVTTAAPGSTAVPTSKRPATTTAP

STPAHEKEMTRLFLSRIAASFPSQLSGWTGTDYCSWSGVSCAANGEVSVDLSGRGLSGHL

STLRGVDDPAVRVVSLDLSCNMGIGGSFVDNWASLSRLRSLDMSKTSVHGFIPSAWNGMS

SLETINLSETQACGGLPNWGKSMVSLRMVNLSHTKMRGVLASSWASLPALESADLSGNAF

CGCVPAAWRKNAVLEAAAAGANSGLTATKCAKKNRCSLMSYICW*

>Lp_000021800.1 Cactus-binding C-terminus of cactin protein, putative

MDVLVIRFCVHYLDCSQVADSTQFVRLAQNVVTSSVERVLVPQLFAKGSASLPALCETLV

AEHTADEVQTAVREINEFVEARGALGAWLADAAENSRLADDDDDNFDAESYGGAYKRAAG

DRDEESASLKHEEEMGGIFVEEKAGGALRGVDGDPQEPSTTADGFALASTLYRACEFWVR

VQAFLNALSLHSARLHVFKMPPRYEKELLLLLYSATRGTGGGGGSGSGGGAAAARGEGSY

TSLTEQGHSLTVAATTSATCTVVKEEKDGLVKKEPVWDVEDGSFAEGDDAVLQSYEGVRE

EGDRSPSSSLPTRQPRQLYKASALLADVLALSRCPEPPSSQGRASPPTPAAVPGLTSDAS

QHLKGICPEFYKMVYARLGVMVARDRLHRFDQSSSVKPPELTVQQRNLQDQLQQQQLAAA

DAAASALPDRMEAGLPTDYVPDDAYYDKLIHQQHDTVEEDEEVYKGEVMDYYLSHPGRYA

SSSATASASSPRSRQHGGNRGHNNNSGDGEAPLMKPHRFCKVKTGFSWTQYNRTHYDSRT

NPPPRTEMWYEFTLFYPALANTKRDMRHIFRIEDAPEGPNDQYCLLVFSVGPPYADVAYR

IRKKQWDPRRGGVRISFDQTGRYKLFFRFTNSNYRR*

>Lp_000021900.1 ribosomal protein S20, putative

MDYPKKNQAAPAEGQTVRLTITSRNPKAVESVTSQLLTRARDEKVTIHGPVRLPTRTLKI

TTRKTPCGNGTNTWDTFELKIYKRIIDLHAPTEQVKRITSFTMEPGVDVSITILDR*

>Lp_000022000.1 Pentatricopeptide repeat domain/PPR repeat, putative

MEKKLALSQLRQSRFVYERGLARQDAERRAKLHKFLDQHKHASQWSTSLAALEDAVRHGV

SPNDAAVCRAIQQCGLAGRLPVAKRLYTDLYRRLGRPRPLAAHVAFMSACADAGDFSEAH

RQLCALRDRDVAQHAKNPRHTPVVNDDLTTEYLRAALCASLARGDSAGVRLPVGEAAGAA

SPASGQGSTHPGASDEADDGEHGADTTGSSPSQRDGASNESRGSGSSGNLATASPWQVAL

DTFLALRKDAQVFRAHNELTPLLLEHATQLACVGGQWQLCLAILRGAAAEQALIPPEAYD

AAIRACFHAQQHTDVLQLMEQLVATRVAPDERSVRLALISSEEVSAMERRSQAVVSSSPS

PTARGSSSGWSMALTLFHALERNGLPLYQQSYEAPLRACANAGRWEEAFQMLDAMRRDHR

PISPPVYAQALAARMEATTTWAELQRLLQVSALADGSNTSVVLYLAALRACMRQGDWKHF

AQLNREMRDRDIPETYDKMRILIEAAYLQGKYHSVLMRFARFENITSFERRRVVKDQLVR

LYEEDFELPLPLLEMVLDSYEKVKGHKDPLVEAAYQAALRHKERLSGQQSLAGDHTAPDE

WMFSQAAREARAPPKFN*

>Lp_000022100.1 Rtf2 RING-finger containing protein, putative

MGGDGQALANKRSLLQKSRVYVTAAELEGTNAGEKQTAKSRSEERWRHCALSLSPLELPA

AFDAKGNVYSKQSVVDYLLRRKEAAAGGELSEAATVHLKKLNDVREVSNDAEDSGTICCP

VTGFTTSSGVHAFVGFWGCGHVVCASTVPRWTDLNSQIDVDCPVCGVQSFYVRLVLDSEA

DAETQLTFLRSHLKKHRKRAREE*

>Lp_000022200.1 hypothetical protein, conserved

MEGSKKFEDAALRGIARHHVWKSRTSDLTVVNLRDPGELLSTREATPTPAVAAAAVAPPE

AVTPTPATTAVPTPAAAAAVADVKRLVGQPPLPLPPQPRPTTGKLESLNALLSKLQLSRP

AAETLAQPRQPQPPVTPPATHPSTEAAAAGVAASVASPPPPPQPP

>Lp_000022300.1 Protein of unknown function (DUF773), putative

MSDQPISIHYQRMEEWLEDRKSVFSYKYKKPYSDLLALAPRLVQLAQYDIPALEKQIKKN

ETVVEECHRVGEEAAKTQQKLSDKRAAMLAEYRIECEGSSVVAAVDRRIDAACVALNAAF

QGYVRAQVAPFKQYYNALLDRTAAGFFGSDAFAAHFPWLQRAHEEADLAPSDGVQHGDAE

VDAEAAGPQIDWGDDDIEDDLAGQAADAAAAAPTVEINWDMNGMATRPKEGEGLPVGDGK

HFSIDLALAKHRVSVLAELQALLCFCHERSSVQTVAASAPTETIYDLLSVSREAEFARMR

ENFRRKDSFVDRIDRFEQQMMVARNRHAAHEAKMHDAEAELAQLRPQYAALLAKTTATRD

SALACLGQMFPDRKILIVGDLNKFIS*

>Lp_000022400.1 ATP synthase, epsilon chain, putative

MFRFCGRRMVARTLPLLAIPHDLPEAFEFLEHKVVDKDIHAPYESMETMRLTVTRQDEFI

FKEAPVKCVTITGVNGENGVYPGHAYEIMQLAPAPLSVEMPDGTVKKYFTSGGFAHINNE

GSCDINCVECIPLADLDLEAAEKALAQQNAALSGAKDDKAKALVEVRIGVLETVIQALKH

M*

>Lp_000022600.1 ribosomal protein L1a, putative

MSVRPSVSVYSASSDSVVGTCPLPAVFTAPIRSDIVQFVHTNMAKNSRQAYAVNRLSGMN

HSAHSWGTGRAVARIPRISGGGTSTSGAGAFGNMCRGGRMFAPTKIFRRWHRKINLHQKR

FAVVSALAASSVPALVMSRGHKIENIPEVPLVVEDSIQGYEKTKEAVAFLKAIAAIDDVN

RVNDSREIRAGRGKMRNRRYVARRGPMLVMPDNKGTRAFRNIFGLDLANVNALNLLHLAP

GGHVGRFVIWTKAAFEQLDKVFGTFTEASAVKKGFTLPVPMITNTDVTRIMQSEEVRRVL

KPKKLQPKKASRYQKPTNGIKNRRLRLRLNPYVKRETAAAKGLRNKANRDARRQAKATRV

AKAKKAATKSAKK*

>Lp_000022700.1 U6 snRNA-associated Sm-like protein LSm4p

MSSSRRPITPIEILRNCRGKEVSIELADGETVNGVVMRTDRAMNIVVKQCTRTGADGEAF

WKSRECFVRGASVKNVRMTESALAAPPVSKRHKGPSHRGPNAHKDKSPAGGKKTSK*

>Lp_000022800.1 Mut7-C RNAse domain containing protein, putative

MRCAVTCSTSPHSVAEEPRFIMDFSMHKVAKYFRLLGYDTLCSRDVPQKELIDKACSDRR

ILVTCSRPLVSAVEVHNRRVRLIRRQTASAAPSRHVVAYDSDGESIYSDDDVEADVELRC

LFTEQWRSRDFHRTIRELIQQAGLTYDPLRVFCRCVTCNELLVPVEKKKVRNRVVDKIYE

LYDEFTECPTCRKVFWGFDGENVINYKSFRTLNLLRTLCIAAGAPVEEERTRLTRLRCFR

SFPRLTKVLVFSYLEDADIENVIEVFPALRDLADEVYESRQTGQPVRKLQLRKDM*

>Lp_000022900.1 RNA-binding protein, putative

MSLAGAAAPSLPSGVTLDGMQMMADGSAGAGIRAPNSAMNPSVNSANAANSTAPPSYEAI

VLDPNGPRSQTNLFVRKLASAVKEKDLKTMFERYGAIMSFALMRDIHTGESLGTAFVRYR

THEEARAAMTGLDGTELYGRPISIQWAKKEHDGTPCGDARRKIHKLFVRNIPLTVTARHL

RQVFSQFGSINNVTLHSDTAPAVPRDAPADAQAGQMRNIAFILFQEDGAAERAVSALHNT

CPFPSCDGIPLMVKLAEDNRDRLGRKQKADPMLNPAAAAPIMIAANGQPATLSTPLSTTI

SPAQLSLDPGAAHVLGATMLPSNIAPGMQFSMAPNGTSAAPMLQATTDANGNTTYVYVTA

PTTAGPRMITPPAPGPYFVQTPAGLQAFSAPMQPAQMPAYYTTTAATSPAGGALPSAMQT

PQQAYFPSMAVSPMFVDAQGNPVTPMQQNIIMPGFYPSFTPQQLLAQQQQLMMQQQMAQS

QSQATQQPQMAQQPQPQPQQTPTQKRLSLPSLAEVATTAGQTPQQPPMTMTPLNAPFMKG

NSSAGSKNLSERSPGAQLLSQGPHPDILSNHSVSPQLSSTASNTNLVPTAGSSVVVTPTD

GGMAVPTNTLQPASGALRTLVPMTAVAVSVSPNSQQQQQQQQQQQQQPAPTNAGDGDDEH

PLFNYDALNMEGGGNSSLTAASSMTMTNDLPVSALHLGIEAEHIAGSGSEVPAAARLQAA

KH*

>Lp_000023000.1 serine/threonine-protein kinase, putative

MSSPTASLLPTRDSVKGSLKLRLREGHNAAPSSSKHDKSINCNAAIPCARGDACQESIAK

RTRTEECLHGAAGGSSDTSSTLSHAGLRAPSSSSPLLSTRSGNFHSETVPKSSRADHADV

SADSVPRPLPPLHRSLPAGLDSELSAGSSAELPFSPTTAALLGGCGSCSNCDSYLTSSQE

RSTPSSPPPLPSLQRIRQHELQASDFQHEEVLGEGSYSLVSLATHRASGLLFALKEIDRT

RLRRLQLEPQLRWEINLQRTLRHPHIVRLYSYFITPSCISLVLEYCRGGTLRQRVRAAPQ

ARLTEPQASRFTRHVAKALAHLHRLGVAHRDLKLENVLLTEDGIAKLADFGWSRPVGSSH

LHQSSTAGQKKEFRSPGEATRRPTESSEKAGEREETTEDEGRRTVCGTLDYLSPEMVSGE

THSYKTDVWSLGVMLAEMLTGVPPFYKESTQQTLHAIRCEAPNLCGSPTHASVPDHDGAD

EALASSSAAEVRQGRPTLSAGALSLIESMLQKDPARRPTINDVLQHPWLQKPPRVTH*

>Lp_000023100.1 RNA polymerase Rpc34 subunit, putative

MTSSSTAAAAASVEQRILTRLSTAAEEAIPYERLKRELNLGVGGGEGVIRQLMEQNRIRL

RRGTNGDNQIYISIVNNINESLSLVLEAVRASGESGIDQTQLLNRVRMPKTELGKALAAL

VSKNLIKEHRSFTNRAKRIYTLFHIDPSAHVTGGTMYCGDDLDTAFVDEWRRELVRFVST

RRMVSFDQVKRHVEAVQSASASGGSSHNNNSGGVGGGIAAGGSAASMGGGAGGTTSMTPL

AVTMIDSTSARPLLSANATNTAPVAAAVAVGSKQLSEVDLRTLVHTLVLDGVLEMLVPNV

QEEVNTPQYQIACGINVMRHFTVQARLRQRGQAGVEAPPRQRVRRDAAELNIDGMTEGTA

GSSSNNNVLPDLENDGIGSAAMEALRQGSLWEPALISQPSAQEAVDGWAYMPALGFPCLG

CPQLERCSVSGRGVVNPKGCAYLRDWLS*

>Lp_000023200.1 carnitine/choline acetyltransferase, putative

MKRIAASELQSNVLKLPRLPIPSVAATVHGYRDALKALWSDEMTAPHLSKLDAFVSSSAP

VLQKHLVELDKTAAEANVAPYTYLEAMMAQSALNSRFPLEVNTNAGFVLREKFPGTDNTQ

AGVASAMTHAIACWIQEVRTKGLLVPEDPAKQFDVSPLLTEFGRSLIPSKEADAIHTTPL

SKLQHVIVLHDGHPYMVRVFDENQRVLDRALIQKAFEFILSITPDVDNPAPVAVLTAGSR

AVWGQAYQELIKTPENAEVLRLFHESIIVVCLDGVKWGDNESLAEASALHGRKEELENRW

FDKHQMIVSEDGQVAFNFEATASDRVHWAKWIGDVLSILKEDGRSGVSTNGVDGADVSAI

VKHLSVTYGKSFVTHIRAARQEALAIVKDTEVHSIHIPYGRKQLSALKVEPDAFVQMCLQ

LAMYQLRNKLCSTTELCSTARFFHGSTELMHTATEEMLELATTLAQHQQNGSSGAAATAA

LDTNGKEALAKLIHATSTRHASLTAAAQRGEGFDRHLMALRHVAQINGDKAALAFFEDDL

FAKTNSAVLSTSEFSKPWLRYYTFGPVNHNGYGLGYVIDDQEVRLSLSAFTNSPATNVSD

LKTALAASCDILYQLLGGSPAAKPAA*

>Lp_000023300.1 hypothetical protein, conserved

MLLRSAWRLCAKSGAAAGTTAAAAQTKLAAAENNSKDSTSDTNPTTPTSATRGSASPVAT

SASTSWMAAAAWASLGSEFKGVSEEKFLKPIPDHFLTPRATTDIQPAEELLSKLVEENTE

RYKGIDVRDPSSMAVYEGERPRWMTMGGQVRAVSEFISGHLCHHISLPEWKDLFDLEYAE

MDLTYWLYVLHVHLVSRRATSIPIEKFNRRREVLEELLVTMFDSWAATSEDIMGRPPLNK

IKYYIRDMYYVTAVNFEEALLHDGPGADLMLMGFLMKFCPLPRPEDVPMYTYYTLVHYIR

FHTALFDRISDEEFAKGNFNFLSPTDSLIFDKYSDIAYDEVIRGWTVEEGNGEAAAASAA

SAAAESDQAASSAPS*

>Lp_000023400.1 Leucine rich repeat, putative

MSSADIASTNDVTSTAAASETSGSFATDAASHVTHALTTDAIVRECIKQGFYRNPICNEK

LYLHNRGFDSIAPTAFEPYTDVKVLWLEGNGFSSLPCGAAYVQVQPPIRADPFADVAEGK

KDNKDAAEVKQQSEHHVDPSRLGTLEPSSAAPPRSLPLPADIPPEKRDAFSSLYPTVRQL

YLHNNLFRVMPDLSRFQRLDSVNLSGNFFSAIVPHCVHWDRKKTTEASEAFPPTGEETEE

SKNVALVPSADDAAARRSAQLEQYRRMADEHAILCEHCPLPEREEEETPAVDRRNTSSQT

HLATTAAAAASATATTADVPIAATRPYVPRQPSVPDPEYRNPCSSLRNLNVAGNRLETFE

DCLGLLSYKALTVLDLSHNSIQDGEALLLILERLRRLQSLKLSGNPLVRTLPRYRKRVLS

RCKQLLYLDDRPVFDEERRLVTAWARAGDDGEEKERCLIRQEKEAAEKKRLDDFRRLIAR

HNDAADGEAPHADYVRAVTTAAALSAAADTAPAITTTITTRESAGRSHRFLRQSHRCAPE

SRRPSSDPDNSPTSSESDDDDDDGSTDMSAGAATVGGDGDRIAMRVQENTNKPARRGASR

EGPTRVLSSNQRIAEEEPPAHKGSGDEDSDGDIFVPGA*

>Lp_000023500.1 aldo/keto reductase, putative

MSAHFPPLKFGIPPIGIGTYELRGDACVTAVRAALQLGYRLIDTAAVYRNEELVGEGITS

SGVPRHELFVVVKIAMKSMGSDETVRGGILDSIRKLRIRYADCVMIHWPGCGGLRPDDAA

GHHAARARCWRIMKALQQEGKVRYLGVSNFLPRHFAELQCFDDNSIEKHHDMVVEMGSSP

SSSGQQADALPTPTPSTSYTLPAVNQVELHPLCVQRDVDMYCRAQHGMVLQQYSPLGKGD

MRLLRHPRLLEVHARYFASAAMSSSETNKQKKTEPEVLDEETADASRVYSVPDLVLMWGL

AQGYCALVRSHQTEHLQSNLAAAKDYFASLPGACVDDSDASRTRPLLTTAQLEVVRNLRL

HMGVEDAEDLHLCWYSSEIA*

>Lp_000023600.1 DnaJ domain containing protein, putative

MSGFTIRRFVLQNITAVAAHCPSVTVASTAVAVMTGVTVPPTSGASASSSSAASAQAHGA

SRVSSWVQTMLTGGAASSAAAKSRFGLVTAEEMEWIRRRRREDAEESRVVLATRPGRSML

RGVGGGALNCVLGFVVSPLVFLAVALERIRLGSGVSALLVGPCYGFVWGGVFFACAQYAA

VQQVFLCGYYSCVAAPLYYCVNRRPLASRKAETSDATAAAAATPRARAWLFNVLSCRFEA

PQGTLHAHHSMLLLYDEPSVLRERAMKRLAQRDRNKHKKDAHYSGRKKRAGAGPDDDDYY

GLLGLSHDATARQIKEAYSQKALHLHPDRNPSPNAAQEFDRVTKAYRVLSNPQKRKKFDL

AGAKGVEDTGVKKREGVRALFGGEELNKLAGDTFLGSFSQRVIDGLDFTADELAVIRQCM

YESCRDELLSKYLAHYDPSAAATSEEKTTNGAAKRTTVTGTAAAKKNGPWKGDAVALQLR

KILNTGLAKEVLFTIGHEYKRVVAYFDMEKAGTSLPSLAVQRGLFYLRVAGPHRWHLQRQ

KIKYLMTVRSHTFKNPEAMVDLAWYTSVQELEATARHVALTVLYDPTLTAAEATTRRDAL

DALADTFILYGEVHKGVSKATVDQLMNSLREYQQQRQREKDSE*

>Lp_000023700.1 hypothetical protein

MSSHNQKQINEAAAEAMRAADDSQQEYPTEEPSVKSAEERRRDEVVAEAAADAMRRADDA

YECDDDEL*

>Lp_000023800.1 ring-box protein, putative

MSSSAVAPSPAASSAGADAVAPTGKPIFTIEEFYPVFFSAWERETGQCSICCNQVEGPCV

VCQSNAEVTSAECGITWGECGHAFHTHCIEKWLKTRRVCPLDNKEWVDRSDWNNSATI*

>Lp_000023900.1 hypothetical protein, conserved

MGRLPKLRFHSHGGSHESPPPPEEKSYKALFQAAVTAVTEQLKRLQPLHRQIAIGTAAVV

GLVVVCKTASHVHTLVQRRRRQQFLRRCEQDGELHFFMLPRSPWSPSLSPACTRVEAYLR

VNGIPYKAVETIDPNGSPSGELPFLVYKGRRVDQLPRIFDFLAAEFTVTMDDALTREERA

IGAALRRTLEYSMERFLYRTVFVDHPTLAVTQIARALHISELRARLAVRGYAKQVQQRLT

ITAYGALISEQYENEFLRDCEALETQIGEKRFLFSDTDVTSYDCAVYALLVPFAYMGQYT

ALSTAYMAVAESTVLMAYIARMSKRFFSDVALQFDAADVETSCTSSELEEEEAEEGGSAT

GSETVHSDIDVSGDERKALERETEQNSKVQSK*

>Lp_000024000.1 Nodulin-like/Major Facilitator Superfamily, putative

MICASTSYAFNLFSGELQHKYNYDSRQMSTINTVGMVFAYFLMPYGFLCDYFGPFPVYVI

ATTYFSLGALLMGLTFQGVVKGSVVRFCVFNALLSLGSQLFDLASMTAIVSIFPTRKGWV

IALLKTLMGLGSAIIAALKTGFFNKTPANYFYFLMALVIVVAICVMWLMRLPSYHLTGYE

QSHLSEEEKARRMVHRVART*

>Lp_000024100.1 hypothetical protein

MPRRIFQALTKTEQLDTTVSALLTVLNGAGSAFGRLSMSFFEMYTQQQRPEKRIPITYAL

FVPTTFLIVSMVLFLVLPGRSLLLAYAVAAIGNGFAASNTILVIRTLYAKDPAKHYNFGI

NALVPAAVLLNRLLYGEWIAVQADKQGRKVCLGRSCVLMPLLVMIGLNITAFFSTIYVHF

SYRNFCRSVLAERRRLQEEAANAEATGEVEQAPKTA*

>Lp_000024200.1 Nodulin-like, putative

MTGQNMHERTPSQIGEAKAEQQYPNEAAAPLSDELGGMDDEAKKAVHQLDTHNIKRLSEF

KRFVSLTAACFAMICASTSYAFNLFSGELQHKYNYDSRQMSTINTVGMVFCYFLLPYGFT

YDYLGPLPLYILACVLAPLGLLLMGLTFQGVVKGSVVRFCVFNALLSLGSQLFDLASMTA

IVSIFPTRKGWVIALLKTLVGLGSAIIAALKTGFFNKTPANYFYFLMALVIVVAICVMWL

MRLPSYHLTGYEQSHLSEEEKARRMVHRGAT*

>Lp_000024300.1 hypothetical protein

MAGEPLEVEGLPTEKRLTTSSSETSETRRREGANEEPFEDNLGRDDLQLNTSEAAPAKHR

EGTVKTDVDFVAPQYQTTFLQSCCTVRLWCIFWTMFCGIGSEFVIIFNARFIFQALTKTE

QLDTTVSALLTVLNGAGSAFGRLSMSFFEMYTQQQRPEKRIPITVAFFVPTAFVIVSMVL

FLVL

>Lp_000024400.1 Paraflagellar rod protein, putative

MLKDKMAQALEMFGPTEDALHQAGIEFVHPAEEVEDGNLNRRSKMVEYRAHLAKQEEVKI

AAEREELKRAKVLQSQQYRGKTVQQITE*

>Lp_000024500.1 hypothetical protein, conserved

MSSREQSPEIVIRDENSAVSTPENKRHLVNATAATKSVESSAARPERTSAVRHIDAENGS

MNAKDTSTPNGIPARTSAFNASVKSDTSNPLPTISQLRPSGSQNRLSGSTSANGSTAAAP

TTATTTSEARTSTTSTASPTQSRLSGAPRANAPSAAAANSPACAATTAARRPPAVPPILL

QQQQQQQQQQQQPVPLRTPLKTPSASLPRAAASSNGSRRTCGSGKSKAFMPKVSTPAPNY

NAYMPDVAKYLKEVVRHRDEAATLKLLPSAQLLEQAVLQEQAAYEQIQQQQALAFMQITH

KKKEAEAKMKEQQTTKAYKEKMDEQKQQHKEDMLQQKTSVAESTKEMEAEIKERLRLIVS

LQPRLNIVQAVMAQIADPASDCSLLADLMQEDKQLQRVLKADARYNKSKTADVDGGAAPY

WWKQCVCASFNVAAIVKQEPCRASVVDANISTKVAADKKVVVVKDADGAVVAWAHTRIGG

NTNALVEAFLAAKPEVAELPAAAYKGVLWPKDESMEVLRAAKGAKSATAAIVPEVITILS

YKEVGSFELMKKDVLAAGEAAS*

>Lp_000024600.1 hypothetical protein, conserved

MDMDPMTRREADKMEGILVDLIKELKLLSMLPEEFSTWVRGGTVVAAMGADKNSYLRLYE

EHAARKAEEVTTEREEQAKIAAAGDHVDHRQGSVPAYAHKGLGKPASSTVSLPSPQGLAA

NSPSSSTSSAGWAELRNEGGGSGVNGLAGSALSPEDEEERQRVIKAACFVHAAAQYGDPI

DSDDNEAVVQVSNFAQLERDLLEAATTTGYTDPNDLQLHHLSTRALVDTLREGGYDAAVE

YFVKRLYKDSDFAKAAKRQRAGAQLSPADAAHDAAQMHVYGCGMVEEGVDHLRELVETLH

RLVHMRNQSTVNEDIHLYQLLHDAVNRDQAGTADVQVLNQRFLDVKAARQREVAELDREI

QQLEEELQYVRKTADVELEAFQRAQETTQNDHRCTLRQQLEAHRSAAEAVARTLEQAQAK

NVDELHALRSQWAKRQAAVSGAIADYDAQTALLETAMRQLNQEAEEDTEKIVQLEAEVQN

LQKQKDEYAWDRTVAEQRQEHACVIRQRLEQDARVIQAYYRAFRARLQAQQALEKGNKRG

KRKRSAKK*

>Lp_000024700.1 trafficking protein particle complex subunit- like protein

MSAPPRTTASPAQLGDAAFDNNAKVSAEFFALTYGALVQQMSEELSQEDDVEQVNQQLYN

MGHRIGARLIEEYSVRSGAAPCRSFTQAAEGVALVGLRMFLGVSAELAQVKDSADTFSIS

FQENPLALFVELPEGTLKEHLWYSNMLCGVITGALSLVGFQAEARFSRDKLRGDAKNEII

LHFKGRERETFQVERN*

>Lp_000024800.1 EMG1/NEP1 methyltransferase, putative

MEYAQKSARLPRSAEERERWKRVIVILEHCPLQTIQTDRGFELLSDRHRSYHARHNQDPA

DWRPDVVHQCLLHLQDSALNRAGMLEVYLRTKKQVCIAVDPRLRVPRNIRLFEKMMVSLL

FKLKVRASTGYLSLLRVVGNPITDHIPAGTRLYRVEKDGDLVDPFRFTAACGYADDAMAE

QKTDRAISLARRRLTSSTSGTLTETTAEAGTAAFAQVQRKAAERRQFQPFAFIIGGMSRG

DVTVDYAQPGEVSSIRLGDRGMSAAAVISTLLHGFEEEWLREDNEAC*

>Lp_000024900.1 OTU-like cysteine protease, putative

MFNSNLDLPVPVTARPIGMRGGGASSSKDGLSPESHHLRGTQNHHTFRSSSASLNTATAA

SSAASPPPAGYSASSSTAFSTAARGPFPIASPSTPLPNGLGGSMRMISSTTGSTAGDGCP

VPMQRPTITIFTEFDDEDGGDDAAQPWMSGTNYLLHTSTGYRDPSAELHGTGLGARQASA

LAQTETTLSPLHYQSLCTRPTNDDNVGMATSFFDHPVAGPTYSSSQPPTMVTEGSGEERN

STSSAVVTSNSSNGMVSSTTSHGPQQANTSFSKLDDSTPSDQHPVRLSATPVVPIAPYVA

EPSCRRRVAEDLERLYGSNSRQPSANAASLKPTNTAAAPQAAAAAGGESHDAARSPTPPP

LPETPKKKRSSTSFFSSSSSSKKAAKAAKEAEAKKKTAAPPTAAERRQSREQVIRVGVQR

LHQRLNELHLVAYHVKNDGNCQFRAISHQLFGNEDYHDIIRSQVVSYMRAARAECFDFYF

ESPAQADAYYDNLAKPGSWGDELSLRAASDCLYVNIHVLSSEERNCYITYRPAMDRAAFA

PSFLIDIAKLRERRRAERLLLLRSHGLQDGASSSMASFDIVSPGLGSSHAGRGGDCSGFG

TVSTMPDLYGRCRAASAIAPPTTITTTTTTATTAANLSAPTLLTPIKEDGTSSAGQRDHS

SSSNGGVAARLCPKLQPMSRVASTFIEEDDDNDAEMDANAIQLALHKKLQQSEIRCSLPL

GVQPCNSYSFGPGVGMGGAHGLGPTSMPLLQPQRTALTNQVNTTYANDGSALLLRPVAAA

VPVPRLVSGDEDVMQGPRANQMERFRAVGAEVQPLGDATQAVNIFTQQMESAPVGAVDHS

VDHMELRQAADNTKADASPAPAAASTPAMASTAQASRRTLLRPQKIAVFADGGSSFSHQR

LEPLAMASSVPFTPSPLTRPSEEPVEMMLLASSIHRGGGRCNSSLHQSFSNVQTVNSYGG

YNGGCGGSMYAAESFNPMQGSFSQSFTGGACAQSLGNLVPVSTHDNGSFMGCNSTDPCHA

HSGDSGSPISYFKFEPRTEPIDIFLSYLYPVHYNSLSVAQQQQ*

>Lp_000025000.1 surface antigen protein, putative

MTSLSYLYLNNNELSSSSRGGVGEDAVPFLCVNHFHGCVPVPGRCWSPSLSVYAALKPEP

GPLSNRCTSGSGSGDSLEGMAMAEEGTSPLKFLRSFAALNPSLASIWTGNYYCDWAYVSC

SSYSPSLDFADYRYPAYSGTLVLPELGDDVNGSAVIFTAIKVRSMGGRVTGTLPASWGRL

TGLETLYLDGNNLTGTLPSAWGGMTKLDTLYLEKNALSGSLPSEWGSMAKLRSLDLNSNL

LSGTLPVSWASMTSLSYLYLNNNTLSGTIPGEWAGMTGLSSVYLKDNHFCGCLPAKWRSS

YSPSVSADEALKSDRCTVINRCTTGGENGDSLEGMTDEEVSTLKFLRGFIALNPSLASIW

TSNYYCDWAYVSCSSYSPSLDFSPYR*

>Lp_000025100.1 hypothetical protein, conserved

MFTVSRKLRRTPLNLHNGLLPALRLSPFLSQRGATPNHTLVTSIPRHSSSNAALSCPSRN

LFVSSIRWQQQQQQGQRPPSPSSSSSELPSIEPVLVELRSIVTSRYPLGELYRALSPESR

KILVTHKLPLEELLLHLPNNFVLFRSRLTVGRAGRNASNSGGRAAGVLVRPPYLLPPGVQ

PLRLPPDAKPLPALAQIFKTPDASTTGSTATSNADVATTDTVTNSRGTHPSNVGTGFVDS

YCTTLDRIQEVLTYIPNEWTDFSKLPISKEVKMRCMEYPSVRPSVFFLKHPKYFDIRTQS

SKKHCFEVRRSLALQKQLQGVVPPPK*

>Lp_000025200.1 hypothetical protein

MNWVDGQNTSLRLVVAPSGHVSFTDDTSQRWTLFFAEDTQLTRFLACVGVAFYSLYGAPA

TTVFFQNLSLPSSSLQLEAADKATVAFT

>Lp_000025300.1 MORN repeat, putative

MTSSPSTDFLGNALPADFADVAVASKRTGKGYLQYANGVIYEGEWLNGERHGLGVCFYPS

GNIFVGRFRSGLMEGNGTMFFATGECFSGEFRHSTIYKGVYSSRGREISGVWQDGIRVSE

MPAKAPEVLQRARAVLFSTIVEQVNEYLWAAASEPTPLSLPSRPQETALLRPPMDIPTSA

DDDANNAAINNHIDTMNAAMSNNNNNSSSSNMRHADSSPSLRAQRMPV

>Lp_000025400.1 2,4-dihydroxyhept-2-ene-1,7-dioic acid aldolase, putative

MSAGGAEFKAELRAGKPKFGVFLNSASPLLAGQFSHSGYDWLLIDAQHSPVDSLTVAQMV

AAVRVGHAKVMVRVSSTRDRAGIQSSLDSGADGVLIPYVNNAKELEEAVSCCYYPTKGTR

SVYQPQQCMNAAGLLGYVPEANKNVVVAFQVETASCIENLEEIMAVKGVDIAFLGQNDLC

MSMGLYDGRYVFPQMYFSPELQGATEKLIATAKKNNVILGLFLFGTDRVGEFLEKGFTFI

SIGCELHHAMTQAATHVKALKEISAAKGKPWTNQPSALV*

>Lp_000025600.1 protein kinase, putative

MESVVKLNRKLGVGGRGVVYEGFDHAKGRFVAVKEIAYMETSMVGEEDTALQEILSELAC

MREAQHPNLVQYYGARRSAVGVQIIMEYVSGGSLDYVLTRCGPLHEAVARAYTRDVLEAL

RYLHEVLHVCHRDVKPANILITPDGRCKLADFGVAKHVEETTPTSSPSCGAREGYLRTAV

GTPWYMAPEVINGGVDDDDDGDDANVCAGGGTAAAEKAHVQPACVEGSFGDRSNAEQTPC

HNADSLASSPPPLYASTSYYNPLKHIIRKGRLGRGSIGYTTRADIWSVGVTVYEMITGTR

PFGADLHTPSAVMFHIANCATAPPQLPPELQVSPTLQSFLDLCFVYDKDLRATASELLGH

PWLRDSASDAAAAAGRQPPLRRLSDSNACGKDSTSSHAHSSTDRDPSRPPPTPPPASSLS

VRQSQQQHPSVFDGVPLLDAMDLPAYPSAAHTVATTQDSSSGSSSKNSAKETEGGAAVVG

YQPARSSGTRLSNAGLYPHSFLPPSPASTTVAEASFVSPAEQAEASARYAAARQRIGHTR

SSAPCTPAVQPSSGSSSSGGLQTKYGAFVDVLSHP*

>Lp_000025700.1 hypothetical protein, conserved

MLRFAAKALQWQPAKVSGWGATSTVITAAAASTAPVPPAGIPAPVPTGIPAYGGAEGPLY

LTTNFARRASQFKKRMRGKSQYAFGREEAQRFLDAEVPKADVSAWMSAGNNAIALAQLLQ

FFEIQPEASVASLLLSQLHRHTEDVSINPYFRLQAALCLLSIRPEENYERVFQVVGQVLQ

QLQRTPAPLPDITATQQDEDARKATLRSVGALWDLLAVCAKILQRCGKRAPPTMNDDLAK

TIMIHIQSTTPNILGQLQHTLLRIYAWMVRAERPESNQVLYILVQHTGEFARDNFATLTL

ACVRHNQLVPLPIELVERLTRAAFQYCTNVNAKEASNILASLAKLLQTLSPNVNGVTTTD

LNRIQDYYSALLEDYSARIVRFMKPGDVLYWSNAEDVSSIVFAFELGGHMRHRIVFEAYA

AYVQHCVEAFEPPQLALATGILRRAQLLTPPLAARLSERIEVVLGELRLAELSHICATFA

VLPMPRPTWWEEAKAVALRLYVPDASGVVRLNLAIAFPDEPNLVDTVDYSQITSKQLVDV

LPITQGSAQFEEPVVSTLCTRLAAPGERFTSDDLRLVLSCGRPALLQAAQEHLRRAFAQP

QWNTDTLYSLPLVCDPHHPERNQEMFSVAKALAAAQAASIGALQFVSLVELLMTSFGDSD

AAIRQYVQVGGDDLVRAAKVPMTAVVRYLSVVRRYPSIVLSTEWLRSFTDCVERFPFAQK

SDLEDLLISLRSLYEDVAKTPALQTLLSLLVEKSYVVLTEADEQTARITVLLVYLQSGMT

LPLLTPQHPTLAKVMSNDAAYSPQVRRALAMMPLPKAAPAEERRGRFVLKRLDKSPARKD

SSAYALDLNTSDPFEIPLDAETASITEPAQQPRQQQQQQ

>Lp_000025800.1 iron superoxide dismutase, putative

MPFAVQPLPWAYDALASKGISKEQVTFHYDKHHKGYATKLNAAAEANPDLAKKSLLEIIK

TVKGPAFNSAAQIFNHDFYWRCMSAHGGGEPKGKIADAINESFGSFAKFKEEFTAAANGH

FGSGWAWLVKDTTSGKLKVYQSHDANCPLTEENLKPILTCDVWEHAYYIDYRNDRAAYVN

AWWNVVNWEFANKCYESSSGSSCTNSNL*

>Lp_000025900.1 iron superoxide dismutase, putative

MPFAVQPLPWAYDALASKGISKEQVTFHYDKHHKGYATKLNAAAEANPDLAKKSLLEIIK

TVKGPAFNSAAQIFNHDFYWRCMSAHGGGEPKGKIADAINESFGSFAKFKEEFTAAANGH

FGSGWAWLVKDTTSGKLKVYQSHDANCPLTEEHLKPILTCDVWEHAYYIDYKNDRAAYVN

AWWNVVNWEFASEQL*

>Lp_000026000.1 protein kinase, putative

MGSGNAKGQIAVRSKGATEGECTIISLKSNGGDCGVGTNDGGNGSQGACPTSSRGSPKLN

PHHNNSVSGTATHVPELTLPRVAAANRGGQNREVAAVNGNGGGGGGAMGSPLNLSRQENN

NHSASSPLGEGSPIPLRGLANHVGYICLTDIIGTETSGSKYIKMRSIGKGAYGEAYIVKR

NPAYDAAAHPAAEAQIESNALANPTQPNGLYVAKIMDLRAMQTQDRQYAQTEIMCLAHTH

HFAIIRYYEHYVLDSDDETVIIVTEFADHGDLRRNLYSVSSPPPAFLDTDGASTSHAGPT

TAATDAGEAGASRMLLSEREAGMYFVQLLLALHHIHGRRMIHRDIKSANLLLTSRGFLKL

GDFGFSQKYESTVSSETIAGTFLGTPYYLSPEMWKGKRYGKKADIWAAGVVLYEMLMGGR

RPFEAGGLPELRSCVLEQEFIPPVAPPASVIAESRHGDGTAAPFSSDIRELLVVIFQKEP

EKRPSAEELLHRPLMQHCLFMFEKHVQSLVSADAAKVAADPSVDRQQLFFPDPADQTLVL

QGIAEGKQVIENEAKRTISESTVPRYEGVVYKDNHNGIWKERYLLLEGNTLTISLSKGKE

AVHGGERSKRVPLSSIKSVSPCEVEDAHVRVAASASDGYKPPFAFAVAMMSSNSIVFGVA

SAAELDQWMQNLMRALQID*

>Lp_000026100.1 hypothetical protein, conserved

MSLARRARERNAQPKGETYSRADEALWDDEYLLKLFNEQLENSGAVSEAAHSNDPQSNAS

RISEAETGEDDALSASSGSRSSTSSRSSMDHKRKRESKTTGAAPVAASSAPSNLHLPEDI

QALVQSFYNAGFEAGRYVGRAEAKEGKSRKRHR*

>Lp_000026200.1 hypothetical protein, conserved

MARGNPYARGGDVMKTRSEKRKEKQAKLAKTNRVIRLKRRLRRIATKKFAGQEDNHMTAL

MESYILRRKAERAAERAKKEEGEHATRGKVVAAGAGMSEEEEDEGDFSGEEREEDSSSSN

SEDAFSLGSGGDDSAFEMDDEDGSEEEDEKDIATSRQRAAEAKRQRRGSGRGAHRGGRGG

SSWNAKREGAAMPKHQGGERSRGGRGSCRGRGGRGGQREVSASSYQQRSRKMSTRPLY*

>Lp_000026300.1 hypothetical protein, conserved

MSSPLSAPTASTETYCGLPVYALRDVLSQLPLPPPFDAASISSKWRCVLAPSAEMSATVS

TAADLLLSEEALMPYLEAGIPVRGHVERAARVHAGCGQDSVEWLTTNTWNAFPKPQIALR

RGWGSPGTDMQESSFHKMIGSFAEVFHHAPEVEVRLSEQEMLEAAAAAKEEGDILPLKNA

PTTDSGAAGSSNTEDEVLDLQLFHRDEGCPVMPILGEQLVWPSYVDDGAVCDTATWVSQR

GMVRHWHLNDSGEFAMQAALPLRAPSSLPPPPDGVATEAAWLHDGLTPVVKQIRNLMCPA

MSAATTATTQDATVTVTEESAVKPKNSGSHLPAMVTIFAPKGGYDWVLHDDESTMLGKVV

ALDLFATPDEALPCEAVVLPVLTVAVLESGAAPLIIPPNLAQLSIALRDCVVAEQRRVSN

LWLDDVSYFLHRCARWLTSPIIYAYLQQDLQDDAFLAGQVIPFLIRVFEEHGEDTAYHAA

VRRRVVCSLFALATNEKHYGASEATRRSLLTLLRGGNAGMRAVLQSAPYRTSGIAVNAAT

TTTAAPSAADAGVAPTLEAWLTLYWNMNWCWPKPGCVLRTPKVVHPLGSKLSSVQQTTYF

IPVVYPPSTCSPVYGSAEETLERTVQQYFDMKQVESRWKDLIAYLRTRKQPADDLLDELF

*

>Lp_000026400.1 hypothetical protein, conserved

MPSLADEADAEFQRLRQEDNPYSVIKLRTSDYNFLTSEEPAIVPIHIIEARRQEDRRHGV

VDASATLQDFLKSIGVEVADTRDLTSGYSSLQVEESTRAYYGNPEAEAYRAYRMQMLRFA

DDEQWLKEKTFLEGQMAYDRERLRYIETKRQKPEPGPIEPVTNYKKAFYEQKLWKTERKS

*

>Lp_000026500.1 Phosphatidylethanolamine-binding protein, putative

MRRFARCTTAASAAGGVLQCATAAFHMPGWKTQSSTKDARSREQREADRKARQIEKLPRP

EALHDFQYPYEKTILSDSMFEYPVYESELDTPHLFNLTPPPDFFIYWNAADFERTSYPPV

PKLDHRMLVPSTHSPRWLEHRARMLRYLRKHEICPAFVPHVKADVNLSVVFPGQYSTRAR

LTDDDGEPIPTPPPETQLTPRNFWFTAHCGNYIELTDLQQPPSIFFFEEDNTATASPRDG

AAPTAMEAEPVSAAPNYYTLVIVSPDYPYRVPLSHDRQHADRGFFLNYMVSNLSAQSAAQ

VNGGAVVDTQGLQKKGDVVIPYVAPLPTEDAGTTRHLCLLFKQVGHVANVRTLSPEDERQ

HFQLAARSNFRLHATAMTTPEAAAEECRGDKNQNNAATGPAACLASLRAVEEAIPADPSA

VTFFTTKWDIQVQEFYEKVGLPEPAAPVDEEVEALLEYHATPPQKLRVRARHRADGSVNT

GEDPHFWGQTAPTRMLDGSMQRGSGWSRRTAMGRNGVPVVCPH*

>Lp_000026600.1 hypothetical protein, conserved

MDFETDATPAELLAADGTLTASTEETPFYNPAMTTPHSLLHELPPRPPSPYKSMQHNTHR

TSPDKCSVASSFFRGRRNLYLGAAAAAFTGAGSAASTRSMPSPFSGCGDDFVYQPTQRGD

SAVIHVDAKTHDSVMQQYLVYEAPRERLVEYEENERSALLAGEEVAFTSIVIRVLQARAE

LLAGDEAAEIVVAAALQRAEARREMLQVIEVQVSTRLREQRLGEMLEVQFRALLPAHAAG

CRRIEADEREDLRRLFLWHKDHRPLGPGCFIKGPEEDYRLRYASRGTSAATARSPGGRSL

VMRGQRSGLSSASSSRAYAAQLRQQKAASATPNGALGELVLFDQFGERASGTMSSTQDGT

LGPGEEAVFAEEGRLRLLAEKTRRHDELRERRRFTDLAEDQADARQYVLAEEALCRMHLT

CAAVDDLYTAKEATRVRKMTEASEAGARAELEAQRSALKERLRREHGFPTNAQEEEAEKD

KVGEEACREAPLDGAAAAVATSVAQTPDPGQYSRKKAEAVQASSARTNSVSTVVDSSIPT

DASLGLLPDVENENGIDYVDRSTPLAYLRLIADDTEGSTMCHQLTTITSSKAAFDEHDEL

*

>Lp_000026700.1 pumilio/PUF RNA binding protein 7, putative

MPGMKLDFLKNPSQRVATGKSLPLKQVEAIVLYGKPAQRAKVVQKLLHAVYGLSLNKTTH

HILLTLLDHCDNMDRVNMLYNVRRKLRDLANSPVGNTVVQRMLEKVPVRQKKEIAEAFVL

NVDEDEFKRLCEHQFGNHVAQKLMEVPECVEMVRAHFLPHLAALSLHPYGMRVVAAYVDN

VAEGCTQVIDTLFPSHADADPDGDEAAVAASDKLDQSILALFRSAEESMVLTALLRHART

PVDVKDAIYAHLAEFVDVYLDTAAKVSDDSACATGKKDEDDGAFAMPDFGAGASMGRPSG

GTKTLENVHVYCAALEHGDDVQRAELWAALSSTPKVLFTITHVKGAVQVGVAAVRYVEAA

RKKLLHAMYQSASPSSPAKTSSLTPEKVQSPKKSRKDSAEAENGKKDSLVEVASDPVRSV

LLRAFIEVARELLPDKDTQDLTSQAVTLAQNAVSSPVLQKLVETDLSGESAGAMLQVLLP

HPSFEELILHQSASFLLQSILQYAPEGVRAPLVAALSAYYAAHLHDALSFAQGSRVMQKL

LAYAPDATVVDTVNQLISAATKEEEAEEAKKAAGNSDDDSEEEDSVVPEEGERKLSRKEQ

RAINQAKHYKVTSHALVSYALHNHACYVVQALLRETRARQLERERKLLMNELKPHVFELA

VSPWAGRVVLDAMLATGSAQLADAMKNVAFLRAEEWLSDVSEDRRKRGNGVDPTLRNILK

RQREEIEKGIDTKTPVAGTGGVSGSAAPPLKKKKLFRSMKK*

>Lp_000026900.1 coatomer subunit epsilon

MTDVLFDVRNALAVGNFHQAIADGSTARSMSTKPADVASFNAEKNAVVALGQIGLGQGDA

VISQLRSESDPLLVSVRTWAELMCALRDNDVLSDAVAAVVGRLQADAEKVSADTIYKAVF

AATASLYRRDVIGALTLAKRWLGELPKPDGALATRRTVELHAISVEALLRLNRPDEAANE

VKRMEQVDSESIMTLLCAGIVALHQAALNVVPQQYEAAVASFKEVQMRCGQSAMVSNLMA

LAQLGLQDYDAAERSLLDALAVRSNDEATLANLAAVSAHKSNTTDGAERYIQQAAGLRGP

WAEAYLMKERSLDDAIEAFKLEA*

>Lp_000027000.1 DNA polymerase alpha/epsilon subunit B, putative

MQVEGDWRVAANLYLLLGSEHPPARVEKPLLTPQERAHGCPSIVRVEARNTANFRSSSYC

GLQDKSTLTVRKNVVAYYRDMLQRIQLWHSTVNKKETAYADQSPIVKPEPRDDDGEAEEE

GEKAVVDDLSPSAHTPSTAVATPLFRAVGILTQVQETSTASTAAAQQAPWEFYVVCDLDD

DGCYVRALQAMTEQSTTTGPFMLLQETNNDNNNSSARGRLCDKASVANVGAAGEETTSTK

AAADEEAAAQDGTRLSLYTRQVSQFTGLYPGMAVGIIGEPFQRTARGVLTGVLVRDFVLP

ARPMLPWRVDRPLPSSAVIATSYPHSSESGGGARIHFCSGPFPRRDVAGLLRTVTQQALH

RGADVLIIGGPFIPPFTNEFERGLLPSLGATFNEMLDTFVDTLEETLKNYYATRPLLPHM

KVLLVSHRGDVTQVPVLPTTMYAIADTEDILVRSNPCRLSVNGVHVSVCNEDVVGAMRER

MVERWPTAEGSLRRVVEALVDGRLYTPLYHFPVTSIDMKHLPQLRMDYVPPRNELLDIEP

TTTTASATATTVAVSAHGGATQKRLRGDNDDEDTNNGGDRTVQSSSARSWDALLQLTFRG

RSNSNGDAALKSKVKQTKAEPEEEEEEERSGAAHLSRAALSVSAQVDGSRNGNGSTSSSN

GPGGSVVKLESSLTAAVVSNEEFMPHIMFLPSTRPHFAVVTHQSEEVDGPDLDNTASATG

VLVVNQEVWSTRSSPKFQLRVAEVTIPNTELVLHRGATAANGVTCGVLHIYAA*

>Lp_000027100.1 Midasin, putative

MQLLAEYGGYEDVTVNFAEAALLASMAARASCTTDTRIVAALAHSTGARALFQRDDALRL

LQFPSPLVHSLPMPPTFHTSALLRHRVVYLDSCLSLLVGTAPQGTSRSFVEGVFAQYRTL

FHQIEEWEAQERKADEVAVLYKEKALVIEGDDEKLLRKLKELFPSYEKEFSDEALEADNS

AAETSGKKLEEQVEGRRAKFARILFQERDGYYLRHLVEAHRTFYWRLVSSRVPGPAGGGG

GGAAASASSSSSHHDLLSAFRNRFDALAAELHAYPPSASAVGSTRDGAQVPEEQLLLNGF

AARASILRGEMSPTAVSLEERRESGFNIFLDTDVYELSRFSKPLHALLGAVQQLAVTYPD

TPSLQRCLRIGHKIAGLPAMSTPLIKVMAGCEVLLRECYEWERNASHEVSLMRHMTELSS

FVLRWRRLELHCWSHVFQAKRAEFELHAARQWFTLYDVLLRDAEPAESEDGGAGSDGRSG

SSEDDSDDVSRMLRRCRTAFQHASQFMWDSTFGDYAARLRLLQGFGFQLLAADGAQAPLA

NTVLHVCDFFAQFEPFILKQCRNAVQPIEEDIAEFAKIMRWEDANYYAVRATAEKSHMKM

ARVLGSLEEALRTPVLPAITAEEQRCEDDASLGFAVAGAEKQDEKKKKKEKSAKAEEAKP

QRTKRQRNGKAVPAASSASQESSSAAAADDAASATAGNARLAKAHEFLRDAAAEIVENVT

ALQQPKTPNQMKVRALKTLFSRLAEAGVSHTHVEQVGAWEVVFASTEALLGCREVHADQT

IAAASLAAAASEQYRFARWLQRLREVERRPHHDLSAAQAKRGTGTAESLFAAAVRAASLL

CQLFHLHEQLRVLQMSVQNAAVVSDAADADDTSAAAAPVASRALLDDTVELACLAQSVCG

GLTWMVQQKLVPSDAAEAIGLVHELTSVSNELWGLCIVHEPMRRSAVNVVPPGIAAAVFD

ALHGLLGVASRLQACATSNVAAAARPVVEAVTAVLRTHEQAGSTLERPANEMKRRRTEEV

PASWGLRLSRALTEVDAIVAAGEDAVAIVDDAMPNPAASKSEVEETEEAKPKSSDEDEDG

DVAADASAVYARYAQYGATMQASLQQLLPLLVELLATKDVTTATSTAATPAAHTLTPAEK

TELLPRLSVCLREVARARQAWSAVLEAQCHLGLIVARLFAVLLKKGFCKTDDDEEPEDGE

GDGEGGTQQTGTGMDDGEGEKDVTDQIENEDQLMNMKDKEEQPEKEQQQGEKDEDEEDNA

ADVETDFNGQKEQRDESDAEGDDDGEESDKEMGDVEEGTEQERKKSKKDAGDADDMNEDG

GDAAEEVPEDDLAEEEGDKDDDDNADETGGFEDKAEEIREAEAERDGEHDMVGDDARDEM

DESNNDDGSESGDSVERSDAEGEEREESAVDEELPSDADEEPEEGDDGSEHHADDDATTD

ESRSEFGEEEVEQRGDEDGEGASDVEDASDAADENVFEGGRQDDREGEEATENQDKDEKK

DKSSKSHPEHSTNEEEQPNAEQEQDDAGQNWKKQQEQNDQAQRRDNTEQTRSQHNPYRAV

KEALQRHQRQMQQLNLSKNVEVKDEEEEPAKEKPPKSDEADGPEDMEEFDWDENGDREGL

AAVEQADMKPTEASDEEVDGERTDSCDDGESDAEDGETPQDTRRKRHQNNKDDLTKLEKK

DAVEKAVKKKSKKVRTTVRDDEDSAAEDEEDDAAAAENTDAATDANAKLERGRQLWLEQE

TAVQGLSQQLCEQLRLILAPTLADKLQGDYKTGKRLNMKRIIPYIASQFKKDRIWLRRTK

PNKRTYQILVALDDSLSMQCNNAGVLSCRAVALIAKALQQLEVGEFGVACFGKETAMVHP

LEEPFLADSGPRLFSEITFEQKSTDMKKMLETTLTYLDDARDRMHGQTRSTTQQLQQIMF

VISDGQITEDRAALRQLMVRAEENHQMVVLVLLDINAAEGSAGDTSAAATAAAAPLTKNS

KSLQGLSTAEQLRRLKADRERRMQAVKTNSRSVLDMQIVSFSGKSVVRRPYMEDFPFPYY

LIIRDIQNLPEIIADAMRQWFELLNAGV*

>Lp_000027200.1 deoxyhypusine synthase, putative

MVVEAEATTATSQSLRRRGTRGRRQAAHKSTSTTTSLLSPASSASSAAAVHCVDFQSLVQ

TSQENALYAVLSSLPTVGLQATQIGRGRHIVQQVLHHRAQGDRVFLAYTSNLISCGLRDT

FAYLARERLVDCFISSAGGIEEDVIKCGGSTVIGRFNLDGRTLRRRGINRIGNLLVPNDN

YCWFEDFFMPVLATLHEAQRVSRWQTHTRPSDFIEAMGAAMEKEHPETCSSSLLYWCYKN

GIPVFSPAFTDGSMGDMIYFYNFSKKGLVVDPLKDVPRLRALAAADGSGRNTAIVLGGGL

PKHHLLRNVPMDAVVMVTTGQEADGCVSSSVLADDLACGLLKEGAEVVRVQGDATVVFPL

MMVAETEPSA*

>Lp_000027300.1 transmembrane protein, putative

MAAFLRAACVLLALFLVATTAQAASQSYVLERRVGSDAEWVNVGSFAISRVSPQAPARVS

NQQLGEQSMSMEQREQFAAADLIYYRAYPYRQGSGAPAHPVTVVFTPCSLIRGFDAIDSK

TVVLNENIRVVPGPNTTLLGLQMSSETNFFHSKMMNGDECDRSVVQKLFPTVRLRMKLGL

VHPVTASRKVNYEDLKVLIASEAERQGKKPKTQVRQVRNADGELVEEEVPVDDRSFLQKY

WMYLVLPIVVSVIQNLKG*

>Lp_000027500.1 hypothetical protein, conserved

MSLTSYNRVFLASWLDKKMEWRFVYEHRYFILDGTRLSYRLEEHGAEKRFGTLTSFEPWR

ETAKDETAKGYCFTVWLLEGGSWYLRAATKEIYDSWFRAIVTVLIPSTSPSAAPSEAGGG

QHSRVASSLTETSALPTGHTSSQQLMGEGIARSQTPARKTAAERDLDVTERNTPARDDAA

ANSSEDTSSLTAAALASISHRAGTPVSAQDAVLLSSMVEKERAWRGQWRPRYMELREGRL

LIIRSSAASSSERHRFVVEAVDTSPLASQATWLLVRTSTGDRFWVRFGSVEERQRWLTVC

GRLLRAECSWHWSPLAEDDAGRSWRLRQVTSGSYDDLHVHYHCAAMVPSFRDDILCPSLF

IFGGSDRWCRPFFSPQARAFIPHADAAYTNGRLAALELAGPHLVRPLQPAPFKEAPHALV

RPLPRYGATLTCLPVVAPVNKTEDGKGGADAAAVELLAARVVLLGGRTGGGYQPPATELW

NVWRSKAACAPGVELRWGRQDLPSYELPHLAFHDAVYVPWGPHGAVEKAQPHDGFLLVTG

GMDVECRCRAECYAVVWNCDNSSRNAADVDAEERAYTTLPTAYAFGQLPGPRAYHRMAVL

EDGTVVLVGGRGVENASVSPSVLTLAPEVWHHAAAAWGGPTFSHHSHHIDPAVSDPQVHE

VETASHQSENWCSISSEEDKEEADAPTSQWLRAYFDDSPLPSGSLAATSSAQQLVKGLPS

EMGDVAVAATGSGGRVVVMGQATQPQQEVKLFLLDFEAVNTDAKKISAGQQFPPGTRRVR

CREVLLFVGAVPNRVVGITLHIFNGYLYVIGGCTVDKSKADSYEVCGPLRILLE*

>Lp_000027800.1 pseudouridine synthase A-like protein, putative

MSKDSSAAAAASSSSLPLAPRTLGNNKKKKMERAFLFDRYPSRKIALRLAYHGHVHDGLA

KQKETDNTVEGIVCDALRRVRLIPEDGPHNFGRCGRTDKGVSALGNAFSLTARASCSADA

SPQLPPLDYCNTLNNVLPPTIRIVGCAHVDDDFDARFSCVNRTYRYYFCHRGLNLAAMQE

AATYFVGTHNFRNFCKLDVVNVSSFMRTVLSAGIHRSNELPELISYFEIQANSFLYHQIR

CTMEVLFLVGRGLESPSVVATLLERGDCKPTYPLADGTPLVLWDCGFDNVQWQLSRRAFL

AVEQELQDISIALMIRATAAAAMRSQLFSWYEDAKDIFASKDDRDDKAGADARDKEQPSS

YAVVPHSAAFRRLDGWAITGCDWTEAGTQVQMKARKRDLLYYLRCDSATESRPAPATASS

PSPVGPDVKAHTPQNYVPLLQRETERTFDEEVCGLSDKKRARYEVNEAKKAAAAAQHTAG

DDDAEATDE*

>Lp_000027900.1 Adaptin N terminal region/HEAT-like repeat, putative

MSKFYSAETQQGHSSHFIQYIRRISEASSKQEEDQIVEEDLKDLKTTLTSSTAKDEELLK

EYAVRAYYAELMGHSAEFAYIHCINLSCHHKLNFKRTGYLATSLMVNPESELMYLIVSSI

QRDLKSPNYLEVSAALTAAAQTLRPELMTVVQGDLPGLMTHREPLVRRKVVEAMHAFYIR

SDAAVGDVAAFRQALCDRDPSVMDAATRLLHEVIQRSPDAHRDLLDSFILVLSQVIERRL

PRTYEFHRTPAPWLQIRVIQILTILIGNDPQRAQKAAHVLEEAMQRADNGKLIGFAIICE

LVRTAATIPSQYALLSLAAEAVSGLITARNPNLRCAGIQALSYMVRVRPELAAQHQEVVM

SCLEDADETIRRKTIWLLFAICDSDNIDPIARRLIRFLSKVSDPFLKRSTTRGLCRLMEQ

YATNPWWYIHTMNSVLAVAAECVLPATIQRMLKLIAEGQGVDETADTNFRIRCVEAYFTI

TGGSGSGGDGDDAAMKTAVSDAAGIGSPNVTDSSIIAVPEVLLRIGAWVMGEYGFLTNHI

SDETLLDRLYDVMERAEEGETRCWVLMAIMKVSTQAQRQRNSPAAGGNGSSGGALVVTED

DADDVVAQYKESRCIPLQQRCYEFAALRKQPELLQRVLPKDGFCEAMDADPELNFLDGFI

ETAVARGAQRYRQPADLAALRQQRATASALHAETGLRTAAYAAARPTDIRPVWSTSAATT

DEGSAASGAPAPAAALPNAGLAQSVLQSSERLMLQPTATKRWGVQNLREMEAEEEEVHLQ

MAAAAAVGGGGVVWGNDTVINSSAEVPPVSSLPAGGTGGAFTEQSPRSALVSAGFGRDRG

STGSAEGKPVSAKNAKFVRSIFGSGRRKGGDEKALSSATAFRIVSPAAPQSVVKTVSTHA

SPVASAEPTQPASPQANMMMDSLFATEAISTPLAGRGAAPSADWFTSTPNNDSGSPLSAS

AKPAPLSLLQPPAPSFNTEQFGHLWQAMGAANERKEQVQCKDLARYAACDGFLQQHLLQS

CSMRVVQIIGKELIAAAAQNTAESGGSGAAAAAKAYVCWLPSV*

>Lp_000028000.1 phosphopantothenoylcysteine decarboxylase, putative

MATNATRKANIPSEEEYPSVHLLLLITGSVAAVKTGLLLDQLSTERCNIRIAATKAAFHF

LRRAQLSKTGIPFQSIITDEQEWSEWQAMNDSVVHIELRRWADLVVIVPLNANTLAKVAT

GICDNLVLSVMRAWEVRTKPVIVCPAMNTAMWTHPLTAAQLSTLETLYREPVSQEESTTP

DEAKATTIAAQSADEKATVNAAGMDSANPVGDAAPAAHLPATLKEAMFQIVGPIVKPLAC

GDVGIGGMATVEEIAAHIRHTMELIRDEKRRHFREAREKAQSTTPAAAVARPAPPASVEA

EKPSPAAETPAATTAA*

>Lp_000028200.1 SET domain containing protein, putative

MKPAARAPLFTAVSFCAPLHTAKRSFAKVSSIHGKIESPPGGFVTEDIKIDMNASHGGAF

ARGVFAGREIGYGREIMNIPAYCMYISENERQPLREQVLILTKEIFTKVVCGTAAEQEYV

KHRILSLMSGGFSYFTRERDVFDFAEEVRAPGAQGAIINGSNCLLSGQFSSYDLQKLPLI

IEFNRFEVDYRGRRGICLFPEASYLNHSCEPNVELAITYNSAKNNFYLSARAVRPIREGE

ELFINYMPGNNLPLSRLALAMKKRWGFECSCVKCKSRAVGAVTVIFVALLIPIIVYIRRF

IVERTKEKHRSL*

>Lp_000028300.1 hypothetical protein, conserved

MLDQWKMDLESEDHRTRRDMTEKFIARNIKGETERQVTSSSTSSAVFVSDCTCIMGATAA

HPSYLCSGLSNGSVCLLNTSNLSKIYLFEVDRDPRSAPRGGASAAQQLVAVATHAIVPGG

TVSGAAATSASGAATGQLLLCMTLDNVVCVCPMDMSTVLERKKVSRGDVAKVKPYPSTKS

ATVLYSSVQFNTKGNRIVAVLGLSQEAASSGVRHCVAVLSTPDDAATLQSRFSCEREWHT

LVAPMSPAAAEAPPPLELCYCGWWSVDVLVCLWSNSCLQLLRAADLVVVGECRLLRHCAA

DAVTNATVSRPSGTDVAESATLNIVALVLRHNVAATFYVTSKRAAEAVEAPGLKRARSET

APSLSFSVCPTLQSYCSEEIPIREMHLVRSFSWTLMLLLESGALVFLDVETMRLSVHRAV

HRQRMPDAGRYHTSASLSLPRHFFCVVHGRPFTAAIVEHNAAILLKAQ*

>Lp_000028400.1 hypothetical protein, conserved

MFRSSLRRGVHRVGYTHPHQLPVPCAQRWDLRLARARIFQEYIEEKAPGAWQLEDERHMS

PEFHTFTGYPMRNMRPGYGQNLPEFLMKKRLPNNTHYELFARRDIPNEDNAMYGKLLYDT

TMHGTSLPSMYRMHKDINKAQRNDRKLSGNRFKVLNSSGAKNPPSGFEPIPDASEEEDD*

>Lp_000028500.1 proteasome alpha 7 subunit, putative

MAGTGSGHDQSTDVFSAEGRVFQVEYAGKAVDNSSTAIAACCKDGVVLAIEKIHSSRMLE

NGSNNRIHAVDRQAGVCICGLLPDGRAIVSRARLEAENSRDIFATPIRGSVLANRIGEFM

HVYTTHYAYRPFGCSAIIASYADDGPQLYVSDPSGTVAGYYGIALGKAKTVAKSELEKLD

FSSLTCDEAVPKLANILHDVHDKQKDKLYEVEVAWVCDKSDRKFVHVPAEMIPPPPSS*

>Lp_000028600.1 hypothetical protein, conserved

MEAKGRVLGAGRTLSQAVSTQVKTAETRALVLKVLSGESESFSLHELEQALCFFTSHNSE

YTDVAADEFRILAEETLNNSIRSVLVYDNFSGYFGDAEVYSLSLAFQYNLSVEALTLSGI

DVSDESICALCEALVRSRVNYIDLSNTPLEDEAGRSIAALAHVNPYLRTVIVDDTLIADD

VLDEIDVACQFNHSNWEGNDGRMDESLFRGADLGRLKHRLQQTIRAQHKKIHFCVAHLFG

CCPNGDLCLYSHSLGTSGAKEVDTSLSAKISELFANGGDWEERLPPRPSDGASWRNPEDE

APKKPRLNAVRRERLKAPNDSDKEMKAQSTATAAAGTAAHSRWAPLLNGLVGICCGMLVV

GVASIIARRFTRSANAV*

>Lp_000028700.1 Vacuolar iron transporter 1, putative

MNRHELSEQSSLRVVAEPAGGYKSCDAARAAFRAGDIEASRREHMKPMHEESHNSSASEY

VKSVVFGGLDGIMTTFAIIAAAAGSNGNYATVLIFGFSNVIADGFSMGFGEYVSGEAERD

NAITERRREEWEVENSLDLELDEMVQIYMSKGLSFDDAHTIVGIISKDPKMFVDFMMVEE

LGLLVDLDDAHGPKKQGAVMFASFLFFGSIPLLAYLPGKGQGTDAIFIFSCVLTMVSLIV

LGSLKGFLVGVSITRSAALMVLNGTISGAVSFVAGFLIEAALSKGVHASPAPV*

>Lp_000028900.1 hypothetical protein, conserved

MSSGALGRGSYRSVVAGVNPRCIPTYYSSAYELIQLYRANRDVTRSFLVRDKVFDNKFPG

CALANGLFKMVPNRREGYHAREVTEAIRHRTIWIQRIQQQRAINAAILDDASKELTPAEM

TTRFSYQTPDSAAYFSPQKYSAANNWPNYWQHPTEKHVVPRLRWRREPELGGITRVREAA

ATANF*

>Lp_000029000.1 hypothetical protein, conserved

MESDGAKTPAQLNDTAAFNEQHIAPENGSAQTPAVQSNSVSQSAATLPPCNDELRGTAKR

NGTQRGIDFTAKFVEKRSNRTTNNKTGTKTEEEEAAARKKAEEEEAARKKAEEEAAARKK

AEEEAARKKGRGGLCVC*

>Lp_000029100.1 Domain of unknown function (DUF1935), putative

MGCVPSSTYPEHIPRNDDDSAPFGTTSVHNGQNGDDYVVSRQVSVALMISGDGTHPVHSS

SDCSNKPDSLCTTAVPPLRMQSQGDVGADGIGLASTTYITSSGKKYKFLYGRPVGFSGAE

EIVSCLDDGGRQGNLYRLVRSKLRSIGEEEASMGDDTDGAGRRHTDTSSESEAKERGEGM

NLVACSVTARRQSANKSAERALQEQYVVALLGTDTILDTSAIERSRQRQRLAVLQTKSET

SNGKCNNSASETPRLSLPPPPSPPPAKAKEEADGLQMSTRRDLDLEEETDYQLFPQDHYS

TLPAQRQAGAAVPIALCPPSRILRVPVQYSAVEHRMTAAPGKPGSKDKNWKDIVLPQRVF

RLATSETRTWAFYNDSEYVMHVYTLFDRSSKLEPRDSTRLWPSRVFDDGNEAPTPPPVFF

GLFFDEDGEEAVPKSEMPDSVTRRPQHIFGTRGMWIAELLIPPRSTRLFVEGKIRGAYRM

LCTRLGLDDVVAATEMAQRVARRRNARHAPERLHLSLWPYRSSAAASASPSAATKAAAAA

EEQKVGGAAESLPLTSFKTMPHIPHRGNTVTAVSSVNGSASRSKNSAGLRKTMEAATRTQ

QARICQSATTAAAMTTTHPVPAVASHHIVLHTAANMSNPLSLPALRTPRAAPSQSLPPSA

AGPAASPPAFPVCKMAPTSLGADAKLRALPLEAAETETAAAATTTQSGGAAVPPPLSSPE

PVGEFSVIQKHTAAAAAVAVAAEDTAVNSSSKSSMQTELPGDHPVRRGKRHDSVPGDNAG

HKDSSCASETADQNAPHFSPSLSSFVARRIQRDGDNGDNGAEAMDDTVSDAGSSCVEFAK

IRPDRQRTTSLCGEVPLSGSTTSAHCKSTDQQQRIAPVRKSGVSRAPEALSPENTNTLNN

RLPPWQPKMSTLTPQSTISRCRSGTDRLSTSSYRTVVGDDSG*

>Lp_000029200.1 Domain of unknown function (DUF1935), putative

MGCTAAKTQTQFLNGKPTFQGDEVVKGFEKGNGLLFRITKKKKKQETWAFYNDTKQYEMH

VRVTFHPGCDIKPLGNAKLEEEGEKGEVVVTVTVAPGATEMFIEGHVNGFRSKMDAFKVG

GCDGAAPAVTV*

>Lp_000029300.1 small myristoylated protein-1

MGCGASSENANVTYLNGKPTFKGDDVTKGFEKDNGLLFRIVNKKKKQWAYYNDTKQYEMH

VTVTFNEDCDIKPLGKTRLEQQDNGEWVATVVVYPCETEMFIEGRVNGFRSKMDALPLSD

EYRQRQEEKEKK*

>Lp_000029400.1 hypothetical protein, conserved

MRLSSLLRRTSRKGPGSWTEPILLHSRHRLSEGSPQQQSSSSANAAASARNGNTKRKNVG

RSPASSSGSAASFYDGADNAYQYASARGMDSGHGSASHPSHYSWKADPALNKAPPGMDFT

GALRGAEHGALPHKTTRVTNAAPPIQDLGNNNGGGGSSKAQQSTGTNWLGGDRGKAIPDT

LEHATMQHAFGAGEFSMVDRQKLTLTRLLRLMADATLKPRDADSRAFGGDGATKLAEQRE

ELKTMDQRIVPFQHDDLLNDYLKNVDRLHTPSGAICQREVLQGRLVGLLFFTESERSLAF

MRRLQPFHKAHSPDFVVVAVSLAGKEMMDVTRSFGFFHCTHRDGATWVTRDAGLMLRPLV

PLPRLVIVNGTTGIEVTRGGVTAVMANPSTCFQAWRRNESGYRWRDWLQSMYM*

>Lp_000029600.1 RNA recognition motif. (a.k.a. RRM, RBD, or RNP domain)/RNA recognition motif (a.k.a. RRM, RBD, or RNP domain), putative

MFCVATPTSVTGPAMLSAPSPPPTAVPNSVTPLRCPTEVQFYTPSSTNLFVRYLPHEVDD

ERLRQIFSAYGTVTSSMVMRDIHSGASMGNGFVRFATHEEAARAFQEAHGMPLFGKTLAI

QWAKQQHDDTPAGQDRLKMNKLFLRNVPMDVTADQLVELVRDCGDVVKVTLHNDTAPFQD

RCRVRRIAFIIFDNYGAAETALRKLHNSFPFLSCDGIPLMGKLSEDYKSKERTPRTPPSG

LPPHTPHNNASVNNNNSNPPSAHYGYPGSARSGTCSNSMTPRADLPNSLSTSQIIIETPR

KIDTPRSVGMASGRTNQTPQTLSVVEVHSVPSTPHKGAMVEGEVGVAPLRTRERNAAPTT

ITAPSGGPTVTIRNRPSARS*

>Lp_000029700.1 hypothetical protein

MGRRKSVKPEKTEELRRRPSQYRSKNGGRSRNGSDESDGQGQLMSREPSMRKLHHSLSKS

GSSFHRSSRSAEDNQPAAYKGFHFPSGEPQQQLEPLLTSHGGLPSGNATTHNGSNNAPCN

SSQPGSHGRAPSFYGGASANICSPVMNGHYDGVSPIHNTNVGTAANAAPLTRRDSQVRMS

TDTLTPANLSPSQPPPPPTLPLLSQQQQQQNMRYPMPINGPLNVSATSYQQQPSIDFNRM

MQMYRNLPQLPYVMTPQGPLPPPPFPF

>Lp_000029800.1 hypothetical protein

MDHNGGGGRGGRGGRGGMAGAADHHDEAVQEALEARFGPRPGGRGRGRGGGGGSGGPSHR

DAEDLWDDPDPETFHGNRQLGQMNWQQVELNSVMQGDSGHDMLEAPPSHLRQDAPPQPRG

RGGRGRGQHYRGAGRGRGAVAP*

>Lp_000029900.1 hypothetical protein

MKRVLVLNGDSYVGRHVVRAFHQSREYDVEITRSTSAHTPGTPLCQQQQQQQREADEHAS

LPADIRSCVCGIVPKHNDDTEVFRTRVLANDVVVAVLQDDVYEATCAIRILESTHYEVEK

TFVLVSSVVTWAYTLVSERARARLTRQRERQEARDTFFEELLDQEDEARGDGGDGNADDG

SPAAAAAAAAARAARRHELEPRLPPSLREPLPGEDEDEILELVPDRVYTEDAYAQRVPHP

RFQHWYALEHLVKRANTQTLHTYVVFAGLPYGAGEGEAMLYGLLRSAWYHQTLLQYGPGT

NAIPMIHVQDLAAILFKLGNSYDTLEERYIFAVDQGKVTQRQLLQAVQARLGGSVQPATS

SQYYLLPTSTRVAAAAASTGDVKATTTGLTAVTRAFASLSGDGVATVNTVRSSLPTHPLH

PTTSSSLGNATSATVSADLVKLDRLELPLPSSPQNLFYVFGNEPCWGDGGALLSALVLSD

IQAQPAAALNLHPAEAWTALEGFTLHLDVVLAQFREAHEQAFRPVRVMITGPPLSGVEGL

AALVARQCHTPLLTLPSLVRDYRAHVANVREALRRLLVRRLQRRRARVQTRLIRRALDER

WAKKAAEAE

>Lp_000030000.1 hypothetical protein, conserved

MNPSQTPAQASASESGSAAPPPPKTQAAVPLTSVEHGYAPSANNPNPNASHDHTSKGTSL

SSPSHPSKRGSLSGTPSHRTKSNSASTFTPAMTAGVVDSGVSSTKRRSGQATTPAAAADE

TGNGALNSTNASLKGNAAATLAPEAVQERKGRRASRRGNANWFESEPTKRPADTELNQNV

ATVNDGVADQELARREQAERAQRQAEQLAQHISDPPTGFQPTLPRLSELDVTNSWDKLLR

MIRNEYDMSCLTNCLARELDEDVAWNPEMLLVQLTSDMMDAAELQKDNEVYVPVDASDIG

QMTGGEVVRKRGEKTVPGQGESGPAPGDAAGDTTTATNSNKDNAPSPKASAGFVADDTPA

GRSPSKRKSSAASKASSRTANSPAKKEVKPQSNKKSGVKPLPNKAS*

>Lp_000030200.1 FtsJ cell division protein, putative

MGVKSKKKAKTRLDAYYRLAKDQGYRARSAYKLIQLNRKYDFLAKSRVLVDLCAAPGGWC

QVAAQHMAVGSKIVGVDLVPIAPIRGVKTFVGDITDDKTRKIIVTYLKKEPVDCVIHDGA

PNVGGVWSRDMYDQNALVLASAKMACAMLKPGGWFVTKVFRSPDFHNLLWVLKQLFDKVE

ATKPQASRMESAEIFVVCAGYKAPKSIDPAFFNPQKVFAEVGQEKIVSASGLLVTPKNNV

PAGYDEFATVPHRVASFSDFLRADDPKGFLKTHHELRFSDEADKQYLKSKSSKKELVYLC

GDLQQVGEADLRRMLRWREQLLKESARQLQTDANEEEEEGGEVDSMDGGAGSDRGDADDG

ASEDGQAAAAADGSHDYHFDFDSPEGVTQIARELLEIRKKKAKELKKKQKKVVDRKLKQI

KGLINYDPNMSAEHMTEADGFDYKGTNTDAFDKWGEEAHKRIGKDDTGDEDREPDELDNF

TVEQLGRIPEADVAHIMDTTFTEPEDRGNDPLNPVMSVNLAAQDDWDLGSGEEGEDGAAA

DEAEPRSKQLVEGEEYFMDVDNYGNYVPAERSTRVTLYEAGLENDDDDEAEKSEAHAGGE

DESPEARKKRAKAEKDAAQLEEAGKSSKWQRHQLNIEKVLNDTFPKPKDHDRVKNKKRQR

ALEEERITMSAPAELPDPDKKRHVRTRFEDRRSEDDDDDDDDSDAAVSDLSEELSEGDLD

DYEINRGREDSRRKRKLIPDIGKLTTQELVRQQRKQTLHDNKEVRKQNKRNKNNTNSGKK

KSANGKEDTEFEEIPIAMTDPEIRARTLAIAQKMLDPKARRDIIDASVNRYVFNDDDDLP

DWFVKDEQRNCRVVLPVTADEIEEQRRRFQELNARPSRKVMEAMGRKRRKAQRMLRSLME

KGKADPRAREKSGNLSVRKLMRAQVIKGQGKKKNKYLDRQEMGRMRREREKLKRNKKKR*

>Lp_000030300.1 Rab-GTPase-TBC domain containing protein, putative

MSRVSSFVVEEEVVAALPLCVRRLRGPGLARRLPPGSAYRSAGAVLRRVLLYSEDGQARV

QVFRTDGLGVPKERSTSGPSTVSPLPPVSATGAGASTATAATASSTGPSGSTKTGTPPAK

VVTSSNFAKWPASLSSVGLMSEAQEEGINTRHATHISSQDASVVHTRSQLRRERAATLAT

ETERRYEEEQESTLAGLLHALPLLRVVPPLSLKQQAYTQESSPPVNANASRNTAYAMDRA

SQLFPSSSHFITSSNRPNSNISIGSSSNGAVAGGVSPAALRVAQARYGAGYVGGSVGGDA

ARANAVIRDQRGLGLSAAILLSKEETTKREFPQAPAAAPDAEAVVKPELLATATATPPSP

VPQSGNASQELRLAEAAPATADATSAALNDIFIDGGLNVTIKLGASCDEPHTLRYVVWGG

CKPVVLRAVVWRLLSDYAPVQVSRQHAELERKRRQYEGYTRQYCSALTMLADSEPAVAAA

AATGASSSSSFMPPSPTRGGGAETLTTSVLGGAAAPQRESASLSRLSALPRPGATGVNRT

SLGGGGSNNNSGADVNIAAASLASHERAIIRQMLLDLPRHKSPVFHARRSVAGMARCLFL

WSQRHPAVGYVQGMDDVVAIFYQVFLTDALRQYASEQLMRQGRSSSPSAVARARLGAVPG

ERNSNQNTSYDSVEDAEWDEKSSERITREIMRLLLASLHAHRTTAGSASSTPTASSFAET

HVVAPCLVNNLAPGASELTADVATTTSARTAATRATPVDASLTLLPFLTEADVDVYFRNP

AALDAALADLPESYLTQVEADTFFCVGRVLSFLQDNFMTGQPGILRNVRRLEALVRVVDP

AVIVFMEEYGLTVMDGCFQWMHCLLARELPLSLLLRLWDCYLAIGIDVDRPLNVGMTAGL

PSTSGVGGGGVGATSSDEAIMYFHVCICCALLRMVHTHLTGGPTATSATTETTTMTTVGG

WAAALVTALPGFRSRATQSSSGESNAAAAASSRARPSIDEVMTSLKRPFEALFPNYPTTA

VKQQRWDAAQQQQRPGGAAVRGPAAPQSAPPPPAAAELAEEDAAERWLDLLIADAYCIWR

LHPVTS*

>Lp_000030400.1 GMP-PDE, delta subunit, putative

MSSLSITPEKVLTFTAPTEDFLCPLTANTYGVEFYQFTIRDVEQNKVLFEVGQAPDSAPD

EDVQETLARAAQLTPEEAAAMRTIRYQFAPSFLRKHAVGAKLVFGINGDKPVPKFRMIER

HYFRNCLIKSFDFEFGFCIPHSTNTWEAIYDMPRLNPEWEEAIIANPYETVSDSFYFVDN

KLIMHNKAFYAYNGPER*

>Lp_000030500.1 D-lactate dehydrogenase-like protein

MTAPISGEYNIGTFEERQKKYALCLEEIGKVLKNPGKQLELRKSKLGWYSKDQSHHLHVL

PAAAVVPTSIEEVAAVVKICSGLRVPMTPSGARTGLEGGAIPYAGGIVIDTLNLKRMDFD

VENACVWVGAGVKKLTLNKECAKHGFLFGPDPASNPTVGGMVSTSGSGMSTLRYGTTREN

VISLKVVTPSGDIVQTRQVVRKSSAGLELTQLYVGSEGTLGIVCEICFRLFPLTKYAAGG

YAAFKTTGDAVRAVVALRQRGFPPTLLRCELMNGHNIESTNTYCKMSLPTTATILLEFTG

NDWWLRDIKRDYKYVEKIFQEAGHPTEMRYLRPGKETEDVWNARRQCHFSAMHARGAKRA

EKCFGTDICVPVPKLTEMVEFTEEKFQEANKACYLCAHISDGNYHLIIPYADDAELKILQ

SIEDKLIKKAIELGGTITGEHGLGCSKVCYVAQEHGQAHIDVQESIKKALDKHNVMNPGN

FYPSQVAQYHPAHL*

>Lp_000030600.1 Mitochondrial carrier protein, putative

MSTPSSLSGPGAPQQEAAKSKPHANAAATAPALLTRPQNDKGFLYNLFLGPGGGATLAGM

CEIVIFHPFDTVAKRLMSYQHRVIDVASPAATLRNLDHVVFGKLNERFTDPTTGVVRHIP

ALERLKHVYPGSTYAVAYKVLQRIMKFAGQPYMRDYLHVHHSRLFFRTDPATGEFVRGGR

GAMMLEATAGCLVGVCEIVLLPFDRMKVLNQTNKAAVQSRNIFSVIRTEGVAKLYAGALT

TATRNAAGSFLLFGGTAFTKEYAFRLQNYRDATFAQNIAASTVGGCVGVFFTSPMDVIKT

RIQSQRLTERMSGWQVFTSTVRCEGFSAFYKGITPKIITSAPRLVFSYTMTQYFVKKLRG

E*

>Lp_000030800.1 Transmembrane adaptor Erv26, putative

MELSNKPGTFEYYGDDDDGEWWARPRHAYKSLGPFRLVSTLMVAVAIGLVAFGLVCAVMF

VADVAEEYPSRAKWALRALITFNVIVHVSIMLIDQLSWWRSVLSLVVNALYLRLLRSFPF

VPPMNSPLVLCTVAAVLMESTMWYWYAVKLMYYTALLNIIGFFLMLWLVPVGLLSSCVLE

EDRLPGAESVYGSGGGAPSGIGGAGGSGGRKKRTILNRLADLISKP*

>Lp_000030900.1 ribonucleoside-diphosphate reductase small chain, putative

MLVKPNTASGVAAGAKRLRPDEPAPATEVSRTSGEAAADQSRSEERLMKPLSCGAAVLPA

DKAVEGTAADDEPLQQENPFRFVLFPIVYHDIWRKYKEQESCIWTLEEIDLGNDMKDWVK

LNDGERHFIKHVLAFFAGSDGIVIENLAQRFMSDVKVPEARAFYGFQLMMENIHSETYSV

LLDTYITDSEEKLRLLHAIQTIPCIQKKAEWAVRWIGSGASFQERLIGFAAVEGIFFSGS

FCALFWLKKRGLMPGLTFSNELISRDEGLHTDFACLLYNTHIKHKLPRERVLEIIVDAVN

IEREFICDALPVRLIGMNADLMAQYIEFVADRLLVSLGLEKHYNVTQPFDFMEMISLQGK

TNFFEKKVGEYQKAGVMSTEGTSKKFSLAEDF*

>Lp_000031000.1 ATG12/APG12, putative

MPRRNKKHASSSARRRKECDGEEEELEDGGLTTKNTVPGDKGVLAIPALHATQVPEFQAA

SLCSSSASSEPYVSPCSSDLGDQGDEAPMHHHESTMAVNSSLSHRSQALTPGTDREQHGG

TREAALGKDFHCTHEPTDKRGTSECFKGTPSSFATAHKQADLTNAPSSFGVASSSPLSSP

PPPPPPRTHFQYIHSFDYRHRLAAKLRALYGEQSIPVIVEPAESQLRANPNSAPPYESQR

VAAETRLRSIGIGRGLLTCLGVKETSTRGTHGFSTPAPLALSASAAAALASPSTRSTLKC

VLPASKSVAEVILTLRDRLALDSAQSIFLSVGENDALVPGNSLLGDLYQRYCHRDGFLYF

SYLLENTFG*

>Lp_000031100.1 prostaglandin f2-alpha synthase/D-arabinose dehydrogenase

MSAVGNTVVTLNNGVKMPQFGLGVWQSPVGEATKNAVTWALQAGYRHIDTAAIYKNEAAV

GGGTRASGVPREQIFVTTKLWNADQGYESTLAAFEESRKKLGLDYVDLYLIHWPRGNAIV

AKEGKKYLDSWRAFEKLYAEKKVRAIGVSNFNIHHLEDIFAMCKVAPMVNQIELHPLNNQ

AELRAYCKSKNIFVEAWSPLGQGKGLTDPTLISIGKKYHKTAAQVILRWDIQHNLITIPK

SIHQERIVENANVFDFELSAEDMAQIDAMNTNTRYGPNPDDADF*

>Lp_000031200.1 calpain-like cysteine peptidase, Clan CA, family C2

MYIKQNVHGIKEALFGGLLYRYSDEETGRWTFYNNSKDYEFHIKYLFGANSSLEALGDTS

METQDDGILAEMVLYPLETKKFVQGTIDGYESKLEALPLTEEYFAQHPELDEEAYYRRLE

APKSSKF*

>Lp_000031300.1 calpain-like cysteine peptidase, putative

MHESAINRTVNQNESPQMMPLTYPGSPSTSSVPSSLSSLEHASPIVTPSPAKAALMRTAL

QRLPTSGSGKSRSRGGDGSRVDCASASTAPPHLEGGGDDVPRIEEMAVMAASLMKPYHTI

CRKLGNTNKKQSSFPASHVADRLARTEGPDGGGDGATTRATTDSSTSTPFMQTLLFSTPP

PEVSKAVAASYPARWFPSITPCFDGGLLYRIEWRRRGHTPLPGESADYAYVAWCFHNSTP

CYEMKIDVAFEVPAGCCRRAPPLRGWSETTLSWMEEKEATASASPATVAAGTHALMKASA

TCPPLSTIGFITGRAGAFEISVSGVPVSQGPPRTLASPFSDMKLDTGRRTLSSEEGLRLA

LKERRRFVDPEFPPTNASLQGKGGTPDGATSSPTTTAVGWMRTSHIVARLVGFSCDSLAR

TLQPLVPTIDPLYIEPGELGDSWLVGAMAAVAEHPGILLRMFRHPKSSENARIERAIGAF

RVTLNVQGWWRSVIVDDFLPVTDGNYPRYAHSRRDVRELWMGLLEKVFAKLRGGYANIIA

GDPLDALQVLTGWPCARYDIANFKDIAVASSAFASRLQRYDRHGLQIIFHTAPRLSSPTT

ERNGSRGSSGGVSSGAGAGAWGVSFTDPSLSLDDDYPGSGGGRGVDTSDENGLVPGMVYP

VLQVLQFSTNPFRAELTLLQVRNIWGDVAAWKGRWRCGSPRWAQWPKVAEACGMPIHPSH

ELKTEEGGEGGGEAQGGAAAESSGSSSAECTCQRQKYMWLEWNEVCRYFSGCGVIFRLAL

HHDYRVQGVFEGVRPSVCLRVTVANRSFVGLTLSMQDTGVTAPQMDDGGAASGHPPIMVS

LAREQANVVYILRNSQLDPDNPTSLFTFMQASEASLLSLLTPEDSPYLVIPRMLATAAGT

SSADGAAPPPSPPVVNRPYVLGFFQKDPVGVKRGSRVEFVQLPATSAAFQNSTCFSLGNQ

VKSVRAKFQVKSPQAGFPSTYVHTELSEEAGTPADDDFDTASACV*

>Lp_000031400.1 hypothetical protein, conserved

MAAPLIAGILVGCGLYYVGRIAPRVARRASAASGASTARVIRHTRPYHRYEYGFQSAMSE

REAYMLLGFKAKEAEAVFRRPPPEEVKKRYRAMMKDFHSDVSGTPYIATKLNEAKDTLLK

*

>Lp_000031600.1 WD domain, G-beta repeat/PFU (PLAA family ubiquitin binding), putative

MGSCEAEEAFILQADGCTHTSDVRYVSVSAQDCSAVFSASRDNTAKWITIPEAGDALHDN

LTLIGHTAFVNFVLFHPCIELLDNEPCIVTGSNDHHLVLWNTQTAAVEAVLDGHSSGTCC

GAVLSFTTDEASADMRESLAGDIVSGDWGGVVIIFDHKSGRPKQLYEKHGTAVRGVAQLT

STATVLSCSGDKTIHAWDATTGKTIQIFSGHSDVVQCICAIDGQRFASAGNDCSIRLWRL

GAESPYQVLEGHDSLIYSVCWSPRTSELYTSSEDHSVRVWHSAPTSDMLVTVQVIQHPCV

VWSVAVTADAKIVTGGSDHTVRVWTRDYDHIASIEKLEALEAAVSSQKVDVKVAKSSGTA

AATGGLDVASMPYTHEIQQHRGTQEGERLFARNEKGEVELYVWNASQWEKIGVVVAGSDA

ENYTGAPNQQREKHHHNGQTYDYLFDVDVEGRMLKLPYRVGDGLVETAKRFIQDNRGVVT

EDSQEEIQNFLMQHISSEDLARVPGLEGLSSGGVSQAPPAAPPPSASAALATTDALLAPW

TAPLTFDTFNPAAAQAKVNSLLPNDGSAFHNAVGQLATPPPDVGAVGSSLVKLYRGLPVG

SRFPAIDALRYLLIISPDKKAAVEEVLRGLTALWNGQQPQLPTSPAEWLGSLRLPACVLA

RVSDQHVSLASWDTAAQSTLVWLVARLPAMTALLASDTPPATRAHCKSATAAIFRNTAVL

LAAPLATNASRLPVEMAEQLALAVVQQSAILFVSERSDSPVVQDCLRSLRTLLLLPTPPP

SWRAAVVAQMQKSLSFSLRSIATGAYVEGQSTAVWLLAEIGCPAVS*

>Lp_000031700.1 cyclin 10

MISVTETTGQQISPKAPATAENYGRTQASSCHHHHHSSEEPMTKPLICSFFKRASAASAA

QNCANCANDRGSASGRVSSAATLSTHSVDAETPSHLPPYPTPMEEPILLFPLTPSVKVPL

RHSKRSGRVLNESETLQEKHAQEYRFIVPMLAYAIECTIAEHEIIRQSAGLPAYISLNSS

PAESPTRQDPHVGASENEGENETAAAAGTDRNAALGYKVFQTAINAFSTREVPAISVHDY

LKRIVKYTYVSPSVLVCCCLYLDRLLVMYPSFLLHPNNVFKFLITSTRVCSKVMDTRTLN

NRDFSVVGGVTNEDINVLEFTFVQLLQNRLYISRHTFDMYCEPLRRQAAHVEAEGEWDGV

TLTMVLPVNGGGRSAGYPHQPSRPSQARNSACGSAASRSRRSSTVSQNEYSLSMASQSGV

SLLRSASRPAFPPSASTGQPISVDAEFLAPVRQPSAATVSSARRHPLSSSTNNNTSNTIN

GSSMNNTSTTSRSPVLPQVHPPATYNAAASTAVPIIRNVGSAATLRSSSNFEDTASSGGL

AQSDNGTSVNQLQPQPQSGVDAADDGDDAHVRSIPVSGPRGFSSSWDTVRTTRTLAATAG

GMSGVGLSTSTITNARCGSPNAVPAGAGASMSKSRSLDGVTISTEGNMTPSNALSGERTP

TRRAFVSSARGDNVALPPVPQNGRRGRSNSLAGNTGATIPNVPPPPEFRQRQ*

>Lp_000031800.1 hypothetical protein, conserved

MVYTRWKCDRIPVLQLKLFTQEYNVMAGVGLLSMVFMWKHASFCSEETERKNGWWAGYPY

WRDPIARRNEVKYKQLINNNNVDITDPKWTGCSKEQLERLRVIV*

>Lp_000031900.1 MORN repeat, putative

MPTSGEQKAQKVNVEEIDVTTGFKKAEQHVWNAYASAFLTPGDEPEMDMDAWIDDFCQRA

EESGAIFDRNFVKSVCTLGIYAGPRDEFQQRSGCGKAIYATGDMYDGEFYEDKKHGSGQY

TFVSKGRSEADRAVLKEVEQLGKTGVDANDATDEIAKKLQLGPHIVEGVLMYGPHPCYRG

EYVRGKRTGRGVMKNKDATIYKGEFLENRRHGQGVFYYLNGDVYSGNWKNGAKDGYGTYH

FVNGSEYRGEWVQGNFTQGQWIFANGAYYEGKFDKKNRPLDDAASIHYPSLEMAQTGTFK

RGVWAPTSALQICSEVPVDGMAWAD*

>Lp_000032000.1 eukaryotic initiation factor 2a, putative

MASYTVTDSPDTVDYKTKCCCQTTDNVYYAVPKEYFRLNQTLARRKLLVAEPFTVPIDSK

VMDHLLVLLEKATILSAQAVGDETKGSNNERPEWMRDLNKRQQKFVGACLGITSWDGKDI

PFYEETMPKIGDVVWVKITQVNDTSAVVQLLEYGKREGIIPYTEVTRRRVRSMGKLIKVG

RTEAAQVIRIDKDKGYIDLSKKLVTPNEAKTCEAHFRQGSEVRSIICHVADECDINAMTA

METIAYPLYKREPGKHAWSWLYELNQTQDVDRILGPLSIEKRVSDCLMSALKNAMRMKVL

TIFSEIEITCFACDGVEAIRDTLILGRDFGEGKDPQISLSVNIIGPPKYGVRARTDMKEE

GIQRMKEAIEAMKAEITKRGGQLKVVTPPQPHGDADKGEKSFDGDDEEEEDAD*

>Lp_000032100.1 surface antigen protein, putative

MASLRTLNVSGSRLSGSVPASWGGLWQLMSVDLTGTGLCGCVPAEWAGKAVVADAALTGS

DCAVANACSKVVSGSQSESSSSSRRVASSWASGILEQQQHVAGGRVRGGALCALSRGQRH

QLR*

>Lp_000032200.1 hypothetical protein, conserved

MAWLNHRLRRMAVAAALLVLCVVDASLVGARTASDYTAAQQASTLQFLQGLCDGQSFPPR

CVDGHLLQLVVRDVQLVVSGQA*

>Lp_000032300.1 D-tyrosyl-tRNA deacylase, putative

MRAVIQRVLSGSVTSEGEVVGSIQKGLAVLVGIARDDTAEDMEYIFRKILGVRLWSNADG

SKMWCRNVKEIDGGVLLISQFTLMHVMKGNKPDFHHAMPPDDALAMFNALRDKLRADYAA

DKIATGKFQHYMNIQLVNDGPVTLVLDSKNKG*

>Lp_000032400.1 hypothetical protein, conserved

MHVSIAVRSLRGPATTRTFLKAPSLSTVLRTCQSPRRHPHTSAASPPHSNVPPPLAGTPL

RRCAAHHKCSPRRAPKLSAASSSAAVAPSWIDVLALPTAGAAPSRIALGEAAALCLSMES

PKCYAVYRPCDGGEGSAPHARVAPHHFVELLAQMRHAPSLSLEARRVAEVMVDLLNKPPH

PQFPGPWDISRPEVRELKASAIAYVMECVAAGDDALVWLAGLLGLTDTFGEGETAPASTR

STPVALLATAVKERLEWRDAVATQRVCLEDADGDWLLSSSPIVMGAIAAAMLRSTTDGAK

ASALELLEAAALSRLTRRSSVGAHPAATETGKAAVLFNEAVAVHCAEVAAVRGDVETVTR

LIFLLYRARGALGCATLAEHRRQQRAQRRWGVAALLPRTPVWMRAAWWSGDAPLTSMDGD

AADVRFEQTVVRLLRSVMHAALYHPPRSGDPVDGSVAEALSLWDGLRGGTSWPGLAAMAT

EMLTYLVDQHRLLRTVGGERDSGASAAARLQQAGLRVYTQLRRAAPAAPCHRTDAQQEVL

HHCVALVMQLFSIRTVVHLEKSDVVVPIVPTSAAAEHVLRIFAHAKGSSQGEALLPYALA

ASLTLLSAATLKAYRPPAALFGDLARYFAEVQSLHYYCVASGETAPALPHIAVCGSFILL

HVVLLSHAPLGAWAREDGDLWSFVQRLRSLDSVEDGSRSSGNVPQHSNESRAPLLAWLRV

SNCQQRQLLWSLLTSLKQREMSWCAAALNLFASPACSTAVFTAADTATCNCSLPQPELRT

VMDLLYECGNTDDPAQASATLDRVVSTCARMPVRTLALTALVDQLQRKSASGASAAVLVV

TDASLRAITQRCCCADGATAADPMAAYVHLFEPLLRTTAARPAPSAVFFVLTPACLMELQ

RLRAHDSTSVVETLHAALSGTPPHGLYHVAVASAWPLLPSLATALRRHGGLRAVDGLRET

TAAARYLEQAFPKETRPAVCLWTEEADEAPPSTPNPLCAVQVSLLQHYGAREVSTYRAHM

EKAKSDIFSLLCSSSLTSSRASDLHVFAEVASTESSAARRVRNETLLSAVSQRRKGQRTL

*

>Lp_000032500.1 acetyltransferase-like protein

MSSGSVVTIRRAQREDCERMYDLILELATYEKAPECVTVSKEEMEELGFGDRPLWSAFVA

EVQDGDAAPHVVGMALYYYRYSTWRGRMLYLEDFVVTEARRGAGVGKMLFDRVVQQAKEE

GCHGMVWQVLEWNEPAINFYKKYDAEIDPGWMNCMLEF*

>Lp_000032600.1 Nodulin-like/Major Facilitator Superfamily, putative

MVEGLAHTFSQTKHVADRVARGLHVIDEVKRFRALSCALFCSICVSLVFAFDLFSDEFQK

RYSLSDGDLSTISTVGVVFCYFVVPYGILYDYMGPLPLLVIAGVTGLVGCLGLGLVFDGK

IKGTTATISVFYAFMNTCSGLFDAASVVTIVELFPRNRGPVIGLVKVMTGLGSSVFSTIS

RSFFSKNISGFIYFIMALTVAVAIVSIFLVTLPPYFINGWRKRGKTEEELAALASLKAIY

AKKFVPIRRIACGYVIVACLVIFFTITAPVLAYTNVSKAGKCVVGAITVVLCMSFWVMSL

PIRSLGGVDEAAQYCDSFDGAEPFDDTVTREIDGGKAPLEVSGEVLDDSREALNDSCEVL

DDGGKAPLEVSGEVLDDGGKAPLEVSGEADAPEVEKAVIEEGPQDPRYGGTIWQTLSRPD

VWLLLIAFVCQGSLGTIVTYNGSTIYVARTGRARSAGLGSLYTAFIGVGSAVGRVAMGLF

EAYVQQQDPEKRKVLVTISLPVAPIVATVAGILILVLPGDALLFPYILVYFEEGVFNGVR

ALIFPCIFASHHGILYIMSCFTNVIGVICFNRFLFGLTVDSEREKMGHTVAQGCTTRACV

QTPIIVVTCMAAFAAVLASIVHIRYSRFVNKCRGAKPAKEPPVAERQPSTADVLVA*

>Lp_000032700.1 hypothetical protein, conserved

MSGPGSSSRYHNQPASARRHSRDGDYSRHVDEAPAQHSTHRHRHRSSSGRSSPHKHRDPA

SQEAQRFRPEDYVLEDGVPPLRRQSTQPPRRCPSPTSHSFVAHREVEANVYRRREVEPTS

TRRPTTPHQHRSPRDSTLVDPAVSTALVVVEEKQVSPRRQSAHHAARASPRRSHHDSRIE

TEDGVVRSPRQHVSSTTSPRRAHRQTGSPIRPERRAESSRHRHRHASVELRTRSPYSSAH

RHRESLSARRASTPEATHRQDTLRYRSPPLPEESKPRSTSRHARRHSHAADDEPEKKRTT

STSHRRDSAHHGRSSRTTPRHDSERRHRHERHASITASTNDDDDSRLARHASRDRQASQD

SSMVPRVPVSGGSTYVRPNGNGHISTRRNSGSYIEVISVNSARPESSSSWLQSPRRPRDA

GPPIVIPDPRCEKILERIDSTRHWIKNMKEEVAKDREREQQQQQQQQEREAEAEKKRERE

RERERERRKEEVARQQEAERARREHRRAEERNGSAHRHRSSSRGSQRRTRSSREEANGTA

ATPVLVEPERTVDSRRHPRPAVTPAAPQEKSRLQSAGDVGRSRARSTHAPSESTRTAPSE

KDASSRQSTRPAATPPAPAPAAAAVMHDTINSSRQDGFDYPIFSFVSFLDGNTFDNTAGL

CQYNTQLAESLSDEHLDLLREVVFHHDEEAFAELLYGDDVQAEMDVIANTLGHSEDPAVQ

REIVILQRAAVRRFNEKCSTALRTLLAARGGLADAVQIAASQLERQAYEAGANNANSP*

>Lp_000032800.1 Protein transport protein yif1, putative

MSFTPQSPSINFASPSSAPGSAFLDRSDPNAMMLQMGLSYGQSILQQQLQQGEAGLTYYM

PFIRVIRNYFAVDNTYVKRKLVILIVPFLTRFVRKPSAGGEGEFGGGGGGASGENSEQVF

GGPPGTPAATIPSGSPATRFGPSPASDGGPQAPTLPIHDVFACDLYIPLMSVITYIVLSA

FIDGANSLQREITADSLMSTAWVIAFWFLLELLVMKGLAYAFRVVTSAPFLEVASLCGYK

YVLLCIGVLIAQALPPSRLGSGLVMLYGLLANGFFTLRVISLRLMREDGRVPPRSRAYAY

ACAILQMPAFVWMFVRPLYTSE*

>Lp_000032900.1 Domain of unknown function (DUF4379), putative

MLRQTCVLAAAEFKQKSRWSGVWPNMHYGAMYLQYSVGRQLPMQGVNWVTRDSNRLTNFG

ARYQSVIDDVDVKRNEEELQIPLSDIRWNDHRRIYWKCSFCGSTYRKSVSVRTKFHAGCN

MCKGRYASEVLREQTSVTPLKEAQPELFRNLTENEKNDNIGALSVTSKFRAKWKCTSCGQ

PYQATIRSRTGQVEPGQAPLH

>Lp_000033000.1 Periodic tryptophan protein 2 homolog, putative

MQSVFQLAAVHGMLYTGGNVAFSPDGAQLYSPVNNYLSAVQLQAAGHLSLTCSNSSITCF

DLSPDGDLAIVVGQRGLGFFYSISARVVLDTLSFPPNCTISAVKFSPCGKYVAVALEATL

QVYTAPAKRVVSFHGCHRIEQLHAVLTRPITSLDWTSDSAHLLLCGQDARMKIVPRQGKL

HQKGMALQQNALVGHRSAVLGAWFTNDDNSEVVSVAADNVVVTWHRAAITRREVLQAIAT

AQLQARVGESEKSGGDSKEEEEEDVDDEESSSPKSFLERKRLEQLRLEGVRVSIADDAYL

PPILRHAYEIRDKFMLSHKGSVSVTAFHKPRGLLAVGYSSGIFAIHTLPAAKGGELTLVH

LLSISAQSLTAAAFSPSGDWVAFGSAHLKQLMVWDWKAEAYVLKEQAHYYDIACAAITAD

STNIISGGDEGKVKVWKVASGQCFATFTEHTGPITGISTSASTNAFFTSSLDGTARGYDL

VRYRQFRVFAPPEQTQLSCIAVDPSGEVLAVGSSQVNKIFLFAVQTGRVIDVLQGHEAPI

ACVAFHPSGTTLTSGSMDHNIVFWDLFNQNESGERLKGDGEVLDIGTEVLCVTYSSSGRR

MAVLTAKQEISVYETTVANDPQLIKTFLTSFDAAGGWRKEVGPNSANYNTHFTRISFSPE

GEKLLAGGDSKWLVLYHAMQGYVLKKWPITTNLDVQGAEEQYQWRNATEAGFLDDIDVED

DDIHLTRRKLLEMPGSRHRHFATGKRKTELKARAMDVAFAATGSEFIAATTDGLLLFSTR

VSRPRFQPLQLSARVTTVEVREQLASGQPVLALIGALNLGDAVLGVECLRCMPRDAIPVA

VTAVPSALFPHFMQWVSEEVESCRGLEHALLWAQSLLLHSNEAFGGVAAQQDPRVLPALK

TLQRSLYQHRLLTQLSRENYFSVKYLADAARMSTVTLKPVEDAAAEEA*

>Lp_000033100.1 hypothetical protein

MAKVKAVTAGAIHDSLTSQAARHRTAVAMEPNHAGISTHTSLSDMCTPSAASVRWMATAV

NCRPGYVVEPTGRPSGYQLM*

>Lp_000033400.1 dynein heavy chain, putative

MELSPAMKDCVTAVTKTIKKRLSGDVVDRKDVGTVVELQVQASLSTMPEAASTLFAAMTK

AACGADKGSTKLPTGAIIVDAAAPLPVRTFADCVRVSTFRFEELPTDNPTLCAIAEATYI

RHLQRSSDLTVYGVGEFPPCLLRDALSRFDVSVGGYVVLSIVVAKDKSVVVRLFTTAVDT

VMSSMCSTAFTSGVLPATWAKRSSLEPHQLLDALEDVQLAISEYWHDSAQDDHARSVAVF

LMSSVGSDICDFFTDKIAAAGGVFRGDKKLVEEAHLCCEEWVAVCGRLTTLDWASEWGKR

YEDAALVCVRDRLRTALVLRGIIEEVSELLGADDFRALKTGVLWDVFSDVAVMDSSASVQ

NQWNAALAAYYRRLEPIEHRCATVLRELFAARAGLAPQAILSEFSGYCHLMKRPIIAREL

VTERDGLLGKLNDRLAAIRLEYEHRAESTEDERVLEEEDRRCQTGRFFPGVVNNIIWLRQ

LRGRIEEMIEMCSSILNDLPNASAFATAAKQLLEEVQDYEQECFRGWVADVEDKSDVLTL

DADAPLMEIDAKGRVEVNFAERLVQLLKEVRVLGTLGFAIPRQITKIAQQGAQFLPMGVA

LKQVAHTYNSMAADIIPCTRAMLLEPALGFEKVITADGSKKLTWRSAKEAARFIGQLQNA

SQSLTDCNRRLHKIHQEMEGLVMELFEVSLLRSRDRWMRKVRQMREKIEQSGFQNTEAWR

EFWDMQLYKAMEAQYQRGLESLHETIEEMKADIVWDAESGRAALRPSLEALRAQYYQRIH

DFITFPQRFQGCCEKSVFKDMPSRNANGIYAVLRHAAQLFKRVAHELKRFTPYLVIGQCG

RGGNPTLEQIVSKSLTEVQHWEQSVRLLKQKGKEINSEELFIKCDCITLCTAAIKGAVEE

HLVRLTDALKVTLKQSAEQHLTAVNDYLAKAATCLDSKLTKLDEIGESNLLYGELMEQRP

AIEVEFYHFYNKNVLLQNMTGTAGLDFTKTKECWDTFMKRLDSHEKEVEDQLNQMRNTVA

DSVKEWENDSIRFTTRWHELKPKDLNSQKPLEFVTKKKAELQTLKERGEECKKQCEYFHL

DEPDLQPLEETEADIADYFNMWSMLGDFQQQVQEICAEPWISFRAKLYRFEDFVKMWQEK

LKEIPANSVTVHIRTMLDQWGRCVPLLKYVRGDGFTPAHWTELFTLLKLRGVTQDTVNFG

QILDHHESILNNEAQIKKLHSRAQGEAQIREALDDVRAWGTEARFALTPHPDRAGVVLIT

EWKDTMSALSDNRALLLSMKESPYFSLFSNDATKWEERLSTLDEYLRHMNQIQRKWVYLE

PIFRRGALPNEKQRFDRIDAAYLTVMKSVEKDSRLMALAEHGEFKSTLRDVSEQLERCQK

ALNEYLESKRDSFPRFYFISDDDLLEILAQSKNPSVIQSHLKKLFMGVHSVQFDAQKENI

LQVLSLEGEVVTLLRPVRVTEEVEVWLSQLDVEVKSTLKAHVAQCVAKPDIGTYASQVLC

TAEMVSFTRKVETAIRESAGGSALKKLKAGLQTRLRELTAYAGSNSDALVGIKLKALIMD

LIHNIAVVDLLIANNVEKESHWLWKKQLRFYLDNAQQCLLRMGDAEFRYSYEYQGNAPKL

VHTPLTDKCYLTLTQGMQLGYGGNPYGPAGTGKTESVKALGSAMGRQVLVFNCDEGIDFK

AMGRIFVGIVKCGAWGCFDEFNRLKIDQLSAISQMIQVIQQSLKSRESNCLLLGKEVSVN

TNAGIFVTLNPAGKGYGGRTRLPDNLKQLFREVAMSVPDNELITSTMLFSEGFTHARALA

KNIVALYRLCGQLMSRQQHYDWGLRPLKAVLRLGGTLVQRWKKANAGKTPTQNDETELVL

QSLNINTLSKLTFDDARVFQGLLRDIFPGVESREITFGELEAAVKSAVESLGLQPIASQQ

KKVLQLYEALQQRTGVVLVGPSGSGKSTLLNILRKALQTMQVTVPLHVMNPKAMPRRRLL

GYMDPDTREWYDGVLTAAARDAVKQPKETRPWILCDGDIDPEWIESLNSVLDDNKLLTMP

NGVRIQFGANVNFLFETHSLEFASPATVSRMGVIFFSEKDVQLEAVVESEMKTKSPHVQS

VVKPLILKYVFAAIDEAQRLNDFAVTVTRMGLLRTCLLHVMNTKNVQDFIFSLARGLGAC

LNPESATKLAQWLYSTSGQQPCSASRPFDSYWLESQRRAVEFTADLSATVTPAELLAGRA

PVVKTVEVQRLVSMLQPLLDDPKYTPIFVVGPEGCGKGALLEHVFAARPSFHTTTINCSA

QTNSTHVVQKIEQMCVLTNSNTGQLYRPREGERLVIVLKNVNLPKADKYGTVELHSFLQQ

LILYQGFYNQDLEWIGVERVQIVASMNPAPSAGRYPVTPRLLALASIVSVSYPSNASLVQ

VYATYWSNLLRQTDIGQGKDYEKGTQLAQFMLQVYDKVRKQFEGEEYAHFSFCPRHLTKW

VANVLLYHIDTRTTLPAVLAYEA

>Lp_000033500.1 hypothetical protein

EASCAEAKPSPLQAGLASAAGGSFAAGKGCRGASGLGVRGRRVLRSVFRSSKAEPAACRL

GIRGRRVFRSRKAEPAACRLGIRGRRLRSVFRSSKAEPAACRLCIRGRRVLRSAFRSSKA

EPRD

>Lp_000033600.1 aminopeptidase P, putative

MSVSANSSAYPFPISMQMYREQRERLGASLRKTFPDGGHAAVLQAASEVPINSTDCNYLF

VQESYYYYLFGTEMPDAYGAVLEDGKGVVFIPRLPAEFATWMGRDPPPEGVKAQLEIDAV

HYVDEMEAVLKAHGVHTAEVMQGTNSDSGLSVLQAKLPDGSSLTKSTSFLFNALTAQRGF

KTALEAEVLKYVCKVSSDAHVTVMQMAKPGMSQHHLESTFLHEVYYKGGCRRVSYTCICA

TGHHGATLHYPDNNSPVEDGTMALLDMGGNYRGYAADITCSFPVNGKFTEAQKIIYNAVL

DAHDKVMRAMKPGVQWVDMHLLAIRTTCSHLIAAGILKGDLEVLMQKEVMQYFQPHGLGH

LIGLDVHDVGGYLDGCPSRPTKKDCCRLRTARLVEEGMYMTIEPGCYFNEALLEAAKKNP

D

>Lp_000033700.1 Cytochrome b5-like Heme/Steroid binding domain containing protein, putative

MRAVVALCLLRLTCANTREAAHITSAVLLTIPLLALGCLLLATVAGVTYMLLWSDGDADV

GQKTKGVRVLHSVAVGTSRAERIPRAEVAKHNTKQDCWIIVHSLVLDVTHYIPVHPGGES

FLTNSAGKDASTSFDTVHDLGDIELYAPEVIIGVAAD*

>Lp_000033800.1 Cytochrome b5-like Heme/Steroid binding domain containing protein, putative

MEDTILCDVSAQENPKRPPIPITPISRAEVAKHNKKSDCWIVVHNLVLDVTEYMPLHPGG

ERTISVYAGQDATTCYDSKHYPDTIELYAPEVIIGQVAD*

>Lp_000033900.1 N-acetylglucosamine-phosphate mutase, putative

MLSEAVLKSLAQRIDSHHSLRHDPKCKALAYGTAGFRTIGELLPPVAARVVAIAILRAWF

CGSLQAVENCTATCSVGFMITASHNRAADNGFKIIDTDGGMLAASWEHWCTDAANAATGA

ELVAVLHKCIDAADVRQPPEFKSPCGIVLIGRDTRESGIAIEAAVVDILRDVLHVPFTSF

GILTTPQMHFLVAKANEPDSIAEHVRLETYYEQILSSFEELFTYQTSVRHSGVSPPQRVV

VDCANGVGACGMRQLLTYSQFRSKGDVLSQAFSVALVNDSTDNPAVLNESCGADYAKQHA

IPSEKMQQWPHLDRGASDGLETHFYCLDGDADRVVAFLHGGCDNDNWVLLDGDRIAILYA

MLFHKWLGQEQLKELDVGVVQTAYANGASAEFIRSKLRIPVYIAATGVKNLHPVAHARDI

GVYFEANGHGTVLFSDKLVNASSAGRFAQVAPLIHNLRCLLSQVCGDAICDILMCEVALR

ALEMTFQDWVALYTDRPCKQTKVSVRHPKLITNTPDEQRALSPQGMQEEIDTAVSTALLK

CDAARSFVRPSGTEPVVRVYAEASDAAVCESLSAEVCSIVQKYCN*

>Lp_000034000.1 transportin2-like protein

MYTPSRDDLVNVIKLLHNNGVEVSNSKAAYAELKQYEANPSFCILLSVVFGAATNPVADL

PLPVDWAQYRQLAGITLKNNLATARHALGEDAVREAARHGLQALRNPPDARISRTAAQIV

VKVIALTSFKWWSSNGFGDLPSILLNELLPAGALKTLSALYCLQYLMEDLPKQIGASSEH

IIVKVSQLVLSSNAPLAIRKAGFRMCFNIYEQASLLDWNVETFSPLQEGLSKASYFFANV

CTSLLESSCGGDSAFMILVLRSCVFLLDYFDYFPQITPQDCQRYVTFWINNSVQLVCNSQ

RGGEVNQELVAAAIDLISTVVDLYDRNGGETALRFLVAGIPSLIPTLVPALVQHSLLSNE

EISNIMDADDYRIRDTTAVRFTVKGGTKDISQDDMLDEDAAAMTLRSSALKCVDVLSSFS

SDATFDSLIKRIEALWVSADWRAREAGIVLVGTIANGCTFELRGILQSLVSQLLEFVNNP

SEHVCVVSIAAWSLSRLGDSIVTSLPAAMNTIVPLLSSRLQSTSKRIQITTVSALNVIYS

VLENNGQLGLLGPHLPGMLESICSCLPVYSTNNLAVLVDLLGKLIPLLDNAAAAEKLSSV

IQTERQNRAAQFEETYVALYVRGEPNVLLNKDIFSLDRATIGFLTAHPNSELATSNLATW

NGVLGDIVNRDVTDDDDLVFNTLFICSAYVTSVSTAALAQWLKATSWALPTTTMHFLNTT

DTYEIKVAAVTLLSAVIQVLGPDALPSGAHDSLLNKAAQELGEADDPQWKEISVRLITLI

TSKYPGDLSPVASDALKAANDALRSDVFGESAYHFTQMAFDLCYVLEALPGYTPYFQIDA

MAQLMAATDNSQEKSDATIQLFRALVQAPAEVFRSHLPTVIRIVYSWQQASINYPETRET

IQLFLRRANEICPDLLQSIFQSLPAPFRDMITSFYQ*

>Lp_000034100.1 metallo-peptidase, Clan MA(E) Family M41

MRRYIGAVQLTVSRRAIVVRFKPPVASQKQVTFVLSPTFQQKRSYYTNNGAYPPPPPPPP

QSYYQQPQWTPYNGPYQGPPPPLTQGGLPYGQQPSPFPSDLGTKDRPVVVVSAPQKASWA

TRFWMFLLLGIGISCFLSLIEEFNDRFQEGMPSSKSGFSRSGLSGMFGAMEVKPVNLDNL

EVTFDNIRGCDEAKKELEEIVEFLKDPEKFHNLGGRLPKGALLAGPPGCGKTMLAKAIAK

EAGVSFFYATGSEFDEMFVGVGARRVRELFAAAKAHSPALIFIDEIDALGGRRSRTDQGT

SRMTLNQLLAEMDGFDSDEAVIVLAATNTPETLDKALTRPGRLDTTITVDPPDMKGRAEV

VQVYLDKIKADNTVNAMDIARGTTGFTGAELSNLVNLAAIRAAVLNKPKVTAEEIEYAKD

RVMMGAESKKIIPEEERRVTAFHEGGHALCAVLLKDEGADPVHKATIVPRGNGIMGLVQQ

QPDKDKYSQSKRQCLARLKVCLAGRVGEELLLGPDDITTGAGSDFQQATNMARHMVRQFG

FNDTMGFVDYGTADTAEGAYISDETKLMIEKEVHKLVDQAYVETKKMLLEHRPELEIIAK

NLLKYETLTGKDIEKILHGETLPERPPRFAGAGDNKLSSPPRGGSAEAKDSHHTIPIS*

>Lp_000034200.1 hypothetical protein, conserved

MMRRGRFGRSTRCFSFTASKCGATVVQWSAQRHQSQNSLGTSRDRQRGQPNADPNTGSQM

SVVESPDLTPHIQFGLNNLEHVKLPNMSDEARRRSDIDKERDMRRKFGEYGPGERLTKSS

QQTRRDMLEEQQDVDGLPWEVRWRKSLIPTSEEVIQDIRDRFELYIQDPIEREQEWYLHW

KDRSFKIQKDRMIWPQGYTDYLDHYDENGRRRVLPTDQRWSDSSWKHLADTRYKDRMWLI

EGEERKAVHQSLQTDRALMEEEEQRQSEMGDVYHGIVSGIVDLDPEQTYQALSGTADPLE

VAKTKMKLQAYGAGQALLAGKKQLDPSQIDPISGFPKADMEPLASGITVEQGAAIATQAN

IQYEMFEQGAEASRQAGYDPVVALMKAHHNTSKQYGDRPILRTQVEANIEKVKQLEAERK

ALEAGEVFRLRSDAEKTVAQIEESILAESSTGAQGAAKDGSLAPGREGEAEAAPTAAAPS

EEGFIHVAERPSNAEPDLPNELYRVPQSKELSEMPKWYRETLVETEPLIREYDMVHDPLE

RKQPEKEYYVPPPLRDSERVTLDDAPDGVSAFDEAKMHKLKSLPELVAGYKPLPLFREVA

KDGTIESAAKAREEEENAQKYVDKNGKPLKKSERLRLERQERLRKSKLTYDSDILLPTLP

WETDSIVDPYRGVEATDAVEFDKAKLEKTWRAYRESFQQALTEFGNLTQVTTEDNCEKML

FETMDRFRAGAVGDHPEIPLEQEEVFRLIFEAHTKHFLADFYKFKGNRKTEANTMKAEAD

EILRRASAKSKLLGQNFMNFVQEMTSIELDTIRKNPVQRYAVMVRDRKYEPISKPFMKWI

RNELGEFNVMEFQSLEQLETHKEFLHRHLNDARVRCPSRIDGVSDSARDAAVEAFYQWCR

GALAFYAGKRLQRMYIDFADTRFRSRAEELFEDSIELFANYAKAGKDIVQIPIPDSDPPY

EYEEKEFLDGESAVFAEVSGNLDKAEALWHSGTTKRWYPMTDETEYMWFNERRGRLTLAT

DISDRCLENVNKKTHPSIHPFPERARVFLEGAPLTQETAEHLWLRYMGDLYWEHSEFMQR

QGRFQTSRSYREKCLNLYHLAVRTAHERLPASWKAPLLTEAKYMLRVYEPSEHVMRHLQD

VETVVEKLFAEGEPRHWIEPFSELPYRLAVVHANLPAYGEAITKEVNRRLKWQPPNELLM

KNMEQRARGGTVLPELVPYFNTVNAVAESFFRRKILQWRAREHEGSDPFLFWTHLYFAFR

VYPQSRDEVQQMWDMYEPAYLYIYTQCALASRVRGHRLINDALRRMCQILLSGDAEQKAT

LLKELTKVREKMAHIIDRKELDRTINALEAHLEDPSQLTYTYGTTLLYKDAVLHEKVARN

PLLRTQMAAKDYADLRKRQEQLRVNGTTLNPQQAAQLQEQQGAAEDEPIDSQADPSTKDM

FAQH*

>Lp_000034300.1 hypothetical protein, conserved

MSLVKAENDDSFVAEVAGEVQASCKGMNEHVSAMIKEYLQRRVEQARRDAQQECNVQVEQ

IKQTVATAESERAAAEERCAFVKKQLAEAAETLHRKDARLRLLGCFLSWKRGVERRREQC

RLAEESSRRWRCLAVYQAYLQWRLFAAVRRGTNVEADELKKRESREQELLGQLGAYRELL

KTEQAKNETLNEKLKEAFVRGMSALNREAVQVLHGSDDKQGDDDVQAIAEILSRSGHSQK

LSTSRDKTESMTHSSAQHQSICPVHQVDRSGHFYHRCYAPGYCEYYARHSRSRTPPSLSS

ESPALPSPFVVRADPQAVRNFDAGVSVPLRHAPKPSQTRWKM*

>Lp_000034500.1 hypothetical protein, conserved

MSLSQAIDLLRKVYTCSRNDERRNATLQLLHWEKGLPGTETVQIGLGLLKSANEGLAVQA

FGAVLLRHGLHARRIQPTQLPLMDLLSWYCNEPTLAPLLRTDLLELIVECVIYSEPAVVD

QVLSILCTDTDSQPRRMLLLSELTVAFIDPDFDRMPRESIGALKRSITRRGSDLLRAATV

SLYAYYMAAGGEQSTALASNTEDSVEAALALVAALAPQVPFPVWQEHNLAVTLEVLLRWA

PAQRQVITCATGLLRAYSASDTHKAAVLQVLLNIVLRKIPALAAERDIATLDDVLNLLLD

LPREFTQLEGVLLIQSVLSVFSLPSIQFAQQAVQLMERLDDSTFRQIDPLSVYSCLRPLL

PKDLCHPTDGANEEGRELSREQFGFERLFDDVFSDFRRGASRVLTLLARLYPERTNQYLL

TVLSELPDPRGTADDPRTPSGFVQQSSLTYIYWEATQFMMECLSAAFEYSAVNVNACVSA

LLERQPTDAVLRPPYFNMISYFWKVRDDQALNVWEGTTTILFNCINDETSRQMDDLDVVA

ARRRAHTLLVQLCVEYGRRFTPLIGSMMKKLEPMLVHSNGMERSLLYEALIALTAILPPH

ESDAYLRTIVNPLVNLLSSNPAMTDQGAFNRVICAATPTDKDDRGVIQGCVNTLAAVFRR

CQMTPYVVEKATQLFPLVGRLLLFIHSIQRSELPKEFRCIVDQGAEERDLYLPGNQRRSD

AHLSGPTRSARGVLMNMRIALYQIFGALSPILPAEQFSGLINALMTTSALPTHVVRALTE

KCLLPIGKEHPTLLVYVFQLVTHFFVQRTEEVRQQRLLSSNQRNSKDEVAESKQWLYYAK

DILTFLRSNVLDSQSWQRSKNLLLAAAELAMSVFESGADTRTAERFLLSLVNLNTDASAT

PEVQASLSEMRIVVYARLVSYVTQSPVEELPSAAREQVAYSISEPYVKWFPNLAPALTMA

GISLEQQETLSAHLLIVGNVSGQRRKIKDFLLSVAASNLNPSDKA*

>Lp_000034600.1 hypothetical protein

MAEEHATGVSRPQSQQQQQQQQQQRPAQAPTDTQGFFNGLFSQLYGSPRSLPQRDSNVGF

DASATPSVSSQDGFGGRSSTTTTPSASPTTATPNAPHTAEFNSAPLSLAAVNEKKNRVHN

GCRNGSRQKPVTASALDTAAAAAATEANADVAASGQKKAPFSYAAPPRGPDVCAPHMLGY

RQCLEVNPDFKTNCTWALDNYMRCKEDMEM*

>Lp_000034700.1 hypothetical protein, conserved

MGRSRSSSGGFGGFGGFRRSSPHASAPHPPPPPPPRRTEVTNIYVQRPMYGGGGSGMLGT

MAAVAGGSVIGHGISNYLYGNNNQAPTQPAEAQQLAQAAKQENNACAPQLVGYSKCLEAN

PESADSCKWAWDYFLQCKQDNPAQ*

>Lp_000035200.1 aminotransferase, putative

MCNAAGPPLKKRKPQPLPATEPFPIYLDHNATTPLCEEAWQAICRVHKAWGNPSSTHPFG

LAAKYELEEARKKVQEALHAPTADSIVFTSGGTECNNLAIVGGTLALRRRHPQRRYIVST

NVEHPAVTEVMKFMEGTSTSTACTGASDAAESHKSALVETVRVKVNPRTGQLDPDTLRCT

LQSLPDGPAAVAVVSVMFANNEIGSVNDIKALCRVTKEVCGNDCLFHSDAAQSLGKVVVD

VADINVDFLSVCGHKFYGPKGVGALYVKPGVKVSNILFGAGHERGVRPGTENVLLAAGMA

EALLYACKNIDAFAAVMRATRDELLCVLKAELAPHNMDLVINGDQRVALPNTLNCAIFKR

VPNHKTHEPVTYISAQRLILSVGDEVCMSAGSACHSTAGEGEEIVVSDPLKAVQVGVERA

IGTLRISTGRTTTMAEVRRAGRIIARKAAQQFAE*

>Lp_000035300.1 RNA polymerase-associated protein LEO1, putative

MDGTPSTLPAVAAPAKLDERSATTNSPFAAAAAAAATGFEDDALLADGGGAAPSLSALTM

QALFGPAFRVEEEDQRAFKDPALIDTRAILQELFGAAVREDDVDLFQEESYDVNKMAKEM

RTNKELLVYAEVERYFGAASTQVLRSDEHVPFTLLESALPPAWVADRTKAAAASWLLEMP

AIYPNRQTLHADPRPCDPSTCDYLESNKFALYTPSNVLRWTMEGSACASNTRMVRWSDGS

VTLHVGGDVLTLNPSQEPSLHLLGESLEVGKAGMEIDALIGSVMPEKHLTAGLGGAVSIE

AALAQERRQRELDNSDRNLPFADLSMPPIDWSRPRKGRTIQEEYVREEYENREKEMKRRI

KEGRPMTLTEQLRLEAQLQEHVAGATAEELQAEREDALRQATLKAAQRAENRGVKRSRFD

RDLDLQGGDTAGGERFGQRDPFLEDDGEEKEVGVEEEEEDVEASEEDERSDDSFGRELAD

MYARNNTRDDDAAVASRKRVKTEAATTSRFDGLAAALRSLLTQIPMNAEAFASVDGTLSF

LGMDDTPDDVVKAEVPKMLAEVATELPTVNTQRVQEELAALFPGETF*

>Lp_000035400.1 hypothetical protein, conserved

MTKLVRKLKQMAKKRAHRKTVQKRKIERAQRELERRNAQQSTKLEDEVDREMARLQGELE

NGAQRPAGEAAPEATNAAVKRAVRILGDLVLDAPAKKNKKKLTRKQAKRKEKMIERGTAV

SDSLSKKWDHKKRRVKVRAQIRNEDLHN*

>Lp_000035500.1 protein kinase, putative

MSSTFFDTQGNKFLIECVLDSEHDSYASLFTTAVTYRARHVSADFDDTLSLQPQQPGGSI

STAIKANHLIADTVTVTYIPLSSSVQGIAASEESAAFLALRDHVRLRRQVEHPFLRSLFD

VFYTQQRLAPPVLSAPAAAEVRRPVETNSPTAARARKPERHAVDRGKVDGGFAANGGDAA

DRGDAESFPSVTTSPSSSSSSSDAFAALVLVEEFVEGCTLADYADAVVHKRLRPTSAKLQ

RDAAAIAYQLAQLLHYLHGTGHIQCRELPLGNVALDQNKGFVSVRLPLSAVRVLQEPGEK

CLSDVVAALVSETSQLNRESGRCWTEKPRLLRAPELRGAAYWDAMWSPASDETALFRAAD

VWTLGLVTTLLCTLNHKLFAQTTRAERLGVVGAQIQHLPELLPHDMQEDMLHLICSCLEN

APERRPTVAGVLKSAVFVTNRSHTKLEQHRAVISIAEAVANANKRKMPPVALQLAESSAT

AAARTAAASADSELLLLSLQETAWDFTPMCRDVFSPAGGTAARPAKVVIPYSEQVAASLK

QATQDASQDLERLCVQCARLYQVDPSGAAACTASCVKDDSLRERLRRSGQMPRDLPATIH

IFEELTERFAQLERSSPDASLRFIELLLEGFSSSPQDVEAVRESVTLADALLRISQTSPS

AVVEGGRGTAEKTDTFDGSTLSNSSAVDQRDFQVADVLRSMPSMPERVIASDRAANTSAV

LYNQWLKKERKRFVKSDGYY*

>Lp_000035600.1 Sybindin-like family, putative

MTLYSIYLFNRYGDTIYTKQWKRTSAVQHGEDGLVAGFVYTLQHISSQLSSTQAGGLRAV

HTPLYKVHYLETMTGYRVALFTDKGMSTALVQGILTELMRDVFTNTVTKNPRYRHEKGML

ITGSEFEEDLEKLFRQKKLL*

>Lp_000035700.1 SpoU rRNA Methylase family, putative

MSALMRRATAQRVEELTVSSRSHVADKSGTLAKHVVAASRRFQSFLGEQRSVARLSKAAY

RHPLAPKDPSQRNVAIPSTAGRAAAAVASGQGSASPFTASAFSASLSAEELKKQAQKALL

LQMQQDVLWDGTVIDDTSNKLVQHFVKLSSNEKYRQARQMLVVGGRAMIEELCRAGFRPR

HLMINAGKPIPNWARDQEDTDVVLVDRKVAETIAPGTDGYVGDFEIPPPPMKEQLIANHQ

RLNRVLVLDNVDDPGVLGTLLRTASGYQYDAIITTNHCADLYDHRVIRAARGAHFQTSVP

IYTLKEEDGDDVYGVLNHIVERNNLLPLCYAAQDDNADAAAATPRSGGDSKTASCVYQSS

VVGAAPVVLPPPRQSGSLYAAASAAKAAAAASPYAGAPSRTSLSDFCLDRFSRTTAAEED

DHGGYVLFAGPNHKRNMLRRLTARVARTTTQLLLDSLPAPTEAPSDLLISMSVVLHALRP

RGNWDYLPVDAKHAQSSVKLQTKHASVDIGVNRLQMSVHDINMDEAEQREKAHLDNENMR

WRRLSRRCGSDYDHWMDAEQRRVKRMLDDEQRRRASPWQVKRKLKTRPMADWVPNIIDEY

RQSLGRDALTREREISSEFRRPPPRK*

>Lp_000035900.1 dynein light chain, putative

MMQGDVDATTFSLRPDHQHKFRPNELRPIVKAVLENRLEEQEYNADEIQSISKEIADTVR

DRIRATELERYKLIVHCMIGEQRGEGLRTGCKMFWDSDTDNYFEEVYVNKSLFAVVTVFG

VYQY*

>Lp_000036000.1 Poly (ADP-ribose) glycohydrolase (PARG), putative

MKKNRLQQQTLNHFFKPLTTRYSPLQSDSSSGPHAVSAEVINVDEEAMSEMVCRAPLPPQ

ASQGAAPSTAAGAAPRDEAWLLGQTQVIFPWSPLNTRVGANGEARQVWDSIQHILREPLR

ATCNEDLRSLLVKLDQLMNGPGNTFEPLRRAVGKMSAAEQTYLFDHVLPWMKERVLHGPA

LFENRTIPLLVQGKTQRVVLSHDEIVSLMCCCFFSLFPQRSEAGKRGGCGRGQKGSRAYT

RAVGTTPAKASDAGAAATHGGVGGPLTTWVSRKLPCCNFSSLFSCGAVGRDACLDSKIRG

FIEYFFCCHRHDADPLSPAHNRCLELTRASYVDFPKFEESTQPLSAVSMHRTGLIENDAE

SLQVDFANRFVGGGVLRSGCVQEEIRMTLAPEMVLSRLLCEELAEAEVLFISGAPNFCEA

TGYAESFRFKNGCDPWASQVEGDSRCSSLSYVPQLRPAQATHPRTQKPMLIHDVCVLAMD

AHNFRKHGRQAEQYRWPFLQRELRKAYVGFHGVPESFAVLPTARTGSIASGHWGCGAYCG

DKELKLLLQWCAAAEAGGRPLHYYTFDTPLNDFDELRDKILSAGWTVGKLVQTLIQYHEY

RSKVPESEAMRPFQFVEQVCTVVQQPSSY*

>Lp_000036100.1 hypothetical protein, conserved

MWRSQLLQCEAQLRELVAQGVHEPPSMDRITQVMSLFDEVTSEVEAHQGNSFLGSLLRLF

RVEFCRAIFLDGSDPTEGGASLSVAEAAAGAAAAASARTMKGCGGSGGARLLDEDGLPQT

YFDEAASLQRTKAALTSALANGASGETLLLLERELEDKREQVTWYEGELNRVRTQYEKVA

EECLGLRQTAEAQQREAAAVQKRLQQDVQSLHVENKDLQIQLFRLRKQLTGGMAALLKDS

YRQLKLSKLGLTQRLFNEGDERVALLVLLSQVESRVNEILDSYDNDFVLASESGRQKLQL

KMAQTVTVLLEDMHYCEASYRRLVGAHQVKIADQDRNNGDAASEQGEEGDRESGRTAATP

LQRIPVSVVHSGKDEEAPDESDGYVAILFDPKIYEEFQSRHAVRARLLQYKKDRDEESAK

ASRKRALDEHTGFVFPTTDSTFLTSDRTAASTAAAAAAANNVVTQRETLSTLLSGMFGAD

PMASKSKTDPPFNSSSASESAVNDAAMPLAAADASAAAAAAAAAPSQDERLKKWSLDSSV

VLNGGSLSARAAPQLGQGAGVTQSKQERSPSPAGPNKRDGGGTSLATAVPATSAGSSQAQ

QGRTTLSLDTAAAPQASLEDVVERQKAEWVAHILGPSSTSVAFTVPGRHPEKGDVVVVHE

KAAEVVPAELMMRRVLHHPMEDLCNERFLSTVEVFTGENPTQQRLLCRVNHVDPSVPIRV

PETTNFVRVKYALAENAVPPGTTTRSANTATGAAAAAAGEAGANTTSAAPTLEDVPPGTP

VFRGNPQLRLFKELNNKTLVEGNLGLQYCAGAVNSTTASTVAFQRLHPMSPNRGPEWLLY

QQLFGAYRSLNPRMMEVTTIDHMMACASERYFTRMEYRYDECYLRAAGQCSNHQLRHDVR

ERLFKDQHTLSDFQEALVDELEARYGYPELVAKTLYEMLCYLNAMAEKDTVLAAYLDAIR

GFAPPTEIHFMSYMLYHLSYCWPESSPSSAVPVEDVRTVLEYVYRNASSIMPIKPDAILK

DYDVATRSAPLNFVNFRQFIASTMAHQEESILLHLYGLFHTYTQHAVVDGAGWDMYTAVI

GKVWRQKDERRNLVRYLTSCLGVNRSTSPTLPQLTLLAASAWSSNLWE*

>Lp_000036300.1 Leucine carboxyl methyltransferase/Cupin-like domain/Cupin superfamily protein, putative

MSSLADAAEHPPAAGRMPVSKNNGGNDAELLSKKKQKKANKVTQKIKGLNGSVDVMVQHT

NDDSVVSKRSAVAQDYIRDRFLRYFVKKPSRRSPLINRGYYLRMAVMTDLVTRLLEVALA

APERAAASPSVASAVPGLSHTPVSRPLPPVQVLSLGAGYDTLAMRLLLDSVDVLPHVDET

HKAAGPRTTTTAAFPRGDVLFVDVDFPAVLQSKAALMSAAPPHSFPAEWVLTPGDPNRPV

TSPHYTAVGVDLRVASAELLSRLRRYGPSNFTTTNFTIIYAECVMQYMPSEDAAKLVELL

ASAFPNAVFLAYDQVSPTDSFGRVMQQSLQQKNSPLLGIQACPDGAHMTRRALASGMQRA

WWANFYRVSRYVLSGTEKQRVEALEAFDELEEWSEMCEHYGITMATTRDLWNSVLTHGCL

VHETFVEYDGDGVPLIANTTPTTTTAAGRAALPLPGAAERRAVSAPTSSSLCVAGSLHNW

PSARYGFEGWGNGGVAVEPLRNGDRLLVSFGGFSAGKQHQRVSSVFVHSLQEGELRVLTA

GEDSTTTTATTALPSSAVAASTSPVTRLPQALVFHTFSRIGAGLYLVWGGRTNPAAATSD

AYLLALDIPRTVSHGGTVCARWTPLSVANADAACCPAPRYRHTMVCLSSLEDSGSAREAT

LLLMGGKVTTSGTLSEAATLDCYRVTVSPQDASISYEPLVGLTGSEAGVVPSAMHSAAAV

ALSADKVLFSGGVLANRDACDNSLWLLNLSSQQWTRLPIDLGAGRFSHSLTRLTVNHHDY

FLMLGGSTWEEKCRVAMGLLIPAEIVNAEQSAQPILPLPLTLPSDVPWWSRHSCVALGEG

VVGVLAGGYTCFSFGTFASKPQLLFLGDEVDESAWRGSGAAPAAAAASTGAADAAADAAA

ATRAGGPPDTSAGAQPVYSYDQLLSKPWPAVREVIDYSSDAFLAAARAAAAPVVFRNVPL

GACVRKWASPAYLKNVEGNATVSVHVAEKSQLLDFVRKNFAFRHVSLAELVQHVEDATRL

YRATKETPSETWYYRSIAAHMKSERSNLWTDFATLGRDFVLPPGAKEFIEPRLHQSCLRM

NAPPLQLWTHYDTLDNVLCQIVGSKRVVLFPPSEYNNLYMSGSSSAVINLDAPDLVRYPR

FIQACKAAQEVVLHPGDMLFFPAMWFHHITTLEEGEAKGASPSATAPPYNISVNVFYRHF

EDASAYDTKDLYGNKDILAVTRLRTDLHAATRTLLSQANLHDGDPGEAAPPIPSEYAEFA

LRQFLQDAEVDAANMSNSRQASSTGVWRGA*

>Lp_000036500.1 denn domain-containing protein

MADVDVSAGFSELKASFVGYRKKETAFTSKQQIFYNKGNEHSCINCFLVMRLDRNSKGEE

VVKILWSYPYTPNTVLVRYPNLGKFAMSNAGTSYTVEQLSYTFVLTDSKGVREYGHTTAF

VNGEAVVVISPYPWCNFFYRIAYLFSTNGEEGGHTMIKALCKCSTPPSGGMFNTPLDLGM

TFNRPYDRLCSFIDTAPLDMLVIFPNIDDLFSILTDLLLEKHIIIVGPNFSIVSNVVMSL

QALIAPFDWMHILIPILPTSLLDVLAAPPPYLVGVLSAQLPHVRRVPVDSAVAVHLGSDG

VCERVDYVNETQDHLPHSGLFSALRTGLTILKMRHPKDQTVRDLCSLFLTYYASLFGEVV

LKGDRGFVSNAKMSDQSRVFFEKLLCTQSFCILSEEVKKALDSENSTDWMDNEFIVAVVR

AHPDIFAAQHAALVEEEKNGGGFVTKYGDCFGSKENFNGFTAVVHGFGGHQLGVGRLLLR

CLCSKWCGPDISDDDDNALYSGRRMGQAILQKRFRSAAGEVPVIEELTETTPTVNGPTES

GAREFEVSVSNAAESSSTRFPSLRAIEEEEEGHASSPSREPQ*

>Lp_000036600.1 hypothetical protein, conserved

MFRRSRLVAGFLPQLKLGANKTDFFTSLWNKPQTRDERIKEYVPEVLEESLEEQRAVLEE

STSRDVIVELGNKILQEIDNKSPTPSIAPHLTKLLEEYGVKDVIAQRPLSYLLYPNSSVM

AAGEQPNELVSEFADNFRAMIEEVEQNGCSVKPPHTPVAPIVAPAEEAEVKEEAKKQTAA

AATVKTMSEEGAASSATTTTKPDENETHAGTGKPNKAGDVIMASKEEYEPMDITMFVKVA

AGMAMANLHCNDLRNAVRCVDAGIAHAKEASRLGGLHALKAGILVRQKKYDDALASAMDA

VAASENAQGYLQGAYALRQLHRVEEAIQLLEQGREAHPMNTQFEAQIAALKKDVKPALPA

SAGAAAALGKSEGKEALTE*

>Lp_000036700.1 tRNA (Uracil-5-)-methyltransferase, putative

MPTTEKDLQTGCSDTDGGVTAPSYMRVLRALCRSITSCEAIRVKGKHEGYRCKATYSLLP

QLQVSPLSMPRVNEIAAAVGVFCTTRSRLSALAPDFFFEVCVKVSRDDQYMVKLSLLNQR

LDCDSIRAAAAATTPESSCSCSRLCRGSVASALSVPCVLSNSGGSSNGDAGTTETLFDLW

RRSSEPHALEDHLRAVHPHLVALVAHVCQPSSSPSLAASSVTPTKDLLHRKPDKGSAYVP

LTRDGADAMVEYTPNGHAFWLSADSFCEVNHDMETAIYEAILDFLRLSSGRATSVECLLS

DRSAVPTSGAESRCACDTSASAPDAGNAETPPSTIVAALPYRRVFICGRDVNSVVRTFEE

YYDATTVVTTCPCVYADTKRNNMAHCIRCTKDKIADPLRAFATGKEGASDGERQTRCVRS

SGSPVESHCLITAGRHGLHPSTTTALMELGAAARLSDLIYVSCNVESLTRDVHVLKETWY

VAHARTFDFFPGTDYVMTVLHLRPVASCDVCGRGGDLLVLPVGLPGTGKSTCGQALENFF

ARPFYGEESKAGKVKSKKSPSSLSASSPSSVLASPPSAQKKAQQEDAATSPACFVPRSFL

SLPQQTLCFRHVERDQVFHEKKLHTGLKAAKQQTHAVLLSALGVWAEGEKDERLNRETWT

NDVTTSPTPCLSQRRRVLYLDSTNGSQEARALYHSLWHVSSLQTHLRDRECWRSERDGQL

TVPCTVSCLVLLFDAPADASELLRRLQRRRLHPSFPSTEEEQRRKLNVLTAALSNSSNGN

SEEGGVGNDVTTACWHVQACAKGNAAVETVEEVVLTVCAHLLFSRELVTLLRGDALQRLR

RDVN*

>Lp_000036800.1 chaperone protein DNAj, putative

MLRFLSASAARKVASLSGATACAAASSTVGSGTTPLRFYSTSGNKDYYKILGVDKNADLK

AIKKAYRKRALETHPDQGGNKEEFAEVAEAYEVLSNPDKKKLYDQYGSEAATNPNMGAAG

GPGGGFGGMGGRSAEDIFAEFFRGGMGGMGGFGDMFGGTGGRQASPTLQPLEVRTRLTLE

DVYKGVTKTIRVNRPQMCAECTGFGTKSKTEKPKCTQCGGSGSVVQQHRMGPGMVQQTIS

ECPRCRGTGTMAKPEDQCHKCHGKGYRTVSQDVTVEIPAGVPSNVTLVVRGEGGTIPGCP

PADMHLHVEVSPHHVFQRRGNDLVVNRDVTLQEALLGLHMPLKMLDGRTVNVETSADHIL

KPEGVIKMSGEGMPSTTGEKGDVYIFTHLKLPSKLTSEQKELVGKAFGVPARDAHASLGN

TVKARVMRETREQLEEQKRGIWASQEGSGYGGGSGSSRRSNGGMPGGTQHAECATQ*

>Lp_000036900.1 3'-5' exonuclease/Zinc knuckle, putative

MLAWSKRLFQKPLAAVSEATATAAATVVSESPLAVYGGSGLSSSSLSEAPATAADTAIAT

AGPSISPLDSVRAEILHYPYEVISTESQLRKAVDLLRNSRQISLDIEAFCTPESVKTPQL

GQISLVQTCSDAAPVVFLFDILSLSVPAFAAALRSVLRDEAIRKLFFDCRRDIEALSTQM

NLVPTRVLDLQLFFTAVQWKLRSVNRRSGMTYVLKNVAGVERQESDSAVQTAMAVGDRPV

WDTRPLPSHFLEYAADDVRHIHLLSTYFPTLTQHVSVEAVERLTAQYVQHYGMGSPVTVE

ADVQPAQVNTAWLERFIGPGGVCGFCGSKGHIESECFRKLNNNVRCSYCGAVGHTSRNCF

QKHPELLKCERCGQLGHTATNCFKANPCKYCGGNHRSENCHQRASLARPSTRKAEKDRRK

VS*

>Lp_000037000.1 hypothetical protein, conserved

MLGSQSWYTALAVPTIVIGAFVATRKRLAYQWREELMDPEGTVYPTVTDADMEYFRRVAP

ATAVEAAETLAAGGRNHSPLRTTAAAPAAAAASSSSLSSLAASAEDASGVPLTTQDNIGL

NVRSDALPPELRNVLLNEVQRWSQQLGNPLDHRKVDAIERHLHATIADDVALQDGNGSSS

SFSSSFAHEVEQRSTAGAAGVSGAAAKSWLSVDFLRSTRVIADHPEDIQAMKAPWGCGDH

MRLEQMPAALRYLVCHTQRVFEGMGRLRHVYIEYSPSGEFYRAPRPPKMYDGHDYVIIPL

RRDGRDTVVTMSPVLRSRVSDLREVALHSWTTRDVDALVPGGCMLRVYGTARYEWGWGMR

PGPAWFGSRLNRIVPPLMAEATSSAPDEAASWRLLRQLPWWTKSPAPLWRRVGLWKEANV

AVPASSAPKDAALIVLHYEGPRSNNKQRSLLLQPEIWIFGRPPSVETYETWYEDRPTAES

VKEEGVVRFMIRNYFDMLTVS*

>Lp_000037100.1 trypanothione-dependent glyoxalase I

MSSRRMLHTMIRVGDLERSINFYTQRLGMRFLRKWDFPENKYTLVFLGFDTEKNSTVLEL

TYNYGVTSYKHDEAYGHIAIGVEDVKAVVADMRKHDVPIDYEDDDGFMAFVVDPDGYYIE

LLNTEKMISKAEAMMKEQGTA*

>Lp_000037200.1 phospholipase A1, putative

MPSVTDYVFSLHYLPIGASVVLPAVAWWCGSGWVTCTALCTASLLVSATAFCLEPLLSFA

PAGGRFNVGLRELRGSLGGMQPPISVFYPTTTAVPESGIEYLPFNDTNYIRGMARYVRLP

YFLLMDLFFIRARMRFDAPAVPLFREDGTPRPIVLFSHGVSGFPRLYSTLLMDIAARGVI

VFAMTHMDASAAYCRDAGNEIHIPLNTHVKWTTAEREPQLDIRVRETRNVIPRIRSGELL

RSLGYDSDVVERYLAMHPSLHLVGHSFGGATMLAVSLEEEKAAKEAQMASPVSSVVAYDP

WSLPLQGKMFYHKLTDTEHPDHFTTPTLQFFSESWMRDKAQYDFFADVQKLAEAQTHTIQ

EAAVIDAANRKLKSKEATWYTRKDVHGTGHLSVTDASLFSPVVFRSGYMTKPPRAAIVDF

AHDTVQFINAIAGPIPTEVDVANDPVLAAVLRH*

>Lp_000037300.1 prenyl protein specific carboxyl methyltransferase, putative

MDDSADTEALEEINRLNRELRRNLILETALIAFALGALTILAIVLALYAYLAHKDALFAF

ALYILTVHIVFHVSEFVTAALQRPHDTHPDAFMVFHSKAFMTASGAALLEFFLEAYAVPE

GWKLSPTRHPLLAFFLRINRAGSIFFALLVVVFYAIRVAAMLQCGANFSLMIEPQRRSSH

RLVRHGLYRYLRHPAYFGWFWRTCFAQFILANPLAAVAHTVVTWYFFRSRIPYEEETMEQ

ADYFGEDYKAYKRRTYVGIPFCAHHTSPR*

>Lp_000037400.1 hypothetical protein, conserved

MTSVFTYGSHGANTMAKKYGEMAVRSSKCGSKNFVYTKSHDDAHPFPRHHHRQGDNPVRL

CFTFQRWRWIFHDFRMFGLFGALRRHYYIGEAWRRKDEKIFVGKDDNGTKYWLSRRSQGG

FHVRIVEAADPHWFRGQSPHTASPMWLKWVQGGAAHTPAQMRARGEWGHNSRLGMPLPFN

IKYNEWSPLNGAEIYSRDPTWVSAPGLLVNPERRALEEAGYSRWVREKGQPMYMPFCGVH

DYSDELVEEFYRGQWAFGRESKGNDHDEWKN*

>Lp_000037500.1 nucleic acid binding protein, putative

MVASITFRLKSSNHVGTIDMEGSVMSYSAAQQAIAAKLCAPPEEIDVFLAGSTSLMHPDD

DLPAYAVVDVVRRTQSSRPALPTRPKPTLLSTSTDAPGSSGLAGGYGGGLGADMNADGQP

LTEEERLAQLQAEAALDTGIDGVSMRHFRGGRGRGMGIGRGGGGFGGPPGEEGGGDYYFG

GRGRGEAFRANMIENFRPPPKGYICHNCGKGGHLIQHCPSAKGGKALKMLSFPVGIPESM

LEECTMDDPAPKFITRDHRLVKRRVDPSAFTAISIAGVTDHRKDDDDADVVGGRGDAVAA

QPLASGVAHEALADEKDHNAVSDDVNGATTTSSSPSPAPAPIDSKYLCVVDHLLAREAMK

LPCCGRLLCQTCFTKMAEEAFDETRSLDDDDGGVVCPSCGEPLLMDDVVPAPEERVQIKT

LLAARKREREA*

>Lp_000037600.1 hypothetical protein

MATPASHSPDYLSEVFRFLSARRRAVVRTRGQAYTPFQSSFHAVHSTLQDTATLPVRTTN

TATDQEETGRPATKTVSQSKEPPLAFQSFSTAPPAARREASKVKVADYAAATRPQRSSPP

PLFSSDHALPSFERSVPHSASQVPLSWSFDRRKALDLDDSVGSFEFPTEQQPDGEFLSPV

LRQEFASTSKTPLASAASLQHAGRAAGNARELGSLGNVPSRDSETNDCLTSVNHAASFPC

SRRHASIPVLEAAAKAARSCSLASLLAQHGAQLFLADDSPHSFPGTRRVSLRRLVFDTDD

DDDDDGNEHP*

>Lp_000037700.1 ubiquitin-activating enzyme E1, putative

MEKENEVSIDRRYLDKQSRTIGTYGLETMTRLISFKVIIVGCGGVGVEIAKNLALAGIHT

IRLYDPRAPTVQDMGVNFAVTEQTLTSGKSMAELSASYIHELNPNTRVRAIEALKEEYVA

DSIALIFTAAAPDLSLKTLNKWNSFCRAHTPTVSFLLAMQTGACGSVFADHGPSFTVKDA

DGRPMLQKSITEVVTLTDKTGERYTRIRYETPEGQIPGALRDYTQIKLSDVQGLVRENGE

SVNGQVFHGVVCPSDPRDTVRVYPALETKGYSPYQTGGFLYELKEVTNLCFRPLQEALVA

PGAFVPVSPMMDNSEESATHLTLHALLRYADQHQGHLPQLHNQDQAEEVVKLAKLIQEEN

KAMPVPTAKRATGKPGKVEFPYKLPPPPPPAPLVLEAYDESSILKEALLARAELQPLASF

FGAVVAQEIVKITGKYSPIHQWFHLSCAAVLPSEADYSGDANFRPTTSRYDHIIAIFGKS

FQKQLANLKIFMVGCGALGCENIKNFALCGITCGQNGSLVVTDNDRIEVSNLSRQFLFRE

ENVGQSKSAAAAARMRQMNPSSRVDPRQDYVGTATEHIFPDTFWQSLDVVVNALDNMETR

LYVDQQCVRFHKVLVEAGTMGTGGNVDIIVPGKTSSYADGGAADQTSGIAMCTLRNFPYI

YDHCIEWARAQFDEMFVSPMQTVHQIIYDPAAFTQRISHEVAAASSAGERRSLIAKNLGP

LKSLKRTLSILADGPTIEKCAALGWDYLFRMFRDRILDLQAAFPRDTKKKNGEPFWSGHR

KFPAALDVTATTVVANPDARNFLIAAINLYACMFGVHPPKPEARFNDEQHRWMEQYRTDA

WLQTEIGKLVVPPYVAGAVHDLDDDLKTDTEDGKEVSLEEEEAELSGLLQEVSALAAKCH

GSKAAPLEFEKDDDDNFQIDFIAAASNLRAENYAIPTQDRMKVKLVAGKIIPAISTTTSA

VTGLALIELFKVLQNKDMSTLRNGMLDVGTNNYVLFERDAPIVNRTKIVTTYLPEQDYTY

RKKVVRVPEGFTKYDMIRVPVTPTTTVAEFAKALEAELNKTLPDGVDYQYEVDGIGVGKG

LLWNGRPSHANTNVPLMQVIERQKTAEANGSLPAPFWQNRTQYCDLSVTVSIDDGDDTVD

EVDVETATVCLVIQQ*

>Lp_000037800.1 hypothetical protein, conserved

MHFAAVDPVSEPPAQRRRLEDEESAEEKAVKARLHNSWPEITVSPESHEITMSETDLRAT

LFDHDRAHEHCLNERWTLRSRFGLHNRFGLSVTFRSVAVVSDVDPPGLDAYLTHACVVNW

SITDHEKRQFYRFNGSDERSPELFSMLVAKKSIRDQPAMLQAVVEQLDSDRLVLPDQLLG

EAASTRLTELDLQFGKNTFKSVTPPRNYRGETCRPIYTVHLEGVSNEHEDPDMEKEVRAV

VELSFTPRGVPPAVDGVRGVMSNGNWEEDEFCYCLHHTKSVTGYLRITRASDNREVARDL

DIKRGTLWMEHSFGGVVPRSVPEARFVRALCRRRIAAEEKAPIMHDRCLIRLYDEQTNCL

TVTRVMAGNTSDVVSCCATVQSGKTRQGYQYNSKVTLTEEMDDAYLSKDTGIAYPTRWKV

HCPMPNGAHLELQLTATLPNQELITALAQPSVWDGTVIVKGKMIQADGKESNVSGDGYVT

SRGRGKLQAEQTLFSMLRGVVTSAVAQPAVAQLHSWEAIAEGPALTAVAQLSIALQTQAF

EVTSSQQIVLAAFVGTYGYVFHHPEDAAQVKKALQWCYSKWVTFFGVSSISYRTLTLRAF

MMQELCDLMHAKCAAWFTPAAEALDVAVPVEYIANEDVADSAAFTLPARSLVQQPPSALE

VAQIKALMDGTWVMDPNETKGSMNAVLMEQGVSVLWRSVNNNAVPTWKISVNSAADTLVI

DESTMLERRSFVIALTGLEWAWESVSRGPVKSRSCVLSGGRELYVETYVKGGIERTWYQF

HNGGRTMVQNIFFFPTATTPKPTASCERHFNIQLPPGSPTSP*

>Lp_000037900.1 glycosomal phosphoenolpyruvate carboxykinase, putative

MAPIVHRNLTPPELVQWALKLEKDSQLSARGALCVLSYAKTGRSPADKRIVDTEDVRANV

DWGSVNIKLSEESFAKAKKRAMDFLNSRKHLFVVDCYAGHDERYRLKVRVITTRPYHALF

MRCMLIRPTPEELANFGEPEYTIYNAGECPADPSVPGITSKTSVSLNFKTREEVILGTEY

AGEMKKGMLTVMFELMPRQDHLCMHASANVGKKGDVTVFFGLSGTGKTTLSADPRRMLIG

DDEHVWTDRGVFNIEGGCYAKAIGLNPKTEEEIYNAVKFGAVAENCKLDKVTHEIDFYDE

SICKNTRVADPLEYIPGALTHAVAGHPHNVIFLTNDAFGVMPPVARLTPEQAMFWFIMGY

TANVPGVEAGSQPVAKPVFSSCFGAAFLVRHGTFYGEQLARKMRQHNAKAWLLNTGYAGG

RADRGAKRMPLKVTRAVIGEIHDGSLDKEEYDVYPGWGLHIPKKCTNVPSHLLDPRKAWK

DVKAFNETTKELVGMFQSSFQKRFAAKASDALKKVV

>Lp_000038000.1 Phosphoenolpyruvate carboxykinase, putative

MPPVARLTPEQAMFWFIMGYTANVPGVEAGSQPVAKPVFSSCFGAAFLVRHGTFYGEQLA

RKMRQHNAKAWLLNTGYAGGRADRGAKRMPLKVTRAVIGEIHDGSLDKEEYDVYPGWGLH

IPKKCTNVPSHLLDPRKAWKDVKAFNETTKELVGMFQSSFQKRFAAKGERGAKKVVPKYV

ETAHL*

>Lp_000038100.1 hypothetical protein, conserved

MENDAKVDALQWEVTKDATPSPKTWVELARATAAMTSVDAVQRANAVTLVYERALRVFPS

SYKLWIGYLQFRQLETGDLCSPNEWFQSVREVYERAVTELPNMPLLWLGYVEFVMASRVP

RVTMTRHVFARALATLPATQHHLLWKVAKKWCSAPAVPGATVRAVWRVFLSFQRSLRAKR

EYFQVLVEKKDYNSFLQECVHLGLPQKLTKDNVEGRDMLLADVNFWETVQTALQAKGWRF

TGDVAELGQLIALGQSRCASPVELSMAFAVFLYGQGYMQEGRRELRRSLEEAPEARTFTT

LYQLAVEVEDQLVESFAVDPAIRLLDDDAYMRVVQHLFSSADPLTHLASLSREFPLLLNQ

AQLRNSPYNAALWLKRAELVQEDVYADRSTAEDLQAVYRQAIQRCTAGMSRVDNAVAQLY

HSAAQQLLRTGKITEAVTLLHDGGWCVPFASTALNVQLVGLWVEVQLMASHAPIAVRDAL

ADKLTTGSSAASGGGRAKRSRTGLLQSSAVLPEVMQHPHVWTLWCDVCAAVSGADEAAWQ

RLIDALMASKALSAEVACYVARKAYEGGHAAVAVAVLDRSMVAFFGYAPSAQLFVLEQHL

SFLCLRHGTQNVPLHQFRELYSLVQQITPAALRVAPLAVMDLHFSCADMEAAIGLYGTAV

RMVQMAALAAVATLHGNADHLRLLQAALEHSISFTERYRGYDAVRTFCGDLVRRLQHPLL

LQRVVLHWAAIEKRSGNVALAHTIMDACSDSQDPSSEHGAVYWRLWESLCTQRTEFENVA

RRRQQASIRFAESTSTH*

>Lp_000038200.1 hypothetical protein

MPSSTIHLRAFELHAGPKTARLVTVSLEKSIAEVYTHLCSVLKLDVPISQVTFHKTFNKK

IDTTPLSIVAPIGSLGLTDNDIFILRQGHRLRSERKESDGTADHSAKVAELPCTSTEQRQ

QPNVQPGCTTAPSEAWENTEPRQPCAALPRKASLNGSKGVLADALTNGELGEMVNLQRSK

VCIATDVRRINTYRQQFAQFNHVSHAFVMKMFQLLAALSRGGLTVPFAAPQRGPTRSTVS

VKDLHQELLLLFCRRARLPFGEADRLLASGAPGMDAVYEALCYYADAMTAPNPSMKTKVC

EWLSERLLPLRCAAQMICVLHEGHEAEYLELALLRLHGQAPTAMTWLCLKDSAAQERRCC

YYSAIAEFGQASVPTHVSAAEIPVLEAVDLDSCCSTWGLNNISYKGHLLPLLSWMWSRLV

AAVMMGRDDSSVRLDEMLHLYSECQAALTWVS*

>Lp_000038300.1 hypothetical protein, conserved

MWALTAAPIQNNEARQLLARYQKQNGFQSNMWLLPRHLTLFGVRALYPAQLLLPTRGLLS

NPPRAVPFALLAPPTQRRILHDCPVPMVPPGRYLFLERTPAVTRWRAATVAECFDAAFIQ

SNTSYGHMQLLCESNCAERLLMPEEVAVLNAQDTSNPFLIDTGLCHRSLVTGATLQDAIG

SVLTTIAAQFCYSSFDWVEASVVESAGLRVRPLATPHRVNCTEKLGVVHVSQLPMLRQEE

LVESIPRYVLLKSQKTSFVYLHAQWRNQSKLGLTSPLRRSDIPPADCDGDPPVDLLLWVA

VTPKDEFSGPITKRERSVYRRFYNAQQLD*

>Lp_000038400.1 neutral sphingomyelinase activation associated factor-like protein

MAVCTPRISLYWLDDHESYLGEASCTCPSTSWLPFAADTHGMLCLATAHIFFDDGGDTII

VFPLHHVEGATIQEAGEQLVLSFSCKRAFKREVLPCSGEQPAMPGVCVPLFNTRVWCFRV

PRVSQEWFTETLRSVLLCQRTEELLTRREVLLQTAPSLISFRVRRQVPMREQRGVLQLTA

THLVFQPLFCLASHSAVTLQRGECVHSFPRWVVFEAVGLDLYTSTALDHAPALSLLFGNT

CERDQATELLQRFLGIPPYTVSLTGVAEAWKRRELTNYDYLLHLNKLSSRCFNDVFQYPV

FPWVLSDYTSAELDLSASATFRDLGKPIGALSPDRLEMLRERAQFLDEAEERTYLYSTHY

SSAGVVAYYLVRSHPEFQLCLQGGTLDVAERIMEPIPQVWRSVTTNTSNFRELIPDFFNE

SFVALCGPPRLALGLHSSGRPVRPYVELPPWATNARNFVKRHREALESDYVSQHLHRWID

LIFGVAQNGEAARAADNLFHPFSYRQLPNKQVSVPGLHLSPHEYAREFGNVPIQLFRDAH

PSRNDSEHGVRCTAEAGECGEADMSLHHRRLAEMMEALQSADEEAEEPPLRPDLSEAEDI

RFATTPATLVEVASTTLACTSVRVVAFGLAAQASPKAATESSAILLLVGDDGRVVTLFSA

ASGERLRTFPDFDGQTTAVAHYAGNMYVFTDNRTCYAISLTSLSVTRSFAELTAAPVVHV

YLSHVAVALADTEARVSWWGAATCTEAASQPLRSLPFDVPPSLTSEASSRVLCLGGATRS

STVVAISATHEAFLFHDDACEACTLRNVPAEAEVLSAATVERVLCFWVFFRSEALFYDSV

GVSLERIVFPAATEVVCARLGEQLYPLCLYTGRFPVRVRVLQRRGENEVQLRRCRVASPC

VTCSGAALAIVGPQTGEGASSLVLTVAELKAQVAT*

>Lp_000038500.1 Nucleoside 2-deoxyribosyltransferase, putative

MIKECDAIIADLSPFRSLEPDCGTAFEVGYGAALGKVLLTYSSDTRTMVEKYGGMEAQGL

AVENFDLPFNLMLADGTPVFGSFEAAFEHFLQHHAAT*

>Lp_000038700.1 hypothetical protein, conserved

MMEATVAQNLGLQLLRQRPKVPQGFDTWAVGVSLPKMANFGQNPCVSALGSYDSNLARLL

SGTVSASDTGTGVAGDTTLSDAFASQPACPSAFSVLTHKCLAVGVRERCTLSQVRIAPEE

FLTRGAAPSAVTREEAAVAVNSFSSAIWEKASETLFYLSLLTLKYETQTP*

>Lp_000038900.1 hypothetical protein

MSRDVVAWKTAFVISCRLVSCARRAVAALACEKLVTLCVVASDELPPRWKERHCCCPGVC

VRGAFSKPPPFAAMTGVDVFVFELLLLWALAGGGETTETLPWRHAASGVGMTSFDPSPVR

VGEGGAAIWAPTVVVFAVRPTLLPVVHADSPAAATASPSSGDAALLAACAGDGPAPLRLP

TRVAAAANGDCGRSDGGIGTNETSSSFTASFGVTGETTAPTPAVWKGLPFAERGCEVG*

>Lp_000039100.1 Domain of unknown function (DUF4139), putative

MPAKISYVSVPEKLAAAFTRVKAKNTSDFTLLAGDVAVFLDGSYVTRSRLDAECAAGGMV

ELDFGVDRAVEVKRVLLRQANRKVLDSYLKGTKKNVKTYPYKVTVPNKKRAFGDDKGTVQ

VKLIEHIPVSSEEHLRVRLVSASEPQEGVDLFDDGADREEQLQRKARALKDEGVVEIERE

VRAGESVEVLFSFEVEWPSTATVYGL*

>Lp_000039200.1 hypothetical protein, conserved

MRLFLTVIPHSSLRAAQDSSSPDTITIFDDGVWTRSEPSPDAFTQVSATFVAQEVQRQAA

LTELLRTSKEDVVCFRCGPRPPPQLDFMLFHKQDLLSLGLQTKTSSVRQQTSAESAGVSS

TLATPTISLSSTRSERSLPLSSPAKSEQRDKSSSTSASMYDSFLSTWLLEVVTVQKLQAT

LSGCLFFVDGTAADLNTQQDVRAPLSTRTGATRILRTADDVHTYLTHCERAVLELTKRRT

SPTPYHMWVTVRVGASPCVTAAAPAADDVSAVTFLDLACPADYTGMLRGGARTRLREQLR

LFGRQLSTASSEATQLPSLPNANSPTASTADRRWLGFLQSCQLSRVGMIGVFCLTQKTGR

NPFDVSPEENGCDLLALSAELRQRALVDAVNNGALHPQHPSTREHLRRLKLQKKEKKTKS

TQLIMPTSPKRRSVSTEAISRSVPQIEPVIESMPQLPSLKHVPSPVLLRNVKSAQSAGSR

SPTNSNNSRNNVANTARLTDLFALLDAEEDMTRGTLIDSEARARDLVWMRWVLRAHPPNT

SVIDVARQLSSAVSRLQRQKVRSAPLRQPPPLTQADLAATARAPSSSRHSNHSSNKTPSS

SSASKLHAAVPARTPHSCDMVHMALPLITLTASPSHVVSSVTSPSYRHSAATSVVTRHAL

RPEVLDDAGGRPRRDGIASAAESPQVSPTLSATGRRRRYSALEVALLKRAHSATQRSSPA

ASQQNGGEVELLYPLVRSFHAEVTAQDAVMAAELMARSTIELRELNRRRTLESLAAEQQA

TAAATSPTNVRMHNGRQYSASPKASRVFFYA*

>Lp_000039300.1 hypothetical protein, conserved

MFGNTYHEEDGSKAAQAFETAVTKGDVGPLGPRILVADSDNDDSLGVPPPPSPPSCAAAV

AALPPAFSKRATKCAAELLKRQQHASSAAQKRRCMQQLCRLGGAYRSLGQGQAPTVWVRY

ADLNAQVIRETEAYFDGRLHDRFASPHISRRNGSLSAPMKPAMQLGAQTTTGNVSSGDRS

PIPRFVFSAPLVAPEETADAGSEGFTLSRGEQFALSLLSMEDGGDLAATTQPMPTYLLRK

DASTLQQHVNIPSSAVMVKGGDAIAGGFSGHGTPLHNCWVYDETENGLSSVQTSPSAAHS

SESSAAASPPCKRRRAVEVLAADKRVRSAASSTRAPTTAARRLPFDNDVIGRSGSSSRSS

VDDDEVSTPSFSATASSIYIPRSLSATVIERQNHRVQWSLLRQQSMFSTL*

>Lp_000039400.1 hypothetical protein

MSSYRCDDNASGYMTVTSAATGKPKRRRVIHCVEPEDDASVETYYADDPKTNDPLRYTTC

MSHHIMARIEDDVKRAKMREENPESAIPSKGYEYDVDAYRAVEEYKATQQLPRGSNVVAA

VLYTPPRTDVFLPAISPPSPRSRRNSNSTSRQPSRGNSTANSPSNQSRHGGPPQASARRR

GQSSRTSSQVSQYPSVYRSRSSSKHANNSNNGSVATISSSPQPRLPAGGRLQPLDQADPT

PPLHNSGFRDDGRVTGLSDQPSKANTKLFLEPESLEDEEAATTVDAERQRRLRLAEQLAQ

QMEAEGM*

>Lp_000039500.1 Thioredoxin, putative

MALVVGLFVLAAPAMAAEKEASRVVELNRDNFEEYVHGPMRHTFVLYCVKWSRQCQTARL

AWDRLSISQSTKELRDVFTAAYVDGDRYPDIIGKMNVQGFPTATFYTPIYPEGVEYGGTR

EPFLLDSFVFQFS*

>Lp_000039600.1 protein kinase, putative

MPSLNNARRGETSDDYARHKADATKAFLENHYQNMLANNRNGRVAVGPRQKEPSFSDFHL

FKCIGRGAFGEVFVCKYRSDTTETLYALKRLRKADMIMKKQVVHVRSEKDVLAEAAASNP

WVVHLYRSFQDALYLYMVMEYMPGGDMISWLCDKGIFDVESTRFYIAELCAAVASVHDMG

FVHRDIKPDNILFGENGHIKLSDFGLSKRFVEKRGNLLDYTDQPSTGAGSTSTDGKDETT

AYASQSDNATPAQTDDGRVGGIAHGRGRDMFQSIVGSPGYIAPEILLRRPYGVGCDWWSV

GVIMYEMLYGIPPFYSQNPNSTCHKIKNWREYLNFPPNSNIPDDAVDFMKRLICEPEERM

EYDAIRCHEFLASLDMDNLHALKAPYIPDLSNRLDTRYFPEIRELSAPLQQSEEQRVREV

DPRGVMFADFRFNYSGRENDSKSS*

>Lp_000039700.1 nuclear transport factor 2 protein, putative

MEQGAFNDVAKKALEFRNRYYKMLDDPQERPNVASLYAPDVPMVCEWNGHPLATVEDVRN

YLASLPKTSHQIDMVDAQPLPDNEGGDSFLLTVHGKVTYSDEHVREFYQRMVIRRFDQRY

YILNDYYRWLSERAQ*

>Lp_000039800.1 hypothetical protein, conserved

MDAEVASSDAVPRFVDLALFLCGTALRSLRGELQPADAVTVRFYCWFLQKVCFLHALSND

DAEQIALDSVADDDHHDIAKLRSTPENDTSPLSSANIRCGSSKEVNFKVASYELRQAAKQ

SVGAPTAEEKADAAPSGAAANTFASVAGHVHARSPSEPSAVHSSYARYAESTIKLVRPPT

DVGFSVLAEFLSIAHEQGGASLVQRMAAALADALSEQTALPGTVSPAEQRAAIAAAASSP

SSFSDVPAVVRVATLSKLFDEDLLDDELSTEAGNATRSFHIWVNYEWASASFLRQTRLAE

VSTRWLQELCYYRDGRRLAAACLRSSSQRPLPLSKSTLSPLPTNVKEMYTALEGVFEGHT

NTRKLLMEMALLQRTSHLTDNALAKLLFLQGPPIAANVSRGELTNRGTQPILMSITGGML

LYAQYYLPFRSLAVARQCVRVALQMAQEVHSNSILALAHYTAHVIAVHQGRPADAANSIS

IALQLSLGSGALDNTGAGGSEGGTAASPFSADSADAQMASVAFAGAAQLLLFFPGAVSAA

LRSVLSTGRLGSGTVFGGEGDAAAGGSASTVPTSQNNNNSGGGGGGAVSAQTVAQSIRHA

VLRAETSLLHAPPAEGRWVGVVAGLHRETLLLIGAVYGIVSTPTSIDDASLKSLLEAVER

EAALTSPLFQAQSRRSLFWEVLRHAAYHALNLQERFLPFDVDTPPSSSATAALRCLAAAL

CALRSHYGDAAVQVASDNIFFTCVVRYCAACRLQDSGHATAAYAVFTDVGQRLCSAAGVE

AEQTSATSTGATTSDIDDTSTQRSSQQQCWSPDHLLLYALAQHKRAKVAMFLGLVAVPPA

AQQSLLSVSAHYNFSVGVLTAQLMEAETCLHNGRYTAALATARRVEQSATRIGLPSLAEA

ACALQVTAHASRSDWCAARCVLQRLQPRNGSHRVFLLLYKFAVHLELLLLEKSVSSHDVR

ALARNWLQLLQREKLLCPSGTAVAGGAELSLSEQVCLCNTVSRAFSLLGRNTSSLEAETE

ARLRKLQGRQGAPVPDVYVQGDCDEVCQHGLSSES*

>Lp_000040000.1 hypothetical protein, conserved

MSVGRRLSSSLGCFDDAETAELFSQDSGSTVSATPPRQESPCDRESELYISTSEEEVRRA

VQHSPTALPVLQCAPDWLPQRAAEEEEEETRSADNATSATTWAADHVDYLTLFVRWRVSP

PLQRHQQQLDNSREWTSTWMPYAAPLSEVFAAAFATKIAAVLHTPNTATTHSSGLDSALS

IATNTTIAATHARQAHLTGSAQLPLGRAAFIDYVSVLGGMQEKSSGAERETLGEAAASVS

RVHCSVHLHFTADRLKAPCQNEINNIDGNLATVTSPRCHSFDVFAVSPFTTLNGVPLHTH

YRYHFRADDVSCCPAGVLSLKLGPRCAVDIALKGLPLFDASELAREHASGSLALHRPPQR

LAPQQLRRRSTLAVASTTTTAAAAAAAIATTTTTTSLFASDSTAVLATLSETSEPVALSD

TAADDADDVVVEGHSSPELLEEVQDDIMPKLTKRQPPTKPAPPPRAAKAARPPRHSAAQR

KEKTEIEGGEEEREPAAPAPDGKVIFTTGLRLSDSEEDALKALGAIVNPPLRFACYARLL

VAQKPLMRSVKLLTVLPYVEEVVHQSWLDTAMHTHSLDIPTESFLYSERRLPGSIESVNN

FELRETLRKLPQDRQRLLMGQRFWVHKATAPQDPPMNDLKTVLTASGGVVTRGIHAANVL

VMPQQRPTLKCWRSLLDEMGCGFQVLAQQRQHGLLLVVPDDIFKCVLQQRPLVHSTVHVP

RAEPTGLRCRSAKGSSGRASENSSSNKSNCGAKSKGNGVAPKSGRRSSQGSRKTPRPAAS

QRSPRPSTRRRSS*

>Lp_000040100.1 WD domain, G-beta repeat, putative

MSSASSSLTVFQEVELPVPPGLFYNNLVVHRSGDYVSYAAPTAIVSVQTATGTPVFRPLQ

LSSGTHVEFLAGVVDPSIENIFLVAALSNRTAVVVVNGQQTNSISAKGPTEIYTCVAAAR

AVTSETVVLALGGSEGTIETLRYTLEGRSTTAAEVSVPVQAHEHRSISALDVEATVSSAN

DVVVATEMVSGDSAGHVVLWKSGSPVLCVPPPDTSDAVTAAKLLPNTSRVAVAFGGGQIK

VLERGSGAAVIVIQAHARWINALSYHPARQLLVSAAEDGQIYVWNVAAPDPHGVCVAHGS

VPNELLTGVVMIGTNNVVAQISYDTMKLRLMSFS*

>Lp_000040300.1 hypothetical protein, conserved

MSASAEAPDPSLDAASHKSSSPRFFGTCVIEDVHGRPASRPLSLFLTPERTYALQRSQQQ

GSAGSFTSNFYDKEMLLSNPNFLSPDQVNFSDNSSNAPAAATAAVPSAAVGADAADAVNE

MRNGHRNGGSNWEMAIVGNQRSGAASQSRNGHTPLASSISGLYITQSGIIIPPIQGIPQP

KGLPAPPPVQYAKLKGKGKGKAGPKRSRLAVKGAFSNMTSVAVLQHVQPFQKKTRQVEAA

GWSRLDFTADDEIDDVCADEENPQQPHSSPSDQMPTAPPAPLQHTASNPMAYETPQTLPA

AAAATAAEGALMEPAVSHHSRCSSAASQTSLTPSEAAMVAAAWEEDAVAAASANQVQPVQ

VDEDDWDRAEEEAMRIRAQMLAQQQQQQRLSQEANAFLRAPVQRSYPPQLPLKQPQETTF

TPKAPTQLRTATSPQQQQQQQQRVTSPSALLKSDSIAEMAPYAVRTAASAKCSPSVVRAV

SPQLPTPGHEVCFAHRFGSAEERHNNGEGVVVGGSDAVSSSVAAVQNIGATAVKQRSASK

TYPVGFEAYAEKGKQRRAEAERAQRERAAKAAGAPLPSRDTDTAPINTTTTTTGAAALEA

SCSAEGESERGRLRRSRKMSVLPPPRGFEAFAQRGQARREEVKREEEQRQKAEEENCKHV

PRINRRNSVSTSAITATTATAAAASRKISLTSTATFEENRGAAEAAAVSTATPIAGSTLI

ATHDAAAQSHSTVFDRLSKEAEQREGRRRQLEQKFTPFFVPQRVTAAAKAVSEGEIALKK

EKDEDMTTAEPMTADAAARPRAPSRNVFEDLYSLSKKRSAPPSPADATGKDQPARAKSAH

TDSAVAADVALGVAVQDDSSEPPKNKPKVSSTAAATPAESSATRKKSSAEVEQYIVSMLT

REEERRARWAQQQVAKAAEEKERLNHPTLNPKTGELAERARARYRVREEQKRQQEAEEEK

ALQAQGQKSVAESLAQRQPRKKMSEQMPQLQRSGVSANSAVEYRKVPHANSGTTTTTPAR

RSFGAEFYEHQRKTEERKYRSLEQLRLQQAEEELRECTFRPRLNTVSEKMAQRMSVESYG

VPDDFEGMSKSATSQEAHYLHGNLPGFTDFSCHRERSPFTSAHSRSLLHELRNGDTPTQG

PLVQDRSSISESEEKHFSLNLQPIQLETASIEQLTDVITPQRSPEPLSTQLRNLEEMLRE

WKELERECSPMLRHRPQAGLVE*

>Lp_000040500.1 hypothetical protein, conserved

MPQEQPDRGRRPREVLPPSPSGNTTTTTATTTRSSSPVDTGASSSSAAGSMGELHNAPFL

HQSRRYMDENVRSMLHQLPSSPKQLLRRLEQQQQEIRLLQNENTTLKSREVEVSSLTVYL

QDRINATQNQIDRIKTQVLLQLSNPITEAEYNRIEAMPEPQRDLVDAMKLGIYRQLNALR

TSQQAATQRAAELSTTLAQVQQDNADLKVRLAEYESQGDSEREKMEKRNRQLASQQARIA

ELEGTLSSIEAKNKSLYVDQEQYLSAKLTAQIKTDEVARLAMRLEEAEMDTERYRANAEC

CEQKLDILKAEYYELKLDYGQRVLKLESALRASEEKLKTLGDLEMESELFISNLAASANG

EVSFDSPAGVVTDTKSGDGGDRTSTYESWLALPRSRKLAHTLVVTKRCLHLENKVSSLEH

ELEFKQTQMARLQVALDGARDALNNINSPYVLVEKAMDELATKNEALKRKVDVLEHEKAE

LQVKLQRCGENMRVLTRHRTELLRIKKMLRQLGMREGFVLPAVDDEEDDDNDDKCGDKMK

ENKGKRLRSGGAESSSLPASGGVQLPTSRAAAAIPPTNADPAAQAGTRPPSDSTPPSFAS

MQPIEIQS*

>Lp_000040600.1 Staphylococcal nuclease homologue, putative

MGNTCCGGSVRTAEKPSEVRKEDYAFFDALPAGVQEATVDHVYDGDTLTIREQNRARVRL

LGIDAPELKQQEPFAKEAADYMKRMCPPGSKIWLRASSNERKDRYNRLLALVFIRNPSAG

MPGYICVNIALLQKGLATFYEPRGSVEYKDQMLKATEAAMSARLSIWGKVNLRKQVFTTP

NGAAFHNSDCLAIQMVKPQNLHRQVMSEALHEGYSPCRECKPMQLRA*

>Lp_000040700.1 hypothetical protein, conserved

MRTRPSFTRTETSRAPPPPQQQPITSTSPTRSPEAKLFYYRAPSRGIPSPTNTLSIDRRD

DDGNAGGVYAVRPSLQRLKNGHESAYGLSHELEKPSTLSPESEKQRYRVKAGEGDSDGFF

FYDPSWTSEDVYDVKSNSDCRRTQHHATTAAAVSPSSLLTREDVENSDMLNQRSAFDVSP

PPPPSLSNSDPSSAREKQATAASHGTRENERQLRRSLRRYQRYVETELQPRMEELRGALQ

EKETECARLHADNVILANRLQRLQSALNSVDSTTTPTTESVKFSPPVSAVRSDDPALAVA

APLTVVTPTPLASDAQRGSEELQERLREASAEVTLLKRQLAALQGRHDRCVQSLGEGSVT

SPPRTTPSASRTLGQTEVSESRETARVELLKLCRALAEDGLGFTAVLRDVQDRLSSDRSP

SDASSSKDGVRSSPPHATNPFKAAVEEWRRFMRVTKAAVVGVSGSHALEGNPPHVTTDVP

TACTTVLKALLTTLQTEHGAVAASMEAVQQLEKHHAASLTHHMRVVEQVRREAERRIEEL

EADHEAEVQTLESAIADLEQQVSMTTASRRPWTAGLFIHGCADETTGVCSPSSLNRRSSQ

EVGGAGGAVGGGGARTTGGSANTNGDAIENARRAAAGLAAAPAADTRAQPSVERCDADTQ

TSLSLEWIQACLQKAREEPMRAVRREKEAELVELMSTEVETLRRQLTDSRAVVTRLREEQ

RRFLGDVTLPVSYFDNFSSFPTYV*

>Lp_000040800.1 deoxyribose-phosphate aldolase, putative

MTDLHMTSRPGYTGQLNGRILERVHFLARELDLPHLEEKFPHLKVTDGSWRKVASFPDDA

DLGEFIDHTQLKADANDAAFVKLCDEAKAHHFKAVCVNGCRVAECVSRLRESAVQVACVC

GFPLGQMTTAMKAAEAKEEVEHGANDVDMVINVGKLKSKNYRYVYEDIKAVCDVCAAAKA

VSKVILETCLLTEEEIIDGSILCVAAGATFVKTSTGFSTGGSTPEAVDVMLAVVGNAALV

KAAGGVRDRSTALQYVRAGVRRIGTSSGIAIVS*

>Lp_000040900.1 hypothetical protein

MQSNYHPASSNNTRSSAHAVDAPSGSRYVHCPYYMPEMSPLAANEGAVGTHDSGLNVYRA

ARVGSLGTPHMSGLWGQSTSNSVRGPYASVGSNSSHHLRTSSYTTEFTPDASPAAAPGYH

AADLDSVVLLPTRPLLKALAKQCRPSPPWCVGSVVPSKLGDEVLRFSDFNAPTQEEESSC

QACKASMEAVLQQVWPGASLQPAGTTAAGQNTTKGVTLHFYAAGTTDATEEQLQRWMRAA

NEVGAQANFIRDDRELPCAIFVDSRTGHRCCIRYGDQAVAALAGTSALLSRSIADKVVAR

AVFNTLLTLLNQNKILNESGTARTMLSGEAVAIMLLAVMNSYGATDVPDAGRVLLDFFLT

YGFENYFNPIQTSVSSRGFAVPTAKRHLNAQLSVLDPVNEEVNVTPLVDHVSSIQAVFNY

CYTALSQYAQINSTLHRAQSALSTIIGGEPYWVRVLRYYQLHVEPYYSVIQQKKPTLIQF

L*

>Lp_000041000.1 hypothetical protein, conserved

MHKGDLRITTVPVSGLQYATSPSLPTAVKAIGSAREPAVVHNTSSANATLTGSLGPLLPQ

STLDLYDRFYSSLTATQPAQHQYQHQHHRSSSNNEHESHSEGDNAGAPQANWSASLMLPD

ALASIGPSARRLLQLDAAQHEDGAATESTAAAAAAAGVVGGASATYGASEAWKMSSVCFG

ESHLTRLPVVPAAARTLPVYTGKMKSASSRRARGAKASNYAATTTLPTAASAAGVAASSQ

SSSQSPNRTVLVSQAPPDVRPPVLSSTITNGGAINAAPPLVTQLHSYIQRELIHSAAGGS

SGGDGALVPSAIEQLGPYREAFRALCSAFPAYASLFSDIQSAYDNVIQAQAELLTDACAA

VAVSDVERNTNQEQVTTLHGQVSDLQKELKSMEEALKTRALAEKQERQQQSARQRPRSAQ

LADAIELRRELEAAQQRVADLERNSQADLEKIVILIGAVRECDRRLKEYERIVASVTGQV

SELDEFKRIAGEAQAELQLFRKKYADYVPVVDFQLMKEYLAAELESAQLQTRRWRRAAAV

RGTQLDIMQCRLATLEEERANMIKAAEAAVDDNSASTGGDAAAAAAAAAQAYRYRLTPRP

SWAKLHAELPELAVFAADAGQLQLTTANDHNDDAVMATGAGEAAAALTRAKGLPTVKGPK

ETALQVEYLVQRIRALEAQIATQRHQQLQQQQQPSAAKQTSTQIADEPSASLRAASVLNN

GPSAALENAESKNRNNSSAKPSAAPTATAAPAATTKPEASALTRRASRRAQHRFSEGLQA

SSTGAHGGGVPALAAPLELPLVGLGYGPAVPVYLRASGVVARRPVAPSTIVSLVYHFFLD

ILPPYMDQQDQLRRDDEDGTKGDGDGDRIGACLHEYLRSEMTTREDLRGYDSVAHLFMNL

IRDGEGSEWFCDALHVLLWTVRGVLPPRVAVDAAVVVAQVRRDVRALAKELQSSRLRRQA

LSECLQPVLELKSPSEVAELRAALGGETTFSVDTLCSDTHPFMEVLLVQECRASADLYVT

FLSALSARATTMPSRATSATSRGTGSDKTAVASPPSPPQLAEGERVVTLADVAAAIEEVE

PQTPAIVVRELSVNAASASGRQDFLKPPASPTGAAAVAVTGPNASAADGSIANKSISSTE

EACVVVRLSDIVRAIAAAPLIRRTLRNSPENCVL*

>Lp_000041100.1 hypothetical protein, conserved

MLFLEPDVDCLRLQLRDIRIPIVVLAQKPDAVAATTATPSTVTTEAQTTKAPLLPHLPRS

TAVPAQRQQQQQQPFPVPTAAEPSSPRPASGAVDAVSSATLSPLPSNRPSATVSASTRTT

ASAAASSDGAHINLEERADALHRALLSALYDRFPYELRDTLEYAYVLVITHHAAPSISSQ

PFTAEELSYLTAHNGFLPTELRCRTFPSHLNAALDPACTAASAVATVAAAAAPSSSPAPS

QQATQRRPSIRVTDDAAQVRQADVQRSAHNAAAAVLRDVPMAPRFYDSFFLLARDLTPAQ

QALYYETRCRRQAVPAEERQSSQPTFSRATATPGSKRAGRRGRRTAATAGEGETERFQVL

QGHFTHFIPVREESASAGHDAFALAAAAQRMSGARSGYRRVVGESEEYDVSPNNRAYTTD

AAGAPTAAKAVADDVCALHFSVYRIPLTRFEPEDQQYYTDQREELRWLQRRRCCASYRTR

RREERRRRTRLGTGNEALAGTGKPCWWPFSRSSAAEVPPLLTPDVQKRSCSSSSSSSVSS

NFSSCSSSDVEDEEEDEVAALGDGITWRRCREIDSVMDQLFSSEVSAATATSALTGPENY

TPAEQRRATRFFERCHKVLTNYAFAHSSLASEEDHADESPAADPTELLSSTPALQPHGLH

FPGVSYFGVSRLLYSFAPCDCYHLVMRDKNPLYYAHSSRASYQPQTVPLCLVSDLTHDSP

RATAAAARITANSTQTRATQLRRAASNAHGEQVYDDDETAGSAKRSAANAGRPSPGPDIS

GKVLWLVNPAAPAATGSLKDSVEGSMAALQDCAGGPVPLSRPAQSSRASSGCEAAAALRQ

SQTMTEGGLVRSRRPSRAPSPERADSSWRLSAHAKRGSQQGKPATASSAACTEGVLRITA

SLLSNSISGNPRRTEKSLTEKGARVPDFLLPDAARRGSGIRSHGDVVAVSPSEAPSSAVV

NLFRALAESSLAQHKLRYGAIRSVISVAVLDSTHDLAGGAGTQRYGTPEATTMRQSTRYC

CRGLIDHAWVSMPVQCTPAEQEAIQWMPVMLGSTECVSDVGLRTAVHLGVFPLSASGGDS

SRNSGGDGVMSEDAKGGVWPTREGAVSSLKPSLTPSTAQTHVAAGVPGKSTPVHPSVETS

ESKNASPQPLQPRVVNSATVPVTKAAAPPAQQPPGSYENQPASSSPQHLSHETADFSASA

ARLARMLQPSDAQQCSTPPRSPPPPQQQQEEESQVLGRHREEVEVDVSVDRDDEEAPPRT

TVTSMRAARGGVAMTFDVGAASTGRPKAPGSIPATTAARMSMTYGGAEDDQGFENANDPR

RAFCASSVRDSRDADYVGGGGAPQQERKAIRWPDKPLPSVDPRAYRIPSPSSPWTSLSAT

RGVHEGSVQPHVSGARGRQSEVETIEMVEDNVDDAEGFTSVYSDKESYEGCGGQYENDTK

SFKEEREVDQARERETERRRAPGTTELFKATATSTTPAAARVAATTLPNPFYTHAQGTYT

TPSTPPRTPRGPPASFSALTAPAPTVPSRLRPSPQDERFFDNPLSSADPSPRRAVEPTFF

DQPAEPKIDANAGPRIEERAARWPPSQPPQPCLASTPRHRSTAATSLSLAALADQRGASR

LSDAVTANSLSGSMKDWSQNVRGGAMTDQTRRQTTDLDAAECFDSPEGRPGRGAVGECPL

NSTADTTIAQNPSTYFESMWGAQKAAHPSADPLLGSTRQNTWVSRPPSGWPTSPHSQQQQ

QQQRPFFPDAFGRQGTTPFASQRAQVRKTLGMGLSAGGAASAASAAAPGSAARHLSQTLC

IDEYDDDAAMKAPAMNRATALRSRVRPRGVSATDAVWWQPVRELSSAPNLRFSSAWPQSR

ATTTTTRATTLPLSSSLGVGTMSRPSVSMRLSTSADQPYNVSFAEFNFGESAHLRKELHS

TPREHSARPTSSVPSFTATSRWTTNRPSLNVEHTYNDEAKDYLCRAHEPFTQEFSPSSLH

SHLQEPTRSSALLGRCPPLAPSFRNASSHRYSTAAATTPTAASSRTPCCHAYSTSADRVE

AERHRRALRRQQRLNNYQQHLQENYMCGPMPKRFCFERPPLHPVSATSDTRDAPTGGIQV

LGHVFGDAASGAALPRNGTLATQHRFAHAATQAAAAAASPVSSGAWTPRTTASPSCLPPF

SGLHSDEKATSAACANTPLSDAARAFSLGVHPALTAGGGGGRGVSYGAQQDGRGNETSDV

PVLFRGGHNAEQAAQAAETRLREQQEQQACMTARRQMKQLDDAEAAARLSSGVNVSFTLR

DIAAVFLRLPSTTMRASRRSAADSRAVCSDGDTGLEVLDICKAAALRTEGVSCIISRKPT

QMRGGWESYKERLNLYQEPFYLRDQAFILLLPVPHVPELKVCVAIHNVNDVVALVCQTPV

PRILPTEEEEWMRQRRYGWGSDEENDECDKAGDDASGGRPQRAPGKSNNGLSTVTIEERR

WFFGLFRSRRVVQESGGSGNCGGNGVFRTSAVASWRQRQQQQSAAPANEDAARRATCRAL

RRQHHAEGLCLTWRHRMHIWYALFQAGRSCMSLSACTSPLLTDPAACQARCALLCDRVAA

YAAVCHRLETLQAIRQAYIERRAEELSRQRVLKLCDAPYLKSRIRLGESEGEAAAMTKNS

RSAGGEKHMRDEWQPYSPGQPLLSTLRGATQSEAHAKRSCVASTVSDRPSSTWSELHGAH

TQPPAAAFTRLATSHGNYGSREVGGSRYERDAANDRHRAESPAPPTSHFDQPKQTTQGRY

TTAALRVPSARPGTTRAMQLRCDASRRVAQGEAPVSSLRHATRLFAFSGDDRVDAGVSNS

GGSGAIADAVSAAAPTSGSYLFPAAAVSSVAPYASSVSIAPSRHISTQQQQQQQQQLDEG

RAVLAASGSTPASRLALSAAAASTHMDLILHLDRDDTVARALNHLAQVRATSIVPPLTTV

LSAVPRSTQLVRGAGGGIREDSNVLPATAESHDGPSSSSTPEATTVTDTAPLYAGFVPSP

VELQRRLTPQPFDISLGQVQQLLQWQWEHHQHSYTLMPLPLFRGAVGPSVDFSLFASTRT

TEQCGAGKANAAASPTYAVSPLARELILQLVHWSWLLLPVDYSCRRRGNSHAPRAGSSAF

FPRGARGRSWRLRRWRLLFWLTAFTVVAQHPAGCFTLMLGIAALRLAYNGLTVGLIARLP

SHRRSRDGAQLHDMACQREWRPRLLLLCGPRIRLPRLFHFGFARHHRKHQHCYCSSHPPS

FATHPFLTLLARNVPPLRERVSLLYNLSEATAQSWLERRREHFSAQTVVTYVLRCQTCTA

AVLSRLQLALCGYSTTFSLWASLLCLAYLLLYLVYVSVVGQLWRSSASAYSGAASQFTSS

ALNSSVEVMASLLRHYWVTISASASPAGGGSWSSGSGKPSPLDLLRDLSASVWKGVVEPV

RFLQQQQRAQAAATVPAEADAEVDSASRSEAADAGRGRSPYFYQNGRYYDYDATPTPAWA

TPQRTLLESAPTQPTASAGSTTTASFSDNYSSMPLHNTRQGEPIHLRENAEVNLLWFFVF

AYFATFCLPRSPFRWLWRRVWSVLTHDDALARRPLLTV*

>Lp_000041200.1 hypothetical protein, conserved

MASLALLVAVMLMTGTAATATDGVSAVGKNNRQAGYSSTGHLGRTRQQFPLAGFVDASLS

GSEWATSPLSSSASASSAASANTNRGAFCGSWPRDQCALLYASNVAALLVLVSAVGAWWL

LRWWCCGTEGLHGWRRVDSAGADNDSDARVAGGVAEHERLLRAPTQGPDDMAHVVEEPEE

GGSAPAGEGNYQVNGRMARCVHRLNLHGTRLWTVLRMTPVGLFYGLSQPSSPTQPPSAAA

TRRPSSAPPGDATAWASDDDDDWPVRRDATPVSAADRRPFLRGVVSVVTATADSSESSND

SVRHSEDGGAAHEADHFSCERERLNVETVERECAVGRSTAAIRPPNSLVTQHILPQFIDY

NAASITRYDGEEGGFRDSTDSDSQQMNRRGIKTARTSTSSLTASANSSAHTPLFPAREAL

VPRIDSADSISRYNADERDASEHNSSAARVGFPHVETAIFDATANACVMTRPHLLQVDLN

SLNDIFEPDHFPPPPATLSTSEAPASSIASQLSALDCYIPPPANSGTATPASMRGSGACL

PLPVATVAPSCFAALPVSSSLNSDLDMAGEEEVVVFDTTQRSSSYVPSTQPQPQQTHSPL

TPPPPPPPAAAEKSVKARRKRSVAYYSEPWQPILVPQSTPQLVAAAAATALPTTAAAAVR

SRDSSLPPRSPMPQLSGALEDELDDLMIGDDESAQSNQTGESGHIFSGSSTSTSRTVTPA

AENEVESAVGTSTMFCAAAMACGGGGCGSGSHRGTVGFVTVTPPASASILLRPEAPPVAL

FARTAGAGGALLARCSTDTTGGSSTWPSCSVGRRPLHLSLEERTLLLKSRKWLFPLIDGA

DEIESCEVGEGKESHESDEVNAKPVDA*

>Lp_000041300.1 Fumarylacetoacetate (FAA) hydrolase family, putative

MAALAFPLRQTVLPIVGKGAKKLNFPVRRIYCVGQNYDSHAREMGGSTCRHNPFFFCHPT

DSLVTDWDNHSSADATVSIHYPPLTESYHHEVELVAAIGLPGSGVGGDAANWVDAKDADG

PRYANVAVEQAHEIICGYAVGLDMTRRDLQSQAKVNGKPWDLAKGADEGVVVSPLMPASD

LKAAHPELFLDFEKNPRKTVLCGEIFLNVNKNERQKGNLSCMVSPVTELIAHLSKAVALR

PGDLIFTGTPSGVGAVRPGDVMQAGICGIGTLLVTVL*

>Lp_000041400.1 ubiquitin-protein ligase, putative

MEVGIYEPKTQIKEVAAELKAMNAERLYDAVDAMSTSLVMGFERYLRKFDSETAIPLLVD

VCETFIREDAQADILVISLRALSLMMEHVPSSYEAAQPFHQPLMQLSSKVIHKALCTEWH

YKHSNNASMIEEGLRVMRFISKDDKTGGLIQYALVGDLLTLCTDPQPLVARQALETLFMM

ASKVVMPSELEKPTRSVTSGFLSFFKSKKKSSSTRSSDNGKGGRGASGTGGSSSPPGTVA

APEDIVPYADHPTVVQVENVIAPLLVSLVEQYAKSLASIPEHWGLLELALESLGTLIERA

LICHRPHTARALASPLLPKILFQLIVTTESNVALEPSVRVDRVLLCETTLTLLTNCHRNM

MLESLQLPVARQFFRLILEETTDEITTLLEDPFPRAIIATRSTQRRDKDHITSIAALQLF

VLACPTVLPDAFGLKPKLVLPVHQWMWEDELRHDNRLMEEQCVSLETSWARLDHKSRITV

HLKQLDADLRTMTMSKGAHGGHRNISRHYVPFVYHFADEMALRPVVAVKEAGPCSVDGTT

LVWKSEGSATKNSRAAAAATATATTVSLTERQDSTNTTRSSSSNRRGNTKHARSNSPLST

SPPTTATSLPTPVVLNTQQEVPAKHIAIAEEYFSALCLFAKNTSGQMAKQVATCACASML

QLMFLSGASQRFEQLMEKAVLPMCEMFREALISADKATKAVVLTMVGWMLYHPMADPCEF

TEGALRCGLLDQLDILSKTTLLVKDKPTRTATLAEKAGTLAGTIVEKIKERRAAQAGGAG

TNANVKADTPLGGVFLSGNMLAVQKVLEELRSCRCAKDKGTSSPVGSPKFADKTHESAIK

ELLHIFNEATDITAYEVFNVGVASALLYYLLGDMTLEELVGENVVYAAGASASTFTRETV

SQPELHTGPIDSNRNASSWATMMASGMHAADILAMFVDEKRMRCLIQWAVKYPAGMRRLV

TALVANIPLQSHLPLVESVVTFDKVVCKTPLQAHDALCRIAPLIMLCGDAGRRIAAAIKT

GNASEAITPTSATTNVPTPSNAAAGRAASGRGDDNTVTPLSSGFAPSNGGADGKQPRSNS

LLTLNRVGAANVLREAPNASNIARIAKECCMEGHRLRFIDVYLTTHRCDRCGRNIPGGYN

CRICNYDICDDCYTRYTGNVEMIKSIASMERNRVHLCASIGDLERWFRTGSTSSKGTQLA

PTSQSSLHHLERFTIRVQSYLSRYTAVGNNNNSSSASNAILPASLATAAATLPLPASGEK

GSTAAAAHNGNSGKFGASFLSFAEPFNTAAGGTTAEVAEKLLGNPAVVRRLTYRYLSRFA

TRGVLNDEGTAITPAQAEAMDAEVAQVLATEAELHVAEELVQEGVEERYLLYRTSCGLLP

LQETVLSTLYRRAFALGDHDVLLQLENCLMRLDSVGEEPLMLQRNGEVTMSNPAASSRKR

NPNVVVTKKSKAGSVSSVAAERPGLTVMTSSEKSTEAREFERHRVAMDQTYLFHHFENDA

ASFCACGAHSRAEFHTEEPPALAFTAPDLASRTDMLLLILLYRVLFEEARGPAKYATETR

DIFGEDAASIFENNTITTAVVRSLEASALRVSMLPPQYALPRWVNFLLREGRFLIPLPVR

ARVARFLAYGARRSFTRHMRVFRNQRNDCAVAILPGEWARMSNHKYTIDRSAFVRDAYVM

LRKCADARFPISFEFKGDVGVGQGPTAQFYTLLAAETSKAHAELWRVNGDKQTTSSSGGG

PTTPAHTSTGTHIPVPLARSGSNSGGSAHVGSTVTQGLPPASPPTRRSSVTPTSSPPRSN

FSRPASITSRSIVNSNSSAVVGPPSSEVTPRPTTTQAVHHVTPTVRASTSGSFASVSAQS

AANDIYLVPPSEGFYPRPLDGVTRYADDMDSVPCTPLTRRSHPAHEELSMSTLASQSSGH

ISLTLSRAADGSFNAAANNNNSNNNNGRSSNAVATAAHYDVAKAALLVDSNVGLMRDFVV

EQRERVRAYYVVGAALGRAFLDEQIFPLPLSPALAYFLQQRFPAYQFVLSDATRIDPAPE

FPIDVFELPMSAVSWVDQSVAQSLASLSKLDSATLAALELPFTLPGDDSFELLPGGAKMT

VSKKNLRQYQRRVVGALLYESVAVPLYFLTMGCRDVVPYEALQVMDIEELIGMLCGEDKN

PREPLWTTEEIRAVLVGDHGYQNDSPQLESLAKVLGARLTPSEQRDFLLFCTGCPRLPVG

GIRALGAITVVKRSNAFAEARNAAVRAGASTVAEATNEHGGQPELDVEDDEERRPLPGMY

ERTALSDQEDSSTAVQESPRPDLVSTGPALSEEASGATGVVFPELPDSEWALPSVNTCFR

YLKLPPYPTEDLLYKKLLQSITQTGGTFELS*

>Lp_000041500.1 hypothetical protein, conserved

MAGEEELRELLPKLRFNREFKKRQAYRYKKRVEAREKEMELISGKKRPASSKGDQVGKRS

TEKPVAAKKKGAAEPKKGKTAKKARTPKLKVANAETGKETLKKKKARELVATIEKKKKST

AASKVSKKQPQQELKTTEKKMKATRSGAAVSSAAAATPSPPLPLTPTERDRALLARAIRG

DDLAGGEGASADAGDGAAFIRQDPSLYPDLEFLDEYAEKKHAEVVSADQLLENAHHFFKD

LGKREQALNRVAKEHINHLRAVNRRGGDKDGFRYVVPKNVKSVVRELLAAQKARDGVEDA

EALIDPQSLVSTFGGDGVAETSDKPVRRSRRKRNRTYSDFYQFQVSKKWTRNAENFLRRS

RPNKMLFEAKKHQRSIKNF*

>Lp_000041600.1 Nop53 (60S ribosomal biogenesis), putative

MGKNSRSRNLWSDERGTKAINRPQPVAVVKGWKVAPDILPAKPIAPRRRKYAPVRPNNRD

AVATPLPGQSYNPVDDDHQQALRKAVRQLDRKKKKDEKFVAMMMHGRDRKYEGNFSADKT

WEEEVKEATPKPAKAADAKSSNKKKNKKKATKTETAQEARQAMRHRRHPLREVVAKETEH

LDELLASHAKKQEKREAARLKRRAAKKANLNVKHYGRHYHTPLVVDVAPTDKLVGSLRHL

SGGYVHPALDRMKSLEERNLVPARMRHTYNKRKVLKPKGEVRLKREEFGIMPETSF*

>Lp_000041700.1 hypothetical protein, conserved

MSALFKLVLGQKVVHPNGAKPKAEPTSHANGDAATADYADDASSHAVAYEAKSATGAPHN

GQRIASPAARVAGPSLTDTIGRPAPTAAPSTNRSASSSPKAAPAASPSPAQKMPSSLPNS

LRPSAPTRTLLLRIEGCTVAQDIRDALRDLLRCPDLPYALDDTSLKSLTALLTSYADDES

ITEPTLHLLAIATDLEAYPSKLESNRRTIEVTTADKRHTCEGLLRVLVEEVPLLLESVNA

SAPFWSRLHAVVLLQRLEEYEPSKVNHALLAARGVGALLDALNETEHDNALRNQALLLLT

SLTLADRELQTLLAFDNAFESLFDLIQREGGVLAGGTVVRDSLTVVHNMLRSNKATQKFF

REMGCAARLAALFDAIPAELAACTTADQRRGNVSASLSNWPQPAVLSDKLERLLDRVSNP

DVLLNVLMSVSVLACVLRGHEESEEEFHSTQDALLRCGLLHPLTRLAFCGLAIDDATRIE

AVRVLALLLDQSKRAMEEWLSAPAMVTLARSTLPYSVRVWPAQRALLSYVCETTDTTLVN

AGVQLFLSTLSVPACQERVVGVFMGGLIGNTVSGATAVGSSSAASTLGAAAKATSVGNNG

ANGALNNGSSSSSGGSSLSDPQCGSALARLLLSPATSAVEKYYTAQVFRTLIGLPAAAKL

SESLVRSAVPTELQQGKLDILIKAQPGWTLSSRLTPSFFNYYVSYLLFAISGGAASQQMN

TSALGAYVAVLLAWTGACPWAAAALVQEVSWCDTLLRQARRDGAAHLRLWSAMLLARGCV

VVRAAAAAASGKSTPHLGESTTVAAGTALVQRFMEVVGGGAALDTILFDAQASTPAWQHP

VPSGLRAQTPTPYDEPFVAQAEQLVQEFKQLLSTAAGTSISSASLPLLPHPAQPASGTAA

LSSSSSPPPPYAPVTVTPETAPEYTSLPPQGQPQPSQQQSVLVSAPPSFASSADYQVPSS

STLSHTTLPHAMTGDAPAGHAEPNAEALALIQALQAQLHAAEAERARLMSTVDEWRGRAE

LSEAQCIALRKEQAERSAALATAQAELARQRDVAAAAQASASSADVVEGLRENIRLLEEA

LNSKDEEQQQLVESLNMMEEQLRHATDAAAAANEQRATALATLAQQQQLQQQQQQQQQQQ

QQQQGPPPAWIAEVEDERGTLHRRLEEAQQNVGRLSHALQELTKDYRDLLLMVAELNEEC

VSVKALSSMAPTPARPLPEWPSTNPSANWDYDHGVPAPSAVTGADADQDVQALVNSLPVA

AEGMWSATAESPSTQLAELARAASSAAYAAATDVLIDSSPSQPSPPLLVGPPPHHDEHHR

HFHEQTSISPPTHQCSFEHHGTLHPVTAALGPQQRQQEAVPAENDKSHNNGTAGTSPLPS

ADEFFGVDGGNEDESNGNDVFGHAVSTAVTAAHSGHSDLPAGAHDRSANPLPSPLASQPQ

AKPTSASASMRNEHIASDAMPLTPPPPPPMAPSSYKRPVSALAGNSRHLDYKAKAGPNEQ

RPNPHATAANVNERNGTVTSSARAPPPPPAAPAAALPHDESSESAPHAVTAYNPFADIQS

GDNDNDGLAELR*

>Lp_000041800.1 hypothetical protein, conserved

MEDRANASITAGAEGVAAPFAPILGECVLKLTHSTTYEPPFYLFPELLPQVPQGTPPLPV

WDAVADAVDISASSWRMADASQLLAASTVATSNRARGGTSCSSPRPPQAMVTLTSMIAEN

RLLAAGLDAHALQQGSFSSASIIQQLATLQARMALTDEEGAAIMSNHFINLLAWQKLREI

NGDERNADLAHNSAPSSSSSTTSAPKSSAASALSRKRAHAAEGNDDSSAWLPSPDPWASE

AELACGGSNGERFVAAGEENVEDRVDLLRTLAELYQRPWRTMVSQGQQTDCVVPPGTVNV

TSPFANTTKLPGAMLDPAAELGWGVSTTAEEHNPLFFGSVRLSHNACFYSVLISARSSQV

GAAPQRVPLRQLVTYIGREAGAQRAAAVAAAEWVPIHAGLKAFTSQPEVLSRLHFALVLR

PVYNHKTAMLASGGVAGSDAEAPPSSSDGEEDEDGTTAPGAFAVVHTKTPDHYTLWLVNY

GRNGVRVAGRGWVLGEPRQLRAGDTLVLGGDVELRVEQHSTAAAACATAAVDRADSSIPS

QVKQERAEDE*

>Lp_000041900.1 Yos1-like, putative

MGVSIYSLVDAIVLCLNALAILSESRFLSKFGLATPNAQQGFMPRQDSVFSDCSPAGFDP

DTGAGSNNGVVRHSSIKFQIASLLHSVRLLLRWPLIFANIILVVLALIFG*

>Lp_000042000.1 Ring finger domain/IQ calmodulin-binding motif containing protein, putative

MRDKRQQKLGLWSAVALQQHLASTGGQRGRPLPSVTSCSTEAQEAQLTGAARGSFAAVGA

TADGAVETMGATTEDLRTFLGPAGSLPGHPHKPTLAQRMGLVPAPPPEPSAEDWKAVVQR

AMLREQQSHYGDSSTHVASVCSICQMSFLSTSDEGQVILSCSHVFHAQCFHAFERCVRAQ

QRADGAGLAEVTAQLACPVCRTQHYYKRVFYEGKAMAQRAAIVKVQAAIRGFLARRRYVQ

RRLRSNADFRTRYVQERLSRLSAAWSAFCAQQERQRETTLVALAVQHQAATAAYLTVEEW

EAIWQAALKREAESSSSYGGGGGRLGVLGVLHCPICLEDIHETFFTQRRRETEASGTQAS

GEEFLTALRAAYEKRTRAAAGVKTGVSRSRNAEVALTQGGKRAHLADNIKGPNACKAAIK

SGVATKPKTHSQARFPSSQPIRGLSDCLPTAATTTLPPLTPASALQKNTLASGPRSGVLL

SCGHCFHTACISCYERYNEQRITEMAGSSQTATCIVVADRCPICRAGYAKHAL*

>Lp_000042100.1 hypothetical protein, conserved

MSGIDYSKWDRLECCSSSSSSSGGSDEEPRPNDEHTSSGDRGAPKVTRLEYPTHITLGPS

GVQLGKAPPKASATPFHPSVSQRQVPQLETSVQNAELTTGFTSSSTSPRDATQQLHSNEE

GADAADEDEDLLYESLARNGGREGTEHWWSQTEDSATVSFLVPWETTAKMVTRFNLHEVR

DDTTGQHHAQLDVLIHTPAHYSTAVPLNVENADATFSPSSGGVVHLCKRFRYPIKLSEEL

VEGCWQLHRMPHRHVRLLVVEVFKEAIGQGMTLWWDRCFVSDSVAVIDTQKIPDRVQTAA

AGGTAAQTKAEQFRQVWDDAHEEFRRRMRERKAKQL*

>Lp_000042200.1 hypothetical protein

MRCCTSKGRWKRSREAAEVQAKQAEVLFSDYRDALGKVKDDLHFSHRPFLLTTAAAAVSS

SPSSSLEREQPARQPQKTALHRQTSVDTLADAVRLTYTQIQQLRRIVLRNSADSLCDKVK

QKQRRAFFSWWARRGARRVRATALELSLQRSLLATFYAQWKRRTDSDGRQQLLRRERHLR

AVIGRSQKAVLSVYLRKWRLFVTTSSTQAAQLRLYQLRIARLWSTSGPSVTACKQRFFAV

WRAWLTIRKAERHHAAASALLETQHADEHQLHLLRSSFTNWHEKTQQRRLARLYHRLSRV

MAMQTVQSVLRRFYRSWAAKAERARRLHRLEVLVADQHKRRVTALARRYFNELRYFRREQ

QFQRMMRGVEASLLTLADRVGGIEDVVLAQNKPPIAVATAPPRQLFLSSPHTQVAQYPQQ

RMVMQEDKTAPQEPWIPATDDDDLLARVEELLSRPRSSTSY*

>Lp_000042500.1 hypothetical protein, conserved

MSSDDIDALRSELLRQKIIFEAELRRQREAFEERLSSIAQELADREADCRNLQSVITILG

RKVDAIAEQVSGSSTAAVARPPTPTRSVSVHQSPFRRNLSSIPGAAHTSQERVRTNSLLS

VNSRRASPLVPTSSVRRTNSALARLPSATALSRTESPHVIGGHPARPLLRTNTNASAASD

TGSNGDSRPRSRKLTRRRTNSSHRLTGIKATGIW*

>Lp_000042600.1 hypothetical protein, conserved

MQRFASALSARSFALGPSQRCLRGPVVMFMPRRGSSSAVPPSTPARPPTTAGTAHADASA

HEAQAMAPELDAKAAEQVRLAQDIGEQAFGENTEILKQIGYRGLRAFVVCVVGLSAFMWA

MKRKKAELAAQEAAKADDDDPTQRYLEEMRGLGFDVDTLEEELEQERVAKLAAKKAVKSV

V*

>Lp_000042700.1 DHHC palmitoyltransferase, putative

MQKPASFLPERGAHAHARARVTAANSNLWQRREEGSEQDEEGSIVVHVGTSSAAARSIAI

PPPPGAAAITATGPSSSSPLPDGGDVVDSDGESAVAPATQRLTAEGPHAVSTAASVRLAS

SPVNVPPGAVPASRYQNMYDGSLYLPPGTTKRALGRLRLVWCCVLTQESWHACFVAGPIL

FFTTLFVALVVPPGEWFSYVFTAVFTAGSLTCLTLSVTLDPGVIPPAPLSEQPSGPASVL

VAGKMVECKVCKTCHILRPPRSSHCKFCDVCVEEYDHHCGVLGSCVAKRTFRFFGGFFII

TTFLALYVGIRSFAVVVSTDFRKGNEDLHLLGIAAGSVVCILAAILGAAMVVPCAVRYLM

LSAMNETTKESMRREQEQEQEQPDQLPGCVPVEEMRDDDYCKNVLRRLFSPLGKSRVPFN

YYV*

>Lp_000042800.1 hypothetical protein, conserved

MKLAPRERRQRSDYLVDNAAALQIGHLFLNWGKKRSKRKGKRGACGAEGEKKKPAPAKKA

AAKERQSPPKKKPPAKKAAPPAKKAAPPAPTKASESPHAAPKPAGRPRKYTAPLPRQRAA

QPQPSKAVAEGRVVQQRGRRPTRPPVIPKPVPTPAMPECLPSDSAGSVDTSPMVAVVVRT

MNGGSFRLVLTADAILYELKRQILCVIAPATAAQNALQRLPMLVMNGRALIPDNATLRSL

GFTATSTVYCFPEPS*

>Lp_000042900.1 TFIIF-stimulated CTD phosphatase, putative

MDEPVASKLTLPPKGSELLRENLFEENADEDEGELLKRREPTMLRKGRKLVAPSNDGAKK

YTVVFDLDETVVYARNGPLYARAYLKDLFRSIKDDFEVIVWTAGERDYAKCILEEINEDH

IIQHLVYRHKKWFNEEDYTKDLRQLGRDLNYTIMIENTPDCVRANPQNSIIVEDFEVLPE

TTDEESSTSPTPVPPAPASASSGAFDQTNSSSSEQRRSESAEDASDGCAPAHAAATADAT

SGKEDGAPTSQPKRRRTTDRTLFLLREVLSTLVKSGETVPAFLASCGLLSQQTVIGSDGS

TIPIYHLGTRRRRKDAGAPRKVVKVNRDKAARAPTEDAPLAVDAGNVSPKPRDKPQHRDD

DNDDGTPSHAPSKKRKLKSETSLDEVPLLSSDSTDSVQGARGANDEDANRST*

>Lp_000043000.1 PHD-like zinc-binding domain containing protein, putative

MPPRVRVCLRLKFVKLPNIDSERVFRDSLAFSDPDDRSYDDLRAIVEAKITFMELPLSEF

TVFYTEEATQDRLVVDNALFSSLLRQWSRKAAKQRRNSPCAAVAPALLGDSASQCDDNVD

KQLRASIGDDNDDDDDDDTHPPDVVASFEVELTRLSGGGGINARAPAAMTTVFPAVPNLV

RSSAFPVASATPPPPTASPPSTQLAALTRENLSRLCEAKTNSHANSSAADPSVNVPASPL

DPNADVHVRVHDLTCPPTHVKFFLRCIILAIRFKTPKPGMTVAELDLGDADDVTQAITAV

TFDEVVHKAIRDHLRGDRRQILELRQVYVRRKNDVDMRYQTNPHPLLLRLDRSSKLEVVR

ILATPAPQPGSAGVVTVREQLGSAGVVDVTQLMGLQQRRHGSRVGGVLVSESGASAGPLH

GGAYPAATSSSSSSSGPRRTTFTMSGTSGNVGASVALVSGVVPSAFGQELRTRQEQLIRQ

TPPVATAPLEPGVPIVTARDVRQREPLVEQHANREEQRKQRIRRKTEVSTRCLLCGLDCN

DEQACMAIVRQLLSKSLRNRGTHIPTFGELKRDLCDRRRVPPGKEPTRLLCAHTFVNGST

RTVHVVHPRCAHLCTAYQTGSELEDIAESELEMNVCALCGMPGACVACYHPQCTEVYHVV

CALYSSGYVNFGQRDPFLPCPACPRHTQVPLAKKKREDGSNVLHVDHSCWEDGIAFDSRV

VESTDLRDPDENDGQ*

>Lp_000043100.1 hypothetical protein, conserved

MTKDRIHYRQSRRNAHGAEEVERGIGITCTRDARDGGPQRPVALMKWAFDDYIVEEELPE

ELFAPNPSADTSSSTDTAQAASTSFVVQLLSEGVPDTVVEKLLRERLPRSVSPHFLTPID

FVCCAARYVWLRCREASAPTLPPAFWHDQHWHTGVGDVYVSPVRRIHVSATAASFPARFL

PRTRYTVTLRNIQSSLGDVLPQLRRVAQDGFLNYSHVARHGVGLSRIYDDAALLLRRDYP

AFLRHHVQRLTEGTTHVQREMPLLLETLASPRSSARDWRAIVDGLEFAVKSDAQVYARTR

QSGLYPPHHDLLLDFVRRAADVLPRHHDSAQLIREAVKPLVLQETLRAITDVHFNAFASL

RWRQSGESVVVGDLVRVQGAAASGADATGAQDCFVGVADHVNGCMTSSAHVEWSSADAEG

EAAVAATCAVKRVASKAEAANYSALDVVLPCFGKGVAELVPSSYAESAQATLFANLAREL

RVEGLAELNLAPCAAFRPLLQRPHNMSFYLVDEKRGWDWEENAGSTLLKQSLYHDQDGLL

RCRSPFARRTGKNMVARMGIRDDVARHFFLKPARKPGMTCVMQLTLPAGSHATSVIREAF

TVATLSPSAIFRLLAK*

>Lp_000043200.1 hypothetical protein, conserved

MSLDKSRISIVSSTKVDGNKLSSVGDTADSLVPASHRQQTKEEKCCPVCCVLYGSIISTF

AVFCIAASGTAVSIFAESRVNYTVNGVMRQIGWIGFPGMLVGASLHYFLAEAMWSGKHNS

WGQAWTKAIIVNAGLWSAAIGLGTLGWRKGLPQTAAGRRLYHRYPIPAEPLESRLLRSSR

EFFSGMGATYWLSGVVSGHLGFVTCVTFCVSTDRPYLMMAPHGGYARRCMPPWRRQQLEK

MALGADASAAEEAAALPPPRQKQAIK*

>Lp_000043300.1 hypothetical protein, conserved

MDSSTDSFTAERRQEDAASLHVSSPPATDDDTQRPLLFTAHETEVSFLPASSPAILVEET

ETFSRVANPTAEDASESPEHETAHLLSKETLRTVGAGREPALLGAEASQDLLEVRPAAVG

TGDSKEEEEDKKATAMVMTDTDDDEADLARGTAIQDKYGATSAWVESLPLAATATAQTRC

GKRAREKRESQSSSEAHGGGTLRQRRAREKAAEAELQNAFKHSQRALRKQAKSVNMRDVF

ARSITLPSPPPPADANASTSLTFPPSPLVAEDSSSGNGVRTAGGNVFSPSSPSFRLGTPL

FSPVGALSPTTPLGNGGAWSPCVVGELTNQFLANVKHQRRQKCELFVSRELTQSQTMSQA

RSLHTHPYGEDGTEETGHPSLQQQQHLPSSMTDGGTANGRGFPAPVDELVIGDEDNEAAN

WMPAEASSPASLHGRRMVMRDTNANDDIVLLGGSSAGEEGCYTTSPSTTASESRKAEDLA

SQQHQQQVVQSLSRKHEIWKLKQRQLKAQQQVDLDERAKAKQQQQLSVASSTAASATFAS

ATVTTSATTWQGFIRTSQISSPAGTAFRTLHKANLSPDAISMIRRLNSFDSTSAQRVVVF

GTAASTKQSKDESTQ*

>Lp_000043400.1 ankyrin repeat protein, putative

MTSITPLLPTRRESPKETIFDACRRGNVQRFTEYVEKGGCLTEYDDQKLTLLHHAAFAGN

ESFVKLLLAAPATQQVSIDALDAEGWTPLHYAADRGHAGVVRLLLEEGANVNARDTSKRT

PLHLAALSGRTDAVAALLREGASKTAKNAVGMTPLECARAADQADIVALLQ*

>Lp_000043500.1 Heat shock factor binding protein 1, putative

MPPKTAASRAKGADQKSASPVSAAPAAMAGNAMPSAVPQGGTQELTSYVQGLLQQMQSRF

EEMSNNIISRIDEMGARIDDLERSIDELMQQSGVEDGDTSAAKPHK*

>Lp_000043600.1 BTB/POZ domain containing protein, putative

MPVSKSARCDEQGGSARGGNATRQPKRGGGRKRARDTATTTASVSADNTEPLVSPASNGT

GLREGGEATEHAPLISSPSATPAGRSSVREPPSSTPSSIGVADEEEEENGLVRSAMASTA

FSNDEAGSVNESASVARLRSEVCSAAENIRRNMETSISRFTLADAELERLASARLTVQPK

VDEVVQSRHPELYAEVVTLNVGGEIFTVPLATLLGKDSPNYFHVLLGSDEAGGVTPVFKN

EDKIIFIDRDPTCFKHILGYFRGYQYFNLLKEDTVRRLKVDAAYFQLPGLLAMLGELERE

AELQFNTGPGVSLERNRLRVVYGVAVVGDVFLVTGRHIITYEVKIADYIGFGLVSEACVN

TDQEFHKIPDCCVYYMSGVFYTNYPYHRKEENLERIENGDFVTMKIDLDKGYVEYILKNS

RKMVSIGRARRLRFATTMKLASRVRIVPPEEVRRRLPPVHYTFDE*

>Lp_000043700.1 hypothetical protein, conserved

MSVRPNASLLSEYYPTSSSSLQQHTYLRVLWLPLSMCFLWSGETLTPKNTSAPLAVTSPA

SGGGSFSNLNHSGCSTANASNVRGSFCDRHAKAFVDKDETVGGYEVALRRCLTVHPPVPA

AQLSWDAFSPEVLKSIAALSKLASTPVELPFFLTKDLGTSNWENMDGHRATVAVIVVIDA

AVLSVTADHAVDLVATIFTSQFKDKVCRCVVVDPPARLADKLAGSSLFRCVSAAVAVEQT

AEALLLDVAQGVVSAHAAALRLYLQPNKADSTLVRTPVDKPSTFAPSRTAAAMTASRFKK

KCADILLQCGAVTCAVAAYGETQFGSNADCLWCSATVESIAAARYHYLRTTLLRHRATLD

ATVVQLQSDDPPWGPGLTAAVEELGTAVVQYGDSLKLTLSSLKNGSVPHSMQNRLTRDID

DNVSAQITLLKGLLVRVRVCLEAKTWEVPSDPQSYRLGSDVRRCVENVFRLAYAEIDVYL

RESLRQLSKAAPSGGLWPSSSPLQLGSASSSGGPQSATITMLRKRELETRFKRLELLAAR

EARQPFLEELSGLRSSFAGGYGAEWTERSLLFFAYLCMVNGAERRARALLVEMASVRTRL

QLVEDAVEMLLRVCALTGLELPLAGLEAAPAADTATVRGLDPLYRLSRLPAEREAAAAAA

AAGGGGNGSAANVSLTAAPFNMFSAVEEDSPDRGVARLNQSISNAALLNVPLLRELMELF

GRLGPPTATAALRCQLATLLLFQHPHLLDKPTQRALKAVMEETSGLLEPQMPATTVPSPF

FLAWEALPLPPHLAPKTVPVGGALFTFIDTQRLKLTILCLNGKVLGSRTVWTVGDVASVL

VTLYNPLEEPLVFGALALRCRGTRTGEKEAEGQQTGSSMSGTAAVGAQARLPQSRKTTED

VTLSEPICYVLSQVEVQPLSKRKVLLHVQPTQEGTLVIDGVALRLSKMRSSQPIVGQLPA

PMHIPVLQRLPQVSCTLNTSELEVFGSQRIDFTVRVVNCGRVPISCISLTAHSEQCQLEG

CEGCKERRNDTDTSVTLNKRALDAASRVPLEPGDVVMIPGVLEAPPTIAKFGAHYVLFRT

DLSLPHPEPEKPPNVPGAVPIYAVIPRRVAETRMRLFHSPGLVVTSVALTKDRRAVEVRV

ANRSRLYSMELQLSALTFAELPDAFIVAGAEYVVPPIKLTRILPAKEQNGLRFSVPWVVR

ELPHCSGTLDLDLSVIGAEEVSAEPLDECVLTLDVELPRLPFFRSPYRPSASSIGSLDEM

NDAAAQQALGTASPPADGPVVAYRWATGGDIGYGGENSPLVVSCSRAPPPVVTSALHSPT

SVSALPLDGRAVAPVTTVPDFAGATRPNSAFASRRTTSFEFTAQPSATNTPLLQRAKTTV

PVLSDATANHNPPASLNAPPAKVDIVIPAVTPIHLCLRVAAPRWRRAIPLRVRVSIDAHF

DVAVLSGAVEAEAMVGNEGKAVYVREFELFAFKTGKHLLHVTITDGAGRELTNAVQLIVE

HSRAA*

>Lp_000043800.1 hypothetical protein, conserved

MRVAPVYHTVLKQLSQACKTRPHFVEDVRFILSLPLSAHHDKHTVVHGTTSAAGTTLTST

AAPLEIPGIGNACEHTTVENYLLRPPSMCYPIDGEALFALCYADLQGALRQPPRADANVN

PVTQQILDYRNLLWLRDRLTRVLSDKTTMQLSLSLSIGDGPDADDFIPDEFFAPQSGSAT

ERVVTGLHGGFIDVASGTLVDDAGADKPTQQSTSASASPPSPSSSQKEVPARPAVLASGA

VAEDDPEVVYSAGAVGLQKEMFVLRHREPFPMAAHFLAGFPTIPATELAQCAHERSALMQ

LTKKIPTTVTCEDGHIRLSVVVEPFDPRYRTREHAGFGYFAKTHRNFSLQFSMQLLAPDA

EGVAERTELLVVNSYFLRLDMELLQVTEEVGYLDASDVFRMLRERDYGDCDSDDGGEEEH

SKHNGNTNSSRRSNSASDDTTKEKHVSAEAKDSAATSSSPSDTARVFSLFFATPTDAPMV

LKGLLYYKTGKRSELAKAPIRCIPFGFVRLEP*

>Lp_000043900.1 QA-SNARE protein putative

MATRDRTSEFLQYRAIRPRRPETEQLLAEEDNMNRVYVTPLWVEKMDDVRRVEEQIKEQM

TALEKLRKDHLKVEFSSTRDESREEAAIEEAQDTIDRLFKQSEKGVKDLDIAYTRDLPDG

GTDAELSILRNVKMCLVNEINTISKQYRESQRRYMMDVKKQQLVSQRWAGGDRQKAVEQQ

LENDALMDQYLQKGMTQEQVETIMLNQQMADERVKEFERIYSSIKSLHEMFKDMNTLVIE

QGAVLDRIDYNMTITHTRVQKARQELQKAAEYQSAGTFKLCVLFMVVLIIGLMIALFFKA

VL*

>Lp_000044000.1 hypothetical protein

MIIVFHISDSQGLQHRCSAICGPSTTAVGAINQLRSKLCIPTEVEVEVHLANDVPIRHLT

APLTSLRKQRGGSAQVLRMQLWASLSGPEAAVREINYSTAEAVDPADAAAEDEDDNVAPP

TLSQSERRSSDCTAHVSSVPTTRIMFAGPPSRANVKPVQAKMPSSISTPVHARPAPHVRC

ATAAAGSSANPPSTSEGVAVAATPVKNAGSRKPSPVTTVPDSAEAASTPSITARPPESTA

TSTAASASTLVIKRLDSRPPRTPRMEMPLSARGPVRRPPMDVQWAEKWVPQSARGVAHAL

APPVARAGTSGSNGAGAGATSNGRHAEAAGEGGATLHRTGCARRDAAIAHLIMREYPKIV

RREPKAIVAAVKDNPHLASLSFSAEFTAASDLDGVDDATYARCLSAGLTHGLELRSGGAA

ASGSGSDGARLALYGELSSLRHSCCPNAAVQYDLFAAPYAGNCRCAVLSGIPQGQEITYL

YKHADSLAFLLLSRDRRRNILQRKYFMVCKCPRCTEVVEDTEDIPAPIVKKKGSKTGTAS

AKPQKIFTRTKAQREAEETLTGAFFTDSTVDRDAKKQRALMEEMRKDFGALQIIDDTGVD

ITLSLVGNVPPIQRTKQCNRLLSFLRKYGTPESVLRLHEHHWRMNLARAAYVQETVRLCA

VKGATPEARLRDPHSETLFTPTKTVYDVCLKQLAVEALFIPAGHPHSLTTYESFLYLVAI

LPPSLAQTVTRAAHNTVSIKWKQLEETKEAWSVLKRTALPPQVRQLLQHQESNHSNNGGT

TASAELPSQPVTARGPGSHDLLAAPTVKLPPKKATAKQKS*

>Lp_000044100.1 acyl-CoA dehydrogenase, putative

MFRFTCKPLMRRTAVALSAAATGVKLPNPVTYLTDDEKMLVEAVRAFSLQHVVPKSLKMD

QEGKMDQEIIKEAFSAGLMGIETPAELDGGGMTFFSSILAIEELARHDPSVSVTVDVQNT

LVNNIFFSFANDAQRKKYLPKLATNTVGCFCLTESGSGSDAFALKTKAEKRGSKYVINGS

KLYITNGGWAGIYLVMATVDSSKGYKGITCFVVDSSETPGVSVVRTEDKLGIRASSTAEL

RFENVEVPEENVIGEVGKGYKIAINILNEGRIGIGAQMLGIAQGSLDIVMPYLFQRKQFG

RAIGDFQGMQMQYAECALELHAARLMVYNAARKKQNNEVFIQDAAMAKYFASTVAEKTAS

RAVEWAGGIGFMKDFGLERFYRDAKIGAIYEGTSIIQLQTIAKMIKAQYDKS*

>Lp_000044200.1 galactokinase-like protein

MPAESYSDDRLDSTIKELEPIFRREFQVKNDSDVEWLLFTFAPGRINLIGEHVDYMEGWV

CPAAVQEGTHILVGRVKHLETEAKPKLRFYATYNQEHFDMDSIGGGKHNKAWTTFVRGAV

TLRLRRLGADIDHPALRGVCMVVHGTLAMGAGMSASAAFGVALIHAVNSLVTKTYQHCPT

SAGRRYAVVPALPRQDLVELAKEARRIETDHCGVNVGIMDQFISALAETDKFMFLDCKKL

TFETIELTPLLGHGEYCWMLIDSMIKHDLLGGTASTYNAVRNDQENSQKKIGEHRYLGKP

FSFSLMVRNPAEYNFDGNVEKFMAEFKPLMTPGEFERGSYQLMEQLRTLEFRRINDAKEP

LSQEERVKAAGQILNAGHAGMRDLMKITTPELDYIQELINQDKDVAGGRMMGGGFGGCII

LLLRTSAQERVKAHVRKHFKARFKLENTCYMVERVGCGGFVVSLERRNKDKSKL*

>Lp_000044300.1 hypothetical protein

MLYTEDADAAVSAAGSLNTAPSPPTSSSQRHNASTSTPSFHKGGDIQYHLRAPSLSHLVF

LLGTPLLCFLNGCSYYLFWRSRYFEEASSPQVAAATTPAAAATPFFPLVVMASASSSAVA

PSAFYGVTAGFAFAFIVTQWVIQESAHVFVCAQTERARSASFRRCGVLFVVAFLPLTIAQ

MVLISQDYHLCKGAAWLLRLEPIGSVAGTSAVVRATLSSSASHRYAAVWIVSFVLTTTSM

VVILSAMWTDAAVSWLQRRVFAAYLAAQKEMGQHELS*

>Lp_000044400.1 hypothetical protein, conserved

MEVGKLVTSEACGNIFYFVVDETHPDEPQPDASRCYYVVAPAASNFSYREAYCVLCRALC

GLENRTYLHRWHALYEQIFGTTPYVHFVNAVRDSPLTQPNSFHHVPAFPGGADGLSRPTI

TLPALFLDETTARLALRKAEQEQTAESEAETQKAQERQSSHNALLQQPGGLQGCSTPLSK

PVKTRRVNAVAVEGAVGEPTKQQAAVAAAVEQNTELLLRRNTKATEGAQKQKLDTVLGAA

SLRREYTSAVAFDDNRRTPQQLRHDLRRERRQRLEAEERCYHQTRALEEAPASLQRREEQ

AVTSGTIAAAAAADIAPTEKQRTPAEPLRKEVQQLKRRVQELEAELADAKRRHMLECDSA

AEEKEALNQQLWQRSQAQGAQISGFLRENADAVKRVEEALEKREKELCAVYTRALEDRDV

RIAQLSQEVLQHLHSSRQLATSRGDQEKMQAERVAHLEDMTSHLKEWNVVLRRQVSESAE

EIRELRQRLQDSQSQRQQTLIVEREEQQQPSSFPASLSLRCSVCGGLPHAAAVQLATHLR

DAQTQLLTARTDAERKSQELNRAAQAIEQLTRGVKKAKLDVESCFHHTVNTIDPLHSITA

AQSIV*

>Lp_000044500.1 hypothetical protein, conserved

MLLFSLRRLCSVSSLTDSVAAHSPSSRGIHKSKRAAQEQLKHRLRQRRLQRGRNIAAKEA

EVQRSMENVSSSRDILVGQLADEIFARGHRHVASATNVNFVPDGNPATPLVALAGRETCG

KTSLLRSLFRSAGEVGRSNRQLRRDAMNFFNVGDVFNVADLPGCGGTSVPWSTVLQHAVL

LRNFARCQPSLKMLYYCMDVHYKHGVYVQDIDLLRFLCREVPNFTIVITKADQINDHAAR

LVHMEDIRKELLYNDIHHPVLVTSAFHMGGVDTLRYDMVMNCLHAVPTERLTLTEARRLS

ARLFSQKELSTIRPLTIAPSLVEDERQAWLEDIQCNEQTEAVKQPTHARIAAQVSTEAAG

GLNSINPQASDGVDGDDADLQPRQPAEAEGAERGEDVATSRDAAMALPEAAKSLDAASAS

ASPIPVVFDDPVTQVAYERVVKMLRNKPLFRYVDATSPWRNPLRWPRHVVPTKHPKVNIM

RCPEAPENPYLFQPQFVAPRADLYFRRPNVGVRKSSQKGRYEADRALAFLVKSYTIPYFP

DIVDTAMQPTPWAFLGSREAYYERNGGRQLGVRLLNYAMDGALNPLSDSPAPAQPELAKE

VEKLEAKRYGAPIAMLRPPELDQDTHAPSLPHRS*

>Lp_000044600.1 hypothetical protein, conserved

MSSLVFHTEQAAAAEARVNGCGSDDTRSYLEAELSQPVRDSIRALLRHHPRDPIRFLAHS

LQEGQHSRHQKTLSTTEATTATAAVDVFPSVTAHTSSPAHSAQHPSRSTTTTPPVSLVLH

DGSTCEVRPVSADFARSDALHSSCLPAPSSFPSVLYREAASLHEVGSCLRQLRQLAPNAA

VLLFVEAPAPTSTVFFYQGAAYACSLRYPTTAIRAQGDVTDEVQAALSTYGGAVQDFEAA

LRTTAADADPAVRVELLPSVQTNYLAAFDQITRALSDASTEGSVQRGSTTPPASSSPPPS

PSLSPLPSVLLVSYRANTARHTHARLLAAYGPYYEAAQRLALQALRQDQLERKKKFTFTF

AMQYWTEVQRRREAANASTGNATAATASADLDLEVNETGSERANATRAAGECGGEKTHAD

AVPSSAAAKFLAKAKRVATRAATRAATRAAKRGLPTYDANQQAEDDVFLVVVRRLEHAAA

TLIQCVFRGHRARVRCQRERQLRQQETSSFADGYVALDEQQKKKAAQQRASSFGVDVVPP

LLQASLERLSEEGASFRHIWTDLPTSPPPDSRRCWIYRPVQRTTKASNGDGAVPGQGKDE

AKSSITRVREDWVQEELLIPPLRATEPRAHINLAQRFADYLAVAQCTVLPSTMQLFADSP

GMSVLQRRAYDSVKSLCLTLFFVAYSELRQRGCASVPATFSQYMREFHSPVLTWFNVPHG

SSLLLQSTRERSMAKEHAAERAAAEVVRLQLSTHEAFLVAQSESKASTAAPGGGESGDGG

EASEKAFTRLSSHVFLSLQPLLPASQWSRHVAELAGGFATSRGGTITAPQHSADERSGIV

HWWSGAAVPLCCVKNTPVAAVARWDAQLLDAAPAYDWFVPRVRSVAADAFTGGIADASKS

CASRGAVARCDAASSATTVIREWLRGDFAMAADEALAKYTRELLSNYSYLECNRVSRYRC

GVQRSSSAATFIDVPHDAALVKYVAVSAANEAMKPVEAPLENSTRDGEDALSTVTDSAVA

PSCPIESHDRRERVIEAAATRYNNPRQSPISASPRASVSGFGWSHSTSAQPSVSEAASVS

SKDSQEEPAVMDKSTTLRHEDSESAVQSHHTTKEEKTSWSVQTVDEVASEVATTVTAATL

RHTRRALRFVPGLEGARQLDDFVDLVVAAVATGSVGAPTCLVFALDITNEAFFALAAALV

DHRLHGSRSVHSTSASASLSAGSDDTNVESDEHLRFLGGYHAILNDTLSDTSTAGSTPEV

TPEKRPNMSVQLAVTHVSCLLNATTNTTADSAAAPSLHLLKRLKHALQRAEGAAVATSAS

SAADVQYYTTQATQLAEQYAWLVLLEWYLWSPHFPFTTASEFSPARRRSSARSAKSHGFA

EVMQTATAALQWMEEMDPWAAAGITAERCPDPFHRRYSNGLRRWDDRHYLCFGVYTDSG*

>Lp_000044700.1 methyltransferase-like protein

MPATRPELENPPEVFYNASEARKYTVSTRVRKIQRDMTLRALELLNLPKDEVTTGLNRSA

LLLDIGCGSGLSGDVLTEQGHAWMGVDISADMLRIAKEDELSFYDLGTAQQQQQRAEELT

LTSSSSNLRMDTSHVKWGLVTSEDDEDEDVEAEANDDHDDSGDTEDDEGGEEVEGPVPGP

HVVEVLRNDIGAGLPFRPGTFDGCISISVLQWLCHSTKKGEVPQRRLIALFQSLYNALRR

GAKAVFQFYPSDANQVHMVTHAAMKCGFNGGVVLDYPNSARARKYYLVLQAGQVAGGFVP

PPALTADVEDEEDSDIDEEDEEYYDSDDDDEAEHFRDPHSRKRVYVGGRDAVHRHKRARA

SPAHGQKRHRKDNRPETGSREWVLMKKEERRRRGYKTSADSKYTMRQRRPRF*

>Lp_000044800.1 hypothetical protein, conserved

MKESGSDAGSVAAPLPQEQITMLLNKQDFNDKVCQDKSNLCCVLVTSTLCRQCGIRHIPY

PKPPSRTAEDSTADGDDNGEGDGEDAAGELDEDGEEDGSPHDGNSAAAGHFYRDFETHLV

RKAATAVQRRHVRFFHVCACAEGETCVDFLIAADPAYRVLNKKPTVHEMAQLHTTAHRQL

HELLRLLEVRSTPQMRFYLAGQPLRYSMMATDMGGVTKANDVVMATGANWVKWARVLENA

VVVRNAVMHDADIAEREQARLARAEAKRLAREARRRQATENDAEEEDDE*

>Lp_000044900.1 hypothetical protein, conserved

MVRKRPATISSSSPGERGRFESRSPPSDSNSSNHIGNKNHPPKARARTASSNLALEDGLL

PLVVPVQKLFLPGDMQRQQQRRSSDQRAPFQKKWPLAVMTPAGAPRAAHRPLNNAAEHKA

AARANRRAGNANLVAKANPTAMTATPRRPSNVAVSKPSRIPDMRRPNSYDRRPAAPHSSD

RRSPDQDARTRRANTAGEVARQNPEEEEDDEELTPEEVLLLCGVQPGSAKALWFTSMHAQ

LDTRASGNASLMSEVGKPYSSPKLVQMLDSACAVSAAPPSSRTPLSTPPATCNPAASLLS

IPLQIPVELLSQDENASFSHASVSASTLPVPPPLSPPVTAPAMPPAVASAAQLPHAPSRR

PPSFSPPGEHAGRSSRTETMSEDDGEKGPAPHEASSTSSEHSDEDEGEEAEEEEETSEEK

EEEESTGTDESDEESSEESYSPSPTKFGKHTCKPASKAADLSHLRPSRRQLQEQRVRVLS

LHQQKKREEEKGYGVV*

>Lp_000045000.1 calcineurin-like phosphoesterase-like protein

MSSQEDVLYYLYDPKVVAPASNKYIRVISELLAGYYDSSYINNIDVMDILRGGALEAVEL

CRDAREILAKEPTVLDLCVGSSDEMVFVGDIHGQFNDLLHSVLSVQLCKSPPVRNTPVPS

SPAEEEASARACDEESSSGATPSTRVPRSPATSVSWHRVNSNEFTSQLDKTIRFLFLGDY

VDRGPRSVEVIVLLLALKIEYPQHVFLLRGNHEEAQTSRLYGFYSECRAKLFVVPRSDGD

LAQGGSESLIQRSPRSSSGKAGTGDGSSEDDDEASNKQGERERERSRPNNSEAGSSYSLS

YPIFRTPLNTNGAVDAWMSFNATFCWLPLAAVVRCRAGAFFCAHGGLSPTLHRISQLLRL

RRETYGSENCETTTSPSSSNTCSSRSSPEPLPSPQETSPYGSPVTKREPHNIIDGLLWSD

PSESESGCRTNVRGCGYSFGPDVTRRFLSANYGYAPPRRPARDGDDDEEAEERRGAEKPG

SASVEAELTESQKMQFIMRAHQCVKTGYQWTQERLVVTVFSAPNYCGLNGNKGAIAMLRG

AAQVSGSQIELEFKTYDSFKRVLSAPGSQIGLGRCENSVNGSNLYAAGGGGSSSSSNANG

PAGGANTSTQARPYQPPRNIVNNPVLEAYFGPSTR*

>Lp_000045100.1 Ribosomal protein S19e, putative

MKSSHGRERAPQNPDWYYIRCAAVLRAIYLRPGVGYGGLSKRFGNKKNYGSRPEHTVASS

KGPIHWACKSLTKHGLVRLAGRSGQRPTRKGHKLADPLAFQVRIRQLVASKQVRDLCIDY

LVEAAHGCRSFFFFLSRALRGDKQDGLLLRVSSRRFRGKHKRCRKKEA*

>Lp_000045200.1 hypothetical protein, conserved

MAIDFDLTLFIKETQLQHGLRAEDYVRYHHYTTNRLAALRQQLNLSNDKKKFLHKDVTAQ

NATDARHLMLLALYAERCWAEAEVMQVRVQAKREMRGGDANKPKGGVPPADQYRKRLNKS

VKWAAKLAEVANAVASERVNQECTAYLNDTAGRCHASHGNFKEAKEAFLLARATYAALRP

TSTEMQWTVIASKINELDDRVVYCMQRLGEDPISYHPAAIFSSCSGADGVAEDGTPQAGG

ASQLSWNGRTLNVFSIKVKDALREAQAVEVEATEEKLRETRGPVPVGQSNRVLDLMDRRI

NYYNDALAHARQDLRAAPEGAVKTEYQLIVHYILFHVAHETLRRTLFLAMLYSRRFEATE

RTLRAGGRVSGGGSGGSAASAAASQVIKQHQQQQRKKKSEIAPTQFASPLEVVRLYQAAA

GSVEEMELLPGVAGRGDVTSLQAVCLAGQLFYTGESWRVSQEWATAMQFYRDALTMLRGA

AGPYVAALQEHIEQRLMQGVAQVVLAATATPTQNSSSGSSNNAASPTVYLNEASEDAVVV

AQGVMRFPPDYQAAPCKPVFVDIASTFVNYPVEEAEAAAAPASGQKSSENSSNNNNSSAT

AAKPKEKKGWFRWGS*

>Lp_000045300.1 metallo-peptidase, Clan MC, Family M14

MPRDSNTNRTLKTLAKDPEPPSADAATNITVTAASSELFPRVRIFPSTGSLASVEDRQED

ERTHITNNHHNDNTNTAHQYNSFPARPLEPSSHAAAAATALPAITSPSSATAGSLSTNSL

NDFRGSPQKSSPVVHQQQHRLSKSSLTSTPPRSTETAQLARCSPVTTSTTERRRDCGTRR

ASKTDGGNRASSLSATPPAHPPSLNVRGSSANITSPPPVSFSFHAATPTAQRSPPFPQVP

TSPTPQLVQLRPASRSRPYISGGGLAGSPSSTPQAHGIPTGDALTLPSPSCTPHHASAAL

PRTPSGHQTPVPYPSLHSPVSLQYLSLHVAAPVPFSSIDNDDDDDNNATVAAAPDTTEED

QKDLLVEKIIQNPHKMGQSNGSCEKAYPPRGTSNSRSSPEESGSRPARSHSHAELTPKRT

DSLSCCKDGRRTPSASPANEKSAQERPDTPTVPLNSAEVDDADWVFQRPPNNQRTFYFKE

DNLEFSSQFDSGNLIQVERVGPFQYRMYTAMDCGNSAWQTNNRQWFHFSLRGGSKGAVVT

FTFVGMMHSNMFNFDWMPVTAVTPSRPEYTRLPGKAKVESLENMPETPGYPLLVYKAVSK

DDADSDGDNNEDDNDADNGTAANTTNNNNNNNNTSAGEGVAFSVFTTNGKCSAASKKKKS

RRKRNLAMNLTFDYRIEAEVPVTHTPPRGFPDVASIYIASNHPYTYSRLQRNLRAWKELA

QKSNATRSQSVQTAAKAVSSSCSEPCEPTETRGAQTPSNTHFDDNTADGSGATKSTVEVP

FTGIYYHSEVLCKTLEGHDVTLLTISDRSRMTMGRAPLISDKDGIPHSSATRTTQRPFAF

SGKRYVVLTARVHPGECPGSHLMHGCIEFLLHHTDPRAIALRHHFVFYIVPMLNPDGVIR

GHSRVDANGVDLNRMYRTPSRKRHPAPYAVMALLKSLGDRVALFIDMHAHANKRGTFFYG

NSMDGAEQVENLLYAKLVSLNTPYLDFRSCNFSEANMFAVGKSGKGKDSSSRVVVFTEAG

VVHGYTIETSHVMADAVNPIVALVNARGEQLEKVLPMPLQLTHTPATFGDTGRAMLVALL

DLKSINPLSRLSFTPYHSTRGVSLWLQRQLQIETAEMLFAQAFKAHGKEVQAVSHETGNN

LLGPIMRSLTADEFPDKVTIKKARLLPRTTYSDVRSFLPVETAVTLLSQTTPTGPPRSLL

CASTATNGNGGGHGRRRGGSLAAGANSGVTASSPVGEPPAVIATKRRPRPNAPGADMTAE

F*

>Lp_000045400.1 hypothetical protein, conserved

MHSLRDAPGYAEMEDYYDSDYVPNDLIARYNALLQERRRRTEQQFIAATQAHGFVTAHDG

LVLTTNSATSVDQNTQSLTNATLLDDSFSRRHPPDHPQHHQQRQQQRLSRSSLRSQQRHS

TAAVAGNTNACGGLRQGPRTSVALDAIYAARAVSYGNTSTSTASNDDVNATPSPSPAQRP

VSSSASPTPHHTATQKPRTQSRKKVKKTKKQASEQKSDMERKPVQHDLYDSAAEALRQER

FFSESKRLGKPFVPSGNSGLDVPTRFMLGDCVKILYRSIVPDWREASPMVVSTAEDLIAV

YFSLEKLEKSQVTALLQYMNACLLHNAAIREFHLTKVPEGWDVLTDDGYVLYTFRPPWVK

KRVFLPDTVNPLHAHLRDGDN*

>Lp_000045500.1 hypothetical protein, conserved

MNPSASAYVPSSAVAETLSRRRPTSPATPPSKTTAATSSDSKTTSSTNGVQLTAAERLRG

LKNISAASALQPPLKATEVALSAPASASQTPVLTLVAASHVPALPVTSSSNGSVVPHSGV

PIALKQKGHIRYTKQQLLSLRPAAMECVTRGPIVDHPPFLKIPESFARLLELPHALEYVN

VDAATRLVEHRSYVSPLLTSSSSARAYAAGPAAATVGAEENDAEDDIAHARLYRCAVCAE

GGPLPCPAIQRVIQRASKFAMSTKLGAALMQPALVVESLLRLALVFEAKQSSLRLRLLAE

RSPRVVHLVEEYLCGEGYHLPLPCVTAHAEWAAMNAMLKFDAIRDNKTRVITDLSKHWKK

RRTQFEQFVHFFAFRDARPVRPAKNIITQSVKPGMVAMLMGDVTAVLLLHRLRFFIPPDY

YWSVYPFLRQSLGEGTLAYDVVRHLGLYFSSVQPADMYNPVERPQHVM*

>Lp_000045600.1 WD domain, G-beta repeat, putative

MQQWTLERNSESPESTGRPSLLQHNTKLFKSESRCARRSGCVELRSRLSGVAVSAEPLNQ

HRHSNDFHASDEGLEEEEGRSSVPQRLSRHIPQRVGIDMAVSSFNLISSSVCGAPPSLAS

PRNFTGLYAGRGNASALAGGGAAISGAAGHATTPGSAAGPHPSSVHATAAAATVSAGVGG

ASVVATSTVPPTPASSALTSFSESMVSDDSSYMTSLYNDVLASHLIGHRPSSPSASGSTS

DSRGGGTFGYGRSTTTTNGPHASAVAAPIGDVDDGRRVSETGSEERSGGRRPHAEAAAGA

AAARRGLGGEVLRFADTASSEATPCPFGAGSPVDGPQVRVVAYTTKGTRPAPALPRVHSR

TFYTGGSGSRGVGGPRVFDTTPERVLDAPQFPTDATQLLDWGANNTIAIGMGQSLYSWSG

DSGQAAKLVDLDSAARIRCVQWLHKCSCVALSVQEGTTAIYDCRTSDFLRTVRLPVGLQV

TGLSVKGPVMAVASDGLHGTTCAYDLRAKDALIATYEGHSEGIASLHYCAAEPFYLATGG

RDGSVRVWDARRAATPRYAFDGVHQGSVNVVRWDPQKRSRLFTGGEDGVLCLMDTHAPKR

TIEMDHNADEEGAGMGSTLEHLAQYITRAVNTQFPISGLACHGTSGEVVTAHKLKGQLQL

RKTSTFHLLSTFTALHCDAALSCLTLAPDKESVCAAQGDDTLKFWRVFDNNAARSSERGR

AAGGSRTVWNELHQDPSECFEDALR*

>Lp_000045700.1 heat shock protein 83-17

MTETFAFQAEINQLMSLIINTFYSNKEIFLRELISNASDACDKIRYQSLTDPSVLGDETH

LRIRVIPDKANKTLTVEDNGIGMTKADLVNNLGTIARSGTKAFMEALEAGGDMSMIGQFG

VGFYSAYLVADRVTVVSKNNADEAYVWESSAGGTFTITSAPESDLKRGTRITLHLKEDQQ

EYLEERRIKELIKKHSEFIGYDIELLVEKTTEKEVTDEDEEEKKEGENEEEPKVEEVKEG

EEKKKTKKVKEVTKEFEIQNKHKPLWTRDPKDVTKEEYAAFYKAISNDWEDPAATKHFSV

EGQLEFRSILFVPKRAPFDMFEPNKKRNNIKLYVRRVFIMDNCEDLCPDWLGFVKGVVDS

EDLPLNISRENLQQNKILKVIRKNIVKKCLEMFDELAENKEDFKQFYEQFSKNLKLGIHE

DTANRKKLMELLRFYSTESGEEMTTLKDYVTRMKPEQKSIYYITGDSKKKLESSPFIEEA

KRRGIEVLFMTEPIDEYVMQQVKDFEDKKFACLTKEGVHFEETEEEKKKREEEKAAYEKL

CKAMKEILGDKVEKVAISERLSTSPCILVTSEFGWSAHMEQIMRNQALRDSSMAQYMMSK

KTMELNPHHPIIKELRRRVDADENDKAVKDLVFLLFDTSLLTSGFQLDDPTGYAERINRM

IKLGLSLDDEEEAAPAEAAPAAEAAPAEATAGTSSMEQVD*

>Lp_000045800.1 ATP-binding cassette sub-family F member 1, putative

MEEDQANVAAKTAQVKSMTRKERKEVEKREKLEEELRQLTKKANAVNGDSDNPFSVTMEA

DQVVEGSRNITFNKVSVSVNGKTLFKDASVKLSAGSRYGLMGPNGRGKSTILRLLASRQL

PVQSNLDLLLVEQEQEFTASELSAVEAVLQSHKKQHAYSLEAKELHAKTELTTAEMERLH

FLEEELDVMGASQADARARRILFGLGFPTEWHERPTSSFSGGWRKRIALASAVFIEPDVL

MLDEPTNHLDLNAVIWLESYLVKAYNESAKRPKTLIVVSHDASFLDEVCTHMVHVENYLL

NYYRGSYSDFASQLQQRHQELDKKHESIMKTIRDKKRNGMSNVQVDAWVKDQVNAGRLEP

QFLEKRRDYIVNFPFPDPPELRDGCVCRLENVSFNYPNSPVLFEDVSCALWTDSRITLCG

PNGIGKSTLLNLMTGVLEPTAGHVMLNRQVRIGRYNQHFVDKLPLEKTPVECIQGLGISA

EDKARRLLGSFGLEGIVHKNQIATLSGGQKARVALAAISAETPHFLLLDEPTNHLDVESI

EALCAAIKAFKGGVLVVTHDARLIESTEMQIWVAGNKNVVPFDGSLEDYKNLVRAEFEKE

EAMRLEDRRQQQEDKAVIRQLKQSGVADVKQALQEQEEEKQKEHADGLDAFLNAVNKKKK

KSKKSKEEKE*

>Lp_000045900.1 beta-galactofuranosyltransferase-like protein

MARFRFLGLWYRRARSHRRLSCSAVVALGLGLVLLCYFDLFKNVWNLSFLSFFQPSKDKA

VSDDELFQCTEQWLQCSGVPANTVIPFVILPLTLEWEEFGRFMCHVNAHTHYLYIVQNGN

VDEMTGLLRRLRAAVPSKQLIITQHPNNIGYAAAVNEGYRVALAKPHDEVPFVGVFNTDV

DFGDGFFESYVPDVYAALSPDADRIRELEEEVTQEEQAAKVAGTKTLRATASSIPGLSLA

SLTPDRVRYAPAKEREREFSQHVGMFHFNYQCMCAFFVSRLALLTGGFLDENCYPAYFED

YDWQFRMANLGFRTFIGSAERYGNFSHHVGGNNRVIRQGTKGRSLSYVEEAKRLSRMLNA

KPGVDYGESKWAWHREANLAMSLTPFLTVGFVIPPDAWILDQNRLAQIAQIGVGKQPAVE

MHNTYNLTLLEALRLFDKTAK*

>Lp_000046000.1 cysteine peptidase, Clan CA, family C51, putative

MRDRRSLTYGKVPGDDASGVKPSSSSSASAAPRRGKELNVKDTALCDEYAIRLRREADDL

ERRRPSFWHMRCSSSALIGLLIWVVIGLVVYYSVAGNVSSANMSTSPITAEFVLPEGCRG

DYCLQEGGTEAFGAVLGAHDGVYAYSNCYAHSCVSFLDFVYPIPLPPGAHTPLDDPKTTT

RLMRTGMRWQCVEYARRYWMLRGTPVPAVFGSVEGAADMWDELTFVTLLDNVTTAPLFKY

ANGKALGHGGSAPRRGDVVIYPRDLEGKFPYGHVAVVVGVELPASPADEVAPAEGRVYLA

EQNWHSSPWPGPYHNYSRWLPLRVSTASPGAPLRYTIHDDYHQIQGWMRYGDP*

>Lp_000046100.1 Tyrosine phosphatase family/Tyrosine phosphatase family C-terminal region containing protein, putative

MLSDLQLQRHVELEGTTNLRNLGGYHTKDGTKTTKWGVLYRSDSLADVPADKAQSVLVNQ

LHIHNAYDLRSKKEVAQNAYHIPHINRYAVSIDTRELSQALRDGGDWLNDTAAVVKGMQD

IYRDFVNRHGKAIGTFIKGFLNSKPSPDNAAVFHCTAGKDRTGWAADVVLTLLDVVEEEK

RSDYNLSNCYLECPKEIAEYLLSKGMSKEVMTVLWGAVDEFIDAGMSEVNKLGGMTAYAK

SHMGLTEDDIQQLRDVLLESQKSSY*

>Lp_000046200.1 Tyrosine phosphatase family/Tyrosine phosphatase family C-terminal region containing protein, putative

MLSDLQLQRHVELEGTTNLRNLGGYHTKDGTKTTKWGVLYRSDSLADVPADKAQSVLVNQ

LHIHNAYDLRADKEVTVKSYEVPHITRHVVPIDTTHMARWVKEGEDLHSGPVTFRVMQEI

YREFVRSYGPTVGAIIKGILASSPSSNNASLIHCTAGKDRTGWSVYVILSLLDIKEEEKR

SDYLLTNAYFKTPKDAYDYLGGLGMGPDAMKVLWSVFDEFLDAGIEELSKFGSVEAYAKS

HMGLTDDDIQQLRALLLE*

>Lp_000046300.1 hypothetical protein

MADQWGEQWLEQQSLVREWLVQESLVRESLVQESLVRESLVQESLVRESLEQQSLGQQSL

GQQSLEQQSLGQQSLSQQSLEQQSLGQRLLEQQWLGLLWSWCSSLRK*

>Lp_000046400.1 hypothetical protein

MASSDKNKASSPEKRASSSSSKHRSSSGSGKHSSGSGKHSSGSGRHSSGSSRHHSSGRDG

RSSGSSSPHHHHHHHHSSESSPSKDAAAATKPSSVSTAAANTTAPTKAPASAIPPQRASV

PLGASAPAAGPAKTVSATPRTLGTTPSSAVKPAAAATRAVPPPGAPAGPAAATRPAPSAT

PSAVTPRMVNGAAGGAAGGAAGGAPPGLQANSGSMMGSMYGPNGNPAAGGYNGAGGYGSM

AGVGSQNALGGYGSMGAGDPMSGYGSMNGAQEGMGGGYGSMTGGKDGMGGSMYGGQSGMG

GGYGSMYGPAGSMMGAMGGSMYGSQSGSMYYNGMGGPMSGSMYGMNPMMGGSMYGMGGGS

MYGMGSMYGMGSMYGMGSMYGLGRYNSMGLGSTYASMYGLGRTTSRSSFRSTGGYVGGSF

GGLWRA

>Lp_000046500.1 hypothetical protein, conserved

MSAVVPPPPPTGSILQKQSSFSRSKAGATTTAAASAAANKAPSNTPQTKPETAPAQTPNL

MAAKPAGPMPSTAAAPTATPSRPIGGAAPPPPAGKWGPNGQLAPASTGAYPPAMSPAAGV

KRPAQPPANQKMGAYPPAMSPAAGVMRAAPAPGPAGQANANTVAATPRSAPAAAGGAKRP

ANAAQVPPTSTPRAVNGAATGAPPASTPALARKNSKNGDMGGGFGSMGPGGPMGGYGSMG

PMGGYGSMGPGGPMGGYGSMGPGGAGAAGMGSMYGPVNRQGSMMGPMGGSMFDGKGANTS

TLGPMGGSMYGQNPNGMGDAAAAGSMYGQNPGAGSMMGPMGGSMYGAGGLFGSMGPGSMM

APNSMMGPMGSMYGMGGPMAGSMYGGMSTYGIAAQWGSLANMSAAGRGSTLNRSGISSEY

GGFAAEAATVDLDDLCPNIETFDDAKDLKRSGKRGGNHLYSRDKHEKIVTTNNSNAQALT

IKRKADVKASSAAGGSASPAADSSARSTKRGTAAGAAPTGSSSTSNGGKARAKVPSNYNV

HSLIVVDKAGKTAVTTVKDPSTVVYEKGGKKETFEADEAIERAGEKEEVDSVLLSELRSN

WFNGHCSSLMMCAGKGKKDAAQTVVRDFMQKCIDRLEKNEKETGVKFDVTMTMVALRGAD

QCCDLLKNGADYVKMAMGSSPIYGPCLRKLEAKVVKSGAEATSHFDAGLKNAKEEKEIVA

VFYVLKTIKKTGAEVDVHLSSMCVALCGETVTHMTDIKDKAPSSPHRLFRYAIDGACVTV

SGLCISSDDEEAQGALEVERKVREVKNMPPRSGNVRRFIEFTYKEIARQKEKASAATDEE

KKAREAQIERMEEMVKDANELLSEPEKTQPKGYSTER*

>Lp_000046600.1 hypothetical protein, conserved

MGGSMYGAQPGMGGSMYGGQSGMGGSMYGSQSGSMYYSGMGGPMSGSMYGMNPMMGGSMY

GMNPMMGGSMYGMGGGSMYGMGSMYGMGSMYGMGSMYGLGRYNSMGLGSTYASMYGLGRT

TSRSSFRSTGGYVGGSFGGLWRAPSGMGRSGGKKDYSIPPPSSKLSEDFPASNSEDGKKQ

QSSEPSKQRNVDAVAKDKDGTAPKRDMKGVTPSVVVVDNGSSKGDKTKLRPAPVAMPSSH

RLTKQVNDNVHVVAVAPSESVANKVDKTVKVGETTYTMDEVAVGSVDIEKSDLLTDIVEQ

THCGHNVSLLALCGSQKYEDGIPAITAVVRSVMESFAAEDAQVTQVMASAVVFPEADKIV

DLLLDDAKAKPVKVELGANPIYGPCVVNTSEKKVETADEAAAVVALAAKKAEQRGLVVVT

YKIKQIRPSTSSSGTRDVYVSSMLVALVDDASMAYVEAAEKHSTTSPAPLLGNAIGGASR

TVAIVHIPEKDAVKAVGKAVASAQQLREVKNTQTRSGNVKRFVDYTERATEARTKTSPET

TAKINQMLKDAKELLAKPEETPLVVYSPFGAGHSSSPVTTAANNTNRDGTATRTPAEKGT

KSDAAQQAGEDDKPETERRVKLTVCVDGTKAMPQGELSEVVMRATPTMVPESAMLKSLRH

NFCRTRNIALVSAETQPSVELKNQYTWKCVESILSKCLSEPASSAKSTVVELFMVVIQKR

QMLCDLLEDSSTPKPLAVASSPLFGPIIAETKHKTLCKADEVQPALAQALARAPPHLTEA

DALIVMTAVLKQVQKNGDVSVASFMCTSGPSGAGVRGAVSRNPDYSRSLYTYALGGPCVT

ALLVSVGDDHATNATVKSSLEDLMPLTKQQNHISRDGSVENFLDYAKKSIAANESRLERA

TSEEERERLKMASSRLQVMHDDYSALIKFPQENSPAFYIGEKRVSEALRTDDKPVAEAAS

AAPNSGAEQSRDSNRRAPVPIRAVVVVVDGADDEAAATNGEADAAMTMTVTDKEIALNGD

HYTPSEVVMSKGGMLRSAVIDEVHQVALNGYNAALLTNDVSGSTVGISMAVKTIVVILRG

LPQNSEAFWTVVVSKDNKVKDMLADNSPYYDLHLASSPLFGNVAYGANISPVTEAQVEPK

VREVRNEVRENGGVGYIYVILRISRPDGDVCMPSFLATIAGDSVNEYDKLLDTHDKNQLL

STAIGGPCHTIYVAGIRSLSGKNAKQMLDVAAKMMKVQNPPLRSGSLKRFIAHTEPSLAG

MQHKLESDGASNPMLLTQIGRISTMLKDARSMLNSPGGSAPAVYKR*

>Lp_000046700.1 Sugar (and other) transporter/Major Facilitator Superfamily, putative

MSGEAVNATLSTAFVSADLQAPSSGNAASALTSVNGDSFPSPLCNTSVVSNKSGKTSEVS

STRPRKGLTNLSFQLPQPTASVERRPSLREREEKEANGGVNGDADPRDVDSVCTEGHLYS

DPEGSHHDSNDELIENNAELTEHLAHGTYNHHEGDEGHSGCGSNTGSPQASVDDLLGNVR

EGGSPNSLPQGFSSTNSTHSPNTKKRTRVNLLHLFNRVNIKVTVVPIIGGLISGYTIALV

PVYSQLFVSGTNCALYTAQVGCEAVPFADCVWRTTTTTQSDGTTRSLGYCGWPTITCREA

YPNDEWMGGGGNTTLAELNCLQDRRCTWSYSAKECQNPSGYTTRELAIFAGSMIAGNMFG

AILGGPLVTSMGTRLTFLVSGLLSVVCSVMGHVDAATDEFWVLVTGRFVLGIFMGLITVA

CPLYVHENADPFYKAKIGTMFQIFGTIGSFITAII

>Lp_000046800.1 glucose transporter 2

MVAHSLEKEAELPDRWVEDLDASLDLSKNNNNNNNNSGILGAGADTAAPARPTLRGFFSR

ENLTVAAVPFVCGMINGYTIGYVAPYTQLYKTSTNCALYTAQVGCEAVPFADCVWRTTTT

TQSDGTTRSLGYCGWPTITCREAYPNDEWMGGGGNTTLAELNCLQDRRCTWSYSAKECQN

PSGYTTSELGIFAGSMIAGNMFGAILGGPLVTSMGTRLSFLVSGLFCIVTCVMGHADSYV

NDFWVLASSRFVLGIFMGLITVACPLYVNTNAAPLYRRKLGTLFQVFTTFGVFLAGTIGL

GVGQSVQFGADDNADISGRMQGLACGQTALSLAMIFLGVFSAESRLKYGKKVGALNQNEY

SYRKMMPQLFMSVVVNGTMRMTGFNAILNFAPTILGGFGLAPLVGNTVMTVCNFLGTLIS

IPLDSFFSIRSIFLFGSCFISCMCLFLCGIPVYPGVASESVTNGCAITGTLLFIVGFEVF

VGPNFYVLCQEIFPPSFRPRGNSFAQLWQFIFNLVINVCYSLAGGGFSGGPGGNQHKGQS

IIFIFFGGVGIVCFVIEFFFLKVWNEVKGDPPLLATAQPEKEIRGTQGDASGIIAVSAAS

RDVHEAGNRLAQESEIHTERDMDQ*

>Lp_000046900.1 AAA domain containing protein, putative

MTSDVLSSPKNKIFVDVKVVGVLGCSASGKSTAAHQLAQCLGSPLYPISTDQFFLDEVCA

QLGTYDDYRCLDYAKITQWMHMLTRPFHYTSCSPSSEEGDAVDLDGSWKDHEAQARDEWC

AAMLLRLPDLKPYFRVQAEVSAEDVANASAEVREDLKVLHDDDDADDDSVGDDDSLCAHE

AEEAERRANAVFAATTLFPRAPSRQRERLKEAGPSTTSSSLLHRITMYVVWEGFTLLCSA

AVNAYVDVAVHVRCDFETACLRRFFRSPRHHLVQHAIRPVQEQRDVDENSNNNDDDMDEV

RRPRSETGAVVVARVVRQLYRTRIEQMWTVRSRTAQREELLHDILQELREEITDDSDGVA

ASSCSSLAAGVRTVECLLGAEVFNPPRPGFLNIVVDENNRRAVCAAVCDRTSSGRLCWTD

EGTPTLAFQHFWENEFESWLLRASSLSGVSPLSWAALGASSASEDFLCDAGSTNRGRGAR

DERPAFTQNVAHASTTFDPASGDATTTVNSAVEDNFTGRAYVEKTLAERGLQALRQGSTH

TTGAAVDKCNSNKNSAGSNAAHEATVPGMPSPSVVAKALAPFYYEFRYWFFFEVLYYDRL

LAPLQHHRLRHRSTLNLSVSEVSERRWWTVENGHAVQGKKEEELLQQIQHIATSILSSSC

*

>Lp_000047000.1 histone H3, putative

MSRTKETARAKRSITSKKSKKAPRAASGVKRAQRRWRPGTCAIRDVYKRQFQKSTDLLIQ

RAPFQRLVREVSSAQKEGLRFQSSAIMAIQEATESYIVSVLADTNLACIHAKRVTIQPKD

VQLAMRLRGERH*

>Lp_000047100.1 hypothetical protein, conserved

MAAFPTRRCAIALLCAALVLVAACARANSIVVNGDVSQSDNAFDDNSITFDLGSVSADVV

TVQLINSKVSGSGLSIVGYEDSVPSSVTTRVSMSVTSTTVTQSTIAFTGVMPPNSDIRLT

ATTATLATAQSLFDFSGLTLSGNVTVTVEDSSVAWPSGSTNTGSIVTYTAGATNIGISNK

GALFILNATAVNGASVLHIATSSVFSITDSGVLAVDYGGCDGCSSALVTIDVPLKVDGTS

MFRIMHGVVGNGAKGLLASTGYVTVSGQSLYLISDSTIDSGSFFDYHVSGNANDSTAFPF

TVTSSTVSFLNLVGPSLGIPDGAYVPSTADSSSTVNGGGCTIGGTALTDTSGYLSKGLKV

TQVVNSNGAAGGTCANANCVPGYSSSGAAEADGVACSCICTAKIYNPPSCTTVSDPTQNY

HSATCSLANCATCSLLYPSSRCAQCNSGYVLSASYQCELDNPDNATTTTTTTTTTAQPTI

TSTALCSVAYCEKCSPTDGSTCTSCRNGYKLTSGACIANLNGAAAAQSALLAAVACAAAA

AFYVL*

>Lp_000047200.1 hypothetical protein, conserved

MPDFSQFIKYLNANLPAPTSQGAAQVSVGGKGISSPNGTIEGLGGPGHSDTVAFTRSCDT

TWRMLLPSDAAAKESGARSSAATAAAAHQAPSHPTVTVGRMMVSTEDRWRIQAQMRREEA

RSGQARKEYNKKVTRAIKVTKGPSDNAFPPGRGRPL*

>Lp_000047300.1 exosome complex exonuclease RRP40, putative

MAGAAATLPLAKGVAELVPLKRHVCLPGDPVLMVQSGAVVAVGGGLRLLAQPTAASSAQN

DVTDVFLAEYCAPLEKSTHHLHTHIPRYTVATPASRRYSPRSGDPVIAVVARKVSQHYYY

CYIGGTALAYLDAVAFDGATKVSRPRLVEGDLVYCYVKPRTAGTYVDGATGASSSGGEVE

VACTAAEVGLPPKDWTSGEAVFGPLHGGRLLTLPLAYVRRLLAPLPTAAAGDEAGSSSGP

QHRKRAREEGSEEAELPASFLLQLLGRRVPFEVAVGLNGLVWVRGLSSETDASVAARRTV

AVSACITEAQYDATRAEMEARVESYFPS*

>Lp_000047600.1 hypothetical protein, conserved

MQSLRRVVPSTRLPCWRLRLTARSSTCGSPRRTYTAGSEATQAVLHPADLTHSLSTSVVA

AAASGVCGAEASAAHDAPPSPVEVHQVIARAVQRGRWTHAIRLLLGALQSRCVPLAETYQ

LVLLAALRGGGWQASVELTEQVATSPLSTTALYHTAADLLLSQLGQESHDFSEALTRVVV

DELVPLMLADATSNVAWKPRHRESVLRVLAETNHPQLVSQLFRRWSTTAVAAPFSFSPSW

TGAYACGEREAALLMSAFATTGDWRSAETLRVRLPAAADDLLVDYVRAFTVALKNSNAAQ

TSAEGRDQLVPWEVAMDVAGRHTGCSTLTAAAAQLQRVAHLATAPTDVSRWWWSAETTVH

ARLTELRRRLATGHNEVAELLSQCHVTTAGVAELLDAVADSGVLSSHGKGASPYWAEWLD

VVLTLSRYCPNALVSAKGCADGLSRLLPEPHIGVPRVGASPSPSPVSSPSAAPHVGGDGG

DSVEPLWRRVAARMLAEMTPHDGDDGELRCVLTFTATLHYLLYAQEQAASPSSFSPDSTA

FSISTAVRDACALWTQRLHRLQSSREGLSHHPRSPALVECQYAVAVHLRTVSVYAKLPTL

EQCIAAVLSDAAATTALVDTAILACMEPLGQLGSAAALCEAMCTAFPVDAPAPSHAAFVL

LLCTCQQLRDGAAVAAVRPLLRRVESFLRTTPHAEALMVLSAQLQWCWGGSWEGLRSLAV

PVLLSQQRWAILARLLACCPPPLSAQEQRAFAACVAGQRLAELQASVSAGDVAQALQRWD

DVQRSDDRGADAASLPAPLHAALVSLFAAHRRTMEAREVLAVEAAATDTPWPSLGLRVCR

RVIRHPASVVRMSDVWEAAQVASGSAVVKREATAGLAMYAAAAYSLLERDTEAFSLICDA

EAYVVRYDASHHHPPPPPQQQQQRCTTFMQEEENSNDGASGDDGAYSFGSAVALHKTDLQ

LAKGMPLFVQCAMPSILLAALSRAAAAGAAVESGRDAAAVWRVIRFVFPLTSHTGAAIRA

WAAVLPQLHAAALKLDNEQSNEIPSGDNGAAAAAATVPLVFSLARRLLHEAVEQRIAVPP

VVLELAVTAVAAGAFPPDATMELLNGIRQSLVSQCNDNGARAESSSLSAVRSIASQVAQL

LAAQGHHAQALEWVNEFGLWSATDESRVAAETAWQWIQASYAAAQRHFDERASLHRRHTS

ATSEEKAATRHTAPTEGLSLQSLDALLDCGAWADAFSCFLRVVAAPTARRAAATTDAVEF

SVAAMETAELRDAFLSPHVLNRVMKSMAQHAPWRTCMQAWLLLCTRLPLLPWCTSAGALA

PALDQLLSAMQQQGAGPHEVGTVLVWIVALLDPSPAVVATLSQRFSSAIATAAAWTRADG

EACAKLLLRLHRVLGEGRVRAAAAAIAEAEAALTVAPPLCVFGEGSSADAAGAPVASEAW

LPPGRPALSSDEVDALVVLAPLLLLAATSRLEVLARRHLGGRFTGYELKALLWLYRDELR

QRVNAAQESAGDDAVVQSLSQNVALHVCASQAFAQVPRLDVTDASGAAAAWLAEEIFDGL

MPLRVASRLWAACHLPAARLAACRWAPVVEAELRAEVLRFHGRPPARLRTAPQLSRQTIA

HYWSCFHRVLAPELHFGPLELCCLAAYAPAVAEIRRRHRDTTAAGYRADMCELAARVHRA

HFSPSRVPAEDTLAYLRRPHAPAAIVAVMTTAHKTLKELHVQLFEGTAAGAEEAVAHALP

PRLRRHFAACAAAVSSAEWTPSRLQQQQQQASLAIAAWRLTAVAEAERSVPHMPCTTTLV

GASERADVQTVTTSPHIAMLLSEAQLRLPSLLLRSWVSWVLVECAVPRRPIPPATSLLLV

EWTRAALRALNVVAFADNANDDVACSGDVVSQWRAMAATGLHELHHRRCSELSVVWQLLS

EADAWPLMNAGHRVALEKMVWPTLAQVKGLPAKTRGNSGVPPNAAKKNECR*

>Lp_000047700.1 hypothetical protein, conserved

MRRFGGQVSLTVAVRCAPAVCRSLHSGPPTAPPAVAASASSPVASPSPSPPALPTSEDYR

ALLLDLLLYRKETLEPLLIALRRERAELDVKMAQLEAHVRELRGIREELRRFAETKADEE

RKKLHDAVMKAAKPPQPGLENQEPSQKQGSDSDEIVL*

>Lp_000047900.1 DNA topoisomerase type IB small subunit, putative

MQPAQTPSTAPPAVPATLPKKTPINVAALKLKMSPAVLAALTAAKAAASQPRPSDAAADE

DALLGLKPAATVAAPPSKMRRVERAPPSSSSSSSSSSSDSDSSSDDSSYSDSDSSDSNSD

TSSAASSSVASTESEEATLFDIAQSQGLVNREVLSQVEEVEVASAVPPRPPIIRSFPTDV

EKALARYQERLNREENLIRIKDDNKAVSLGTSKINYIDPRIVCSWAKAQDVPINKVFSAT

IIKKFPWAMNAENFDF*

>Lp_000048000.1 hypothetical protein, conserved

MSSNDVVVKRRPSRPQVHPEPHEAPHIAEAPHDEEAADIEAAEKVAHEYEEVHYMERRDA

ENEVTEEAAEEDAEAEAAEKVADDYEEADEAEDDDAREEAKEEESDAAMEVVENVAHKYD

EVGAAEDDDAREEAKEEASDAAAAVMAGRKVAHAYDQVRYEEGVEAEDEAPGELAEDAEI

KAARRVVHDYQEVGHEVEEEAQKEALEQAAEVAAENAAAERVADEYEEVAEESGAVPSRQ

PCKPSAVGAHHHADLAAESDDEDHVCSPPSAGEAAPVAAAPPQPVKTARPHDAKAKKTKK

TKAIKKEADAAPAAAKPAASKKKKPTRRVNNANVENQPPLPEGVNTPRSVAVCQKHGVNP

AELAPYPKEHFQGPGVTEEVAELRYHSYEKRRQARMAELLPAYREAAKHSSSRPKEKAAS

CAAAEEMDEEAEEGEEAEEAQRPTTQPTNEEEKMQQQFEAQHKRLLEQEHRKASGGGACD

GDRYRRASLRASYPRGTSPFGGASSASSRRYSASTSALGRSTVDQPYSALKIYSHSISEE

RPLTQSEAVMIEDINEREARRLDTQERAVIIQENKQLMFVERELEKQRRASASVQAKAHE

RERLQQEHFKHSNDRYNEALARRQYLEEERQARLQESIAEKEGQVHANDPYASLLSRRRQ

SASMIRRASSNASARPEQHEIEADAP*

>Lp_000048100.1 hypothetical protein, conserved

MAQWIPKTAWKVANLNKRYGTPYMSKGYAALDPQCSLDAYASLQHNVSAETIRNAVTAIG

GAAAGAVVIDVRNEAERRRQPILSTAVVALHPHDILSGAAGPILPADQARAELFVLASEM

QRAANTCAALRRWGYTNVSAVSATAVVEALAEAQASMPTAAAVSAP*

>Lp_000048200.1 hypothetical protein, conserved

MFTPYPLQRQQSANGMQTALVYPYTYYPNVSLPYPVQSYGSMFGAYGSAPMMVPGTGNTT

HSVSTYVGHSATPMYFVTGPNAGVSATTTPPTRAVNDAAFPVATACCAALETSNAGACTK

LHHSGSGHSLSTQSSCSGGESASLLSSSVTPAASAIRTRPQHDVRTWGFALVDQLHPSLQ

RIPVPPSCICEPTMGISLYDNDVTTSSTRPCKSSVCLLHAQGLGCAEGASCRCFHISPQY

LQQCRSTTESLCCGLHNCYYSQEMLASNCVPHLMNRRFVLSLDDASRFPQDGDKPYLIDL

SLLNFSLTVGLETLPFVDGAHMISWKKHVCHLNMDGKCKWTKDCGHIHMCRQLAKVLQDA

ESLAVIKALQAKNTQKSSADLYRDVILSETTLRYVRSSSVYPLVASLIEAGDLDSLRALA

KAKCALLPSQCVALQKLGVKVSMSDSVSRAASVSPKLIGLLALKH*

>Lp_000048300.1 Protein of unknown function (DUF1637), putative

MKRLIGSLQHFSKTSTEHVELFSKLTLKDFGIYVSATDAVSYTHDNNYPIDDKVFLLKNS

LFQCRYDATALRFVPQPWKMSAVDQLGCSTLYQDEQVTLCWFVIPPGRVLPLHDHPGMTV

WQRVMHGRLHICSLVSDAPPATMKSSPDATPATVVFSGEVDGIGEAVYPARVLTFGERDG

SMLHEIRNVDPERPALFVDIIAPPYYQSPTNIPCGYYGAEPVNKNTENLEGLPSECGIHW

MLRAGDKALLHPRPDYLGPAMDAYVHVA*

>Lp_000048400.1 FAD binding domain containing protein, putative

MLRRSFCRRVAVPAASTNAHDVIVSGGGLVGAAMMASLQQLRSRLHAEGHISGASSGSTN

NSAKNADALLSSQLSRLMLVDSGKRPTYDAANLMHTLRTVSITPVSSKILDNLGAWDRLT

TKHPYYRIAVRHEQANSPTLGSGARSTSFFMTSVLGNTTSAEPLLEFTDLRKPVGFICYN

TELNSCMVDVVEAQQVTAAKVDGFEDTLCFGHHLEGVTLPSSSSVDGPWGTAKLVADKAQ

QDVQFGLLLGCEGRGSPLRDVLSTPSLQEDYAQTAFVCDVRLEKVDDGNVCSFQNFFRDG

KIIALLPTSDETANIVFSTTPQHAKALLASSQEDLVAELNRRLCAFAPNDIPKILEVPQA

TSAVDGSVRRAQGSFPLKLNVATTPYAPRAILLGDAAHGIHPFAGQGLNLGIYDICALNS

VLEQAIRSGQDIGSSVAVGQVFAGHMLGHTAPMITGMELIKKLTYGTPGLASVGMKVLNS

APLVSTVAKDAILQVSSGALFASQHKGCFLLQ*

>Lp_000048500.1 Transcription factor e(y)2, putative

MSGANSSSGHIAYEVMANVQKAELSAYLRDELAGEAHGAQWKSQMRLLVQEALGRRAASG

ESVDAGDILAEVMPRVRAAVPEDVREGLFRRVAAQLNAS*

>Lp_000048600.1 hypothetical protein, conserved

MSSVSSAEIAKRIREGNQKRREEERKKQQLEQEYLDVVSEKMQKESQKRSPTRFLPNIFI

FLLFVSFPLSDQVVDWHLVCIFCALNAVLALAFTNPSDWISVTSTVIINFFLVRFSTEIY

DLPDRLLQVPLVLLLNYVAVNALIVLTAYFLYVNPRFLDYKHRYQQPQQRRGTGKKKTAM

SDDVAVLEAFAKEKAKAERLDLLLCLLLLLNLAALVYLDVIPFEAVRQTGVNVFRLLK*

>Lp_000048700.1 hypothetical protein

MGRRRKHNKKNEGGSTTTPPPEENDTEVDNAGVPSSDNVLPSTTDEAEANLTEPAEAASE

PAPTTEAAPTQQQQQSATEVSTAFEATPAPPMPTPSTEVAAGDAEVDKAPQPPTTTTTTT

AAAAAMPATAPLEGVAALNAYALENASTVSPTMTAALHSKPAAKSEVENRKLAEGHTVTE

ETSGAPTSPPRTSTKKVAFVTPVVSSVTSVGETDASRESNCIEARCGSCSVM*

>Lp_000048800.1 protein kinase, putative

MSSCTDSQLDDLPREELLRLLKELRHAQTTQTESSQSNRGTTSTSTSTNNTGSGRGADAV

MSASAHVKDLESCSENTRNSPNGPQDRSMGRSRPNPHTIRGVLSTHSESVSATPPNTSNT

VAAVAAPQEPGQPRLLTKTASEDEATADSGNGTDDGVPAVPKRSDTGVDVTSSQTEGSQA

AAETEKRKAKEDMSAKMVTSTEPGTSTGTIGAVESLSRLSLLSNTLETVNARSPCSALLR

APAAHHRAAHALAGRGASRCSSSTSRSSSYDTVEKSAADSALVASPNRSPQKDSIPPQPQ

QRTRSLRSPKPGGQAFSTLFVPQAPLFNNCGATNDPLCVALSTTIETQSPVTLSSTTDGD

RNSILLSSEASIQPTQQETLPPPQELSLHAAQTTSESNLRSFSSTLSTSETFVGMLQQQQ

QQQQHQSVVHPLQLSAVRPFTLEQQQQQQQASHTPSLSPSLSSAMVPTLHEATAEPVQLP

DGSPFEHLSPSPTSLSSRTTTSTSTGTAMVTTTTTTTTTTTTRSTTPTHFGSFTKSSDVS

AVASLLSVTPCFGQSMQSPLDIGDCSSHAPRLTMPQCNTNTAAITAIGDGRSPERRPLST

FEGAGHRFNSEGEVTVMSPSSLPSAVVLAMSSNGTTFFSPPVSPAPPTGKVAAKRPSDAS

DSRDEVVPHRFLSIDNSTKDSAMRQGSIEQANGPAAGNEGASSADVPRQGRKVILALDPD

GLTESPQGPLLKRRDGPELPAPSCVPSTSSPTIQSGQEKPLIPDSEEQSPRPFALAARRG

NMNATPTCVGTVTASISTLTSVETMHITTSTPKAAEDAASTKFKRWTQIVSWDKEKNGSC

ASCAKGHSSPVKRSTPAATSSPLDQRSVTPLHVTPPTSSPPSQPVSAPAPPSLSDSSREV

VKARQTASLRRVRHARTGDRYINNYRILKSLGHGSCGKVKLAYDEVESRLVAIKSVRRVD

PRKRLGGLTTAQKQYNAFMREVEVMKRLRHRNIVSLYEVIDDPSADKLYLVMQYVDKGVV

AKVEVRANSDYVCDPVPPPQLAYYAREMLTGLQYLHRHDVVHRDLKPDNILVSKDGHAYL

ADFGVAETFDTSYLQRRESIMQQSMAMSVAGSRAGGPQVLGTKGTPLFIAPELWDGAKSY

GKPVDMWAMGVTLFTLLVGKLPFRSPEDITDATYTPTVPEEFGEQWRVLLGGLLNRDSSA

RWTVEKALQYVVTNFVEREIQRSASEPNLSDVLCGSRAAVNSVASAPPTFHDGSTTPPNT

LTASTESSSNPTQEAENVKEKTEEVSDDNNKTRKGELSGASHLPSTLRRPFNESARMRME

PVSPGPALHPHAPRQQGGAAPTGDELVDVAFSTSSACPAPRDAVCEVKNSPSHPPRVVTA

AVAVHGIDTRESGPRSHGLENLILAHFPQQLPQKQPSPQPQQQQQHQRHSSSGESVDCPE

MPSFEGSTSDIAPGAASATRDGGGGDGAETAAMVQLQQPCGTLSASASAYRPFAVRRACR

VLAEAQSSTTPEQSRTQAFSESVCCPLSTVEVAHVTLPLTLISTPSTVHSTELVSMTSSH

IPSAPVTEENAVVHGSGLSTRLLPPLAPRPFSLSQPLEKEESLMTPVKVNPKTVSPYLTP

TPHSGASSTQSKEERDTSLTRLVVPPCSAADSTSTAEMNSSSLTSRALPYTLSSRDTSPK

LESREHSFNVTPMDRVGTESSGNAEALPHPWSPVRLPCVNAVAAPMPTTISSCTLVAPAF

SAPSALRRMKVAPPSSPTRSLAAYPPLTRANSSVPTGGEQTKGSRSEPLPEA*

>Lp_000048900.1 hypothetical protein

MSVAFDNGAEQLSSSASSSFISEEPMSDPRVNPSPMSTGTAHSSEPASAATPVANTHAGG

PGGPNDLNVNKVSGTRAGMVEKPVNGSPVTRGVSRFVDPEDENGYVGFTRRIN

>Lp_000049100.1 DNA polymerase theta (helicase domain only), putative

MRRTLVATKGFHAPRPSGNHPPAEATSSSPDIAARPPAHPVEQSPASTTTAVAAAAANSK

EAGPSLKLKLASTHPQQQQQRRPSSTPAKKAPRKSISSASTANTTSTTASPSKATATHGG

TVSSLLANRGALSGHFSANHMRRGSRRESLSSFSAPAAPLSACSGGTGGAAAAMDSSSTE

EATASPADVLSSKFTPAGDTEGAQGEVTRKRSRSPSVHALRAQAACRPSTASASPSRASL

AEVLSEEQVGLPSRAARRLSSSYLSATPHTARNVFYPPPAELALAPSPQSADAPHHVSHR

HFTEVQRVTTTTTTAAVGNSGEEDVPLAVLYAAYTLTHGQDTSSTSHGAARNDLHTPERL

RSRTPYSEATRMDNAAGEMVAQCGGIADPGVETPTPSSTAATNTTASSSQPGTGFLSALE

YQQREQHNAVHASIPGGFLDSPCSVVLRSLYATVSQQEEGSPPPTSAAAAGAAPPPHAAL

PLPTQEPQLSTQPEAPAPQTARRCSPASVFSASQDYPLLQGSPGVSPPLFPNAAVGAFFR

GVDAFPPTVMCAEPSAPEMRSGAAELSSSPSAIRAFRERATEEEEDDGVSASLHAPPSCA

DAASMHRSVGSGGDVSIRDLQAAVGGRVLPMPEEEPPELDKVLEREIAEDLRLSMDVAFT

PSGAGNGAPPLLSQSSLLYVEEEEEERNKSRRSGLLLEDAELEPPCAGSAAPSAAAAEDV

AAPKAVHTGEALELQPQPSTSIALSAAPPAAASSSPLFLHIDASTPIADSSSAMLHLDST

AITLSGSYQPPPPPPPQQQSPASWPLHEAPLQCIPAPLVRDRDEKISLAVASAAVALSSG

GGGGSRHRTVFSFSSRKSVPAAAAAAVDGATIGGVGGGAGGCGTETSMPSDPARPPTATT

PQLPAPGSCPDKEGESEQAIIIPPAFVTAAPQGDGEDKETPHLLAAAPALLGEPLPDSES

TKFGIDWQSAKLRAESTVPDNAPEAGRRNGAMFTGPDLALEAAPSAETPVAASASANPLR

TPPPLPSREASATAVNAQSPLRLFAVPSVSARRGRTCFTFHSAPSPAELDNSNSNSNSSV

MLPAVVEGTSGSSASGNSLPPVQRADPVDKSMTSTALEATSALMQPSSGVSVVQHAPDNR

KAASFFPATKPALEPTLLADDDDNTASDARKESTGATYAQNSAALVTPVSDAFVAPIPVR

IPLPVHPSSLPTPTMTTTAIAVTDSQRISSHHRVLHDKEEETQSAYIGVLAGHPSPPPSP

PSADHFYDLPRSVGDFYASRRGVAKLYDWQHELLTQPEVRRGRSFIYSLPTSGGKTLVAE

LSLLRCVLNRRQSCFFVLPFVSLAEEKTLALQPLANVFDFNVDGHYGSFGRFPLCGAPAI

YVCTIEKANSLLNYMLEENRTAEIGAVVVDELHMVGESRRGATLELFLSKLLMIDQARQE

LRRKAAAAAAQAQAERDGAQTYSAVKGVHVTAPAHGWPGNEEEEYSPGMEENTSYTTHKT

HGEEEGEEAAAARQRQAAAAVALESFADPGPLQIIGMSATVPNLRTIAEWLHAACFERDF

RPVPLHAYSVVGGLVLRDGQRNERNLSGDNVNQHLIELATEMPEASVLVFCASRQQCVDT

AKGIVNYIKAQAIAGQQLPSLPGNSATASSPAPFSVFGVVFPNVATKSAVSSVPSQASPA

MKSLLGDLEALSHYEASQLSEVVPYGVAFHHGGLLTEERELIEAAFRRKHVRILCSTSTL

AAGVNLPARRVIIKTPYVGRDFLTKARYLQMCGRAGRAGLDPYGESYLLLSRRDQSRGHA

LMHAPVEPSTSQILEDDQTLTRSLLECVGVGLVTDWASARRWCASLLSPHAVGPVDGEWK

KHVQAVMAKETVDTRRYIVTGKVEDSASASTDTATAVPGPQTATSPESSETSRSVPAPVP

AQPPAPLTSVPTRALESMVRAALDTLARCGLVAVTEVHSEDEEVNNDDVSAVQQSTSATE

HAVLQVCVTPFGSSSVRSCFTVEEALLLREELEELRHTGLILSDDLHLCYFLTPLREVGE

CNWELLLMIMARMSDSRQRIASLLGVDPYFINQQAMGLGGPLQATEEGRRRLFTAKRFYV

ALILADVLAEVPMTTVEQQYNVNRGQLQNLMRSASMFSSSITSFCHAMEWYSLEAVLSSF

VKRLGFGVKPDLLPLMEIRGIQPPRARALWNAGFKTLSLIAAADADDMVAKVKLMNPADS

KAAKFFTKRSALMVIREAHLTLQSQIKEKKGELQELTLRDSSAVL*

>Lp_000049200.1 hypothetical protein

MSVVSATSPTPAWSEFSHEICALLNCNQCVVLVGDADLVRQTTPLVDRACQHPSVTDAFT

KRTSGSGAASRCCAPADMPHVTFTTETNQQIAQKCMAVFGRIPFLAIVDMRDDDDEHSFL

FDLNSDVTTVANAAALTEEMVSFLTRVGRGTEPRCRQGAPPPLKDVFEPTHGAVLCEARA

AVTATFTRLLGKTPATSSGISVAPCGGLCVFWSDRCPCCPGVLMLVEAMVKLIRLVAARH

AEHPSGNVSFVFPFLAANIDDNEFTEEEWPVAQREQVVPALVAYPAPNYEPVLFTGERLP

LRLAQFICAHCLPSAELVACTPHIMEEVGRVAAQLSIDALMTVVDESSEAFIAAKAAHET

FVTSDTNTAAPPAAAPTTDSLEQLLTSLSEIRAAVLPFSLEAEVRRMSAEGGAAAAGSLS

DSVPADDDGGDETDADDDMAETKGSGSGGAGVAMAAAPGTASSTPPTTALPALSMTDVYK

RPREW*

>Lp_000049300.1 mitochondrial carrier protein-like protein

MLAHSKTSEYVQRRGASASLCTAAATTTTTTTTTTIAPSSAPASLIGSDGRKASPAAVPH

GSKASATGSRGTLATATSSSTSTADLYADTEVVAMASSSAALLTKSVLHPLDTLKCRVQL

LRTDILPPNANCSTWRGLMRTRLRQLRHQYAGQWAPRYLYGGLPVKLAFYVPYQALYLSS

YNYAQRALQDGWEREVGGGNGGRTHRNPSYLWRTVAAAVFAEIATFCLRVPMETMKMRVQ

STATTSSVQAVVQLWRQGLRSNLRLVVPHTIMHDIPYSVIQWVMYESLRPWTQQWGAKLP

SSSSAATADGTMTTTTFFSRYGAELARTFLSGGFSGLLASTLTVPLDNIRTRTVVATASD

PKLTVGRVVRVTYQREGLRGFVRGSGMRVLWVTTNMACFYPLFEGICYILQCRADAKKET

AVGGH*

>Lp_000049400.1 hypothetical protein, conserved

MRRGSVRSAVVAQQQAAASSAFPSMSPGTVTVAATAAITGLPLYTAQRHKSFSETIHNMT

IMRMGSSHKPQTYTSKDAGGGPQGGAGHDPVFANYIFKKITGPLRRKTSDQPEDFLNVDE

ASKWMDAKHAAKVLGIKEEDLPKLNREILEEKWRKAYKERTNAQQEEVLIATEVLLEYLD

SSVYMKKSRQYYRQFIDNARVEVDQELTAARRGRSQSAMWLFGVAMTGACFIVLFVAFSR

RYVTRKDVSTIGIKTSEYFLMTFLQPKNPEPKPDYNTRYFNTPTSMEIDQKSGRFANQFM

SDEEARHAAESAAYTERETAEMVKLFNDERERSARDKQNELARNSRVAVYLPEEVNEGKQ

RAASRSDQVTDFEKMSFRDFGNMLASNFGGGSRFQRLTEETSARSERLYSARERMGMSSP

KSTDS*

>Lp_000049500.1 hypothetical protein

MQPAKDAPLFTVQSVVQGLASDSALLLSLVTTVERTATAEQKAAFVHHFVALLLRCRDEA

VFAGSELLSELFILDLDDAMWRQRDAAVFLCRSSTIVSCLLDQLSRETFSSPEGNLLPHL

VAATRVLSAGDDASSSSIRTPMGVSPGQWSHHSEDGPLASTAQPDGLSVYDRFSTSELVA

LSTAEQQFFDLGRSPYLIHSFSSSVGATGMEGSRPPLQASFTSPSSQPPRGVSPYGVNGN

GPAGSAGWRDSFRYTRDTEASNENGSRPATRMAFTGVQIREAVDFFSETAAVSDPKSVHN

TELALNVVDVRAEAVLTLAFQIQDVVARHISDCPLVLRRAFWLWLRATSGRSGPPESAEI

SPELNVTTASAADAHTPPLNLSSSSSSPAAAKASSAPMASFEFVGMATMVMLHMLTPLLL

SIPSFVLPVEEDEDSEAYVTTTARFRFMAKVLQKAAYGVLFDERNDAGLTPLNRGMRELH

RRWVSCFSALADYRNASSMNWCCPLPSPTPGLAVSLSGLAEEQAVLQRYFQFLDGFLVPY

LQTTVLLRRFTEAIHLPQWLRCTYFGERFARGQIGGVCNAATPPSPQLDSKDVAAAASLS

SPSPLSSAAAAAAAAALQKSASEDEKLQYMGKAISHVVTGLSSATCASTTPAVSAFMANF

ISFLGTNRAKVTTVVLYEDLLRRYWAQFPQRGATASGNAASVERAEAGELLAFYIYLAVQ

HNVVSPEYQLVLIPSVSAALRAEGEPKYFSSSPFSTIDTLRWIWRVLEGVPLSYRRHCER

IWTATIASGERGLQ*

>Lp_000049600.1 hypothetical protein, conserved

MESESLDRAWDPAAPGAGALRATAIVQLYFRMTSHHPLTILVVDAMCLTFGADGVTVQEL

VDYLGVTPDRIRFGLEGIPAEMRCTAQQLEVEGVVTASSSSTGAEASADHNATSTSLSSN

QYDAAGGGGSARPGEKEVRYYLNYKRLLPLVYAHVTRLLLDTCVTDVPLCSYVEAVKAAQ

LAPYSRPTDDNAHAFFATTAAPGNALPAASPATGGKPGGPVEATAAPVATHTPAPASSSS

AILQLDSTGEHVDVSDAMKRRSAIRGVYCLGCLCFFLPEEFAPTLSRCPRCGKDSLRLCI

QSIQRQLNARIADQRTMVKLLPTVSQVWKRAMGPVLASGARGQQAKEGKASLAVGSGEGG

AVSSPTPPPLKSGSMLASSAQQSLSCALASDPFLFQQALAFLFLYATRFASVNDAASVVD

AQQILTEPEYRERLRGKASLADQFRSRHRHATSVHVRLVSQRDIDIARREDSHQKLLKRA

MLPPWLRHTSALEALGGIHVYTRTAAAEAEATTAKVVSLTEGGEGEPWKSEATVMNEHAK

AQRQHSGAVWKPGKIAAPTAGEKRKRPAEAESEAELTRMAGFIAAHYYEDDYDEVALPLS

RARQRA*

>Lp_000049700.1 ribosomal protein L1a, putative

MSVRPSVSVYSASSDSVVGTCPLPTVFTAPIRSDIVQFVHTNMAKNSRQAYAVNRLSGMN

HSAHSWGTGRAVARIPRISGGGTSTSGAGAFGNMCRGGRMFAPTKIFRRWHRKINLHQKR

FAVVSALAASSVPALVMSRGHKIENIPEVPLVVEDSIQGYEKTKEAVAFLKAIAAIDDVN

RVNDSREIRAGRGKMRNRRYVARRGPMLVMPDNKGTRAFRNIFGLDLANVNALNLLHLAP

GGHARRWGIWLSL

>Lp_000049800.1 Ribosomal protein L4/L1 family/60S ribosomal protein L4 C-terminal domain containing protein, putative

MRTRRYVARRGPMLVMPDNKGTRAFRNIFGLDLANVNALNLLHLAPGGHVGRFVIWTKAA

FEQLDKVFGTFTEASAVKKGFTLPVPMITNTDVTRIMQSEEVRRVLKPKKLQPKKASRYQ

KPTNGIKNRRLRLRLNPYVKRETAAAKGLRNKANRDARRQAKATRVAKAKKAATKSAKK*

>Lp_000049900.1 coproporphyrinogen III oxidase

MPLPVAEVKEFLLNLQDRICHALEEADGQAKFIEEKWTREGGGGGRTRVIANGAVIEKGG

VNFSHVYGKGLPPSASEQRPDMAGCDFQAMGVSGCIHPLNPYVPSSRFDVRLFVAEKEGK

EPVWWFGGGFDLTPYYAVEEDCREFHQVAHKLCEPFGPDVYTRFKKWCDEYFFIPYRNEA

RGIGGIFFDDLNEWPFEQCFKFIQAVGQGYIDAYVPIVNRRKSTPYTEQQVEFQEYRRGR

YAEFNLVIDRGTKFGLQSNGRTESILMSLPPRARWGYNWHPEPGTPEARLTEYFLQNRQW

V*

>Lp_000050200.1 60S ribosomal protein L11 (L5, L16)

MVAENKAANPMREIVVKKLCINICVGESGDRLTRASKVLEQLCEQTPVLSRARLTVRTFS

IRRNEKIAVHCTVRGKKAEELLEKGLKVKEFELKSYNFSNTGSFGFGISEHIDLGIKYDP

STGIYGMDFYVVLGRRGERVAHRKRKCSRVGHSHHVTKEEAMKWFEKVHDGIIFQAKKKK

TMIRRRRR*

>Lp_000050300.1 SET domain containing protein, putative

MWRQICARLNVMLAPAVEQQYIHGKGCAGLVCKASDVIEEGEVLAVVPYLACTSPIMALA

SPWGTQLGAAVGSYALEDGGVRVEYSREGALTTAFTALAMQPRSPLEHYLRSIPLGDVDA

ASMEAVLGSALFAQLKVIDALNAAVVERMHGDLRAHGVAVSLADLQRAHRLCASRCLDVP

GSEEFFGGPALVPVADLINHDSRPPNVAVYAESTQRLAPLLRRHSRMNVMDTLYKSYAFC

VVVRATEQVESGTELTYQYVDATADPSLYADKLYWASRFGFVPPDLPAK*

>Lp_000050400.1 hypothetical protein, conserved

MESSAKGGDETVKVWRPVTTPWKLASPHPTSTDVSPRTDPARQSASAAPPLWIKPPVLTS

SSTAASADSADTAPNMEQRWDEFQRLAQASEAAFRGMAEAAGRQHAEVSAQQKELAAYQE

SQNAMSAQQRQQIDQLTRECAALRQSDASAKEVLAHQADRLVALEAELQGTRVQLAQASL

AHQQSAQECSQLQQSCKAHEIHTKELQASLRFADEVVRSMRRTVAACEKEKNEALQDQHG

RFEGYRIELTAFYDRRAEELRDEFTAKVAAMQAEMLAAADAREEQLKMSWQDTATKLRRE

YEEVTRLAQQRKAAMDTEYRERREQLDRDKEAARVQLRQEAEATELRYRAREEALLDDIA

HRERELREREAAIRAAQAQHEQEMQRRIQAREAELRSAHDAALQRMSEQAAAEREKLSEG

FMERLQQLSNAHMQQERELERMHREKEREMAQRYRLSGIDGSDRGDVRERRPAELAAGGS

GTDAAREALLKRFESVEQRQRERSDKLRSVLSSQEEGDHTNGL*

>Lp_000050500.1 transcription factor-like protein

MATVGDEAERSSQLAEELKQELYECSTCLEKVKLQQPIWSCIECFQIYHFKCIHLWGQTG

RESEVVSCPHCRHTQPKPLVDHCFCGKVPKPKYDPMITPHSCGQTCERERPFCAHKCPMQ

CHPGPCPRCQLLVGPVSCPCGSTTYTYPCGQPDPETTCDHVCGKPLACGLHTCTLQCHRG

PCMPCVEHVDLTCYCGKSTRHFPCTRETGFVCGEVCGKPLSCGNHTCTLLCHEGPCPRCP

TDPACVHTCPCGAMKLTILRTSCLDPIPTCGQPCHKMLKCGQHRCQLTCHVGDCPPCAVR

VETSCRCRKVRKRLPCAEAQTFTCVYECGTKLSCGRHKCKVVCCADRGKPQAESHLCFQV

CGKQLPCGHTCAELCHASQQCPPCAHIVTTPLRCRCGAEVLRPPQPCGTQPPVCRRACQI

PRSCGHPVGHQCHYGPCPPCQTPVERICPRHHVAVTVPCGATEVTCEEACEALLPCGHRC

NRICHTDPCVEDAHPCRQPCDRPHEDCGHRCTKLCHSTSPCPPCSVLVACTCPCGRISEK

VPCHKIKGRQESQGSKYVATVPCNDDCFFSRRLEALASLSKTKNEKFLYSLFLWDTARRD

LHAVQKAERQLSKFVEGSEQVASLPPTNSTSRALVHTLAKYFHVLSEGVDKEPQRSCLLT

KTGSTSIPPVLLSNAVLDPQMDPLEFLAQRTKPSLREKLCLVVTGAHVSEILLSTLLSDL

AGRYVIAPPEVSKDGQLSFLVAFTTQKRAEEAVKLLEAANTQHTLCISRATA*

>Lp_000050600.1 hypothetical protein, conserved

MSTLTVVCSARWSHHEVTRALRQHRISVEVSDNLSYCDFVCGASSVLYYELRSAEAHHDQ

ALIISRLGEARQRCGTQPVILMVWMSNPEPSLEVLSWLNLECGVAQQCGLLLVWSVDDVV

QFLASLVASAVTSLEFNAAGRHDVGDAPLPILIDALTQTPQIVTRNDIVRIANRKTCMAD

VLMSEAGDWEGIAGLGHKKVLRLQHLFRTLFLSSQQTVESVIGSTAPAKTANTAPVAAVN

AMLNDDEKSAPSVPTVPPDANSDATTLGKERMMRALQRRRDEEDAEDN*

>Lp_000050700.1 heat shock protein DNAJ, putative

MYGTDMDEMLNAVFGAGGMPGMEGMGVRGGFGGRGQRARRGHDVAHALPVTLEDLYNGKT

VQLERKRTVLCPDCKGSGSKKSVPRGGNMCPLCRGSGARVVVHQMGMMVQQMQTMCDACR

GTGEIIDPRNRCTRCSGSKTIEVDAPVSVVVEKGMSHKQQIPFARMADEEPGVDRAGDFV

VVLQQVKHDVFTRDDCDLHMQHHLSLQEALCGFQFKFTHLDGRELIVRQPRGQITKPGDV

KAVVGEGMPVQKQPNKFGDLIIEFLVDYPERIEESQLQLLRQALPPPKSVDATAKNEEEE

GEVCYVTREDLSILEEEVKKDEEAEEENEGPSAGCTTQ*

>Lp_000050800.1 RNA capping enzyme, nuclear

MQLQSLELLSKAHHDESHRRYCCRATDAWYRCLHGAEAFCAGTSMQDAEAAFQRMLTAPT

ASVEDAGSSADALQCVLSCANVTGSTNTAEFPGPMCSPLCKKDIPMLRQRAYTVTEKSDG

IRVVVVALWVADFPQWTCEGDTETNAASLTHLSSVFVLESTRRNLQQQQQQQPSDSPSSS

SSSSSLAADAVLQGRRVTLHTTAVTAATSTVSVEEGETYTLTTVEGATALLRRRTSGRHF

AYAVDRTMDAAYLLLDDHTSAAYRSFVLDGELMCVRGSVAARQASDGGAAAAASHVLLLG

AFDLFCFTPAGGDAAAKEVKLVEATMMERYEALKALIATCAVPPAGVDGHVAWYAKTMWR

VADLGACLAKLHYSAEHHCFLFDGPYGPTENDGLIFTPEVFPISVGSSSVQLKWKWRHLL

SIDWLLTASDKQPDVYTVSLFFVKKNYGHRNDVAGHWRLRKPMYINNPHHFNIPVDGAVV

AECAYDAALQQWYIQRLRPDKQGANSIVTAISVYESLVENISLPRLLNLLRGQADAAEAA

RDAAAEAAAVALEDALRADVYAEAPSQGSVTAAPPSFDVAQAEKCLTATMTLRAIRESRG

NTELYLNAYTNNTNKAVKFPLPFPLRKIRDCVGLGYDPDTAATSAPSLEEALYIQLGNAG

GCYAWSDFVVHAFYNGETGYWELIHLSPHGNNKDALFDNVIEHLEWLLRHAGHKAEWASL

LQRRRDKPLVVAGAPTSEATAQTSRHYGAVAKELANAARSDLRRFNNWVKSLLISTAAAA

VRKTLKDPAKLHALDLCCGRGGDLLKWQHLRPAFVLMTDASVECVAEAAARYSTSEGQSM

KASNGKQKGFPAFFAVHDAFDAASGLRGNLVKRGPFSLTSCQFSMHYGCRSEDGMRCFVR

AVADSLTVRGRFIGTTVSDAELLARAKAQGPRYGNHVYSVHFHDDAFAQVQAADFEPARL

SYGVAYSATVERSVQGMQEYVVPWGSFVQLCAEYHLRLVEEDNFLHFYEQQKETADGKAL

LAEMHLKRHRDDATAGIPLSPEEVEAVQLYRLFVFEKTEKEA*

>Lp_000050900.1 tubulin folding cofactor D, putative

MHGEATPSKPAEGTAASTGPQDNNAVVKHAQVAAEEDGEMVTMVTNNNNNVNEDGEEAED

NDVCEPSFFEEEAVCTALLARCSELLPPTNEHLLQSVGADPLGVAALLEVRPPAIIDRYQ

DSPHLLHPHLEKLMQPLVELLLRYLPNAAEVWAKEEEKATSAATADPSSASPAAPSAQDA

PTLVVTSSLGQDLSMFDPDAPKTPLHVVCKALYSIIKTAGEKCCTSHFPNSVSHYEDVFY

TLQLWVADPTRQREWEVRYCLLLWLSNLVLVPFSLALVDTHQTGCSSGAAAMRLSLSDAT

LVTASRFLADTSKCREAAALLVARLLTRPDSARHRQLFFDFATFILKSSVRTDAAAQQGK

RVEQSTQTAALQRVPHEAPWAFLVEGTQTAFRSLLSQPFLLPGVLLAIAKTMKLGRREEL

VPFAATLLASVSTVYEQHPTDSLLCKTAVKVGQRLALAMLKKKRAGWRYHRHIASLSANL

ASATRSDEAAAPAKNSENGNSNRAVEEEEGSGHDEGGDDVIDGDAESLETGIGLLLQAIG

HKDTVVRWSAAKGVGRVCERLPTAFAEEVMEAVMEVFANDYSDAHWHGGLLTIAELCRRS

LVDTALLARVVPIVAQGLAYDLSKGTYSVGAHVRDAACYTCWSIARAYDAADLTEHVRQL

SVSLIVTALFDREVNVRRAAAAAFQECVGRLGNFEHGIELVTTVDFFSLATLRHAYTVVA

PAIAPYATYRDGMLQELVGVKLLHWDKTIRQMAAISLGLVAMQESAETVVNEILPELLRR

VDDTTMATRHGAILAIAELIRRLPPSSTWTPTHVKQFVHVLTSLESSRGFRLRGGEYVRQ

ACCAMLQAMAEQRLTLPDTVEVTRVNGRTAKVRTHEVIYSFLRDTWVNILEWVQLAATET

FAVVGEAYWTTFQPVFHGKVLTELLEGCGAGQPPTRRRGFLSAVGGLPATLVNAAWVPPP

PSAETDGGSAEAEKITAASPSTTRAFEALIPIVQAASLLSAADLANPELADAETRRNAVQ

ALTRIVTLVDAAPAATPAARSLSLSPVVTPSWYTGVVEGTMLRALRDYATDQRGDVGSFV

RLAVLAGLPAVVRYGLQPRVSGPLLCTPATGLRVLQGVVRCLLEKLDRVRAAAGSVLVTL

LLLLRERCGGLLGGKTQLGHTRSGGEEDQEDDTAVMAVEQAEVALFAEHIARLTGEGAAT

TVAAVEAAAEVIDWHNTEQVMSQIGPFLLIRCPVSLAHAALEGLIVAAGDLSEHVGRPAT

AALLRAFRYDGSSSSNVPSPDVAARRLSACLLAVLRTHEHEERMLKPASRVLDLLINEGV

FAVEQHAAVLAMLQKELKHFALNIVVLLAMVPLLANMCRSPDVAVQRGAWTLALTMIASR

YPKVRAKVATDFYTSLLVLTSGSAAGAAPSAGHPLEGCRQAMEHLMKVAWDGNDATRIRG

ARNELYGMLGIEPPGAKAKTGEASLEAPRGTATRPARELGGAVAATYKSLVQETGY*

>Lp_000051000.1 hypothetical protein, conserved

MEDLFDQVVKGSPMQQLNLKGTDTPEYTILCVQAQAPAQRVSLLQHRLQTSTESSAGQNA

SLLLKACVSTQLSPDDVEEMVENLAGIAKQHSNWPQFVRWVCAIIHYYCTDDFTLSLFSE

FGVPKVAVEALRRYLSDRRIVLAACTLLAHFNLYDIGDGITQLANVLQTHCGDATIVRVA

TRALAEFTSYYKEIPDRFLQASQEFLDAKGVAALEAVLHEHISDEEITTYAARITANVTS

SGASNMLDADSPVMMYLADALARYQQSEMLCGHVLRVFSNLPRSQFIDWDCVSSLFNNTK

SELVVLECIHFLCGVAINVKEMKMRIYSTGCVPRVLEMMRTYQSNAAIQEEVCSLLSYLS

FDSETITASITESGGLLLVLNAMRKFPGNEDLLMSACAALSGLTFNNQAGQQVIVDNGGV

ALILDAMRRGKKARLQENGCLAIGTMCWNSDLKADVVRLGGVQVIMKALEEHYTSSGLVK

NACRALAQVAFNCERYRDEMSAKGVIPLIIRGMEQHPNYDRAQMHGCVALSYLSWTNEDN

AAQITANHGYKVVVDAMRNHPNNHEVQEHACRALANISNVSLYDSAAALEQIVAAMRRHE

GVSEVQEEACRAIVTLSLVSPANKDRLFQLNGADAVIAAMKRFPHIQLVQQEACNALAHL

AYEHADLNRAVTRLGGVSLLLTAMRTHKSSPKVQLNACGGLSALAFDNTVAQQQIFELGG

VQCVIHAMDNFERLRMLELGCSVLGTLAWNTEIKERVAVDAIPEILKAMRTHSSNALLQK

STCRAISQFAFNSENNRQLLADAGAIPLIVNAMRTHLSTEKLIVHALKALTYLCWENTQV

AETIINEHVEEVLQRIVEHYEQTHRVFNEAVHLSKILFRKTTGSPSPSLRIVSPPIVSPM

PIQQTPGLRYDIPAPPSPPPEDDPEDHFYSAPVDGPVEGPYTRDAPAADEYRGGTRGGRN

GRGRGAVRRRGGPPPRRGGGGSVERGGGGRRGNGMDHN

>Lp_000051100.1 4-methyl-5(beta-hydroxyethyl)-thiazole monophosphate synthesis protein, putative

MRVLVVAADYSEDIELVCITDVLARAAISVTLASATASKHIVLSRGIRVECDALITEVAA

GDFDAVLLPGGMPGAETLGKNETLKALMHEMRSQNKLYGAICAAPAMALGPMGLLEGVET

VAGFPGFEEKIPAGVKYSESAVVRSGNCLTSRGPGTAIFFALAAVSILKSPELAEKLAGM

LLVDKMSEMDAVRALK*

>Lp_000051300.1 hypothetical protein, conserved

MLRRCASIATPSSHASIPFTVASETQKRFLKIAKSTFGFYLARRGQRKFPFHRRPHNKNT

YAMNINAPYFWSFMTAKSQSFFLPEENYITGDWTGKFFVSKRQVYTLQHATSGGKVRVKS

FPSVFELNSPSRWNVGKEMRTLTKPRMDLIDDQMLTKKQRLDYVKAGFLPK*

>Lp_000051500.1 hypothetical protein, conserved

MRRLFIPSLRPLTRCIVVGPSSALPKASSTPEFGRRRRRLQRRAEKQALEVQNDDEAKNA

CFERYGFVQKPLFMASWQEFLKELQRVELGWSLAPSAGGTLRLKVFDHSEPGDGLLCELS

GSTSRSAPMAEFFEVCGTLCQGSTSAGEVQFMDGDKHSVLQIASKKRFPSSELKEEPPII

PFTSQFNCRGTSVSLSVLDKQTNVTTPLFSDISIQTLNYAFMTALPIFLKRSDIGVRNAD

FVTKDQMRHFRFAWCFLRRESWMTPVDMTELDCLLPP*

>Lp_000051600.1 calpain-like cysteine peptidase, putative

MSIQEVADSNKLFSDAVFDRDNAHISEDWKRIKDIYPSGVNQPLLPETFSREQFGQGNHY

ECFMLSALAALVRFPDVIRNCFVTKKVRQDGRYTFQFFRGEEWVKVEIDDSIALEDDDVL

YMQSPTEHWWPLLLEKAYAKFYTAYDHLEGCTLQETFHDLTGNPVLNIPMDAKLAKAAGA

EVTEGFYWLDLAQRIQSGQFVASVLTKDIELENMGLQREQQYGVLEIFSLTGTSSINDIV

IHLHNPFEDEEYLYKGPLNSRDTTWDARQRAKHDVDDERSIFLPLNTFLKIMNSVQLCYM

TPVEPDATYFEDEWKGETAGGNPTFVTWRKNPAFVVRNDGSTQVKIVVMIKQEDQRRTAD

VEVGASYCQCGLVVVRPTYPNPIPTLWLTSNNHKPIHKSLFLN

>Lp_000051700.1 hypothetical protein, conserved

MRSRRQGVSDAVSVLGMDPWKDDRLRDFVAINTIQRLHAYHLPPGSPMPPVDSVNVDRAL

NLLLLPRAEPDYRPCAGERPPTRAEMRVRRRAREREQRRGRDGDDEESGATEGSQSSSSS

STASSDGDEGDDTDEEKEKAPLHDGTDSAGLAGDSSDQTPARQPLPDHPSHSPEASADAA

MSQSSSSTVCAIDRARALFFGAASFHSSTSPRRLLNSPHVPLTPSVTSTSPHQESLPPAP

SATRAATTTDEGVAEGDGPRARLSTASRSLNGELDAYIEANSTPLRPLAPPPPLQPTPPL

NIMESTATIHRGSDGEENEGEEEAEGVAVDLGERSPNREGGIASDVGKASEANTLDRPLD

ATPATAKPTAESGDAGGRLNRNEENASDGDEGSMHRSCDACDAIIQYYQSMLTRGVVLDR

YNHKDLAFAWWVLGRNMDTAALQSRGPMRREALLITIRNLYYSLLSAEEAKKTKEAAAAK

AAGSTGASDRAAGKSARGEPEDTAQTKTRAQVGAETASSVRQNEAKEGDESAAASAPATR

PRGRGRRLTASSFSPAVPQDVPPSARKRPRSGQDVRGVESDGEADTQTEHGEASHSDSAA

SSHLHPPPPPQQQHPSHQPKLRQLGTLTEGAHISTIATRAQAKVPPLPLSRGSLTTEDTA

LTTPPHQGSLAPVDTTVVPGAALEPLAAALRGAETSQDGPPTADDSASEASASLVSSATA

SRAGSIAPSSGRRRRCAEDRRRALEETWTSTRTNRRIASAKAVEQLAQVVPTAEAGSRVR

RPTATASLAVADERDSKTGAAVSKGKAAPTKSSAGKPEAATAHVKAVGEDAVAPTPKPKS

KATSKRSRSPISDSSDGSDEEDVRVKGADVADRKSAEECREEKQKKPRGKARQPATKRTT

GSTTKAEEDNGASADAASKPEEAEKADTAACTHKKEKPPQEPPQREEKEPPKQPEKKEEH

RGRPRGSFKLPRIMLDGVPVAGTASNLRKAGVLLPPTASRGKDKKRQETTAIGALLSALA

VSASSNADSKQRRLAPTAAAPVSRELPLGLTTHERAVRARIQQRMGQLLEPPRSPSSPAA

IPASSPSDGAPQASAELPMDVYSSYTFLLSSFAAHGQLGGGHRNHNNNNRGGSTATTAPA

LWYGPAAPSAAHAKSAEKEGETSAAPLARLRGRRKKPEGGVDPALLGPPMRHDQAVLESQ

VNARLAQEAAEASPRSSCPPPPSRAKRESAESVSWAEGSTAAVKREGGADGDHDDDDDDD

GADDGVTTRVASRSDDAASSSAASVPLLYPFSSSVSDVTQPTTFSRGPASPSPPSSSGFP

TTNTTTSAVKAEPAPQPPADFADLTYAQQCLLVWTAGDLLEQHIQRRQTAEARRERLLAR

YSRMATRRMAARVAAVVESGLGEEETDDASAQRLVQPLTNDGATAGAGQRKATEDGHTHS

YGSRTESDSSGDDDSNSGSSGEEEREAEPHTTRQHSRSHRTASAAGMAEDKSGESDGDDA

SSVSS

>Lp_000051800.1 hypothetical protein, conserved

MTAFNAVSRHAALATVVLSALAAVSANAAKSEYKPKFTWWAILIMMIGIVVVISVALVIY

FCVFRTKQAEEKKLDDVSDAHSEGAAVPPSNNAERESAHRQE*

>Lp_000051900.1 60S ribosomal protein L28, putative

MVHSADLQWVLVRQNSRFLQKRGGIRLSNDPFNNNGNWTKRQSGFLNEKAAVVKPAKGGA

ICVTTKDGSSNNKPKQMYKKSVQAAGVKASAVSKVVAAVRPDLADLTFRRARRMARIHSR

TAKVAAARKARSEKIKFSRKAVRPKH*

>Lp_000052000.1 hypothetical protein, conserved

MVQLVDSYANALKTFDPSAWLLFNALRDKVVQSAEQLLQSFEQKALRPPLGVADDFFPAS

ENEEASASAFFHNSRSPAQPREAASQLRGGEIGRDDGLLSQVDGGAAQLRAASNADGLTL

RERLERELALLTQYHAHVTAAGATAPTAAASTTPASGSTASVFCQLSPPSRTVLAFTAMW

MTTKFWGSVSESSTLSGLVACFIQQQQQEQQQQEEQQQEEQQPWLSNSRSPMVSSGQDNR

EGASHTPRSELGGVKAATPTRGGARLACPLSLDNSGVVTVRSASTPPRSPVEALSSSSAL

HAEKMGAPHENMTLFPATAATPMTTRTSVTMGCGKVESSFAYTLKSKGEEEAKRAVALPS

LPDEGGQLMAALNIRDYDDVPGYAQLLPPQPAMWFDGVSDLVDEIARDVEDAEIMLLRCS

EYSIPV*

>Lp_000052100.1 hypothetical protein

MYKKSVQAAGVKASAVSKVVAAVRPDLADLTFRRARRMARIHSRTAKVAAARKARSEKIK

FSRKAVRPKH*

>Lp_000052200.1 hypothetical protein

MSATRRQADEMQAQNLRLKEEQARKERELLEEMAKKEAAFSMVRRRKDAEIASGREKLES

TVAQLEKEQRDREAALDALQAHQEKLQAALASTERTAAERDALQQQLAQLQEERVRLSQV

LSDQEKLNRDLQRIQEECDETELQRDAAVCALEEMEARYHASVFHLLTLLEFATAWEEEL

HERALAERDAAAAADLDEATAAAEKTQNELQQTRGGRA*

>Lp_000052300.1 kinesin K39, putative

MVRPSATKRVEQRVRVSVRVRPLNPREQKAAEGSLITVTANQQTSVVSVTPVSSIGNDAT

EDGVVGNRRSTQDFQFDHVFWSVDKPDASGGSPATQADVFETIGLPLVQHAFDGFNSCLF

AYGQTGSGKTYTMMGADVNALGGEGSGVTPRICLEIFARKASVEAEGHSRWSVELGYVEV

YNERVSDLLGRRRKGAKAADEVYVEVREHPSRGVFLEGQRLVEVKSLDDVVKLIELGNGV

RHTAATKMNDRSSRSHAIIMLLLREERTMTTKAGETIKTAGKNSRMNLVDLAGSERVAQS

QVEGQQFKEATHINLSLTTLGRVIDALADMAKRGGRAPVHGCAVPRREADVHPEGLAGRE

LEDVHAGRGEPERDELRGDAEHAAVRVACARDRDCGAGERGPARAADPRAGGADGEHARE

PGRWGPCARGGAGGEAGAAGVGGAEACGGPAGAGEGA*

>Lp_000052400.1 mitochondrial RNA binding complex 1 subunit, putative

MSSYFRGGGGGSRRGRSAGGYRGGSGGRGGAFSHGGGRYANPDGAVNDMADSASVHSDMG

SDCDRGAHDVLGEHLSRPPPGLSCSALIAFLQDVEGCNYSQLKNLTGKTFHLTSNTMPDP

SAVTVRFLRIQPDPFAPGSQLQVTVPAPFSTRALLRSGCSTPPPHPSLSATPEKPSDSST

AAVSAEDASWRRVAAEDYLLRCLHRELAHQQHSSFAIQLLPMSQHVLPRSTVQVVESESG

DASELQDMPDGYIHVFLRVKLPGHARRIDGRGIHRILFSELLPLFQHAILRCRHAALWEH

VTSVSDQIWLRQQLRSAGLVAFIANGAVLPRVAGDTDQPLADTTVVAFTAPPSLLQTFHL

PFSQRVITGAGLPHGLTLIAGGGFHGKSTLLRALEVGVYNHVPDDGRAFVVTDPTAVKIR

AEDRRSVQGVDISPFIRNLPHRKDTTFFSTSDASGSTSQAANIMEALELGSTALLLDEDT

SATNFMFRDALMEQLVPPAEEPIISFVRRVRDLVQRHQVSVVMVVGGSGQYFPMADTVLV

MHAYHVRDATAQAREIVRQNCTADDGNSHQNSGINAGGLVEGAAGGPLTQATSAFPLPPQ

RHFNWDLTFASLVNSGRRGQGGRVKIGAVGTERVRVGHEEIELSLVEQLVEEGQLNAIAQ

CLAMLYDEGSASAEKLQRGPLLSPPHPPPLWTPSAASIANASHSAPARLSPVSDYCQLVR

NCESRLRLARLELQTPSCYLATGFTSLPRVFELGAALNRLRTLATLHK*

>Lp_000052500.1 hypothetical protein, conserved

MSKEAANPFERADVFHLFDGDSSVASSSPSAAAQGNTMLTEVQYKALVQMMASSLGLTAD

DAERLFPSYRVEATQSTDAFSAREPPLCAHLVREGAEVYWSQPSPARLIPDVRCCDARDF

DKREPFPDAGHGPHVHCLYCFHTAIENASPAGRGVPPPVVEDFRDAATLQAHQQTHHDRY

VAYLKMLEDCSDGSIDAEADALSDERKAACEVAAEQESRHVCYMAAIGTSACPVVVFYCA

ECDCFAPLVRFRPSDGSTEDGSATAVVSTEAACEQVDACAWTSTEEWNVILSRLLLSCHV

LHMIDSSRFAGGAKGARDAAGSSAERTRPRHIVYLFKPILFHEGVEEKLVEEFEDGAECT

YTAVVLPHASVAGEPPELLLLHDKEIGQDAEDRSGTDGADNGSPQTYAPVVVGFAMEWAS

EALLQSKLNLILRQHELGCLSSGVAVPPFHVALRWVYIEDTEEWVEAHVPYHRTATTQAA

LPCAYADAADEGIRVAATYKIDAPLPTTEENKAPAASSLLPPSPKIVPSSSNVEVDGSEE

DPLAPDGCPYSMESRTIDFFVRMTALAKAMHELATWRLSIDENKEGLP*

>Lp_000052800.1 phosphatidic acid phosphatase, putative

MGECILDKILRVIVFFRLEDYLLCLICGLVAFGVSKARPHCRPFSWTDSTIGFPYSGKGT

FPSWTLPLIAVLPGVAYVIGEAIRHVWWVPRLQARRGLGEKVEDNGHRQHSQRSRSWKHP

QEQRNSIEVVATEEGEEQQLAKQPQASVATVSEPDRTVPCSSAGVASSPILDTAPGLTNR

SSASPRPQSLGQGDHSIVPYGLRGAEASNGDNTNRDMSSGSGGSGPQHGVSSPRQRSGEA

STRRGVLLRAPWQRFLAHAHMWVLTQAFAVTFAMLVVNSVKVYAGRLRPDFLARLRREGF

TESSTEVDWCAAGKGGRVSFPSGHSAISFAAFVPFSFYVLHTLHAFSRGGVSLWRIVVGL

FPLILPITVAVSRTRDYRHDFDDVVAGSLIGIASALIAVGANLMVNGKTGQLMPRLPA*

>Lp_000052900.1 PAP2 superfamily, putative

MSCRFFIRHFFLWRVPDYLMIIVMAIVAAVVGAKVRPHCRDFEWSDPSINHPHAGKEAFP

MYSVVIAVIFIALVYFAGELLTKWNRPAGKMNMCLHVNGWIVTHAYSILLAFLFVNVSKL

YAGRLRPDFIARLAKEGITEANFSTFTHHQICHAARNGRLSFPSGHSGTSFAGYVPPCLY

LMGLMRTLNGGKVWLATIALLPLILPVAVAISRTVDYRHNFDDVLCGSICGASCGVFAVL

ISFRVSMRGEWTLRDHPDDVAETRAWLHRMLIDGPERPMLTVNESDSNSSRWSEGDSQGE

VEQEVTVSPAVENCLTTHPHHQHHSGETKRHDGKGSRPSNSEKVKGDLSVPSRCVASLPG

DDDVSPAVFQERTSNLSPSNWNMDALSRAY*

>Lp_000053000.1 hypothetical protein, conserved

MLRRAFILRSGHGDLFPTSKAAAHLNPTWVMDRQVPHAPGKGHCYAEFCTPATHSKFQVN

SPEVHEVRAISTRSPFKKELHHCVTVRPSEASIEATLVGVNSELDHHHKKTNDTPLARAN

IAAKLAAERHAKTRDFMAHKRTPLARSAKYANTIDYPSSQFFIEERVNRLRKIENTNQNG

YVREYMPWEPKPAPPPPPPPKK*

>Lp_000053200.1 hypothetical protein, conserved

MTSAFTRGERAWSDAATTSHPTNDASSSSFLFPSRQPQRLSRDFSVAASPSSPSLDEIRA

RLHDVMRSIDDTLVEAEAAPSMVQPFSRTAITTGAAAANSAFVPSTPCFVGGRVSWADGT

LGRVDQRPPVRPTAPPASSSSTSSSCSALPLSSAAAAASDGEVDSAATGATAGTGLSATY

KAMLDYWHGGAASSLLPPPSTVPSLSARLAVLRAAEAQQRQVLQRRADGASTTFPEPAEA

GETEESLGLHDTRFARRDRRSPLRYTEESRPAMRGFRDPHSAAAAAAFSSPPPVEGAAPL

PNSPAPHTCDTAVADTSVAHRRPPPSPQTSRPINPQSYQFYQSKNNNNNPSKHGDVFFSG

LPSTGASGINVSHSSEVLVHSPNTSRENSPGSVERRVRREARLEETSHIVTLALERQAAA

EAAWRAGQSQLRTPPAQPGGKSSASEARMGHPTAFVSEHYTSRQERADGGRGAGDGWTIT

TAPLDALQARLTLSTSAAALVTAVNTVDGREGNGELPMREELAVRARLAARRNGRESATS

LAGVSDSPPKLLPDSYARLSEARDRDASQHLQEEVAHQTKVQVDARARVARARVNVPASE

TCISQRSAELQAIRAHAQQLEKLRRQMQRETAATAPPLTPVQPSANTGRATQQAAVGAMS

GGGGDGGGMCAEAASTASSSTLNIPEVSPSPPRAPSRGVVRLPAPPQTAASSCKPFLSQS

TRSPAVEQGVTPADSTNMHNRNISSRMTALSTAAALKASSVEVDATDDNDDGVDGRDAAA

STSVSATSLDDARAPTPEQWVSPKPMRVASSHKLSDTALSSPAHLQNGQGPLPHEGASRR

RGAREPAAAAGGAVAASADVSSSQWKKRGALVDGDGDSGSDATQAEAASQRDWASSTQLP

PPPARPARPPPSPEPKKPHTTVGTQTRSDVGVTQHHHHCHIENRDALSKPAEGLMAAPLT

PRRASQPVVDAERDIISSSPSAVRPSHPGDHDDNSSVLTANGDFESRSGVGSLTPERLAA

IQSTRLHVSPTRGTAWQNGCASAFSPQPVSSSSLASSSCPSSSSLNDLLALSPFAATLSS

RHKRRSTTALRNSQTQTLPSVSAPSSDSPTRGPSLHDAPRSAAPSPRTSPLYAGGDGGRG

GGAAAAAAPLLVHERAVQRAVSRRTALRAQAMLCEHGVSVEVQLAGRRLPAVVKLSKDKR

ELLFYLERVTEAPAAVSPPGAPRARSLVTPPPPSSTASVVSEPPSRSPRAFPLHPQQHPQ

PQHPTPADPNQRGFLYSVAPSVQLREAGSRGKVQPAETWVRRKVMASPPPPSGGARTSEV

HLVPASQAAPPHPSPARFRAPPSPTLHPMLVQQAQPAAPVSSRVAAPSIVGASRASRSRL

PASLSRSSSVSPPRVMHVRELHHFPCSYARVYVPYGVMGYEEDCGFGGPGTWEDVQGILC

GPAAYEVLRRYGCPLFASMRGRDYVPYRVYVIIPEFRRVDIPQDAVLLVLDFKQRVDWVL

FLLAMQLYLAKDDGDVDDEKRCDGPTSTRTTGNTEAAPTASRHARPPVLSYGRALWMLAV

QRLQRARALRGLNPFESHVSARQFERPTDESPSRQRRESLLRGEVDTEDESRKNRAEGQA

ARPRRVPAASAVSATSALSSRTSSPPQYGVQGAPTSRLASPGTGVSAAAAARPLQFVAPS

SLCPPPATAPPNAVLRSPRRGRVMDPRLAQRRQQQQPLRRSLQPQQLLEHFQSPPRVPSP

AATAAAPRILSNKNNSEGGHNFNYVQMTNGNVQHVTSAVRVGPSQGRRRGPPAASTAAPN

QAMQAPQHAAPAPVSSAEERTSPLSAEATSKPRFRWLKRVANTLNSGRRGSASGKHTPAA

TAEMRSNGVQ*

>Lp_000053300.1 hypothetical protein, conserved

MNGNASSNGTGGTAPTTPHIGYGGISMNNSLHNNSNTSGGGPASNVSSGPASFPVNLGLS

SRPTGEADISNDVIARLSELATAALTSPDKDTRDGAAQQLLFLSSYEYWETLRTLLPRVH

NNYLRFIIVKAILFLVSNELGPQERTEVQQYVLEYIEERRYAGEELPFYLRNALFSVFAS

ALFSNWKIAIIRAESSEQMNSAEMAEGIFEKMRLYLTTDETLDCVLEILTFFSRQNSKHF

ILSVKGSFAKQVLPFFFSAAANMLSISPAKAAAVCSTALECAPELEAPLIRLYHPSDPSI

TVLRWPAWAPALSSAMNQCGQSLLEQPDGPHAAVFSRLLRQCSAVTSPQEEYIATRDEVA

RVLLDISQRLLFTVRANPERVELLRLSCALLVNTFERNDEATVEFLVQNPELIRVWADAT

RFILEKPFDEEETDLYQALLHLFYLIAERLLPPKPRLRNALEEPLENFYGASTPPGGSSV

ERHEERALSSQLRSPLAAPRGPLPSDRTREVEQQVMDVFRVYVDVVMDTAHQRADSQELR

SATTYVLQTERILQPIAEVLFCERIDLVPQLVERLHQTLHQYELCVRARREQASEQIGNA

NNGGDVNGRSNYGSSSAQPVLEELCMTMAVQNMHMGNGAAGVDASSTPLFFTHVCLSRLS

VIVSIFGIAMLHGTASRDDSVLGTIANFARELLGQEDQVTEDLLLCLSLFDDAAEGGADG

FNSSSANNNNNNLSGMPPGPSTSPGNNAQAQIHIGILRALFFFCGCVYETNLSRNEEFYD

ITVNLLCYVYRYHSDQVALVADANMLLNRIVDYGSSGTYFLGADKFMGLIEVVKEDQLAL

LQTPGQLGVAGLTSEAAEARHGFLTAFTFYVETRYYAGFPIADVVNTVTERCFRDDHMQA

NSCLAFEDLKAITKGIHQPDTLLVMLEATIRHKDICSAVVRCEARAAPDMIAWLSVLCSR

SRQYLEEQNLSSIPWELTSLVMNVLCLFFSFLSYPPSSTPSLGASASGPFPGAAGGAASS

QDFGVMADGPGLTNPPVRSNSNGGYGASSSFFNFPTDVVDAAVVYDISDILHTFCTASWC

NLGIVLFYERTTVEHFFCGAVELLTTTSVQHLMADEGRARIFNAIAAAVNSSGNTFQQLH

LLYMRQGVWNRLVRLLIQCLNYAYAPELVEILYSIVRSDQQAQLRIPQYQGLEGNTLAAT

FNEICTLIAVAPHLTYREMTGCFGLLQMCYDGAPILCGECADKLLDFCSAYHRVRLRLII

NSLRRGDGEQLLTQYMTYFGQSSVVPVLSAW*

>Lp_000053400.1 2Fe-2S iron-sulfur cluster binding domain containing protein, putative

MLRKVGARPYKTMVRRLAATDAAAEAHGKKAVLQLVRFDPETNFSRVESYEYDKHHDYMV

LDLLIAVKAHQDPTLAFRSSCCEGVCGSCAMNINGINSLACITFAQHVTTVGPLPNFPVI

KDFVVDLRHFFQQYAYIRPFVRNANLHRNKVDGIVERYNNIARALSGVSPAEGRAMDALQ

EEIASIQRSETTIAALLRIADAAVDAGNATQVVSVLERVEKCGVTLDPVKVTELLERALK

NYAAKTN*

>Lp_000053500.1 Peptidase family M20/M25/M40/Peptidase dimerisation domain containing protein, putative

MSSLKQQLDQMVKDVQPQVIEWRRHIHAHPCLGHQEGPTVAYVMAALQTMPAKLVITNPT

KTSILADLKGGAGEGPMIALRADMDALPLTELTDVPFKSQNPGVMHACGHDTHTACLLGA

VKVLCEMQEKIKGTVRFVFQHAEEVSPSGAKQMVEAGAMKDVDMIFGLHNRSSLEVGHTA

SCPGIASGAVIDFDITVHGKGGHASAPQMCNDPIVIASDIVMNLQTIVSRRIAASKVPVI

SVTTLQSGTGSFNVIPDTANIRGTIRALSDEGEKDAPKLVEQTANAIAALYGATCTFDWP

EVVYSMRNDQKCFEIVKKVCTEKLPAGAAGFNVVTDPSFGAEDFSEYERVVPGCFAYFGV

KNESIGACYSGHSSMFKVDEAGFETAVRIHVGLIEELLMPG*

>Lp_000053600.1 heat shock 70-related protein 1, mitochondrial precursor, putative

MIGRKFDDPDLQADMKHWPFKVTVKDGKPVINVEYQGSLKTFFPEEISAMVLQKMKETAE

AYLGTTVKDAVVTVPAYFNDAQRHATKDAGTIAGLNVLRIINEPTAAAIAYGMDRKNDKG

ERNVLIFDLGGGTFDVTLLTIEKGVFEVKATAGDTHLGGEDFDNRLVDYFATEFKMRCGK

DCRVNARATRRLRTACERVKRTLSSSTTANIEIDALYDGSDFFSKITRARFEEMCRDQFE

KCLEPVKKVLADADMKPQDVQDVVLVGGSTRIPKIQQMVTQFFGGKEPNRSINPDEAVAY

GAAVQAHILAGGQSDKTDGLLLLDVTPLSLGVETAGGVMSVLIPRNTTMPVQKSQTYSNN

ADNQRNVEIKIYEGERPLVSQCQCLGTFTLTDIPPMPRGKARINVTFDVNTDGILVVSAV

EESGGRKEAITIKNDTGRLSKDQIEKMVQEAEKFAEEDKANSERVEARNTLENYTFSMRA

TLDDPDVQSGVSQEDRQKIQAAVSVASNWLETNQDASKEEYMEQTKAIENVAHPILSEFY

KKRVMEAPPSAGAAPQQQQQEGAGGEPHDTAHGAQDVD*

>Lp_000053700.1 AhpC/TSA family/Redoxin/Thioredoxin-like, putative

MSFDLFHRNSSYVLTRQDGLTVSLQQALRTKRYVILFLAGQWWAPCRGVAAQLCSFYSSL

HDTFNFEVIFLSTDRSETTMLDFFHSSHGDWLCLNYNDAR

>Lp_000053800.1 tryparedoxin 4, putative

MAPLFDSKAVELLRKEGTVAAAEALIGKKYIMLYFSAHWCPPCRSFTPILKDFYARNKER

SSFEIIFVSRDNTAEELRSYFDTAHGDWLALSYRDAQTVGEGWAKQYGIYAIPSLVVLEN

TEQHPIITSYGRDMVLRDPAALQFPWSNADAIMIAARRSFMWKAAAAVVVGMFFLCFLIR

*

>Lp_000053900.1 hypothetical protein, conserved

MLNEGAEDEEAVIRQQTLLGVNLQARREELQSVQEELRAVLRAKEHAETTKQRIEVEVRD

MERYKSTLDGIVAFTLRELEDAADGVEEAEAQMRRSSQLALQDVLAAQEALRQRSLELKD

DE*

>Lp_000054000.1 Lipase (class 3), putative

MPAMTWFHRQWRVATDDFAFSSILYAALLLSSGVMLATRASTASNNYLKGCPGATVEWGR

SIMGLLCLNFITGILFLVTSAFSFRGGVFELSKRAAVPVLLYVVSACVCGLCVLALLSAK

YAIYDGETRRCPRASTQHVFRGAIIIDLIVFASFIVSVVVAYDPSGARVLRNNSDYANMW

WERFRICCCRCRQRDRADDAYTDLAQVLAAAFRGYDIVPSDIAAGMFLLHGYQERSRRLL

SSKVHYEPNPNGYIERVSTQARIAVRLTPHQVALAHELQYYSRFYMAAYGWMLFTFQHCC

TGVPRLCCFDPCMSCRSRPGQHVDQCCYCDLTALLHETMVPEEDVLYTNWHNTVYQPVHY

VAFDRSSDAVVIAIRGSMSIQDCVTDFAATPEVIALDDVNGKYPASEYYVHGGMLRGARY

VLEQLRQQGILQMILRGRYAAKRVVVLGHSLGAGVALIMAAVLWSDNPSLRGRLRCLAYS

PPGGTLSRAVMEYENDFAVGTSLGYDMVPRLAQHTFDAFREAIFDVLAASRMNKNMIFLN

IARTHAIAKPFHPSTADATAQEQRSTESALYRASLLHAPCVPAKEALKLYSCTTTIHLMR

TVRVSPFKSCPACCTFYTDEYFIPMIRGPEEVQMLMSSPTMFTDHFPDRYYRVMKKAIEQ

LDRGELDRFYVDSLPAPDPLGTDAAPMDFRPPRFNAEGQSVADYGAVV*

>Lp_000054100.1 Domain of unknown function (DUF1935), putative

MGCVPSSTYPEHIPRNDDDSAPFGTTSVHNGQNGDDYVVSRQVSVALMISGDGTHPVHSS

SDCSNKPDSLCTTAVPPLRMQSQGDVGADGIGLASTTYITSSGKKYKFLYGRPVGFSGAE

EIVSCLDDGGRQGNLYRLVRSKLRSIGEEEASMGDDTDGAGRRHTDTSSESEAKERGEGM

NLVACSVTARRQSANKSAERALQEQYVVALLGTDTILDTSAIERSRQRQRLAVLQTKSET

SNGKCNNSASETPRLSLPPPPSPPPAKAKEEADGLQMSTRRDLDLEEETDYQLFPQDHYS

TLPAQRQAGAAVPIALCPPSRILRVPVQYSAVEHRMTAAPGKPGSKDKNWKDIVLPQRVF

RLATSETRTWAFYNDSEYVMHVYTLFDRSSKLEPRDSTRLWPSRVFDDGNEAPTPPPVFF

GLFFDEDGEEAVPKSEMPDSVTRRPQHIFGTRGMWIAELLIPPRSTRLFVEGKIRGAYRM

LCTRLGLDDVVAATEMAQRVARRRNARHAPERLHLSLWPYRSSAAASASPSAATKAAAAA

EEQKVGGAAESLPLTSFKTMPHIPHRGNTVTAVSSVNGSASRSKNSAGLRKTMEAATRTQ

QARICQSATTAAAMTTTHPVPAVASHHIVLHTAANMSNPLSLPALRTPRAAPSQSLPPSA

AGPAASPPAFPVCKMAPTSLGADAKLRALPLEAAETETAAAATTTQSGGAAVPPPLSSPE

PVGEFSVIQKHTAAAAAVAVAAEDTAVNSSSKSSMQTELPGDHPVRRGKRHDSVPGDNAG

HKDSSCASETADQNAPHFPPSLSSFVARRIQRDGDNGDSGAEAMDDTVSDAGSSCVEFAK

IRPDRQRTTSLCGEVPLSGSTTSAHCKSTDQQQRIAPVRKSGVSRAPEALSPENTNTLNN

RLPPWQPKMSTLTPQSTISRCRSGTDRLSTSSYRTVVGDDSG*

>Lp_000054200.1 Domain of unknown function (DUF1935), putative

MGCTAAKTQTQFLNGKPTFQGDEVVKGFEKGNGLLFRITKKKKKQETWAFYNDTKQYEMH

VRVTFHPGCDIKPLGNAKLEEEGEKGEVVVTVTVAPGATEMFIEGHVNGFRSKMDAFKVG

GCDGAAPAVTV*

>Lp_000054300.1 small myristoylated protein-1

MGCGASSENANVTYLNGKPTFKGDDVTKGFEKDNGLLFRIVNKKKKQWAYYNDTKQYEMH

VTVTFNEDCDIKPLGKTRLEQQDNGEWVATVVVYPCETEMFIEGRVNGFRSKMDALPLSD

EYRQRQEEKEKK*

>Lp_000054600.1 hypothetical protein, conserved

MSWPKDPFIVDSAYTTPLPCQPCERELWRLGARLYEFDTLAKLSVNEATAAKAAGGEAPS

SSSSSPSMSASHITATPEHTRPHSITTPIANPGVSGSSSTRAEVVGRRNAFLAAAVAAAS

GTSSCSVSPPLAQGTGVGSTITTTGSRSFATIPLSTAVPAGASLSQPVSPFAVRGPASST

GSWSDNGSLSGTTSYIRAACQRVLEPLQRQLTDFAEQRMVLGAGVVDVAVQHPLPCLRLE

LVRLLLTYPFWDWAMCAPVAVAAGMGDVELLQLLLEVKSLDPNTGFPLTVAVRCHQEEVV

LPFLLSHHRIRPNYGGAFYVAVVTGNIRAMHTLGETAEVNVNRFANNESSSALLYALRQY

LICRRWEQQQEQVDPPSSTPLLHAAKPHQDVTSPTLLRPDEMKAAEKEISATPLLATHLD

RSAREGLAARSPPFAASPAAQQGGQLDNPLLSGRSSEVALEGRGQRSSHGACEVAASPRE

VETTAAVFDLPTDDAHNGAFRRDTKARPLLSARHWREVLLYLLDHPAIEVNAGFYMTPLQ

ICVMAGSAEVVSWLLQHPHLRPNRLPKATTTLCNSYYLLQHHALSPSALECIVATPIEMA

ARLNHFDIFRHLARDRRVQVPVRLTKNLECGAADGAAIPFLTVLAQYREEWEGWGWRWRR

RCSLAASVALTLFAACFWLMVFLWPTTLQRCLLVFTATYIAAAVLSVALYLWEVHGLRYA

ASPVSRGEGGGGGGGAADTSLTDGAAALLALVRHLCPGWSALQQQLAVAMLVACPGMVPL

LDLLCAWCLYRVYRSRRRLQSAPSDASAHAATLTTASPTSPSRRRRRHGQQQRAGERPCE

LERGSSTVSWGWSVGVSEFTPSSSFSHISTVAGSAGDAGSSHPLHSVASAVHNNNSSCCS

RTGGNVQPAEFHSTSTTISPSATVPGRMSSMASPSLSTNRNRGPWVVPGRPSRYLFTTEY

HAPDLLLRALAYSAFDRVLALPRLLTCAVGIIMFMYMVFPTAAPSPSSSSAVSSSPAAVA

EAIRDHSSFTLYYGVQLAATTLALPAMYNTTSGAPVMTAAAASQDLPAYTIGIGVVGLCG

LLSSVIGGGALLGMIGSLPAAVQAFFSSPGDRAGASREEGNKVLVRLPAGNTDYWAVRHH

HGRGALPEYAL*

>Lp_000054700.1 hypothetical protein, conserved

MSYLQPPPLRTPASSELDIVASIRQANARLAASAATARSRSRDTELSRHGGGDPVAKAAV

SSPSPRPPPLQPAVQANLHDRLRSTHSPPESVATAAPSLASSSASPHRELSAPSQLSAHA

RPPAPITRSPPHPDQRMEHVDMSPSPDVRQTGPLRVSNRPVRSPSPIVSTAVPSAAAEER

SAAECDSIQQQLQRTRQQLEEVQRLYSYEKRAHLQQRARQLRDEAQQCQTEENVVAQVAQ

LLTEYEELIKFRDVACAEHLEAVMQRVSREWQRSAATLEATREECAASLLSRLQASCAAQ

QEALKNAMQEHLTTTAQKEVAAETRQFQTIATAVQDQVESFKFEYRAIIEQDFEERRRLM

DAQTARREQQWLQFLKEEHARMIAAGETAARESTQRQLETLHAAMRDITELRERLVKEHA

QRQADVGRAYVDAYEGLAEEYAAASLETASFAQQLQQEYAAVIQQLHREIRRATDAKQAA

EEQARQMEVHMQEAIAVQQHDLEARVAARWEAKLQKERDSYRAALAQLARKHEDALHDLQ

ASCTEKEVARVAQYEAELFSLQTQLTSQREQHAAQAEAAYGELTDALRHLEEEKAALLKE

VSQLKEERRTAECAHAAAVQRLRSEQAEYFAAQLRDLDERYDAALKLHKERTNAGAPTSA

CSGGNDGDSSVVAAAHALQRVAQLEKELHDVQRQHTEDRQHAVDESTALWSAKLKDAQQR

LMEERDALEVQHRRLRQSLLTEVREREEAVEARCAVQRQAHEKEMQDALLAEKASAQRVI

DRLQREHEDDIKQREADAATHVRLREEALTVRERALSEAKAAWEQRRADEQQEALQALLR

RAAAEGAQLQEAQEAFAAQQLERADEMRTAVQAEAKAKFDAQLRDAQQRWMQLLEADAAQ

RYEAWTKARKAELEAVHILHAQEVSLLRASYEKELERLRHHHVRHIEDFKEEMQAREKAW

GDARAASLTAYEKAAADRLSAVLAQEKATWETMHQQQHQHQQETALDSVAQRAVQVLAAT

EEDRCRMENELRDVYVAVINDQEAKTAQYLTELHTKHEHELAALRQDHTTRLQQQARELQ

ELFATRVQQHETRLQKVLTSHAEEMSALRKENAQRLAEQRAAAQEALQQAQQQAMEAQRA

LDVAMRDAQREVAQAHQRQVDDLQKQIEAQAAQLLRCEADTRVQVQRAREEQQAALRDEY

ERSMASLREALDARNQSYATLQTSLYDQVHAEVACVQAKMEKAYVTFTQEQQKDVAERLA

AQAAAQVAQQGRLQEQLACLSQQHEVALASQAAKLRIDHDNACAALTAVFEKQQEEWKRL

LEVEQVARRTAEDEVRNLKAQTAQLEVTQTQQQATAYRALDQKYQHVLEQALVRLQAERE

ALAQRSLEEAEQRFVSEMLQGASVNTFDDHDSAPTPAAVPAFANRTHELPAVLTGMISAP

SPYHVSLAAESLPQHARNGVVTPAASRTPCPAASPLPLSPAGPVELVEHAEPGISSSSAT

TTASLSPSATSPVSMQQREQQQEKMALQRLQQLWDVLEVPPDDRHAFLDFIDTFPNDTPQ

RRQVWEGELQRLESQLPLLEALTRRDYVARQLRTLRKTTPPLSARTVNEAADLKSASCGG

KGDPIERLGDDSVGGKDTTKRVAASGSRDGGVPPELSQVYDRLVGELATLTAELRRDVTA

HEAQYGQLFCYNGQRVMETLNQ*

>Lp_000054800.1 pteridine transporter (truncated), putative

MGSNDRYSDVEAAEVVMNRSSSSTLHANVASAAHPQQQQQQQQQHGLVSNEEDYVHPESM

GIAHCAPGLFRIPFYGRSVQYLGPKVPSMLAICNFMQRGLANNLISYSKYAMFRSRFGTS

AKRYQRLAGVAGLGWSLMAFTAVFTDTFAFFHHTKRWYLVVSCLLGFAFALGFALLPAKP

SSANTGAAFMFLTVWSIANVIILVASQYSRVLRKSPRAGPPLVGVVYSCIMIGGIISAGV

QGPLSDHNLQQVGMIIAAVAQLVPAVFFGLNWLQEKTNEKERLEDCENNYQQALVEAQRW

DESHGLSMPEAEEEEQQTACCAGGALTKMLEEEEDEEEEKNAKVGFGGDSTRVPVLAAAL

DDDLDHVHPHHRGSTIDDATAPAQPRRVDQVQREEPCIVRLACGIIEVNYRVVTDNWKIV

VYCAVMTAGVIAMTVVTVLGNSYDLLYCSIAVSVVCILLAFYSLPLTIAKTNVYIFLQQL

FYLQLPGALDSFYMAPAACLPDGPHFSYTFYTTVGSIIGNVGGLLGVALFTYVFSKMSYR

VTMVFATVVQIIASIFDLIIVKRWNVAIGIPDHAMYIMGDQITYQVTYYLGYMPIVLLLS

RLLPRGSECMVMAVMSSLGNFGSALSNSVGALVIELGWPVVADTVNNVCDFTNVPYLILV

GHFACPLLIFPLTLMLPAAKISDDIDIDGKVIRKKVQAAVAADESNHVKAELSAASEPVR

EDQ*

>Lp_000054900.1 hypothetical protein, conserved

MQTRTRPTTQRPLQAVLSSEAPASTTTTTTTPAHPSSSSALPSPAVARHQASRAVAPLAA

EALSSSPPAEPTGHITLQTPSLVADPHCHNSHRVSTPQHTARSSTASSSVPHMPALPVSA

TTTPTARSSTHHASSQQQQQQQQQQQQQQQTDRPALTSPTAASVAPTAPTVPPARLRSLS

NPKSSRQEPTTGDDAPAPGDGGHDGGLPPSSSQQQQQQQQQQSRRHSSDSSSRLPLMLQP

GKLTSAPPSATLNASASCTLTSPARMISQPDTSPLAVTSPARRCCLSGGVVEDAEREDCN

NRSRHNSTNSDNHPEWRSGRAAGDTQARPRPTPVQNVETRDDGQVGSLPIPSPPPQQQQQ

PSSLSATHAAAVTSRRSTNDTTSPNSRNSNSGCPHRRRRAVSPEAEKTSSDAYLRENPRV

RTLVNNLYQQVLTVQPEDPLHYLAHLVFRTPEVEKKDGEQPTAPDVEEKSEVDAKELEAA

PQPTVVLTTAAVAGNNVLPRSGSHGSTYPPSARTTPPATSDVTATTMPHPSIATTTTIAV

DARRGSEGDEASTPAPPSAEVLTQQPQPQMRPPLSRSGRPLVTPRTTYPHSGSGGAVDST

AAVVVSARGRSSSLQPHPSRRSSLESGLGSACNPVSTATAGMAPATTTSTTANSCSSDTS

NANVNAVTVPGPVISPNVCGAAGAGGNNNNNNNSAMPASSGNSALSTNTNANSSNGGSGG

RTLRAPPPSAPTTSLVTRLISAGSPGSAANLRSTLGSGSKLGAPFFLQRSSLNVANGVAL

GHSIGSGGGTAGLSGHSSSRATVHQASFAGSLSGFERGEATPSEVSSLFSTNSADLQEFI

AEFRMAKEECCGGGVECPFITLDELATIMETVAFPFTDAAAVVDLFDELQPCARYLTNAA

AAAAAAAAATPAVVVVDGVSGGAGGVRAIGSGHIRRPRDAASASITATSASIRGSVNNVY

SYNNSNNAGAAATAALADGASKLRVSSSNPIAAAAATQRNPSEGGCADMAVACQWRTDEA

HSVVMSCDFRRAEATTSIGTTAEDNVATTAAAPATTATTSLAMSSFRELELRSGRATATT

AASTAEGYAALPLIPTPPLAKTISAESDDRAAAAGGGGANGTPIVPFDTFLARMTFKIHG

RSEAIRIAFYGMMIDDEVGSAPPVTQSGTGGASEMAITTTTTTTTTTTTSASAVSAKGIC

NVLSTDSLTPSEPAGTSTTTLTTLNYVNMPTCTVPLTRCLSEGLYARLGMVDVTASDVQR

GVRSAGLPTAPEDQRLCECQLEDFARLVRAVSAVADRGFSVSPANLCLSSLRDSSLPHGS

ASATNSWKEEPTSAPSKTGSGFPMHTIRYL*

>Lp_000055000.1 DnaJ domain containing protein, putative

MWRTPVYRNVQKRIHQVLTRQRTAIFFSKHTAVVPYLTFQRAYASSSSNSSSAGDYYNQS

KEDEEDKVLEEEEFAALREGEDDEFMPLTHRLAHKADALDAELRRLRYTLCDQPQFLVDT

AAATFLGRPTDMKAVIDLLLAAPKSTGITDAASGAASRNPEKPKTSHAASTTCSSSSNSA

SGGRFTSVLSPKLEAALRSSPYGAWTGTSAPVARLLCQRIFWPRSKTADAAASASSHGTE

LATMDADLDYLRRARALHQLNYYSRLNLTPKRHIWLALYQILWNLLIAAQNACGCVVYGA

LRGVKERGMVAGLCTGALTGAGKGLVFLAYGWVLSPLLHLSRGLCNSLYGPVNAVTGRYM

FDAISGRWMQCTVADTVYFRHALQREKRVLRTIGRAEFRRKRMKVEHKWSARMASMGFSV

ESFAEKMKAGKAKRGGRDAGASAKAEEKLRNPYEVLQVRRTASAQEIKKQYKKLAMVFHP

DVAQSRQGGSLSAEEKAAAQHKFEEIANAYQILSNPEKRKAYDMGGAQGVHLHETKYGKF

MSRTPEEMVQSIFGGEGFRRMLVGELLRSHWALRYEAQVSVSIHELEELQCIRVRQLAME

LAAIADVHARRPTTPYYGGAFSASTAAAAGHAQS

>Lp_000055100.1 hypothetical protein, conserved

MVDIEDENENTLPNQDCTALRGLSPGSWLLPMLPYEAAATASRTGICSHSNSNNNNNTPP

RGVNYGICDRANEAALPSTVSRNLCARESSSAPSDPALSGTSRPILCSGCGNTTCDSAST

LKHNASSSSSAAAVGAGAPRHHCALPALAVVPECGACVLNALFRELGAATHFATTPAASL

FNALTPTCSSAAAAAAAASGAGLFSSFSGGGANPNTLFVDPMFPGGVPQSGWGTGPESYA

AQRQAFVREMLVRQQRAAMAAAAAAAAAAAATATATATTAATSNAGNAASPLLSPFAGNY

LQSGNSLLGSPSTFAVSSASTSANFGTSGSGTVFPSAGAAPAHERVTAPVSVFFQVRSPT

DSAVMLLQPPSPPSGASVPSISILPITTQIELIGIPIDVSASSANTSFKTLLSPTNTLMT

PRNIPASSNNSNASSLPCGLGVAGVTRPFTLPEFLPQLTKLSTNVDGYLIIVLRCGSRRG

STGNASSVTQAAAAAAMMAAEEDPRLSTSWRVRRNSLSGPTPSDGDTRLGCLRLAEESSI

HSCFASCTHNATASFAGPAASAAASGSGAVRSRDKSRNDNDVGGVHRRYRVVEPFGGPQL

RLLFQQLQSCGVTQPALSVVDVCEDGPGSNTTNDVAKSPASAASACGLHNEHSTQPYDSA

VVSHTSTQGSHNGAGDHGSLDGRPQLPVSPADTWYSSPCSPVAVAAASSSYSSARLLVPA

LQKTATLLDDLSRVSEQLEKPDTLMLSSSQHTAQERSTCGEVVAPHSTAATTATTATTYS

SSSNKHELRRMAAQDHAMNSLRTTAHQLGLPFSCVVYPSRAAASGGATFPVESPLRSSSA

NSTGGRNAGNSSSWQTSTSGSSPAGGALLSRGLESLAATMVYRDLSAQQ*

>Lp_000055200.1 FYVE zinc finger containing protein, putative

MLHPRNWKDDHTVERCSGCATAFSFLRRRHHCRHCGGVFCGPCTAQQLWLRPWPGVPATS

APTDLRNESSGGGRRQANASVVSSAPHTPQHTHSNSSLSEASERGRVSCESHTNHTLCAV

PDALHADEGVETRRATSVFHASASAKGSPMQAEASRTPMSTCGAPTDVSRGKGERLKGKV

TAQVSAAGGSRGSAVGLTSSSTESRPEDDGSTPPQDVNDCFVSLPLSQAATAAARSCPSP

ANRQQQQQQQEQPQQRTPGSTLNDVTANSSTSTSAENKEPAPHILPEPISTAAVSTTTVD

SPQASDVLFSPSMYVVSPSTATGEVSPHAWATPAAGSGLERSASQWLRGASVFGASSSGE

ATSDASFHERSGDGQRSLQTVPAPHVTNTNASLHTSPNPDRDRGTAPATKAQNADAAAYA

EASYRAEVDQRGVTWYLCRICRSCYDALLDSILDAHEHSPLPPQQVQQAAAQQQLPRHST

PTAAETSFLPSPSLQPLWQYVRISSCTKTSHTRSGGHGAVQKSSRQQLQLGTAPQAATAS

PASLASPMTTKPLLSSAWARLRASVSLPTSPLASSALLLPTNPNSPSTTASPTAAAAATI

AGGGAAAGHASDKVSSPSPLSSPKLRPRGLRRSCDGSPSTAYVQPPPSCTVASTTTATVS

ATGHAAKSLPSTIGVPSLPVRLSGPAATTGTAPPSDGSRYSSPTSVGHPCEASMLSTSSA

SFMRVSQQSTEGACVSKQQQQQQQQQQQQPPRSSRAAVTPPRRRFISIGNSTAHVRQAVA

IARRRRRVAVILIDEREPLDASDVAWRDSAGFGRHPRGENVSAGERQAPQQQQQQRHAST

ESETETGPILILPCESPDCPDSGVAAGGDDVGAAKGRVPAQQQQQHGPAGAAARLPTAAP

ASSTVHRPTTAAAGAGAVGATPPSCSRAQWRSSRSRQPRH*

>Lp_000055300.1 adiponectin receptor protein 1, putative

MPLKANEVTVEEIKPAPQRPSLTIQKEAHRLSCSNASEENLPDSDAGSPPRHHTCAVLHG

DTAASPSENVASPASAASPSESVASPPFFPTSRHHSFFMNDLDSHAGDSDQSPTSSTHPS

LPTVQSPTAAFAGVMARSRAAKAAATAENAVHNQSTKRKSNSSSGEVVVAVKAAHPQAAA

TGPSDNPSMLTMPTAQNGTANDDNNDRNGDGENDESTSAQPSATQEQAVDVKYVVKSRKH

RHRLVATAPPVDPNRAQQWNLLTLGPDPSLPLYSFKEIPHWQKYNPYIRSGYRAFYTAGM

CLRSMLGWHNETINVYSHLLTFLAFVVLTVLLYATVLSKAITVSSMKASKLMYAIFCFGS

MLCTLNSSIYHLYNCHSHQRVMTAMGRLDFIGITVLIVASFLPPLYVVFHCFTTLRIVYI

SFILVLGSAAIIGPWTDVFHEHVGLRVTIFLGLGFSGLVPAIHSAFLLPFTSAISSVVTG

MLLMVLLYCGGLVFYVTQFPESKFPGHFDFWLSSHQIWHFFVSMAALVHYFNCVSMYQMW

QVSDGICN*

>Lp_000055400.1 adiponectin receptor protein 1, putative

MMSDTLTRHEACLRCRAIVNNKAAADAAKTVEEAKNKLIEKAQRHLSVHSAPSPITRNPA

LPLYDVATIPEWLRGNPFILSYYRAGYTTKQCVKSIFSLHNETLNIWTHLIGFLIVFALS

VHIVMNLELHRARDYLVFSVFQLGSLVMLGGSSVYHTLSAHHCEQVHNIALAFDYFGITA

MIVGSFYPPVFYFFSCSNMVRAAYLVAITLLGILGLMGPFFTFFNAQKFYWPRMILYSSL

TSVGILPTIHMFVSLPTNEQTVPLYEGIFLMLATYCVGMVIYVLKIPERWYPGHFDVWLH

SHQLWHLFVLGAAVVHYFTCIGAFQMWRVTGGFGGECS*

>Lp_000055500.1 Phosphorylated CTD interacting factor 1 WW domain containing protein, putative

MLHADDSRALLEEILRFRAALAIQRELERVCAEHLSAPVADAKGLLPAAIFEGHQQAAKR

AAAAAPSHEEVVCEASEARTWVHPIIDVRRRATVRRTSASATEATGTTCYRLRGVEYVKG

DIVGVVEPLEESGQAKTTQSVSLKELCLATAEPLYSDALLPALEAIEPHHASKSLLDLWL

NKLCGSADKMDSSFNQKRKKAWRLADEATYLFYVARKRLAEQLSFLSTSVDDAVASAAPC

KLTVSLHTSSARLRQFSCLPPDASSFFSGTAVIVDVQWNTEAATANRLREWLLPESVSAE

GSATAADGEVGWSNLDSGATTRLAIPITSDAILRLHQRYTEAAATVAPSLERSHAFLCSV

ARLLLRYHALSGGQMEKESGWQAAVPEQVIDVFDHYPVTSSSCCATPLSDGMAQSALAAA

HAAVDVTFPPHIVTVECFASPLNATRRFFFSVFHDTDAAFGSLGNLFRCRDAQHCLDAVS

DAAARAAAPAPHPTATLLRLECNPPFDHEVIAAAFTQLLAWLQTSSSSTTELLVSVLVII

PDSTKAHANEVRACAEQSPFCRWLRSIPASDCLYLHGAQQQQQEEPAVSVASATTAPTMA

VALTSSPHKRKRARKDADDDNNNQRGHATRLIQLSCPTRVFVLQTEAAEKLSSGVAIGEE

VARVWRLVSRPFVR*

>Lp_000055600.1 hypothetical protein, conserved

MEQTRSSNDDVDPTAILVLVTGLPASGKSSFLGAAQRYIATLEDKSFSLFGDKRRGCIAA

VLQLDHLLMDLHLPQHPSDFPEHEADNSLASSTQLVLFSPERWKWATEQLLRLTEQALRA

CASEAARVRNSAQTSKAAIPLIFVEDNMHLRSMRDRYYRLCRKVEEEEQNMAQQSTEDLR

SASRTFFALLELRFTAPLSVCLERNAQRRHSLRCDRDGEQVAAKALDVPASVIVSMDAVF

DRCYDAATTPKAQEQRTMLGCSDVNSPLWRWTSSTQPWSLLTLACSQHPAECAAASEENG

GHERAPQRLPTAEALVAEFFTAVLQSAARHACHAQYAQLIARRERRQREADTADNSAMSR

TQQDQARVQAHAHQLDLQLRALAHTFLTEQRSATAQTANSTCGAVVGKRASKLKKEALQR

FKEEHAEQRSHQVSQEEEEEKMAAMHEACFLKFHQSLVSLVSGQEMEEKKKR*

>Lp_000055700.1 hypothetical protein

MSNDSSEPYKLLPLPFVVRLPAVIAVAAGMPSETAALDLSDLTAACVETKAVGPGSTHPS

TTQDITVSCGDESSSAPRTAPPHRGPWVCQVYNGNRPDLTIKVPTLVLAPSQAAAPLSSS

QEERSDQSSCSDDTISSHEGSFYASLRMEQKEAEKQQQQQQQQGRRQNPARDAYSNSLSS

ALTGASEQTSESSLQAEIINFGLTPTSVSPTIAITAMTPSSPFTPFSPIAQQQQQHMPQP

PSYCAFRPSCLRVIKERDPSFLAMSATSRASRESNSKSGRSPRSVASSGPRTPQEFLAWR

RELAEARCAREAVQKRKNEVEAEEGLANTTGAVECGPDPLNSDVVVEHAARSCSGGVQRQ

PRPSAFPTTVAAVGTTANVTQLESCLLPRSVTPPVAEVFHRHQPRCGPLSPCTRLADPRG

PPAQSAPSPYDRNLHPLGDKAPQLQQPRQLTSSDDFSRLSQLSSSMQSSQASTETAGVRK

LWLDSAPRPPAAQGKLGRVELLRDDREGWNRGEQVAMGVVLVAAVLFLLHMFFFVDVTYY

LPW*

>Lp_000055800.1 hypothetical protein, conserved

MSQLAPTELHVVKNRKDVFEASSAESTSQGESCLASTTDYGPAGLPVFQNPASEKQESED

ENSLFQGHSDSFYASLLLEQQQRLRPAQQDKVTPLSRSRDADLDTPGSCRPSFDTAETTT

PVDSTDVNSPLFSQAHPPRTLQDLLAWRQRLVEQRSSEEAPNEHQEEHEHEQQQEYQSLE

QPSADISSHLDTSCNGGGIQLPVQRAPLPPHVMSHPVLSHCSPQSPCDATLDRVRAVRRY

QTLPPYQVSRKAESNEDVVYSYVAALRYRTLPARVRAAALSVEPPLPLSAPPSKVTCPAE

LSATVMHDPLDDERVSFTLRLLEEDAEASRTKRFAFMCLFLLLISFLFEAPYLPGGKMGI

F*

>Lp_000056000.1 hypothetical protein

MPICECVVDPNILLLPSALIFLYLGGVVVSIVNTADYFQKRKDVYPSALIAAHLSFCVIG

TVVFFCVSSVLWLWSAKYSFTHAERMWRLCVGLWAVFFFKDLPLLIIETHAYLQVGWQKG

NFMDASFILQIIFFVPSSLATSATISWYAAGFLERQFGDTMQVSLQEKERQPRRVQPEVA

AQLAAAAVLHPPLPLQETHDCTTHPPRIYASSPPCDRNAATEVAHEPLMGPPPTNNVVAE

YSNDFVLDQGGVHVRLPVNDLHVVEDESDASTYPAVI*

>Lp_000056100.1 hypothetical protein, conserved

MGQGLGGGALLGTALSMAAAPARTTLANHLLIIATGAGFGLALDFTSDCLAKEGQVYIHR

DVVLNAKDVLRHVYTFYAPCESNAMAKFFHQKHEWLVLESEGRRFYTVQKCPASGNVVMD

MRMSLRSANDLGLVAAGRPTQTGEIRQHRADMEFDIPNDVQVAYMIAWLRKEDPRWSFST

ENSRQFTTRARYALNDF*

>Lp_000056200.1 hypothetical protein, conserved

MNRDQIARRIDLIGPSTGAIVSVATWCALHGAEAESIMAYIVEKMKHKETSDAQRASLIY

LIHELLLTCATRGVSDSAKRSILIAVSRMLPRAVQDALRQKSSDHTVFVVALQKATEWWA

MLNLFPTAWLSQLQRSSQAAQEAVSHHTAVPAALLQVATLMQRYQHAKEIWLQNKHIKAE

EGVSTTTTTTAGSGTGVNGQDDSGGNGGGASASSAVVDDAAHRCLVALRKAVESRFERNA

ALLAWCEAERAELEGHVVAHAGGGAAAGLVKTEPSTTAASHGGANAAVSVKEEAGVTGGH

VAGGRSDDDDVLGSFFS*

>Lp_000056300.1 protein transport protein Sec23-like protein

MSSGGYGYGGYGAQYDQQAQQTQQAYTSGAPSAYGVYGDAQNEAYRQYDTYQQQQQQQQQ

PYNAYTSAVQSGVPQGYQQDAGQPQRREQQPVMGVVENGEYDAQAVTEAPVRWSWSLYPM

NRIDGARMVAPLGCLYSPLASPCTQLCYAPTKCTVCGGVLNPYSTLDPRSRTWGCPLCLS

KNSLPPQHQQANEYNLPVEMQASSSTVEYVAQMPARNPPTFVLVVDTCLDTDEELQGLRD

FLLQSLQMIPEHAKVAVITYGTTVSVHEIAGHASYPRSMVLRGTQEMTVEQLKVILPYPD

RFVAPLGSCVGQVTQLISTMSRDLWPVMKDHRPLRCTGAALSVAASLLQVVSPNTGSCIL

TFMSGVCTSGPGIVVDVSREKMIRVHADIRDETSAASYWSASCSFYEKLMHRIVAQGHSL

NCFVASLDQFGLAEMKMCVQSSGGVVLNAESWQEEPFRVSLHQFFARREEDGALKMGLNA

TFDVITSPTWKVQGVIGPCVGTGKKSTSVAEYEIGLGGTCQWTTCQLDSTTTFAIYFDTT

SMPAGEAAKNPLRYTQIITRYEIGQETRTRVTTLTLRQAANPPIQDLVAAFDQETAAVLL

AREAVHKTDSMPLFDVLRWLDRTVVRLVARFGTFTKDQPDTLRLPNEFVYFPAFMYHLRR

SGYLQIFNSSPDETAFLRLQLLKSNVEDSIVQIQPTLYSYRMDGNPQPVPLDSTAIQPDN

VLLLDTFFEVLVHYGATIAAWKNAGYAELEEYAYFKEFLEVPMADAQVLVSSRYPTPRLI

NVCQDDPDARILYNRINPSRSYASAENGAYGSNEGELVYTDDASLQVFMQHLKKLAVSQ*

>Lp_000056400.1 diacylglycerol acyltransferase, putative

MDDVQLQAQSPASPLSPLPDKAAQRKKTPKRRSRTAQMTPEAETQLPPASSLGEKTAVQN

PTSMSFRAHVASTIHYLASEVSGHSGGPHSTSNSNASNTSQTSSQPSEPPLTHARPPASP

EEVDEQLDRILYAINQLQQLQRRGRYIAAREVRNLRRAYLFAVGEEQLYREALQAEAEGT

NMTSAASPAPTTPAKQQQHHGLAGSSKQLWGRAAPAEGAPERPREDGEKPQQLSKEQNAQ

SNNVSSGASASSQSAAPTWLVKADRPAGTSSPASPPPPPPQPALNAAAAECPRAGDSSRV

EYSPKAAAANRLLTALEQYVDETPTSASFDVNGARFSWRSIIDTPGERRLQTLVVSLFIF

FTGIPISFAIFFLLMLSRYTAPLMVLYFIWMFTFGRPNHPHAKNWRFAHLAIWRYYRDYF

PVRLVVPRSVRRKVEKDRNYFFVYHPHGVHTFGALLNFGVDSNDLRDMLPNITVHVQTLK

ANLFVPFWRQLTIWMGCGDASSSCIRKTLRSGPGQSIMLVVGGAEESLMAKPNANDLLLF

KRKGFIKIALQEGTPLVPVYGFGENNVYNVADIAKAPGVQRLLELFKRYAGFAIPLVRGR

GFFNFSFGILPHRRPIVVVIGEPIEVPRIAKPTAQDLDKWQARDIEGLRKLFDEHRGIYD

LEATGLRIIR*

>Lp_000056500.1 hypothetical protein, conserved

MFSPVYEDVSGLFGRPMPVSSSSCGPAGELHFPSAHFNNNGSVTQTPPQQQQQTMRPSFS

LVDAAGNAITQPQQQSQQPPHSLASSVNGISFNTLNGSSFIPLELYLQQPQPQPQQATVL

SGKPTAFQTADGVKYFLSASPSGAPILAAGPAAGLLRQQSGSSPVYTMEHTGSNVNALAQ

SGSFFSTSLNTNNNSNSNGLQSSSGDFSFFVANNLPSITTTTAAAGASTLAKHVNGSVTG

ITCLSPDQRSRTLVNGSPTLNMQASVNRNLSVAELMGNKTTIEVFDPDFKFIYDVPSSAV

VHLPPARLNITRLVLCRNYRPYDPQSCAMGGNCKFVHADCDFTKLEAHPIHVNYIWRHES

LCTYPRLSAGGKRRVMQSDGQVAEIVTERLLVTKGSLASAQSRSGAPLNICEGYENNGMC

YQGQRCNFIHTVHVDPFVQGDFKRAPRKRELSSASSGHGFSTAARQPSNSSASAEHSQAQ

SQVFQQLQAQQLLQQRRSTGSPDTETSTSSNMTRPAVRNARASAEPSSLLSSATLTRLQE

AVSMQLHTPAATSPQQVAPSFGISLTPFAVQTLTNTASAGTLLYLPRGAVEAVVIGPVGL

PEAPKSDAPTPRSQK*

>Lp_000056600.1 ATG8/AUT7/APG8/PAZ2, putative

MSAYVSSTPVEERVAKCASLKADSCSVPVVLEGAQGGKVYFSVLPRDTTVAQLMSVVRRF

DDAGAKKAVSLLVADCAVAPTTTLGELHDACKREDDGMLYVTYTSEAAMGGALDFCCLHV

D*

>Lp_000056700.1 FHA domain/Zinc finger, C3HC4 type (RING finger) containing protein, putative

MSAPDPSKHVSGTATLNDHTPDDASFADEAAVAADLFDLAAPPLRQPLVARLVPMYAGLP

TLNLHQDSGTVVVGRSKELSEDYRINGCDKLSAKHCELIVDPVTLRVELRDTSTNGTFVN

GVRVTKNEKVKLQNGDKVSLTRPTAAAETAGSDSGGSAPAEASIAANGRAEFMLQRLKCE

TTKADMVAELTCSICCSVFHRPCSVLPCMHVFCAGCISGWLWRQQREGIHHTCPECREHI

TDVRPTHRLQCCVEQFLLSDPGCRRSAEEEAQLDAADTIPPTGLKLGKRQRGSEDEGESD

SDSFDYNDTSGSSDGNTGTLRHVGESYVNRMGLSAARCTECSIPSSIDGFQCPAGGPHLR

CTACRLPFAERPLCGRPQRCHVCNNAYCQLYRAGGCSADTLQPFRAYELHTPVEVLPPQV

FGGNTIEQGIFSTYLAAHNIMIKDAWTTTLTKFAAGEWVPDMVMINGAMNTNSAVCPKCA

VNVFAELLFHYRRSIPVAELPESVTRRPNCWYGKECRTQFHNPKHAQNFNHVCYQEKRKE

*

>Lp_000056800.1 kinesin, putative

MSERLSVAVRVRPFLPHESLASCVTVHGNQIAVGESKSFAFDRVFDMNATSDDVYVTLGQ

PLADSFLSGYHASTIAYGQTGAGKTFTMAALLSDTVQEIFCRLTEEEEKRDSCSSNNNSV

GVVSSSGPAASLNTAPTFTMSLTVLEVYNEAVGDLLSRHAGQPYATSIGAGGGGARGGGF

GPDAKKSVATQRSALQLREDPTGGVYVVGLTEVAVESEARLLALIDDAIGNRKTASTLMN

ATSSRSHCVITLTLQRRGLCSRCCFVDLAGSERLKKSLGFGSPTDRRGNSSGTNNGNSVY

HNGVINGGGVGSPPEAALAPANAAVRMREGININSGLLALGNVIVALCERKPHVPYRSSK

LTRLLQPMLEGHARTAMIACVAPVASSLEETLNTLKYADRAKRIQIDPHLAVAATTTAAD

AQQLITLLREQLEEAKRRLAAMASQGHSNSSSGGIASCPAAAKDLLAPSSADVEQLRQLL

VQEQEITKRLENDLFNAEYTAMVEVEKRKALETRVVQLEVYISDQRHQSGGNNSNNNNNS

ITDNTNSNHGSTSDRCSSERNSIELSLHPTHHRVLLDETSRFRSHSAAATVGADPSSPRA

RLTQNMARLQQLEEEREFLAAMRAKRVRDTQWLEAALAAGDVAAAEEGKGEEAVVRHDDA

SVFADAADADASDHLTMDQLTEEIQRKESQIATLQKENDEVSAQLAQYEKELHDTLAEKA

RLQAELRRAEAQLEKSAMEQAQKEVEKATLRASFLDRIRRAEVKATEYRRRVREAELEVR

TRQDSINRTRQLQEKVMQLREEVTRQRLEVRSTKKQSDQRSAAHQHEVLQLQRQLQLTTA

QVAQLHQRMDRKDAAIAKVKKLLAEQQRQQQQRTHAQLPRPHTPPPPTAAAKVLEPATTT

PVTVHKVPPLNKQQPSSRRISLKQQPSSTATPSTVSLPDTPPSGTSPVRITKRRLTLLSL

PRSPQPSQHDAAAQALIDRELADLERMEKELAELQEYREVLLSAQTTDAPKWHRAREGFT

RRLAAIQRELAATDAAAPAYANLQKEEADVQEKLRQLETYRHMFEDADEQLAEFENRIEN

LNEARRFHLQRVRRMQQQSSASGATRMGDVSRDATADAGSDGEAALPSVREIHSTRLAYS

ANSLSRKSAGSDTPPPSPPVPRLRRSLGHDGGAAAYTAALEREVARLKAETQMLQARLKA

V*

>Lp_000056900.1 hypothetical protein, conserved

MTDVQSSSQQQHRRNNRSGSLSDADEVVDYLQLAGRRYREHVELLRQEQAVREAQHAPFK

PTISAYAEQASKTSVVRQGSSIGNRLHELHQKKLAILAEEAAESARRREAEERSVCTFTP

AVTAMADRTLRRHSNSNSDDDRHVSALLLRWEERRRARQVRQQAEATRREWASVTGVPRI

SAYAVEKAAAERQRRTVPIEASLLAEAEARRRRQHAAFEQSYATSSTAALTVDEEDRRHG

YSHTQRRAGASPPPSFVPAISSYAAQIQFQQGVVDRLYTYHRTQQQQNGPRSHSRGYDEE

MELHCTFQPQLSPQCAALSKLYYDDEGEASASNPHDRLYRNAHHPSKYRKSMLPDTGTGQ

PIINDASRRIVEERRRQLASDGDPGALGHSPGSRLYPNTTAPDKKRQPTFKKKVVTARDV

ELQTALTFAPAVSPTSEAMWRHRVTALKASGAARNTVEARQLLWRKAERKKEEEVARMQA

QRRREEAAACTFHPKAGRPPHRRSGYIAMPIEARTTLWARQRDARLRDLRAEAEASAVEE

CSFHPHIDPVFPLPRQDAALATGVEAFVERQAEARRLREEAKEWWRPQYARKSIAVNRSK

VADVADASVAGRVEDSSLRRNGTSALPYDDDDGDGDGDVPLSSQRESHEEDDESAEVREH

RISTDGYHAEGDEEEEVFVQHWASWQPPSTSLTASSFSHVSQADVPSAESSPRQRAYVVQ

QPAATSAGPYRWKRSSTLSDTANQPAASPTPLSWRKPLRYRAR*

>Lp_000057000.1 hypothetical protein, conserved

MQRYHRLLCAATAPPAASASPVHDAAARHAALFSLYRRLLKAGEDGEMMQHCVSVHGLDD

SVKYGVRLLRLHRGLTTVDAAARRTRWFHFIRRTRQELARRYYAWSIFYLRLKLRSWNAI

SDMLVYVLFLTVCVLLYEIYHTCRVGVNRAEERYRTLAIPIMQTFDALEAAQERKKMLRK

EMEEDIVRDR*

>Lp_000057100.1 calmodulin-like protein containing EF hand domain

MFVVQVAADIFGNKLNFELSFPSRPTVQEITRASEAAFSAEIANTRAENVPPHTFHVAKI

KIYDEDKSKWVDLLGEGQLVDYCQLYAFQPENPWHKETQKPIPPATKPPVAASPTPSRSV

AGGAAAPSASRPVTSTSSALAPYTGGRSEPSSSRRPYGASAGVGGNGSTALVIPRGNVDA

SPEEKLRVVFSEFDIKSARMIDLDDFKAGFHNMSLDFSSATVEDLFERADVNHDHRISYS

EFERFARMYPIMTDCLYFRSKAFWEEEQLKKEIQAEVEAAAKSEVVLDSAQRSLESAEGE

VANAQNAVKAADEDLRDRTERMRELAKDMEDARKEKERVARDKKDREQDLIAIKEREKVA

RKDLQDYARDSDKLDRRAAALVNDADAADEKVRQLMKALEDAKRAADRAHQAAEQAAGEA

DQAKAREKDAAMEADAIAREIPKAEDAVRVADRNVSAADVALKELDSAGKEIGRQADEAA

ARRDAGEKSVAEARDRVAQRAREVDSARNGVAERERAIKQKEMELDEHRRNRELITQHER

TLIEQELRLREQRDSLEQRETKLMSEASNYLGNMRNNLATRSYSRDTAGY*

>Lp_000057200.1 RNA-binding protein 5-like protein

MTLSFDIIAGNNANWGFDAFSFAPELTTAPSPAVENGSRRQGHRLSDSGWATPATATTAA

AAAAATAAAAPRRLSASQQSIAGANAALNENEENISSMKNDALYVTLQTSDPAVRAFDSN

LYVASLPDWFTDADLYELFQRFGPILSAKVMCHKGTHHCKGYGFVLFQRTEDAAVARSEM

IGHVVGGNKIQVRRARSAASAPLGDCGATSSATAVSGVAAFADVPRATLPTQAKAAALNA

SPPFLASPPQPLPALYSTTPTYVVTNGSEVVNGAYQNSSNSDGTNASNNSGSGPAMFVAL

PNGRGVVQGPDNVVYMLLTSPQLQASNVIF*

>Lp_000057300.1 RNA helicase, putative

MGPNRQRKPQSRGRGRGGRGRGTASAPAPAKATPSMITNAGRELIIDVLHHAASGGSVPV

PEKHFDANIGSRITFQRNSPLTPSEILQRLYEGLGFPRDLVTQYLTDLAQASAVPAFSDV

SELLDETGPFQSFLVSSSFELFNCMTWFAREETEDKMTPEEGDAMLEDEMTTVEGLFSDS

FLGRLHFDDTDTEDREVYFSFHTPEDTPILVVIRFSDYYPTETPSIYIQPLRGSTTESLP

ALVVARSCTKDISAVNRRAILDAAVHAINGFVGTGCLLALVSAIHGAVATLEASQPASST

PQQDREAVLKERAEAQQHRKAFFNALIGKQGNSNGNNNALDDEEQDRFIQLSMPEKVELA

TVDVDAVSRDTQRREFLLKDAALDASLQSSWAKLQKEGSLRKSREALPAYKVRETLRDAL

KRHNVVVIGGETGSGKTTQIPQFLYEFMCEEGHGSSANILCTQPRRLAATSVALRVADER

DEAVGGTVGYSIRLENCVSKKTQLTYLTTGIVLRRLQTDKFLGRVSHVVVDEIHERGVDT

DFLLILLRDLVRRRSDLKVVLMSATMDSELFARYFDGAPVISIAGRTFPVKVMHLEQIIP

EVNYTLEDGSPYEKISGDRNTRRRNTRKNMLNLDLEDAEEDVEREKDQRKLARAVHASPK

TLDTLARMNYDVINYELIEYVVDYIDNILRIPGAVLVFLPGMAEIQRCMEQLSCNPRLAK

SCLFYNLHSSLGSSEQQGVFRRPPPGKRKVILGTNIMETSITIDDAVFVIDTGKAKENRY

NARKSLSELVTVNISKANCRQRQGRAGRVQEGFCFRLFTEAQFESFEDHQLCEMHRVPLE

SLILQIYALHLGDEVEYLQKALTPPEERAIHSSVKVLTTLGALTAEKRLTSLGQHLANLP

LDVRVGKMIIHGALLHCVDPVLTMAACLASRSPFIASVDFRTEVENTRRAFAGETLSDQL

SSWFAYNKWATAVQQKGAGAARKICQDCFLSPAALQQIQSTKRQYERYLVEAGFIDNAIC

GHLSPNKFVFPPFTTMDDRVFEAGGQHFNSNSSSSRCILACLVAGLYPNVAHLRVPRGPK

GGGASHHTGGRPTVRFVTFDGSECLVHPSSVAGKETSFTSPLVVYVDKVKTSATFLREVS

VVAPLHVILFGGGNLEYLPKYEELCVDEMTAFKCRQDDATLLTHLKTQLDSALTQKINDP

SKSWESISSAVVRAIVKLLKEDGGRAGALTIIDRRQPRAPLTEPLVPDSADTAAAASSPV

VPEDQPFKTNKSCFLCGETGHVSRYCPHNSTHNKGGPPVRCFICGQWHFPQDCTLVKPLK

R*

>Lp_000057400.1 RNA-binding protein 5, putative

MSYPSPFSRNVYIASLPEDYAEKDLLDLFSPYGRVISCTLKYDKDTGLCRGYGFVLFENE

QDALNAVIALQGHSIRNTRVQVRLARPEASAKKMYPAMMQQQLMSAYMPYQPVYVMYVPS

PMYSQQQSSVQG*

>Lp_000057600.1 N-acetyl-D-acetylglucosaminylphosphatidylinositol deacetylase

MNSLFLAVLCLLAVAFVLRWVRLSNLKGTVVRGDVLLVFAHPDDEAMFFTPLLTSLRTRN

ISMHFLCLSNGNYDGLGKVREKELVASGAFFGVQRRNIKIVDRAELQDGMRSAWNTTVIR

KEIEMYMQKAGSISTIVTFDRYGISSHPNHIAVHNGVRELKENMPPGLLYLQLRTRSLFT

KYVGPLAALPYTMFSATYVSRTNFVALVHPASAWASMAAMQRHASQLVWFRYLFVLFSSY

TYVDELEEL*

>Lp_000057700.1 Peroxisomal biogenesis factor 11 (PEX11), putative

MMQQEEGGQEALRVLGRNPFEGITTKTRLRQLLQTHQGRDKLFKVAQYLIRIKLWWNSVD

FNVNYMPGEEFSRLEQNLMTIVNSRRMFRLGRFFGEFVRMRVTLIKASELVYIPVNGGQW

IALFIQCQMILDMIARGLLFVKSLCEDVAFLVQKGFFHSNVAGRLIYIATRCGLPVLTID

LFLNTLRLYQGILDASVAEPTKEIMFSKENFSLLSKYDRVDKLRRKLASTTDEKEKLKEA

LKAQQAGTDAASNQNNVYVTSYAQLLWTDFELHWICVTETKLLLDIFVATSNLRGWKLQG

AVSTAGLLSGFCSVYRVWTYGR*

>Lp_000057800.1 hypothetical protein, conserved

MEVKKFIRLFPRSALSARYVGSLDNVEQRDVIVTLVEDAIFCDSWEVYFAFAARNNVVIP

EEVALTAVQAVGRDPLSVTLWVKAASCCGSSESRMRLYEFGLSVPLYNWQKLYAEYRAFA

EQNGASDILAEEECSRVADVARHESWPDRYARFDTDGEVEDVRRGWLTLISTMVSRLDSG

SLAKDLQLRRIELAMRQMCSQLPGDDSCWYQYALFQLRILEDAEAARKTVESALAAAGPS

FALENVASAASAALAGEGSDGNKDMSPVSTATEVMLEQRRAAESLALKGATADGIRCLRA

AGEAAERRNLADWKVYSQWCTAEDMTAYDPKMATQVLKRGMAHCAHSPSDALLLGSEAAR

YHLLQRHGDETIEYAKEQIEQQTALHHRGRMLASWNALVRIESLVGLSFSKAAERRAEIF

PQTPITTFLERCRVGDYLPCSKATFQWIQFTEDYNVDKSSVEETPFQGFVSQRKKALASR

PRTIVEEYESPNDALWEAFVPPENSHPPRAADDPDEVTGARELRGKLVYRVKVDSRTAAR

CRREEKTRRQSRLGAEPAESGSALKSMLGRVKAVSLTEYQTRRLNAVSADWLVRLLTVSG

VGLGRVLKERAERRQSEEEPQWEDRR*

>Lp_000057900.1 hypothetical protein, conserved

MPDDPLFNPTNMQGLRHSSRFAALGMLAVIWIMYEKLRHSHHYITYKGPENPFARIRHRR

YPGGSFMFGWGNNGLNRDCGVKEFECWAGYTGKEYTY*

>Lp_000058000.1 hypothetical protein, conserved

MTCITGDDTGIVKIWDISRSSGATLKFSYGEQSRKRAIMGMCWQDSSTSSVAFSSNDGVL

SLLNLSDRIITSAIKADCVAGLPNAMSFVKGKLVIISKDGTVTSFNSDLTGVQSFNGNGP

LDAVHIHRKFGMIAMGGRENDMCVYDLASEQLDVPVFKARNVRDHILDVPFPVFVTGASV

INPYVFATTTAYHQVRFYDRRANDRPVQEFEISREVERRPTTMLQWNANKFLIGEASGDV

HLYDTRRGFTSRAKLRGGVGSVRCMAKHPSGHQILGVTGLDRKARLYHVPTGKLLMSIYV

KQKANCVLLDKQMPLTDKVSSFSGIVNTKQPEKANTLGDSIWDDMDPVVDELDEQSEVIV

EASQMKRKARRTG*

>Lp_000058100.1 hypothetical protein

MMVVESLQAPTGPTAEAAPPTTQSTPPRLANEDPLRTVFLDDVPLLDSPAWSSDDELDNP

TYNKRFRHKKHEEIRLRLQQRKQEERERQLQLEKAQRQTSTPLPAAENGAAAAAPPPPAQ

PTVTPQPQLTSCGAGSQLHSLKRTKTGRGRRSRHPTGSTPAQHRCHSVKKEQSLTAAETG

AHTPTKKPTKAPAPAPRAPSQLPEVVPAKQRHTRPLKAEAVTITSPPPPLMAAAKRTSMP

STQASQSPRQAHASVNAPISLSQTQTQTHVIVSPGATPRHAGTSQQTQTPTISLTVPTPR

QTTITSQTAAPENSSRVVAHNAPSATTVALSSTEVQPRKVRGSHTPSAMFFVSKIVKGGS

VPKHSATMPTPIAVATASPRHADADMTLKRTSPSTAAYSSMSSRDDATSLPAVLNVTLPF

AETESSQAEKREKERGGSSTEAEAAVGFVVTLRTEAAPARSATAPPLPPSRLGNAAARSP

LLASGEGGADVFVPVPPPSPPQLSAELVKACCTDGAATTISEATNNYNNSSASTPSPRSS

VVALPTFDEEQLNSHRLRSAHGKHLRQSDYYNLNGMPSYFRPMMGGHTPRTDKTGAVDIG

WSADKVEAVATAMATLDTAQIIDDWERSRPFDSQDNYSAGSADNYDYDRNEMEGETEGGT

NANDDDDDSSINSPSSNIHSGSDDDDSNSGSGSWSDVLSDASPDSDLDYVLEYHSTYYGD

LLKPTDTPPSRETLSVVDDAEKQLEANPKSTEAAMKADSCADDDGAGEGGGAEVRRGETL

VAQLDQVTVAAKQSLHYSPSSTHSSGSSLTREHDFYLDEVHGTVTPPIYSFYNGLQTNGE

TEKAQKRKKELLFLSHVNFGKDPYALGMEDSVIAMSAPKSNNCIGNAAGTAEATTLPHLQ

NSNGRRASGAPVGPPQPRRGNSAFITDDADPWAYHQARRESWSSMHKRAPSAAGQNDNFC

KRSSSIMSTKYSVLSSNKGLFLQQEGRSTRAPLPHSTTPTTTTTLPQTKPPLQGPKEVLA

LPLQATLPSPQPQPASVVTPPGSPAAAARAVKPLSLEIFGSQTTNNSKSFDLLSPVARKS

TAPPEELAAMKKEKEQQEGEDTALASHSLQELQLQKRQQALFSFAGLQYHPPAALQEKQS

TAQAPAPPSKPSKTAPMQPEELRECPVVLPSCRHAAPMEVPPPLPSIGQPSPPSQDELSP

PLMKDAADHDRGRPPSFEVELVSGRHLFPVSEDATMDGAHQCDESDGAVSHEVVNRPSPH

DVYLPITGVEVDRFRNLVGSAYRPPVGTSSQHLPMGRRRLPDHDLARESTLAETPAEPTA

ANAGNLPHIPVPPTPLAYVPHGSPAAAVMAEVADDVPSTHTSLRSFAGVTPVTPDMLLRV

SCEGSVTSTATTCCSPGHRYRRRHPVSRIDAIVEEAVLLSSCLCPESHFVSPMRVAAHGS

GRERDLSHTRPQPASASQRDTAIGSSCAASQPPLSPSTSRLSGADDAEGRVLGRISVCGA

ASAPSSVVPVGGGGSGFLRTVEAPTTSATDHAAASASQTSEKRQNSGAAMPLTANTAIEI

TPLSVRALVKPLDAHVSSSSSSSFEGSRVARPQRRPLAVQSPANGATPTMRNNAAPINDD

GASVNNSRSRNHQASVIESNANNSTLQPNCPASVPQSVPRNLNHNSCSNTANDDSCNSVV

NHAPSPSSPMDVHGGAVASTEAPLSLFAPACLAVVPKRLTPSPEHDFTRRNGHPPSHATT

PSTVKRAARQENSTSYLNTSHDSIVDEPNMPTERVVPPPSSQPSVPGLTMEALMRQQVLQ

EERKHAILQDRRLLHPPQQFDTRPTANTARELTRSRGRGRNDAGGCHTTEDLLTRITVRD

DTRGWRGKPDSSIVSTLDAPLSAHQLSRFPNV*

>Lp_000058200.1 hypothetical protein, conserved

MFRHLQRISLLLGLCTARWAHVINTVPTNAAEKDVSSSEVVNVAQIVSETVRTTDENNSL

KTPTVDSDDVSVMSKRDNEYIELHKLQYMYSQDGPFIGSRTKDKKVDQVSLRKRRHIVRV

PQPEVEYRPNETSFRRLPKHYILPNEFELRALYPMSTSLKLIEGQADCVDHNAFDVDEDP

APIAYSMVAPPDWKPDVEYPYMVVLPDHRGIPRDFEDVCANFFERPAHREHMLEQRWVII

SPVVNLRHNMQIPVEGVVARFCDWVTDNFCVEHGKVHLFGKGNGGYVALRTVLENKDLVL

SVTAILGRNGSPFRPLDRAQDKVKNFNGVHSLVYVPGLLRKQDWYYKFKFMLDMARVRPP

IRNVHFADVRDHQVYYAINPQEFWNYMKYFRQYNTKMITESGYAV*

>Lp_000058500.1 hypothetical protein, conserved

MKHFRAASQVQARRRGAAVLSHILTSHRCATQAASGSSNNSNSDGGERRGGLDVDAVARP

MGYGPFSATAKARVLREKAESHASNNSGANTATEAVHPSTVMHNVTLHTNLSLRLSLAGL

PHPSQLQEWAYTSPEWRLAFVRLYRTILRLHNKTVAVSLRTSRGAGPAAAVSAVTGVQTT

DGGEEGADGAAAAAAPRPTRADKDDVSHSPAMTTPAEDDDYLLRYLLTPEQQEFGNRFVQ

GEFHRHMDADAVSAAIFYASWYDYVLQLASGVTSRPMTEKEKRLLSEEQKEKLNALRGAF

VDLRMSKEPQYMP*

>Lp_000058600.1 hypothetical protein, conserved

MSHSFIDNDQGYDASPKVYVNNMNTRHATVGLPNRRPKNNNNKDTWLLLRGNDGLTDTAE

SVGGGRYAESRLTSDRKPSTSNGGAQVAWNRADVPTTARGEAVHPNLQGVYNRIMRRLQR

QMNYAELPRTSLAEQYGVDLELQCEAEEAAAAAAAAATTTSQQGDGGYREGHGAGPPRNN

SISDNNNGSSSSATAAGPLSALSSTQVPLALADTELDALGEDTQLNSKSLSQPASRSRRS

NNSNNSRGGGAAATTAGVGAGVTELNGMPSKDGGPHGGGGGGGGAKGTSRLLQFKQRPLP

TSGGAGDAFENGDGTTTSGTSCGGARSDADVEREIAEEPCPRPLRLVVQHVIDTLEKKMT

LAESGSLRDCIVFSFESRALLDRLLREIPLYTQYTAFENKGGTGSNGVTQAVSGVYQQLR

KRPVLLPRGEYVEDATTGAIRAELVSDTNGGFNHSHEDVSSSSAAAATHLPSINVLPTPS

VIPCASNADYNGDAQTTQLPEVVLPVGYGKSSRLTASAARNNANRNASPLSANSRSNVLG

VRGGSATTTGGGSDGSGPLQHQNSFHYSILRDHLSAANASVPLAADSHGDVSEFTPSECA

ERQPRHHGGGGSAGEANAPFTAEAAAATAAAHRATQLVSVGTITEENGVTTVPQAEYAAL

KQQMQELQTQLADAQQHRSALAEQLCEEAQYTDQKKRIIQYLRETLVRECNMLRTQLRHA

AGATAAAAATTTTASPAPGKSCLYTSRSALGSGVSNAVFFKEASSALGGLNASTHSYARM

HKQSNGAAPAASTLMGTPTLQQQQQPLPKITFDHPDESESLGSVYGGNYGVTVSGAGNGG

GGDGGRPSPQLHHSGVGNNNNNNLSIGGGVHGSLGFSTSVWKVEAVESLLDLALLAVEEE

AVLPPHAAQQLQSGHGDKELLRSGFRKNAKQQLDELRVDFEEREQALKKALLQQTAEHNY

VVAELQAEVKRLRALTDTTYVRNTLQAGVSQIRAELTRVRMHVAEQLHFFRAVLHSSGQG

LLHRAALVDSTMSDNVALTSTLNALKESIESANTLFLPMLTREYECGYHPWPLKERNTRD

PLGHIVQLRFGSAEVVRLRDSLTEFSKLYVAVHQYVMGHAVLPESARPTTGRPLEQLCAA

LALNPTSHTDVVFAARRCHDVEGQLRRKLARLQSRILWNAYQQRTYRERSMAALTEAGID

PRVMTLPVARCIDVLAQERGDLLQARVKVQRERSENAKELYRLWREKGIDIMEGYPTPQT

QRNRLALLSSSSNAAAGEEDGVAIKRTLRTPRFSLALKQGQETDGIGAAGSINF*

>Lp_000058700.1 hypothetical protein, conserved

MDAFRQRRASAPSQFFTPPRSQSILTHPQPASPHPYASNNSGATSLTNSYDARQAASPAR

PSPWRSSASSAQQRSRSACVTSADVTFTSALQQQYETTRLLSRLFAGVTLQWRQVVEQLN

EAGVPRTAENVVKGTPASFAPPSQPSTNADNNNSNDNNSSTRSYLREGLHFDVQALNARR

ERERQHKSLRQERVQRRRTLQHDQTQPYSRRADFADDAIYEPAAEQQLDHYLQAEEDEVE

ELERQAERAAAATTGGADWSSLTPSAEWVPYVVGKSRADQSDETWFADRYDGVLYASPDV

FLKQQQQDRYQSAWSSRTRCTLCDDEETKMTIDLDDEARLRTAVLHGMARSAEKMVMGMR

QVQQILPASAAALRARTPSRAATPQQQRTLTPHAPSAYSVVCPAGMTEVEDKRTPTRRHW

RHGKDELSQLARTQPAKSAEVPLQRRRQPHQHAASPLSRSPLNLSPPPPQQQQPSQLASP

KSHLRRSLSAGDTAEVTHYKTGKTIYPALPAAQRTWIYPRLDDSANNNNNSSGSGNNESS

KVSSTRHKSLLHERHDHRDQHGHQSTPPPSETHRAQSTSKSTTCTLALHGVTPIAESSSV

GVSITTDVHERSREKRVAAAAAAAVNDTLERRRCRASPPQLQVQRQLDWRKYSSGQQAAQ

TARDETKGFFWSEDGSSSRHGERGGRRGSQEPPLIPMGRALSEVQQAHLSALVHAETEAR

QRIAVYHESSMNSILQAHYRQLWSLRDGGGGDTAYTSKTGAEKEERVCTTQNEGGAAPSS

DAAAPPVGTTSATAATLSMPAVSVSPLTSPPASPQSAPVGADVSVNNMCRDEIRTEPEDV

ATAADNPLCSPAAEPTPVTAASDPAQSLAATQSDSDSVFSVHSPDINEIELSGPPSRSAS

GQHGAPASPSPPPSTSSKRTSPLYELPASPMQQVARSPSPPPPPEERTPPPPKQSADPAI

TAAAAARISPRPAVARVLMSPLDERASQVGKRCAQQWDIRIPPGDALEPKKTNSANSSTF

LAAAETAGTAGTAGTTAHDHAWASVQKYVQHGGSSALCPSAVSPVVPRSGGRRVAEWFPA

LTKSATSTVPPSEPHVAAAENEHRGSVVALELFPPKSLPKESPSPQRERQKQLPLLSPLP

VIPPLPPTFSAAKTAKECTNEHELSLYNRGEEREPGHENVIDQSVHSKERGRNAAAAAAL

PASASKSTRNDSSTSSAEGSSYAAHRSSSHSSSSCSNNNTSQLNTRTSSSIRYEAESASP

LAPDVEDVEEVTNYSSISSFCNSLPECLAAVETAGTGTSSSTELNNTDSASTKNKKGAVP

SDLPMVLLLGAAPAFEHIIPTETCRDYLLKVEVPSPPPRPPMP*

>Lp_000058800.1 hypothetical protein, conserved

MSSTNTAAAEVRAPTDEVQHPPTESAPAPRADAQKEKKRPAAASGAVSAAEKPATSKSGA

ETKHASEAPPRQEQEAVVPTGEPAAATPNRSPAMPATAMTEHKKRVAFTHPASASARSVP

PSPQQQPSPSSSSQACHTTSASVGASRPVLSASPQRVDLSPPGSLAADPGHAANTDATEA

YTCAEAERIAAAAAALTPNTVLCLFRRGGDNSHTNRMEVGSYSRYLLSTDHMPAMVLSET

FHGEQPTLGTAPRNTVKPLVEETEDVEAEAEASAERPPNGTHGNTESRLRFSSSSMTNAA

AAAPVTGSAEAAQMSTTWNNTAGSGTLKREVVMLDAATFAGLDEVRQRRRHDPRKGQPGQ

PQLDYVYYNHKSKGYYVPHDPSPETVMLEYNHMYSFGDGTRFAPLTTSPTHSQQQQREAE

ADGKGTYRCNPHAAPPRTFAESRGCVFALTASQRQQPRSKSSSSQHSAIYAPKRVSEAGA

AQQQRRPQRTDIRNGTRSLRTAKAIDLNRLHRTQCPRPRTIEEVLADQHRNYLYSGGAPQ

PLVQDIRRDVSSGKLNWEEVPASLQTALTAAATSKRTAFATSQKNDISSSNNHQQQAKLR

TASLSQPLNGLVDVMEHLRPVPAIVAAMATRSTNGTSAAAQETAVEEQPCSTFEGLHNGS

NKSGKSGAAMAPAALQQLPAISRGNNAK*

>Lp_000058900.1 hypothetical protein, conserved

MSANNDPRSPCKRDDISIAFTQSHYPTPYTDHAIPGSTPGHRLLRLGRQYQPEDILYNHL

SHALIAHGDGSNSTATPWEGNADHPNPPGGNYILHTKNGGKRHYDEPSGRFSEQPWQPSK

RMIAPLQEEERCVVPQRRGRGVYNTDDGYIHPDAEEARRQREWAEQRRREHGGKARVDGP

PDHTLTELGLVQQQSMPPVTASTGIRASSSFHVAAAAADATSDTGLPKEKQDATTSSKNA

RAAVVDEDAGRNKAYNGRALLPSVGAGVPPARGTAVEADAVAVHHRDQRMRCALHERNKA

AAATATRAADAQSVRELPNW*

>Lp_000059000.1 mitochondrial processing peptidase alpha subunit, putative

MFRRAVASSTPAVAATAGAVATCTTQTRGIYQYKFGQTPLTQPFGSGSRMPPSPASTAPA

PAVPKVEVTKLHNGVRVISHNLGGPTVSVGAYILAGPVYDPPSAPGTGAMMHLALTLSNY

NNALFQLDRNIRSVGAAESHFEKNKHYIGIRIDARADKWKSAATAAPRSDRRLANQKPAE

ERFSLNLVQENIFTCIAAPRFHEPDVERFRDTIDNQVEELRWQYPAEYAKQMLETVAFYR

EPLGNPRFVPALSNGAITSSVLLDQYSRYVVPSRVVVAGVNVDHAALVAEYENTPFPHSA

SAPHHARAPAAAAKVDVQKEADQYTGGERQDHEERAKVMGTKPDMDPETICAVGWLAFGK

DRKTLRDYAASMVVKAILDVEFNDSIRYARDEMHERAGVRAFYSPYETAGLIGFTATAEP

NTAVRMVTDAVKAVQSHKASVAESLLSVAKSMAKTQFLVQNTDTIRDYCDYLGTSLSIEN

SDSTVATSVAEVVDAVNAVTAADVKKVFQTMFSHKASLYGHGEILGFPSMRQMGI*

>Lp_000059100.1 Galactosyltransferase, putative

MVTDRRPTTKKKLGEGGNWGLPVEVGMSQKLILWLEYAYHAFRDGPYIMKGDDDTYLKVP

QFLRDIRYIRGGFRDDRMEAPLGQRDPVGINETEDCLYWGSRWRSIEFDGFPYHEGSGYM

LHRRIVQAITEETNRTNAYMLYLAARPFKERLTDTYIRSIMHIEDAFMGRMILEKRRRLR

EICPLRRGSYVEEPKRRQCDLYNQYHHNVTWASLLLHHVSPFDQHYLHYFFQHEHEVAAQ

AGGYNATAEEAANRAAAQWMASHVSSSLYGSNRTVPLHWTHPVKDISSFTAAQDKVRVYG

FKYQRQSLDALDVAYVSLVP*

>Lp_000059300.1 hypothetical protein, conserved

MDTLPAQRPNDLVRQSPPSVVTKSSTPQRKRQRSSTPPNALEASSAPSDVPPGESEPSRE

DAEWIAVKDRLAVAQWRRGCLRLPFDETPFLKDPPNLPTKMLPLSGDLSNQLTREGRVSS

NADLSTSYICPNPWKALRVMEDRGVADTTAAPPKPNRSGFNTLSEEPPTPSEPPTLVHPA

KWNAYWEMYSTCCAEALEDLAVSLKAPQAGYRRVLLTLISTAVVVPLPSDDTTGILQPTL

PTGVLDGAMKATGHIVGVSTCSSGSSSLTSPSASCKVEGSNHVETQKDTASESTISPPSV

LTSPSHSRATSPPASNNTREKEGSASASHSRASSYSSYSSYSSYSSYQSSANGSSSCSSS

ESSDEVTMTFPALATVLDDLLYAHAHYFGAVESIETRHSTTGASGRVTTFVSMQFTTDEA

AGLFYRWVDGLSVEHLVRSYWRERRILRRQTQCSPFTARETSAAGAILRISSQSADGSPA

FPPEEKDLSKSTKLQTREEREDAWWDAFAARVADGKLELVAKLAPHDARCNTGKLLLGPN

VMISTPLVTSLFTGLFCATQVEYDRTLRGFLINFSDADECRLTLHALQGSLKTVFGVALS

FR*

>Lp_000059500.1 Calponin homology (CH) domain/Domain of Unknown Function (DUF1042)/CAMSAP CH domain containing protein, putative

MATVPFSEEDLHELYTWVDEIPISRPKRNIARDFADGCSVAEILKFFFPKLVDLHNYVPA

MSHAKKLDNWNTLNAKVLRKLYFEVPPEEIEDIVSAVPGAIERFLRALRMKVAQIKQRRE

EMRASGELRAYEQEHFVDPPLSAHGSIGGSRGTSARAAAVAERRPSSPGSRGASQVVSAP

AAVVLPQSISPPQQQQANEEVKNLNHLMREKDYTIQELRETVSILSEKIVKLEELVRVKD

EKLTQYREKFGRTPA*

>Lp_000059600.1 hypothetical protein, conserved

MAEPSWKEQVKSEVLQGLGGQIGSSPSPRPSATAPARCDEQWGDDDDPAAGRDAMDVGEP

APSHLLPSMPAVLSTAATFAAAAATSPVIPITNVPKSPASVASSLATDTPRSILRHPGQE

RGAPSPRSRPRRISFVDLSPLRDSNSSNSSDQPHRKRRESLSVEENNEDDEYDFAEPLLR

YETETPPVSVRNAAQPAIDDEDDDDARMRGNASTPRSSSSSPSRSPDRRGSAAAVTKTQR

GRSPDKPEAVACEMRRSPSSSPERPIEVRRSPTRSPPRKGDQTSPRRATRTAISAVAASA

PPPPEEENDVVTLDRSSPPRLQPQQQQQQNYRSPNRACQTAEKKSPAREASAGHDEDILT

QPHQHGRSAPHGMTATPGGRRPSPRRRWPSDAYVDEPPTEVSTTQRVPRGGGRRESLDPE

DDMGSTTSRSSSGDTSHDEYSSDDGPSFHYESPVKAAGTPRRTSSGSGANASADYPRSPS

PTASSLSRSPLRHGRARDQTKSPIQSQPRSPSSRLPERRPSKSVSGSRASSVVSGHHSSG

HSSNRERPIPSTPQSLTEGLDSGMLRHRRSPSREERTASSLQNSTLLPLPVDDSCATKRG

DAATTMTATSSGEVSHSGSHGRWPSAITNDGSDRSVEEGVAPQMPPSLALAGPAAVAVKE

VAKRKKKVERDEPQVYVDPLLRERQHHHSVARQQSPTHRATRAPLSGDVMPLLSERETAV

DAPRARKRHSPRHVGVKSRVEGGGGGGGAFEPLSLRDIMARHDSPAATPKRPLSSRQLSH

TRTSSSSSAAASLSSTRKKAEAEETKSESSRLSSGGVPAEELVWDAPTPSSRASSASPPA

SHGSLASAVTPGGHRVFRKKLVTVVRRRRSGAPPSEDDPVVRMSIVPYREGSPAVLEAEA

RQEALRHDREAPTGGSRSNSVSSRDKDARTASRSTMGAVSATTAVGAPPLERLIDHIHPG

RASSVSTRRSSRNTHIREDRAGEREEDDGGAEETDVVTEEDRRQFRHTLSTLSSLCASRR

SSAARSRRSGSAMPAVEPPHSTKEEEEDNHLHRHHHRHRRSSSTTHTTTTNHSEGKTTSR

RSSVNAAAQLSISKQKTPQPSSPSVSRHTPKPESTVTVAPPSVAATLVAGKEGPGDTASS

RKDESTMTRARHHHHHRHPSTRRESTHATSKAPQRSGSTKLAGAASSPRSLSAEQAVPPA

PTAASAAPTSSLPTLPDAPYRPTQNDEFYYGADTLANEVAQLRQRDASQRIGITPVVMPL

LPRLTFAADQTGGGPRDNADAQGVAAWSTSDNDEDGDENACRATGDPQIADGGASYDGSH

RRRRLLLGSATQTDRFGPVTAGSRGVGRMPFTAAAAPVLRPSFGTNGGVVSNTVPAQAVT

VTDAPLTVAASAPVSALQQERSTVPGVGEPTVAVNDRRESATVATPAEGESESPMSFVKR

KTTPAAKASARRGAAPATANVGFAGAPPVEVANQLLQRASAPLMFGQPPCPSQVMTTSPS

PYAAAANNNMLGGASPPPETASIGVPLFVPSLTVDNVSHLTQDWQSTLRERAGKAKQSAM

EQFLIASAEAGAASTLSGTHPNHNSVNNGNRSGSGGPADGAGVPPDWEARRWSAAPVQPG

LPASEYYGQPQPLAATTATVPLAAHALPPRLLPQAFWEAEREARQQAAAARVAASSKRKA

SVSFKPAASSPTTKNETQRQSTSLSPQAEALDLVEAGDADERSSRGSNTPEKADSVEPLS

PPRSLATRRLTPPTAAALASEQSPFSQRRHHSHSDRDVSALLSPLADPVKSTDRAARQKK

SKADLRTAATAAAIATATQGRHDSDTRGTAGFTIAEPEPSKSKRRDKHDDGNRHPRHGSA

HPTRRRGRSRSVCASSAASHHTEKHMKDTSRKAKEVEEMERKKRKGKEEKAASRHKSSRG

KRRHTNSHRRTPSTTASSTTSSSAASSNLPFSITDMYSFPHRMPAVDADASDVDSSMATS

MEFQLSRLRLQREKEVSLALASLQEEQEKAEARRQRKAEKVRAAAAAAAAEKRRGSRLHR

HRTSSSGGRSPKNSESDDESSRSRSSGEKKEKRRKRKEKEKRKEQEERKKKHKENKRRRS

LSAATKKSKKSEKSKSASKHGPSLLSPPPPLSREREAWGDGGFTSPYDRPVPESAESRFS

SPPYESIGASRYNTVSSPYTRQDYAANTPFFGAGSDAESRWSHFRSRSNGIDNNNNTGAS

RYTAASPYSNYRRVHRDYFDNNDNSDGDAAGSSRYARRRSYTSLSASRWADTPRYFSDGD

VSDHLGTSPAASSAYEPSARKYSHVVRPSTRWADYVREAKRQDQGATSAAAFSAASFAGA

SRYTSFGQQRSQSSGGDMANAWHSPTSGARYSSSYARHRTDDYDDIDDIPKRSYFNDEGT

QPRFTSHTYTTSATTGGRRRQNTSSTTDNVPPSSYRRQRRGTANATTQKEEDILVSTPSQ

DKDEGDGDSSYDYWEDQGSRQPQRRRGAPPPPPTPPGESPAGSTTRTPSTSDQDFMRVAH

DARGSVFASSKALPIVTEHAVTNAQAAHDFAEGVRSVVGALRHYKEQSR*

>Lp_000059700.1 Dihydrouridine synthase (Dus), putative

MGDIEWETLEKAWAAEFQPVPRKTPLPALIAEADRAVAAAVKSLTKEYESVIGSNASPPP

LSAPLSSSPLPSGTGASLGYGSENTSAVSNVDRDGATEGLVDWPTLYQSLCLIQAPMVRC

SRPAFRQVCRLWGTKVSYTHMIIADSFVKSPHARHAEFALYAGEDRLVVQLAAKSGPAAA

QAAALVHPYCDAVDLNCGCPQRWAMKEGIGAALLEKPELVADMVRSIRNATPEGNGDTPP

LPCVVKMRVKDDLRHSVDFARQCEAAGAAWVTVHGRTPHCHPSAAVQFEAVRHVREALAI

PVVLNGGVTNVSGAMAAALRTGCGGLMAANGLLDNPAMFYCNASAAMAEAETTFGVPTLR

WARSTETREAASCDVSISSSSSSSFAPLRPVSFEWAPNRAANAINSGVAFPPASSVVFRY

TVPTMWEAVVTPREAISDFVRCAIRTDLAVPTTVQQVLRMARMYVSPAERNHLALLRSNL

SLLNCLQDIGVYVEEGHIGCEC*

>Lp_000059800.1 hypothetical protein, conserved

MNYQVFSYMPNPSVATADPPPRPIRVTPSALSPLLYAILYSIEFQAPVGPRPATLRDHSA

GRRSSSASGSGGGSGGSAAAPSATALLTSAVSVMSSFWGRTSTNAAATTPSSTPQQQPQK

TAPAFGAAGWSAIAGANPTATSLSPPQALTLAEEAELAALQRQHEQPFVVPVTQVLHKLG

GIHITVRETHNPTITTAYARAMEEVETMVAEHEQRLARQSLRSVSFPSQPGGTPTAAATS

SASQSVVALIQSAGDTTTTTNAATGHDAAPTRDDTTTERYGRAGTALGAAVTAAAGEGEK

QPLVPPVEMSRLSMSPASPTSMLRMGSPASGGATAAWAAANNNGNSSNAYYPSRIDEYTP

GPDEVGLVLSVRLLSKEVTRWRITHERPLAEWRFPIIRTPSERMVIRPSAQSPVFSSSVA

SNAGGSASQDLREAVNEGGVAGHDYILVNDAAQMHQVLEFILKSSYQTTSMETFTDFTSG

ELIFDAHVQERRRFL*

>Lp_000060100.1 hypothetical protein, conserved

MSYTTPTNSILNRDESDTARVSQVIYENESLKIQVETLQEKLRSTGLLDVDQLRQENESL

HSEVATLRVQLDEKTAQLEHRKSSDDRFSNIDQSLHELLMQIQTQRTEMAGMKDMTREKD

ARIAELTEQLKHAGEELSEAHAQLTAASADALQQTAQGAEANALREQVAQLNTEVETLRQ

AAEEEAAGLRKNLAAAQSELLALKADAAAKSSVKREHMTSQEKEVAECREREASSQRQVV

EMEARVELLRTECSQLKETQGQLLRKVQEQQQQLQQQAASAAAAANRKVDLYCPEVKAHV

EKAVQNATDGVQRRLREQVEQNASLLSRMALIQQESEAMLKSKGMSNHEVTVMKERVDMR

SRRNDARHTMQRRVAELLSNEELNKEDIELLLQEMLDYQEEQDQENRTMLVVKDIEAEER

ERVLRRELKRVKDENTSLMKQLQQVAMEGFHRGRSQSLSGSTGGAATIAAAAAAAASPRE

AELPSATSQACTGNVPSPAAATAADGAAASPPIAVLRNPLTEHANATPEPNQEQAAERFG

GGAGATADTPSRPVPSAGSSSPQQPPPPLHQQRPLQQFGGDLWSNMPSRAGLQHAQPQYT

RHTAVHADPQLPPPPQSLNTLGSGGGTSPEANRNVMVHCPACTYRQRYGNRNCEICDAVL

QLN*

>Lp_000060200.1 hypothetical protein, conserved

MQPRHPPSTGNPAHAAATGPTATSPFISLAAQCDLLYCVGSQPHHYAVISEQSVFTRAAE

QLRFLTPSPLTRHRASRSSPADLADAHDSPEASPRVCMVASPPPPFPSIRWFELSREAEE

MTQQQQKQQTQPQPPQSSLPLSLLSTSSGATVPLRRQLVPSGTGLATCAFQNDYPANKSS

LSQEQSGEGDMTALRGEQGGIVASSAAAAAEAAVAKLEGSAEASAAASTIVTFFPSAVLV

RAVAQMVLTMQASKQNSAAVFSLARYCGASLPAWVRAAHQDPALAAKAADAAFHLRDTLV

FSVAPQVVADHLAALQVSTTPQAGADAAPSTTAAAAVAAATVFYAVIPNVDTPLTSVSAT

AGKKRQRSEAAEGSAVEPLVMPGTVSCSRISGTSPSSSACMPISYAAMTPSTFQGLHQAS

RSALHPAAGASPPSPADSTLYDDEAEWLTHGVLRRLFTLPASPIAGPASIASERAETEES

RRGTYAAFLKTNAFLLLSFLVHYSWCLHIHASVAEALAARLAAQRTAPEAAVNDGSFVAI

HIVCLPQLADVRRSPQHAWTSELTYRLRRAKQRGVSASAKTWAWQLTANVLNRNAELQWR

LFGRLGGGACDALPDTTVEGSGTLDDLLKQL*

>Lp_000060400.1 hypothetical protein, conserved

MPSGSSCYRALRLNFACKDASFYLLNDQREAACQLSILQSCRHASSVSEAADKQAKESSS

AADGTASGGGGAASHKPSLDAEAMEAKRQAARDAIIQEIISRDKVIFELKRQHELSMLRV

EQNQARVLKDQEDRGMYYEQNSNVHTFDTISVGLYSQRSTLYHTMSAERLRNLKIFLMVF

TTFATCAYFYYRYIINPDWEYVERPMKMIGSRVQAVREQRWKLLSNDQKFDEMRKQDKEF

ASQHE*

>Lp_000060500.1 hypothetical protein, conserved

MKENGKPPAGPGDKSAPPLRSTTDLRSSAANRASPGVSSSFAVTAASSGVGLDSIRLNTY

HDSSDLKAALRELEAAFATPGAATTSTHASSLETKGVAHTGDRNSRAMNTTPKTSISPSP

VSPNVTVIVEPRAAASVSSPPPPPSRTSASAPSAFIQLPPQLLLRCFAFCDLKTLGVLSS

VSVRLNVIVEQQGSSLWAAAALRRRVPVANAAAARLELRRALEQRARARHAEEEFYETEI

ARMEERLRARAEDVYAQNVDVERIIASGGDSCGGGAPPYWLRRQRTGEPGLADRGSGTSG

NAATIADKNSISAQLVTRLRAEVETLEEVKRLCECKMSLQEASLSQQEAQLQRWQALLSP

GEATTRATSSVNGPSEKDASSPLITAAQLEQFERRVTRLVLSGARTTTSAAAADEDERAD

IPVVFRRGVEDFAGLELVLHALNAHAGVEGAPSSAEDAPGSRALPSAAARDAGKRWRAFQ

QVCPVNEEYENARFYLKSQELRATLPSAAGAPQSSGDSGARPSAKQTPALLRLSGFVRRV

AAMTDSQVVQSWM*

>Lp_000060600.1 inositol 5-phosphatase-like protein

MVTKPPPEAQEHLTAAKAAEKASTSTVPLWARAVVVLVEVVVWALTLGFVKRITALEHAL

GYSPQWHERDRAKESIKGALRSGMGAARRLAQPLLRIETEFSQDSGSDNAGVLTPHTATQ

YEETLNTRLQVDAHIRTFLRDVSLEELEAPLLVNACTWNVDQQPPPVHEESFKTWLLGQE

LTEELKHYQQHRESVMAHGGTVPTPFSHNATNAMRARKMTMVAGASGNVSLSHNTSSSMS

EPERQASDDPLLTEWQWLESKFPDLFLISLQEVEMTGTALVRESTQRRWEWTDAIIETLH

AASNRMIEYKKVQVVQLVGLVLIVLVQARHVDYVSHVRLSLTRTGALSMLGNKGSVAMRA

TIYGKRFLFISAHFAAHTYNEKKRTNNYQAALKDIRFDMPAWSDDESEVLQTFINAAKTS

DQAVENFSVVGGSAWDRLFRFRSTAFQPSFSTAAETRVLDNHDYVFFMGDLNSRLHALPG

SEIRESVAQGEYDYLLCHDELRQLMVSGEAFDGFQEQWISFAPTYKFDRGSDTYDTSRKH

RDPAWCDRVLFRVLESDDVATLKEGEGDVRKDTTADGAGRSSPSSSPSPSPHSRSSMSHG

SGTGTAVFTLPREPSVGGGDWVSLEDLQRTALSQQVEEDVANLSSSASSDFDGDGGTEAT

RRQLNSEKCTAAMAAVAAQNGFDSPSSLRHKNQEFPSVPTRATPHAVWEHKHCAPTFCAA

RRPRGPEPSLNPFAIRFPMVTNHVNALEYTHVPALRQSDHRPVRARFEVKVVALGPSTVG

EIVESVRKVIER*

>Lp_000060700.1 hypothetical protein, conserved

MPNSPAARETAASEFAADKEAHITSLILQKHEAAKRIEALEAEVANLRSRCQQSATSQVL

WGADGAVQPSQAKERAVAQNAHMTEISTWNEELIHTIEKLQKALIRLEADSKGQIITLTD

KVNQLHEENVRLLNENDSLKSRVGDDVLRQRQTNRIEMAFERARIAARVRAAGLQRLEAA

EASLMEAHDDMYYVQRWDALLLPQRDVSFVFHYVSAMTFPLDGIEEFIESVYQQFLFHSS

SCCHGYRVGFHKGMEVFAFQSPSDALLFAKESHEQLVGLSWPSRAESMPCFSSIIENNTV

LFKGPRIHTCIYACSPESYVDPVSGKYSFFGPEVLEAAQAAIEQAPIGEIAVNERWAQLM

CKQSRLRDDRAVPAQGEVTDLRELLGSQWDVVGLPGAHHIVASLLPVQLKSRRGVQPSVL

HPSRKYPCLELKDAAEEARMIVKAMKGALSQTVEQPEDAGKGINGGGLIMQQIRRRANFL

QEAVLKSPAQAVGTEKNAGFSESAAQLLELFSLQQEKQNIIALYHSTEKAFAALERDTME

SEDRFELSKHKTLDASETAYVCTIDTGDDDIWKRLLLKSISDEQFDSIRSTIRAHIHDAA

KVHFGFLMNGNYSDVFTYVFREAEQALAFVSGIYIMVNRTGTKYAHTSLGKGRDIFLFRA

GVACGPMSTIYRNLENGVLKCTGPAIRLSGTLCDLAESGEILAMEDVIRNFCSKNENLLD

AQYNIVKQGAQFLGSSDAPAVVHSILPRPFAYRRPQLRISGRGPMQREKIHLPYRSVLAT

LRLRQDELPRQSVLDMMQQQQLRLQYAESAHMSAEDEYEQSWEVGSGCATFLRNPWVLLC

QPQAEEQTGATPAARRTLVRFKTTEELEADLMHATRPVSTLAFLYIDVANINAITRAVEP

ALLKRVWDHYNYIVQSTLRAFDGFVAKTNSVTAYLVVFEEARMALEAARQLQLELVQTRW

PAELRSLEATLQVKDAKTSAVLFNGPRAQMAVHVSDQYTWRPVPGARANAKNDSSGVATV

DANASDLERPDLSAVHISGVGVDEAFILGRHAHGGEIRLSRPLLEAVGKHPSGKLLLEQL

SMEVVVAPSVIRLAEEAASPRAGSADGAAAEKADKSKSVQTNMFSEECVASVPRRLQGRL

ALLRPPGTSDETGGVFKAPEEFPPTSAAAAAGNRGGSSGIMRRPSAVPIAKGRANSARVG

ADAMEEQVITSIATGGTSATPGGASSALAKVTPLLSTLSPQNSWIKDPHESSNPLPAASA

TNWGSDEEQASACLIAAGVHNEAKQIQRMMQAVLKLFPSTPRRSLSLSLEGALEGSTSEF

PTMTSTNSPLVLLFEGDNNSVNNEADDVSGPRPDSRSPTPSSDARNGTLRHRSSKEDTVG

SVPVSKATGGGKRSTAGAAAASRRSCVASSALGQYTRFLDFSRYFVSMLVNALEIGTELR

TPPPLALPSSSGTGGRTGSVQGVGGSSAFRYGRNSSALVGGRAKSSQSGGFSLPPVPSPP

GRLNGKAIGTASVSAGSNQLLAGGKDNEAMVVKAAAVNRDKPFQSALDYIDDACRSLVQA

CGMDVGRLAAIPAAPPSKPKAFSGRLTSRRH*

>Lp_000060900.1 hypothetical protein

MLSWLWDALFGEDVDECDDMVGVSMAGLQTTADEMRTLQDTISAAKESLAATEKNARLFY

QSIEVLIMETYDVAGDNAETSSDTMELHEMRVALKEQRSTVMAAMLKQKDAVEVRLFRAQ

VQLRTLEKQKRMIEASARQLDHPSLHTGHVTFPTPVTSEFCVPGEAKK*

>Lp_000061000.1 hypothetical protein, conserved

MDAQEISKFIEELSVFLPLSTEQGSDNKAVDAELLNALADYIEGADPAESSLEKLRLSLH

NASDHEQQSLEERMRTAGSRRSLKLLREALQRM*

>Lp_000061100.1 PTP1-interacting protein, 39 kDa, putative

MRVSRRFRQKAAAATEQTLRSVVAVGLRQEFEPQWPLPLPEPLPMHTNKHTLVLDIDETL

IHTYGMSKHDSARDTSPDPAVKGLPLVDYYVMLRPHVKEFLTEVHRLFEVVFWTAGTASY

CCAVLDALEQQVMGLQRSFNSHIELARELQKKNSSTSHTNFYALSRTQTLEKQGYMKYLP

MLGRKMSRVVMLDDNVRSFPLTPRNGIRISSFEPDDGILQRYMFAIRRLQTEKPKDLEPA

MLECIQLGQQEIARLEQDRGLLDVLPVLRAVAQVETNGDVTKELDHWRDSEYVRCDDFME

TMNHRSNVRRDILGATLPERRATAIPPLRNGPMNNGFLESANAEMRMHHARPVPSKL*

>Lp_000061300.1 Protein of unknown function (DUF3595), putative

MAAYCNIVSHVYLFVVLHFLFLLVVLEQHVMALALLVAFLGTLSFPAMQEQPGLVVQPPA

YEEAPRTETDAPEQRGGWGSREPSFTSVEDHRGAKELTITASNAAVVRRHHNAVQRERGH

RSDCAMAVGLHHGTARADKLSCTPPHYSLACEAEEEIVVVPTAMGVGERGSGCRSYALVE

SSSTAPRAARVTTAGTDGSTDDSSLGHLKESVSSALPPSAPHGGWVWATAVVSLFSVCAS

VILWLVISRCSSRGSGATDCAAPPWTYRTPQRTLLSLFAGIQVERHGVRQADFFFTVVTF

AVLGSLSIGHALCAALLRCRRSALPLAGCHLEQFACTVGAWLVNGFTFAPFSLLWVFIGG

VGQGTAFGCLYVLLALLMVLSYPRCVLSWRRRCWLLYLIFLLLLWTLQACLRLAWIRESL

ETYTAETPLDQHRRRLFILWGLLPATATTASADMRWRVVLAVGVWWAGVECFCFHTAVYV

TCTAVPRMACIREAAKYRSLYKHALVCKEKIQLARALALQRAAHRKVEELRSERETAIIA

ATANATTAAAAAREMAEGRTAHDALGYNTAINDDTQANAAGATNSRQHAPFGSCESLHCS

MDRSRSAMRATRVLQGNSCNLSPVLGRSLDRQSSAPMSHVNSNQSSSSCCESDNSCSPHA

PLIPRVAASEARRTKRRASWRNLTNNPTTWAQDCDNFIEYESSPTCPSIKAESLRTTRGD

VTTHELQDRSDFDAVRFNASRLWAREPTRSPARAEDDGDDNENRLVHLLNASPRSTASSS

FSGDVKLHRATTSTASVPPSCSIAASAAPAQRLQVYCASSNRLPSDARRCPAAAPTHNAH

GVAHGVSPFHSMSSTRLIVGCNSPVEDGFSDLGWTTPPAVETTATMFAVATREASTRAAQ

PCKADRDDDKAAASASTSLVRGAEKDEENPSTGITHARGFMYRAFRYIQKELRAYLTAHT

LPVEEVNRTRRKEVNADVAIQNESGPVCRKDGNVTGVVETSQTPASVNSSALPFEGHQAS

LSITRLLVCYVLQHWVWVCALLSLLHFTVASTAINLAPSLASLVYALLLRPWPPRWHARA

GAVYAAGSVLGKCLLRALIISGVVSDVSSMAARSVDALLLCVRLQSSSSPSSVTLSVSSW

VSWFDLVMGVAALCAAMLQWTLVYADECALGGGRVDADHNLNEQQQPSPPAPLAAGCSPQ

ASMAETAAAANNPQHIGRLSRWNRHRRGAGADYYTIQLSFDVVSLMLFCWAYYAIAPGDT

ALSQDNLLYAVQHNRLPGIFVATALGLVVLLIVERILYVLHALLAKYVLHFCLALVYHAL

YLAWRVVRHADNANSHGGGGTTRASAMPVALLMAAKLASLWCGTLQLRHGYPLHRTHDPF

TIKTDVFHWFGHMAFRAVPFLLELRVLLDWSFSATTLKVQHWMLLEDIHHNVYRRYVDMH

DLHWTSRHQGRRFPYYVRLYQGMLSLAAILLVLFFPLFWYSTFGPQVHASAVTAWTSEVA

FRSFSVLPFFTADASLTQTAFHTAATTPVGDTTPSPADLLRFASAKDTWQFVQPSQCSSQ

VWAYTPAAMTQLITELRLHNLRRQPSQRAQLVVRSLVTRSRATETSYTTCSIEESYTLTP

SAATTLVRVLEAWQAADRTNCSRGSSAALTPTAVPLPSFYTPYVVSSGSSVVPMAGSGVA

KVDCTLTLHRMGPYRGFTCLECAPSMISSAALLSSSFSAASSTEDSLHRERSLVYVVAST

DVTTMENTLSLIPNVGVVALYTSFVLVMSTYIRNFFAGDAHRVVLLQLANPEPVAELLRY

LYLTRSSACNGEAGDLFLEQLLFLELLDLLRSPERLLALGGRRVDDYARREYRADLYAVT

KRPFDLRRR*

>Lp_000061400.1 hypothetical protein, conserved

MWAFEAYAEGLVTAAAVEPEAAEKPPRQLHKTSRNNDIFDVDGKPLAASKRHEATTWRTH

HSFSGRCALVKPAAYDGWSGEANGATPTCPPYPSGPSWAEQAWAAYQSWSVTFSLHSATA

DEPHILLSVAPAYFPLLSSPTGETLTPDAKTLASLPAAPSAQEQKHYNNRLLDTPLIWEE

AYALSVECTPRGLFLWSDHWELYGKKILDRCLEVQKGAPRPAGDAVVDFSVNQPLRSYSG

AVRLRFDAADVERGAEEGADSHPAVLAVDIQTPASQVSLAASTTKGAEEVQWQHVVDVPL

PMDALARKAAFRPHVTLLESGDSVAII*

>Lp_000061500.1 hypothetical protein, conserved

MGGCFSSRRHSTNTIGASIAPKTQDKVGAADTSVGSRAAAAATAAVASAEDLKARARRRA

LRALVESLQHGESVIPNSKWRGQFLKTNAVGPAVISTIANYMQRKTGPPTAGAAETTASA

TKPGRAKHSIRTLDLRALRAGDDGFVEMMLALLDDTLVENVIFAGNEITDDGVQRLIKHI

EARTGSAANASAKASLPCQLKFIGLTDNLISSSGIATLATIAPVFRTLEQLEVGRGLSGG

GEDVDEVRDTLSLTDVKAISSYIQDTPTLVTFLYQGTGNYYARSGFSPDGFSLFVDTVVG

HSGLQELYLQDCFTTRPAMVGPVTVQAPHGKAAAAASSATTDEQDESWSAEQILKPIQAL

QSSLCVPSTQLCTLVLRFPLSDDAVQMLAKGLAQAPHLENLSLRGCDMSGKALSYIGDAL

ATNRALHMLDVSYQSNSVAHPAYLAEMRSSSRRRFSYTGNGGAALTAAEIARQELSSGGG

SEAPSREERQHPLLPIIRSLHQNRTLVQLVMLGVNVSIEDIEELCACIERSGNKTLTEVW

YTTSGNDALKMKLEDFLAANREYGAANGGDGAGGIGPSAVSSVRSSVMRSSTTRVSGSCA

NLFPDSTSQSDHTEAPPRTPTEWMSTAHNTNGNIPYSPSPVQLQLPHHPPRPGGNAPVNG

NAAENTSAERHSSLSLSSTMLNLKAGAAKPAANGDSRSVMSNASPSQVTVSLEGNVGKST

ASPRSRTLRFADSNESSVAPPSADGTADTRTVPYTSQLPSARKARQT*

>Lp_000061600.1 OsmC-like protein, putative

MYRRSLAKLGLFSVFMEELRKVPSYLSYKMQSGAKAYVDSASQTLEFLKGEDPSESPEMR

EKRRKHKEMYQAILNAQAAKDAAERAEAQQERSQLSWKQRVALRMQEAKEALQQMTSTKA

GVMAVLQHCTASHAAEVALEQGIDVKNVQMVLEKAAATSAVGHEDVVVGYIDAPSASREE

VMAFAEKLHKACPVANSMHIEWRQGRPDAYSRDGERARRADNLQGEMERAARQVEWEESR

SGASPRADTATAAAEPIPRGMPGSRRAYPSTNWITNRSLESEDDDDEFHLPGIGKTRKPS

ETKDGLSGFQHAANDVTGTPEEVVRPKSTGNSAFSESPESHRESVAAAADAQAPSSADAA

PKPSESASGDTSKRSSEK*

>Lp_000061700.1 hypothetical protein, conserved

MDQKGGSQNTCTTLKSGGSQKNARKSASRNAQKRGLSAARSPASPSKRASTASAQTQKTS

ATPSQTATKEERGSSVVTAAATPGQITANALASINYAAFNHSSLSAKQRRTRMLKDFLAA

HNTGAQQIIEEWEEEHQALWSNYLQSSHAPHSREIARLTKATQQIEQSCQQRLQHANAIT

RCALGGIEMLLTRLTECGTQISADTRVLSGELDPVLAGDVAPALSPGHETVSEHGTPMEE

VRGFDPSKPDPHPPAFRFSEADREEVERLKNVFARAVESITARRENGSCADAAYFSSPLK

PTTADELAKQSNETCPRNSSAAASPVMDGQHAAEKEGIVTAQAATTPVGAAAAADAERLR

QVEELQRELNAVGRAFISLMQRVELQHQQYQAKIKAQRDALDAEATRTKLADDVLRGTVA

QGEAEACSAVVEVRQLSLELRQRMDASEAAMQAALDTLIKESAEVCVANGALHDYAALTL

NRENCLYAYMSRMERQVVEQASVLREAQDTVTRLWTRLHTPASSQRPQQSTSKQSGKTLS

SSATPTKKSDSDRIASDVSSSLPPLYEELLRQSDRASLLELAERLAQHGPDVATVIVRAL

DEQQAHGMMHPAEAAAAREMHLRTIAVQQLLEKLDAEGHLRSNARQTTQPLSERIAHLVS

QYDAYVDFNEQYARALVRQADVERREHAPSQFAFFDPRTPAPGQQSTARTTPAKPANTLS

VPKTYASVSSVETAAAGGAATTAAPSSLSPVSLPYLQLWKSKQQASRQRERGMAAAPSGC

GDVGPATSVDSGHIIKYLDRTTAHASPVFGGIIPSPPHASTGGGGGGSSGALLSAPRARR

HTIVSATASTCESSTPPPPIPPRAGLTSYVLSTCTEKNAAVINAGKEAAAPSTSTGQKPG

SHVLPYRDGDRQFIERQREVFQQLD*

>Lp_000061800.1 hypothetical protein, conserved

MRSLVRQHVSAVAKLRCSSSFHRFTSTQASRGGPVCPPWCFSSIENNQRCFSTTTALRKQ

LPTSTGSSSSAAVADGGGSSSSAAGSHRSRKQTRRHLPSASAASSRSGPSAAAAVEPELE

SAPLPVNATTASTSSGAAASVPVAAQHRSGKSDTAASRAKAPKRGPRKQGSGSSPRGSSP

TIGPTGTARRRPIDEATSLPPVEPPTVGVEVVGSETAGSNPISSTRAKKKVKDARGRRRA

TGTHSRLNSSSPAEGGPHPTLTVAPMHAVTTDSVSSQPPSAGSLAPIYYTRNMLANTPRT

EEQRIAERCSFTKLIMVSASVSFLFTALPRSEGHLKTYVRDMNQVQVPSSASAPVATTTT

TLTAPFFGAASAADALPPAIAPALTSRQFHELRQRLCAEQYATNTVFIHVREMEQLVPPP

HLQSPPAGSKAAETNSDAAAAVAASAQGDSTPGRAARESKMRGRRSRRSRSGEAAASATS

LSPPSPASSGAPLPALTPVQRLLALQEWKKVAARRARETQARYPIPAHFHAITRVRFHNP

HQTDLTVSLGGGWKKSPNSVLRAIVRSMHEKQRGSGELADHEGRDSDSSGERLQGEEEED

DDDDDSSLPPFLVPDPVFTMGEVVTEAAADNLEQEQPAFLALPFAVKPLLTVCASCVPRR

EAWIGTCSPKPSTTSAVRASATEAESPYMTRPLPTVPRFSTRITIMLEHDEMDPADLVSL

AEISTTRKPASATAARASSAPAFGVPSDVSVRGGGVDVSGGHAADFTDTASVDAQRSCSK

AKRPLRLYCLVQDAGEY*

>Lp_000061900.1 BCS1 N terminal/ATPase family associated with various cellular activities (AAA), putative

MFFHGSSSSRDSAATAVAPTTAPSPTSSTAAATTTTSGSQPSIDLGALLPEGWEHSNSVV

ARTAGAMLSNPYFSAGAGLWVLTVGGMLGRQASVMFSALMRRKFVVSLEVSSRDSGYEWM

LRWLASQHSFKVQQMSVLTRTAAFDYSSNDRQHTECLFGPCPNLRHFFFYEGRPLTLTRR

RRDNVGPSYDGEIFETLEFTTVGTNPSFLQNIMKAAQLHAEMEDSNHTVVYMNGGSNWTR

QARPRSRRSLSSVVLPGDMSDFILKDVRKFLDSSAFYKQLGVPYRRGYLLHGPPGCGKTS

YVMALAGELRLSISLLNLSNRNLNDESLTSLLNSAHMDTIVLLEDIDRAFSNECNVTMSG

LLNALDGVGAQEGRLVFMTTNHVEMLDPALIRPGRADVKIEIGLLDVDQSERMFRKFYPT

ASDAMVAEFGSAVPPHVISAAQLQSHLFYHRDDPVAATRTLPAFIASSRAFDASMQEMRE

KKKKMQSLPRAPLLDFD*

>Lp_000062100.1 hypothetical protein, conserved

MTAASQEMADLLREVQKLQKELQPVRSAVAALQKRESETPNPAAAGASLHKVASRLETVR

LKYEPFLTRLQELLSDEVPAFLQQTPLLAVGEEGFNEVKATDAAYDTEAANASLAPAAAR

SLNRSTAGDTGPQAAQHAQAEDAARLLREVCPPATPHLLRPENLRFPQQQLRLRAEMVEY

LIATNRLVIADHVAQSYGLPRQWFPRLVALHRERSCSTSTAVAAAAASPLVTPATRPRTS

AAGGVVPPQSPIQLPPPVAPARAVGGSSVSTPIADARSPAGTPASVTSEPFTALPAPTVI

RLPPRDRLSPPTRPSPARSSAMAEEANGTTDDAATSLMPDLVARVVSAAMYLKDDYMDRM

VLERTLEQSAGLVSASFPLPPSSSQSPAAESLPATPQEQYKACVVSRIGNIPRHHLHTQS

GDPVFDGAMMNVIEDLLVYGLQRWAEDDTGMPDAVADAQRGSDAAETLAAETKKRKAALL

RSLTTDTFLTAEEMLLADKVWNEDILSLKRPTRFYCVETGLSTDEFAGLDGTAPRARATG

EVISQLVAEASGSLESSTGIFIT*

>Lp_000062200.1 hypothetical protein, conserved

MFRFTTPAAPANAATTANSTASAFALAPPPAGFALGASPAAATAPAAPGTAAPPVPGFGN

TTANASPKPSDAPAAPTAVVEPPAPSSYTKLFEKAFRDGTVSDAFAQEIRRRFAAPMEMR

LLPSNGAAAVAPTLANAAQQVGMPWKSDLTPPTSLAGSGPAPGSGTAGAAASAPSTSTLA

RNTAAQQQQQQRQSQVENAQSFLQLLLMCDSGYNNSEAPPAGKAASSQWTETGEEAVKGL

HAAMGSIATSAASNLIAFVKSSVVKHTQALDGESTAAASIFGDNRRKRPNDDPTLLAAQC

LYLLSRHFQLAASVVKEALDLVDAFYYLLKNSLRSRVVALPVFSENSDTCVLNTLTWLCV

FSVVNMVAVWKKLRSSSGDLRLNELIQSSAAASIEATVTTLRKRLRESVQSTTVVPNVGG

LHGGGAAGELRGGAARASAASLMPPAVYTWQVIVDSYVSILTLAEGCLLRAKGSEEGFDK

ALDAFLGERASAALSSASASHTAAASASVAVHRGKVTKRMVELAPVPRTLVVPVELLYIA

PYEMMSYLFDEMLPLLRHLSELEVETHRRYIEQLEELPRISSAVRGGGGGPTGGFQPRPG

AASLHSGGDGGDDGWLRRMTHVGMHGEVVGESFTSEMAHLLEALAICLQHLPPEVLNPDL

DADCAATFFIFQNFVKHMRQVFATQRSGGGGAASDTAAIAVAGATSAVWENYTLKLMTKF

LEVLAMIGRNPQYTQRVVMLLTDAQVECGELQWHSLVCQALECAGFNSGALNVPGMGGSG

GGVSLPAVAAAAAAASSSGLQSGRGAEAEEAYAASSTVVVGGGVSRTLHRQFTRAYARKC

QRQFIASFFLLLRQVFAHPTLRPTISAYLNLELALTFLFAPQQSQVMLGSTLSLISALIT

TASDAQLVWTFLEQRHLLQLPSVRTQRGNNGGGGGGTLDGTAALYDRFTAASREPTSHEE

TLSLIGHCQYESTQGTYDITIGFLNLITALFQNGQPSLAALGVYTTVTNFISQEIMRGVL

KRVFTFQHERYTVFSLAAAALRQALLVRFHGENGRTATLPFASVMAINKAPADVVGEVVK

LIFEASDAPYELLSHHRAAVRQALRLLITAIQTVQEQKIDLLLFDTRTTLNTDLAVRVLR

LCSLQDTILTKTTLQLLLLFPFETASQAAQYWSGLAAKYAPVLESFAQLLHPLSMVPAVV

QAPPELAQLDFDPAELVPGWTSALLTDTKSLLLDLLMRHADVTEPSLTAWMCGFYHEGYA

VHNHRGRADAAGSGSLTAEGYEDDSGEPSWWHTTLLKSVVEGACSDEVERVHPTLAVKCV

KLLYLLRANRLYGALVVRPFLESVCRILFLRLQHFRACQCAPVALSKYAYVLKLLALEAC

YTYRTSPGDLRLAQSTSIPPISVEVLLSLLYPFGAAPTESGQVRYDGFEASAPGAALGSS

RFAAGSFRDGGDSHTDDDDNNSSSNDRHGEHVAESHGTSSPAQTVTVVARDAAVDITSWL

PQALQVLPTFPEKLPPISGGRSHLVPCAADGVVQYNVASLYEALQLEQVRANKPPLTMAE

LRDKLRPFIAANDCFFSYAAGVSFVEGWCQLVSVSCSVVQGLSMSRLRAFALCILRGLDA

TTSMTAAAQEQVCARLCHCLSTVMAHLRKATLVAAGRLSLAASHTAEAPWSRMNHDGDLG

DRQSPVTSGNVLHGAAGGLVPHESGRGAAARSQIHGAASRDLGEERRSTQLASGMTHRFG

KRRPRDESADRRVSGNLSSYKSQNNLFATQVGDASNFYSAAGRAQLAQATLQQRQAEHVA

MAADSVSANTAILQPLVHALVQWGTRIATIRADLYMSLLCLAETPGINLDDVVLWRSQKA

LLSVICADICSGCAATTTPAVAVMSGAAIAAGVVGGVIGSGTGAPGMAPHSSAGEGVSCG

GGSNDGAAVVSANAAGGALGPQVQHAVALLVALLQASAPIRDDFCNPTAGSGDGLGTWAL

RCATALLQSVDNAVCGFFASSGVPLGSLLWHLRSAFDVLSVVSLGHASQMLHSDLLRLCF

AMQAWRHSTQVVLGYSQTALTHEPMMSKPLVEQNKEVLRQLLLGVVRWVNLLLSSLGDAT

PLLYEVQKFIRDNRSLVDYVFISPAAVSSTLPGSARLSGSHLTLCAELSECLRALSSSVL

AVDCRTLVDSMALPDLLNMLSSETVWRRGPSDLYDLADMEGVNAARGGVGVVTEAGAVTS

VAAAAAEAASTVATTRYGAAPAAAGIAPSTGGAELMESVGGTAATTSAAEHARDIVALTV

RNLSPLLLNAEYGLRGTEAESFLGDGGVSATPMLRFSASKRNLCLQIVRHVAHSLAEMAQ

VRTREFRLECHLYALHALVCLLHSFVLPLAQGAPPPPPQQQQTADDFLRYLKELPLQELV

EVLTQAHDAVYRLRRLNTDYRGGVRVHRLDARHPWAAGVDGGEAADGDDAAAQPLSNGMR

PMTTATADGGIPQSTPAVHSTEAFSVPSTPGLLPAASARRSGWDSGVVQGPPQGSPGLAK

ASGPCEGLHHRHGVPSTAAAASHERDPNSTVVQRLAGAPARGADVGNGLSAVGPSMLPNT

TDGEKLRPDSTLWELLPSRYADGETWTRVYDSFQNNGALQHSVNLAESRGASRRWAEVLN

VENEVRQIKVAIANALRATKGAMAEVRKLQ*

>Lp_000062300.1 hypothetical protein, conserved

MFRAHPPASREHTRSAAAGKAYKRLDRRPKRCRSPCEDERRYQELQRQKQEVQHRVRQQQ

EVLASLEENILDFYYTHTIRYEPLLLQLHNNNAARASGAPAVSGNLLSIFPELKEKGAAM

ANIADGTTASTPSAIPSKTRYYYDSALMRLFREVAIQERDLAQQYAAEEANVSATEQQIQ

VDIVDGTLASQLRTEQSTRAGLDKLNTTLTVLRSECDEIASGIQLAESHIEATEKAVEAC

HRRKALLEAKKQEQMDNAEKIKDEVAAFRGSVEAAARDLEKRLLINKSLEDEIGRRRNLL

RRRRKE*

>Lp_000062400.1 hypothetical protein, conserved

MQTEGFHNAAATRDPSLNKTVRDPIDPRRDASESASAHSVSPRSSPHGEELNQASNSSPV

SQAAVRSPYLALHSHYGNQQPYPLHISGQPNANMQASSNVTVHAKRPQIYSPQLRTSSNW

PLSPLSTGVSKAVSAMYAPLRLDSQLQGSPWSGFYQPLTTQSMPSPTRATNGGAGAAANS

SYWNATPSPVPYPCGSPTGYSSGIRIMSPSSQSLRQQQQQLHGNDDSSPTQLSFSAIGRE

ASSLSGGASASAFASPGATGTAHSAILDAPLQVSLVPPPRRDDYPYFSFGNSIMQSTPFT

PTVQTPTSRSHSIYHDTMMELSQHRQELDNPLSLIHAEGTRAFGHDCAAASFAGGSGAVS

SASITPLFVEAATSPHSLPPRVTSETSFASGSGVAVPRKIVDGTLVDWTKLAQMSPPSPQ

LLEPLTARPTDHTDDNNATGTDAAVKRAPHEEDVMDDSHRGSRHGTPETPPSRTDNAALD

DGDAFQQQQDGERSNPLNSTKPPHEARGNVVGPSEASDAEMSISGAAMSPSDADFVRGEA

DARAASPSSVKRCHVGSNSNSLRIATPKKTLSRRHTAQPNTPGVAPAFANVPQGVADFHL

FMPSPVDESSHGFAVQRDGLLMRHSPSEHSSTTLNVVPNCGVWQASPTLFSAWQPTSMPS

SSASSVRAALSDLHEVLRLLQSDVANVSSRVSVNEHTVRQWIGCTLSALLRLTVEVLHSI

QLSAAGEGESNGVNGKAAPEQTLAFAQLRDKIQSGAMNCVETLRDTVSAVQRDRDVLEPM

LLTSMLAHGAVTSLLHCLEKAYTHSDSQPTATAAAKHTDSHDAAATTTTTITASNIPANR

RIPTAAPVPAPLAADRIDELMDSDTLARELGGEWPQFTSDYNRKLLRYLLLRVLYAQLSS

DDGRSDGVGVGRGTTDGGSQHGSPAPSSQKSGYSSSSTASGNATTTALHKIESAWVDDQL

KRWSASKTDRKGAPASDAAASADEATTHLFREALCQDFPSYDVVSDVFSSFREGKCWRCW

YALLDQMMECTFHVRDRKDLYDCVEWEFLHAPSPSASAALHAISAGQITREQVEDALEKV

EVSLASLEEQGRARRVAILQLILRRIFVLGVVSSVHRALSKDAIVSDAQLATFVQVQRKS

LEEVEAQLTAEHAEGSTHIKCIRAFVSCISDASQDVSKASAVMLDGAAEAHGLTGPATPP

MPPPSDSQWNTTAVGPLAALLMAARSRSSTTERSDDDSESTRSEDEEELQKRICEALDLV

DDVMTRVLDTIQTLCVLLHSDVAATEAKRDYNVDPTKIAAPSTRALRRLQASLVESHHSV

MLCLASLHLE*

>Lp_000062500.1 N-terminal region of Chorein, a TM vesicle-mediated sorter/Protein of unknown function (DUF1162), putative

MFDRFVADLLTTYLGEYFDNIDREQVKVSVWNGQVHLRNLKVRRDALRFFDVPICVLMGT

IEKLTVVIPWTRLRSESVVVQIRNARLILSDKETALYDVDQEKREERVRKERELTAADEA

LLLAFKEAQRAAQKAPAVSTTEANADSSDNNFASRLKASILNNIRVEVEQLFVHYTSSVA

SAAGNTDSHHGDDVYDFTASQSSQSHDASSPTISLSQPADQNKSVRQASLTFQIQEMKIC

GCNMRFEPAFVAPGERMSRQLISFCGLAASLRTGARKVVEVPLFRPFNVVLELAYQPVLT

DPCTPQYMLAVHMDESCACVITSESCTTCYELLRYLRYVRERQALRRLRPAGERPTAKPR

LWWRYVLDASLQQVRRQKSALLNSAHKPTPFSWMTYATTKAKRDRYMGLYLRRQRVALEA

TRWLEPLTVDEHAEMVVLEDELSTEVLKLAKRLALERVTVERGEYEKLLLRQKEKAAAAA

NAAGGAASPVVASPASSTAVALPRASTGGWFSFWRSTPQLAVGSLGGGDGAGGGKVPNDG

VDDEARAELRELVQLMTAEKWTDAQRTVIAREFGMSADEAKELAQTNSGGTTNASSRPHT

APRWVIFVRAVSVSLELRSCRAEAQALHRAAPTQETLAKLLLGKLQGGAEWADGNGGRGD

AADTALPTAYYWGSVELLSVSAAKLSSQEQQPGEQEILTIRRTQATSGAGPQAGARQPTS

PLVIPRLAGDAVSRHARPFSPLEACWSILSDECRRTAAWQLEWTQRSSAQLTSRTSAVHR

AHVCVAPLHLVIDVVPLQHLIDFFFIVMWPDPAAHSSSGSGSGASPAPRVANVSVHASPS

ASMDDFLTALRIPTSHPLTRIEERCATDAEARLVILHRKVERQHSIEWDILMDCVTVSLS

EIPEVQGCKFVVTKLLLRNDIAHRLQRQERLQARSSKDSAEGGTPPAALGASETDWFDYA

NIEAASTSLSVFLPSTVSNSKNSRTAGGQGTPPTRPRQSVIHMPIFAGIPFAMVVERSLL

GRCNPEKPLYKLRISSADAVKLSWSRSTLSVLATSCTTLTDLATSVGETLLQRGGGGASA

KASGTDSGSEDVNATFHVGVVPHLSRRELWRRSGAPSAVEATTAAAAAVGDGLSSPDVFY

KQMHPFSALRLASPGARRRVDVRRGVCVLYHPDRLSSPAEYYTLQRGCTRVRRTDETGSD

DGDGDKNASATYVHLYVLQGGADQEEVLTDAYDAAKQLLLDTVGVPPALLQNAVWLESAV

HAWISNQEQTANAGGNVAAESPSIGQVRRALELVMKRTHLVPCRYVAFRCANVEEARAME

AALLHCCVAPPTPSLLLTPAVQAQFNAAVAAPPLRKQPLWSAQLNFPSLQMTCEGREPVA

YWDELPRAGPGDQSVPQTARRDAVLSFTPFSLCYDRFAEKQRFELTVASKTEMYACSPRP

ADADDSNNNGSAQGVAAKQPLLKIAPRSAAAGSASLFLRFVTYYRPQPLPSMKRCGIYVG

TTSSIRVCVGPAMFDWAEAWWDSCGLMTNRLFAEEVIAGYPWWWSPSVAELSQKAKQIED

AWCSEGEYGGNATTVMVDLTAAVLEVVVALPRSAPSSPNGKEEELTSMSFDKPPLVRVQD

ESPVFRFAARDASLQWRLTEALNHVHMDLKSPVLQWRPACAASVVEPSVGWVAVMDPATG

PSGDGPVAALAVDWKLKKSPPMLSHSDWLCNGSPAETATTAEAMAANKTRSVASPSGFRD

SQEMTVELSQATLLYWHPLLLHMIESFQKDVLRRAGTLVDREPCWFHAGVLLCPPLTWPH

HGAPPMTDISWDRLRKSFVLRRVVLQIPEDLTWMEQTDRGAPPLALGSVVTMDTFTYADH

LRVQPVTSAATTGNCQWAAVEQVHVIGLDGVSLQRSATSKQLSPCFVMTLPVAVLTLTFP

LFQVASIWKEKEEWHKTFHVSLCSRHNSEANSVPGEDVTATTTSAAAWRGTVADLAFAMK

LLHANYVAGSNPPAKLETKVKEPSQQQQQQHLSTPLKHAVFAEPNQVDTSPASLIKPVAE

TSTWTLDVGSQLKVCILLADSRTSLCTVEMGCAHVVVDRSRAGELSCTLDASQLLLEGGV

QSTDTNTHVPLFSFPTPNVAAPTTSPQLRIQYGWRWLAEGSTAERVIHASVDATHGVGVV

ANSFALAQVRSVFESPVLRESMNRYAVPETTTTTAAALSGWSKMAVHLQLAQLVLQFPCT

SSIGPKGAAAFHHNLQLCVSTSGVVARLRKDTEAWKGMLHVGQLVHCVLRENCDAASAAM

PAIPLLLQQPAGWSRPVASSPHVSRQTVPSPSRTWRAVPASMDAPKPAQQDMLDELFGEL

PARVSPAASALSTAKSGDQLEGNASPQSLMSASNTAGVFLVVELTHSLSKVTFTTTLKPF

WLLTPSMTHMSQLLTGLRHLMSSAVGGLSQTGETSGVSAPQQAAPPSATVAPKRAFSVDV

RVGAISVFVMPQEVTMGSLECTTQRVEDAVKHALDCEALWLATEAAVVQWDSELAESKDG

VKATTAAAQPNTETNGGQTVLLLRNISICALRGQQECTVLLDRFSIRVTRQCCDSEAGLP

SEEWRLALDPLQLTLTQSHYAALLRVAFQQTSFLATLMSSVSSASQSLPAAAAAEATAGT

DTAQPQAVQQASSGGVSRSIRLQLASLSLRVEEDGAGPEASAAHAGSLKAVYVHLRQFNA

IYHSGVVRASTTESVSASYPSGGQLRFVISVQSCLVGTEGAIPTFSLAPPASHAPSPASP

FVLHVTEASDATKAKSGVVHVKQVTVTVEAIPVMAWVDLLYAPYLQVAVPNYQTAKELVM

QRDLWLTEDLVLTERAPLRAVNSSYSLLYLCGNGRTIYMNAARRGQLILLDEGMTLRITH

AIICMEAESIEAYVNAANGSYVVLDRETCSVVRSCDAAAAAREATDNLFSPAASLPPPPP

SSCAAPTQLASAAVHWVAFEGDVQVQLRVPEPRTAAVNAGGAQRTLVLYSDMRLSLVRAV

SAIPVVWEVSGSFALAHAGIRSEFVTAEGVTVDMADLVSDWALTVQYAEENSFVAGEMES

SIGSRTSMQRQLRNIFLDASSGVEVRVRYSDIFFVLRAARHAQTAFAKWKDAMRRDLWSS

LAALPRTWNVEEVEGSLVKESLMREKRAPSVGSATATNSVVAAAEEVVTRTLVNVQIPYI

SFCAVDDSRNTETPLFRLYANEICTPNVALDALHVKAELQFTMQLDYYRLSKSQWDPVLD

PLRVNLALDWRRNVSVLDVYNRQGYVRLAVQTSSVRSYLTLELLRNLRQLQLLRAHFEAA

GMELYKTMEKDGTGAVEAFSAAPVPSFTLLQTTGLDLAVQLPNYVPGSHHNRSTDGEGGG

VDVADSGDSSATVSSRVLASGESWAFNLPRPQGKELPRAQQKILVRHFVKSQAANDARGA

AVSVASVGVRRVPLSTAGPLLRYIVADVSVSPRQQDQKLIHLHSIILFRNRLNSAVVQLA

LNVSGTYDAVGVVPAGAAQHVSVEVLRRRVCLALSASAAAPLRGSSSKPPPSYALGPDSV

VSLGVSYDALPCLVDKTFLCVASTITEHEDSAARRPVASPSPQTCLAKGKYFDIYSGAAD

KSYFILRVQAAASQPADVRRYPALAPLRAVEVVAEAVVTIHNAVGLPLTLTLLTRRVRPG

MRVGLLDTAPDTAVYTAVSTTTLCANASYGATEMDPLDDVCVSVALQQPNGITLLQWSSQ

DSGDARRSTAASGAANAFYPPACVYCSSERGQRDGQLVLADPLTGATLVLYIKYVKRQAT

LYCPHWIVNETPLALQLADTSSPQVGATERCANPIAGLGGRTVRTATMAAWDDGAKQVQQ

QQQQQQQTGADTPVYLYNSLRAESCSRNRSNAGSNGLFVRVWETAGSSDAGGQAGFSDWS

QQALFIHEAAEVQVVTCASRRTCGAVLVLSCRVELGSQTSLHAYSDTRVMCIRPRWVLVN

KSPYTLYFSQSLSNGCNATQRGEGDVAAPAVVYVKPFSEVTVPSLIATGASQLNPLLSFF

VSDDAQHNIQRCRWSAPLPINVVHEESTNVVYHSLTPLSEYWPQGSAETPGNSNVAQRIP

PNLPPLQPDDIKEVNGELFVSREEAKVFTTTAYAYKGCMMCVEVEEAAQPPILFENRTSY

TVCFQQRGVRRVSTVFPRRRKAWTWDAAPSGPSTLAIVELWLAQEQETSDARTPQLSTTT

TRAAASASSVARTAVCVLNFDPQQIGRQSRSSDGFQQEIEVADPVRGTSSMLFVRVRGVH

GLSYAVSITTEPTIDAYRTLPYPQLSFALQVESLFALLCGEDAQSILLCSVEPLSFAFAQ

GVRRRKDGAAASDLESADTDVQRLQLRFSTFQVDDERTGAKERVVAQLMDDRESGFQIER

KLLRTTPVLYCSIVAFRLTPIELHVEDSFITAMMKYQEVVRSTWDLAWPAASGTATTKRL

TFSLPCPPWKADLATALAQTRHVNAELEEEMKNGMSGQGGSRSSRPAAPLWSRVVAIDQL

YVDPILVSLSLYRSPGASDDPLWKIAGAASLLVGSTQDARLQWDAVQHRGVCDTIWHLFF

VHRDAYKKQMKKQYMSLVNVMGLDTMRNFVSDLLNAYSDDPRDGSGSNGGRAAERRQKPR

VPLRRSAKLCVDQPKEGRARVLRSGDGDAVAATASISPDAEEAEVASMSVTAAWLQRVPD

RLVPTQAQRAVTVAEVARSYQWSVFQAVAQPAEVRAFGGLALARAIAKMPSTPRHTQWGG

EGNARRMSAQEVVRTSAGAARVQCTRCVEVEALRLRRMRDGAATPLPHPVRDGFFTWEEF

AHHIGWYEFIDMCSDEEVSKYASMVCDGAGEASSNVCIVPFN*

>Lp_000062600.1 hypothetical protein, conserved

MEDTVNVFGRGTAAGEAIYRCYVTPSKPSTLDPQLAALLAKRRQEREAAEEAQVHPKAIP

KSRAPVNRPRVGVGKRPTEEEQARWRLQQIPHRRSKATIEAEAEALERNASPPPISERFA

KPAITAKEKDRLADVMAYGAELPKPTELTGAQRAKYRQMNRRAELDDRFTMLRQSAQSIQ

KELTDLRQRLPGQAAATSGASAAPPQTRLSERSTGHGSGSSGLLSPSRPAPSSDGGDGVA

SPAVADGLSVSGARLACQSSGLTKVEYYRRERELQEHLNTVIAEMEAVDYELRGLGTTA*

>Lp_000062700.1 hypothetical protein, conserved

MHCRFNLNRMLVSSTAATSLSTRVSAASSTLRQSTVRTAQRRCATQAEAKASAEAEKATS

AAGGAAEAKAAQHATPVSHRPHRKDWRTHKDIVYVKGVPMKGSLFKLPLSEQFIICLVFS

VAGTGAVYLVRPQIRYLCHHGFLGLTDEAGWKNGPWLYRIIYCLIMFPCYSFILFLCGGI

FGRRVWFSFMIHKMWSRFLTRRASDRLAYILDIQHY*

>Lp_000062800.1 hypothetical protein, conserved

MLRWCSLGRTPRHAFFFTTLPFVSVSHRRYFTGYQGTRSSVKGRRYYTDPLRAPKFGVRT

VVGRFDDQLNTPSSLATAAVDAMMDTTTTHLTTHGSRASHLIEMLSARGRTVRYDSAGVP

VEGSWYLLPWREQMRILAAAIGAFCITKAFFDMVRFELLYYGIWKLGYRNDDSFMKRMLY

YGSTALLAAGLFFSFNLNFFLSAWVMGRREMAAHMVCNGFAHLMPHRTIQLLERRLKLSL

V*

>Lp_000062900.1 nuclear cap binding complex subunit CBP30, putative

MPGEDRSSSRGTGFVHLNTPASIEYTAGQTLSLSALRQRKSRVECVVLPESIAVWRRAFQ

RYAKEEGYGSYAADVEDERMGNGVVLPPLVRQRSGPQRRRAQDSVSARDAFFCSPRRSQT

QSAAGAGIFATGDAAAPLSVSTDTPGSASPSPSPSSQAPSGITIVSHHKVAGRQQFVYPD

HDGVLSAGVVPQIVVTAEEKAEEAAAKAFYISALDMTEEELAALEELQMLWKSRARSHSF

LGSQHRSVVRGSTDPTATANDARGEEEGDVSLGRHRPLKSRRVEGGRRDAADNTAGFADA

EMAVDDVSTLDKELAEALRLADELLRFA*

>Lp_000063000.1 Predicted coiled-coil domain-containing protein (DUF2360), putative

MSTNRVQASREAASPSPPPPSLFATAEADADAQQVVQLVNRFLLSSVQFLNRFAEECESK

LVRTDGALQQLELQTQLLEHLLLSSGAAAGEGDEEAREDDDGERDAASASHRSSDDSSDA

APSYDEHRRHRRRHRSRDSDSGASDTARRLPQPPNNSGDRNGGGSGRSSPPAAPGAYRRG

PPRPPSGAARAAAAAQEAIAARVATLAIVGAPVLLPPPSASGEPPAPPPPLELRPGRLSM

RNHPKLKGYFELLALRVPAAFVKAKMQADGYQGDWLDTPDAPAPATLSTVVRAFADEPD*

>Lp_000063100.1 hypothetical protein, conserved

MASSSVSLNYPSSGEYNVLVQRFKLLNLVQRVAHRDVSEMIDTVGLDSLTRKGTLSRWVW

GDEEDGGNNGAAPRSTGYGRFTALRSAESQRHRELAAAAPRSSPHVGTARTNTTTTSSPP

PRMSFGRSTTGFEARVAAARSNNNEVEEDREDENGSYSTVRAPKTRRRYVVRRTRRVLRS

SPADRDAFFTRLAEPRERPEPVVEDVLVYDAEPSRRGGAARLSRRDSAREVARDSQRGPP

YAHERDFAQQDVERRRRLAPPLPSFAADDIRAHDYGASPVYPPHSHSPVAPLAAALPRFS

EALYDSGYPGARGEQRTAMAVRDDEAAAAPPLQSSLSSVALRPNAEEAESHEKDFTPPPT

RPTAAARNAAIGVVSSPPDGPVRRRSVNFALSEEDEYDSTPRGGNYVVTSLSGSGVDPLV

ASVPPAVGRASPQGHGVTTPAVPQLHFPHAGSGDGVAFSEVDDVAGANAPLNAPGSGRDG

GGGGGGVVGLMGASSSSVANSPRGLKTRSSSGVVGFPKSPRLGRGLAPKAKPKGAVEDSQ

VPVEAVRHAAALIVRSNSTPGSGLSPVTSTPGNSYAAGQRSSQPQTSSARTPPVGSAFSG

GGGGGILLSPRPSSVNVNADHGQPQPPIDDGSLPHFAGTPAGGRDPVPATPPFLTSTGAI

GSASRATASVSGDAGAAAPSPPTVAGVSAALPVNEDAQRSFQEVSSQVDHMLQSLQRVLE

RVCKDGDEVPASKAAATALAFRKGGKKASAVPSEEAYPSSFEEASRDGGYAEDAIEVNLD

SDNIHEDSEGEGEEEEEDEMLLYTRIQHEVSRLGTEMSQFALPNSSEDGEGSEAKEAAAV

TAASGADTSRSGRIPADTAASGPPRSSALQSVQSLAPRAPPRRARGEVPDAVVQRLCAYR

MEHFQYIAYNERLWNTSTTSQFVFAQRLTAALLEECWAEVMAEVDANMSEYVEGLVEHEL

Q*

>Lp_000063200.1 nucleosome assembly protein, putative

MPPKHQREAVAMPPIEDEEDDAMDMGMGMLDFKKYLDPDFSKDFMASLPEKIRQRAQVLS

AYNEDYLAMQKACNEKETAILRRYDALFAPLLQRRHEIVTGAAVTDEEVKKGMPAEHEGK

VSVEPDAAASAEDAKGLENFWLRVLQHHLVIGSTIEEHDEDVLRHLVDVKSSVVEGGYGS

FQVVFTFSPNDYFEEETITATVNATDAASELTLTNITWKPGKNVTMHTITKKQRAKRTGQ

TRTISREVPRPSFFWLFQSKSKADGEADEEDEEDDDEEQRISTLEVLHTCIIPNAVRYYT

GEAPDGSSDADEDEEEEEEEEEEEEEEIHIQRGRGGRGGRGGRGNRGF*

>Lp_000063300.1 hypothetical protein, conserved

MPAVESLLQLYNLDEQDGTITAFDSYGSDVHLGTSSGLLIHLTVSEAAAASQRNSEESFA

LPGRGGRAGDAEASPVSPSARSSPPVLLRTTVSRRCTLSATGTAVQQLQHSRSQRVVFVL

CEGRLLLVHAETYAVLSSIATNVVSFSVAQPVRAISHNSNGACVAPSATTPYRQPVQSRQ

HCGWTAATGEDEMADQHSRTVSHIRTSSEGSYGSSQPWTPRSSVAYPASPTTGPAAGPQY

KTSKAHVVCVAEKNKKELAVYLVDRVSTSGKGGATAPQRPPTSCSPSTTYLTSGGSNDTP

SSATLRAAVVAPSPPRVVLRQRYVLPEPAQRLVMCTPFPLLPPPPVSSARTASPSSAAAV

NTVVEAGLTVCVGMRREVSLLPLLGGVPRCVLRLDGSLPPLVSTGSDHNTYLVRTQAPNT

VMEVGVPPSAAAAGLRSATTAGGAELTAALVNPILLRMGYHRMSGVPGDGDGQRRPLAAS

SLVQAGKEDDLIMGDVFQSDAVVELVLARFPFVFLFTAEHCDVVSLLGEGSVGSSGGNRT

SLAPPSSSSLSSAVQRIPLPGVRHGALRGQGKSLFVASSRTVWALQLYPLRTQLAEMVHA

GRSEEAFQLLAFHQQRALCMAEGNIPSRGGDAKQQPLALLERDLHRMVGFARLYSGDVTA

AVRALRGYLDPRELLLALPDCIPPYAMAHMQPSATTLPAAPTKAEGEAEEDGDTAAAPSP

VRATAAARARERQENGSDGGYTLRDVDAVVSAAQVRSFLYADEDPASPLVYSAWPPPLRQ

SCNPDQKGTQRSGETQWRDASYWAHWGGPSIYNTSADDVSRAWRSTFAASLTAATATVAD

AFVSSCYDALKEEVRRWFAEVLLLPAAVKDNTDTPSATASPWKAPKSGDAAETGCRTSTQ

LDDTTESGPLFLSPSPSPHACTLAQRRAMAYASLVLAWQAQDFHTAYEVVACAAATELRV

EDCAELLCYLSEYRLLALLCFQAGNGEACVSLLKTHVCLFRTLMSPAAADAHLLLLRQPT

YVQVQLTRWRRCMEEKFILERTTPGGLEKQSEGSAPPFLALASSPATLSGGFSRDTLVCG

RGLWMQSSPALVARVAQALFGVYEDEPHSFASTQHQQQQGWAPLLEQIASFYNSQANLAS

SAAHSTSSFAWLHFFPTENSSAARLTLPHRGPVLTTTTATVAAAAAALPTPPPPAAPRLQ

IGNNDRGGGVAVVNAAGVRVLPSPLSEYFYAVEKLDLRTVRRLLASQPRLVLTQDVDGCT

GLHIALAQVRMVASLAHSSSSTARPQLHEHERTAVLQSTTLQVICALVGVLVHAGCPTST

LNCNGWSCLDVAAGACGGCVTVFDVVRPPFLPQWSWRKAELTGS*

>Lp_000063400.1 DNA-directed RNA polymerase III subunit C11, putative

MFFCPFCSTLLLVETLPDGNALQCATCRYVHTVASTRGIVATNVSGEPVLTIHHSFADQN

KKLMDAEDEAGNSAPPTVAVAAASSSATATNTDASITGTTAEELAGDGSAEGGQIMTIPC

QNEDTPCDSTKAYYIQLQMRSADEPATVFFKCVKCGYQWRQD*

>Lp_000063500.1 Proteasome maturation factor UMP1, putative

MFSEVGLARPQAREERLMHPVEFIQHSAPRQEEALRMNNIRTTHGLGAASEVALTEMTLL

GSRRLGALPSSNTLYNAYRGNFTELTPCDMYGLPENDPNVQPAPRALVERQFYGHELTMK

TMGMM*

>Lp_000063600.1 Vesicle-trafficking protein SEC22, putative

MASNGCFIAALIARTHDRLPLCSYTDENYSNANVIRQQEQRIVERMETPAGGTNRAASKG

SYYESFDHKDNIYFAFQDAATDLTLVVAVNKLLLRNSGDINGTNKLACGLLDLIFAEFIQ

AYTPEEITAPNLRAYQFIKFDATLRKCVTRVMQQDRTSGDGIVVGNAAAGNSSGNAAGGA

AGGGNGMTRRQVNPHYDALRQEITDVHMVMRKNLEDLMTHGEKLDTMTNYSAELVDQSSR

YYKKTVHLNRMRVLKTYGPPAVIGVFLLLFFYFYFF*

>Lp_000063700.1 40S ribosomal protein S13, putative

MVRMHGNGRGKAASALPYRRTPPAWLKIASRNVVKMVCRSSRKGMMPSQIGMELRDSMGI

AQVKNVTGRKILRILKHNGLAPEIPEDLYFLVKRATQMRKHLQRHTTDRDTKYRLILVES

RIHRLARYYKRVKQLPPTWKYESSTASAATA*

>Lp_000063800.1 WD domain, G-beta repeat, putative

MSLSSSYHDCTGIRPTGSRNCVASSPASGKAVSAAAEAPSAAAPTRSSVRRVGRDSKNLS

HGHTLGTCGQRQASPEAASGASNVVVLGTASLAMHRDRELSKPLSNSVPQPLSDSRHRVL

SSRHQHHVETSMDGRSSVTISRHPRRSAQPLPSSLNPSFQASVHNARTAESRSTTPSTSL

VRDAAAVHFTASMPAASSSTHLVHLRPLSTSCEADYLNFSYQRGFMGRFRCVIPVVVRTP

VAATLHHRATRSGAAVADAAAPLKDETMENDESCVRASQMTVHADAVDDESAPTTAAAAA

VAERRTHTVATAAAAPHSSSMLSRPRLTFPGSAASVASGSTTRRGRVSGRFAPSVSASVA

VSSLPRRSLRSRPTLTEVRGGGASAAVARVPAANMEPLARSGLHTSLWAATQNGGVEVRS

MADPSEVLASAPPSDARTVITALAAVCGNRVVAGHTDGSLRLFDAVTLQAVGEHHPHTAA

VTKLLYVHSAPQEAAVTETDGHATEANHSRQLHSLLLTASQDRTITVWEAASMTLLHRLK

GNVRGVCALAATSTGGYAFSGSDDGTLRMWDIVRGQQWGITMDERAQLGRRSRKEDLAGS

GMVANWPSQRLCEAYTESSCEGDDDEDRDEVEEEGEDGGAGSDQGPNHTTGAAYNRHGRL

ASPHANGAASPSQLRPPLPTRPPVHPATPHTATTTATATATSHRRLPRGGYGGVKVVPRT

ANTLSRGALTLQQTGLLARSRGNAGPPLTPPHSLPRSLPAAEDAFAPTDTTTSSTPLGGS

DGALAGFGSGISPLRPTLDHEWENGVDGRDKPAPAVHLGYDYSAPAPTRKHRKPKSTTTT

KKKTRKGDAEPAELDVGEKGGTRVRTKSKGMASSAAKRKKVKKAKKSSETTTTTATAAAA

RLPAGVLGQRLETWIRLYHQRVLHSVTSQQLKARFAAEYDAAAAINWPIECAHVECITAL

TVVEDRLLVSASYDATAKVFALPSGQHMRTLISSRRMPLSSVLYDSSVGRIYTGFSDGAV

SVYDLSSPELPLLSQLQSPQTILSCSFAALATAPMRRFVINAHLDASVSAVSPTETEDAG

ESGDAAVVTTIAQFDKTTQAHGPNQTTSSYRVGQPSLRELNAAVSLQPLQQRRVAHLTAL

AKRHATGTLEEKELRDSRQSGLVLTQAHRRRQTTHVFLRWQRWAWRHAMLRQLGALAAAR

ASSVAQRLLGHYMQRWANVVRQRAQAAAGAVLRTVQLKGSDVAILSVTAATRHGLWSALA

RLAASMATASAHSTLRRAYRRWRELQRVRHIELQQSVCFNTLLLSMDAGGAFAPGCHALT

RLTRTAARRAHHAKALWLLTELTERRQSQRQRRHFFDRWHTFAAERRCLLVRREEQAWRL

VEPLSSSLVQPRLLRCRYFANWRTFALYVARSGQLRSERETLQGEWATLRRALVTAATVA

AMQAKVRVAEAAIADAAGERAALMERVQTLADEDSALRTEGALRVLIAGYYVPDAATTAT

AKAAAAASPSAQAGITPAAVNTRRASAASSVDSGGVNRTRVKSFPSGTASSMGNAEDTLL

TEEEQRDRRLLMEASAVLRALKGNSMQCARDDKLLAKAHALALRLPIYEPLLNDLTASAE

STPSRRSGLSQPQHASLLRTTTWSVSSAKNCASTASPSSVVAAAAAAATHQQQQQRRRHS

TLSGPSVFAAALSNNGGVGGGSVTAARASAAQMWAAQPAEENYPSLADAFDAIYASLLGL

LYGAARECGVAANAAIRSTPVSVESCSWKATVIGGADNAAAAAGGAVSPLKDSRREGVIN

PKELPENSNGADDDLNALEGVQASAVAPSWLAQVPLKQRRTMVGEVLKLVTLFDSFAAHS

DLPVERAGSISTRGAANTRALPLCSLCSRETAQSLLRHAAVLLELADPQLWPRQLKLNYL

QDAYAAAIAELNATVSSSHASDARLRSSTIVTRSDSEDRDGSPLPTPHVMRPPALCLSAE

VLQRAADALQPTAARTPPMRGMPLSHLDARTSPTMTTVTTTTASSSDRQSTRLSVSKSYA

AGPPHSTLKLTNLEVLAGHRSDNSEEKLNGTVKSMEEEGQTPTPDPQYAHNTSFSHRTFS

AVSTPRSYTPRTATSSVAGNSTGLLKPYLGFRVNVTRDSHAARRTATITIRDVAGLYVNA

EGAEVDGPALAAGLQAGDQLVRFAGYAVTDLAAFNAIVSRHVHSGAELPVVVQRGEELLS

TTIVVGTRAG*

>Lp_000063900.1 hypothetical protein, conserved

MQSSSRGPSVGSSPTVQVTTRVAQNDTHQASSHDNTASCALPRSPSEQQKASDKAADTSQ

RNSVQEDDYHIFTSGSSSILSGCETVAPKSRECSTNRSARRGPHRLLTGLLSNHGGNGGE

GTSRRSKSNGSRKEQQQQDTTSVVVPRMTMVKPTSRASHNPLLEGYEVPGGNASAVGTRI

GAGGYDWPTPPSPVQRSENLHPPASSCRHELLGSPMLPLKSHPSHGGTPGRGERRNKHSS

SSRSRHPDHGESHFVPLSSVDLANRGGSSSVRRPVVEEANADEALSTSEANGRLGIARRR

RTHGNGGGGAASLSTSEIQSNEQLSAPSSSSNVRSMKDAKAALLRHAQGVSASVRVSRAQ

SFHRVPSNAGANTGGGNNNNNADLSTHRSAGWRGTSRERRSLNGSRSGVERDDGGNSLYS

FGVDDDAVRMSISPGSMGSPMLGRPGVLFPGTAVSQELNTTSTSHAGTSRRFSRTEEGSA

AILEVMETSALISHGNVAGDSISNAADSTGQHTRGRSTESVAKSKTGRRGNNSISREDTS

YGLLPNAHSNSSYHGKHRHVFSNGFSLSHTGGNDDSTDSEDSEEEERHRHKVEEVMRQLN

RPQRSTAGNRLSMMSRAAASGSMSMGNQVVAVLSTSTGHTTDGGGTSGTSVDHNNNGAGV

SWSGIGSTGCGGFASFGAAAGDSMTNDNAGGGWTGLGGNSSTEDWYAHMRKKAAEEEEAE

AAVATAALLHSTANESPAEMNAKMTSPGTMSITASPGLAPGNQLPSGTAATSPTINEAPV

PEVTRTSGTRRTSTFASVRCLGSSLTPTPDAKATASASTSANTSVHASSPTTRPLVSSRT

PIKPSKSSTTAPEGGVTYVSSLIGRFDRRPSGVASRADQQPRQITTSAEFSEVPLDLSIE

DIEVVGQDSTTAPSQPRSDSVVSATIPGRRPTLPEVSTAPSSNRRASRSSGVFFTPVALS

AVNVGGSGSASTSAGDATRVVLPVLPTARAEAWTPLSSTNRGVATPGHRRFDYRRRDSRR

RDSLPCVLPVPTGNATSATRAVGGTNATSSASSSGTPVHAVPHNERHSIPSSPSGEIPGR

GSAPPPPPATSASQNIRCSSTQPSASTDPLITIGVSNCAGTDTSLGTRRHSSPNKRTLVP

ATAGVRAGSTGLPPPGQPTGAAPKQPETGATTSRIIATSTRLPIS*

>Lp_000064000.1 protein kinase, putative

MSSSDQLTSAYVSKESSTKTLSTTFADGPNNGSGQSPTNGDVATNLSRNENISAEEHHLL

SRATTAAPMSVMPDGTSEEDHFLDTGVLVSPATEVNATPVVLRQGVSIADISQMRLEGCR

THQLTPSSSCHGSVCEAVPSAWAAGASPHRTSRSSSRAADVQDAPVAVSPSSTGGGRSLQ

AHSCSTSPRTTALDTSVAILQHMAVDTAARVEAARGGQTPAELLLMEAPSNTSTPPVLQA

QQFAAGPAVVEHVAMEYAIDPVGFTCDPDSDLPVSQLHTCSMKPHQRSLASSNSPLHSLS

ESELYGGQVRPLGVSRNIPAQALSQPVELAGDTTAAIAATTSTSALRVGESPDGTHRRLP

LVAMTPHALRAERSPSEAASPLSGLPDPTAAAAMGDASVYNHDFQRIISHEVKEALTRNM

RPIVDDDEDEDEVLLNDVLSGQASPALPPTPKQKRSFAHTGESRSCTFAPFQLPMGATSI

SRLDSVVGVSNQKSGGTVNDFAERTTFRQPLSPAAAALEGEASGVAALAAQVSSGAVTSA

ATSTATSTTASRGGKAGSPRAVASHAWRFPTLGKDADEADDMALNVTAMTTKACGAATSS

PQNTHRTTLPNSCNGNGNHQPEQMTPLSWSWRQHRGGDGSNAISGRPRSTNGQDPGVDVV

AATTAVAAATSAVAGTKGGNVPSTSAVAADDTEVGAGKSVGGGAGAVGAVRFEDGRLYTQ

PPLSTHSLTSLTNNGGVPEDSYPRKLEMVYTVYERLSQQRCNAPTATPKAVKEFVAAKSA

RSAPENLPVTPHTYQLPSSPPQRSPGVTATTNTMPSSGVSSRARRSPSLSCSGALGRSAG

YYSFGTRMSLSHPNRQALPQQYLGSCSALMHSTPSPRTAVLGSPAVLDLVVGSTNSAAGG

VGATANPSVSESPLTHATSSATRMARRSLNSVLVRRIESRPLLQSTLFLKHTLRAIRETH

AQQVCPLDTSKDSNTMSQLALSANTAVPSYQHELSRFANSFSGDAAAQTVGNERQASMAT

PLGSLANVASEDAELSSGQTRGPASASSRSSFADKALQSTSTKMSATRGPEQFHTDPNNS

RANSNGHAYAAALVQTPVEVQSQPETAQRYYRALTEAGQRVRHVHGVDGVEREVDNEALD

LIVYVGMRLMGWLEVVSLLGCGSFGQVFLCKDLRICDGHFVHPSEIGGVDYEYWNCSHAF

LPFSSVDIPPTNPPLVAVKVVKSVPLLEQQSVLEAEMLVLIGAQTAVPAGAAEYGNAPPP

EDPRCANVAKVLADGICYGHHCIVMERYGANLYEYIASNGHLGLPMYQIRSIGHQLFTAL

SLIHDECHIIHADIKPENMLLTLNSSRGVVRTNEAPPATTAAAPSKAAATARSPDTTPPL

CLPRTEEPVANSTTCSPERSYITLRTGPTAAGRHRHGQPPSRRSSGVPLDASISATSKLK

GQSFCHLRSSVTSRVTVVEQTNVPAPEPRRVQSLNQSAGMLPSRAVPAAQPDAPSLAAAA

GSSAAAASAGGGKSAAASPSSMIPRLHVKLIDFSSSCYDGGPFYQYIQSRYYRAPEVIVG

ATYGSGIDIWSSGCLLAELLLGMPLLPGCNDHHQLCLIEEMVGALPTSVVQEGANAELYY

RRLRPGETAPKAAKSTPQTAATAAASTHTPLPRPYALRSREEFLAITQSEPQPYRRYFTY

QTLQELVRHCPLTLEERRMGNGLQPYVPANESSEIPPNATPSPSVRSEMMKQRFLLFDLL

RRLLQTDPKLRPTASQVLTHPFFTSAPPYTKTFKLE*

>Lp_000064100.1 lipoic acid synthetase, mitochondrial precursor, putative

MLRRCSALLCSAAAPKSASPIAAPAAAAAAATAASTSANSTSNLMADVDKNDPQYKQIFL

ERFRKKLQSDKTGMNDLESFVELPAGVAPSAASVGPIKRGEEPLPPWLKLKVPKGMNHRP

RFNRIRRSMREKKLSTVCEEAKCPNIGECWGGDEDENTATATIMVMGSHCTRGCRFCSVL

TSRRPPPLDPEEPEKVAAAVREMGVDYVVMTMVDRDDLPDGGASHVVRCVSTIKEKNPGL

MLEALVGDFHGDLKLVETVASSQLSVYAHNIECVERITPRVRDRRATYRQSLQTLEHVTK

FTDGKMLTKSSIMLGLGEEEAEVRQTLRDLRTAGVSAVTLGQYLQPSRTRLKVARYAHPK

EFEMWEKEAMEMGFLYCASGPMVRSSYRAGEYYIKNILKQRGNAKAISTAALHTTDTADA

AAAA*

>Lp_000064200.1 hypothetical protein, conserved

MIGGEYKKERFSERLAAAQNQPRNRGYLPGTQLKTGGYSTGTLMGNWSEERSDAGYYDGK

AVVKPGLRVPWSTTYREMVQNVSAAPSGAGVPPVVGCLTSSKPSATLTAAPCDRTQFSQE

TFMDIEDHTKSSYPGHQPHLDPAWQATVQDTHRSTFQTSYVHPEVRRQEVRAFVPPVLGG

RPSSQSTGVLLRLRRELELAQEKGGAASTVSAFPGNVIRSVRIALANSCTDATGNVNADE

LQDGFASAGVNAAAAECVALIRYFDEQGNHTAPYAVIVDALRGCMNERRTDLVESIYGHL

KAFSEDGVVRLDKLVQWIDVAELPAVRSGAVTAEAARDAFAAQWDAPSPSAFISPTRFTS

FFADCSFEVPLDNAFELTLRNIWHFSGGRGSCENTSCRRVEVVHTNGRVTQEEIKNDLLI

RGSGAEVEPWLRENLAKQGIKDVKSLRVISPS*

>Lp_000064300.1 hypothetical protein, conserved

MKAVLQVGPAQFIVDGATFHLLCSDIQQTLQSLMPLSESEIFLTLHIDALERCLADDAQR

LASAGGPPPRALHDLESIKSIDSTQQMDRRANTRGAMQQQRRRLSVSRVCEPINVFSVDS

FDAAEAVVQELHARVLEQRLLLEKCVEQGTIAPEVEENVTAGKRSGSRVGGSVNGAAVSS

SPPRRALPLPLLRLDTYLSTSADVLPEYDAQGRRRRRLAENDGNCCFSQLSVKSIR*

>Lp_000064400.1 Kua-ubiquitin conjugating enzyme hybrid localisation domain containing protein, putative

MVRKAMDVGILLSPIAHRKHHKDPFDRSYCITTGWLNPLLDSTNFWRHLESLVTALTGEV

PRANDQTLLGK*

>Lp_000064500.1 small myristoylated protein-3, putative

MSEISYENGQPAYTGDTVVKCFKDNGNGLLFRIVNNDEHKWAFYNDTTNYNMIVKVSFGK

DSKIEAIGNTKMAKDEETGEFKCELHIAPTTTEMFIQGEPNGYKISFEANPIPRSKNANE

AA*

>Lp_000064600.1 Domain of unknown function (DUF1935), putative

MLPTILICVDLPQEEEAEPKKDTAEPTLSGEVSRVNIVRDSVFHDPSMSTASVSEDDEVE

FKNGQPTYRGTCIFKCFKNRGNGLLFRIVNNDEFKWAFYNDLNDYIVTVTCKTGANSHVT

PLGTTTMRTNVANGDKVLVARVGPCETAMFLDGVPKGFKLNYEAKPIPRAECLARNIKIT

DASSLSLNMQGASDDESHQRDSTVKDTPT*

>Lp_000064700.1 Domain of unknown function (DUF1935), putative

MHDEITYLNGQPTYTGDIVVRCFMGDNNGLLFRIINEKEYRWAFYNDTTNYIMTVKVSFG

GMSKVEPLGNTKMRRNEWTTEFELEVTVMPRATEMFMEGEPDGFKISFDVQRISKRNGKL

SKPVALAAPLPPPPQNEKSHSASEKEEETNDRMPF*

>Lp_000064800.1 hypothetical protein, conserved

MTDVQYKTAVNTRASLASRLPKKKAEAAPTNASLHQKAETTYDTAFAGASAGQDSQGLTE

DCSRSSPADGVASSAVGSGADTQQQDEAQRRGYKVLHYSSSEWESASHAASRNVQPVQQL

DFYSTARKVEQADDDARGGLVGSVQRTLDIRIKRAEDAALRPAAPFYLNTRPRVVPSIAA

EERHGEKHNYLDLVDCTQLKPQTEEQISYTDAPLTRLPGETLSQDLKVQSKIISEMGTSR

EFFRGTPKFLSDTPVGYAGHVPMTDRNVAFIHHGDDAQRLFAKSYMTLAEHGGGVAPPPC

GSAVLSLPRRGGSNKPAPKTLPPKSTEAINQTAEGRMQQMALYGTLERERQMNVRNDAQN

QNYF*

>Lp_000064900.1 hypothetical protein, conserved

MSSRTGASQLRFDALQMYHHDPVVSSPDKHEEPKRAPNVVHDARLGRCLVWRPVEGPPLP

RRFTMRERPASARPLDSLTATAHGLPSNVEAKKQLSAPCRLCRIKKEDIQPTLTSSTHGT

QALATQQTNCHTDTPPRGQLLRRGKGGIDDVLKNAREAQNLFYSLQERRLKAEYRNQQQE

LKAELDQQRHYKRMLELSAYQEELVHVYGPSMRDIIADVFFDDGHDPVLAFLKAKREAAS

TQTSLASGLSSEQLGEPTLSRQLWERSEDAAALSQLSPVEADRKRASQEREMLMRALNDK

RCGRRTSF*

>Lp_000065000.1 CEP19-like protein, putative

MASCDDLGAELWALVFHLFAKYKDNCALAVRELGLRTHHDFDSGGDAASPCTPAEVRGSG

WYRDMCASYAALKESLHAKHQADVAEARDLMMSLYPDGCNVSEYADAVRTSAAVLPCTVS

SDMIDYFISKKGDEFHAQGIHTITGEMLVAHVIEELMFSVLIPRQWWCSRSASVARVRRV

GIKGNPPTLLIEYERPTGVLHVRRVHLAAHLREHMPTAQLARRLAATHEALLTESQFQSL

LIRCQRLMSQPPATATSITASHSPLPASVVTAVAAGTTPASPLSTTPSSEGVAKNTKPQK

ADLGLLYRDPDAALQNVDLNDADDVTLREFKEVMSEKFNEHVIKPGDPGYVYDKRLEVAK

PVQQSDWDDDSD*

>Lp_000065200.1 Leucine Rich Repeat, putative

MPQLDNIDGSQVMVTSIDMSGNPNWSDDFEESWARLTNLRYLDLSNTALKGDIPDSWTGM

SSLETVKISNTYACKTLPNWNSGSLPSSWGSIEDLDEVDITGNNFCGCVPS

>Lp_000065300.1 lipophosphoglycan biosynthetic protein (lpg2)

MTSRSLVMMEGILSVIVYSCCSISMILVNKLIMNRYKMNYPNGILVLQTGGALVLIVAAK

AARLVDYPAFSIEVTRKWLPLTLMFVSMLLTSMLSLSTMSVAAQTVLKNLAVVLTALGDK

FLYGKPQTPAVYFAFALMILGSYLGAKGDQWVTVGGLFWTFLNILATASYTLYMRAVLGA

VSNSIGRYGPVFYNNLLSLPFFLVLGMFEIQPFMVAIGEATWEGRAVLVFSMLASSMMTF

GVFWCMALTSPTTFSVVGSLNKIPMTFLGMVLFDQFPTAIGYLGIAIAIGAGCLYTYLNI

LANRAKAARDKEAAEREKDKVAKAHEVVVRVDGEEGVSMVSTNYGLSTAGGVDSTKEE*

>Lp_000065500.1 hypothetical protein, conserved

MEFQNTLNDYFTVVDDFFRKTFRDDCRLFMAMKSRQYSESILGVNMFRKDAALLSADVLP

TPSSSPPPNTASSPSVTKADASEHVTGERAPTPIANFLSISPRMFLRRDGSAVGKVKVAY

EFDLPRVCRLQQALSMNSLGAVTAVGKVKELVEGLTAGVRVCVNTIAPASEDVTSGHVHY

QRGDKYSTLRYQRNGLGSSDLLVDCGITFFNLLLGAGFERRQLSFLEQREGPGQMDVMYA

GAGFTSVNWSIAAKMIRSNDAWANAQVAILQRLSPSTAVACQYNFDLAAAVAKVSLGCTQ

GIQLRFPTLVQTRSGGNSTSTSVNGAAHTSTSEAVMPSWTTPVPLVVAVKAESDGCCSAT

VRGLLNGSIRWGVVARKNVLDEAATIKYGLTLSMEYES*

>Lp_000065600.1 phosphatidylinositol 4-phosphate 5-kinase alpha, putative

MPFNRELLASVKLESISDSDWMDEGREKTNDGRSAVADGSLHAAPNSDEDEQQNAATKCL

IRCFKIALERVITSISLPEKQRPLNPRKDFTIEAVMDFRATRGSGGVACCGTGASGVKQR

RHAHSQMVNAAGVSPFPSPTHQHRRSTRHRELTHMFSLSDGYSNDDDEQQVDYGAFVMPG

AAAADRGSVAGRPRRLSKTLAHSHTFGDFSSDDEDDEDDEDQPPVNFTFTDYSPMCYRHI

REFFAVDPKEYCDVLCNSRWHSIPTPGKSAAQLFFCGRDWVIKTMTDLESEFLREILHRY

YYHVRDNPYTLLPHFVGHHRIQIGAKTFNFIIMQNVFATTNTIHEKFDLKGSTIGRFASD

AEKRRATCTQKDLDINSPLHVGSERRALLIDQIKNDCEFLKRSRIMDYSFLVGIYVLPSA

ADGGATQPSSPGPVLRNVAETTLRMTTVSPYTNLGERAESTGADLADHTRSSNLDGRCFT

SDQGGMMSNKVSGLRQEIYYIGIIDILQEYNARKRLENVFFGAMHDRKRISCVDPNDYAA

RFIAFMSSIIV*

>Lp_000065700.1 exosome subunit rrp6p homologue, putative

MPPKPADASLPTTKTVVSAAFGAVKDYSKLSTQLPADDYDYHLAFPGFRKHIRDDSKTLV

SLMDACCQMLPKRRRISLTSEEDPHSGAVHLAETQRTAVMEAIDSLLENVDSLLDEVKGR

KLDAQEQLSVTFGSELAASPHEDGSRSGVNGLGGVVRLAHVRRPQLSFAKPVDNSAAPFV

PVYHDAAGVVHTGVAGEHPFEEAIKSFAVPERQLLPRAEVPPVPLETCPLSFVDTPEAMA

AMVAKLLLVNEIAVDLEHHDFYSYQGFTCLMQISTREEDFIVDCLQLRSSMAALAPVFLN

PAILKVLHGAREDVRWLQKDFALYLVNFFDTGVALQTLHMPYSLAFAVDHFCQVKLNKKY

QTADWRVRPLPADMVHYARQDTHFLLYVYDRLKALLLNSEGRASIGNLLLHVYNESKQLS

LQVYTKPNIDPAETYKLALGRSLGGLSAVQEEVARDIFNWREAAAREVDDSPTAVLHLSS

VLAIASKLPTTAKDLLRCCSPATAVVRDNVAHLVGLVKAAMAHAGDDVDSTYSGSAAKHG

RGGAEGTGALLDGAAEGSLEWAFYRARCPVGVHRPMTGTLPSLASAVKTVVPADVAEDER

RALLANTMPSPWFTAMKALSRVLASRPQPHVELPGADLMAARQAAVVAAAATKVATEPAA

KEPRAGETADNNAREDNVDGRKSTSSEAEEHPGDANEEASADTKEVQEVQASSAIPADKQ

AFSIKQRYGVGAKARPKVGGKSGAAKKSA*

>Lp_000065800.1 hypothetical protein

MLPSARVTGIVPLDDAAGSYARARHTDAEFQGDYEHQVRREERRPSCVGEAGASPAVAAP

SAAAGPAAAHEGDSNDVADAPQGQQEPPQPPQAEEEQEPPIEDLVNKAVIHANKPIEHLP

LISPPELKLIGRCVTFKGDTTVDGETRTFYYEGLVGTINKETVMLIHVQRYTEEDFQLHK

LALRTRHNNGSGSGEKDEGQENSEDANNNNENGLNMGDAPTTSATAATTREEQEARRRNS

DSMSDSGPNCAANGNDFDFDHTAAAMHKMETPQLLNDLGETHEEATTRVRNRMRGHCGSM

GPIPYTTFSRSRIHRVEFGVDPRSSFYSIFQDPTKLHFDMQCLRMFVRRYIIHTSQGNNP

RMVPLRAFITCRCNCPDLDNELLIQTTREELAHLIKIDRDVKRAKKKKEQGRRNVLRAYR

APNGLFRSTGVLYLTHLPRQTFTIGVIELIVILAVLTYEVTSSASSDLAVIAPYVLRIYP

YVFATVIVSVIATACTLLHAVRMAVPASKQIYLIIIRGLFTFGAVGCSIMTMIVSGEMAN

LNRWMNFYETELLQERLCYFYALHHCSGISESCVTNFNESECQCTGYTSPDQFFPNSCDT

QIVMYIQRTTIPLVCMSFLVFVTYLFDGYLHLRLFHISRLLERRM*

>Lp_000065900.1 mitochondrial carrier protein-like protein

MGDIDREERHLSPNKNSTRVDGTSFSSKVTASPATSPSSTVGDKLATREGDEDEVTVTKS

LHVRRDWYSTIATFIAGGVAGAASRTLTAPLDRIKLIVQEGHLTQAPAGVKASADAAVNG

TGTSVLRRTHPSLLHVARLIKADGGWKSFWRGNVINCFKAGPEFAIVFSIRRYVSSLYED

CVEREKRRRKRSILRWKEHDERCSAISRREEEERASMLGPVEDASAAQRTASGRRSAASG

SSTTTDQLTDAGHLVAGPASAKPASSSASTSSTAPNWRYLTPQELREREKTISAEASIFT

PPFNQLGCLSSIPQLVINCTIGAIAGLGAQGILYPLEVVKTRVVVSRTNEYRGGVREIVR

LAYKKGGIREFYRGFAPNMVGIVVYRGLEMGLYSTAQQSIMLYRMQVLKKTRHDAILNAA

EVGMVGMAVSTVAQTVSYPLNVIRTRLQTQGTNGRAKKYNGMVDCCVKMIRNKGVTSLFS

GLTANYLKAVPASACTFVVFEWAQQLLVGDD*

>Lp_000066000.1 Cytochrome b5-like Heme/Steroid binding domain containing protein, putative

MSASNKKKVSSALSWPPRRWTVSVVVVLHRKGYTKKLGMMGHRWGVALKKRLCGEGASSA

SSGGNIRPVSSVPKRGYTREQLAQYDGVKNERILMSVKMKVYEVAPHFYGPGQHYHIFAG

TEASRALAKADLTGKYLNQYWVNCTEEELEVLEEYVEKFNLKYPVVGWYVADEAFYKVTD

*

>Lp_000066100.1 hypothetical protein, conserved

MPSKKKVIGSAAKEELPDPATVKNGSSATYIGGWSSFETVESITEVSEQVMEAHTGPDGV

VAALPRTNVFLLPSSACATDDAVQPLTVVVVDQMNIEGVSRQQCGRANGAVKTWARSADP

AVTASSNADAPGNEQRPTSGESRGKGGEATAQEPVNPFEDALHDVRYSQTVVVGASYAAR

DVVVSGSCSIEGMPPKIVVPPQLPSKPDLDAVTLDEPEAAATAANDAAGKPKKSSSAAKS

AARNAKKGKAKLTPEQVEELERKKAAVNAEYLQQTEQAMQQAQEQADYLMTFAHPDRWPH

VFFRHVTFAGPVVVSRAHVTFQNCCFTATLAERPQLLVSQYCRVGCIKCTFEAPQRCGVY

ALPSAQVTVRKCLFTGAAQTAWWAASVPGLSLQAGGVVADINANEVGGEAAPEDGEAAAP

DAADGGSAIPGKNGTGGLASPIALAGEVKDIVDNALQKRSAAVGIQTDSAKLHAQACGFV

ALGIGVYVRGSYSVYLSHCTVTMKSAQQLTADACDVVMEANAFHHFANTAILLDKTARLV

GLWRNFVEACAYYGLDCHGGSKSVLVRGNLFTADAVVRIREGASVTLFHNDFQSIPLNDN

VYENPCLQPVY*

>Lp_000066200.1 Retinal Maintenance, putative

MSDGNIDDLIEELYPSKGKTNPTLHSTAGVSQPLHTRRQSEWDDDDDDDDGGGVHGRNTA

GATRTAQVNRNRSGPVRPSAGHSFDDSSSSSSSSEVKHNNAVANARMTNGSSGASDPTSH

NISGDVSTPPHGSSNVTLHRVPFPSLSTSEGAFSCAPRCYLTNIGAVFRGGEGHPTLPQL

RELAVGERSIVGGDYAKAMRKKGAFHAVREILGNGCLDNRGDGTSDGGCPHILCQKCNYM

VVRLQGAEWDDDEGRFNLYLTLRNYYPDWCQLANSTPVGAEESSCGANRLVLKVNPTAAA

YCCQCSWLTVKSAKAVVVTRLSDAFLTHAGADGTHPFATVLPLESGEKRRPPLWVCHGHP

*

>Lp_000066300.1 Protein tyrosine kinase/Protein kinase domain containing protein, putative

MHSNCSTLDIAANCAEGYLTMHGYWLQAPAVIQDSSTCISDEANSGSGVSAKKERCNASG

TIEPYYFPDSILPPHTCGPLVEDDEEDEEEREAEGEGEDERASYEYYFHPSGSAGSAEAA

LSRQHSVPAAIDAVNSSGEASPGALSEDDTVPFLEEELLGDLRDVIGYGGDRGSFVCRVT

FPPALLQVTQQRALSSYSSLWSLSSPPPRDREAYKRYPSQISADELSFVATAYRCAENNA

HCQSVRSSFLTVEEAQYAALHLVLPDAIVTQHHKKVGLLMPLYNCSLKEFLQSLMLNSRH

ETRLAADSLVSSTNLEDNLSSSEDEHHGAFSSAIKYLPVDSIEVIAAIAFQVVEAIAYLN

HRLPHGGTFSGYTHNDLHLDNVLLSYDGDVALCDFELVASTPCPAHSAEIRRIPPSTRQS

PHGLFSETADTWAFGLLLVGLLTGVDPLFTNSIVNDFSDDPQLSRWDRGARVLDWEGNIK

AHVEGLLRMQDPSGRRLHDARVILDICGKCLVNREDAEPLHAIELLEEPLFQVYRKDFQL

ATRTIKAWLVEKRW*

>Lp_000066400.1 aspartate aminotransferase, putative

MLRQISSAALPGAVSGSGVMALCATRCHASTSYFAAVPRAPPDAIMGIAADFAKDTNPSK

VNLCIGVYRDEHNKPFVLESVRKAMATIVERDTQMDYAPIAGLPSFVSSSQALCFGKPML

DTQGDRIASVQSLSGTGALHLGLELLRRSSGFTGPLTVHVSAPSYPNHLNILQHLNIEAK

YYPYYNLQTHRLDVDAMLNYFRQLPPHSVVLLHACAHNPTGCDPTPQEWEKIVDVVRRGD

LVPFIDMAYQGFATGDIERDAYALRTLNSHDVPTYLVAQSLAKSFGLYGQRTGALHVRCT

TPKEKANVLSQLQSSVRATYSNPPIFGARIADEILRTPHLRKLWKTELLGMAQRLQSVRH

RLVAQLRGCGSTRDWEFLEKGIGMMSLTGLTEEQVGRLQEKYSVYLTHNGRIAFSGLNME

NMAYVAQAIHDVSSH*

>Lp_000066500.1 ppg3-related protein-like protein

MMQRHDKAPPDGSKRRASLSSFDRWLVRMQRKGSRDSSSNRLYNSGDYRSCSVDDYHVDP

SDGVLSLSPQPTASAPSGFYSQPGLSRLEELEGLLFTQNRFSHPCALTRGEDQRFGSQQG

AMCALDGAAMYGAGYRPPPLQGPSTNRLLCLPESEADFSSMVGAAAAAAAGGYATNTTYN

GCGGAASLSTLSEPLGGTEADRYIARLEEEVQLLMRESGERDRLMLTVVAARETVALQLE

EASRRSAIEWDECAAAQTITYGFMRTLSFYRTLEERSRAMELNTQIVILEHKLKLSETMR

QQVETTATSTAASTAATQALQRRLEEQQLAESVTATVRGLLEQEYAQLTALMSAMPSKVQ

TLLDEHQEVKATRAEAAVPQATAALSSADAAQLLSLVSRVQHYQDVVLELYCDAVDAKAS

QAQYELNIVRLIFEKQSSLTWAALYEEKKDEVRHLNEELMEMRKELRAAVAATNTATAGV

TPRGLHNVSSFAGSTSSQSYEAYAAPYVPVKKKSSQAHHNAAVSASTATPARVPVPRKSI

GELREEARRLGAHPAVYYPQPVTSPSPSMPSLYVSHTPSAVDQTVLRLNSPSHRDSPPRT

PERHSTSLTKSPVVAVIRSFAPRSASRGASASSSSSSSLPIAAAAAEPKKRVGTPLAAEM

SAAKEAQMTAASAKHSKAAGKNKAPLPSSRSSSASSSISTHTADVPATPVPAVHATAVKV

SALQHVATTASSSSSLSSTHFATTTGLSTGNAASSPSPKAGPTPTAQAVHNSFDDDNDDS

SEEAAAIKAAATTLPRKNGKPQDQQQRQTAKATAKVSPKTESPPAVQVKRISLDEAIRAL

ERAKLGRASGGADSDSDDNDDDTSSLSLSTTSDSSASHPGPRVAQAKAAAAVAATEEKTK

KTSIKVPQTKPTHIRKSSFDDDDDDDDDDDGVAGSDSRGQCNPSSKPPLSHATSTPPLGK

SAATSKPEPAAVVSAASTSKKPHKLKLPTW*

>Lp_000066600.1 hypothetical protein, conserved

MEQPLRLRFLNTMMLMGILSLYVVYDAGFRFLMSQSDTLALHAAILHDAAVLKINGESGI

AFDAAAEATSTPALQAYHLHESHPFSRAADAALSSSAATDLPHLNNEASSLADFPMAAVV

VESTWLSPDQVTVSASYAQRLLEGFRYALLTGAPWLLVVGSAEETALGLQHLRRLPYPAE

TLFGEAATARWPTELTHIAESIRASMAPYATLGAWMHAAASTPLPSYVSPALPITTAAPA

LTRSSVAEGQSAALRFSLSHLLSASALGSTVGTTPSQGRPSTLMLGAEWLHRSDAAVLLH

SSSSSAAAPRVTVYGTDSVYDASGLDEDTSQSPATAKPQQQQQRRQDLGMDGAADDSHSS

AGDRIGVVGGGSWETPRVWTLQPSGKQQVHIVVPGLVAVTVPDANKRARYAATALQRLLE

QTLVRRHARRVGDSSEEGAWWWWLGRSYGVAVVGGEWEQQRVKLLYVKALREAATETRDS

IRRAVQDVHRARCQFEGGGGAGDVAASSLCRSTRRLQLPFTAAVPRAPRVFVLPPAFEDT

PTSEPQPEWFNYTGELPQTVNADAPYAARVLGRWSFYLYTNYRRWMNWFLASLPGGTYQD

HFVLASVLMSDLADHRISWQDALYTR*

>Lp_000066800.1 pre-mRNA cleavage complex II Clp1 protein, putative

MQRPSTRRTVHLHQETLTIQWPAGQSSVSAGGAIVLLSGRAELHRSALTRNLRYAFPAEA

CIVVETFDDAVLQVEGEATLSQAPLSGTLDEIHALLDTARADAIVAISERERTALSSSDD

LREAWQGPRVLVVGENQWERETVARSLLNLAVRRGSPYGVGFVDVDVAMPLVGCPGTVSA

AFVEEPVVAPEDFDVMMPLTFFHGTPSVTSATRKRYLDLCVCVAQAATSLGFANAKFEAG

GLLIHSVSPSSEIQHDVLSDLIAIFAVTHVIVTGANYELEKFLVNAVLGRSVTFLRTPKV

PGVAPPSAVTAAQRRQLQLERYFFGTSRTPRLPVRGVARMCDVELLHAETLRRLEWDEVP

DLSLASVVWADTTASADEANVAGFVVLLEVGKEFFSFLAPSGGELPKPYLIVSPTLQLPR

ELVMPLYATESA*

>Lp_000066900.1 Peptidyl-tRNA hydrolase PTH2, putative

MSALPKTVALPPAYMEGLVIGCVTAVLIASLVSYCVTSSARAKSAKAIALAPVPSRRARA

KRLLEQLRQQEQQMQQAGSSTATAAVAEDAGNEVANGVHGDDSDSSNNNNSCSSDWASTS

DDAEDEEDSEYERMEELRLKMVFVVRHPVQPKISAQEVAVLTATAAVQLVELHQRNNGSS

SLAVAGGRSHEESLGSSPALMSFTPNAEDQQRWLQWYLWWNRIGCAKITLKCPDIAMMEE

VVRCAAELRLPAVQLRRSQFGSQEDGVMAEKAVVPPNVDEAVLIALGPAPADLLEPVTGA

LKLFS*

>Lp_000067000.1 hypothetical protein, conserved

MLKSSLKTPSSIDHDLFPLLTVRSCVSEADESKNGPRFCAEDKMTGAAYEVRSRKLVLAR

EVNPDARMNATQEEEGQLPLLLSSSNLQVIRRQRARIDALLSAAASSTVPPSVALPIDVY

IQEQPSTGDTFVASVEAFAGLSLGDIIRSGWGMMEEQAFLEILNAVESYGYASASLPPHG

NLSSDAIKQLLIVRDGASEARQPSRWVVSDWLLLSDDSVCVAAAAEAFDVEAFIGDLEWT

LHSSFAQLRISTSADGAYLAAAQVEDAINETVERIRLFLTQQAAEKAAAVAASSPDEGVS

QEAAVAASVNGTPPNVVQSVSDNDGVAATASADHAGSDNANVTVGGAASSSLAASSPASY

AQADLPTEPVSLAATATTTSPRKVSDTSKGYGNGVRRGQNSPRQLHDDANNTTRTMTLKD

KMAYHQAALRNEQMLLNKQRRKNAPLPPRPTSAANPAYRAAFTSKFPADDEDVYYDVPTL

GLPIQVKPSAFLMQGRRTSATGGPRSTGSSARKQQSPRSTHGPNSSDCRTPCDGSRSTTT

ATAAPSPETKASSGQRRQQQQQQRVSTPRKPPTTPRDLRERLLDDVVNMAVMNRSRRGQY

LRQQQEEDRKRRELRQHVLAQKTSPRLVKAAAARGQHQHTIPAVLEQPRFTGAATASWSN

ASAAVPAATTYEQQRGIIGRRSGAVLTTHNNNNNNNNNTAAAAASCHSNVTTAAVGTGNA

DAPSGGQAASSFMQADDWPTNLSESPEPIPPSPFARPVMQTYTAPTKAAGVLPHYRTPLG

TPRGRGGAAAGAATATPQVNRDRLGVRSGRVLCPTVGRRLQRTGVNVARTGGLVREVAPS

TPTKTGCNPLRSARGVAAAAPRSTALRVAGTKAGVSAGHPLSARGRRQLAAAAASAAAAA

PAELPVKEEEQQPQPARSVSSSSQQQPQHPTSSSEPPMPYVKPLPLAMLGLRRNSASGSP

HAPTAAGGTHNREAYDNAHHSSDLNMKSLHGAEMSLSNTLNATKSSGGSARRPALPVSPR

AAAAGLTTLPPSSNRGSPRAPNTARPVPPQPALSPRGYRRVLSTGVLQSRAAATAAGGKS

GVRSGARVRSSQLHTTAPSRTPQNGGRETPGTATAEDDAAGGDVAIAGKAAVVTHNPRLV

PRKAVDVDGGKVLAAVNRKSMRWLVPERVRASHAAAGEVQETPRTASGVGLGTPRNRPTS

NLRTGGGEKQQQTSPGAARRKSTLTLGNVRPVNETSPGILLRHHRRATA*

>Lp_000067100.1 hypothetical protein

MVSPKARLYAELAVEQLPRPGVRVRLGDAFVMRELEYKDYFSVDRKQLRRTTWWVEKCAL

PLMLKEGYDASK*

>Lp_000067300.1 D-3-phosphoglycerate dehydrogenase-like protein

MKNVVVDPPYHALLLEGVNPAAKELLESKGCVVETLPSALGRDLLLEKIKDVHFLGIRSK

TKVTKEILDAAPKLLAIGCFCIGTNQVDLDHANKRGVVVFNSPFANTRSVAELIIGEIIS

LSRKMTQRSEEVHRGVWNKAHVGCYEVRGKTLGIVGYGHIGSQVGVLAEALGMNVIFYDV

VPTLIIGNATKFSHINDLLTVSDFVTIHVPETETTKGMFGEEQIRLMKQGAYLLNASRGT

VVDLDALAKALRDGHLSGAAIDVYPEEPGSNKELHKTPLQGIPNVILTPHIGGSTCEAQA

AIGTEVGSALAQFVTNGTTAGAVNFPQLVPPPVDKSNFRITNVHLNIPGALKDINKIAVD

LGCNIGMQFLSTHKAIGYLIMDVDKDVAAELRTRIAALDCSLRTLIIR*

>Lp_000067500.1 Tyrosine phosphatase family, putative

MKKARYLVSKKKLRTIDRDVPHGGVDLDLVEIHPGIVCMGYPATGMEALYRNKYDDVLRY

LDYKYKTDYMVYNLCRECAYQYSTAKFHGRVRSFPFFDHAAAPLQLMPAFVKDVQAYLDE

HPSGTVVIHCKAGKGRTGVMACCLLLALEPAFLQSADAVMKYYGEKRTKDGNGLTVPSQR

RSVEYYARLLQEYGGEVPATLPTIAIASIKFVGMPKKWSLHKLTLLVGDAEEPSFVAHVR

EPSANLHVDRTYTITEDGVECGVLTLLCEEEHRLRQLCGDLRFEFHGANNSLIGVLSVNT

LFMERTYSGEVVDKLAKHCDNSVCHIDFDFVSSH*

>Lp_000067600.1 Met-10+ like-protein/Methyltransferase domain containing protein, putative

MIAQSVRAFLIATLLAFALYLVVQQLNRKPESTKKKGPETKEDKKQKQKQKRQAANEKDG

STKSQRKPFKGIVSQKIDVFTEQVCASFPDITADEARRVFPRKYEVHGHVVVVRLNDGTT

EAALQPLAAAFAASFAPVMVDVVLLDVEGIVGELRRPSLKVLYKSSTALTDFAAVLRRTL

RHRWRDRHAKHRDACSVSAEELEIILTKWTASPTYTMHVENGVSYTLDVARVMFSSGNTT

ERMHFATIHAVDETVVDMFCGIGYFTLPLAMHGNVAAVYALEKNPDSVDFIKVNAVLNRV

DHLVHPICGDNREVGNEVVGKCDRVLMGYIPSCRPFLPRALSFLKRNEAGRSVGVIHYHF

LADKPNAAADALKDVAEELGADVAAAARIVDLRCIKSYAPKRFHYVADLVFS*

>Lp_000067700.1 hypothetical protein, conserved

MENSELSKTILDEAAQCARGRLDSGTSARLIRFLNEAGSRNHVYQCCLDLAQDPGRRQRI

MMLLRNYVHSFPPSLTCLRAIEHFCHLHSTADVSQELLKLTETLLREAPLEGLATRMPRT

SRFGYASEFFLQSDIPSAAPLTATSPMNAAANASHHNSNNHNYNNVNHHVPVTAVLESKW

MDVSIKKNESQKCFADVIYRRVWCPPYLLQFPLLCLESTVERQLKFMCTFSANYKNNRNV

AQATLFFIKQLCDGYVSKLPLQDAPTHFRSVTICYVPMLLEMMESDYLCVRNHVYDFILN

LGVHMQLVDPVGVYPGCTMALEQELVWLLLTVTGRQAVLKVSDETTWTAAAKCLLAVIPT

CYQHLVDCRVLFQILQLPGLWEIHAEVFTTIAMAFARSLLLNKDIDAPPSDNLTIDEAEL

SKLGNYAAAAILTVYRHSITPGARKALFQLLLAFAARRNGLQSAKNSSRLTSPPPSSEAR

AGALRDFVAVDFFWYVQPLLYYMSENVQRELPRRIADGLVKSDFDGTTKSWSVVLPLADQ

ILGILAEDAELSSYMQARFKTVEDMRKKDEGAFVAALEDLLNEVAETVPQLVEEQTDLGD

CKVYSAAWRLAFVSLRWSARYLPDDKHRALTERLTRNLVDYEDERSRTRNRSRIGHLCTY

LLTSLAVLCRTSARRDIISFRIVLEVLLFDRTDPIDVRTALALYYCMMEYICEPAKQGRY

ELHASRNLSDISVMILREKSLRVPASAAQLVSARVLWGIYRSLALSQEAAVCRARHVLVL

MLARHCTERSGAETRTWKTVLADPYAPVALLAAEKILFLSHSEEKPASAAAAAAEASADI

TRDIYQEAMRYAGKSGKRGKPPNGYGLLHEDNCGEVERGRRPVVKASQEMALY*

>Lp_000067800.1 glycerol-3-phosphate acyl transferase

MSSSNRSVKFSFAEAPAAADEDTKHPNAAPTDASAGSTETPAEVAEKAHEGGNVGSAAAP

AASEPLASSASPASTADTDSKSTAATTAETPAPASASPAAAAETKSPPPPKSALKSKDRI

GGAWRRSAFVFCEECGQKIPVEEWPDHRDRLRKVNLVEQVSTFHHKVLRFIGEFLMWILR

SVYFREVTVVGRENIPRAGAVVFYGNHQNQFIDALMMNSHCARPVRFLMADKSLHQPVIG

QFGRMFDSVPVVRPQDVPLVPGEGKMIKTDGQTILGEGTKFSKLNKGDVLIWAVPGCDKC

RAQVSRICSDTEVEVTVPIPPEHVLTEPTDYKSSRRIDHSEMYAKVYDTLQKNHCIGIFP

EGGSHDHTSLLPLKAGVALFSLGAVERHIPVKIVPVGLTYLYGHRFRSRAYIEFGEPISP

PEDLVRLFDTDKRKATGLFLEQLNAALRAVTINVPDYKTLNFLYSFRQLYQPLNCTLSAR

DYLRLTRRLSNVIEERKDSPDFVEFRDKVENYSDFCKALMVRDSQAATLKQLLKADSETP

QVALLLRRTFALYLMAVILVPFFLVGLPIGGIVKYFALKRTKQALSASTVKIVGADVTGS

FKIIISFAVVPAAFVLVSLIVFLYTDLRTALVVFFSLPMAMYVSLLILQEAIMELRAALP

LFMSLVSRHKQFKKLYERREDLARQARAMVHKYDPQLEEEMKVYMDMCESDDELDREPSL

FSLRYNMRRRQATNN*

>Lp_000067900.1 DNA primase large subunit, putative

MQTITASDTAASQRSSLGGAVEKPLGATTSADWMTMYEKKPHGNSTLFELEGMVAKRMEF

LAWIDQQLNSPQAKSFDAVLDAIIARLPEERRSSGQTEASRTRSAVSLGYDDTAAAATAT

TASALSNAGNGVARVNGGRRSSSRGSTGSAAGASQVASIVFEPEEDLTSHLLCRFAFCMS

ERWRDWLVRTERVLLTARIKMEVAKSPFSFLVDLMKLNGLPCASLTEKQLADPILQEYLD

FRRVKADGARESEGRAESYYAVPLSLATRLIKKRSVLCRAGQAILFRDQVQEVFLTVFCA

HLNRGLHSAYLSRIKLQSLEEETSKSTVMSMLDAFLEQFIADPTDALQEGVAGAVKAGDV

QRLAQTHFPPCMRMIDTHLRREGHLKHHGRFTYGLFLKAIGLSLDDSMELFATLMKVKGG

GSVEAFAKTAYGYNVRHNYGMEGKKTSYSSASCATILALPPTVDQHDCHGCPFRFRDEGV

LRAMLAKEVRNPKGRDYPNLRPTPGDIEDIVADCKGQHYTRACYKYFMATHPEARRDTLF

RSPYEYYSASLESEMNASGAAAADGAESARSSAAGPRKRTSMMLSEDAVKPRTSS*

>Lp_000068000.1 cytochrome c oxidase assembly protein, putative

MLRRNWIWRCASGVAGGSSVVPPAPPPPPRKDDSAAASATKSDNKQASEQQQQQQQEKGK

RRFRLESGYDGAKNYDERGQFFSAFLSLGIMSLGLTFLFVPLYRMYCAPSGRGADPKFYT

PEAAREREEQNKHYPVPKKLLKVRFLSDVGNTMPIAFVPLQKEVEVLVGEPALAFYSAYN

RSNRTLLGVSSYTIAPPEVTNYLNKIQCFCFEEQRFKPHELVEMPVFFYIDRDFLNDPMV

NWLDEVIVNYTFFNLEKTKDYIFRSNPR*

>Lp_000068100.1 mannose-specific lectin, putative

MLCSLVAALHGGHRGVWTVALFALVLFLSACQLVALAVVDTVPRLTPEQQKRAVTNIINH

HSFSPPLLRHYYGDGEIPHWMISGTTVITDNYIRLTANEKSQTGHLWNTEPLDMNAFEIT

FGFRAFQPFGGMGADGFAIWVAQLPRFDGNLFGRPTNFDGFGILFDSYDNDIRRDNPMVT

LVVNDGSTTKKFTPNNDFLGEGLASCVFDYRNIFAPNMATARLRYNKGTLSLFLSRNNEV

SEQQCFSASNVDLPVGKSYLAFSGQTGEVAEVHDIIFVHLSPLANTTYDHDVQQPAQDEL

DAKTQLYDNVAMNNRRSTEPSTQVPLQQQQQQQQQQQQQQQQQQQDIEARIRAEAERRVA

ELERQRLEAQQQQKAQQQAQQQPTQQPQDAAEQARAEADRRVAELERELAEMKRRENRRP

ERVIEEDDEDDEVDEDEVDEDDVDGDATQPRRRRRVRARRPRRNTVQYEG*

>Lp_000068200.1 Metallopeptidase family M24, putative

MGRGYSASRSKSGRPTLRMKRSRSMSLHALKKSLDAREAAKSSSATDNGEETANAMATME

GPRKVLKVARLRRAPSSATKDNKPSTPVVSKSAEKKAAKSADGRARSSSSAAAAATNAGV

RRTPSAQFRKNLEDLEQRFEEAMEASEDEDETVMNTTTMTKYKECGRVVDAVLELLTEAC

VPGANTKVLCDTGDEEVMTRLKTLFAKTKDAEGKRLTRGIAYPTNISVNEVLCNDSPFRV

EEATILKDGDVVKLHVGCHLDGYPVSAARTFVVNAPATTNQRNDITNEEEEEKAQQPTAS

SSPSSRVATAAGNAIEAARVALLAMMHALRPGTLNADITDIVAAVGHHYHVQAVEGVLSN

RTKRWVPDGMDCIIGRRVTTEDPHQDVGDCEIGEHQVWCLDVAFTNNDSYRITLSEKPVT

LYRRTPAEFEMDARVKQANAVLQEITDTHFCFPFHFKSLAEPLKAKLGIHVLQKKGIVDK

LSPLRTKAGYVTARFSATVAVTAKRVTVLCGAPPTTPIAVPPAMRSTESADTLAPAVLEV

LHRPYEFADVRAMAVKTDAKHASSPAMKRKRVEAMENEEE*

>Lp_000068300.1 PSP1 C-terminal conserved region containing protein, putative

MSSKANNASGGSRTQRYAGGRRNIARRGDGMKSRKGDEQPAAAATAEERNNTPTVGAKTA

KTAKPAKATATATATTSRAEQRTHNSRNAGRPQELDEAKLHRQDTAASTSSSRALNAFSP

AFVPKQYQPSSQQVGSGEVNNALSFSSPSPLAYMGSNGAASSVSSPLVFIASGTHARPSI

TSPTTPSLSEQPTSALLPPHATSATASAGGVSSSSSAIQLRDAYARAAAAKAASTNRDPR

SVSLISQLLPLQQQQQQPSPAPSRGPGAACTFPLGKGLVPPLPAPQPQLPVSQLQQRPPQ

QTPGPPPRSSAHDTVAAAGPAGAMPAASSAVYGPSVSVFHPQFIDIATGNTSMTPFALRD

DYVDDFMEAAAAETAMEGEAVAMAQEIIDGAPQPLHDRQQQQQQQQQLHPAPPRRVNATT

VAILASRNAPTAQLAMPASTAGRGERVVAVPPPSMCASASMAGQLVSEAAEESVAMVTAR

PTPSLSQRRTTTVLTSTTATPASAANVREEETAFCQETPQRQTATHTNGFGDAKGNSSGT

TSSLAVADGGANAVERLYACLANTDERTEDGRSVTSKMGLTGSVRRGAQPRRNARDDDDE

DRSEKAEGKEEGEGVDGVQSTAETPTVTPAKVLAVEGSPFVAAVAADSTTTGAAPPSSPL

PSRQLLTEEDSAIVFLESSLGESMRGSRAQLQNAAASHSTSSSTSNSIANAHSTAVSNAQ

LQRAMMQLIAQIQSNNSKAAATAANTAAAAGAAPKTTTTTTTTVITATPQKSSANSQAQS

ETTPASFKTPEIERVPSARPSASKNSTCRTITTIIHTPNTTTTTNAHSYSNSISTATDAA

VKSKNVTKREVSSTPKNTQRSLASCLEAIAPAAKITTTRPCGSTASEEAESSHVNPNAMP

VPRSQRRTTRHGGSNDAAANASAPINNDSVASNPQTTIIPTTTTHTMRATSPLLTSLASS

APVTQYAVVIDGHMGRRVTAVSRTPLKAGVCVLFEEDRGIDMGRVVQCDLLEDDDAVNAL

PTLASATSPLSRKDRPAPVLRPATAEEEYRWLYADVKEAEATLEPCREAAARLGLPVKVV

GAVYQFNKAKLTFYYESSARVDFRPLLPSLFSRFHCRIWMARMETPAVEEA*

>Lp_000068500.1 Putative GTPase activating protein for Arf, putative

MSNLNRIEMPKTDEEAKELVAMVRQQPDNRTCFDCPQKNPSWCSVTYGIFLCMDCCGRHR

GMGVHITFMKSAELDSWRPLEALRVALGGNGRARQFLKQHGDMNPKSFYTSPTAALYKRI

IDKAVNDFQESGQLPPAAPIVNSMPASPAPPNASGNTSPAPSFGASPAAPASSTMPANSA

STADVTSQGSPITTAPIIAISSKPTGLGTKKLGNGAVGFGGKKKKGLGGIARVEGTIEES

TQPVSADLLYDREAEQRKAAEEAQERQRQADLAAAASRAVDPDTLSGRRDGHGSDQDANT

PQPQPQTAFISKTVENVTGDLFDETARKPAPAPYSPSRAGSPYSGVGSASNAARAAVSPA

SAIAPASTASSAAPRTGPDFSGQGNQAYVPEGPVGGRSSSGGGGVDYQARASEVMWSVSE

MAHNLTQSAASATESWGAAVKSFLDDL*

>Lp_000068700.1 Calcium-activated chloride channel, putative

MKAATATTTAAAPAPSRRRTTVSEMEMKAAKGAAVHVDPARPPRRHTYTLGQPVLPESRQ

RLVHDRRKEAELQRQQRTPSLTQDAADDIPRAVVPRPSISRSSDGPPFTTTSASVAKNNL

ETVDGPMDTPRNPLFKPARGPRETAFMTREEQEKMRMREVAQLNRLEEEKAKPRKVSNAK

EVAAAAADARQQLGQPNYVAIAAHKATAEAQYDEAHQYTHLVTDLHQRLGLIPTNESTPP

PQEQAVSKEEDGVKAAVMRKPRTSPSQVAESVTDKVLALRRAEMAEIVARLRRLLAEGNV

TATANEIQIIPAAFGGVGGAGVACPACDMCIEFTSEEAITHHLIPVLKALTSERVVMPMA

GRRGSVSVTSSAASDSAITLDSFRVHSSHKPSRIADENGSQQQQLMEWSGPSRYAMPRCF

RIELVPFGLRKVSVHISVDDYLTEHFYKAHALRQEQLFSFALDPPYPKDWGEWTAAHRAR

LLYRVVDNLLHGGPHPPAPVVTPDIVLFATHVDAIRDALWNSVWNSRSLRQLLAVDEDAI

AAYFGTEVMFYYAWMNHYARWLLGAGVLGVAVSLLSSVRLVDPAVMSGAYKTPTATLAAA

HVTLRRVLQRCLDVTLLPLFIVVMIIGSVLCIKTWERRCSLLTMKYHLFQQEGKDEPRRD

FHGTPGRNPVTGEPQLVYPAWYRAVVLQPLAWAVVILFMAGTIAIMICSLNLDGMVSDPS

SRLAIPFIRKYTLNGGVLDAAAHPYFAMVPRIGYSVCISLLSYIFTMLAIQLTRMENYRY

RGEYVRALTLKRVVFEFVNSYAKLIFIAFGRSSMPELASNLQSILYVAVLSRLMSDTVVP

FFVTHRRRVARRILQHHPKLKEAEENASAALAVTPAATGVLSAKGGGGSYAGVPETHGGD

GASPLDEVDEMLDPYDVYGDFIEMIIQFGYILLFAAAYPLASFVALLSNIIEVRSDLFKM

CYVVRRPTPRLGLQENGTWCGVMRVFAMAAVITNTFLLAFTSHQMARWFPEYFITNGTAT

ALHYGSLNGVSHLEDASTSAASVASLANRADIANVALHMIPGTGRIVMFYSMVMEHVMGL

VAAFLLWRIPSTPRVVRHYKERKLYERVSHD*

>Lp_000068800.1 hypothetical protein, conserved

MRAFASCRGSVHVLRRPALFCTWVSPLTSLRSCSTASAAGSTSTHDDGSRRPANGFDQFR

QQQQEYHRHSFYEKGSAPPRGSSASGNQQQPNEQQQDHSSSSSSSFTSTSERVKDERVYG

HGSATAQQRRQMRQQWEKSFFGRVHFDDSMHSRFAEALASEEEEAFRQSIEGHTHAELFP

RWPEDEEAPLAEFKRLRPSLQLRYIVNRLSMGERRIRYAVDYGGLSMMYQLNLGELMVNE

AEKLLRELGWMNDDVAAKIEEVKMLADKIKYDFDLD*

>Lp_000068900.1 C2 domain containing protein, putative

MATLKVTVHEARDLPIMDRTTGLADPYVVVKLDDMEHTTDIARRTRNPVWAHDVRFDTAD

LLVLQEDPLEIRVYDHDIISRDDIVGMVLLDCNSIIYRDNPFVSGWFPLIDSDAGLRGDI

RLTIRIKFHAAENPLAPALPERYVRRLDPRVAEQQLPTSSPSPPAMAASHANVANEVSVS

LPTMPSVVSSSAVGELNFLGQALQRSASNPLDQGNVSSLPQHSAFSQNSSGSAAATTAAA

AGGPAGAAATNTAATTLLSPGVPHSKMHSAFASRQVSVAQPSRSQSLQPNSASVSPFFNL

DTPFTALANAGATLSMANSLNPGISGSTAMNYADNVAAVAAGTVPLPSPALLPFLVEQDL

DPLQAARVDHPTLAAEEEGVFVFSVWRLDPAVFRVESTHSMMEELIVKSDPEHVRFTNLR

SSRSINDARVIQLFKLSGKVRRQLARKVVELQCNAVLGYVEEFDIEPNGIIVRAYGTPCV

VSRVKYVDEQEMQHFLRRKTRYLQSSLVSAAYQGEFAQQQQQQMSSAPLSQGASPLLSGG

GGGGDVAGGAAGVRSPISVVSPGGNGSLGGPTYYTPPFTIHPRLSVCGSTPLMPATPPIT

SPSHYTVTATNNNNNDGVSGAPTRDHLLPPATTSDTNDSVMAEAQMPPETSSTAVQSPAT

TPVHGPADPATATAVLAGPTSDNPTTMPDLLVLDDNNGCTAPAAAEEELPGAVNNNSNNS

DSVGVARHNCYDHTATVAGQVRSASLTSPPPSDRSLAGASFGGAVVNNGSTDTAVVSQSP

LQQQQQQQQQSASQVNVVTRSGPPENGGSGGGVAALPPPVAAAAVNAVLQQPQSSAAAAP

LSLAAVDSVVTGPSRVIISMLTVKDLPAGAIHHIGGYICARSVKIVSRVKSRQMISQERD

AWWMELREELRANARAFHCNTVLGYEEETQYYEDVVLLSLYGTAVMLDVSVTSLRAGPEY

LFRLQRQRVAARRNCTLLHLYEGPRRKGGQQGLGLLAGEVGDLLLHHVCSICHNKPVPEV

LLAACTLPVELSCEAPPRLVQVAVAKTKPNAKGVELAMAVSQALPFIEFALHKQLLFNLR

LQQMNACFSLRVSIVIGPDAIIGTLTGSGCRLAGLPVPRLPRMTVVDPLIANQESVVKLR

DVIASRHRRHYSGTRSSSSSSSSSSSSRSSSSTSSSSSSSSSSRSRCSSSASSATAGSNR

LTTPQSALVKKPEGSGDVARTGSTDDVRAAVAAAALQQQPQLFNASSAVTPNTASLPTSA

STSKRRKQHQQQQRQQGQKSKRSRRKHRSRHHKRSNKHSNESSVSSHSPSEDSSRSAEEL

SHSASSSAGASLSSNSNNGGGGGGGGGGGVGAPEERAKKHRRKRRHHKHHRNARNGGSRN

HSHTNNNSHEVNSNTSVAAADEGNVKRTSHTASDAATKSTSTTTQSSATSTSTSTSSRSS

SRSRSSSSSSSHSSASSSSSPNSWVPTEPSESDEDVYAPADEWHSSGGGVRTKANDYVVR

IDDVEEADMMLGMVDSTSFEDGVLLTLPYVPDSANAFDWQERIVLDRRYSRASTPPFSGG

GGGPSIGGLSDGSMSGAMGGYGSNASYGVWAGNSGSAFATGNAHSDGGQGGASGAGAEGG

GGGAAMSHTVAVAGRTALSTRLLNEYCADAKRAFVHRACRIAMRTSQPLRLRVVSLRMEM

MFVPNSADLQLRLEGCVMVASSVGARQLRALEDRAFRVCMHYTMESLSGLWHYHQQQQQL

QLQSHTSHSNANTGGGSSGPLTPAPRLSTSAPLVTTTNVSGSGRATPSLPWGANTPILSP

MAGTDALGYSASNDNPSLSSPPQQQQQQSVAASFVVPHSGSSVQNLAALAAAAAAAAAVT

GAGSTLSTSCLLPLATSGPAYLDARRLFITCNAPFSMPYVQDITLPPPRVWSGVATAFSE

DMAAASRHTSTYAEYRDSRGSRTTSLARRWHAFVRATGQSIKGLVDRATGGAGDRIDWRL

QQQRRWLSSTADGNSTSLRDGAAGSNSNANGPIDPSMAGLRDANYGHPAARLPQNMSQLL

QLIPVVLFTPLDYVAGRSIVRYLGRLSQHFIREDFNTQTSDDLNVFFQRAETEMMCMVQA

VVRLMGGNALLKHRVVYHEICDSDGSGSAFLFATVTGDVVQVSDTEYWGSHTRDAHARRR

HSAHQRTNNNNNNTSREGSSDEDGSTPWNNNFSSRTRYEDVTEGSDADRSSSTAAAAGEA

TSCSRRRKVRRGASRHRSNSSNRRHGSRARRHRRRHRCPTSSSSSSSSSRRHGNASRSSS

SSSSSTTTSHTVSSSTTAHSGAPTPSRGSSVPLNR*

>Lp_000069000.1 Dual specificity phosphatase, catalytic domain containing protein, putative

MFCPDTVNDTTPDVLELVRSLFPTLPSANPAADRHTSCSVVSTQRESGLKDRWSSAMDSV

PVHGHVLLSSLRRVAALARRHELLVQSTRSAWPSPSSTLPASALSSSSVLVRLVLSLLSF

SATEDVRSSTTPTEKRDVGSVARAAPTRHAGSSSSADSAHPPSALSSSMDDDDDGGGGDG

TTLLPWCTVSAPGATDITYVARCVQHETGQSSSSHQCGVHGCEEDGNAPSSASVSVPGHG

GLCCRIEVRSYPVAVFTTNTVRSLSQYWHCVASSAGREREREEVEGKEGGTDAASPAAVD

TNSSDDSSAERCCLAAALADFRACVCRHELRSLRSVAHACERLLLRLDVLSSDQREAGSS

EDEGLEGGSEAQQGGASDAATVIVIPVPMYGVALSMEDNQDTRLERVGHLTTPLLREATG

EQSPLRALTAVLLQLEAWATDAPERQHPSRATPSEAQAAPSRLLSSQATPDADTAKASTA

HLQLPLLLTRARLSRLHACVTQVLQTESLSLLISASSPTFEAPAASIASLDVMKSAAPDV

LLPACLVHCQLGVSRSPSVVLLYYMDVFQPQWRRAAQLQSQARRPAKESSVSAATSLVDR

TVAVEGFYTLLRALVRARARVKPNVCFAVQLLSLWNKCVG*

>Lp_000069100.1 protein kinase, putative

MAQAPLSTVLRHPLASKRCMMVFVDNGEETTEIFELLSRYEAVAPIGQGTYGYVCSARDN

DLVESFQVNPPTEYEDPSLSAEEREEVYDSATLVAIKKLRQLFEHNQPRMWLCATREIQL

MMAFQHDNVISATDFFIPLGGVEMMTYESILNLQCSFDSVYVVMKKMDYTLREVLDSIEV

MEAELDPNYAVMWKRLTALMKDNHDSDSVAMANGNYNNIAAVVAAATTPEKSGEPQASEM

GDGAPLTETKAEDELKVDAAAAPAGDCGGDAAVIGGICCPITGLRLRALSKDYRKFVLYQ

IFRGVGYLHLCPVIHRDLKPENIMLDRSYNTRITDFGQGRDVGVNTASDYEQTVLDNCTQ

WYAAPETLTVAINNPVGFIDHESFHGVDVWSIGCIAAEMLVGRPLFYTAAMGGKAQLLSI

FRVLGEPSAKAIESIADYRDKDTKELFINSIKKLIKTAPPSTSLQFTLEDLLRSPFGDED

EDEVKLIMDCLRWDPRDRITIQAALHNPYFTKDKYDPTIDPDDTAKRVPSVRPEDISEPV

SGRAFLWNLFLQRHPEVEELWKSLVAKHDKEVTAAAASS*

>Lp_000069200.1 MatE, putative

MADSANNTNNASAPLPAPHGELLHATMAVLAHFFPAAPHAPSALQSACVAAESSSSSNDN

APVPTSAGARAEDSGRTTADAAAEKPIEDCGGSHAVDPAPWRYVELPFLSITEQVRTQSG

VITSDDADEHTPLLALVTLVVRLALPVAVTQMLRISMSFITTVFMGHYLSTEKFAAAATG

LTFTNLSALSIGAGFASAMDTLATQEHGRRKHSPEIAAIFLRSVVCTFAAYLPIAVFYFF

CDPVLALLIHPDLVADTAYFLRMSIFIASPMMLVNSLLKFAQSQRVTQLGVVGAVMGAVV

LPPFLFIFRHGGLTGVIAALSIDRCLTLLAVTIGVMRNPGLRHCWSGRSLTEHIKAVLAN

GTALWRFAQVGLPVLAANCADSWAFEVIGVAAASLGATSAAVWNIVMTVYSQLFGGYVGL

AAAGAVRVGNALGSGKGLLARRYSYATALVSACLTVVLVILLWLGGGMIFRYMQNNNEVT

RQGESMTFLVGVTFLFDSIFYAMQGPFRGTGYNGVMFMIIIIGMWGVAVPTSLVMGLKMG

YDVHGLMYGLLAGVAVTSPIQLGFLWCLLPWEERARLASASPEEEA*

>Lp_000069300.1 hypothetical protein, conserved

MSQCFKCKQSVGTIASRDGPKTLYCADCFLRYCSGVMRDNLFQQCFAACDTPLAVAVSGG

PNSMLLLHELGQLRCKAQQQQQQQQQSQEQRHRRAHVATSAQSSSNLELLPFHLSEAELI

LPPSLSASTAPSLVSAAATAQASDTHATSEATTTATSGASLARQAAVERARAYMGVQFDA

IVKLVQQQPSLWVYRNESLDFKNKKKHQQQHRIKNPTSAPEHIDAAAVAAEEAESSKATS

SGVTAGVKPRSPVHLFENSEVRVFQYSDFLPPDCIAELRHALHVSKLSLTDREALYDRVR

QQVLCRAARRVTDEYRQKQRHRQTEEAKSQDDGDTNGVESVSHSEDALTSGRQWYHLLLG

DNAAHCAVAALEAVVTGAGGEGIVHASAFRGFLHEVVCLRPMRTLLPKETVLCTRLQGIT

STYTPALCTGTSLRSMHQVLEQFVHRMILSFRTMVFNVLNTVQKLSVHPESMQELVQLAG

FLGNSHAGQESSASVEHTSQDNGSKNGNNNNNSKNKNAKALSGRTAQQNYRDLLTSPLPH

VCHSTAATTAAELLTRKADFSADERSLKRFAVQCCVCGCPVSVPAQASSSSSGGTEASTS

REVEVFAVRRGSTTCRSAASADGGASSSSPSSSPQTSTGTPPPPAPALDCYVCYACRSLF

EAWPETALTASTQPEETESKAPDGKKDAVFAACSLFQ*

>Lp_000069400.1 hypothetical protein

MADLLVRRKEAVAQARGGASLEWVQPSRPANVRSVFAQGERTASCDVMKDTGFYTTSVLG

GPGYTYVVSLLASWTLNQLTTPMSVMQRYNDELMYVLEHSGCSSVVGETQLLKEKFPAEY

DELYVRGEADVGKVSPKSVQALTTAKSDTYKVDTVADLQSILGEFRKHHAKDAPVGEVLS

FDTEHLDELNPLFRRWYQD

>Lp_000069500.1 Cytochrome b5-like Heme/Steroid binding domain containing protein, putative

MSSPLPPPPPSSPIPEGCDARQLSQQQAQSQPQQRVRLRSASKCGSSTSLNERCSLPRPP

SSSAAPPAGGAAPRGGDDAYTPAQATPTTAAMHNSSHRCSPDEQLIRVEALIDGMPMMVL

PPPALRAQQEGGAPVSAMATPTTMGHAGHVHTKRGHRPHSHHHSHVRDSSLGSSGGVAHG

YTSVSHNSPFAKPRQPAGPPTTLAVGHSREFLFSSSSSLYTSTPQTLTSAASDQMLLSFS

SANMAAPMTSNGSNNTNISASYAMNSSSVGCGGITTAAASQATTTTTATSALALTEGFMM

RPDAETVQNLPRGKVPRMPGCSMRDWSAHLAVKEAENRRQQQQQQQQRHGGGGGSARSSQ

HHQRAGSNPVFSGPSSCSSCTTASAFSSPASQLLSGSRANLPRMTPQEVSKHNTPDDLWI

VIRNVVYDCTEFQRFHPGGEKLLLACAGRDATEVYDHFHAWVSCESFMGPYAVGILAPPP

PH*

>Lp_000069600.1 IQ calmodulin-binding motif containing protein, putative

MSNIIFQYPCREGTSGLELEAVRHNYTRVTPFYGTSLVNSVLQALEDASLCASVELHAAV

KIQATFRMHQQATIFREVRRKACTIQRMFRGYSTRKHLENERATAQKLAYLKTVFDVFAT

RIQACYRGYASRKMRSNYYAQQAYIHTVTARAAAVLEDAHRARVEQDKLRTAESQRVQAL

SYARRTAQMHHTISTCSIPSVYQRPPVSSERLVDALRSTHGIDGVKAAGGRHTGSGDEAG

KEKGDRVLEDYELEQVAVFTAGDKLEEDIRQNSRAARHHRGNAVAPSSTARAATRKGTPP

TSAAAPTPTSQMPCLPTLDAGKQCQKTSMRTSSARDGGECESAHTPAMPCEDAAATKAAA

SSSTRNAAPNASSLHPKFGVKQHDCVSHRLSYQAGVTQNSTAALERSVDQKIIRSVHGNA

VFKVPAVHGRRTR*

>Lp_000069700.1 hypothetical protein, conserved

MACLSSRSFTEDVQLAARTRAKQHAANQRSALPPAPTSSQLLSILNEAAQLLTPTCAADT

ALQGRRNDGEFAARLGGFPGVSPSSDYAAYVRRQCPTSRRVQSSFAAAGAHSLLGEMLQL

FALALTRLFTEGITSASVVSFPAEWDRRLNAPEAADLREKYLYELQGFSTLFTYVWGNVA

AACPCVLQQQPLQLPDEKDVMGEGNEQKGVTVLRASPAAATDAAVPVFGSNGRSPAPPQL

GNASLSTSDPTAQQTLPVVPSARSWQQACLVTAPSKLISFHSPSTASVAAGRTSEDTFTA

SGQRRQSAPQSNARPAAKEAIAPSHRFTSQGKRRGHFLPLLPLEGLVASGQLPAGETKQS

GEVVERADKVSPQAASTLRTNSPSRDAELPQLSRSARQLAIQQAMEQDREQELKRELGVR

RVRNYAKEEDMLEYVHAFQQNMRSVLDEDDDRGGEDADSGDVGKRESRLSRN*

>Lp_000069900.1 hypothetical protein, conserved

MSFRTTPRRLGRPTVTATPTASLAGSSAARAPLLQFPAFHPAPFQHDKFLSIKTRSLETY

TKVRGDVLRRCAELRSQLQRSQERAKRAGDGATASPSSVMSAASLELPTAPLTPHQAEEL

LLLSKKVESLADFQGMVLSDELVWRMFHLCVQCGAPQQALNSWLLKHILAEKRGPPYPLF

IVQDLTTLLRASVLSRLADSGAGLSCISSSLLAGGEGDHGDGRVALEDGLVAAQRYLRLC

SVRGEGEATAEVDEDSSQTAQLYDAHLLQDYVWPVWRSLEAMQLTLDTLKSKQDTAFGDD

GDEDHSGKDEHGGAPERDDHKAGGVARDLKRTAASRWLPDTAAQTAAVRATLMSVTPLYS

FHERASSDSAVASASPSAGGKAGKKSEALTAHGPPSFSSEVEAIAAPLRALLDVWMTVAQ

CASEAKDTRLLREVQRTVCVGVFKTASSVGSAGSSEAETQTYTLNEAAWCQYTSSPSAPS

SDLSCAAESTARAVFHSFVSGVLSVVQYGLLCAEQERGLWQLHDTSALLLGGAAMLRSLR

SDQNGSSKSNSLPRRLQALAVADQRNAEDAQHDLLCNVLPRALRTTARVERNSADDAAMM

EAVHIFLSQTLAVLTSTGATATRPKVPGADTTLRYADAGLYVGLAMGDEALLQRAAIAAS

MPEGDTEMVRLTAHALNNYRRTVAFLAQDAEGEAVPTPSSAAAGESDDVWPRMQALLAQS

RGASSAVVSPQLLEGSLQVVLLDTARRQAACAAAYVHALDSSSSQGTGAASATAAAQDGE

ESHAGVGEAHEDDAFSLSADVDESRRTAAEQAWSDVEERSVQNIVEGVERVALLLNGSAD

LASSTEREAPCLSPSALSSIAVLARVGTYVEREAGSSATAASSFTASLDTIISRLAADSC

TLLQQALSSPCSPASLTPDVGAADVTTAVAGTWAQWVLLALMARRDWTGVLAVLKALDGH

ASEQRRASKHNAAGAGVGAATPSLLCSATVDPAVFAAIFACAQEDGAASVCAFLRPRRES

LFF*

>Lp_000070000.1 hypothetical protein, conserved

MRCSRLLCTATPERFTILGTTFPKPKRNGMGRENKMRSKPSDNVAWYDKGPVEWLPRPVR

LNYDQLDQLRDWMMRETIAGRTEEFNKIRHLHREWSQHPLMPMLGDVEPKFPLNLYKQNH

RAKHRFLVRWHKANSPTYWMWMPRGPAVATPLHRSSPSQFPEQWRQLARNANGTVAK*

>Lp_000070100.1 Amastin surface glycoprotein, putative

MPSDTSKPAKSTCQKVLDVLCMALSLRIVLVLIYLVMLILLCVIQNISLFSSPKIDIVNI

AASRIVGAQVTKLSETWYSVDIPIYGANASAQVDIKMWTLSVMLDIALPYGYIPQVIKQE

YRVQDVPCKEFRTVFKNMQIFSLLSIFTGFIVLVLTVSNFFTRMFLPLLWFFVWATVAFT

ASMTAMMFRLLCYGECYGEPTAIPPFTSLAMPMGGFALSIICFESYLITSLMTVFL*

>Lp_000070200.1 Amastin surface glycoprotein, putative

MLFVRFLVLVLILIFFVLALVGTLTLPLYSNKITGFDQNGKVRVTLWEIGVSKVDLSGIN

ATKVPSSKAVRINYAGCSSFRATFRAMEAFAIAGTVFGFYALLVSCLQCFCRLKVKLPLF

LFMLLAFISEAILIIIGGVAYGTVFCKNMKNYGNLTAVIFKGAGYKLDSGYIIEIVAAAG

YFITFIITPFTQQLWCGNC*

>Lp_000070300.1 DNA-directed RNA polymerase I subunit, putative

MLPVTSPQLVTPLEQRQVHYVSDNVFSLGTLGCAVCGQAFPTRTNAWQKATCTCCGTLHE

PGTFTAFQRQQCTPHGVSVNHKVCSTNTAAAMFFTEEEVRQATKYITDALGDEFVSGGNG

ASSSASPTAGATGAITKRAGQMEVDNRVIEDAFCETCGVHRPCKTFARQTRSADEGQTIF

FQCTKCGSEWQQNS*

>Lp_000070400.1 hypothetical protein, conserved

MLADEANPTAAPSESTSPVSLAPHPPCSLLVRSGVNSGKAATRRFTTSPAATLPTSSPTA

ATEASPRVVSPTASDHGTASTAPMERVLQTGQPTFSTKLDFLTVNRTVAALERAVERLEL

LSLLDATGPAMATKSGSPAAATSATKTNAVGTTNSSDGAAAAAASFAAASTRGAAVLTGA

DHDASLITAQKVSEALAASQQQGSRGRPSVLELLSEQRLLERRYGELLVQTQPVVSLHPG

EPQLRKQCFGNVQDAQQTALQQELAQVSARLRDTNRLLCTQLQDNPQDADNWAKVSNERR

ELTALLKEVIAELTMGYKEVFQHHQQRPRKGENGSSCGAGSHNNDNRLSSVNVQRSHAAN

DVGGSGGDNSATDPSAAHSIAAAATTSATAHTSSRLSSAAGGGANSETGSQQTALRRFGS

TMQRRRMSRSTHQGPRFPLASSYHEFAVKVLQEEAARQWADGVLAKERALNQNVKQLQAD

LVRERELKEKDVAERKARVSELQLELRRLKAAMQQRAEAAKARGEAAMEGLQRDGAAEIN

DVRQSAQHNERLLTVEADTHSAFAEFVQRRTAVTDTLANEWEAKTQRELKKKEAAKIDAE

SSRKNCAQRLADLQQEQSVQLELKKQREAKTKAEEEERQRAADQRAMEYTAASVLEAALK

AMMTRQTLAKQKKGSKKKKKT*

>Lp_000070500.1 hypothetical protein, conserved

MAKQRKDHRKQQKANVKAKIGVAHKSVTASHQHHPRPHAEGDHATPQSIEEVARLFMGNA

AALGEAVKANAVTAAAQSSSAAKRPRDAAVRASPEAPETKKSKSRTSASTPKGDSGTSIP

QRLTSSSAIDSNAKKQKKQRKLTMLSATKDADADAAELEENEDVALSRAVQARALSFLQH

LNPAKSKMFWGLRDVAQGKATKGFHASSSSPAFAAGPSPNTTTEKAGKKKKNKNNHALAD

DGEEIWRVGGPYVPNDEEEEEEVGCGLRNRQERGKDDSDAESCSSATSSEPSWVTDSSED

SDDDDEVVHARDSEEDGEEESNEGNNKSGGRGGHKGGGRLFQSGDDIWDDDDD*

>Lp_000070600.1 oxidoreductase, putative

MSTPSHFKKLVVTSLSKDFRHSTEVVEAHLPDEVPEKMVRVAIKYAGVNASDLNFTNGSY

FKNAKLPFDCGFEAVGTVVKVGAGVSNVKEGDVVTVLQYGSFAEFLDAPAQTCVVVPALK

PEYIVLPVSAMTAAVALGEVGHPKKGEVALVTAAAGGTGQIAVQLLKHVYGCTVIGTCSS

AEKADFLKKIGCDHVINYKTESLDDRLHELAPKGVDVVYECVGGQTFNDALRHIAVHGRV

IVIGSISSYKSGQQVPFSHPSGTPLPTLLLVKSASLNGFFLPQFHDVIPKYMKEFLSAVE

EGKVHLFVDKKEFKGVSGVADAVDHLYTGSSYGKVVVQIQ*

>Lp_000070700.1 UDP-N-acetylglucosamine-dolichyl-phosphate N-acetylglucosaminephosphotransferase, putative

MTHHGLYASFHDTAVAVTAHAPVLATITAGSVVAYAATVHYIPRVAKTLFKCNIYGIDIN

KTTPEQRQVFAAKRRSGQTEEKEFQRQAVPESLGILAGAVYLSVVMVLSICLGYFRDEAG

DMHTSLPGPLMTITVMLLLGFVDDVLDVRWRHKIILTTIGSLPLIMTYDGSLSVLMPRLF

FDLPIVNTTKAWLLSFSTPGSEPAPFCATLPSTWLPYAIGQSIFEVRDGGAVLVYLGALY

LVYLSMLCIFCTNSINILAGINGVEVGQSIVIAVASVVYNLFQLRLEVQTAVSLGASTAE

AVAHRDMTSDHQIRALLLLGPFIGVSLAIWRFNRYPARIFVGDSYTYFAGTVLAVSGITG

VYSKTLLLFFAPQVFNFVISLPQLFGVVVCPRHRVPTWNPKTNLLSNSHNYTILNVILYY

AGDMHEAKLTWAVLKCQVAACVMGFIIRYVLSSFLYDEVR*

>Lp_000070800.1 GINS complex protein, putative

MSSSGKDSGGVHLAVDSLADDRAFNNYHMSSFLAMEVPATIVPRFSMDRVDCLGGSYGPF

APNYPVEVPLWLALYLRQTDTCAIQPPDYLRVEYLRDVIERERVNDQGFESLPFYFYEIA

KKLTERGGGGGGGSTSGGADDGDVIPNVVEVIRLVSEIHAMRQQKLKNLMTVFEAEGSPM

FIPGVLLTNIVCHELHFLRTSFAIVLQQAATMERERQKVVRLPIVAASPAAGGFTGASGS

VSTRTTVGATSDENGGASALRDGVSHSQGSALTTATTATTAITPGGATQQTDTSAEATPL

VQPPVKKRRTLRQT*

>Lp_000070900.1 hypothetical protein, conserved

MDLTGSAAAASRVLRVLHIDACHRLQSPLRILAESCAVQQTHRQKVQPGWWTGLVDFRAS

PAAPRRHRRSGSLEDTDGHDDKGMEGAELKGAGDDAAVSAMTRGVAAPLGESNAQEGASA

PLKAGPDNDDDDDNDAAMAANAASSDAPGTLVGKRHASCENASTTAAVALDPPTLWCALT

TLELHNTVLHGVFGQLGVLSNLQHLTMRQCSAAAAALDRRDRRQPQQAWFEGLEHCSLLH

TVLLDSCDAALIEGGNMRALAQLPSLQSLSLHHARVRDADVAAFVNAFHDSRRSGLLPCR

LHQLSLKVCSRLIHMPAVATLTSLFVLDLSDMAVQQNFVDQLGLCASAAGRTHALQVLRL

AACGSVRDANPLADLPALRQLDLSHTPVTTAGVAR

>Lp_000071000.1 hypothetical protein, conserved

MGWSGLSDDSGRTSAYANRGGSFPSSYRRRGVGGYRSGAGVGGGFGSFYGGGLNSSALGA

YRSQSHSTDSSNRGTGTARVAVVGVSQTAFMNWWAIHAAGFQVSVVVGVDDRRSCTDGDA

AGADATSATVAVSNEERSASAETARAFAVRCTQFYHLPQRPATSTAAAKTAGAAVTHPSS

SAAPTTATTASQGGSSSSEVDVEKGEVLSVRVRIADDAAASASPPPTSAATTAVGAVSAK

PVTQPYSDAETTKDTTVEPCITATAWSYFMKPYADAQKAGSEEKKSNLFPSAAAATSTSY

WSTRSTMQDNSSNVAAVSTTTTTTTPLVDDVEIVYVASLQGTQHESARHSGVRDEKAAAD

EDAEVRAVLVWLIGNGKHLVVDCALSSETLAACAAAASTVTRGKPEMQHVVLYRGGGVRR

GWSPAAMGQLRNALAVRRGTGQATKDAKKDNTNDKKTDNVPNAAAAAPAKLKTAEADDAF

SVFDFLGGGDGGRAFSGALKAAEGDSGTSNPSTSASNANTTTKETETREGDKTDAAARAA

QEEAVKAAAALPVATSIAVDPDNGVTGTVRQLRFTVRSSSGSGTGQQGIYGVPCASSLGV

MDTVGWDAVAWLLHLLDWTCPDVVQGRVVRRRAGDQAPLCVEAELHYSSDGTVIGPEEMQ

ESNTHEEAGESRQKQPVLRVARPAYLRVLLHVGASGEGGVGAGPGAFQQLARVVGTKAVL

TVDHPLLPAQSHASLAASTMAAAAAAAGASTPPPSTANSSPFKVTTTRTMGLGGATSLSH

GGATAELTTTANANAPILPAVPSVVYSYVVATQDAPPSSLQRVRKEQTITVPGAEGGEPC

AEVRLWQHLRAQLNVTATTMDASSYSSSLSLGYGGVGGSSGSGYRPFGSRYATRGRYGVN

IGRGSAAGGNYGSGRGGLTAAGMATPRTVVKAELTAPEAAAMDVQRAWLVQMVVEQILAS

AAMGC*

>Lp_000071100.1 spliceosomal U5 snRNP-specific protein, putative

MSEVLTLHSAWDVDRHIVLDSAEKLVLVRFSSYTSSSDEVEDRLQDRGSHTMSDATVKQR

KRRRSDEDGGDGAAIDAASLLLQDVDEHYLRTRQMDALLQEIAPKVRKYCTIFLVDTREV

TAFNELYELGHDRDPFAVMFFYRNRHIRVDVGTGNNNKVNFFAFEDIYDFLPIVDAAYKA

GKQGRSITSCDKKFSTVALRR*

>Lp_000071200.1 surface antigen-like protein

MSTLVRLLTLLAVVAACVVVPVSAAVPSHLEEKLAGRTDIQLANQESAPYPVNKNNGDEE

GSAYTDCTLENGVFTIQGAKTMYADTGRPTGLVQVLRMTVSDGSVVVTGFFPVVTLLNFS

NVKGTVSANRPLIDATAAAFDKKLEIAVIDSSVAWSAAETLPSMQVLLGIPATLDGASSV

FVLGVHLTSASAVVKAAIASGTLNMQVVNSSVVAVDYVNCTTCANGIIDVAPVPIFVLNH

SMIRVSHITLNATPTVSIFMTNLSTVTVDSTSLLVVENITARSSNIFSSSVSNSGSNSVV

LRYLHINSIGTALGSEATYTDVTAESGPSDLASATHVEGKCPAACLNGFTLTNAQLSCNC

TCNSPYHRNYCTAMNDPLASYNPTGCTEGCIWCHNETACSMCSADYTLDTSTAVCKRNSG

SCDANCVKCGASMCMECKDGYGVANNGVCVRCAVDHCKKCNNGYTDVCTECMSGTTLISN

VCVPSCEEGYGLVNGTCVKCADPYCRHCDINPSRCEKCVDRMIPDSVTGKCINSGDCSVA

NCKVCDATSAVLCNTCNDGYYLSLNRSACLQASTTTSTTTTTATPTAPCNVPNCLTCYPN

DGNVCQYCRSGYYTFNGQCVPIGNCYVGNCAQCMLRDGTKCSTCRNGYFLSSTYTCLSQH

VNVNGAAAPHSLWLAAVAVLLASAVTHLA*

>Lp_000071300.1 Multicopper oxidase, putative

MSRTFRWHVTLLLACFAAAISLSLCAQLTTPQVIAPNKKGNTKVNLTVRAGRVSIPLEWK

GGKEVFFEYTGRFYEIDESGPLLPGPTLKVNPGGKIMLTLVNGLGAEGSKNAMMVMNAFH

GPNITNVHFHGMHSDPRKDDPFTVYGPGESHVYKISVPRDHEPGLHWYHTHSHGATYYQL

MGGLFGAIDVGEGDFMTTPVHPFRGWDSQLLMIHLYRLGDSDRCDGLSLAAMDTEMGSML

PSDPQFVDRKGNTYSMPPDLFLVNGQHRPTVTVHRDHPMLLRMAFAAGSCYLNISLPKQC

DFHVTAIDGVQLRRTREVVDRWQYFATATRRSLAVVCREEGTFPVHHTDDGSDVIFYIKS

APPRNDKLEASFPVTLPKYSPDYLFLGGSQKYYRDISLSQVYRVKTDPNSSYYVVGQGVT

RWSGSTESKYYYNSFPGRVGTNIASYRSFRGFAVPLFSVVTARLFGDPTDVRPHPLHFHV

NHFQFLSFEPRVGGKHENHTMAMYGVRSGDYRDTIPILDGVTLIRWQASTYTGEVVYHCH

DVSHADRGMMASYLVYNPLDETGKAIERFKTPTTTAKPQLGLHRSHVYVLLFVLVLASAA

AVAWRMLRRRRLESMADVLQAIDVHTGNASPGESAPLIPRSA*

>Lp_000071400.1 hypothetical protein, conserved

MLCGRVILCIFMMLFTACATCAILFPQFRLKATVTTGGSTPSEVTKSIFFWYNETLIKVS

GTTYITRHYSHALTCDKLRTSYQVQAALSVAGAGLGGLACLFTGCWTSAGQKRCLGGLAT

LLCFLAFACCCVSLTLSACSFKSTFCESSQTFEDAGLEQVEGFGLIAAATGGFLIVTVVE

LVAVCCAC*

>Lp_000071500.1 hypothetical protein, conserved

MIYTRVILCILMVLLTACGVCAALFPLMRKTSTDAMGRKVKHEVYLWYTESKYHVNKMHY

YDRVYFRHESCGGFRTTSVAGAALNVAGCGMGAIAAIIAAVHVYAKVKFDLCCTLMVLCF

VTFGLFVASLVTVVFTYRAKLCVNDPVKVRESYKDQSYKLVEGFILLCVATGGFFITTFL

EICS*

>Lp_000071600.1 hypothetical protein, conserved

MSCVYRVFVLIFLMLLTACATCSILFPIFRKPKSKVGNTTTKETVYYWYSETSHVDSVST

SIKRVYTRDYSCKSEKNDYVASAALAVAGAGLGGLACMFVACWINAGYRAALGVISVILT

FLAFACCIVVVSLSAYLFVNVPCKSDSVTQSVKQQDYKLVEGFILMAIAAGGFLIMLVVQ

IIGLCCCFCCHSNFDDVSSDLGSRSSKSRSDDDDFNRCSSHGRSFRN*

>Lp_000071900.1 hypothetical protein, conserved

MESLPGGTELIYPVKVGRDMIMATGFAFFQCLFPVKSIAKECDEMMAGGLKHIGRIVHNC

VTACWSEDPIECAIALGHIGTEPIQSLSANLPSKLFFVMYEFWESTLRLELRRERLTVIE

TCVPRLHTMTDAARAMVVKRVHRAAIKRKLSEGAHARKVKSAADKHSPCEDVEGHKTRSG

TGMGMQSHEPYADMEDNNSHDDVAGMQRRLRREAERDKLSQGIASLVNPAQPQESQSGPD

QQNSGVLVMQFRTLLWQRANNLLRDPVNNYLSALDAVLEGLGKHLNPHEGAEKVQFAV

>Lp_000072000.1 hypothetical protein, conserved

MEAVNISTVLRSGESDTASSLTSDTTSTETTSAAVEKSIDASALETHHDVLDIQAKDILR

NIDHGIYTEEDFIALFGEEAIAERRPLDGHNDPHNDTLSLRTYWIALFTPQVYMAIFYAL

RITFMVALPLGILARHPNVMKVFPAHVVIPLWGIVDCRYTFGEQLASTLLPFSVLSG*

>Lp_000072100.1 hypothetical protein, conserved

MYGCGSGYTGPSADLLYGVGGGNAGGAFPGRGYQLMAASPPAAANNPYASPTERELEQLL

AYQRNRQAADMAERERLMLDQQVNLVLPARQAELQQYQQMLKSQLLQMKLQLFANQQSQE

QGANMDMAYLRNEMAAMRGAVAALSPQQPAGAEKPCRRRQQRKAPRRALTAHSVGNRRSP

GANNFTQRPPRPDTYGGEGRRAHSSRHVDRNTGRYGQDDDDDDYDESWSSLDDELRSDDS

DGGDGVSPRSRRADRSEGSFASSPSQQIRGMLQDVQKELKTLRAMTMSSRQGMDAPFPTS

STLEPGLHLLKLAALEGVDMSIPLDEVDVTVQAYYMSYARDEYLLEPSVERTFPRRPPPL

ARCGAKALLFTVSPVEFIVHQPKEMVVFVLQLQYRGRLLWWATIFSRSRGTFTEGLRDSA

FDIAKALQSPQGLVPGANVSGFIEADSTDTLRRAESFLNQSIGSSAAMGFPSQPPLPPSL

QQLPGLPPLSPPPQGLPLLPGMPGCEGPFLPPTADVNAMVLQPSKGPATRFVLPPPTGVS

ILEWQESLSKHLKKMQKPSPGPAAGNGAAAVKAVTAAAAAANKGNKPSSKTASPQLQQQQ

LRKPSSPCKTTPLPSPESSSSSSSSDEEMIEEQPSGEEEENNDSTSGGSHDDSDGALSSS

PKRASAAASNPTKHRVAHFAEQVEMSSLSPSQPVPQPDPATSTAESPQSQTAPESSSAAA

TVDAESPHSPPHQAHTPPPKRNSSSAGSSPPPPSEKPAVAASPPDPMEAKMPHTSPRNSL

THATAPLPVASSKAQDPLSLSSGSQIAGKVPRDDMPLVERQRLRPILDAHGNLAVPPGYK

INPSNPPCVNLDKFDAPTEPPVNPCVLLGLAKPADRPPASPKAPGHGPKGTTVDVFLDGM

TGLPLDAVCTRLLVYVTDELDPTSGKPMNPLHHPRVFMRKPDLVVFQNLTSSSVEPQFAG

KLSAEVGDRTFAVVIVEFISAAQDMTLVFGHCCLPINKRFFAGNYLCRVKMGDPRRSQER

MVTDIRPAERVADEQRRATEKYNQMQLELSVDAQALHNLLPAPPRKRAECTTLGYLIWRL

DSTNIKAPFFEMPQQLPMSQAELAVFEGRKQHKDTTPAAKGLSTLKAADNAFLDAGATPN

DALGRVVPFSKERGAFVKVEGIRGVGDDVAMYVVVVYMPKAPPGRQVCYTMMPDWLSDVG

APMYKDAPFIFTGITYDTTNTVMFMLLKLTNLAVSAEGPARVEPLAWSMNKLFLEPANTI

RQGRFALPWIAGGLPPSVVNELLTQRIEAVYMRMLKGGEVNFLTPHATLIISQGDSACIS

ALVDDSPGRGQPRQLLIPATLKKSYPTVTCEGMVGCTLRRAHEKCLGGGDPRAVLQRINT

AVQEYLKKTMPTFQEM*

>Lp_000072200.1 hypothetical protein, conserved

MGSGCSTDEEARHHRENKKNRSNPTESYDTWQQKMLLDIGADPLAVVRDFPRMIVYVGTE

KHHNYLPQGCEHVKGSSPAAAAAAATTESARPSCQPSENPLAPSSSTQHQAEKENRKGGA

AGEEARQPTNTSSGADSQDHHRLHNHRHCAKNETDQGRRPLWIETATGSRHHSSNALGAT

QGFSPPQPPPRLGPAGRPSQREPFRDDDAFTARMRQVREEVLLLSELCEERNTFGVAFEA

AWDRLVAQGSNNDGAMESEVVSEYLATLQPQQRDGRQPTSTSAAVTAASKRTARHLVSLD

VHQVLLNACVDSGMLAVASEEACHTAPTSTTLRANVGGALPGLSRTSPFGTGRNSRPQSG

SASAFQLPETRTAAGLLSEEARLPADYGARDSAKKTTKKKLRRASNREGVMKSSSNSQIR

SVASLATPGTPGVVAGMASRYVVRSATFHLMQFATQGVMFFPVQRLKHVLWVPWSSHMQD

VSWTIHFSLKDATPSDLIALKQHTLNALYNNVSTVLPTEASAMDASGLGNPSLQNATSAV

LTANRTGSAMEQNADSFGAFATTSIVSETELRAAAAANGGAGNTVQSAANTPAPAAVEQK

KKRKIILIQHVQTGRHYVEEAERKTTPRYELDWACSIRVDQETLQEVFEPFQHCAVRPNR

KPSPPPSPSNGATSPPASVLRPELKSEDRIGSGVTGVDSFASSPPQPQREELEENAMDVE

SAAATAGFVEGIVSTQQPAVLQREVLAATVEVIAARVERPPRTMCMVSSTWKKRKEELDY

VLEQQYSVHLEDADELHKTRLY*

>Lp_000072400.1 Tetratricopeptide repeat, putative

MSDTRKRTESLSKIYSKKPISEGYNLWDREYLLAAMRVFTFKAEGAPPFVLAVYIDALAE

ITLQMQDLEDAAEQFAIAAGKYQLIDQKVLADLMRFRVTEVSSSAEIALEQVKAYLEAQD

PQRNGGGEDSNAQTKSAFARVYAYLADLLLRSVEDDAPAGPGAAPRATDALVQARAAAEL

AVRLGWDRDHCGYLVLGDVYVAIGDYAKAKEAFGKALEKCPHYAKAIERQIGVLQQLAHA

AEERGDDAAHSSANTELLGLLNRSIEVHPLANTFREKAFLLSEMLGDEAALAFVADSLEH

PPQEEIDVVGSSTKEMMTTLLKAKAAILADGDQLDAALEAAKKALEVSPNDEEAQSIVRD

IRESMAA*

>Lp_000072500.1 hypothetical protein, conserved

MRSQFPSSHLDRRAPSSTHRNASKSGCVHSGSSDSCATADEAPRTSAFTCVSSVASVLLS

SPRQRFILFAVALVVCLAFVPEVLHITQAHAAATDITCPYGWSLYESDHTATTITRQCVR

VFATSSVPAKSAESACVAIFTNNGEASHPRSADGVTPWPFTRHVTVHGAAVRTAAENSFL

QDLFLQAASNGLSDDAILILGGVAGYGVVNWYADASSVSSAGSAYTNFADDVNWQLHAGT

VVMRPSGAWGLLPADSVTAAAAFPYVCAFDASGKADLPDSSSQQSSSGKGVASSSAAAHP

PSAGSSSSGATPQHGNHRARRPAVPQPAPPCLDGWTPLPASVDTSEGVANSVCYFLFANA

SEVGGRLGKTFASAQACCSAAHPKATLASVMSREENAWVVSALLNQFPPSEEACNCDGQM

RPTSTSSSSKSARTVFLGGSYMRDGTGAGLIGSWYADQYLFNTSEYASTSPSSPAWPTER

NDYFSNWGKNEPLLSYGVVGITAPADGCVCNTSSISKDRGAAETIPSTAEAVAGIWVGVD

PRSTQPFLCSYLNDGSTFPPTPPSTTHSPDSSSVPVPEPSAYCIPGWSYLEMTNSCYRVY

PTASTMSPAAVTAATMEESCEQVLRGRVVPAKVGRVHAVSMMSTAESNYVGALLQLYLND

PANHVNTTSVIPVSLAGVSFIDYGESGFYISGVRELDPQLALSLWASAEPAFVKGCVAVD

VGGLWYYVPCSTYTSTSTPENTAVQVQAYTCQYNAKDIDITKDVIHATTTPEPSWEPGDS

TEGSDGAAWLPLHERVTALSGATHLVVAAGSTGIYTLQGPNGTTPAVASVQGLHLYFAPL

SAYYGYAAPTTAGDGTALGSIFAPTQAPPTSVQDLLGVSTDVMFSRPFSPCDGFKDQSRI

ARVRRNSITVHHGNTSEVGYVCISQDGRNYVPTAITYEVTEVTVHGFIPALSRHRAASAA

LASGGRPTWLPPLFCSSAGAAASACAVQQTLAIPQHGTGTVQLALRYSSNGGSTSSDRDV

VATAQSPAVDTMISHLRFSASADCMGAEVPFYLLASEIALQHASAARYTRRSSTSVPANV

TRTAAQYSIFSYQYAAVRAARFSSLRPIFNSSAAPSQLTALQPPAEYYVCVAAQKSSVVN

ASSGSLTQAGAASRRLLSMFNVSSSNGTTLTPAAWTLFSSSAALASESPSSSSSGSTFDN

SAFLLAIADRLLARTSLSPTGAAVRTYTAVPGLKVHLIPPSVSIKGMWALPPRRLAQRTS

PSNDSSGRNSKDASMFAADVATLVPTAATFNRSAHYHLRPTYAYTYTSATAAAPPQSSHD

ASSPVASVAHLHIPQYSSPIVLLDGHEVQPGMLALLSQDANRCNRLQPIPRSDPTATAST

WPSSLDTMVPLSTVTMRVESPSGDDSSSSSSSNSNSNSSTVVTMAYMQLNLNHTGLWGHH

PTRTDDPAAMEQTWYVCLAYNSLSPFMPLREPALKITVHRLHVERFAIDASAAFTSSDAA

AAAEAADASTTHLNLNTGFAGMLWMHGLDVRLWATTTLHSAQVAFSVVLPSSGTPDDTAT

QCAHPSLFYSQQGLGLLTQDSSRRRGRYNSKKRRSASVSPPTDFEEGSGDDDDVDTDVGM

VIPLGFFTRQSMRRMQLCLSVPLPFPTPAASVAAATAAAGEGVVPQHRFFAPLVNTTLDV

RPIQLLYMGRYSAVPASTPTPLEPTASATTSGTVIRLAEGARDVVLPLTGYGIRPDMTMF

LSTQLCHGATTTDPSRIVASQSKLNISVVSLYDLPSSYWDTSADGGDDGLSSSDDGGRAH

RPRVFVVLRNDDLLRKAAAQRLFREKVIIDPLTGVATVARVPLNVCLYAPGTATLVFPTD

FQLVVDPPRVTAIRNLPSDAALLARAAASMPVGELSTLTPTAATVVYGGNMDLLVEGFGL

DDTNTWLMPAVDCSNASSFLWRAPVRLSALPSSHTTTIRIEKSTGALANPLYANASRYVD

PQTSYSSWFATLQQQETVRFGADDGVSTGQVVRRFQWCVRIVENDGTATPAEDAVPYVAT

HVPYQLLIPAVKGFALENARRGAAPSTALTLPTQHPIKGRWFPMQLVGPVVDVMHAIGSP

LYMFLAPEAYTCHEVKNSYLNRGLYTFGNASFAAVNGSAVLLPRWLATAGAYRLCASAVY

PTLAAADEGVEAALQFFTLDGLRVLLIQAVYGVFALNHTTTVTVEQGQEWTLPISGSMLT

NRSMVRFQTSASKCATPMTATRSAGGGVLPDIPIQNDFAGFPLFGEDDASSELEGGTSVI

VNGYYIVLSRSLIRALAAPASYVLCLQPQRSLAWRVAPSITLHVVQHVRHPTHYNYFPAD

GSEDVVAVTALAPTKVALEWNTGEKNKVNIVGAAGQQVSLGLWCFPTPNTATSNSSSLGS

RSSINNSSDGGNSATGVVEELVPCGDHRRVMFVKPLDNDEDPCFVDAEAVPQDVLRGPFV

VDFGVLTLPHSLSVVDSDTDALVRDDSSSMGAAAIVPNMTQPWIMCLETGPERWREPIYP

MLQLQYTRAIPTGFRLGADDASNTTAPFPAHKPDVEATTTAEMDSATGMPLGGNTTLYLY

AQDSSQVVYLNGYGIERGHKLRFGSFCEEEMLPPNIDGSSSSSSSSGSSGSSSSSSGSNN

SSSAAAQMSDYLARVANRQVLENPRLYAEPLTIEDNNGVFLVPSTHLLRSEDARSGSRGI

PVCLSTNDGRTYKKTALMIRVLPSRLRTDTETQYRNLLAQILSNTLLVPQRSRGSVDMSE

LVKVAKGIKDASSPSSDSTITTASEHSSSYNDGDDDDDGATVPTSIPTVYVRVGTQVLFS

ASCSVNTHGLPLLTVNMPNIITLTEQHTAAATAERLLLCFRGVDAAASAAAAQPADANYT

ENDVGALTPAFLWYGLYYRVMGVEVDQVSLHPWAPTAAMAADNVNHVVLRRGVEEDLLLP

VMIGATSAAATTSSAALSPEDQLLQGIERSPSAMSLLSLTRLYIGRPCFRYSSAPQPPTA

VSPFVSSYAGMQLSFSADAVTVGPLAVDESASETSSAAITATTLNRTLVAALPSGDDLPV

PQFPLKSRGSVCFSVDGGLQYLAVGIAQYTEEWAPTLRVTVDVEDNENNEDVAEVDDEAD

DLVTRGTTTALRVYAQSSTTRRLMDTRFVDPRRLTNESGSILQVLADLGRLTTTMLWSGF

VGANGFAMDGFVYLPAASKITQAGTQQPQQPLHFSLDNRLQNIATNLTLTVVPWSLTCYG

TATQHDVFVRQQDVGLYASTAPGVCSAGVRDGARVRVVPLSAAACDDPAMVDATYVMDLS

TVQPGSWGVVTSTSSSSSSSSSKTTSPSSTSPFRAMQFPMALRYVPEGTYALCLRQDLPF

VSTVASSPTDSDEAADEVNRAIYTAVYTPTPLRLHVSYNWTILSISGVSIRADEQGSNGN

TSTSSVVALAQSSNAEVAVTGTAVDVRGVPLLLAFAPGSAQRQQLTRRRLSATAATDNSS

SSNMEDGVTSESWTWSDGCAGVPAAWDPETGASLIVQDTHHNGTTVSGASTGKLLSAPWY

NAQDGVWKIDAAYLQNVDWQPQQSSSSSSTSTLVPYAVCYSVDDGLTFHPAGIASEFVNT

DTSTAVPAPFTAVVVPPTVTSLSASRAVAPGVEATVDQLNWSPTYATATAVRVQLLSTSA

AADAGLPGALPRVTSYSRVDDVDGPAYATRPQSYEYAWTGAFVGNGMGTESGRVLPPSAA

STADVPTVAAAVALVRTSLHGARCDGVQAASADFTTFRGRRGNSNSHVITEDNWENASTA

SPLAAPIFVVNLQHGGVWSPADTAAVAAWRAAQTNHQRVTAFDKMNVVLAHETWLADTAA

MWAANDTAASTSGAAAPLNGLLLTGHPVSAALAAARSNGESVLVCLSTDGSHFYSADVAR

AAVAGVASAATMTTVSAAGLSSVAAAQLSPWPLYLRARAPATVESHSASAGATTATAITT

PALLWMREAAMVMELLVRPVTATSQATRPAPPSLAIDAAALTRFQAALAAQLGVEASVVV

VQLTHSGVVTLPQSDVPAASVTTPATTTATPTTTTSAAPVSRRTTGATSVLSTDDVVMLS

ATAAVQARHGVQATTTAAAMTATAAGENVQAAQLLTYALVVSIVADAVAYNATAATGSLT

ASPDLLYRTVAALRSNVTGPALLSTLVADRTTSSAAAALALHPLTVNFTMSNGMDLHTAA

ATMAEALPTSPPQEVAWRALFLSIPYATVVPAPVPDAEDNDTTSKVSWWVIPILLVLPAC

MGYGTYYVYKKYLKKAPEEATQVTVENAQ*

>Lp_000072600.1 serine/threonine protein kinase-like protein

MTTLDRLRSRRDGGGGGGTNNSNANKNDNNSLTRNVQASASHNGHRADHVDASAEPKTAI

ERWRAQEEQKRREEATRARQYRLSARQWREAFRRLEENRMKWTMRSFIGAGTSGKVYEGV

LDDAAHTPVAVKVLDVGVPMPVGPGTASGGGNNMSPAQQEALLVLLREVEMMEKLHQENI

VTCLRCQVTPVHDRFMELHNQRQQAQQQRQQRPSSQTQRGDDVNSDTVVNASGSPQCNST

NAIAAATLVPVQVEIIMEFCKRGTLASVVRRSPGGQLPVVVARRYLRDVLKGLAYLHRNN

FIHRDVKGENVLISADDVAKLADFGCSRRIVMTNSHAASECHDGTMSSLATTHATDSRYT

TMADYQWCDATAVAQTMVGTPMFMAPEIIQASGAPPVSPSSVDSTERGGFNNPNGSFAAA

ATPAGYTASADIWSFGCLVLEVFGRTPWPSAGNNVYLLMKQIEQSVDDLPPGVPSDTPAE

LLNVLRCCFHRSPHRRSTARMLLRSTWMTCKDEELEEMPPRRRR*

>Lp_000073400.1 hypothetical protein, conserved

MAALVNTAEELYEVVCGLQQEQQATQLELRAIQRRSESLKEKIKEHQDTIANERHKRQDV

SEKLKTLRFTARLKLAQKEGVALAAQDVMKTLHAEQLRLLHVCLDNAQRGSTSTSIAHGS

NETQLSSSSSDSFLHKDTLQMDNAASLANLFKEPENPPYTGVALHALLTILRKKLLKGVQ

AVDATLKVRQSPESTHNAASTCSGPDPQQQPPLFTATPSDQLFLPSPSPVPSVLLSRKRI

VFARASSFQRVTPVSASNVNRGVPQPSRRSVSCNGSQSEAGACGAPQGCITLAPTAGAPA

PRSVTKFTVRVASRSSTLIAEAAALGAKRQRATQLTRDDAQPLSLAPSAAHPCRDGSCGD

PPGALSGVSLSNERDTACPMEVHDGDVAWRCWTSGPLACGPRVSGSAGSGPRRTVWTWTR

DTTEEKL*

>Lp_000073500.1 hypothetical protein

MVRLQSTLLLREDGSEELSCASLDSSGTPLSGAADRFVMAAAVTASLWSWGTTDGGGRRR

GTSGLVLADDAALDSTFSGVFTSSLFARQEERWKPTPSSGEDEENNDEGGAALGRDHDGG

SRMGRGAHNFDAAVADADAVGIALVC*

>Lp_000073600.1 heat shock 70-related protein 1, mitochondrial precursor, putative

MSTNAIGIDLGTTYSCVGVFKNDQVEIIANDQGNRTTPSYVAFTETERLVGDAAKNQVAM

NPHNTIFDAKRMIGRKFDDPDLQADMKHWPFKVTVKDGKPVINVEYQGSLKTFFPEEISA

MVLQKMKETAEAYLGTTVKDAVVTVPAYFNDAQRHATKDAGTIAGLNVLRIINEPTAAAI

AYGMDRKNDKGERNVLIFDLGGGTFDVTLLTIEKGVFEVKATAGDTHLGGEDFDNRLVDY

FATEFKMRCGKDCRVNARATRRLRTACERVKRTLSSSTTANIEIDALYDGSDFFSKITRA

RFEEMCRDQFEKCLEPVKKVLADADMKPQDVQDVVLVGGSTRIPKIQQMVTQFFGGKEPN

RSINPDEAVAYGAAVQAHILAGGQSDKTDGLLLLDVTPLSLGVETAGGVMSVLIPRNTTM

PVQKSQTYSNNADNQRNVEIKIYEGERPLVSQCQCLGTFTLTDIPPMPRGKARINVTFDV

NTDGILVVSAVEESGGRKEAITIKNDTGRLSKDQIEKMVQEAEKFAEEDKANSERVEARN

TLENYTFSMRATLDDPDVQSGVSQEDRQKIQAAVSVASNWLETNQDASKEEYMEQTKAIE

NVAHPILSEFYKKRVMEAPPSAGAAPQQQQQEGAGGEPHDTAHGAQDVD*

>Lp_000073700.1 Peptidase family M20/M25/M40/Peptidase dimerisation domain containing protein, putative

MVKDVQPQVIEWRRHIHAHPCLGHQEGPTVAYVMAALQTMPAKLVITNPTKTSILADLKG

GAGEGPMIALRADMDALPLTELTDVPFKSQNPGVMHACGHDTHTACLLGAVKVLCEMQEK

IKGTVRFVFQHAEEVSPSGAKQMVEAGAMKDVDMIFGLHNRSSLEVGHTASCPGIASGAV

IDFDITVHGKGGHASAPQMCNDPIVIASDIVMNLQTIVSRRIAASKVPVISVTTLQSGTG

SFNVIPDTANIRGTIRALSDEGEKDAPKLVEQTANAIAALYGATCTFDWPEVVYSMRNDQ

KCFEIVKKVCTEKLPAGAAGFNVVTDPSFGAEDFSEYERVVPGCFAYFGVKNESIGACYS

GHSSMFKVDEAGFETAVRIHVGLIEELLMPG*

>Lp_000073800.1 2Fe-2S iron-sulfur cluster binding domain containing protein, putative

MLRKVGARPYKTMVRRLAATDAAAEAHGKKAVLQLVRFDPETNFSRVESYEYDKHHDYMV

LDLLIAVKAHQDPTLAFRSSCCEGVCGSCAMNINGINSLACITFAQHVTTVGPLPNFPVI

KDFVVDLRHFFQQYAYIRPFVRNANLHRNKVDGIVERYNNIARALSGVSPAEGRAMDALQ

EEIASIQRSETTIAALLRIADAAVDAGNATQVVSVLERVEKCGVTLDPVKVTELLERALK

NYAAKTN*

>Lp_000073900.1 palmitoyl acyltransferase 12, putative

MCEGSVHTFRPRSFCGWCALFASYIPCLFAVVLIALNVLPYHLSFLPLLRASCEAGRVSY

AYYYYCTAMMVFAEAMVYGNFFWAISTAPGFVPHEPWAEAPVYQGRAVSENPYEVFELDR

AGKLRYCAPCRQFKPDQAHHCHMCHRCVYRMDHHCPWINNCVGRSNSKYFLLFVGYIPVG

AFHIVFTTLYSCSFQFSNFFSQAMTDENVLTSLILILSMVFSAAMGVCFFAFAMHFVCMA

YQGQTSVSRMIASKKQADELEQLRKRAAVDRLFYMFDLFGADQRWYRMILPFKPDHDIRR

APPGYAQRFGGSMDEYMTSLV*

>Lp_000074000.1 DHHC zinc finger domain-like protein

MTRRSPNQSSDVTHPLIYPASSPVPLQKNQLHPLQHSQQLELPVAVSANLPRDSADAEPR

KPVSDVCPAAPVTAVVYGDDRESGTVSPPQPQPQLNSTSSTANNNHINNNVVENGDGSEV

AKRTEEMSNSAATPVGVPTDNHCPYATPRQLVSLSPANNTITIPAAVAAGAPSPAPSLNP

YHVRQIEPNGSLRFCCICQQYKPDDAHHCRVCERCVFNFDHHCPFVNNCIGRNNYKLFVV

FLLYSGVGATVCGGLAAVTLFAVDRDEIMTKIGWIAVPGVDVILGISLLLFYIQHRVLLC

RGESTLESIIRGDETFQRWRESLSRPRRTAAEKAEANRRKREKVERHNRTLLGNESPWWR

RYLPLPVRTDEMASDTVPGNV*

>Lp_000074100.1 DHHC palmitoyltransferase, putative

MAILNAHTQFHVRSWRELLRMIVAYRLPLCIFIAASVLTIGYNVFFCRLLCTVSHVSASL

ESLYHTLNAVHTDLFWLTFPATRRFSIFSLCLGDVFFALMFTSFLRAIFTCPGYVPREPW

RWPPKADAERQRSLREAWARQQCWMRDQEARAQLQAAHAQQLLQQQYQLWWSMQLAYLQQ

QQQQLQTMIGVSGSAPSTISFPQQNGISFGNSVARTPHAPPPSLSPQEDNVCGSHEKTPH

FMTTLHAEDTVTVPMNSLPRIDPPAASMEASHLPKSSGDNDKSARPLLATSQTHAALSSS

SASCRDLSPTSSVTDASLKDASSLAVAASAASSACGSTAYTRNTTNTAAALISAVTGCSP

HLRPLAHTPQGPSLNPFTVHEYEADGSLRFCEGCHQYKPDSSHHCRACQRCVFDMDHHCY

FLNNCVGRYNYKLFFLCVFYATLCGTVNSALFVFAYAGSAVCAEWGHGWWWVPAGMSSIG

VCVSYLWVQHVFLLVRGVSTLDRMQQLASERFLAKVSGEHRLPGLARSGCHTDCIFSIRV

CSCAVVQAVQHVFSVVTGAGGRAFISNKARLQPCPSSSAKSATDAMLTESKRRARRIALL

FGQPQHWWEFLLPVAPHDDTGLLEEKAIVCVHVEAV*

>Lp_000074200.1 Inositol hexakisphosphate, putative

MMGAEMDEEEEAELQDLKLLLTAQSSRLQRRGSPDLFARGRDEKRSTLRPEEVTALLISQ

ANTKAMQAAQPSKPLLPLHQEMRDDGSPLLSAQRLPEPTRTCNVSSAQVPDGWGSAIRKG

MRYMRPDDFDMDAPFTGYGNSGAGKRTCAANADGDDGDNSADAAMLAALEKESALREARL

ERRGNDLRRAWAASPPSLHGVIYPAPPPVTLAASDAEVQERALAAEDFARNQARNRKRSQ

HMASATMTGADEASRRCHSSNNDCSDHFKSNNVSYRKSNGDSGNMSATTADVAGEDNGSE

GNLFDLVPLPHNQRRYNTGYGSPEPRELGDMSMASLALGRMSMASCSVARPTDGTLAVME

MLSNRPDVLLPSPAETSVVRKGDVLSAKHILARDLEEALHEREGATGMVAGAPYFRIVPK

LNIAGVAQANASAVRTIVNELRRAHVEGSIIWVNLREEPIIYINDTSYIVRERANPFKPI

IIPNVTGLGIEAIERKLKLEVLQEAYENGGNISVYLENKGGNMEDQWTCADREEVLTIAE

VFHRLDKETDHQVMYFRRPITQNIGPQPEDFDFVLDACLEEPKAVFIFSCQTGRGRTSSM

MQIANIVRFYQLCVKDVTADVRVLRGKVNAPSYRTIQKLVSLFPDGKLHERRLVILMEMA

DKGYSMAEHINEAFSDTETTSEVAKMRLQIYAYFLVFSYYCEQRLWNYATRLSFVDWLDE

NPEMKLLIASVREKLDDQLKSERVVAPVASGPEADAIRMIRQRHGNVLSSGRILCSLPMS

SNNKTNADIVALRQLAPGVPIFTCGRLSGAARDELVHDIRSTFPRAQSIHWVSLRAEPMV

MINDVGYTLTDYDSTPDAAAHGMTMHASVQAMEQMEDRLRRDVLLEAQDNGGFIVLHRIN

PAGERETLRVKVVSVRTPGSMMEEFVEQSGVRYSRIPMPFSGQLLASDIDPLFRYLTQAG

LTVDDAIVINDSAGTTRTTVALNILTLYAASRLGDLRSTQTVENLSVLLSDGSSDMLVPH

AQVAAKADVPSEDVPEHHVELLIASTICQMLTAGSLLRTVDAAIALGGRGRHWNILHELD

YLKRRISGLGNKKPQCIVDALHALRCYLLVLLACLYLDAQNEEGFTLQGKRFSDWVEEHK

EVSNIVEHLEQRGEAALNYVAAGNLMKADLSRRSGDVLTANFGLKADHFPGCQKKGLRPA

VCGAPNFRKVDFVNVYGVAIPTIIGIHNVLSLLGASNEPLQTYPGQSNDSELCLGFAAPR

LFDPAFKPEELQHPLRGSVVWVNLREEPILYVGDRPFVFRDLAAPYVNVELTGIQTEKIE

IVEYELKRDVLREASEYDGKFLVHDEGNPGELVGVWEPATEETVKTLREVYDELMVKAFR

CQMLRLPVTDEQSPDIHDFDLLVDALLPRIAKHLDRRETLSFVFNCQMGRGRTTTGMVIC

CLLIGLVIPEYYDELHSAYHDTLEAPGLSDFSRGEYAAIVQLKRILAEGRTAKYQVDLVL

EACSKMQNLRTAIEGFAISVSSPDITESGRARAHHAGVHYLKRYFNLIVFAVYLQEEYDR

MSKGMRRTFVDWVALHPEVTALLNTCALK*

>Lp_000074400.1 Meckel syndrome type 1 protein, putative

MDFVHFLKSQRYRSRVPLRQLQFTVAVYRAIVVEETNVELLAEVTVPWDGKVFSPLETLT

LYKDLAQLSQGPTTAAGEVATMTTTVTGTPAESVTPAAAAAALGINTPSAPPLTTTEAAR

SGAAHNPTAGAAGPLSPDALALPSPRELVAELRNPSSFFFTRPSLEDFIDRAEEDCPVDP

PQGPSLLAPVVLRQHRRDHQPNRKMFLMWASGDVVLPEGATAPPPTVPLSAADTATTATG

QEGGIAVVPRDAFASTAPLPALEVHALAWKGVERVLCTLTAEPDEYVFTARPSLNDVHTL

FVDAAHIYTFRVTVSKAEAGGGGRAGLLLSDTAAGAALDATAAPLDAVLANVRELAHYAE

EQYEQLEIGKDAVARRLLRQAALLAPADKSGGERAAVAAPAAAAAAAEDVGGRSRRVSLS

FGISTGAGAERLGASMSAPALPRAAGVGGTVLSRSTVMRSGHALPRGMCQYYLFGTVDRC

VGIAESTLFLRCQLVEDGAASTGLYDPLATSSPSAVCEFSSQLAHISTFVEHECVMDIDH

VFNLPFDYSFVGAALPSGPLRLVVSALTEGAAAEGIQSAVAYACVSLPIASPGRHTLKAA

MWAPHKTGLEFLRSTLLGGAPSLVDARQAGPPPSHRTGISVKEGLYADSVGSVYVTVNIL

HHKNHEAL*

>Lp_000074500.1 hypothetical protein, conserved

MQPVTSSIGVDSYNSPSVNVTYKRANGEVPLSVNTPQPVLPALLRPQFVVLSNDDLRGLE

RRIYYQDRAAQRDNRIEERAARVAAQSRLTAEQREAKLRQHAARHAKVDGEVRVKQIQEE

YRTIGMR*

>Lp_000074600.1 hypothetical protein, conserved

MSDPAKINHYDSRVYTRKGATQRVVVREKPLIKSRLQRLADYLRPTTRHGFNVVYTFELA

KVMACIIFPVFMLLYWKRVQKNLPDSWEQQFGGLQHRQFREDQLPEQDTDYFSIIENFQE

RRDTALKKKQKEVTENKIA*

>Lp_000074700.1 RING-H2 zinc finger/Ring finger domain/Zinc finger, C3HC4 type (RING finger) containing protein, putative

MSGEEMPKRGRADSPSDAPARTTEVNEVTDSPPSTPPVEVAEVAEVIDVTPPNAERDPVL

ERQLDALDVDEDFRESIRAMTPNTRRDVLNDIIRHQQHTGIEENLHTLGVSRALGGPIFT

FSSDAGIYDDDDGDHSEDRDWADEEEEEREDDREAQHSDDDASYNYARSPFMFGQRAMGP

HDDDDDHSPARSNVRIFSEQEGGSMNVVDFLQFVSQHIQRQPSGPPQRSTRGRAAQPPQQ

RSILEERMLSLQNLIGMLQQANAMQSMGLHRDVDDMSYEELLELEERIGNVSKGVPPALL

ESCMTRVDPAPTDGTCPVCQEELVMTAAVAPPPPSSTSRRNSSANASKVCVKLLNCPHVF

HKSCIAQWLAGNKTCPVCKQEVLPQADGTSL*

>Lp_000074800.1 Qb-SNARE protein, putative

MYGLFNSYEEDFNDTVRGLHESCGKLQGTIDAQQIHDRDPSHVYHPPPATGPLSRAQQLQ

LIQQSLSHAKDLLTSMTYEMTDVAAEEKAATRAKVETFRKTCNTLDSDVARLRQSSNAAD

RADLLRFGVNASSTTNASGRDAFMSEADAETQAHRLLALQTTERLQDGTGTLHKAEAYLA

QTNDLGRENLNILRSQTEQIAHIHETTHDVDAEISRSRQILNQMHRTAIKHKLWLIGIIV

LLVGFILLLFYLH*

>Lp_000075100.1 Tetratricopeptide repeat, putative

MFTTRAIASSLYRPAAVVLGLTALSTQLLPVASSRTKGSPSSLYFSRRACSTQADHTSSG

SSSAAKPVSAVNVEEFTLNNEIVKRDLENMAKWNALSEEMDAARAAADYAALLQHVAKGL

RMLDEMGAANAPIQCECLLCMEASQAHYNLKQYDDALRCAERARNSLVADGKPELQDKAQ

IAEIEVFMGFTLCKQGEGAEAQELLQKVLNWIDVDAKSSMPMQAVAAVNLRRSVLTGIGE

SITLQASALAKKGETAEARELFAKALDILIEGLNQHIDENDVNLVKSTLVSILTCFEGLG

DVAQAVTTCRKYVSWCRRHDDAAGVAEGEEMMADLCGRHNVENPLTAEKAVK*

>Lp_000075200.1 hypothetical protein, conserved

MTSAIRFSRSQSQNSQATGSSNSDNNDVPTSGRASQASQHRRTHVTAAAASPEVSEAFRE

LPSSIPSASSQPASQRTKTTTSSSSRQNRSEGSCQSRRTVSQPAAAEAADPFRYPWGVYC

SSPFLPTSSPAVAAVNAHMPVDDEGQRRPHAHDGHRRVCHHHRHHRHHEVFPFAVPNLPW

IPPPPLGGVPLTAMSDVNAHLAQYYAGIEAAAAAAPQQQQQQQSPQLQPGNVRSTRRSAA

AACSSSSISRELDWPTPDQLPLPSVFPMPPRMLRQQSSRDWALFPPPPLPPWWFLQAVGI

PARDGFSDSRSSSSNSDSDSDGDSYSDESSTSSSRASSPSPHDRHDRASCIPPPPPTSSP

PPRHQRPPWQQRQARRYTPPAGSVETPTPAPRGSAAHTTITTAASSRSLHSTEKRLKAGA

KAAVPSVSRRARPSGDGTDDAAHGAHRASREGASRDTTNLARPQPARQAFPKAEALDERS

GVRGADTGTREELQDGEHRMQHALSTAESYLARIEDLYHRLRHRYDGMGPSPVKAVEQVA

DQAAAPCYRERADDRRAETEDDDRSSSPQRQQEQRFHYTAVPTREALPSPLPTSASATYA

VPLKSAASERASTSASQSLLQELAVLESQWQRLEGLKQHGVGASAAHTPSSHAPQSFSSV

SLPTAAGTAAAPSLARGSDVFSNQSVMELINDRKHLLALAA*

>Lp_000075300.1 Triple RNA binding domain protein 3

MRARGGRGDRGGRGGDHGGFQRGGRGGFNRGGNRGSFQRGGGRGGVFGRGGASAHDGEHA

AETGEAAVGKSRNPTQVITTVTDSGTELPRKLNTKVFIDGLPYTYAPEPGKPTLEEEVLQ

FATAWKVGKPLRLIKKPGQGFGFLVLQSPNSVATAVRVLNGRKFLGRALRVEEPKPKDLE

KMKDVGGMKDMGKDSFTRQVLLTDLAKVAQPEIIREVLRDVAPQLEKKLEAIKMTSQNRK

AFLTFESEADVGPAINFLDGFHLLGRRIGAAQAAAPGSLPFSHGGTGPSRGGLAAAAAAA

NGGKGSAATSRDAEDDEEVAVVPLGMEPAKPSAAVPTTAAAGSKSLSARRREEAACGTGS

NVTGRTEKYNLLDDGPRDVYVGNLADDVTEAQLRQHFAPCGAIRKCEIIVHPETHLSTGI

AHVEFALPAYAAYAQERYHGSRLRGCVLRVDRGETASVPLAAELPPAEVDDDYDEDAYME

RYGVKDKKAFFKGTSLAAEMGADPHVDDDDEEEEQIVATKKGSKKDKRQRAEEKKGKKTS

VSSPAEKRARAEAPTEKKGKRKAVQPAKIEDDEDDDDINNLTSYTMVEDDDEEERFYDAD

NVHVAAAAATGGKGASFGKKSKKNFKSKAAKKKQ*

>Lp_000075500.1 flagellar radial spoke protein-like, putative

MAGVDENEVRHSPLWGHLTKVLSQVIRERPANALEAMGAASNQILTGSAVPPMKGTMYAD

PRPSARTAAPADSFTNTRWAANTNAALAPPKPPRRPRRADGEADEEEGDGEEAEEGQPGV

DLAEHPGTLSDVITEQRYFNQVGLGLPPSDAYRLLVGLRQLIRAEPLATVRFWGVVTGSE

ADYLVAECKLDQERVQESVALNGDEEEEEEEGGDGEEHAPISQVADVLSTSAAHRRPRRG

PRPSTAAEEAGAGLNSLCYYVSTMADPITWTRLPDVAPHHITVARQLRCRFTGNLDAPVH

GHPRLLGCERHYLRAQIARITSACRIAPAQMFTTEGAIPESEEEEEGKSRPVPTSVPAYA

AVPPLLPQEQPDEEDAEAVAAVQAWLSGYAKEELMQAKGWAHIAPTVLRCGRVTAPPPPE

DDQGQSEEEEGEEGGEAAAPPPPPPAPEMIAPFLSPVSLDVPLSYLGHSRAQLPAWTFRK

AYHGEGSTTAVYVAKSLVWPGAATYAVTTAGRPGASYQMLYYGTGLKDLQGAYYAPPLPP

PCCDEYAEKPAEFEGERDCTVDEELHYAPLPPRPAKEVEEGDEEGEEEA*

>Lp_000075600.1 phosphate transporter, putative

MAISTCQLDVNTTLPLVCICTLLSFLCGVGIGANDLSANFAMVVGSGSLNMRQAIIYCTI

FELLGAAFMGGHVSSTIRNGIVNAALFARNKDAVVVGMTCATFAAAMWLYLSTVFGLPVS

ITHTVVGSILGFAVFASGGFTYVKPNGLVVIVVSWVAAPLAACAVTALLFYVMRRDVFKV

KGHSFEQALKVLPYCLFASLFVDFCFILIEKPPIMSQTFAAYMPLAAQYLLLLAFIFLFC

WLVTFFVFPQVAEAAMQASSFVWESEALRTEPADSPIRVNQTEEMKNLSFFKPKRDGAGP

GASSRQQQQQLHNAGSAATFANFSNTTTPNMTAAPPPAPPPPSSRLSEAHDPLATSSVSL

ETAPTLEATAHSSSLDPHANANINSVSSANFTSASFVLSSIVASDNPIVHKGSGVRLAPV

PTSRTTSPMPRPQASGANFARVGSSTVNETSYLPGHNVAASNQEERESRLRHAATTGNYG

STATTTGAGAATILRSGSYVEPEMTDPRTPASVHSEDEFGEEDWEMDHPMQPIRFGGILI

KPFNPRAEYLFTGLQVVAGSMSSFVHGAVAGANATATFVILYDAFTNHELETPGLSSQWS

VLPAMLGIAIGMFGLGASLMKTVGMELVTVTPARGWCIQTGGTLVTMILTGIGIPVSLSQ

SQIGAAIGCGVLDAKLGGVSWTIVVKVVTGWVITLIISALTTGISMWLVSAMLCS*

>Lp_000075700.1 Scavenger mRNA decapping enzyme C-term binding/HIT domain containing protein, putative

MQVSQLCSGAAAAARRKGHAAADNGPRTKLPFLINVVKKAQKSANTATYKSSPPSTSSSS

PTDVALAEQSFKPYPLLRSGEAKRTAESSLLYKDDTCILVNDAFPKSTVHCLVMPLELRL

DSLNALTKRDVPLLRHMIHVGNEYVHFLKSTSPKLYGKRRFITGFHALPSLPMLHLHVLS

MDLDSVYLKTKKHYNSFATFFFLTSERIVDDLERNSRVTINKDVTRLQQMENQDMKCLWC

GAPLANIPAMKAHVRTCTENKSVEN*

>Lp_000075800.1 Qc-SNARE protein, putative

MNSIQPQSTLFRRRDPPPAGESVSPYGSPSSSVPRQPRYQAANSSASVDTNAAQEQENDA

LMQALLADMRRTKKGFTKLGDEVREQNALLDRLKATFRDAQGSLRKTMHNLDKIGWGSYK

HMWILALFVIVFFMLLYLVLRFR*

>Lp_000075900.1 ATP-binding cassette subfamily A, member 1, putative

MSFFRQYFIQLYGFLVKTFLQRWRMPISTVVEILLPCLFTVLLSISYWRCKSTTEPAQMY

DGGSSAVAMNLTEFTTYFMCKKFESTLKVPWRPCSPTMNSTNSVCLSQIGNGQEICLPTA

SYAALSGILYTMYYGSGPLALNSMDGHLALSAMGAQIARDDNPTYFGRSGRASLSHYGKL

LVSSDSEAVAQKFMAFCRAKSGMCAEVLYGTPFASLESAKDYAAKNENTVWGIVDIPSAS

MSGTGETEFTISMNFTATANTAKGEVKSLFSRGLKTDGSAGYVLYWTSGFMTLQTFVQEF

YMQEALSSYTVVGPAAYETNTTTGVEGVSKYLTPYGSEVIPMPTAAHFDNEFLTEWAYYM

PLLAVMAALYPTSRLVMLIVSEKHNGIREAMLIMGLHPSCLFFGWYASTLIIDIVASLLA

AMFLKVGFFSRVDYGLLVLLYFSFMQQNTALCFFVSSVFKNPRVASWCIAFVLFVCAIPS

YSFPVGMTDTQKIWCCLIPCVGYAEVFNMMLDFVSAGRHYGWPQARVGYFDYPMAIGMMW

ASFGALMLLGFYLDRVSFGAVGRRAHPLFFLMPLWNLFHKHKAPVIKMSDMGIPLRRGSL

VEPETPTGTSEPFGESSMSSPLKKNTKAAEDAHRLIEHYNDVVDPLDTSVAAVFHRLRKI

YIGGGILGFFYTYFTGLFRKGDRVVALDGVTFAMRTGEVSVLLGPNGAGKTTIMGMATGM

VSPTNGDVYVRGYDARRHLDQCRQNIGYCPQRDIVWSHLTVEEHITFYARMKGSESIHVR

EKVEYVMDLVDLTEKRNCKASNLSGGQRRRLCVAIAMVGDSSVLFLDEPTAGMDIKGRKT

VYDALNRSREKRSVLISTHLLDEADRIADRVLIVNNGLLCAEGSTMYLKSQMEVGYVVTC

LLDGGMTATDENRAAGALMDFVRTKSCSAQRGEVDAEPGCVVLGVERRGREVSFRFPMTL

LGSAGVDLLKQIQSNSRRLHLRNVALNLTTLEDVFFTVTRTRPLMTAVAEGELVTDRDAL

NEVSSPLTMASTEDLYESRASAASVFGRHFRALFLKRCHYAQRDIKLLVYQVLLPVIFLL

LSLFVNLVRDPNQPTLRLDMTMYPDYATNPSQVMTAYSTFSGFVENMVPYPMKVTNAFQL

SASVPDGAWGSYYLTDFRNIASGQANASYDMSKYLLDEITTHTSPRYIAIAPVGAVYQQG

KPKMVPTVMHNSTASHAAPQAVDALYHLARTQLFGSDVSLPTVVNSPMKLGEFEKNMVTI

NKQVMVGIFIILPFIFIPSNTIGYIVEEKETGARHMQWLSGASVAAYWLSSFVFDMACYI

FTQILAFVIFVIFHRTEYVGKHTVGPAIVLFLFFGVTSIPVSYFMSFFFKSSFSAQSIVF

CINFTFGFLWVTVESMISEQALRFAEVVTYILRVFPAVCFGESMYVLAGTEMANLMFPKR

KKKSLFALLHFDSSGKPTGGIGTGLIYMSCVGVGCTVALIILEYLRLQRLNAVFTRCCTR

ENAADVEEHEMLECADPTVYAEEKRVCAVETGPETDKIAVQHLHKRYMGARHAAVEDISF

GVHEGEVMGLLGLNGAGKTTTVSILAGEVVATGGKAFVNHYAVQSIVSRSYVGYCPQYDA

LLCNLSAEEHLWLYARLRGIREKYIKEEVPELLRELGLYPFRTQAAGSLSGGNKRRLSLA

IALVGHTASVLLDEPTSGMDAVARAQTCEMVRRLTADKSVVLTTHLLDEVEALADRVAFV

VRGNLRCVGTPQELKAAYNKEAAYTLNVLFPNTVRLQAEEQDVVERVRDCVLQMVTAAEA

EESAAKRSITCEVSEVHPCSMQLLVNGDLSAICAVMSQLQAGRMEGIPATTYVSVSQPTL

EDILLLQ*

>Lp_000076000.1 Zinc-finger double-stranded RNA-binding/Zinc-finger of C2H2 type, putative

MPSRRHSRQSFRNVSPEVLAKAYHPFDKLIIGLPPAMPSWVRAYGPATEWLHLPPNPLIP

SVRRQLEAGDANTAAVLPTPSSADAGVRDHDRSPPPSASRHSPKSYRCDTCDRDFASEEM

YEAHMKSHIYCTVPGCHFTCRDTRAHLMQEHMDALHNRPDAPNLVDTGAYLEQRKRRFPT

QEVIKDKVEELYYKAARGVVLPEERRRWLRQHGVDVGKRARTEASFIARGSVPEEGARSP

SAQSNAGSGRSSELSESRQHRRSPLRSPAEEHREEPPQPRFIQPLAALLPKDPTAAPPSV

PAASEQPPMALPQQSSPTPTAATSSSPLPSRQPPTAASHQVKMIPLGPNGTLTPRQRVQL

VRERYAAAKEVPQFYVCHRCGRKGEHWVDDCPTKDDATYNRHVVWGEAKMEVGEGGKARS

RAAQKGRAEPNSRDVPPEMVAKEEVKASPPPQSVQQEASTDPQESPQEVPHAEEKPEEKP

DATSSCADHDISASTAPAHSSTSGAEDNDKNNDDDAPPVPQSAAPPADAPVEVTRVMAPV

AAARYAQQRRDPPRRPPPPPTLYERLTEEERMNEQGLLLQAMRFFTARDFFQEKKQ*

>Lp_000076100.1 NF-X1 type zinc finger containing protein, putative

MSASPQLTQEISDDLVSGRYECAVCSEPIGRQHELWSCRCCYGVFHLPCIRFWADSLTKE

REKQLQSTSGVATQDELDRFRCPLCQSFNPKTSLAVYQCYCGKVTKPAVDPMLVPGSCGQ

PCERRQADPCCPHRCTLLCHPGPCPPCTRAREQACWCGKHTKTVGCSSGVHGYECGETCG

KLLDCGQHRCTALCHEGPCPVCTVMVTETCYCGATQRTRRCGAVPPGDSPAGPAAASGFR

CTHRCEKLRDCGKHVCGLMCHPGECEKCFRTPERQKFCPCGKTRVTVQRTSCLDPVPSCG

LPCELPLPCGHLCWLTCHDATPCAPCREMISLPCVCGARTMTIPCFCQYLPQSEWEAARQ

QCELPASALPSCFPPKCTQVCKKWLSCHKHRCTNTCCVDQEHTCMQICTKKLSCGVHQCG

QLCHAGPCPPCSYVSYEPLYCRCKRTWVDPPVPCGTKPPQCHYPCSIPRSCGHPANHECH

MDPPCPQCVVLVEKLCASHQKPMPYHIPCYKTEISCGRKCGKNLPCCGKFCELVCHSGPC

VHKCSQNFPTLADVLRKGQRGNAS*

>Lp_000076200.1 DNA repair helicase and transcription factor protein, putative

MNIGETGSIFIERAHPAYAHVIDFLVSCCEPVSRTHHMEQYRLDSSSLSAATAEGTYTLA

MIEVILRHFRLNDAQELPVALERCAALQQLATALDESTSASLEEIEAAAVSLRSSASLGG

ASAGKDAEFPRVRAHAPPLPPCLREADFTETVLAPFYPSKANMCHLQVQLPSAVAPSSLS

ASSSGLSAAPTVKLEKTEADVTTSAASPASSAPRGRVLQLFRPASPAPATPGATAASVAS

TASPALATVSAGKALIALAVVRPDDVQKPLPPAIAAMLAEEASASRVQIVLQPRLRRFHS

FDTAPAEAKASKRKDETLFYFIESRQRAHLELVLDDLKEFLEPVLLYGKERWILSDVDRN

PVSAAAAGGGGAGCSSAERIVRDAGRPAVLRMLYQQPNADRSVKASVGGSTSSTSATTSR

FVYKCQIKSGKLKEVKERLFSRFHIRADCYYDYVQDRTLHVANLNLASHVRLRPYQVASL

ERFHRGSKAHQGVVVLPCGAGKTLTGIGAAASMQKTTIVMCVNNMSVFQWQREFLRWTDL

TEEEVTVCTAKVKQMPGRVFITTYNMIIAKRGSSDGVAAEESRAILNAVTAQPWGLLLLD

EVHTALAHHFQDVLNTIQYKCVLGLSATLLREDDKIGDLRHLVGPKLYEANWLDLTRAGF

LANVECAEVQCPMPLEFLKEYRDVQRARLVLGAGKAVAVRRGRRRRRSEDDEYGDDDDEA

AKTRGRCGRGRGAHATTMGLNSRALHLASCNPYKLWCTQALLAFHQQRSPPDKVIIFCDY

LADVRFFAHHLHLPFMDQRTSEAERANLLQYFQHSADVNAMILTRVGDVALDLPCASVII

QVSGLGASRRQEAQRLGRILRPKPPSLDNTCAYFYTLVSQDTGDIRTSYKRQSWLRDQGF

AYRILHSDSVLNEFMRVGGQPCCIGAPRWWYQTTNTRDDAIVKKRDDASSNANSSSSNSV

LFDGIYWAPFSCEAALKIERAFLQGRDGCVLRGGELGPDTPRPTAAALGGYALRSTESWE

VTFSSADAPQTFGTVRIGEGGGDAALRVRRVRRGRLDQSHRCTAPDSSNNCMQYALNAVL

GSHGAVSALMNHANESLSSDDASDYDDDDLPVEDV*

>Lp_000076300.1 hypothetical protein, conserved

MPGSLIKESSHLPALRPASGISVTHATAAVGGPTCAAATASFTSVTNSTMDGVAAAQPDS

SGYMMGEVFEEDWQRPPGDLAYLQDLFASLDGQRHASPPQTQTLATASTTTTTTATPVST

TAGKSVPAGRRGGDGDGAEEEDVAGLWRREQQLRVIERRRRRAQCRCDAVEAAFARSVER

RMRAMQHLAIDPPAERLVGQGTAQYVPAALLPDDLLDAEARLRPEPLPLLPHSVVETHML

QRLTSGADVPHSVHTATSQPVAAITDGAAHRPHEADALVFLTQLDAGKAPEKGGQARSTG

SDSHHPQQQTSSKNRSETPAASLTPPVLTENTGAEGPLLQPEECVDDDEGDDEDWMRPAN

VTAANKDRWRELGYRVVVVGGGEAESHSCGGAPAASSTSSASKRAAASSGGAQSQGTATA

TTARASLWRRTPTSRLPPVCLVQRRLPEEDMTAAELRARQPTTKRAAFRRGDSGSAEGRH

WKMSEK*

>Lp_000076400.1 Ankyrin repeats (3 copies)/Ankyrin repeat/Ankyrin repeats (many copies), putative

MVKQQRSAIPPELRAIARAVADADVHEFPELVPAPGHTRPSLDALRGVTTEVNNGVPLTA

QEFKLFRAAYAECQQHLHSVTNGVITAGTPVADVLLLGARMDTLCTDLGLSLLALFPSLR

EIHSHLLELSEKLAVGRWEGQHHSDVLLDRLVRAVQQTLERAEQALRWYESWEEQLERSL

AEQSIAELASTTTTALGSPAHSRTSFPTREVAAPASGQRVSVTATAGDVAPLHAVKRWSV

VEADEPASSANPAAAAPVAGQAPFPLAESRRPSAVADGALPAPLSGKRRLPANVADALAS

VNADLDVHFAHLRWYVTVTDRGSVYFDAHDFLEEQVLRVTWERYVGRNRAACAAEDFFPF

LKLFPPWTHFAVMAVVDFKDCGVVSVYSLRRLLQVWGPLQLLGVNLRHDMGHGAVDLSQP

FAYLAASLASRPDAQAGDYVIGLSDKLGELRVAVLRHARGYRRHLWATASEHTRTYSSAN

FAGTVDGESGSTYASLKGSRKHFATISYTLSHDTGAWMVQGLTREEFDSAAEACNSFPEI

FQRPRGQLLSNPHSPHSPHSPHPHLALPTVATGLFPTPTTSTQKPATTPTGETADGNVSA

LHRACYRNNTHYVRTLLDRGGATVVNSALVDPLVGGSFCWTPLLCAVNNPHSDPVDVVRL

LLQAGADVHYRDDADCTALYYAIANRYAETTQALLAHCPTLQTSPYTVPLLVAIGAHDCH

LRECDVRRLVDVVPSAAVLRVVAAYELDMSLVALASSIVEEKMNGRNYAVTSAERRLVAE

RCAAGATSADRFTPAEELQRQQLIAHHTHCCLQARLEMEEAVRVLYGREYVLSWRVWLAY

LDYSAEERQRRGRAMVPIYPLGMLRATPVGHARNAVAAARRFTEIRPRHLGALQLAMQSS

GDRCVFGATLFRTATAAEPTDYTI*

>Lp_000076500.1 Exonuclease, putative

MSPHNKRGKAKAAASKQSRSERYQHTSTRHDYRSKKGSNSSYLSTTTDARQNRVQHTFGE

TDPLDQQIFDYIIVVDVEATCEENNRNYPHEVIEIPGVLIDVRTGQVDRARSFHTYVKPW

RNPTLTPFCTQLTGITQETVDAAPTITEAIKLFEQWYKETIPRGAKTIFATDGPWDFKNF

IHEHHVLRDHVAFPSLFYEYLDVRTTFAHRLNHGVPIKLDAMLRRMNLRFDGRPHNGFDD

AYNIARLVVAMMRVGCVFDFVLAIPLDDPYHYHLEGYPLYRREEGSGHVDRDVVEDIAKQ

CFGGDYFKFGQWHRESVRTYRLEHPKEFSNANVMALKRRNDRALARRRQRTWRLVTLTLI

ALLLVGIAYVLNAHHAVAASLRVKVNPTTTAASSGDSAKLNLSNATAATYTQREGE*

>Lp_000076600.1 Exonuclease, putative

MPNATGKSGDRPAPTLVPLSHRHSGGVGGSAKRSSSSSSVAVHPEHVGAAESFGSTCAAA

EKVNTPPPQQSMPPRATSSPFRSVTRTLDEEEDEAAVADVPSLPSAAATASPTAGVMVAE

TTDGGALNRNGSSNSSAVGANSGAPTATGAGGIKTPKRAQKKTTSGVHLHRRTNNSSNNN

SGSSGGSGSGGVGGAASRSASELCRAARTLAAELMSALMEHPPQAAQIAQYRQSLQSSSA

LTPHPAPGGVAASADTAASHDPLAQEGATQKSSAAHTTEFSGAAAAASSSHSFLEAVRNG

RAKLSHAASPSTSAVAPTSPPVLNKAGSGTPVLAPAATGNAGSRRRSASPTVALSPPTSS

GGGGSAAGSSGRRQPLELMPEDAAAIPQLLSLLREIALSCASLGTLNGRVPPSTANASTA

PLAASASSNNSSNKGIDSGGSGGGAAAAAVTATTDEAATHPSCSPLHEVEIDYYEEQWQR

VRMHLQLVRGLLLNGHEFADALDAALGADWRTAATAATATPPAELAETKRDDGLSPQLAP

LPAPSTEDVVRAAVEAAMNDSVVPVMPTPVGQDGPLQSTAKAAAVTGSGRAGTVTVSSTN

TTTVTTTAASKSTNTGTDSASATTTATSTTTFTTTTTTFTTTTTTLNHNARPFHSRTPPS

FPPPTVTQADPSSTPATGASPPANAAAAGRGTSGGTTNATNTTPRASNNSSSNAASSMKQ

LSENSSTYSPSPHVTTFLERSTPPASGRLVDDAEGGGLMNFPSTPLSQAAAPYQPTMSLV

GGTSPRGGNASTSSSVPFHLESREEMLRRRSAAPNAFYASMTHGGATHYLGGPPSAATTP

GTFVPSSVPPGSPPFALPSSGARSMPVSPLLTYGSPVAASVCPYDYILVLDFEATCEEHP

PPNYLYEIIEFPVVVVDVRLQRVVAEFHRFVRPHHKVELSHFCRELTGMRQADVDAAAPL

EEVILQFERWFAHTVPLHARCVFATDGPMDMREFMYHHSVSRQGIRFPALFYQYIDVKQT

FACFFQCSQGKIKAMLEVLHVPFEGRLHSGLDDARNIAAIVVGLLQYGCTFCEVPLNRLP

LNGLLLSSGAAAATGSGSTPLSLPPSTAAEGSGRHRSTAASPTYKTAPYSVDEDESSY*

>Lp_000076800.1 hypothetical protein, conserved

MSSSRGAAKDCALPRVSCKAEAALVAAARPLPLSQHRSTADQRQAFLEHLYSDYSELHAQ

QDEIHDRKNQMDFDMFESNQQEIHDVLFANVEATDGALGDWDTWEKVEATMKRYAYGRPQ

HTKQEKEEDAAMRSRIEALDEVACECNATIRALAQACTAAQQEVGGLRKLLHAKDNEVKT

LSNQNHIAALQLHYVQRECAALRGMRNENETTLLKQDLSYLIEASKRLEAENQRLRGRQQ

VVEAALREPKAGVAATASVAVAAAAKAAPPALSPATPQPSEEQSSAFTPMELSMQPIAAK

DEDLDRCLKDDNFRRQFLMKEVPLSGAVVNAKRMTASWKRRLEAEVHSALEILDRDAAAP

LEVADVVSSAAWPWRGTQSDGTSQMQNGVFISHASYEGTPRKCSDEQTVPVLMAPTISPL

PTATATAAATLPATTSSPTPPSSPLTGSSTAEMARRSAEDEAAALSVPTVGALSAASDIE

SAAASSDPRKGVHKSVASVANDSARASHANAPAPQKTPHTAAHVPKRRSVVVAAAKPVFA

PLQSAVLRCAELSRANAMRRELLHVAHLRQMLADEMARASADRASQLASQSNMPQSARDV

SLGTVADIVATASPPMEASLTQSPPSAEVAATSVPERLRQQVEKSRTSSPAAMAGETPEL

DGGEVQSSAVFGTAATAAGSPSSADAATAHHGTPTPTPPPPLSLSSSLRLSNSRRRCADH

ARACVGSLSALVLPLRHHAAALQHEVAELRASAVTYSAELLEALALLGRAFNEHETRVVQ

MTEDQATEKAMTVATERVLEAAALQRTAQILKDRFGAALPANAFTPAALPETATDEEWLV

NELHAAAAAMIKESVQPRVGPPTEERGIPVNGGATKVRSTAARPAGQLAQASAAAVVPSR

PPPSRPSRQYAKPVEQDYFETEVLRPFRLQPNFGNGGAARNKAGAPFSDALWAGNDADDE

EEEETASNAATRAAGRAVGCVDGRARNRPALPAQWHWRTRTGYDIGGALHNSAAESRPVL

PPRRPTTFTAATTTVTQSSQTPSPADVQSIQPTVLRYVFGGATESTPCFASRQIQEGTAF

LYGKEFEGFVQEYIFPVISTANRISADGRMDPAMRAAVEDLREKERARRKRGVRLLFDRV

VNSIRTRRLLRRNVYQGEAFVSFVGLLYRNWRTKLERNLRCVQSAQATNRNALFSLLRLK

PFVSPGDAFTKPAPPSSHLPGKTKTGTGTSKPVVNAALVKSSFHFDKAKFNTPS*

>Lp_000076900.1 Domain of unknown function (DUF2431), putative

MESAAASGTGFGLREVSPLLPDPLSILLVGEGNLSFAYSLVRRLSRSAAFRRATQSAANT

LGSATDVVATTYDAEDELAAKYPESAAFRAYFAAKQRVPVRYVGSINATALTSSLADAGV

DVAGHAFQLVVFNNPHIGFEDLYRQRSLISHFFASAAELCRRGPVMCQPQEVVVALCDEQ

AQRWDLLGCAARSGYLCVAAQPLLSTDFPEYVNRRHQSDTAFPFRRMVQYWFVQPAPELL

PTLRELRREIKKWERDRREQSESKKPCSSFDCASWLQMAESLLPDDDASLLSESPSCGAA

LGNNGGCTCLNSSLNYTAGDAEMPMPLLHPSLVARVLPSLLRHDGATTAPSPTADYFTPY

LPSATWVPLYQAQRLMEERSRGCGAAMPAAANRSEAAAAPPHLDAAQLGRPLTMREAKKL

ERYLSGYGAAMRVKARQQKKESAAASASAAKAWVCTDCTPSRTFGTQLDLRQHRTSRHSG

AVQLAPTLYARVHTQIEKSAVPSAGDSLDHALAAMSLEDRQDGYFCDVCGLQYKTSQAYD

EHLHYLSPLPGGGDDAPLLCDLCDPPKRFTDRRGLEQHRYAKHSSTSLP*

>Lp_000077000.1 n-terminal acetyltransferase complex ard1 subunit homolog, putative

MTSYRGMTLCDTLAFNFVNLDQLTETYTTSFYGEYVTHWPEFQRMCIHPTTGIPMAYTLG

KAEGLGEDYHGHVSAVSVAPTFRRVALGETLMVELAQMSEFVHNAYFVDLFVRKSNQVAQ

DMYHRLGYIVYRTVLSYYRGDGPKGPFKGNEDALDMRLALSRDKERRKSSVIPLDRPIKP

EELEWS*

>Lp_000077100.1 beta galactofuranosyl transferase

MILALFLIGYLFPLVCFFRRSLQETFDDAPRKGEAYVSENEFFECVGERLSYKSDHPARI

PYVLVPVTMDYQDLKHLFCNITAPMTYIMLINNGEFKPLRGLLDRLERHLQVYMNKNLFI

IHHPENTGYASAVNEGLRHVITFSVKEVPWVFVTNADVRFGPKLISNFVHVVQDKTKNQE

DRLQQLDAEVAREAEMAAVLPDRRFAYRNSSLPIVTATSLPFRIRVMPYEEMKKQFNGTY

GIFFTNSVEHMATFAISRLLLATVGLFDENYYPAYGEDHDYVWRLEALGFGKYLSPKGQF

IHFENANLDVIADTRTRGITKYTAYSIQGLKFGRMNYQPFRLHYRWSKWFPGNPILDASE

GRKQLPFDGQIPVDMWVLDAKRRNTIWQIGENKLCRRHYQLYNLSVLNFTVAPI*

>Lp_000077200.1 Catalase/Catalase-related immune-responsive, putative

MPGNESPSVAACPMRLTTEFGAPVGNNDDTMTAGRRGPTLLQDVWLLEKLAHFDREEIPE

RRMHAKGSGAFGTFTVTHDITQYTKAKIFSEVGKKTDMFVRFSTVAGEKGAPDLDRDIRG

FAMKFYTEEGNWDMVGNNTPVFYFRDPLRFPDLNHAVKRHPKTNMRSAQIKWDFFTMLPE

ALHQVTIDMSDRGIPADYRHMHGFSSHTYSFINAKNELVWVKLHFKSQQGIKNLSDAEAE

EIAGKDTESSQRDLVDAIERGDYPRWDMKIQVMTQEQAKQCPFNPFDLTKTWSQKEYPLI

DVGVMELNRIPENYFADVEQAAFSPSAVVPGIGFSPDRMLQARLFSYGDAQRYRLGVNYS

TIPVNAPRCPFHSYHRNGAMRVDGNNGANIQYYPNSAGEWLSNPSAVEPALPVDGYAGHY

PHQEDDDDFYSNPRALFELMKPDEQARLFENTARAMQGTTKEVQLRHIHNCMKAHKDYGM

GVAKALGISESEI*

>Lp_000077300.1 hypothetical protein, conserved

MMEATVAQNLGLQLLRQRPKVPQGFDTWAVGVSLPKMANFGQNPCVSALGSYDSNLARLL

SGTVSASDTGTGVAGDTTLSDAFASQPACPSAFSVLTHKCLAVGVRERCTLSQVRIAPEE

FLTRGAAPSAVTREEAAVAVNSFSSAIWEKASETLFYLSLLTLKYETQTP*

>Lp_000077400.1 Leucine Rich repeat, putative

MEGLASCPELTDVDVSNNPTLEGLTSLAGALRVETLKASRCNLRSLEGLSSCTALRVVDV

SDNKNLTSLVGLAGLPCLEKVVAHSCRLTTLHGLRSCPSLTDVDVSRNDFFDVFDVAGAP

HLMRLKKVGGDPTCLSILSTLPVLTELDISCVRGVRDLRMLAGAPLLETLNAKECGLVSV

EGLGQCPRLRTLNVSDNKELSSLAGLAGAPLLETLNARRCGLKNVDGLNSCPELKEVDVS

YNERLTNLDGLAGAPCLKEVNVRGCSVMNTRVLDPSVRVITKGN*

>Lp_000077500.1 hypothetical protein

MHNSKREPFRDLVEPNELYPLDTNGAPVFLPPLPYRAGLGPNGVPQPPTAESGQLVVHDP

SRNGAMLLYDPRKIDPNAQPGMPMTLPSPANGYPRHANAPLVRPPPPKKVTRDHKIIKTR

VTRIYRRGTGGADSENDTLDGEEDEDQVHEHESGSSWETSGSVSWEASSSGSSNNGDADG

EKGSTSDTGAAVAKISHVHRKKHENMSLKLNEAAALTKPCNDDDEGVKREGVNTSVAHHV

KHSRRSKTTTTVCHVDDASEL*

>Lp_000077600.1 60S ribosomal protein L9, putative

MVRVKSHTTLHIPEGVTVEVKGRKVTVTGKRGTLQKDLTHLQLDFRVDKKKRTVTAIRWF

GSKIPVACLNTAKAHIQNMITGVTKGYRFKVRCAYAHFPINVSVDGQNIEVRNFLGEKRV

RRQVVPNTVSVSQTDPAKVKDEIVFDGNDLEQVSREAALLHQMCLVKNKDIRKFLDGIYV

QTKTNVEVEE*

>Lp_000077700.1 hypothetical protein, conserved

MSTCASESPVLSGHVSHLATPTRHRSPFTTADAELSAITAECTDCERLFIGRSSRGGNIA

ANNTPVQMKETAVSPHRRRDTVSPTMTCRASGAGQVTVSPRCRRRAGVSLMDVDALGGLP

RIHLAVPPSGRRHGLERSQLSETSGSLVLRESGA

>Lp_000077800.1 ATP-dependent RNA helicase, putative

MYLCYSTTAAPSHTLHSFGSVVMNGHGSWDAAVESAADTKKKKGGGFQTFGLDKPLLDAV

LKQGFTVPTPIQRKAIPPMLQGNDVVAMARTGSGKTAAFLIPMLHLLKAHSKMVGIRGLV

LSPTRELSLQILHNGFALNKFMDLRFAALVGGDSLDQQFELLASNPDLVVATPGRLLHIM

EEASLHLTAVRCIVLDEADRLFELGLQPQISAIVQKVPESCQRALFSATMPSVLAEFTSA

GLHNPVIIRLDAEMKLSEQLKQSAFLVRNDEKVAALIVLLKGVIHVGEAASNNAQALIFV

ESRFHVEYLQMILAAYDIATSAVHGQMDQEARRLAVRSFAKRETSVMVVTDVAARGLDLP

LLDNVVNFSFPFSPKLFVHRVGRVARAGRSGTAYSIMTFEDFPYYVDLMQFIGRPLQSAQ

TSGDLLFAADDGCYGRLPEEDIQLELDFLKRLHANDVEVRNMAKVVENAHKKFNRTKKKP

THEGIQEARKPQYAFDRTPLHPLLLEKVGSTRVRADEARFDLKRFKPKELFLEMGTKEKL

FEIRHPETVQSLSRSAPTTDHGDEDGHASGRGDERSASTSGGGAGAAGATAAAAVPEKRL

SFAERMLQRAQERKKRERDAGSAGNATADDAIMRLVTHPRQTALSGAEADTMESGAYRDE

NFFMDAERPETLDKAHYSVRDATMDITAETAEEAAQQRQVFAWSKKKNRYVKMHVNDARA

LLKGVKNEAGKAINYKSKLEAYSKWTKKSNMRIQDVGEEEDLVPLKRAKAAAQSALPADG

DGEGDSDYVDISNPNQGKKLRIGRKQKRLPKDGHVRSFEEMALIKRKAEKEKSRLARKKQ

SVGGKRKK*

>Lp_000078000.1 FtsX-like permease family, putative

MPSHGYTDLSDDCRHPLRSSGRSPHAEVMTVEPLLGIINGSNRSHEDDHTGMLERLPTMA

SSRQKEPSKVQAGVMLLEDDSSALSSDRNLLGELKMTVQRNRRLAVDLAYDDESLSLWVR

AKHVLRNVWHSLKMFRLFLSLAWIDAKLRTCSYCLGFFSVFLVVMLCVVMVSLLTNMPIL

FLRLSEALWGEYDLRIEAGGVMSAATSINYTIIKEMFPSSNAAYGLHAPRIVDTFNAQKV

GSCIGKNASSLWYALDGSLCSSEKACMDECSTVNWTPVNLVAIDTAAERRMGFGIHYSQP

TPGEGEVILSHRAALLMGHVDVGDLVAFYGDARPNQRQTFANFTGGITDVIMVFKVIAII

DSDARKFPYMTSFAVANYHTFFRDEAKGLQPGTSADNVAAVANTDPNNCASFVYFIMEPY

TRLRAYRSTNFNTIRRHVSAWASDIISPLGFLQITQQAAQLRGLWSTRMMSVFLGLIISV

VLLALAFLSIVLIYTLLTVGVETKTYELGIQRMIGFTKEDLVVLVLINAYSFTIPAWLLG

LAAGQGVYVGVRQLFMRLIEVRLPLGVTGASIGWATLAGLGIPIVASFFPILALVTQRLP

DALNSSRGRTVGVVFKIRRNDSTELNGTMFGLGVLLFAFGFLIYYLFPTGLIVMNLNLVF

YIFFAVLIGLLAGFVLLALNFERVCQICVSYLLLFWESKAVFSFMQKSLSAHRGRNRKTT

LMYSLSLAFVIFITVAVQIELTAFDYTTRQQLGCDVHVATGSLTLREYWTVEEFLDTYTQ

SGYVTGYTFEYSAPSLMHTTGQRLDSLGRYRTALAVVTALPPNYYEALGSSFLLINHVDR

LVKQYGLVQSLYTEEGMHKSIMSFGAANYLGVGDAEGVLLFGAAQEVNKTSDETAPFMMP

ARPVATMDLTPVQSMSKYSSDSTPVIVAIPSLFHQINDKTSSVLDGVVTSIRLRVRHART

YQQVGDTMTSILSSLGRTATITTITEKTKDMKTANDILNLFFILAELMILIICFFSLMSS

MTTNVLNSSKEIGVLLCMGMTRFQLYRVYVWEAFILVVSSGIVGLIVGVIVAYTMLLQQI

LFVQISFPFPFPYVQLCIVVVVGLVSALASSISPVAYLLGLPSVTHILRRSIT*

>Lp_000078200.1 hypothetical protein, conserved

MYASDPLRRKLWAKSESGGDALASSVHLTPLRKTERATAAQTSPARSNASTTRSPLPPVA

WRTSSSPDFEDTNSPPGRTTVMDPLAALRRCMKYICSHPEVYGNDIRGLLDRELMHVVRL

KASEVSLIQGQRPLLVDPLDSTSLRQASRSFASPGALPSITGTTNTTAAPALGGASTCDD

CVPVGDLFGYGSVSFLPANSAGFSALMQDTRAHAMNSGLKTFSGRDASNGASALPLLPGV

QLMDERIQWNSTLPPSLSAAGAAEADRKSGGGGNRTTAGDADDTVVRSGGDAHVAAAAPV

APAASSSLADYSPTDPYRGLQRPDDAQLFRLVDEPNQTIPPAASLRGEALCTADGTALVL

DHDGYEVTYLRTQLQQLEAAFNAKCVRLHELESENKLFAEQMRASEEAAARWSAEHHAME

GKLEALRRELEGWKDRANDALAAATQQNKQRSQHAKQLASNQINDAKQEAARVSQLWRET

ERALQESRRAFENTEKEASAAHKHLTDAFHYIERLERRVARRDAYVQLCERRHRSLEEKY

EKLMWGYEELSAIEGRYSYVDYLLTTRPLWSVCLFLALAHHRGDYIAVEDQGELKQRLAI

MVAPFTAHSDAAVASKAATVSTDLVDPQSWLLFARTPHGGCYAGSCYDPSLVLRLIVAEV

TAEVMRCGRAKQLRTNLHGSVACRVLRWGERFGIDYLGGQDNFPDSGELNRKFVPVLTLA

SVLLPLRSGGSGGGVFLQNEAPAGEAGTAPSAASVAAPIIPNNRHYDEATVRFVLRCFWK

ERLNAFVRQMETRVAALEAKRQRLERLRAQKQRSSRSVNPIDPGKAPGVQLAEGPSVNRG

QEHAEDEAAEEEEEIDEDATENAATTATFLAALVDFAARFTKVAVNAGDGSAGTSAVAAA

PPGVVDIDVTAAESSSSSSPPPAAADKSTLLRVRGVLARSYSAKATSGGSTSRSKATGNA

VAAPTEAALWNCGRVFFATVLLRRSHTTSGDTAAAATATTSASAGVADVTKSTFNETSAD

AEVAALADDVRELLAALYFYTMEYKSSDADFRLFYLVSHQLIPEMVAVNFFASLEGFQRD

CAALLEKRIQYVAGERDGCGDDVPKSGYVMEPTGTTTAVTFTTSADALEQPVEDPLTCLT

ETVQIIDEAPLDGEDVEIEPDSDGEGYGSIIPFHAKANVHDRAAPVVDAAAPLLGAPSPS

SADYDAHSESARNDSSPSSRGTQRRTRELRLLHNIRAYLKERERGGGVAEADNPQNTSSC

NGGDLLSHSGDVAEDGAELIHNNGSCSSAVASPTAATLPAFAFGDATPEWKALEDAVAER

LAPHTALLDAFRARCVFKRADSASVSSFPSTASRRATATSTGGTARHDKSNSNNVSGTLG

VKEDRGYLRQRHADRKMRYDLTKCLSATRGLLTLDDVLALLKRHCFATYAVSCCGTPRFS

LTDALMEGSAGSVTQRASLSRLAQAATPTTSAAVRPFSIVGYLPPTALQLQRLRFALSLD

QPCDLIDVAKLFTVDAVTKANSHLYDTYLTLTLDLFQQQQSFLMQSVLASCAGRHERYAA

EGAEDCDGQIPIASLRKGMTHALSTVQGSVQHATALVNHFVQYDELMRLEDEVKLEQFAD

APLLLNVPAGLTASPCRKSEANSVMEEVEDAEFNLREEAETCSLLHIAFAVRMTYIVWGR

LCADTAAAVVHRVVQRAVPTCCSNTGDAQFPYSRGDNFSYIRVAEMPEWDIFERGVHQQY

ALTLSRLRDGHNADDNPLRRTLRGEFMKALGLSLPLPYAITAAAGAASATNAAVGCVSFG

GDGAAADAVTLYPDITHAFRYEDTTPAQPASAESDAHAASSSSTGKTKKKERAAAAAATP

HVSASASKRSSAIPAEGEEKAACKLPQLPDYGLISFSRALNFAPSITAAAAVGGGAGPSG

SSAKKGAGKKTAKKPPAKKAGGADSCSLSADVQEEYSRALSMQLQSFATQIARLAQPVKP

LIDEVSGTPEAPPTDGQQLHAAGGPLADAVAARATEPTCIFASPSGDAAAAAAGPTMSSA

ARVTTTRTRSVAFVQDMRSGRGQDDDRLSTVNSNGDALSESGVNAGRPLMWRPVFAEEAR

GAEANGDRTASANPSLDVSKLYAACAALSIL*

>Lp_000078300.1 protein kinase, putative

MPPKLLKRLPPKKTVPPEPPEESDEFEVGNIRVGPRGVEFIGEGDLQVRRPDLNIDNLQR

VRQLGQGTQGNVSMYVTPDKSVFAVKKITIPSTVDNRTRQTVAAELRNIFTAQSNDFTVT

LYNAFYRNGALRLVMEYMDWGNVDELIAEKVKIPEEVAGYIASQMLHALAMLHTKANIVT

EPNQHKSLRQIHRDIKPANVLLSTNGCVKLADFGIATSAETIGVNSFVGTATYMSPERIQ
[truncated: 6,048,459 more chars]
